# Supplementary material for: Enantiodivergence by minimal modification of an acyclic chiral secondary aminocatalyst
Source: Nat Commun. 2019 Nov 15;10:5182. doi: 10.1038/s41467-019-13183-5 (PMC6858435; doi:10.1038/s41467-019-13183-5)
Supplement: Supplementary file 2 — Description of Additional Supplementary Files [file 41467_2019_13183_MOESM2_ESM.pdf]

Description of Additional Supplementary Files

File Name: Supplementary Data 1

Description: B3LYP Geometries for the Relative Free Energy of the Enamines Species in Supplementary Figure 6.

File Name: Supplementary Data 2

Description: B3LYP Geometries for All the Optimized Compounds and Transition States in Supplementary Figure 7.

File Name: Supplementary Data 3

Description: B3LYP Geometries for All the Optimized Compounds and Transition States in Supplementary Figure 8.

File Name: Supplementary Data 4

Description: B3LYP Geometries for Whole Free Energy Profiles for Ia Catalyzed Mannich Reaction of 1a and 2a in Supplementary Figure 9-11.
