# Supplementary material for: Enantiodivergence by minimal modification of an acyclic chiral secondary aminocatalyst
Source: Nat Commun. 2019 Nov 15;10:5182. doi: 10.1038/s41467-019-13183-5 (PMC6858435; doi:10.1038/s41467-019-13183-5)
Supplement: Supplementary file 3 — Supplementary Data 1 [file 41467_2019_13183_MOESM3_ESM.pdf]

Supplementary Data 1

Ia-int-I

C	-4.33042400	-1.15843800	-2.09097700
H	-3.65485200	-1.73622600	-2.73103600
H	-4.79262600	-0.38642700	-2.71463300
C	-3.50460900	-0.42818700	-1.00076400
H	-4.21681400	0.07815100	-0.34449700
C	-2.70755300	-1.44265900	-0.15926100
H	-2.04898700	-2.03772800	-0.79470400
H	-3.36401600	-2.11994400	0.38419800
N	-2.61530800	0.61949200	-1.51444800
N	-1.83045100	-0.71839800	0.83009300
C	-0.57907800	-1.50533100	1.15261900
C	0.37615000	-0.74719800	2.06048000
H	-0.11049500	-1.70973000	0.18741200
H	-0.90063200	-2.45376800	1.58696100
H	1.30996200	-1.31521000	2.12502900
H	0.60920600	0.24248900	1.65125100
H	-0.01541300	-0.62851000	3.07462200
C	-2.58821800	-0.21662000	2.04823000
C	-2.97440100	-1.31071500	3.03074800
H	-3.47087300	0.29593200	1.66284400
H	-1.94758100	0.53244700	2.51597800
H	-3.55806400	-0.84750600	3.83315300
H	-3.59830800	-2.08188100	2.56851300
H	-2.10133800	-1.78752300	3.48520000
C	-3.02181200	2.00542300	-1.26725200
C	-4.28233300	2.49129600	-2.01318900
H	-2.17441600	2.65671200	-1.50430200
H	-3.19853800	2.10380200	-0.18806900
H	-5.10717400	1.81668800	-1.74109900
C	-4.64415100	3.90098800	-1.52606400
H	-3.84066700	4.61265700	-1.75934900
H	-5.55871200	4.26039800	-2.01272100
H	-4.80871400	3.92285700	-0.44138800
C	-4.12467000	2.46100800	-3.53758200
H	-3.86191100	1.46210800	-3.89932800
H	-5.05893800	2.76415300	-4.02679000
H	-3.33702800	3.15233400	-3.86328800
C	-5.39512900	-2.05698800	-1.50183100
C	-6.57614700	-1.50244000	-0.98381600
C	-5.21275800	-3.44464500	-1.41574500
C	-7.54820600	-2.31246400	-0.39340800
H	-6.73206200	-0.42746800	-1.04801600
C	-6.18348700	-4.25926500	-0.82550700
H	-4.30362900	-3.88884600	-1.81472200
C	-7.35402500	-3.69529300	-0.31120000
H	-8.45795300	-1.86573600	-0.00024700
H	-6.02505600	-5.33329100	-0.76997600
H	-8.11048900	-4.32727900	0.14663500
C	-1.68009600	0.28714200	-2.49696200
C	-0.95463800	1.09953700	-3.28787000
H	-1.51259500	-0.78523200	-2.57579800
H	-1.10053900	2.17663200	-3.26158800
H	-1.52449600	0.12529900	0.31798800
C	0.08571000	0.58389800	-4.24496700
H	0.14864000	-0.51133400	-4.22210500
H	1.08765800	0.97651800	-4.01469500
H	-0.13077200	0.88179700	-5.28165600

Ia-int-II

C	-4.51524300	-0.30145000	-1.08643300
H	-4.71031200	-1.10952500	-0.37315800
H	-4.62729300	-0.71853500	-2.09305900
C	-3.04850400	0.17278600	-0.96036500
H	-2.92829900	1.01011700	-1.65364700
C	-2.77992400	0.67188600	0.47327700
H	-2.89623500	-0.14664700	1.18544800

H	-3.46708200	1.46746600	0.75796900
N	-2.08691100	-0.85331300	-1.38050900
N	-1.37397100	1.20131700	0.64334500
C	-0.93336400	1.11364600	2.09185200
C	0.50403000	1.56294100	2.29952300
H	-1.05060300	0.06224800	2.36336500
H	-1.64269700	1.70301700	2.67576400
H	0.78742300	1.33470200	3.33219500
H	1.18700100	1.02284700	1.63366000
H	0.63350800	2.63766500	2.14510700
C	-1.17242000	2.57156700	0.01521400
C	-1.86444400	3.70302400	0.75750900
H	-1.53898800	2.49184300	-1.00852300
H	-0.09357300	2.72483700	-0.03212000
H	-1.68298200	4.62609700	0.19709200
H	-2.94741900	3.55932200	0.81726900
H	-1.46763800	3.84156100	1.76729000
C	-2.11461000	-2.15462100	-0.70558200
C	-2.73531300	-3.29269200	-1.54120900
H	-2.68562900	-2.05963500	0.22381900
H	-1.09730400	-2.43189600	-0.40299900
H	-3.76412900	-2.99438500	-1.78452200
C	-1.98746300	-3.53499000	-2.85688400
H	-0.95749200	-3.86421000	-2.66958000
H	-2.48641500	-4.31480200	-3.44570500
H	-1.94571100	-2.62654200	-3.46700000
C	-2.79183300	-4.57063400	-0.69427300
H	-3.36719500	-4.41747200	0.22768100
H	-3.26129500	-5.39036100	-1.25106200
H	-1.78212000	-4.89621600	-0.40942400
C	-5.50396000	0.82568300	-0.87507100
C	-5.68100500	1.80416300	-1.86559300
C	-6.22906900	0.94162600	0.31899100
C	-6.55867700	2.87181300	-1.66753500
H	-5.12739800	1.72357000	-2.79875600
C	-7.10891500	2.00904600	0.52163100
H	-6.10215900	0.19007000	1.09498100
C	-7.27485700	2.97863400	-0.47073600
H	-6.68525800	3.61905900	-2.44686700
H	-7.66343400	2.08169000	1.45377900
H	-7.95865900	3.80895900	-0.31532100
C	-0.91349700	-0.39410200	-1.98445600
C	0.28359500	-1.00263000	-2.10600800
H	-1.03372800	0.59810400	-2.41882700
H	0.44574500	-2.00202900	-1.71067400
H	-0.75160500	0.56644000	0.12230500
C	1.44097000	-0.37169900	-2.83124300
H	2.33196800	-0.30190600	-2.19041300
H	1.19911400	0.64212400	-3.17377300
H	1.74125000	-0.95636300	-3.71376600

TS-rotation-Ia

C	-4.22323100	-0.75227100	0.30121200
H	-4.11174000	-1.11887700	1.32823600
H	-4.59323900	-1.58600100	-0.29865700
C	-2.83405500	-0.31617000	-0.23562700
H	-2.99558000	0.13895600	-1.22573900
C	-2.30815800	0.74174800	0.75600100
H	-1.97566100	0.22420900	1.65840400
H	-3.09506500	1.44372700	1.02635900
N	-1.87824400	-1.43600500	-0.31897400
N	-1.13809500	1.58991200	0.30416500
C	-0.58248500	2.35335700	1.49237800
C	0.64411800	3.18284300	1.14795000
H	-0.33374400	1.58822200	2.23060800
H	-1.39602100	2.96358000	1.88884000
H	1.07763700	3.55346000	2.08256900
H	1.40301200	2.57673600	0.63974700
H	0.40388800	4.04804200	0.52380000
C	-1.43239000	2.45554400	-0.90691300
C	-2.50155200	3.51068000	-0.67251700
H	-1.72306000	1.77298500	-1.70549700
H	-0.48106000	2.90616600	-1.19175400
H	-2.60042600	4.09329400	-1.59434800
H	-3.47804700	3.06906300	-0.45326900
H	-2.23318500	4.20166300	0.13195300
C	-2.37863200	-2.76010200	-0.74244300
C	-3.03711100	-2.89076800	-2.13563600
H	-3.08586700	-3.11249400	0.01635400
H	-1.51952700	-3.43847300	-0.69772300
H	-3.88314500	-2.19194400	-2.18950700

C -2.08398800 -2.56043700 -3.29176800
H -1.16524600 -3.15834700 -3.23162300
H -2.56110100 -2.77547400 -4.25632300
H -1.79667300 -1.50445100 -3.29793700
C -3.59290400 -4.31557300 -2.28044600
H -4.30309000 -4.55463800 -1.47882700
H -4.11308500 -4.43962900 -3.23779100
H -2.78312200 -5.05700700 -2.24292500
C -5.24208100 0.36737700 0.24402900
C -5.68468500 0.85360800 -0.99687100
C -5.74846600 0.95454800 1.41147900
C -6.60357600 1.90240900 -1.06832600
H -5.30544600 0.40436000 -1.91205400
C -6.67156400 2.00290900 1.34459600
H -5.41386400 0.58941100 2.37973700
C -7.10050900 2.48154600 0.10433600
H -6.93500100 2.26486700 -2.03807700
H -7.05349800 2.44438700 2.26154400
H -7.81693000 3.29701200 0.04999100
C -0.62787300 -1.05667800 -0.88662100
C 0.55724300 -1.49398700 -0.43747600
H -0.65830100 -0.37401400 -1.74399200
H 0.57339700 -2.16275000 0.42347200
H -0.39535800 0.93702500 0.01508600
C 1.87365100 -1.15449300 -1.07043200
H 2.56614600 -0.72495700 -0.33379200
H 1.75488100 -0.43749300 -1.89115700
H 2.36402400 -2.05171900 -1.47368600

Ik-int-I

C -4.44896500 -1.07065100 -1.97865400
H -3.78502800 -1.55615200 -2.70194100
H -4.97648500 -0.26669900 -2.50535300
C -3.59784900 -0.39954500 -0.87127700
H -4.29292500 0.06239800 -0.16711500
C -2.77133300 -1.44757800 -0.10570800
H -2.13216600 -2.01237600 -0.78700200
H -3.40972300 -2.15147100 0.42527900
N -2.73301800 0.68188000 -1.35677700
N -1.86595900 -0.76940400 0.89179400
C -0.62320100 -1.58824400 1.16417000
C 0.35768300 -0.88386300 2.08776000
H -0.17147400 -1.76025800 0.18458100
H -0.95353100 -2.54855300 1.56499900
H 1.28412500 -1.46695000 2.11157700
H 0.59869200 0.11948900 1.71838000
H -0.01478700 -0.80484800 3.11288700
C -2.59215800 -0.29391200 2.13851700
C -3.00209600 -1.41421200 3.08059200
H -3.46059500 0.26452300 1.78730500
H -1.92106900 0.41360600 2.62744300
H -3.54969200 -0.96482800 3.91557200
H -3.66601700 -2.13900800 2.59969300
H -2.13902500 -1.94411900 3.49328200
C -3.17573800 2.06541100 -1.16417500
C -2.25852600 2.85541400 -0.22068500
H -4.19105900 2.04928200 -0.75528400
H -3.23901600 2.56982300 -2.13856600
H -1.22949100 2.81190100 -0.59833800
C -2.70272600 4.31234000 -0.07393900
H -3.72693800 4.37684000 0.31563600
H -2.04831200 4.85994400 0.61413100
H -2.68058900 4.83136900 -1.04065300
C -5.43776600 -2.07160200 -1.42338900
C -6.57988300 -1.63252700 -0.73549300
C -5.21388300 -3.45098700 -1.53795700
C -7.47498500 -2.54761100 -0.17760600
H -6.76702500 -0.56487300 -0.64081200
C -6.10792200 -4.37049500 -0.98178000
H -4.33309100 -3.80586700 -2.06843200
C -7.24116200 -3.92147600 -0.29860800
H -8.35610600 -2.18928000 0.34870500
H -5.91862500 -5.43613900 -1.08365700
H -7.93802900 -4.63484600 0.13346300
C -1.80015200 0.39987200 -2.35749100
C -1.09632900 1.27349500 -3.10265700
H -1.62773300 -0.66406700 -2.50444100
H -1.22661700 2.34536900 -2.97141800
H -1.55812500 0.08892700 0.40629500
C -0.09008300 0.83963000 -4.13385400
H -0.02690100 -0.25361200 -4.20128200

H	0.91825300	1.21700400	-3.90699900
H	-0.34311600	1.21885600	-5.13493300
H	-2.25797600	2.35984000	0.75993500

Ik-int-II

C	-4.62680200	-0.25705000	-1.02559600
H	-4.84501500	-1.03994800	-0.29141800
H	-4.77913300	-0.68846500	-2.02115500
C	-3.13905400	0.15848000	-0.94209000
H	-3.01371800	1.00171900	-1.62726600
C	-2.79646400	0.61219700	0.48852200
H	-2.83933900	-0.23996800	1.16921600
H	-3.48855300	1.37046200	0.85275600
N	-2.23018100	-0.89360600	-1.41897400
N	-1.40006900	1.18466400	0.59342900
C	-0.88312900	1.08158700	2.01475900
C	0.54233100	1.58761400	2.16350700
H	-0.94320400	0.01970400	2.26301700
H	-1.58542500	1.62458400	2.64976700
H	0.89042100	1.33486500	3.17029000
H	1.21178800	1.10412000	1.44275900
H	0.61581600	2.67228000	2.04545300
C	-1.27900400	2.57112800	-0.01830400
C	-2.00540700	3.66084900	0.75400300
H	-1.66627700	2.48689900	-1.03398300
H	-0.21022100	2.77641700	-0.09033500
H	-1.85061600	4.60340500	0.21840400
H	-3.08386400	3.48499500	0.80756600
H	-1.61310700	3.78478700	1.76762500
C	-2.19667800	-2.16175400	-0.68319300
C	-2.25659200	-3.37942200	-1.61401600
H	-3.05373100	-2.19527100	-0.00630600
H	-1.29887300	-2.21370300	-0.04599500
H	-3.20682100	-3.34981900	-2.16236900
C	-5.55551700	0.92076600	-0.81475600
C	-5.70699800	1.88907600	-1.81956000
C	-6.24200600	1.09725800	0.39461800
C	-6.52116600	3.00569100	-1.62035600
H	-5.18228500	1.76268100	-2.76427700
C	-7.05787700	2.21416500	0.59877600
H	-6.13445000	0.35453600	1.18197300
C	-7.19858800	3.17285900	-0.40795500
H	-6.62826300	3.74424200	-2.41081800
H	-7.58278900	2.33363900	1.54316400
H	-7.83223700	4.04179600	-0.25107800
C	-1.04335600	-0.44314900	-2.00814200
C	0.17189800	-1.02852200	-1.99991400
H	-1.16915100	0.49715700	-2.54489500
H	0.33083400	-1.96996500	-1.47992200
H	-0.78469900	0.58161500	0.02571700
C	1.35501300	-0.45793500	-2.73262600
H	2.20869000	-0.30166200	-2.05777700
H	1.11674100	0.50647200	-3.19770800
H	1.70878200	-1.13116200	-3.52747900
H	-1.45813600	-3.30962500	-2.36173700
C	-2.13230300	-4.69358200	-0.83999000
H	-2.92907100	-4.79028000	-0.09101500
H	-2.19910700	-5.55709000	-1.51181900
H	-1.17156400	-4.75390800	-0.31265600

TS-rotation-Ik

C	-1.28156200	1.37742100	-1.05420800
H	-1.49493300	2.35579100	-0.60826400
H	-2.11441100	0.71822800	-0.79561800
C	0.01480300	0.80785800	-0.41443000
H	0.30048600	-0.07574500	-0.99889500
C	1.10362300	1.87316800	-0.56982400
H	0.97042400	2.65248600	0.18269800
H	1.03735800	2.33309100	-1.55476900
N	-0.23575900	0.44643000	1.00372100
N	2.53213700	1.38908700	-0.43393500
C	3.47437800	2.57666400	-0.47499800
C	4.91016000	2.21172700	-0.13083800
H	3.07666900	3.28422300	0.25599400
H	3.38597200	3.02157800	-1.46770800
H	5.47553700	3.13998800	0.00109200
H	4.96107700	1.64922100	0.80905800
H	5.39916300	1.63246600	-0.91877900
C	2.90938800	0.25965100	-1.37367600
C	2.72012500	0.58422900	-2.84695000
H	2.30483100	-0.59792800	-1.07497200
H	3.94793400	0.01728700	-1.14743400

H 3.08261400 -0.27122000 -3.42645900
H 1.66829900 0.73888300 -3.10286300
H 3.29433900 1.46331000 -3.15515200
C -1.17197900 -0.68656700 1.18354600
C -0.83756300 -1.99285100 0.44401300
H -2.17933800 -0.36201600 0.89844400
H -1.21343600 -0.88181100 2.26127900
H 0.19578200 -2.29145700 0.66474400
C -1.18812000 1.47731200 -2.56076700
C -1.19614700 0.31026600 -3.34200200
C -1.05145500 2.71350700 -3.20721100
C -1.06438300 0.37622200 -4.73023300
H -1.30516300 -0.65566300 -2.85313100
C -0.92130100 2.78466600 -4.59788600
H -1.04554200 3.62630700 -2.61572900
C -0.92466700 1.61606600 -5.36334100
H -1.07256100 -0.53820100 -5.31802300
H -0.81675800 3.75280900 -5.08107500
H -0.82246900 1.66941800 -6.44404900
C 0.91422900 0.28436200 1.83832400
C 1.41635200 1.26866900 2.59809500
H 1.37930800 -0.70790500 1.86935800
H 0.92155300 2.23963300 2.58415700
C 2.64226200 1.13486100 3.45250400
H 3.05684300 0.12135700 3.41044900
H 2.41724400 1.37401400 4.50063400
H 3.42451000 1.83713500 3.13276100
H 2.60465000 0.99875000 0.51614500
H -0.89401100 -1.83266800 -0.63988200
C -1.79990800 -3.11733000 0.83723700
H -1.57371200 -4.04260600 0.29441200
H -1.73966300 -3.33489400 1.91122100
H -2.83879600 -2.84420700 0.61124000

IL-int-I

C -4.31538300 -1.13878800 -2.02369100
H -3.63360300 -1.70582700 -2.66654100
H -4.77357900 -0.35949200 -2.64084200
C -3.50074400 -0.42840800 -0.91202900
H -4.21709900 0.06333800 -0.24912600
C -2.70520800 -1.46167600 -0.09250700
H -2.05527800 -2.04926100 -0.74409900
H -3.36210300 -2.14480100 0.44305100
N -2.59741400 0.63178300 -1.38767800
N -1.81651700 -0.75886500 0.90076000
C -0.56599000 -1.55609300 1.19785700
C 0.39925600 -0.82151000 2.11442500
H -0.10494600 -1.74232400 0.22527400
H -0.88720200 -2.51247200 1.61516600
H 1.33133800 -1.39429100 2.15937100
H 0.63302800 0.17614100 1.72549600
H 0.01615800 -0.72395500 3.13403200
C -2.56090000 -0.27252700 2.13232000
C -2.95237100 -1.38104500 3.09625400
H -3.44064900 0.25619000 1.76261400
H -1.90988000 0.46093800 2.61037600
H -3.52278300 -0.92732300 3.91345700
H -3.59008000 -2.13496600 2.62448600
H -2.08116300 -1.87781700 3.53251000
C -3.05991800 2.01405900 -1.21867800
C -4.15258500 2.45692400 -2.19841300
H -2.18474400 2.66613800 -1.30758100
H -3.42205000 2.11300000 -0.18962700
H -5.07801400 1.89314000 -2.03709500
C -5.38336100 -2.05033900 -1.46006500
C -6.56749200 -1.50969200 -0.93453200
C -5.20119500 -3.43974700 -1.40883100
C -7.54301400 -2.33516900 -0.37185100
H -6.72393800 -0.43360400 -0.97098500
C -6.17546500 -4.26982200 -0.84663900
H -4.28980500 -3.87351200 -1.81407100
C -7.34942600 -3.71973300 -0.32528700
H -8.45526000 -1.89891700 0.02723700
H -6.01708700 -5.34491400 -0.81867200
H -8.10885600 -4.36363400 0.11047200
C -1.72590700 0.32644400 -2.44109800
C -1.12941600 1.17441500 -3.29868000
H -1.49523300 -0.73463600 -2.51317600
H -1.34883700 2.23936600 -3.26732700
H -1.51817900 0.09272800 0.39380900
C -0.12605500 0.72603400 -4.32603600

H 0.01497300 -0.36170500 -4.30670600
H 0.85866300 1.19016500 -4.16577600
H -0.43556300 1.00438600 -5.34397600
H -3.83799900 2.30969700 -3.23665100
H -4.37604700 3.52090100 -2.05493600

IL-int-II

C -4.45154800 -1.25869700 -2.03737900
H -3.77140300 -1.81494100 -2.69098700
H -4.96636400 -0.51310600 -2.65313200
C -3.63061600 -0.48260700 -0.97629300
H -4.35010700 -0.00801500 -0.30370600
C -2.77273000 -1.46085600 -0.15251600
H -2.10938400 -2.03528300 -0.80130500
H -3.39131600 -2.16000800 0.40733000
N -2.79826500 0.59691200 -1.51151700
N -1.89854000 -0.70505000 0.81493500
C -0.61934500 -1.45542300 1.11761100
C 0.33612900 -0.66239200 1.99460500
H -0.16765200 -1.65997300 0.14440500
H -0.90633400 -2.40613100 1.57077900
H 1.28444800 -1.20713000 2.04666600
H 0.53593800 0.32709300 1.56771400
H -0.03718500 -0.53986300 3.01513300
C -2.64742800 -0.21798400 2.04486200
C -2.99181300 -1.31737000 3.03682800
H -3.54758200 0.27342700 1.67295600
H -2.01643600 0.54676800 2.50000700
H -3.57602800 -0.86512300 3.84505200
H -3.60200300 -2.10676700 2.58740900
H -2.10028200 -1.76858900 3.48164100
C -1.81275800 0.31066200 -2.55923800
C -2.31351100 0.49825500 -3.99824600
H -1.46277200 -0.71666100 -2.42582000
H -0.94495000 0.95821800 -2.38309000
H -2.73357800 1.49743000 -4.14685600
C -5.45711900 -2.19869300 -1.40790800
C -6.62407100 -1.68952500 -0.81683400
C -5.22971200 -3.58130200 -1.35937500
C -7.53894600 -2.53916700 -0.19190600
H -6.81436200 -0.61881200 -0.85032700
C -6.14328800 -4.43558100 -0.73475200
H -4.33004100 -3.99008000 -1.81385000
C -7.30035700 -3.91675600 -0.14781300
H -8.43881600 -2.12716200 0.25781000
H -5.95068700 -5.50512900 -0.70864600
H -8.01228800 -4.57953600 0.33703700
C -3.19517600 1.91565000 -1.28461200
C -2.68273300 3.04356600 -1.81327200
H -3.99942600 1.99558800 -0.55517400
H -1.88382900 3.00012900 -2.54993900
H -1.62802800 0.14697300 0.29737700
C -3.16217600 4.41544100 -1.42214200
H -3.96933500 4.36552400 -0.68059500
H -3.54181200 4.97832300 -2.28777300
H -2.35443200 5.02462900 -0.98933300
H -3.08238200 -0.23629400 -4.25412900
H -1.47792800 0.37355700 -4.69745300

TS-rotation-IL

C -1.28513600 1.37383600 -1.06160900
H -1.49930200 2.35163000 -0.61476900
H -2.11966900 0.71513200 -0.80727500
C 0.00878000 0.80312600 -0.41755500
H 0.29496200 -0.08299300 -0.99866900
C 1.09882400 1.86789500 -0.57124200
H 0.96605900 2.64499100 0.18366100
H 1.03281900 2.33099800 -1.55459400
N -0.24503900 0.44704500 1.00135600
N 2.52715500 1.38303400 -0.43764400
C 3.46887200 2.57147500 -0.46848900
C 4.90613800 2.20368100 -0.13389800
H 3.07331400 3.27032600 0.27202500
H 3.37650500 3.02773700 -1.45569100
H 5.47123500 3.13089000 0.00646100
H 4.96104700 1.63061900 0.79936800
H 5.39265000 1.63394700 -0.93028700
C 2.90506500 0.26186900 -1.38700500
C 2.71812300 0.60064000 -2.85737700
H 2.29945100 -0.59805000 -1.09731800
H 3.94312400 0.01676400 -1.16152900
H 3.08174500 -0.24906700 -3.44458300

H 1.66672500 0.75755300 -3.11374500
H 3.29276400 1.48271400 -3.15610100
C -1.17849600 -0.68995600 1.18025800
C -0.82125700 -1.99291700 0.45195100
H -2.18236200 -0.37078500 0.88081800
H -1.22916800 -0.87437800 2.25840600
H 0.19641400 -2.32498400 0.68850900
C -1.18601600 1.47683200 -2.56763500
C -1.19161600 0.31177400 -3.35179200
C -1.04601900 2.71456100 -3.21051000
C -1.05402400 0.38113000 -4.73932500
H -1.30341900 -0.65533800 -2.86586200
C -0.90996500 2.78914000 -4.60041000
H -1.04196200 3.62589700 -2.61676000
C -0.91078400 1.62243100 -5.36876300
H -1.06033600 -0.53180200 -5.32944600
H -0.80287900 3.75843600 -5.08072600
H -0.80394400 1.67841600 -6.44888800
C 0.90737400 0.28197000 1.83280400
C 1.40488700 1.26158600 2.60125400
H 1.37907200 -0.70755800 1.85261700
H 0.90481600 2.22994800 2.59679700
C 2.63207900 1.12613000 3.45352200
H 3.05327300 0.11590300 3.39983600
H 2.40620800 1.35211100 4.50439000
H 3.40935900 1.83715300 3.14122600
H 2.59974000 0.98514200 0.50916400
H -1.51323600 -2.78613700 0.75843300
H -0.90038000 -1.88888000 -0.63585200

Ib-int-I

C -2.23876600 1.83749000 0.07460700
H -1.32907100 2.16608100 -0.44407500
H -1.94081800 1.56848400 1.09528000
C -2.75989600 0.55128200 -0.59800500
H -3.61123900 0.18997300 -0.01970200
C -3.23234100 0.86721000 -2.03452400
H -2.46294400 1.42729400 -2.56974700
H -4.15373900 1.45135800 -2.03945800
N -1.77061400 -0.53708100 -0.57655800
N -3.49084900 -0.37976100 -2.83978000
C -2.12129400 -1.82397400 0.02353900
H -3.15061700 -1.75384900 0.39097900
C -0.67136000 -0.45557300 -1.42786900
C 0.13691000 -1.45253500 -1.84466900
H -0.47292800 0.55284800 -1.78540100
H -0.01414000 -2.47181000 -1.49736300
H -2.69717200 -1.01156900 -2.66138600
C 1.30577000 -1.22138600 -2.76272500
H 1.23322100 -1.83177600 -3.67470500
H 2.26033100 -1.49035700 -2.28703300
H 1.37607800 -0.17113600 -3.07186400
H -2.11483300 -2.61117500 -0.74832100
C -3.23290100 3.01570100 0.13986400
C -2.57012100 4.20543800 0.84855200
C -4.54527700 2.63069400 0.83844200
H -3.47045100 3.33326300 -0.88589900
H -1.64073600 4.50176400 0.34666900
H -3.23661300 5.07626000 0.86516500
H -2.32381900 3.95279000 1.88839300
H -5.09805400 1.85868800 0.28956800
H -4.35012000 2.24788900 1.84904300
H -5.20576900 3.50093000 0.93226500
C -4.75322700 -1.10241400 -2.42122800
C -3.63213600 -1.40306000 -5.11346700
C -4.89044400 -2.17723500 -4.70338200
C -4.89703100 -2.41269900 -3.18848900
C -3.49183000 -0.10194100 -4.32875000
H -3.64987800 -1.15167500 -6.17923900
H -4.69556000 -1.26671300 -1.34554800
H -5.78311800 -1.60437200 -4.98807000
H -4.93446100 -3.13379900 -5.23497300
H -5.82811200 -2.88976600 -2.86470900
H -4.07521200 -3.08499400 -2.90677300
H -2.55298400 0.40890600 -4.55235200
H -2.73873000 -2.01874200 -4.94080800
H -5.57565200 -0.41358600 -2.63371600
H -4.32553200 0.58184100 -4.51128600
C -1.21446500 -2.25051500 1.19554500
C -1.61927900 -3.65385200 1.66417000
C -1.27443300 -1.23916800 2.34487300

H -0.18039000 -2.29307000 0.83087500
H -1.55172200 -4.38694400 0.85035400
H -0.97147400 -3.99751900 2.47900900
H -2.65301400 -3.66034700 2.03627200
H -0.94121500 -0.24827600 2.01943600
H -2.29867700 -1.14242600 2.73124100
H -0.63208800 -1.55315300 3.17639600

Ib-int-II

C -4.59370200 -0.31720600 -1.55208200
H -4.71494300 -1.31153600 -1.10486300
H -4.64861700 -0.45240900 -2.63985900
C -3.18365700 0.21543800 -1.24186100
H -3.07161800 1.17690100 -1.75025900
C -3.00912600 0.43983300 0.27581300
H -3.19972600 -0.48608200 0.82299700
H -3.68075200 1.21056200 0.65804900
N -2.13078300 -0.65550800 -1.78777500
N -1.60416200 0.86920000 0.62215000
C -2.08291300 -2.05295300 -1.34333600
H -2.70902600 -2.16011800 -0.45148200
H -1.06321900 -2.30362700 -1.02414800
C -0.97087800 -0.02305000 -2.24591300
C 0.27845200 -0.51408700 -2.37445700
H -1.14911300 1.01108600 -2.53933500
H 0.50118700 -1.54720000 -2.12211500
H -0.96439300 0.34037800 0.00976300
C 1.41516100 0.29994100 -2.93017200
H 2.26502700 0.33723000 -2.23372100
H 1.11105100 1.33284800 -3.13980800
H 1.80262700 -0.12617000 -3.86747400
C -5.77031700 0.57170500 -1.09681000
C -7.09129400 -0.06630500 -1.55067100
C -5.65708800 2.01097600 -1.61850900
H -5.78102000 0.60542900 0.00233000
H -7.19313500 -1.09061700 -1.17109400
H -7.95194700 0.51193500 -1.19371100
H -7.14694100 -0.10834200 -2.64646100
H -4.78508600 2.53471000 -1.20847000
H -5.57207500 2.02361500 -2.71325100
H -6.54529600 2.59340300 -1.34590500
C -1.35619600 2.33777400 0.33999700
C 0.23021400 0.86174200 2.31572000
C 0.53184300 2.33560200 2.01757800
C 0.10880400 2.68530400 0.58590500
C -1.23290800 0.52162100 2.04685800
H 0.44485100 0.61680400 3.36155700
H -1.65336300 2.52224500 -0.69323800
H -0.01528800 2.97236500 2.72571600
H 1.59919200 2.53734400 2.15641000
H 0.23892700 3.75359000 0.38284300
H 0.73112100 2.14022800 -0.13699000
H -1.43160100 -0.54458200 2.17145000
H 0.86576000 0.21560700 1.69465800
H -2.02531700 2.88842100 1.00694700
H -1.91272300 1.09032000 2.68787100
C -2.56149100 -3.07445000 -2.39491900
C -2.55528100 -4.47923000 -1.77803900
C -1.72929700 -3.03219100 -3.68125800
H -3.59736500 -2.81409600 -2.65124800
H -3.18444300 -4.52970200 -0.88009900
H -2.93040900 -5.22216800 -2.49162700
H -1.53789400 -4.77793200 -1.49136700
H -1.73217400 -2.03260500 -4.12780900
H -0.68624800 -3.31206200 -3.48603200
H -2.13041700 -3.73474900 -4.42214400

TS-rotation-Ib

C -2.84813800 1.59931000 -1.98275600
H -3.75570400 1.43525700 -2.57897500
H -2.07341800 1.93031100 -2.67744300
C -2.44746500 0.25652100 -1.33419500
H -1.65736900 0.45484100 -0.59445100
C -3.68826500 -0.31563800 -0.58778500
H -4.08983400 -1.16481900 -1.14126600
H -4.48222300 0.42526500 -0.48234200
N -1.94612400 -0.73212700 -2.31663700
N -3.41172200 -0.79654600 0.82143700
C -1.49126300 -0.32077200 -3.64433800
H -2.24882800 0.33217400 -4.08695500
H -1.47355100 -1.21403700 -4.27702000
C -1.61582300 -1.99653000 -1.83943800

C -1.05891200 -3.04954700 -2.47068600
H -1.87372500 -2.14832500 -0.79454400
H -0.74560600 -2.97815600 -3.50876100
H -2.52357600 -1.31284100 0.82029000
C -0.81994900 -4.36992600 -1.78995600
H -1.34336300 -5.19400800 -2.29759500
H -1.16204100 -4.35775500 -0.74703200
H 0.24633600 -4.64121300 -1.78347000
C -3.07841400 2.76037800 -0.99164900
C -3.77270900 3.92176400 -1.71785200
C -1.76295300 3.23924900 -0.36021400
H -3.74787800 2.42934900 -0.18671900
H -4.74736900 3.61756900 -2.11888900
H -3.93564900 4.76827900 -1.03987800
H -3.16109600 4.27883200 -2.55693900
H -1.22862600 2.43533100 0.15917600
H -1.08921700 3.63755700 -1.13048800
H -1.94703000 4.03866000 0.36733400
C -3.24620100 0.34593900 1.80167000
C -4.15977100 -2.29747000 2.68231900
C -3.96651600 -1.16160500 3.69447200
C -2.90068300 -0.18141700 3.19060300
C -4.48062700 -1.76370200 1.28926200
H -4.97894300 -2.96045600 2.98087700
H -2.47070800 1.00244500 1.40592600
H -4.91645400 -0.62815100 3.83355000
H -3.67657100 -1.56977700 4.66828700
H -2.80646100 0.68082200 3.85928300
H -1.91890300 -0.67282300 3.15790800
H -4.52624900 -2.56331500 0.54665400
H -3.25044300 -2.91159600 2.62900200
H -4.19901700 0.88181300 1.79832200
H -5.41983800 -1.20395200 1.27071600
C -0.11578300 0.38309200 -3.72239500
C 0.11628400 0.86759800 -5.16007000
C 1.04682200 -0.49613400 -3.24713900
H -0.15081300 1.26563300 -3.06818000
H -0.67889500 1.54810800 -5.49014700
H 1.07064900 1.40041900 -5.24698000
H 0.14343800 0.02075800 -5.85915200
H 0.90190200 -0.83899700 -2.21803800
H 1.15612000 -1.38366800 -3.88254800
H 1.99014000 0.06320600 -3.28786700

Im-int-I

C -2.23876600 1.83749000 0.07460700
H -1.32907100 2.16608100 -0.44407500
H -1.94081800 1.56848400 1.09528000
C -2.75989600 0.55128200 -0.59800500
H -3.61123900 0.18997300 -0.01970200
C -3.23234100 0.86721000 -2.03452400
H -2.46294400 1.42729400 -2.56974700
H -4.15373900 1.45135800 -2.03945800
N -1.77061400 -0.53708100 -0.57655800
N -3.49084900 -0.37976100 -2.83978000
C -2.12129400 -1.82397400 0.02353900
H -3.15061700 -1.75384900 0.39097900
C -0.67136000 -0.45557300 -1.42786900
C 0.13691000 -1.45253500 -1.84466900
H -0.47292800 0.55284800 -1.78540100
H -0.01414000 -2.47181000 -1.49736300
H -2.69717200 -1.01156900 -2.66138600
C 1.30577000 -1.22138600 -2.76272500
H 1.23322100 -1.83177600 -3.67470500
H 2.26033100 -1.49035700 -2.28703300
H 1.37607800 -0.17113600 -3.07186400
H -2.11483300 -2.61117500 -0.74832100
C -3.23290100 3.01570100 0.13986400
C -2.57012100 4.20543800 0.84855200
C -4.54527700 2.63069400 0.83844200
H -3.47045100 3.33326300 -0.88589900
H -1.64073600 4.50176400 0.34666900
H -3.23661300 5.07626000 0.86516500
H -2.32381900 3.95279000 1.88839300
H -5.09805400 1.85868800 0.28956800
H -4.35012000 2.24788900 1.84904300
H -5.20576900 3.50093000 0.93226500
C -4.75322700 -1.10241400 -2.42122800
C -3.63213600 -1.40306000 -5.11346700
C -4.89044400 -2.17723500 -4.70338200
C -4.89703100 -2.41269900 -3.18848900

C -3.49183000 -0.10194100 -4.32875000
H -3.64987800 -1.15167500 -6.17923900
H -4.69556000 -1.26671300 -1.34554800
H -5.78311800 -1.60437200 -4.98807000
H -4.93446100 -3.13379900 -5.23497300
H -5.82811200 -2.88976600 -2.86470900
H -4.07521200 -3.08499400 -2.90677300
H -2.55298400 0.40890600 -4.55235200
H -2.73873000 -2.01874200 -4.94080800
H -5.57565200 -0.41358600 -2.63371600
H -4.32553200 0.58184100 -4.51128600
C -1.21446500 -2.25051500 1.19554500
C -1.61927900 -3.65385200 1.66417000
C -1.27443300 -1.23916800 2.34487300
H -0.18039000 -2.29307000 0.83087500
H -1.55172200 -4.38694400 0.85035400
H -0.97147400 -3.99751900 2.47900900
H -2.65301400 -3.66034700 2.03627200
H -0.94121500 -0.24827600 2.01943600
H -2.29867700 -1.14242600 2.73124100
H -0.63208800 -1.55315300 3.17639600

Im-int-II

C -4.59370200 -0.31720600 -1.55208200
H -4.71494300 -1.31153600 -1.10486300
H -4.64861700 -0.45240900 -2.63985900
C -3.18365700 0.21543800 -1.24186100
H -3.07161800 1.17690100 -1.75025900
C -3.00912600 0.43983300 0.27581300
H -3.19972600 -0.48608200 0.82299700
H -3.68075200 1.21056200 0.65804900
N -2.13078300 -0.65550800 -1.78777500
N -1.60416200 0.86920000 0.62215000
C -2.08291300 -2.05295300 -1.34333600
H -2.70902600 -2.16011800 -0.45148200
H -1.06321900 -2.30362700 -1.02414800
C -0.97087800 -0.02305000 -2.24591300
C 0.27845200 -0.51408700 -2.37445700
H -1.14911300 1.01108600 -2.53933500
H 0.50118700 -1.54720000 -2.12211500
H -0.96439300 0.34037800 0.00976300
C 1.41516100 0.29994100 -2.93017200
H 2.26502700 0.33723000 -2.23372100
H 1.11105100 1.33284800 -3.13980800
H 1.80262700 -0.12617000 -3.86747400
C -5.77031700 0.57170500 -1.09681000
C -7.09129400 -0.06630500 -1.55067100
C -5.65708800 2.01097600 -1.61850900
H -5.78102000 0.60542900 0.00233000
H -7.19313500 -1.09061700 -1.17109400
H -7.95194700 0.51193500 -1.19371100
H -7.14694100 -0.10834200 -2.64646100
H -4.78508600 2.53471000 -1.20847000
H -5.57207500 2.02361500 -2.71325100
H -6.54529600 2.59340300 -1.34590500
C -1.35619600 2.33777400 0.33999700
C 0.23021400 0.86174200 2.31572000
C 0.53184300 2.33560200 2.01757800
C 0.10880400 2.68530400 0.58590500
C -1.23290800 0.52162100 2.04685800
H 0.44485100 0.61680400 3.36155700
H -1.65336300 2.52224500 -0.69323800
H -0.01528800 2.97236500 2.72571600
H 1.59919200 2.53734400 2.15641000
H 0.23892700 3.75359000 0.38284300
H 0.73112100 2.14022800 -0.13699000
H -1.43160100 -0.54458200 2.17145000
H 0.86576000 0.21560700 1.69465800
H -2.02531700 2.88842100 1.00694700
H -1.91272300 1.09032000 2.68787100
C -2.56149100 -3.07445000 -2.39491900
C -2.55528100 -4.47923000 -1.77803900
C -1.72929700 -3.03219100 -3.68125800
H -3.59736500 -2.81409600 -2.65124800
H -3.18444300 -4.52970200 -0.88009900
H -2.93040900 -5.22216800 -2.49162700
H -1.53789400 -4.77793200 -1.49136700
H -1.73217400 -2.03260500 -4.12780900
H -0.68624800 -3.31206200 -3.48603200
H -2.13041700 -3.73474900 -4.42214400

TS-rotation-Im

C -2.84813800 1.59931000 -1.98275600

H	-3.75570400	1.43525700	-2.57897500
H	-2.07341800	1.93031100	-2.67744300
C	-2.44746500	0.25652100	-1.33419500
H	-1.65736900	0.45484100	-0.59445100
C	-3.68826500	-0.31563800	-0.58778500
H	-4.08983400	-1.16481900	-1.14126600
H	-4.48222300	0.42526500	-0.48234200
N	-1.94612400	-0.73212700	-2.31663700
N	-3.41172200	-0.79654600	0.82143700
C	-1.49126300	-0.32077200	-3.64433800
H	-2.24882800	0.33217400	-4.08695500
H	-1.47355100	-1.21403700	-4.27702000
C	-1.61582300	-1.99653000	-1.83943800
C	-1.05891200	-3.04954700	-2.47068600
H	-1.87372500	-2.14832500	-0.79454400
H	-0.74560600	-2.97815600	-3.50876100
H	-2.52357600	-1.31284100	0.82029000
C	-0.81994900	-4.36992600	-1.78995600
H	-1.34336300	-5.19400800	-2.29759500
H	-1.16204100	-4.35775500	-0.74703200
H	0.24633600	-4.64121300	-1.78347000
C	-3.07841400	2.76037800	-0.99164900
C	-3.77270900	3.92176400	-1.71785200
C	-1.76295300	3.23924900	-0.36021400
H	-3.74787800	2.42934900	-0.18671900
H	-4.74736900	3.61756900	-2.11888900
H	-3.93564900	4.76827900	-1.03987800
H	-3.16109600	4.27883200	-2.55693900
H	-1.22862600	2.43533100	0.15917600
H	-1.08921700	3.63755700	-1.13048800
H	-1.94703000	4.03866000	0.36733400
C	-3.24620100	0.34593900	1.80167000
C	-4.15977100	-2.29747000	2.68231900
C	-3.96651600	-1.16160500	3.69447200
C	-2.90068300	-0.18141700	3.19060300
C	-4.48062700	-1.76370200	1.28926200
H	-4.97894300	-2.96045600	2.98087700
H	-2.47070800	1.00244500	1.40592600
H	-4.91645400	-0.62815100	3.83355000
H	-3.67657100	-1.56977700	4.66828700
H	-2.80646100	0.68082200	3.85928300
H	-1.91890300	-0.67282300	3.15790800
H	-4.52624900	-2.56331500	0.54665400
H	-3.25044300	-2.91159600	2.62900200
H	-4.19901700	0.88181300	1.79832200
H	-5.41983800	-1.20395200	1.27071600
C	-0.11578300	0.38309200	-3.72239500
C	0.11628400	0.86759800	-5.16007000
C	1.04682200	-0.49613400	-3.24713900
H	-0.15081300	1.26563300	-3.06818000
H	-0.67889500	1.54810800	-5.49014700
H	1.07064900	1.40041900	-5.24698000
H	0.14343800	0.02075800	-5.85915200
H	0.90190200	-0.83899700	-2.21803800
H	1.15612000	-1.38366800	-3.88254800
H	1.99014000	0.06320600	-3.28786700

Id-int-I

C	-2.13389200	1.83017300	0.05841600
H	-2.89641100	2.61702000	-0.03237900
H	-1.26667800	2.16814100	-0.52434800
C	-2.68961400	0.54489300	-0.58495700
H	-3.56264600	0.22430900	-0.01401900
C	-3.14250600	0.87725800	-2.02315800
H	-2.34608400	1.39777900	-2.55861700
H	-4.03268000	1.50972900	-2.02541000
N	-1.73194300	-0.57288500	-0.53521700
N	-3.46138700	-0.35244300	-2.83694000
C	-2.18284900	-1.84976900	-0.00507000
H	-2.91217500	-1.67792900	0.78970700
H	-1.33150300	-2.39024600	0.42322500
C	-0.67813100	-0.57518900	-1.44631700
C	-0.03332200	-1.64836600	-1.94941300
H	-0.36788700	0.42042800	-1.75867200
H	-0.32635300	-2.65360800	-1.65461700
H	-2.68935000	-1.01717900	-2.68095100
C	1.13627400	-1.53481900	-2.88817100
H	0.96073700	-2.08994700	-3.82082000
H	2.05517000	-1.95058700	-2.44950100
H	1.34232800	-0.49081300	-3.15430400
H	-2.64381200	-2.50095000	-0.76865000

C -1.72403100 1.68380400 1.53471400
C -2.91270800 1.32941300 2.43922700
C -1.04655000 2.97345600 2.01853600
H -0.99217000 0.86674800 1.59507300
H -3.36281500 0.36610200 2.17298400
H -2.59673200 1.26343700 3.48733400
H -3.69605300 2.09701400 2.37554500
H -0.17620300 3.22667900 1.40015200
H -1.74351300 3.82145600 1.97809400
H -0.70377600 2.87404000 3.05566600
C -4.74990600 -1.02928900 -2.41821300
C -3.66391000 -1.33943400 -5.12401200
C -4.95073400 -2.06544700 -4.71420200
C -4.95447500 -2.32124600 -3.20249600
C -3.46444100 -0.05594000 -4.32322100
H -3.67924400 -1.07249000 -6.18606200
H -4.69288900 -1.21174600 -1.34513900
H -5.82014400 -1.45019100 -4.98234100
H -5.04070400 -3.01151700 -5.25855300
H -5.90229500 -2.76274500 -2.87684100
H -4.15990500 -3.03214000 -2.93747500
H -2.50773600 0.41914100 -4.54981800
H -2.79581000 -1.99432500 -4.96704400
H -5.54454800 -0.30396500 -2.61427500
H -4.27190500 0.66284500 -4.48905600

Id-int-II

C -4.58128100 -0.29795100 -1.60796700
H -5.26767800 0.50040800 -1.29387700
H -4.80308100 -1.15942400 -0.96456700
C -3.14869400 0.19870700 -1.33889200
H -3.02954000 1.16610000 -1.83490300
C -2.97755200 0.39186000 0.18174300
H -3.12498700 -0.55902100 0.69842400
H -3.69241400 1.11659500 0.57900500
N -2.11168000 -0.67738400 -1.90734000
N -1.59726400 0.87535200 0.56156100
C -2.03327900 -2.03940500 -1.39903400
H -3.03687000 -2.44107800 -1.25051900
H -1.48153600 -2.11025600 -0.44583000
C -0.90629000 -0.05585600 -2.24739000
C 0.33871200 -0.57065900 -2.17316300
H -1.04266500 0.95668400 -2.62698500
H 0.49804900 -1.57606500 -1.79009700
H -0.91608700 0.36444300 -0.02285300
C 1.55721500 0.16692400 -2.65607500
H 2.32459900 0.23310300 -1.87210600
H 1.31307600 1.18726100 -2.97562500
H 2.03020400 -0.34198900 -3.50874600
H -1.51992600 -2.66998200 -2.13171000
C -4.87468500 -0.67183900 -3.07176900
C -4.64560700 0.50487000 -4.02988000
C -6.30967500 -1.20303200 -3.19221400
H -4.18529400 -1.47830700 -3.35571300
H -3.59842400 0.82785300 -4.03757100
H -4.91057100 0.22622100 -5.05704200
H -5.26485300 1.36743000 -3.74792200
H -6.47458200 -2.06429200 -2.53258700
H -7.03961000 -0.42897400 -2.91946400
H -6.52806100 -1.51934100 -4.21927300
C -1.39161200 2.34802500 0.27286600
C 0.16335100 0.95745400 2.33444100
C 0.41665200 2.44019800 2.03648900
C 0.04548900 2.75790200 0.58310800
C -1.27009500 0.55453000 2.00428400
H 0.34113400 0.72849500 3.39070500
H -1.64329500 2.50920200 -0.77609700
H -0.18895500 3.05930400 2.71201800
H 1.46723200 2.68814900 2.22138900
H 0.13974800 3.82892000 0.37435900
H 0.72414800 2.23430000 -0.10391800
H -1.42885600 -0.51812400 2.13027400
H 0.85218900 0.33436100 1.74762100
H -2.11375800 2.87928100 0.89947900
H -1.99974500 1.09853600 2.61072700

TS-rotation-Id

C -4.25182200 -0.69475300 -1.47848600
H -4.86558900 -0.18486800 -0.72674800
H -4.35221800 -1.76221800 -1.25314600
C -2.76316100 -0.28456500 -1.29181100
H -2.56480800 0.57242500 -1.95088100

C	-2.65169700	0.17916800	0.17948300
H	-2.69314400	-0.68579700	0.84591800
H	-3.50165900	0.82867900	0.39028100
N	-1.83890300	-1.40418600	-1.63549800
N	-1.46201900	1.00787300	0.63647700
C	-2.19536600	-2.22170100	-2.79479200
H	-3.22765800	-2.56036900	-2.73502500
H	-1.56017000	-3.11257300	-2.79656800
C	-0.46719500	-1.10750000	-1.57764600
C	0.51537100	-1.46871200	-2.42305500
H	-0.16181900	-0.56313700	-0.69236700
H	0.29787200	-2.01783200	-3.33504400
H	-0.62677300	0.41543100	0.65528300
C	1.96491000	-1.16393300	-2.16160700
H	2.56067900	-2.08138100	-2.04951200
H	2.09919200	-0.57016000	-1.24856200
H	2.41260600	-0.60380800	-2.99463800
H	-2.04890500	-1.69689400	-3.75303900
C	-4.86213100	-0.35588100	-2.85683500
C	-5.11497800	1.15259000	-2.99326100
C	-6.16243700	-1.14377800	-3.05960100
H	-4.15899700	-0.65063100	-3.64527400
H	-4.19915900	1.73820200	-2.84569700
H	-5.50482700	1.39564900	-3.98896200
H	-5.85199100	1.49213500	-2.25309400
H	-5.98123700	-2.22560800	-3.03604400
H	-6.88857700	-0.90851200	-2.26981600
H	-6.62679800	-0.90066300	-4.02282400
C	-1.15967200	2.18941800	-0.25941300
C	-0.45958300	2.20900200	2.59463000
C	-0.13538200	3.41627600	1.70753100
C	0.05075300	2.96357500	0.25566000
C	-1.67650600	1.45087700	2.07389700
H	-0.67582800	2.51674500	3.62333000
H	-0.97929700	1.80275100	-1.26228400
H	-0.95633700	4.14371500	1.76378100
H	0.76934500	3.91800200	2.06621000
H	0.20383400	3.81814000	-0.41194700
H	0.94174700	2.32586700	0.17218400
H	-1.87817000	0.54671200	2.65254000
H	0.40111600	1.52786900	2.63546800
H	-2.06792300	2.79886200	-0.26820800
H	-2.57139100	2.07898400	2.06638200
