# Supplementary material for: Enantiodivergence by minimal modification of an acyclic chiral secondary aminocatalyst
Source: Nat Commun. 2019 Nov 15;10:5182. doi: 10.1038/s41467-019-13183-5 (PMC6858435; doi:10.1038/s41467-019-13183-5)
Supplement: Supplementary file 4 — Supplementary Data 2 [file 41467_2019_13183_MOESM4_ESM.pdf]

Supplementary Data 2

TS-I (Ia-S,R)

C	-4.22332500	-1.01606900	-1.90577800
H	-3.63896300	-1.58901000	-2.63325600
H	-4.71208300	-0.20105300	-2.44784100
C	-3.26931500	-0.38087500	-0.86261600
H	-3.88335900	0.10321000	-0.10364000
C	-2.39960600	-1.46817700	-0.22074900
H	-1.76857700	-1.93804400	-0.97425500
H	-3.05159500	-2.23437900	0.20020600
N	-2.44435700	0.71293200	-1.45678000
N	-1.47543300	-1.00000900	0.86640200
C	-0.63461300	-2.16826000	1.33980900
C	0.52492000	-1.73726900	2.22845700
H	-0.25251100	-2.63904700	0.43501200
H	-1.29923400	-2.87222400	1.84502300
H	1.17289900	-2.60517900	2.39056300
H	1.11437500	-0.95246900	1.74432100
H	0.19678800	-1.37992900	3.20890700
C	-2.17464600	-0.27984400	1.99947200
C	-3.30243500	-1.06971200	2.64915900
H	-2.53990200	0.66220800	1.59485200
H	-1.39688400	-0.01814400	2.71337900
H	-3.67797300	-0.48448300	3.49523100
H	-4.13988400	-1.23844200	1.96465300
H	-2.96614900	-2.03609100	3.03709800
C	-2.79711300	2.09099700	-1.04768200
C	-4.09284900	2.59088300	-1.71596400
H	-1.97181000	2.75529200	-1.28915800
H	-2.90446800	2.09452200	0.03807200
H	-4.90826400	1.90581400	-1.44878600
C	-4.43015900	3.97550700	-1.14590600
H	-3.64753500	4.70237100	-1.39713900
H	-5.37453000	4.34185800	-1.56469700
H	-4.53201500	3.94770900	-0.05453700
C	-3.96486400	2.63908300	-3.24224400
H	-3.74277900	1.65497100	-3.66951000
H	-4.89922500	2.99278700	-3.69327200
H	-3.16298400	3.32609700	-3.54219600
C	-5.26121700	-1.90074100	-1.24916700
C	-6.29676800	-1.32846100	-0.49367400
C	-5.19107500	-3.29675900	-1.34776000
C	-7.23849300	-2.13371400	0.14980500
H	-6.36344700	-0.24552000	-0.41315000
C	-6.13464400	-4.10573800	-0.70771300
H	-4.39190500	-3.75153000	-1.92843900
C	-7.15971600	-3.52639700	0.04429700
H	-8.03534600	-1.67502800	0.72947100
H	-6.06716700	-5.18691600	-0.79662700
H	-7.89350400	-4.15394400	0.54311200
C	-1.57863400	0.44164600	-2.41165400
C	-0.52422000	1.28379900	-2.86893800
H	-1.57514600	-0.58535800	-2.76453800
H	-0.60712300	2.33978300	-2.63396900
H	-0.78435100	-0.30517000	0.42936200
C	0.00968500	1.00955500	-4.26084800
H	0.05518600	-0.06277700	-4.48022000
H	1.01189700	1.43127500	-4.38001100
N	0.28043300	0.90632400	-0.21489100
O	0.14164700	3.19638200	-0.26573400
C	0.08574100	2.05746300	0.48499900
C	0.15236700	4.53357200	0.34501300
H	-2.01151900	4.70403100	0.33851500
H	-1.33295900	4.13633400	1.88024900
C	0.33250300	5.45175600	-0.86499000
C	0.83126600	0.87939100	-1.47867500
C	1.86701500	1.81655900	-1.85840400
C	2.75350300	2.55914600	-2.22827900
C	3.80233700	3.41432300	-2.67844300

C	4.15063400	4.57423100	-1.95623700
C	4.50248300	3.10458600	-3.86339600
C	5.17248100	5.40386500	-2.41466600
H	3.61770600	4.81339600	-1.04161400
H	4.23799400	2.20909000	-4.41851900
C	5.52196100	3.94125300	-4.31322600
C	5.85940700	5.09205300	-3.59238000
H	5.43313500	6.29565000	-1.85120500
O	-0.17882700	2.05795500	1.68974800
C	-1.18052900	4.81747300	1.04194900
C	1.34097300	4.65355700	1.30163000
H	0.35568600	6.49820400	-0.54258300
H	-1.18603300	5.84895700	1.41292400
H	1.20918000	4.00741200	2.17174800
H	1.43794000	5.69012300	1.64314900
H	2.26769400	4.36925300	0.79119500
H	1.26742700	5.22718800	-1.38836500
H	-0.49699600	5.32636100	-1.57036900
H	6.05518500	3.69559000	-5.22755900
H	-0.64234100	1.47746900	-5.00888200
H	6.65479300	5.74200200	-3.94668400
C	1.14069300	-0.56116900	-1.89968400
O	0.50069100	-1.52219300	-1.51080600
O	2.13920400	-0.63605200	-2.77488000
C	2.42163900	-1.94724700	-3.34245000
H	1.48526400	-2.37335500	-3.71435800
H	2.80271500	-2.59251700	-2.54488900
C	3.43505000	-1.73794100	-4.44717800
H	3.03128900	-1.08027400	-5.22458700
H	4.35588000	-1.29326900	-4.05471800
H	3.68269200	-2.70295600	-4.90273300

TS-V (Ia-R,R)

C	-3.75677900	-1.39509700	-1.88703800
H	-3.00854800	-2.10081000	-2.26382100
H	-4.14482000	-0.83934100	-2.74533000
C	-3.06926500	-0.37666300	-0.94090800
H	-3.84818700	0.23580800	-0.48669500
C	-2.30130000	-1.13683500	0.15081400
H	-1.54672400	-1.79148900	-0.28841000
H	-3.01543000	-1.76410900	0.68521700
N	-2.21264900	0.58085900	-1.70488400
N	-1.57328000	-0.30152500	1.16902200
C	-0.94743300	-1.22398500	2.19954500
C	-0.02557500	-0.48615600	3.15897700
H	-0.36762800	-1.94165400	1.61776400
H	-1.76070300	-1.73681900	2.71633200
H	0.51655700	-1.23133900	3.75100700
H	0.71204800	0.10839600	2.60868000
H	-0.56220900	0.16547000	3.85478900
C	-2.39413000	0.80738100	1.77922100
C	-3.63317500	0.32837700	2.52168600
H	-2.65625900	1.48452400	0.97070400
H	-1.72100700	1.35700900	2.43623400
H	-4.15519600	1.20903300	2.91027500
H	-4.32775800	-0.20821400	1.86726000
H	-3.38578700	-0.31432200	3.37182900
C	-2.72203000	1.96721300	-1.83118900
C	-3.88990500	2.08538000	-2.82876100
H	-1.90789400	2.61785800	-2.14331100
H	-3.03966800	2.29542700	-0.84086500
H	-4.70044600	1.42375300	-2.49527700
C	-4.40593700	3.53042700	-2.78558700
H	-3.62279100	4.23454500	-3.09535100
H	-5.25652500	3.65357800	-3.46536000
H	-4.73421300	3.81008600	-1.77724200
C	-3.47239200	1.68507100	-4.24760200
H	-3.11023900	0.65189200	-4.29452200
H	-4.32198600	1.76900200	-4.93475700
H	-2.67312600	2.34038400	-4.61713900
C	-4.88308300	-2.13197200	-1.19575900
C	-6.08242800	-1.46279500	-0.90648000
C	-4.74474800	-3.46987400	-0.80366500
C	-7.11986200	-2.11639400	-0.23910400
H	-6.20016300	-0.42462100	-1.21011600
C	-5.78302300	-4.12803100	-0.13768700
H	-3.81928400	-3.99754700	-1.02222000
C	-6.97236600	-3.45263200	0.14767200
H	-8.04352000	-1.58514400	-0.02438400
H	-5.66141100	-5.16711200	0.15707200
H	-7.78001200	-3.96314300	0.66546300

C	-1.14770700	0.14541300	-2.33385800
C	-0.08198200	0.89947200	-2.91551400
H	-0.99504900	-0.92957800	-2.29329700
H	-0.24536900	1.96717900	-3.03023900
H	-0.75327700	0.11680800	0.66110200
C	0.57557200	0.28134000	-4.13089700
H	0.66072400	-0.80131400	-4.03401100
H	1.57806500	0.69310600	-4.28129700
N	0.84023600	-0.23529100	-0.43552500
O	1.70880700	-1.71833800	-1.95924700
C	1.19844400	-1.50845500	-0.70478400
C	2.28286800	-3.00938800	-2.35584200
H	4.16715500	-2.57570600	-1.37774100
H	3.06810100	-3.58185900	-0.40627500
C	2.82705100	-2.73590600	-3.76058000
C	1.12482600	0.81362000	-1.29488900
C	2.43489800	0.94442700	-1.88081200
C	3.54812700	1.05905900	-2.35406200
C	4.85465200	1.18591900	-2.90896700
C	5.29234100	2.42458200	-3.42255500
C	5.72192300	0.07410800	-2.95612000
C	6.56654900	2.54217100	-3.97402600
H	4.62592500	3.28134000	-3.38432100
H	5.38894500	-0.87908200	-2.55686800
C	6.99418500	0.20305900	-3.50980100
C	7.41978100	1.43426000	-4.01980700
H	6.89571300	3.49978800	-4.36784200
O	1.03283000	-2.44100800	0.09692200
C	3.43206200	-3.38806500	-1.41637200
C	1.18990500	-4.08096100	-2.40428800
H	3.34165100	-3.62620400	-4.13823000
H	3.93339000	-4.28697700	-1.79253400
H	0.80048000	-4.28590300	-1.40603900
H	1.59592900	-5.00693200	-2.82789700
H	0.36368400	-3.74736300	-3.04343400
H	2.01957500	-2.48683900	-4.45630600
H	3.53869400	-1.90406000	-3.74337800
H	7.65575000	-0.65823300	-3.54249100
H	-0.01938700	0.51108200	-5.02465600
H	8.41276000	1.53025700	-4.45031300
C	0.59379700	2.12142400	-0.70314300
O	-0.28812300	2.15906900	0.13844200
O	1.15360800	3.20221900	-1.24365700
C	0.63524700	4.49275800	-0.81005400
H	0.78385000	4.57995700	0.27040600
H	-0.43991100	4.51920900	-1.01460600
C	1.39123800	5.55329900	-1.58110800
H	2.46585300	5.49275200	-1.37742900
H	1.23054600	5.44140400	-2.65882900
H	1.03692500	6.54487200	-1.27876400

TS-VI (Ia-S,S)

C	-4.07681100	-0.37258500	-1.09412100
H	-4.30318200	-1.28641200	-0.53590200
H	-4.14493400	-0.60402700	-2.16167000
C	-2.63189800	0.09183100	-0.80768200
H	-2.50008000	1.04213900	-1.32343600
C	-2.38592400	0.27650700	0.70155900
H	-2.06147700	-0.66160100	1.14969200
H	-3.31092000	0.57945200	1.19058000
N	-1.60059900	-0.81555300	-1.38985700
N	-1.34871000	1.31109800	1.04940200
C	-1.01865800	1.20372800	2.52356200
C	0.01189000	2.22827500	2.97857200
H	-0.63518500	0.19184900	2.65372100
H	-1.95624800	1.29681100	3.07688800
H	0.35721600	1.94560100	3.97880000
H	0.87098900	2.24983700	2.30183700
H	-0.40685900	3.23721700	3.04141500
C	-1.73950000	2.71263700	0.62700500
C	-2.95935700	3.27244400	1.34370300
H	-1.90518800	2.67791200	-0.45073600
H	-0.85189200	3.32349900	0.78074200
H	-3.15845700	4.26999300	0.93784600
H	-3.85686600	2.66812400	1.18457900
H	-2.79075900	3.37843500	2.41972600
C	-1.71094600	-2.27368100	-1.15099900
C	-2.42633500	-3.05694500	-2.27212900
H	-2.23735300	-2.41053700	-0.20568200
H	-0.70997300	-2.68013600	-1.02119600
H	-3.45439700	-2.68982800	-2.35644400

C	-1.74454200	-2.90565100	-3.63580300
H	-0.71357500	-3.27856400	-3.60565700
H	-2.28927300	-3.48141200	-4.39312200
H	-1.72063100	-1.86236000	-3.97047000
C	-2.47676600	-4.53007800	-1.84047300
H	-2.99611600	-4.65070600	-0.88207400
H	-3.00445900	-5.13069800	-2.58970900
H	-1.46429000	-4.94097700	-1.73217100
C	-5.06576700	0.71991800	-0.74048300
C	-5.13477200	1.88440300	-1.52080000
C	-5.88637100	0.61819800	0.39056100
C	-5.99495800	2.92695500	-1.17245100
H	-4.50600400	1.97385800	-2.40423600
C	-6.75126800	1.65953600	0.74131500
H	-5.84325500	-0.27941400	1.00288500
C	-6.80483000	2.81838500	-0.03713300
H	-6.03409800	3.82293300	-1.78636800
H	-7.37986200	1.56429300	1.62277700
H	-7.47334500	3.63031500	0.23620500
C	-0.59509500	-0.27260700	-2.03768800
C	0.67389900	-0.83656900	-2.36677700
H	-0.69779100	0.79391800	-2.22306800
H	0.78332600	-1.91097900	-2.25735400
H	-0.42903700	1.09460400	0.54551300
C	1.31694400	-0.30316200	-3.63010200
H	2.39223000	-0.50323100	-3.63063800
H	1.16875000	0.77322100	-3.73357200
N	1.16153200	1.03720100	-0.22992200
O	2.13917900	2.22943800	-1.92864500
C	1.52168000	2.25183900	-0.71100700
C	2.73179000	3.43175200	-2.52374500
H	0.88521200	3.95728500	-3.53212600
H	1.13444000	4.80418000	-1.98820100
C	3.40584900	2.89188800	-3.78736300
C	1.64626900	-0.15482800	-0.73625600
C	3.02151800	-0.26796200	-1.15746500
C	4.18507200	-0.39967100	-1.48101900
C	5.55109700	-0.56012100	-1.85577100
C	6.35765300	0.56289200	-2.13506000
C	6.10939800	-1.85195000	-1.95347700
C	7.68981700	0.39160700	-2.50680100
H	5.93283100	1.55865700	-2.05534400
H	5.48984200	-2.71700100	-1.73530000
C	7.44256000	-2.01154100	-2.32609500
C	8.23536600	-0.89280400	-2.60433200
H	8.30369800	1.26221600	-2.72068300
O	1.27120900	3.30434400	-0.11005400
C	1.63205900	4.43355200	-2.88582400
C	3.77899600	4.02946400	-1.57971500
H	3.90380900	3.70723100	-4.32329700
H	2.06490300	5.28051700	-3.43060900
H	3.31330700	4.44003800	-0.68236300
H	4.32714500	4.82764800	-2.09295100
H	4.49637100	3.25658900	-1.28098400
H	4.15430200	2.13540900	-3.53044400
H	2.67020300	2.43646400	-4.45842700
H	7.86439400	-3.01012100	-2.39940200
H	0.88062500	-0.80641700	-4.50308700
H	9.27449800	-1.02155000	-2.89453400
C	1.22144200	-1.31484200	0.17509100
O	0.32839700	-1.22742400	0.99752000
O	1.90220900	-2.43614100	-0.06701100
C	1.55717300	-3.60398800	0.73218300
C	2.38058700	-4.75795100	0.20212800
H	0.48277600	-3.78960700	0.64138700
H	1.78050500	-3.37956600	1.77969700
H	2.16598100	-5.65774400	0.78894900
H	2.13645300	-4.96141100	-0.84625500
H	3.45170400	-4.54168400	0.27772900

TS-II (Ia-R,S)

C	-4.14984300	-0.43125200	-0.78207600
H	-4.37963400	-1.16899500	-0.00701200
H	-4.24814200	-0.92228200	-1.75481300
C	-2.68755100	0.05197900	-0.65031700
H	-2.55798600	0.84990500	-1.38026000
C	-2.40449000	0.58301100	0.76659800
H	-2.13562600	-0.23452500	1.43762300
H	-3.29729400	1.06283900	1.16491900
N	-1.68919400	-0.99188400	-1.01973400
N	-1.28160200	1.58329300	0.85596800

C	-1.00421400	1.87490600	2.31887400
C	0.16645800	2.82598600	2.51948900
H	-0.76910500	0.90289100	2.75372800
H	-1.92709600	2.25865700	2.75891200
H	0.41816800	2.83603700	3.58544000
H	1.04798100	2.48197800	1.96735400
H	-0.06291100	3.85308200	2.22141000
C	-1.49640800	2.83068400	0.02815400
C	-2.70104300	3.66095600	0.44695900
H	-1.57758200	2.51397100	-1.00980600
H	-0.57241600	3.40142100	0.10035900
H	-2.76749300	4.51979500	-0.22950300
H	-3.64091500	3.10678200	0.36746900
H	-2.60376100	4.04734500	1.46608500
C	-1.81739800	-2.34229900	-0.41960100
C	-2.59568500	-3.35551000	-1.28884300
H	-2.31599500	-2.22521300	0.54386000
H	-0.82154100	-2.72693600	-0.21237300
H	-3.61340700	-2.98090200	-1.43825100
C	-1.96091700	-3.58436100	-2.66468700
H	-0.94255800	-3.98119000	-2.56983600
H	-2.55362700	-4.31027200	-3.23373100
H	-1.91493600	-2.66318300	-3.25624500
C	-2.68621400	-4.67340600	-0.50522900
H	-3.15162700	-4.52879900	0.47712700
H	-3.28678500	-5.40442300	-1.05859400
H	-1.69004600	-5.10686400	-0.35049000
C	-5.10976800	0.73821200	-0.70038700
C	-5.20853100	1.64104300	-1.77014700
C	-5.87428100	0.97163400	0.45044100
C	-6.04826300	2.75323600	-1.69039900
H	-4.62195300	1.46847300	-2.67003100
C	-6.71595500	2.08503800	0.53428200
H	-5.80740300	0.27859100	1.28584400
C	-6.80295700	2.98040700	-0.53465900
H	-6.11354700	3.44238500	-2.52836400
H	-7.30146900	2.25166400	1.43468900
H	-7.45585900	3.84674500	-0.47019100
C	-0.73838000	-0.68113200	-1.87929400
C	0.49982700	-1.35884500	-2.06511700
H	-0.85503300	0.28288500	-2.36763000
H	0.57516000	-2.35297800	-1.63854200
H	-0.40985500	1.08031500	0.52219100
C	1.16374100	-1.22811800	-3.42015300
H	2.23457000	-1.43923200	-3.34667600
H	1.03401700	-0.22863700	-3.84969300
N	0.84480800	-0.26492700	0.50934300
O	1.07306900	-2.49574500	0.99090300
C	0.68459700	-1.25766100	1.41939000
C	1.22878000	-3.63320400	1.90924700
H	3.19294300	-2.93407000	2.49804100
H	1.89912300	-2.48856600	3.63352200
C	1.74622100	-4.73718100	0.98574300
C	1.57423300	-0.42578300	-0.64849400
C	2.77180500	-1.23537400	-0.68638600
C	3.80842700	-1.86170200	-0.77496000
C	5.03902800	-2.57188500	-0.89529300
C	5.83436700	-2.40376000	-2.04846900
C	5.48080700	-3.43882400	0.12555400
C	7.03919700	-3.09198800	-2.17511200
H	5.49725800	-1.73265300	-2.83328100
H	4.87480700	-3.56758500	1.01686000
C	6.68867300	-4.12119500	-0.01084600
C	7.46983400	-3.95164500	-1.15864300
H	7.64417100	-2.95636400	-3.06744300
O	0.17417800	-1.07372500	2.53127200
C	2.26827300	-3.27807700	2.97459000
C	-0.11357900	-4.03867100	2.52373700
H	1.90714700	-5.65845900	1.55558500
H	2.49854400	-4.16173400	3.58006200
H	-0.50734400	-3.24782000	3.16281800
H	0.02388900	-4.94780400	3.12073300
H	-0.84523400	-4.25631200	1.73918400
H	1.01935300	-4.94404200	0.19186800
H	2.69231500	-4.44414500	0.52016800
H	7.02163200	-4.78635200	0.78123000
H	0.72648400	-1.95244600	-4.11887900
H	8.41033300	-4.48613100	-1.26044700
C	1.73569000	0.92010200	-1.36202000
O	0.92226900	1.82248900	-1.27056700

O	2.82950900	0.97536900	-2.11675100
C	3.01903600	2.18074300	-2.91125100
C	4.28340100	1.98003600	-3.71933200
H	3.09197700	3.03465000	-2.23104500
H	2.13810500	2.31934000	-3.54536600
H	4.47273300	2.87056200	-4.32851400
H	5.14499800	1.82006500	-3.06238800
H	4.18638800	1.11738700	-4.38735200
