# Supplementary material for: Enantiodivergence by minimal modification of an acyclic chiral secondary aminocatalyst
Source: Nat Commun. 2019 Nov 15;10:5182. doi: 10.1038/s41467-019-13183-5 (PMC6858435; doi:10.1038/s41467-019-13183-5)
Supplement: Supplementary file 5 — Supplementary Data 3 [file 41467_2019_13183_MOESM5_ESM.pdf]

Supplementary Data 3

TS-III (Id-S,R)

C	-2.10512500	1.31865100	-0.55927000
H	-2.87637900	2.09938900	-0.53579400
H	-1.29225400	1.70559800	-1.18652000
C	-2.71550600	0.08492700	-1.25380500
H	-3.53786200	-0.29245700	-0.64726300
C	-3.23999000	0.49329000	-2.63479400
H	-2.42680800	0.88319000	-3.24597500
H	-3.98095500	1.28462800	-2.49712200
N	-1.73955800	-1.04270000	-1.34078400
N	-3.90062900	-0.58551600	-3.45192000
C	-2.06648100	-2.20396600	-0.50513500
H	-2.17519300	-1.86247500	0.52848000
H	-1.28134600	-2.95115600	-0.55791200
C	-0.62382400	-0.90424800	-2.02632500
C	0.26767900	-1.93354500	-2.44286700
H	-0.46227100	0.08356400	-2.44801800
H	0.20534300	-2.87834000	-1.91233600
H	-3.20071300	-1.37302900	-3.61838800
C	1.68064800	-1.50820400	-2.78842300
H	2.28191600	-1.40944100	-1.87618900
H	1.70301700	-0.54483900	-3.30990200
N	-2.03347700	-2.78683900	-3.74562700
O	-1.42364700	-4.62944300	-2.51715900
C	-2.43501300	-3.89045800	-3.05868300
C	-1.64442600	-5.96307900	-1.94095900
H	-2.00349400	-5.19179400	0.05883200
H	-3.48315600	-5.49561000	-0.88251000
C	-0.22216300	-6.42700000	-1.62182800
C	-0.70202500	-2.58577500	-4.05165600
C	0.15038700	-3.68452800	-4.44989400
C	0.90244900	-4.55594300	-4.83572100
C	1.78705000	-5.57627100	-5.29248500
C	1.39641000	-6.93101100	-5.27111600
C	3.06943900	-5.23646300	-5.77137400
C	2.27314300	-7.91923500	-5.71473800
H	0.40722300	-7.19424900	-4.90949600
H	3.36916000	-4.19274000	-5.79309500
C	3.93895900	-6.23249500	-6.21058300
C	3.54487200	-7.57455900	-6.18351100
H	1.96330400	-8.96041900	-5.69520500
O	-3.62369600	-4.16814400	-2.87162200
C	-2.48070200	-5.86486300	-0.66231600
C	-2.28967500	-6.87982500	-2.98307800
H	-0.24491600	-7.42535900	-1.17201100
H	-2.56034600	-6.85552900	-0.20015200
H	-3.30874700	-6.56230900	-3.21237100
H	-2.31798400	-7.90697500	-2.60269200
H	-1.70272500	-6.87165400	-3.90831900
H	0.38575500	-6.46725900	-2.53113800
H	0.25788500	-5.74043200	-0.91541200
H	4.92583600	-5.96177500	-6.57571500
H	2.16180600	-2.25640700	-3.42488900
H	-3.00648800	-2.64683300	-0.83970800
C	-1.58690000	1.07526200	0.87064500
C	-2.73071700	0.79787100	1.85557800
C	-0.75793300	2.28209400	1.33107900
H	-0.92184400	0.20008000	0.85285200
H	-3.33231100	-0.07115600	1.56503000
H	-2.33894900	0.60513700	2.86121200
H	-3.40367000	1.66322300	1.91848900
H	0.09787300	2.45511500	0.66695600
H	-1.36677800	3.19592500	1.34020300
H	-0.37206300	2.12877900	2.34583000
C	-5.12431200	-1.15882000	-2.77002100
C	-4.94604700	-1.02908500	-5.69227600
C	-6.22802900	-1.54572900	-5.03303100

C	-5.88712700	-2.13751400	-3.66337700
C	-4.26624000	0.00201000	-4.80093700
H	-5.15460900	-0.55060600	-6.65590000
H	-4.78359000	-1.66420100	-1.86581800
H	-6.94249400	-0.71716700	-4.92557600
H	-6.70466000	-2.30640600	-5.66136100
H	-6.79361500	-2.42740400	-3.11873200
H	-5.27402600	-3.03431900	-3.78225300
H	-3.34279100	0.37283900	-5.24082900
H	-4.25092300	-1.85752600	-5.88079500
H	-5.74354700	-0.30021400	-2.48992700
H	-4.93608000	0.84428300	-4.59742100
H	4.22570800	-8.34816800	-6.52778600
C	-0.51009700	-1.34470900	-4.92614400
O	-1.24306900	-0.37231700	-4.87589600
O	0.57318400	-1.41726200	-5.69253400
C	0.91909700	-0.22747400	-6.45737900
H	0.99242300	0.61747100	-5.76571400
H	0.11069400	-0.02365700	-7.16588000
C	2.23276200	-0.51722400	-7.15066000
H	2.53484800	0.35658300	-7.73839100
H	3.01944300	-0.73274900	-6.41962000
H	2.13784800	-1.37359300	-7.82652300

TS-VII (Id-R,R)

C	-1.53143500	1.25489800	-0.81044800
H	-1.97065200	2.24364100	-0.99585800
H	-0.53209800	1.26966100	-1.26445500
C	-2.39464100	0.21916400	-1.55900500
H	-3.39619400	0.21333400	-1.12966000
C	-2.47788000	0.61775900	-3.04094900
H	-1.48469200	0.66788000	-3.49143700
H	-2.92675600	1.61253900	-3.08967300
N	-1.87591200	-1.17165100	-1.35230800
N	-3.28986800	-0.27445500	-3.94110400
C	-2.71991300	-2.05981800	-0.53647000
H	-3.03380400	-1.50920300	0.35237800
H	-2.15280800	-2.93611200	-0.22814800
C	-0.70399900	-1.51973800	-1.81906600
C	-0.14278900	-2.82997400	-1.94740200
H	-0.13675800	-0.71923300	-2.28469500
H	-0.67146200	-3.63664500	-1.44483100
H	-2.77029600	-1.18214500	-4.01336600
C	1.36024300	-2.91919400	-1.77815500
H	1.74866700	-3.82663900	-2.24956700
H	1.61036600	-2.95936900	-0.70991400
N	-0.94428000	-1.91437500	-4.53508100
O	1.30867000	-1.47190500	-4.59369000
C	0.04204400	-1.10420300	-4.96822000
C	2.50223200	-0.83005900	-5.15479300
H	2.35961300	-1.98037400	-6.98519000
H	1.67392400	-0.34480700	-7.11036000
C	3.64794600	-1.66865800	-4.58268200
C	-0.68615000	-3.09751800	-3.86112700
C	0.30539100	-4.02404000	-4.34558700
C	1.13444200	-4.81118800	-4.75689400
C	2.11775500	-5.72026900	-5.24311000
C	2.21730200	-7.01811400	-4.70040800
C	3.00928800	-5.32838000	-6.26363300
C	3.19181500	-7.89796200	-5.16656200
H	1.52940900	-7.32075600	-3.91645500
H	2.92954100	-4.33115400	-6.68587300
C	3.97871600	-6.21715800	-6.72379900
C	4.07400100	-7.50118200	-6.17720300
H	3.26365900	-8.89540300	-4.74178100
O	-0.16952900	-0.06076900	-5.60776100
C	2.48667300	-0.93420200	-6.68284800
C	2.60125500	0.61878300	-4.66986000
H	4.60584000	-1.31199000	-4.97651100
H	3.43820300	-0.57273500	-7.08884400
H	1.78240900	1.21847100	-5.06978400
H	3.55450200	1.05405200	-4.99141800
H	2.56433900	0.65423800	-3.57433400
H	3.68040300	-1.59895100	-3.49058800
H	3.52798800	-2.72195100	-4.85598400
H	4.66134900	-5.90756700	-7.51028400
H	1.86519100	-2.06210900	-2.22521500
H	-3.59568400	-2.37159800	-1.10567000
C	-1.40135900	1.03564600	0.70881100
C	-2.73024000	1.25489200	1.44525900
C	-0.31834700	1.96901100	1.26822400

H -1.07326600 0.00040400 0.88073100
H -3.52426900 0.58803700 1.09033300
H -2.60992500 1.07689600 2.52035800
H -3.07904300 2.28768300 1.31424900
H 0.65265200 1.78764700 0.79112800
H -0.58330000 3.02120000 1.09978500
H -0.19598600 1.82434100 2.34810900
C -4.70466500 -0.49583000 -3.46687400
C -4.02884200 -0.60652900 -6.31034900
C -5.47836600 -0.82161200 -5.86429400
C -5.50130600 -1.37144400 -4.43440100
C -3.28055200 0.30484500 -5.34480700
H -3.98218500 -0.14709800 -7.30411300
H -4.66681000 -0.95922400 -2.48274300
H -6.01891700 0.13438200 -5.90433800
H -5.99135200 -1.51342900 -6.54170800
H -6.52601500 -1.43132800 -4.04990900
H -5.08518100 -2.38359100 -4.41637300
H -2.23004200 0.40437100 -5.62294400
H -3.50129900 -1.56710400 -6.37254400
H -5.14780200 0.50048600 -3.36933900
H -3.75415900 1.28931800 -5.27471700
H 4.83229600 -8.19055700 -6.53814100
C -2.00760500 -3.77193000 -3.48778800
O -3.06092600 -3.16076400 -3.40619000
O -1.87492400 -5.06362400 -3.20360500
C -3.07042400 -5.76610400 -2.75656200
H -3.82307000 -5.70609100 -3.54847300
H -3.46025700 -5.25539100 -1.87026000
C -2.65893900 -7.19241200 -2.46124100
H -3.53133100 -7.75914200 -2.11804400
H -2.26156900 -7.67788400 -3.35879300
H -1.89425700 -7.22314900 -1.67775800

TS-VIII (Id-S,S)

C -3.65951100 0.47114700 -1.65075100
H -4.22151300 1.30088000 -1.20316800
H -4.20815900 -0.44555100 -1.39985300
C -2.28248200 0.44456700 -0.96569100
H -1.76871000 1.39332400 -1.12865400
C -2.43977100 0.22139800 0.54334000
H -2.71341400 -0.81309200 0.75152300
H -3.25172800 0.85143200 0.90987300
N -1.39439400 -0.60925200 -1.54257800
N -1.21817400 0.54923900 1.37007600
C -1.71403800 -2.02149300 -1.26334800
H -1.26478900 -2.34037200 -0.32328600
H -1.34037300 -2.64119400 -2.07787000
C -0.35009700 -0.25633400 -2.24446500
C 0.83039000 -1.02804700 -2.49320200
H -0.30674100 0.78989900 -2.52450300
H 0.75048900 -2.08988600 -2.26980400
H -0.33127300 0.45432100 0.78579000
C 1.56697600 -0.75630800 -3.78755700
H 2.55244800 -1.22943300 -3.76809100
H 1.70390600 0.31378100 -3.95090300
N 1.16236500 0.76104600 -0.29215700
O 1.67219300 2.10454800 -2.08990100
C 1.16761100 2.00939100 -0.81379300
C 1.91950900 3.40547100 -2.72613900
H -0.04055000 3.44994200 -3.65650100
H 0.05343200 4.33394300 -2.11405800
C 2.64198700 3.02732000 -4.02122800
C 1.80493400 -0.29893100 -0.94236500
C 3.14755100 -0.10630900 -1.43657300
C 4.28771600 0.04346500 -1.82696400
C 5.62285800 0.21987500 -2.29255800
C 6.41243300 -0.90067900 -2.62428400
C 6.16651300 1.51326500 -2.43599400
C 7.71380300 -0.72605000 -3.09015400
H 5.99451000 -1.89684200 -2.51217500
H 5.56148600 2.37655700 -2.17695100
C 7.46962800 1.67657900 -2.90166600
C 8.24558600 0.56030300 -3.23051400
H 8.31512700 -1.59467600 -3.34403700
O 0.69783800 2.99418700 -0.22964000
C 0.58910600 4.09748600 -3.03479600
C 2.83269200 4.26635000 -1.84810100
H 2.92304200 3.93237700 -4.57052100
H 0.77100400 5.02503100 -3.59000500
H 2.32892700 4.56986900 -0.92960600

H	3.13390600	5.16285300	-2.40148200
H	3.73691500	3.70670000	-1.58365800
H	3.54963500	2.45498700	-3.80363400
H	1.99736400	2.42256900	-4.66785400
H	7.88073100	2.67666700	-3.00803600
H	1.00848100	-1.17620800	-4.63409200
H	-2.79801000	-2.13046500	-1.21260000
C	-3.62354900	0.65152700	-3.17997300
C	-3.02623900	2.00622600	-3.58942900
C	-5.04051800	0.49221700	-3.74823500
H	-2.99881300	-0.14653500	-3.60626000
H	-1.99194700	2.13297600	-3.25013900
H	-3.02715200	2.11635000	-4.68022500
H	-3.61558200	2.83203800	-3.16952200
H	-5.46400300	-0.48785300	-3.49656200
H	-5.71291800	1.26153400	-3.34641500
H	-5.03798400	0.58822300	-4.84040700
C	-1.26419800	1.98885700	1.84477900
C	0.09384400	-0.07871300	3.41064000
C	0.06773800	1.37059600	3.90858000
C	-0.05805000	2.32438600	2.71599100
C	-1.12612400	-0.39049100	2.55154800
H	0.10478900	-0.78765600	4.24641100
H	-1.28694500	2.61186400	0.95094900
H	-0.78174700	1.50935500	4.59226800
H	0.97886800	1.59284200	4.47559500
H	-0.17285800	3.36288700	3.04822000
H	0.83845500	2.28322100	2.09133400
H	-1.08259700	-1.40105700	2.14838000
H	0.99618700	-0.25965300	2.81523500
H	-2.20121100	2.09328900	2.40108700
H	-2.05680400	-0.26403200	3.11522000
H	9.26117100	0.69181300	-3.59355600
C	1.66568800	-1.57882400	-0.11206500
O	0.78726000	-1.75095700	0.71320900
O	2.57123900	-2.49924800	-0.43883100
C	2.46420300	-3.78736500	0.23071400
H	2.54301700	-3.62444300	1.30972300
H	1.47537800	-4.20649000	0.01740200
C	3.58276900	-4.65619600	-0.30234000
H	4.55901600	-4.20695800	-0.09125100
H	3.54169600	-5.63922200	0.17935300
H	3.48781600	-4.79599100	-1.38454900

TS-IV (Id-R,S)

C	-3.96697400	0.06359500	-1.78926800
H	-4.59073700	0.93723000	-1.56045000
H	-4.43976000	-0.79040200	-1.28823000
C	-2.58535900	0.32848700	-1.15846000
H	-2.19050500	1.26743900	-1.54769300
C	-2.74018200	0.43164300	0.36500800
H	-3.00113900	-0.54067400	0.78538400
H	-3.56006200	1.11844900	0.58840100
N	-1.58591400	-0.71497800	-1.52204500
N	-1.53479500	0.92873300	1.11755700
C	-1.88665900	-2.10050600	-1.13449100
H	-1.85558900	-2.22065900	-0.05086500
H	-1.16673700	-2.77623000	-1.58629700
C	-0.54093900	-0.37439400	-2.25364900
C	0.67180900	-1.09830100	-2.40620400
H	-0.56227800	0.64321200	-2.62795200
H	0.65849700	-2.14927000	-2.13488100
H	-0.66086500	0.42011300	0.76977000
C	1.52410000	-0.77207300	-3.61485900
H	2.56359600	-1.06726700	-3.44315000
H	1.50161000	0.29644400	-3.85392100
N	0.71666900	-0.66042000	0.34927800
O	0.93609600	-2.94162400	0.20305800
C	0.46996400	-1.87597600	0.90968400
C	0.93134900	-4.30901600	0.74387600
H	2.70708800	-3.91593500	1.92568600
H	1.19033200	-3.79537400	2.84403400
C	1.66405800	-5.09689400	-0.34353800
C	1.57600000	-0.51486500	-0.71964700
C	2.79553900	-1.28591300	-0.80582700
C	3.84408300	-1.88952400	-0.90728200
C	5.07521300	-2.59987900	-1.01652000
C	5.39195100	-3.63219600	-0.10927900
C	5.99119500	-2.27702900	-2.03923900
C	6.59647500	-4.32291200	-0.22804600
H	4.69103100	-3.87973500	0.68172200

H	5.74977000	-1.48067300	-2.73704300
C	7.19198200	-2.97482500	-2.14988500
C	7.49818500	-3.99821200	-1.24664700
H	6.83242000	-5.11643400	0.47565400
O	-0.19610000	-2.00311600	1.94310100
C	1.71029100	-4.35046900	2.06091000
C	-0.50317000	-4.81904800	0.90552000
H	1.74153900	-6.15098300	-0.05669200
H	1.82945900	-5.39001100	2.38577400
H	-1.03754300	-4.25005900	1.66773000
H	-0.48335800	-5.87474900	1.19949900
H	-1.04607400	-4.74129100	-0.04285300
H	1.12264600	-5.03694700	-1.29441600
H	2.67280300	-4.70030700	-0.49535100
H	7.89073700	-2.71981900	-2.94185900
H	1.16492000	-1.32004500	-4.49482100
H	-2.88476400	-2.35783100	-1.49609100
C	-3.95614600	-0.16695500	-3.31159200
C	-3.47284900	1.07033500	-4.08133600
C	-5.35718600	-0.58298100	-3.78015800
H	-3.26840400	-0.99674400	-3.52818500
H	-2.44906800	1.35464000	-3.81403900
H	-3.48912200	0.88452500	-5.16172700
H	-4.12322300	1.93175000	-3.87970700
H	-5.69662100	-1.49129100	-3.26738500
H	-6.08923300	0.21026600	-3.57907200
H	-5.36659600	-0.78099900	-4.85853200
C	-1.33280100	2.41647000	0.94746100
C	-0.48560500	1.05192400	3.39153300
C	-0.25548800	2.55834500	3.23513900
C	-0.12861300	2.90765100	1.74869400
C	-1.69245600	0.58671600	2.58634200
H	-0.66037500	0.78250000	4.43945200
H	-1.20108500	2.61064600	-0.11510500
H	-1.09930100	3.10780400	3.67566800
H	0.64801900	2.86473900	3.77420300
H	-0.05272600	3.99078000	1.59889600
H	0.77955900	2.45880600	1.33484100
H	-1.79985000	-0.49494400	2.64075000
H	0.39320700	0.49113900	3.05242300
H	-2.25635800	2.89200000	1.29303800
H	-2.61352400	1.07675600	2.91892300
H	8.43593200	-4.53963200	-1.33611600
C	1.76479900	0.96635800	-1.06673100
O	0.86252900	1.78100900	-0.98985700
O	2.99771500	1.24832200	-1.48085600
C	3.27403400	2.63175700	-1.85367700
H	2.80301600	3.29107800	-1.12024400
H	4.35890800	2.71200200	-1.76625000
C	2.79963700	2.92748200	-3.26501400
H	1.71121400	2.84528300	-3.33538100
H	3.08625100	3.95024600	-3.53604100
H	3.25795700	2.23889600	-3.9821240
