# Supplementary material for: Enantiodivergence by minimal modification of an acyclic chiral secondary aminocatalyst
Source: Nat Commun. 2019 Nov 15;10:5182. doi: 10.1038/s41467-019-13183-5 (PMC6858435; doi:10.1038/s41467-019-13183-5)
Supplement: Supplementary file 6 — Supplementary Data 4 [file 41467_2019_13183_MOESM6_ESM.pdf]

Supplementary Data 4

Cat

C -4.35686100 -1.03945500 -1.96783700
H -3.64125600 -1.54927100 -2.62515100
H -4.83687900 -0.25339400 -2.55735100
C -3.56854400 -0.36322600 -0.82075500
H -4.28074100 0.16137300 -0.17501000
C -2.83605500 -1.44155100 0.00990800
H -2.26117300 -2.09057100 -0.65571600
H -3.51535300 -2.05871600 0.59531600
N -2.56508200 0.61663100 -1.25603600
N -1.84962100 -0.77053200 0.92603700
C -0.62980600 -1.62141300 1.17646600
C 0.43363700 -0.90413800 1.99387000
H -0.24397700 -1.87361200 0.18587300
H -0.96837600 -2.54186100 1.65649900
H 1.33657800 -1.52349800 2.00424800
H 0.69130700 0.06216900 1.54536200
H 0.12733400 -0.74200300 3.03117700
C -2.48699400 -0.19614600 2.17373300
C -2.88457200 -1.24004800 3.20610900
H -3.35524500 0.37186000 1.83545500
H -1.76821600 0.51422500 2.58541400
H -3.37597200 -0.72245300 4.03667600
H -3.59302600 -1.97121000 2.80477600
H -2.01847900 -1.77252000 3.60970900
C -2.95863500 2.03157600 -1.27929800
C -4.03058900 2.46244300 -2.30103300
H -2.04496400 2.61310500 -1.46350000
H -3.29854700 2.28884900 -0.26819500
H -4.94689000 1.89114200 -2.09617200
C -5.39950400 -2.01675500 -1.46964800
C -6.60258100 -1.54486100 -0.92158500
C -5.17749900 -3.40071800 -1.50493900
C -7.55724800 -2.43187900 -0.41995500
H -6.79073600 -0.47359600 -0.89309800
C -6.13058300 -4.29240200 -1.00402300
H -4.25131500 -3.78140000 -1.92953100
C -7.32356700 -3.81048400 -0.45891800
H -8.48476300 -2.04805400 -0.00257400
H -5.94095500 -5.36217700 -1.04193200
H -8.06664100 -4.50217200 -0.07080700
H -1.55958900 0.04362900 0.33677300
H -2.15150500 0.33359500 -2.14244400
C -4.34933600 3.95138600 -2.10879600
H -3.45943000 4.56876000 -2.29226600
H -5.13109000 4.28069500 -2.80383300
H -4.69661000 4.15976600 -1.08892800
C -3.58701600 2.18054600 -3.74139000
H -2.67670100 2.74561800 -3.98422300
H -3.37410000 1.11804800 -3.90671400
H -4.36442800 2.47596500 -4.45640400

1a

N 6.58311700 2.72711600 4.85618500
O 7.19648700 1.00330400 3.56523400
C 7.35114100 1.54523800 4.77087400
C 7.87475000 -0.26596200 3.18716800
H 9.67638600 0.79846800 2.62265400
H 9.72797900 0.11403700 4.26458200
C 7.39844300 -0.47439900 1.75130900
C 7.14026900 3.84712600 4.55651500
C 8.48124300 3.99256600 4.11033800
C 9.64165400 4.03185600 3.74424600
C 10.99380000 4.09405900 3.31369600
C 11.51781800 5.29708000 2.79456000
C 11.81728300 2.95122600 3.40034900
C 12.84218100 5.34831100 2.36793900

H	10.88036200	6.17363100	2.73113000
H	11.41079900	2.02987300	3.80444700
C	13.14063700	3.01712200	2.97243900
C	13.65407700	4.21173100	2.45538600
H	13.24322600	6.27472700	1.96708200
O	7.97394500	1.10643200	5.71782600
C	9.38795200	-0.05779600	3.24099400
C	7.39803300	-1.39947000	4.09476700
H	7.84467200	-1.38695400	1.34280700
H	9.89006700	-0.94980600	2.85091700
H	7.74017400	-1.26041600	5.12188500
H	7.79334000	-2.34955600	3.71897800
H	6.30398400	-1.45720200	4.08908300
H	6.30830700	-0.57427500	1.71585400
H	7.69245800	0.37020700	1.11899500
H	13.77273400	2.13649300	3.04116200
H	14.68694800	4.25752200	2.12164000
C	6.27201700	5.09194000	4.70565300
O	5.11599800	5.05765200	5.06784200
O	6.95404600	6.19123700	4.38410300
C	6.23021800	7.45309400	4.47555000
H	5.86280300	7.56464200	5.50028500
H	5.36817800	7.40227400	3.80320800
C	7.19473800	8.55367300	4.08966400
H	8.05348000	8.57865200	4.76899200
H	7.55933100	8.41536800	3.06615300
H	6.68073700	9.51944900	4.14666200

2a

C	-1.01435900	-1.05931800	-0.00445700
O	0.14830800	-0.94133500	0.33543200
H	-1.57792200	-0.18234100	-0.39192600
C	-1.80309100	-2.34037500	0.05197900
C	-1.01845600	-3.53371700	0.58912100
H	-2.18214600	-2.52499000	-0.96526800
H	-2.70067100	-2.13470600	0.65589500
H	-1.64225600	-4.43380600	0.60247000
H	-0.13929500	-3.73645800	-0.03227100
H	-0.66761900	-3.34532500	1.60963700

H2O

O	-4.62111500	0.73943500	0.00000000
H	-3.65105600	0.79408100	0.00000000
H	-4.89359500	1.67230700	0.00000000

Ia-int-III

C	-3.79594500	-1.29022100	-2.11407400
H	-3.16092200	-2.05224700	-2.57879100
H	-4.07799200	-0.57864200	-2.89591500
C	-2.97342400	-0.50226800	-1.05415400
H	-3.67751800	0.14884800	-0.53327500
C	-2.36642000	-1.49429700	-0.05361800
H	-1.59994800	-2.09831100	-0.53992500
H	-3.13828800	-2.16460700	0.32557100
N	-1.96609300	0.38998200	-1.64515700
N	-1.70058100	-0.86090700	1.13731400
C	-1.07339100	-1.93573500	2.00351700
C	-0.03899700	-1.38328000	2.97344300
H	-0.60091600	-2.63625400	1.31674900
H	-1.88719200	-2.44969400	2.51817900
H	0.43093400	-2.22747500	3.48928300
H	0.74136100	-0.83206300	2.43995200
H	-0.47712100	-0.73179600	3.73527200
C	-2.59662000	0.07675200	1.92356300
C	-3.91999500	-0.53410100	2.36058300
H	-2.74516400	0.95524200	1.29890800
H	-2.00891700	0.40235900	2.78205200
H	-4.43794300	0.20179800	2.98472800
H	-4.57203800	-0.76795300	1.51356300
H	-3.78438600	-1.44020300	2.95875700
C	-2.27614100	1.82315300	-1.64014600
C	-3.40151900	2.27162900	-2.59447500
H	-1.35942300	2.37686200	-1.85957100
H	-2.54748800	2.09418500	-0.61798600
H	-4.30850300	1.70930200	-2.32889600
C	-3.68264200	3.76194900	-2.35663600
H	-2.79781000	4.36856000	-2.59284000
H	-4.50603800	4.11261500	-2.99018600
H	-3.95310400	3.95922200	-1.31169100
C	-3.08532900	2.00183800	-4.06962000
H	-2.87366400	0.94378400	-4.25335500
H	-3.93147700	2.29198600	-4.70506600

H	-2.20882000	2.57764100	-4.39321600
C	-5.03406500	-1.92639200	-1.52234100
C	-6.12644000	-1.12494600	-1.15265200
C	-5.10670000	-3.30662500	-1.28846900
C	-7.26005400	-1.68815800	-0.56312400
H	-6.08495900	-0.05265200	-1.33265300
C	-6.24032600	-3.87470800	-0.69965000
H	-4.26737200	-3.93911900	-1.56861800
C	-7.31998900	-3.06685900	-0.33366100
H	-8.09736500	-1.05265200	-0.28604700
H	-6.27859300	-4.94735600	-0.52741300
H	-8.20202300	-3.50684300	0.12417500
C	-1.00273900	-0.13733700	-2.47513300
C	-0.07299700	0.52146800	-3.21063300
H	-0.98083400	-1.22377600	-2.48810500
H	-0.05782300	1.60642200	-3.23391400
H	-0.91660000	-0.27005000	0.77715900
C	0.90806300	-0.17572900	-4.10730800
H	0.84250300	-1.26620400	-4.01481500
H	1.93983200	0.11899500	-3.87585700
N	0.38342500	1.06258000	0.38312900
O	1.24417700	3.02276400	1.15259700
C	0.12486000	2.35169100	0.87981800
C	1.21786500	4.42111300	1.65277700
H	0.94058200	5.18924400	-0.35567100
H	-0.56591500	5.07437600	0.58278600
C	2.70431800	4.76392100	1.72858300
C	1.29795500	0.82070000	-0.50914100
C	2.11631000	1.75706300	-1.18221100
C	2.85141300	2.52009000	-1.78251100
C	3.72351800	3.39621800	-2.47836500
C	3.63452200	4.79302400	-2.29441300
C	4.69503300	2.86787800	-3.35644300
C	4.50559100	5.63901500	-2.97572400
H	2.88586100	5.19628200	-1.62001500
H	4.75801600	1.79276900	-3.49574400
C	5.56079600	3.72482200	-4.03007700
C	5.46876200	5.10878000	-3.84150300
H	4.43518800	6.71328500	-2.83132000
O	-1.01491500	2.72760000	1.09975800
C	0.49829200	5.31144000	0.63899400
C	0.57071700	4.45640700	3.03666400
H	2.82960900	5.79035300	2.08808300
H	0.60812100	6.35919300	0.93880700
H	-0.48919200	4.19971200	2.98578700
H	0.66842500	5.46441100	3.45456300
H	1.07474400	3.75495800	3.71069800
H	3.22120400	4.08807400	2.41819800
H	3.17198200	4.67921100	0.74203400
H	6.30823900	3.31525600	-4.70347500
H	0.73527800	0.07947800	-5.16361700
H	6.14699000	5.77350800	-4.36923900
C	1.52987200	-0.65442600	-0.79245600
O	0.84790400	-1.53851500	-0.30561200
O	2.58870000	-0.85004100	-1.57134100
C	2.90298700	-2.23130100	-1.91015700
H	2.01153400	-2.68955900	-2.34771700
H	3.14691300	-2.76332300	-0.98522700
C	4.06567100	-2.19781800	-2.87791600
H	3.79643900	-1.65813500	-3.79196500
H	4.93713300	-1.71209900	-2.42591900
H	4.34152700	-3.22296900	-3.14814300

Ia-int-IV

C	-4.52328600	-1.07729700	-2.11210100
H	-3.94990300	-1.60219700	-2.88282000
H	-5.08677000	-0.27941200	-2.60614000
C	-3.55536600	-0.40662600	-1.11245800
H	-4.15072800	0.07955100	-0.34048600
C	-2.60360100	-1.43304700	-0.48406200
H	-2.02056400	-1.92520700	-1.26389900
H	-3.19382300	-2.19637400	0.02293700
N	-2.76271000	0.69337800	-1.75853700
N	-1.61202100	-0.87329600	0.49743400
C	-0.61739500	-1.95861200	0.85781800
C	0.50276600	-1.45824900	1.75908600
H	-0.20890000	-2.30324600	-0.09337100
H	-1.17258500	-2.78261300	1.31161200
H	1.26214200	-2.24420600	1.83046900
H	0.97030200	-0.56273300	1.33824800
H	0.15635000	-1.23548100	2.77266500

C	-2.24305700	-0.21533200	1.70449900
C	-3.04562000	-1.15693300	2.58954500
H	-2.87234900	0.59124100	1.32997700
H	-1.42472800	0.26295400	2.23920800
H	-3.44717200	-0.57321300	3.42474100
H	-3.89373500	-1.60371700	2.06070800
H	-2.42955100	-1.95750300	3.00986000
C	-3.05950300	2.06965600	-1.29190300
C	-4.41139300	2.56204900	-1.84064600
H	-2.26134200	2.73492900	-1.60874600
H	-3.05977900	2.05254700	-0.20097500
H	-5.19440200	1.85912600	-1.52813500
C	-4.71753100	3.92825900	-1.21173700
H	-3.96837800	4.67165300	-1.51099200
H	-5.69807300	4.28837400	-1.54319000
H	-4.72739600	3.87517500	-0.11665400
C	-4.40045100	2.64622500	-3.37090400
H	-4.19817000	1.67523000	-3.83685000
H	-5.37168800	2.99412500	-3.74056500
H	-3.63434200	3.35323300	-3.71470900
C	-5.46770200	-2.03447300	-1.41650300
C	-6.48199700	-1.54106900	-0.58135400
C	-5.32934800	-3.42085800	-1.56487800
C	-7.33773100	-2.41502600	0.09164700
H	-6.60095300	-0.46624800	-0.46188800
C	-6.18745500	-4.29820200	-0.89539100
H	-4.54576700	-3.81370200	-2.20846200
C	-7.19247100	-3.79758200	-0.06404100
H	-8.11979500	-2.01796100	0.73355800
H	-6.06919800	-5.37097600	-1.02380400
H	-7.85983500	-4.47861400	0.45737300
C	-1.89480600	0.39508100	-2.67302300
C	-0.77395000	1.24542900	-3.13055100
H	-1.90575100	-0.62817500	-3.02695300
H	-1.00312600	2.30649800	-3.03578500
H	-1.01382900	-0.11024100	0.00670400
C	-0.37189000	0.92349900	-4.57324500
H	-0.13605800	-0.13852700	-4.69740300
H	0.50658900	1.51026700	-4.85576600
N	-0.01208300	1.07228100	-0.67556100
O	-0.08284100	3.35763100	-0.80460000
C	-0.16200100	2.23386000	-0.01361200
C	0.06521600	4.69845600	-0.23597000
H	-2.07416900	5.05388400	-0.17120900
H	-1.38957500	4.45889400	1.35679100
C	0.27354500	5.57279700	-1.47430200
C	0.43278200	1.01369300	-2.05506000
C	1.52199800	1.92558600	-2.43286100
C	2.44413500	2.63084300	-2.78267700
C	3.53857600	3.46906500	-3.15543700
C	3.32145600	4.63507100	-3.91770100
C	4.85104500	3.14150800	-2.75789800
C	4.39492200	5.45246300	-4.26907500
H	2.31223600	4.88834200	-4.22800300
H	5.02096400	2.24206800	-2.17309800
C	5.91727500	3.96570600	-3.11427700
C	5.69375000	5.12220200	-3.86889600
H	4.21684900	6.34962800	-4.85574300
O	-0.41668500	2.29594900	1.19790800
C	-1.21146100	5.11177200	0.50139900
C	1.29840700	4.74810100	0.67024100
H	0.35707200	6.62570200	-1.18367100
H	-1.12020200	6.14712200	0.85013300
H	1.15405700	4.12936200	1.55820400
H	1.48811300	5.78068500	0.98454200
H	2.17864500	4.38576600	0.12707800
H	1.18633600	5.28407500	-2.00517200
H	-0.57320100	5.47029300	-2.16289900
H	6.92507100	3.70486400	-2.80265100
H	-1.18682200	1.17706800	-5.25901400
H	6.52768100	5.76213400	-4.14402500
C	0.92716900	-0.45634300	-2.14416000
O	0.17486100	-1.40202700	-2.32040600
O	2.22539900	-0.57734300	-1.90472700
C	2.75251500	-1.93234800	-1.79463500
H	2.55534300	-2.45890900	-2.73304900
H	2.21684300	-2.44475600	-0.98937900
C	4.23278600	-1.80629900	-1.50546200
H	4.74595900	-1.27552800	-2.31460200
H	4.40202300	-1.26661900	-0.56758200

H	4.67138100	-2.80608300	-1.41402800

Ia-int-V

C	-4.57576100	-1.02299400	-2.16906700
H	-4.00035100	-1.53587200	-2.94700100
H	-5.10398000	-0.19180700	-2.64681100
C	-3.60326400	-0.43546300	-1.12183000
H	-4.19041200	0.04615400	-0.33995000
C	-2.69239600	-1.51703400	-0.51147200
H	-2.19252500	-2.05420400	-1.32364300
H	-3.35475300	-2.24691600	-0.02531800
N	-2.77357000	0.67085500	-1.71720400
N	-1.64752600	-1.02351600	0.40003500
C	-0.68343700	-2.11808800	0.66292700
C	0.50929200	-1.69521300	1.51748400
H	-0.31905400	-2.45103800	-0.31426100
H	-1.18370600	-2.98363600	1.12804000
H	1.23587500	-2.51486400	1.55038200
H	1.00991900	-0.81628500	1.09771300
H	0.22231200	-1.46686400	2.54905900
C	-2.18991700	-0.40797500	1.63546500
C	-2.91421800	-1.34569200	2.60824700
H	-2.86975800	0.39158200	1.33113800
H	-1.36102500	0.09575400	2.13795700
H	-3.29407200	-0.76247300	3.45535100
H	-3.77043600	-1.84472300	2.14026800
H	-2.24715600	-2.11653800	3.00939900
C	-3.07431600	2.04201600	-1.23652500
C	-4.39896700	2.57040700	-1.81833400
H	-2.26256600	2.70810000	-1.51761100
H	-3.11413500	1.99844200	-0.14707000
H	-5.20499400	1.88478300	-1.52851800
C	-4.68506000	3.94049400	-1.18838400
H	-3.91727800	4.66937700	-1.47600300
H	-5.65373200	4.32041800	-1.53192400
H	-4.71024400	3.88457800	-0.09368800
C	-4.34520500	2.66418700	-3.34707400
H	-4.15590100	1.69156200	-3.81477400
H	-5.29725800	3.03966900	-3.73868700
H	-3.55263200	3.35387100	-3.66490700
C	-5.57158800	-1.97286300	-1.53830100
C	-6.59400600	-1.47653900	-0.71497900
C	-5.47961200	-3.35653600	-1.73939200
C	-7.50147800	-2.34406700	-0.10438800
H	-6.67755500	-0.40374800	-0.55431700
C	-6.38921200	-4.22753600	-1.13265900
H	-4.69086700	-3.75258600	-2.37458300
C	-7.40145900	-3.72377900	-0.31185500
H	-8.28852600	-1.94394800	0.52959100
H	-6.30566900	-5.29797100	-1.30182600
H	-8.10898600	-4.39977100	0.16094400
C	-1.86842600	0.37868500	-2.58861000
C	-0.75935700	1.25817900	-3.05714700
H	-1.83760300	-0.65108000	-2.92390500
H	-1.01065900	2.31291300	-2.94430400
H	-0.46081700	0.40911900	-0.23207400
C	-0.41079800	0.98294900	-4.52506200
H	-0.14056600	-0.06353400	-4.68794100
H	0.42777100	1.61500600	-4.82980100
N	0.04617700	1.20952400	-0.65055900
O	0.04909000	3.46003800	-0.76138300
C	-0.14523700	2.39059000	0.02688300
C	0.18036300	4.82385500	-0.20203800
H	-1.95807800	5.16944100	-0.30673400
H	-1.38917300	4.65706500	1.29715100
C	0.50362900	5.65610500	-1.44142300
C	0.46343400	1.03289900	-2.04529000
C	1.56268100	1.91619900	-2.43559900
C	2.48154400	2.60279100	-2.82426300
C	3.56660000	3.43421300	-3.23563500
C	3.32602200	4.58894600	-4.00741500
C	4.88725500	3.11310100	-2.86259700
C	4.38880900	5.40442800	-4.39296400
H	2.30878300	4.83544900	-4.29591800
H	5.07229700	2.22234500	-2.26937100
C	5.94220600	3.93589700	-3.25327900
C	5.69686500	5.08175400	-4.01728900
H	4.19554300	6.29378800	-4.98638000
O	-0.48248700	2.41055900	1.20370900
C	-1.14529900	5.26096200	0.42163600

C 1.34010900 4.85217300 0.79360000
H 0.60584900 6.71017000 -1.16361000
H -1.07423500 6.31173200 0.72403700
H 1.11962400 4.25002700 1.67776800
H 1.52171500 5.88502600 1.10983600
H 2.25254100 4.47013700 0.32237100
H 1.44059900 5.32271000 -1.89802300
H -0.29716700 5.56885100 -2.18419100
H 6.95757800 3.68283500 -2.96105800
H -1.26879600 1.22504300 -5.16017500
H 6.52229300 5.72068900 -4.31894200
C 0.97783200 -0.44368400 -2.09812700
O 0.34212400 -1.36919700 -2.56632900
O 2.16458700 -0.55431400 -1.52042400
C 2.74414300 -1.89411600 -1.45002800
H 2.80841200 -2.29111800 -2.46718100
H 2.06986600 -2.52772800 -0.86756800
C 4.10230000 -1.75056000 -0.79997900
H 4.75076100 -1.09468200 -1.39084500
H 4.01075100 -1.33956700 0.21109200
H 4.57552500 -2.73611900 -0.73153600

Ia-int-VI

C -1.39769700 0.30881900 -1.75600300
C -0.29720800 1.16671200 -2.36064900
H -1.57212400 0.44757800 -0.67160700
H -0.60522300 2.20658600 -2.19034900
H 0.70797400 0.49071200 0.50704300
C -0.12965200 0.91275400 -3.85711000
H 0.11915500 -0.13158000 -4.06521400
H 0.65681000 1.54819300 -4.27312300
N 0.80776600 1.30265400 -0.09052500
O 0.38311300 3.47761700 -0.43282000
C 0.41524300 2.48837100 0.47717200
C 0.12175500 4.87974300 -0.05267900
H -2.02316100 4.60148600 -0.20038600
H -1.38901600 4.47161500 1.45684900
C 0.24963100 5.61061100 -1.38916900
C 1.03819000 1.02049700 -1.50966500
C 2.09708400 1.87807800 -2.05100300
C 2.94762500 2.61938800 -2.49129500
C 3.94051200 3.52032300 -2.98407300
C 4.62787500 3.24546000 -4.18276500
C 4.23644000 4.70141800 -2.27305900
C 5.58901200 4.13681400 -4.65739300
H 4.40143300 2.33556200 -4.73086600
H 3.70923000 4.91301400 -1.34733600
C 5.19922900 5.58578000 -2.75669500
C 5.87738600 5.30729200 -3.94788900
H 6.11480600 3.91725700 -5.58263800
O 0.15189700 2.58245300 1.66852900
C -1.29420800 5.00698300 0.51040800
C 1.19200300 5.35543200 0.93116100
H 0.07482700 6.68176300 -1.24438400
H -1.52691400 6.06468700 0.67638600
H 1.11701200 4.82697500 1.88345100
H 1.06721600 6.42857800 1.11259300
H 2.19140600 5.19179300 0.51215400
H 1.25119900 5.47385500 -1.81081500
H -0.48504900 5.23351500 -2.10901200
H 5.42066800 6.49376500 -2.20235800
H -1.06739000 1.14109500 -4.37228100
H 6.62724400 5.99910400 -4.32168400
C 1.43516900 -0.47883200 -1.56829200
O 1.01323400 -1.28838200 -0.76396000
O 2.19694200 -0.75985000 -2.61406900
C 2.50949300 -2.17076700 -2.83873500
H 1.56780400 -2.72486500 -2.88664000
H 3.08461500 -2.53217500 -1.98140800
C 3.29077700 -2.25002100 -4.13174900
H 2.69878600 -1.86858300 -4.97049700
H 4.21988200 -1.67404600 -4.06525600
H 3.54511200 -3.29602200 -4.33502000
O -2.08271900 -0.46159000 -2.40002700

TS-H-shift

C 3.93870300 -0.17095900 -1.67537500
H 3.62556500 0.64768400 -2.33174100
H 3.88303700 -1.09930700 -2.25272300
C 2.94803400 -0.29595000 -0.49682900
H 3.31432100 -1.07173600 0.17457600
C 2.79646100 1.03338000 0.25683100

H	2.44741600	1.80435500	-0.43288900
H	3.78559500	1.33927700	0.60543800
N	1.59990900	-0.77313900	-0.95241400
N	1.84554500	1.02071300	1.41141700
C	1.68439900	2.43581200	1.89988500
C	0.59243000	2.59955400	2.95160100
H	1.43493200	3.02792200	1.01744900
H	2.64895900	2.79466800	2.27227100
H	0.45379100	3.66932500	3.14228000
H	-0.35863500	2.18779900	2.60165700
H	0.85236400	2.12503400	3.90272600
C	2.25761700	0.07268800	2.50420200
C	3.62993500	0.32773700	3.12033700
H	2.21451300	-0.93326600	2.08653300
H	1.46870000	0.11178400	3.25321600
H	3.77910700	-0.39496000	3.93036700
H	4.44449900	0.18740200	2.40203400
H	3.71582400	1.32957800	3.55279100
C	1.20062200	-2.12459200	-0.49383500
C	1.98117900	-3.22043200	-1.24555200
H	0.13344100	-2.25496000	-0.65089600
H	1.38963600	-2.17367900	0.57990800
H	3.05530600	-3.05374200	-1.09163400
C	1.61442300	-4.57869000	-0.63236400
H	0.54949400	-4.79895400	-0.77705800
H	2.18988200	-5.37847500	-1.11196900
H	1.82554000	-4.60561900	0.44307900
C	1.68354500	-3.18926900	-2.74865700
H	1.96808400	-2.23436100	-3.20446800
H	2.23891100	-3.98093500	-3.26420400
H	0.61380300	-3.34968100	-2.93544300
C	5.35301000	0.05561900	-1.18546400
C	6.06124600	-0.99238200	-0.57740600
C	5.96481700	1.31163900	-1.29223500
C	7.35096800	-0.78805600	-0.08364500
H	5.59649600	-1.97243200	-0.49268500
C	7.25775000	1.51815900	-0.80248100
H	5.42411700	2.13075800	-1.76040700
C	7.95314000	0.46965300	-0.19485500
H	7.88687800	-1.60990800	0.38397300
H	7.71970900	2.49759900	-0.89535300
H	8.95804300	0.62921200	0.18698200
C	0.87321500	0.00837000	-1.68380200
C	-0.59778200	-0.05650100	-1.83860900
H	1.36474000	0.89096800	-2.07315800
H	-0.97374200	-1.07334000	-1.73110200
H	0.68482400	0.67039900	1.05053400
C	-1.06467600	0.53605400	-3.17084100
H	-0.70566900	1.56111400	-3.30265200
H	-2.15726500	0.54022600	-3.21393100
N	-0.57951200	0.27087800	0.71091100
O	-1.84241500	-1.62497400	0.65923500
C	-0.98418000	-0.84213400	1.37410600
C	-2.59277100	-2.72637900	1.27688200
H	-1.04253100	-4.17493300	0.83348300
H	-0.95694200	-3.50542600	2.47693300
C	-3.50332700	-3.19162400	0.13950300
C	-1.19698200	0.71255600	-0.53513600
C	-2.65820600	0.58755900	-0.59682800
C	-3.86461200	0.53810400	-0.70076800
C	-5.28778300	0.45400300	-0.77622400
C	-5.90374400	-0.49415500	-1.61828300
C	-6.09233400	1.31135500	0.00158500
C	-7.29402800	-0.57987400	-1.67607200
H	-5.28524800	-1.15466800	-2.21859000
H	-5.61901200	2.04250300	0.65046900
C	-7.48148000	1.21630100	-0.06287200
C	-8.08605400	0.27248100	-0.89982400
H	-7.76012800	-1.31393200	-2.32767500
O	-0.56534500	-1.14432800	2.49435700
C	-1.63565000	-3.84539300	1.69335200
C	-3.42182200	-2.19856900	2.44988300
H	-4.10728200	-4.04471800	0.46692600
H	-2.20923900	-4.70361100	2.06183400
H	-2.77896200	-1.85289000	3.26202500
H	-4.07165900	-2.99507000	2.82948200
H	-4.05412400	-1.36592500	2.12128800
H	-4.17652200	-2.38708400	-0.17251000
H	-2.90804600	-3.50176400	-0.72684300
H	-8.09376400	1.88006000	0.54149600

H	-0.69289300	-0.07089000	-4.00254400
H	-9.16927400	0.20161500	-0.94668900
C	-0.82434300	2.22304500	-0.57899200
O	0.18664300	2.65508000	-1.10682300
O	-1.68534200	2.96431500	0.10402900
C	-1.37834200	4.38412500	0.24314400
H	-1.27342200	4.81407500	-0.75708200
H	-0.42279900	4.48001000	0.76665200
C	-2.51971400	5.00296400	1.02049500
H	-3.46863600	4.87566400	0.48860400
H	-2.60804700	4.54719100	2.01249600
H	-2.33330300	6.07496300	1.14724500
