# Supplementary material for: Enantiodivergence by minimal modification of an acyclic chiral secondary aminocatalyst
Source: Nat Commun. 2019 Nov 15;10:5182. doi: 10.1038/s41467-019-13183-5 (PMC6858435; doi:10.1038/s41467-019-13183-5)
Supplement: Supplementary file 7 — Supplementary Information [file 41467_2019_13183_MOESM7_ESM.pdf]

Supplementary Information

Enantiodivergence by Minimal Modification of an Acyclic Chiral Secondary Aminocatalyst

Dai *et al.*

Supplementary Note 1

General Information

^{1}H-NMR and ^{13}C-NMR spectra were recorded at 400 MHz or 600 MHz spectrophotometer. Chemical shifts (δ) are expressed in ppm, and J values are given in Hz. NMR multiplicities are abbreviated as follows: s = singlet, d = doublet, t = triplet, q = quartet, m = multiple, br = broad signal, dd = doublet of doublet, dt = doublet of triplet. The enantiomeric excess was determined by chiral HPLC with *n*-hexane and *i*-propanol as eluents. High resolution mass spectrometry (HRMS) was recorded on a VG Auto Spec-3000 spectrometer. Optical rotations were measured on a JASCO DIP-370 polarimeter.

All chemicals and solvents were used as received without further purification unless otherwise stated. Column chromatography was performed on silica gel (200–300 mesh).

All the ketimines, including *β,γ*-Alkynyl-*α*-imino esters **1**,[1] trifluoromethylated alkynyl ketimines **4**,[1] isatin-derived ketimines **6** and isatins **10**[2], and pyrazolone-derived ketimines **8**[2] were synthesized by the reported methods.

Supplementary Note 2

Preparation of Catalysts

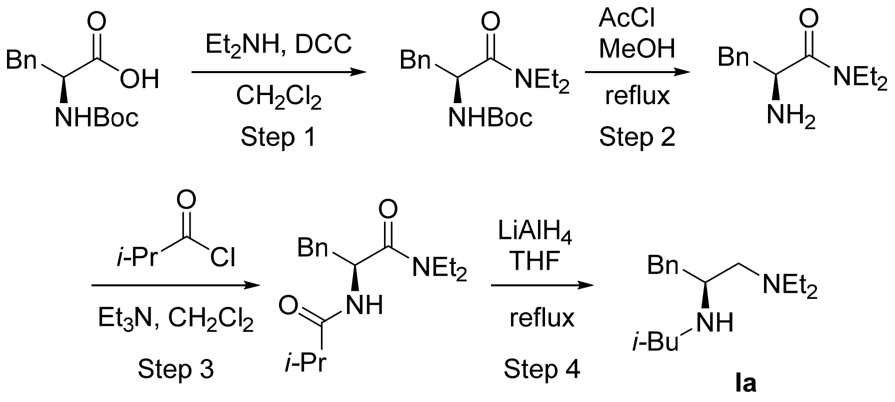

General Procedure A1 for the Synthesis of Ia

Step 1 : In a 100 mL of round-bottomed flask, *N*-Boc protected (*S*)-phenylalanine (2.65

g, 10 mmol) was dissolved in 20 mL of dry dichloromethane and the resulting solution was cooled to 0 °C. A solution of DCC (2.16 g, 10.5 mmol) in dry dichloromethane (20 mL) was slowly added to the above resulting solution in 0.5 h. During the addition, white solid was observed in the reaction system. The reaction mixture keeps stirring for 30 min. Then a solution of diethylamine (0.73 g, 10 mmol) in dry dichloromethane (20 mL) was added, and the resulting mixture was stirred at room temperature for 12 h. The reaction mixture was filtered, washed with dichloromethane, and the organic phases were washed once with saturated salt water. The organic phases were combined, dried with anhydrous Na_2SO_4, and filtered. The solvent was condensed under reduced pressure, and the residue was purified by silica gel column chromatography (petroleum ether:ethyl acetate = 8:1) to afford the corresponding amide as colorless oil (2.48 g, 81% yield).

Step 2: The amide obtained from the above step was dissolved in dry methanol (30 mL). To the resulting solution, acetyl chloride (3 mL) was added slowly. The reaction mixture was heated to 70 °C for 2 h. After the reaction system was cooled to room temperature, the solvent was concentrated under vacuum. 60 mL of dichloromethane and 40 mL of water were added, and the pH of the solution was adjusted to 8 ~ 10 by the using of solid K_2CO_3. The resulting solution was extracted with dichloromethane for three times. The combined organic phases were dried and filtered with anhydrous Na_2SO_4, and the solvent was condensed under reduced pressure. The residue was purified by silica gel column chromatography (dichloromethane:methanol = 20:1) to afford the product as colorless oil (1.6 g, 96% yield).

Step 3: In 100 mL of flask, the product obtained from the above step was dissolved in 30 mL of dry dichloromethane. The solution was cooled to 0 °C, triethylamine (1.62 mL, 11.67 mmol) was added at 0 °C. The reaction solution was stirred for 10 min and isopropionyl chloride (9.34 mmol, 0.98 mL) was added slowly. Then the reaction system was recovered to room temperature and stirred for 6 h. The reaction was quenched with saturated $NaHCO_3$ solution, and extracted with dichloromethane for three times. The combined organic phases were washed with saturated aqueous NaCl for once, dried by anhydrous Na_2SO_4, and filtered. The solvent was concentrated under

vacuum, the residue was purified by silica gel column chromatography (petroleum ether:ethyl acetate = 3:1) to afford the product as white solid (2.14 g, 95% yield).

Step 4: $LiAlH_4$ (1.4 g, 36.93 mmol) was slowly dissolved in 30 mL of dry THF under argon and the solution was cooled to 0 °C. To the resulting solution, a solution of the product obtained from the above step (2.14 g, 7.38 mmol) in dry THF (30 mL) was added slowly. The reaction system is heated to 80 °C. After the reaction was complete (monitored by TLC), the reaction was chilled to room temperature, and was quenched with 20 mL of ice water. The reaction mixture was filtered, extracted with ethyl acetate for three times, washed with saturated aqueous NaCl for once, dried with Na_2SO_4, and filtered. The organic solvent was concentrated under vacuum, and the residue was purified by silica gel column chromatography (dichloromethane:methanol = 10:1) to afford diamine **Ia** as colorless oil (1.68 g, 87% yield).

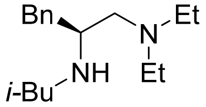

Ia

Compound Ia: $[\alpha]_D^{20}$ = +74.9 (*c* 2.0, $CHCl_3$); **^{1}H NMR** (400 MHz, $CDCl_3$, ppm): δ 7.29-7.25 (m, 2H), 7.19-7.16 (m, 3H), 2.83 (dd, 1H, J = 5.2, 13.2 Hz), 2.77-2.71 (m, 1H), 2.56-2.44 (m, 4H), 2.40-2.25 (m, 6H), 1.78-1.68 (m, 1H), 0.94 (t, 6H, J = 7.2 Hz), 0.88 (dd, 6H, J = 1.6, 6.8 Hz); **^{13}C NMR** (100 MHz, $CDCl_3$, ppm): δ 139.9, 129.3, 128.2, 125.9, 57.8, 57.5, 56.3, 47.4, 40.0, 28.4, 20.9, 20.7, 12.2; **HRMS** (ESI) m/z calcd. for $C_{17}H_{31}N_2$ $[M + H]^+$: 263.2482, found: 263.2482.

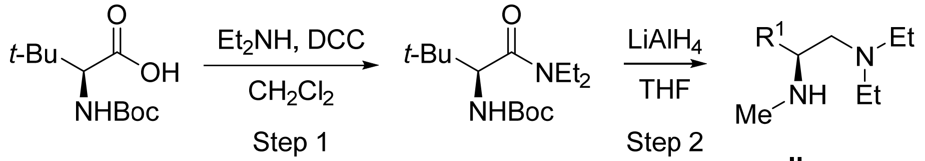

Ib

General Procedure A2 for the Synthesis of Ib

Step 1: In a 100 mL of round-bottomed flask, *N*-Boc-protected (*S*)-phenylalanine (1.33 g, 5 mmol) was dissolved in 20 mL of dry dichloromethane and the resulting solution was cooled to 0 °C. To the resulting solution, a solution of DCC (1.13 g, 5.3 mmol) in

10 mL of dry dichloromethane was slowly dropped for 0.5 h. During the dropwise addition, white solid was observed in the reaction system. The reaction mixture continued to be stirred for 30 min. Then a solution of diethylamine (0.37 g, 5 mmol) in dry dichloromethane (10 mL) was added, and the resulting mixture was stirred at room temperature for 12 h. The reaction mixture was filtered, washed with dichloromethane, and the organic phases were washed once with saturated salt water. The organic phases were combined, dried with anhydrous Na_2SO_4, and filtered. The solvent was condensed under reduced pressure, and the residue was purified by silica gel column chromatography (petroleum ether:ethyl acetate = 8:1) to afford the corresponding amide as colorless oil (1.27 g, 82% yield).

Step 2: $LiAlH_4$ (0.78 g, 20.5 mmol) was slowly dissolved in 15 mL of dry THF under argon at 0 °C. To the resulting solution, a solution of the amide obtained from the above step (1.27 g, 4.1 mmol) in dry THF (15 mL) was added slowly. The reaction mixture was heated to 80 °C. After the reaction was complete (monitored by TLC), the reaction system was chilled to room temperature. Then the reaction was quenched with 20 mL of ice water. The reaction mixture was filtered, extracted with ethyl acetate for three times. The combined organic phases were washed once with saturated salt water, dried by anhydrous Na_2SO_4, and filtered. The organic solvent was concentrated under vacuum, and the residue was purified by silica gel column chromatography (dichloromethane:methanol = 10:1) to afford diamine compound **Ib** as colorless oil (0.82 g, 91% yield).

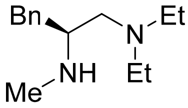

Ib

Compound Ib: $[\alpha]_D^{20}$ = +80.8 (*c* 1.0, $CHCl_3$); ^{1}**H NMR** (400 MHz, $CDCl_3$, ppm): δ 7.27-7.25 (m, 2H), 7.20-7.16 (m, 3H), 2.85 (dd, J = 5.2, 13.6 Hz, 1H), 2.72-2.66 (m, 1H), 2.56-2.45 (m, 4H), 2.43 (s, 3H), 2.40-2.34 (m, 2H), 2.32-2.25 (m, 2H), 0.94 (t, J = 7.2 Hz, 6H); ^{13}**C NMR** (100 MHz, $CDCl_3$, ppm): δ 142.9, 128.3, 127.5, 127.0, 59.7, 57.1, 47.5, 39.2, 34.6, 12.0; **HRMS** (ESI) m/z calcd. for $C_{14}H_{25}N_2$ $[M + H]^+$: 221.2012,

found: 221.2013.

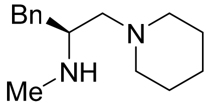

Ic

Following the General Procedure A2, diamine **Ic** was obtained as colorless oil (1.32 g, 67% yield). **$[\alpha]_D^{20}$** = +38.3 (*c* 1.0, $CHCl_3$); **^{1}H NMR** (600 MHz, $CDCl_3$, ppm): δ 7.29-7.26 (m, 2H), 7.20-7.18 (m, 3H), 2.86 (dd, J = 4.8, 13.2 Hz, 1H), 2.80-2.76 (m, 1H), 2.54 (dd, J = 7.2, 13.2 Hz, 1H), 2.45 (s, 3H), 2.37-2.33 (m, 2H), 2.25-2.12 (m, 4H), 1.57-1.47 (m, 4H), 1.42-1.34 (m, 2H); **^{13}C NMR** (150 MHz, $CDCl_3$, ppm): δ 139.5, 129.4, 128.2, 126.0, 62.7, 58.4, 54.9, 30.1, 34.6, 26.2, 24.5; **HRMS** (ESI) m/z calcd. for $C_{15}H_{25}N_2$ $[M + H]^+$: 233.2012, found: 233.2011.

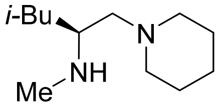

Id

Following the General Procedure A2, diamine **Id** was obtained as colorless oil (0.98 g, 63% yield). **$[\alpha]_D^{20}$** = +27.3 (*c* 1.0, $CHCl_3$); **^{1}H NMR** (600 MHz, $CDCl_3$, ppm): δ 3.88 (s, 1H), 2.65-2.60 (m, 1H), 2.847 (s, 1H), 2.45 (s, 3H), 2.29-2.25 (m, 4H), 1.69-1.64 (m, 1H), 1.60-1.52 (m, 5H), 1.44-1.39 (m, 3H), 1.19-1.14 (m, 1H) , 0.91 (t, J = 7.2 Hz, 6H); **^{13}C NMR** (150 MHz, $CDCl_3$, ppm): δ 60.0, 55.0, 54.5, 41.4, 33.5, 26.1, 25.0, 24.4, 23.5, 22.5; **HRMS** (ESI) m/z calcd. for $C_{12}H_{27}N_2$ $[M + H]^+$: 199.2169, found: 199.2171.

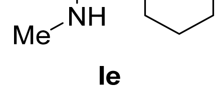

Ie

Following the General Procedure A2, diamine **Ie** was obtained as colorless oil (1.13 g, 68% yield). **$[\alpha]_D^{20}$** = +26.6 (*c* 1.0, $CHCl_3$); **^{1}H NMR** (600 MHz, $CDCl_3$, ppm): δ 2.45 (s, 2H), 2.39 (s, 3H), 2.38-2.36 (m, 1H), 2.29-2.15 (m, 5H), 1.89-1.84 (m, 1H), 1.58-1.51 (m, 4H), 1.44-1.39 (m, 2H) , 0.91 (d, J = 7.2 Hz, 3H), 0.86 (d, J = 7.2 Hz, 3H); **^{13}C NMR** (150 MHz, $CDCl_3$, ppm): δ 61.5, 59.2, 55.1, 35.2, 28.2, 26.2, 24.5, 19.0 16.9;

HRMS (ESI) m/z calcd. for $C_{11}H_{25}N_2$ $[M + H]^+$: 185.2012, found: 185.2009.

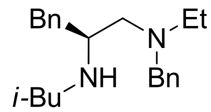

If

Following the General Procedure A1, diamine **If** was obtained as colorless oil (1.07 g, 67% yield). $[\alpha]_D^{20}$ = +81.1 (*c* 2.0, $CHCl_3$); **^{1}H NMR** (400 MHz, $CDCl_3$, ppm): δ 7.24-7.13 (m, 10H), 3.57 (d, *J* = 13.6 Hz, 1H), 3.37 (d, *J* = 13.6 Hz, 1H), 2.81-2.70 (m, 2H), 2.56-2.44 (m, 3H), 2.42-2.32 (m, 3H), 2.27-2.22 (m, 1H), 2.13 (s, 1H), 1.76-1.66 (m, 1H), 0.97 (t, *J* = 6.8 Hz, 3H), 0.89 (t, *J* = 6.4 Hz, 6H); **^{13}C NMR** (100 MHz, $CDCl_3$, ppm): δ 139.9, 139.8, 129.4, 128.8, 128.3, 128.2, 126.8, 126.0, 58.8, 58.0, 57.9, 56.3, 48.2, 39.9, 28.6, 21.0, 20.8, 12.1; **HRMS** (ESI) m/z calcd. for $C_{22}H_{33}N_2$ $[M + H]^+$: 325.2638 , found: 325.2638.

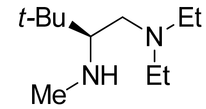

Ig

Following the General Procedure A2, diamine **Ig** was obtained as colorless oil (0.88 g, 71% yield). $[\alpha]_D^{20}$ = +122.4 (*c* 1.0, $CHCl_3$); **^{1}H NMR** (400 MHz, $CDCl_3$, ppm): *δ* 2.65-2.56 (m, 2H), 2.47 (s, 3H), 2.43-2.35 (m, 3H), 2.20-2.14 (m, 1H), 2.03 (dd, *J* = 3.2, 10.4 Hz, 1H), 1.87 (br, 1H), 0.99 (t, *J* = 6.8 Hz, 6H), 0.90 (s, 9H); **^{13}C NMR** (100 MHz, $CDCl_3$, ppm): *δ* 67.2, 54.1, 47.1, 39.7, 34.7, 27.2, 11.9; **HRMS** (ESI) m/z calcd. for $C_{11}H_{27}N_2$ $[M + H]^+$: 187.2169, found: 187.2168.

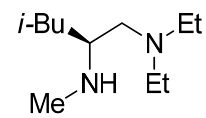

Ih

Following the General Procedure A2, diamine **Ih** was obtained as colorless oil (0.95 g, 74% yield). $[\alpha]_D^{20}$ = +32.1 (*c* 2.0, $CHCl_3$); **^{1}H NMR** (400 MHz, $CDCl_3$, ppm): *δ* 3.06 (brs, 1H), 2.62-2.53 (m, 2H), 2.52-2.43 (m, 3H), 2.41 (s, 3H), 2.38-2.34 (m, 1H), 2.29-2.24 (m, 1H), 1.72-1.62 (m, 1H), 1.42-1.35 (m, 1H), 1.16-1.09 (m, 1H), 0.99 (t, *J* = 7.2 Hz, 6H), 0.91 (dd, *J* = 2.4, 6.8 Hz, 6H); **^{13}C NMR** (100 MHz, $CDCl_3$, ppm): *δ* 57.7,

55.4, 47.4, 41.8, 33.9, 25.0, 23.6, 22.6, 11.9; **HRMS** (ESI) m/z calcd. for $C_{11}H_{27}N_2$ [M + H]$^+$: 187.2169, found: 187.2167.

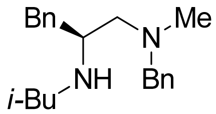

Ii

Following the General Procedure A1, diamine **Ii** was obtained as colorless oil (1.35 g, 68% yield). **[α]$_D$^{20}** = +54.7 (*c* 2.0, $CHCl_3$); **^{1}H NMR** (400 MHz, $CDCl_3$, ppm): δ 7.29-7.16 (m, 10H), 3.42 (dd, *J* = 35.6, 13.2 Hz, 2H), 2.86-2.80 (m, 2H), 2.60-2.49 (m, 2H), 2.43-2.38 (m, 1H), 2.35-2.31 (m, 1H), 2.27-2.23 (m, 1H), 2.09 (s, 3H), 1.78-1.68 (m, 1H), 0.90 (dd, *J* = 2.2, 6.8 Hz, 6H); **^{13}C NMR** (100 MHz, $CDCl_3$, ppm): δ 139.6, 139.3, 129.4, 128.9, 128.3, 128.2, 126.9, 126.0, 62.6, 61.6, 57.5, 56.3, 42.5, 39.8, 28.6, 21.0, 20.8; **HRMS** (ESI) m/z calcd. for [M + H]$^+$ $C_{16}H_{29}N_2$: 311.2482, found: 311.2481.

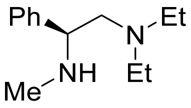

Ij

Following the General Procedure A2, diamine **Ij** was obtained as colorless oil (1.17 g, 68% yield). **[α]$_D$^{20}** = +26.1 (*c* 1.5, $CHCl_3$); **^{1}H NMR** (400 MHz, $CDCl_3$, ppm): *δ* 7.37-7.30 (m, 4H), 7.26-7.22 (m, 1H), 3.53 (dd, *J* = 10.8, 3.6 Hz, 1H), 2.70-2.61 (m, 2H), 2.53-2.41 (m, 5H), 2.29 (s, 3H), 1.02 (t, *J* = 7.2 Hz, 6H); **^{13}C NMR** (100 MHz, $CDCl_3$, ppm): *δ* 142.9, 128.3, 127.5, 127.0, 63.7, 61.3, 47.3, 34.8, 12.0; **HRMS** (ESI) m/z calcd. for $C_{13}H_{23}N_2$ [M + H]$^+$: 207.1856, found: 207.1856.

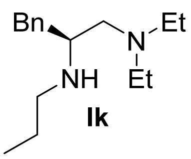

Ik

Following the General Procedure A1, diamine **Ik** was obtained as colorless oil (0.98 g, 69% yield). **[α]$_D$^{20}** = +43.7 (*c* 1.0, $CHCl_3$); **^{1}H NMR** (400 MHz, $CDCl_3$, ppm): *δ* 7.29-7.25 (m, 2H), 7.20-7.16 (m, 3H), 2.88-2.75 (m, 2H), 2.70-2.63 (m, 1H), 2.58-2.45 (m, 5H), 2.41-2.35 (m, 2H), 2.33-2.25 (m, 2H), 1.55-1.46 (m, 2H), 0.94 (t, *J* = 7.2 Hz, 6H), 0.90 (t, *J* = 7.6 Hz, 3H); **^{13}C NMR** (100 MHz, $CDCl_3$, ppm): *δ* 139.8, 129.3, 128.2,

125.9, 57.8, 57.3, 50.0, 47.4, 39.9, 23.2, 12.1, 11.8; **HRMS** (ESI) m/z calcd. for $C_{16}H_{29}N_2$ $[M + H]^+$: 249.2325, found: 249.2328.

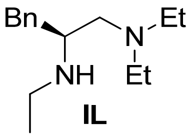

IL

Following the General Procedure A1, diamine **IL** was obtained as colorless oil (1.13 g, 71% yield). $[\alpha]_D^{20}$ = +46.1 (*c* 1.0, $CHCl_3$); ^{1}**H NMR** (600 MHz, $CDCl_3$, ppm): δ 7.28-7.24 (m, 2H), 7.18-7.15 (m, 3H), 2.84-2.81 (m, 2H), 2.74-2.68 (m, 1H), 2.60-2.88 (m, 2H), 2.52-2.46 (m, 2H), 2.41-2.35 (m, 2H), 2.33-2.26 (m, 2H), 2.17 (s, 1H), 1.10-1.07 (m, 3H), 0.96-0.93 (m, 6H); ^{13}**C NMR** (150 MHz, $CDCl_3$, ppm): δ 139.8, 129.3, 128.2, 125.9, 57.8, 57.4, 47.4, 42.2, 40.0, 15.5, 12.0; **HRMS** (ESI) m/z calcd. for $C_{15}H_{27}N_2$ $[M + H]^+$: 235.2169, found: 235.2167.

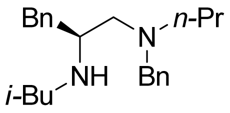

Im

Following the General Procedure A1, diamine **Im** was obtained as colorless oil (1.15 g, 68% yield). $[\alpha]_D^{20}$ = +91.9 (*c* 2.0, $CHCl_3$); ^{1}**H NMR** (400 MHz, $CDCl_3$, ppm): δ 7.28-7.13 (m, 10H), 3.57 (d, J = 13.6 Hz, 1H), 3.36 (d, J = 13.6 Hz, 1H), 2.80-2.69 (m, 2H), 2.55-2.44 (m, 2H), 2.42-2.32 (m, 3H), 2.30-2.21 (m, 3H), 1.75-1.65 (m, 1H), 1.49-1.50 (m, 2H), 0.88 (t, J = 6.4 Hz, 6H), 0.84 (t, J = 7.2 Hz, 3H); ^{13}**C NMR** (100 MHz, $CDCl_3$, ppm): δ 139.9, 139.8, 129.3, 128.9, 128.3, 128.2, 126.8, 126.0, 59.3, 58.6, 58.1, 56.8, 56.2, 39.8, 28.6, 21.0, 20.7, 20.4, 11.9; **HRMS** (ESI) m/z calcd. for $C_{23}H_{35}N_2$ $[M+H]^+$: 339.2795 , found: 339.2794.

Supplementary Note 3

Supplementary Fig. 1 has shown all the chiral amines screened, including our designed chiral acyclic secondary amines **Ia-Im**, pramiray amine **II**, proline **III** and its derivatives **IV**.

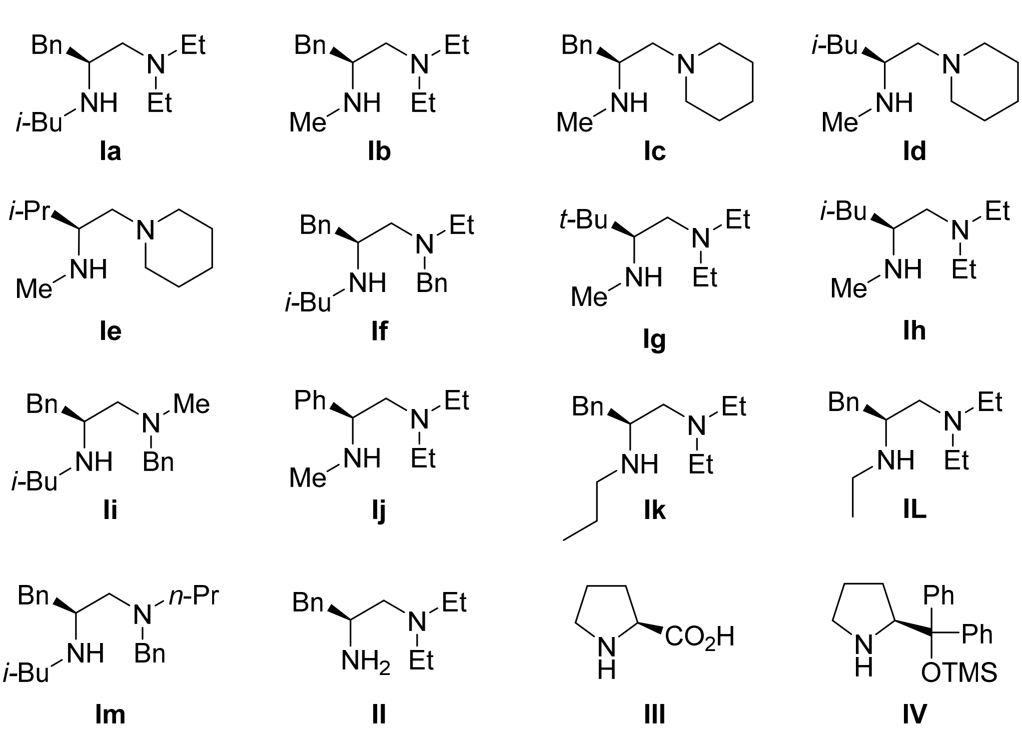

Supplementary Figure 1. Chiral amines screened

Supplementary Table 1. Optimization for Mannich Reaction of Alkynyl-ketimine Ester **1a** and Propionaldehyde **2a**[a]

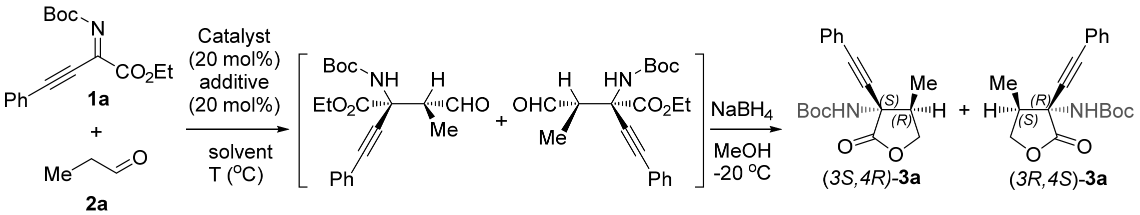

Entry	Catalyst	Solvent	Additive	T (°C)	*t* (h)	yield (%)[b]	dr[c]	ee (%)[d]
1	**Ia**·0.5TfOH	MeCN	MNBA	0	4	84	5:1	(*S*,*R*) 93
2	**Ia**·0.5TfOH	MeCN	MNBA	-20	10	78	7:1	(*S*,*R*) 93
3	**Ia**·0.5TfOH	MeCN	/	-20	12	62	15:1	(*S*,*R*) 93
4	**Ia**·0.5TfOH	MeCN	MNBA	-40	10	77	18:1	(*S*,*R*) 94
5	**Ia**·0.5TfOH	MeCN	PNBA	-40	10	82	> 20:1	(*S*,*R*) 96
6	**Ib**·0.5TfOH	MeCN	PNBA	-40	10	65	7:1	(*R*,*S*) 21
7	**Ic**·0.5TfOH	MeCN	PNBA	-40	14	83	14:1	(*R*,*S*) 66
8	**Id**·0.5TfOH	MeCN	PNBA	-40	14	87	8:1	(*R*,*S*) 84
9	**Ie**·0.5TfOH	MeCN	PNBA	-40	10	82	5:1	(*R*,*S*) 79
10	**Ih**·0.5TfOH	MeCN	PNBA	-40	18	68	3:1	(*R*,*S*) 53
11	**Ij**·0.5TfOH	MeCN	PNBA	-40	14	63	4:1	(*R*,*S*) 28
12	**1k**·0.5TfOH	MeCN	PNBA	-40	14	83	8:1	(*S*,*R*) 88
13	**1L**·0.5TfOH	MeCN	PNBA	-40	14	74	6:1	(*S*,*R*) 81

14	**Id**·0.5TfOH	toluene	PNBA	-40	18	78	10:1	(*R,S*) 83
15	**Id**·0.5TfOH	DCM	PNBA	-40	18	81	9:1	(*R,S*) 87
16	**Id**·0.5TfOH	$CHCl_3$	PNBA	-40	18	78	8:1	(*R,S*) 85
17	**Id**·0.5TfOH	DCE	PNBA	-40	10	78	> 20:1	(*R,S*) 93
18	**II**·0.5TfOH	MeCN	PNBA	-40	48	trace	-	-
19	**III**	MeCN	PNBA	-40	48	trace	-	-
20	**IV**	MeCN	PNBA	-40	48	trace	-	-

[a] Unless otherwise specified, the asymmetric direct Mannich reaction of alkynyl-ketimino ester **1a** (0.1 mmol) and propylaldehyde **2a** (03 mmol) was conducted in the presence of catalyst (20 mol %) and additive (20 mol %) in the indicated solvent (0.8 mL) and temperature. MNBA= *m*-NO_2-$C_6H_4CO_2H$, PNBA = *p*-NO_2-$C_6H_4CO_2H$.

[b] Yield of isolated product.

[c] Determined by ^{1}H NMR or HPLC.

[d] Determined by chiral HPLC.

Supplementary Table 2. Optimization for Mannich Reaction of Trifluoromethylated Alkynyl Ketimines **4a** and Propionaldehyde **2a**[a]

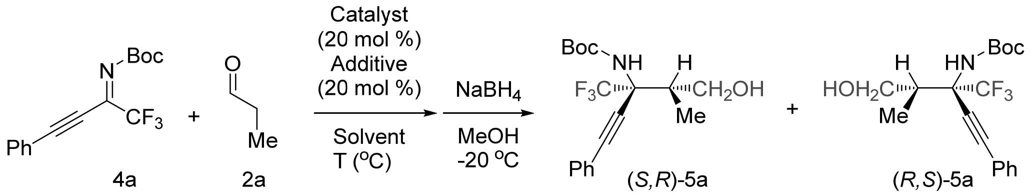

Entry	Catalyst	Solvent	Additive	T (°C)	Time (h)	yield (%)[b]	dr[c]	ee (%)[d]
1	**Ia**·0.5TfOH	THF	/	0	14	63	4:1	(*S,R*) 67
2	**Ia**·0.5TfOH	DCM	/	0	14	51	4:1	(*S,R*) 82
3	**Ia**·0.5TfOH	toluene	/	0	14	48	5:1	(*S,R*) 82
4	**Ia**·0.5TfOH	MeCN	/	0	14	75	5:1	(*S,R*) 85
5	**Ii**·0.5TfOH	MeCN	/	0	14	78	5:1	(*S,R*) 56
6	**Ia**·0.5TfOH	MeCN	MNBA	0	14	86	4:1	(*S,R*) 90
7	**Ia**·0.5TfOH	MeCN	PhCOOH	0	14	79	5:1	(*S,R*) 90
8	**Ia**·0.5TfOH	MeCN	PNBA	0	14	84	6:1	(*S,R*) 91
9	**Ia**·0.5TfOH	MeCN	PNBA	-10	14	82	8:1	(*S,R*) 91
10	**Ia**·0.5TfOH	MeCN	PNBA	-20	18	82	12:	(*S,R*) 91
11	**Ib**·0.5TfOH	MeCN	PNBA	-20	18	48	5:1	(*R,S*) 12
12	**Ic**·0.5TfOH	MeCN	PNBA	-20	24	53	3:1	(*R,S*) 87
13	**Id**·0.5TfOH	MeCN	PNBA	-20	18	53	4:1	(*R,S*) 90
14	**Ie**·0.5TfOH	MeCN	PNBA	-20	24	58	3:1	(*R,S*) 92
15	**Ig**·0.5TfOH	MeCN	PNBA	-20	24	trace	-	-
16	**Ij**·0.5TfOH	MeCN	PNBA	-20	18	49	2:1	(*R,S*) 54

17	**Id**·0.5TfOH	DCE	PNBA	-20	18	41	5:1	(*R,S*) 92
18	**Id**·0.5TfOH	DCM	PNBA	-20	18	45	6:1	(*R,S*) 90
19	**Id**·0.5TfOH	$CHCl_3$	PNBA	-20	18	52	5:1	(*R,S*) 91
20	**Id**·0.5TfOH	toluene	PNBA	-20	18	54	6:1	(*R,S*) 87
21	**Id**·0.5TfOH	MeOH	PNBA	-20	18	67	7:1	(*R,S*) 95
22	**II**·0.5TfOH	MeCN	MNBA	0	24	trace	-	-
23	**III**	MeCN	MNBA	0	14	58	1:2	27
24	**IV**	MeCN	MNBA	0	14	87	1:2	-10

[a] Unless otherwise specified, the asymmetric direct Mannich reaction of **4a** (0.1 mmol) and propylaldehyde **2a** (0.3 mmol) was conducted in the presence of catalyst (20 mol %) and additive (20 mol %) in the indicated solvent (0.8 mL) and temprature. PNBA = *p*-NO_2-$C_6H_4CO_2H$, MNBA= *m*-NO_2-$C_6H_4CO_2H$.

[b] Yield of isolated product.

[c] Determined by ^{1}H NMR or HPLC.

[d] Determined by chiral HPLC.

Supplementary Note 4

General Procedure for the Enantiodivergent Catalytic Mannich Reactions of Alkynyl-ketimine Esters and *α*-Substituted Aldehydes

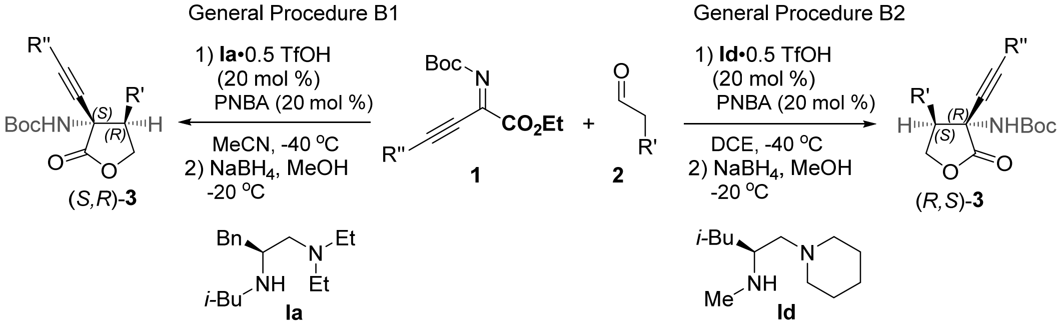

General Procedure B1: Mannich reaction catalyzed by *N*-^{i}Bu amine Ia. To a solution of alkynyl-ketimino esters **1** (0.1 mmol), 4-nitrobenzoic acid (3.3 mg, 0.02 mmol), and catalyst **Ia**·0.5TfOH (6.7 mg, 20 mol%) in anhydrous MeCN (0.8 mL) was added *α*-substituted aldehydes **2** (0.3 mmol) at -40 °C. After the reaction mixture was stirred for the shown time, $NaBH_4$ (19.2 mg, 0.5 mmol) and MeOH (0.5 mL) was added at -20 °C and the resulting mixture was stirred for 0.5 h, then recovery to room temperature and keep stirring for another 1 h. Finally, the resulting mixture was purified by silica gel column chromatography (ethyl acetate:petroleum ether = 1:10 to 1:5) to afford Mannich products (*S*,*R*)-**3**.

General Procedure B2: Mannich reaction catalyzed by *N*-Me amine Id. To a solution of a alkynyl-ketimino esters **1** (0.1 mmol), 4-nitrobenzoic acid (3.3 mg, 0.02 mmol), and catalyst **Id**·0.5TfOH (5.6 mg, 20 mol%) in anhydrous DCE (0.8 mL) was added α-substituted aldehydes **2** (0.3 mmol) at -40 °C. After the reaction mixture was stirred for the shown time, $NaBH_4$ (19.2 mg, 0.5 mmol) and MeOH (0.5 mL) was added at -20 °C and the resulting mixture was stirred for 0.5 h, then recovery to room temperature and keep stirring for 1 h. Finally, the resulting mixture was purified by silica gel column chromatography (ethyl acetate:petroleum ether = 1:10 to 1:5) to afford Mannich products (*R,S*)-**3**.

***Tert*-butyl((3*S*,4*R*)-4-methyl-2-oxo-3-(phenylethynyl)tetrahydrofuran-3-yl) Carbamate**

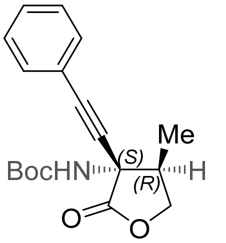

(*S*,*R*)-**3a**

Following the General Procedure B1, (*S,R*)-**3a** was obtained as white solid (10 h, 25.8 mg, 82% yield, >20:1 *anti/syn*, 96% ee) after flash chromatography (elution gradient: ethyl acetate:petroleum ether = 1:10); $[\alpha]_D^{20}$ = −18.6 (*c* 1.0, $CHCl_3$); **^{1}H NMR** (600 MHz, $CDCl_3$, ppm): δ 7.45-7.43 (m, 2H), 7.38 (t, *J* = 7.2 Hz, 1H), 7.35-7.32 (m, 2H), 5.35 (s, 1H), 4.46 (t, *J* = 8.4 Hz, 1H), 3.94 (dd, *J* = 9.0, 11.4 Hz, 1H), 3.41 (s, 1H), 1.47 (s, 9H), 1.28 (d, *J* = 6.6 Hz, 3H); **^{13}C NMR** (150 MHz, $CDCl_3$, ppm): δ 171.8, 153.9, 132.0, 129.5, 128.5, 120.9, 89.6, 81.2, 79.5, 71.0, 59.6, 38.2, 28.2, 11.5; **HPLC analysis:** Daicel CHIRALPAK IC, *n*-hexane:*i*-PrOH = 80:20, flow rate = 1.0 mL·min^{-1}, λ = 254 nm, retention time: t_R = 11.2 min (minor), t_R = 14.4 min (major); **HRMS** (ESI) m/z calcd. for $C_{18}H_{21}NNaO_4$ $[M + Na]^+$: 338.1363, found: 338.1367.

***Tert*-butyl((3*R*,4*S*)-4-methyl-2-oxo-3-(phenylethynyl)tetrahydrofuran-3-yl) carbamate**

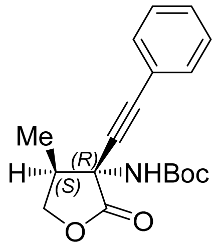

(*R,S*)-**3a**

Following the General Procedure B2, (*R,S*)-**3a** was obtained as white solid (10 h, 24.4 mg, 78% yield, >20:1 *anti/syn*, 93% ee) after flash chromatography (elution gradient: ethyl acetate:petroleum ether = 1:10); **HPLC analysis:** Daicel CHIRALPAK IC, *n*-hexane:*i*-PrOH = 80:20, flow rate = 1.0 mL·min^{-1}, λ = 254 nm, retention time: t_R = 11.1 min (major), t_R = 14.4 min (minor).

***Tert*-butyl((3*S*,4*R*)-3-((4-methoxyphenyl)ethynyl)-4-methyl-2-oxotetrahydrofuran-3-yl)carbamate**

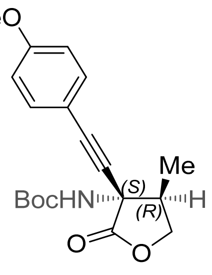

(*S,R*)-**3b**

Following the General Procedure B1, (*S,R*)-**3b** was obtained as white solid (14 h, 28.3 mg, 82% yield, >20:1 *anti/syn*, 98% ee) after flash chromatography (elution gradient: ethyl acetate:petroleum ether = 1:5); **$[\alpha]_D^{20}$** = -6.1 (*c* 1.0, $CHCl_3$); **^{1}H NMR** (600 MHz, $CDCl_3$, ppm): δ 7.37 (d, J = 8.4 Hz, 2H), 6.84 (d, J = 9 Hz, 2H), 5.31 (s, 1H), 4.45 (t, J = 7.8 Hz, 1H), 3.92 (dd, J = 9.0, 11.4 Hz, 1H), 3.82 (s, 3H), 3.40 (s, 1H), 1.46 (s, 9H), 1.26 (d, J = 6.6 Hz, 3H); **^{13}C NMR** (150 MHz, $CDCl_3$, ppm): δ 171.9, 160.5, 153.9, 133.6, 114.1, 112.9, 89.8, 81.2, 78.3, 70.9, 59.6, 55.3, 38.2, 28.2, 11.4; **HPLC analysis:** Daicel CHIRALPAK IC, *n*-hexane:*i*-PrOH = 80:20, flow rate = 1.0 mL·min^{-1}, λ = 254 nm, retention time: t_R = 19.1 min (minor), t_R = 23.6 min (major); **HRMS** (ESI) m/z calcd.for $C_{19}H_{23}NNaO_5$ $[M + Na]^+$: 368.1468, found: 368.1471.

***Tert*-butyl((3*R*,4*S*)-3-((4-methoxyphenyl)ethynyl)-4-methyl-2-oxotetrahydrofuran-3-yl)carbamate**

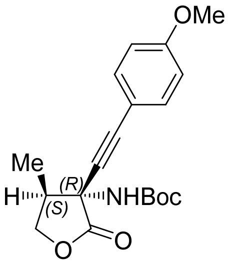

(*R,S*)-**3b**

Following the General Procedure B2, (*R,S*)-**3b** was obtained as white solid (14 h, 27.9 mg, 81% yield, >20:1 *anti/syn*, 91% ee) after flash chromatography (elution gradient: ethyl acetate:petroleum ether = 1:5); **HPLC analysis:** Daicel CHIRALPAK IC, *n*-hexane:*i*-PrOH = 80:20, flow rate = 1.0 mL·min^{-1}, λ = 254 nm, retention time: t_R = 19.1 min (major), t_R = 24.1 min (minor).

***Tert*-butyl((3*S*,4*R*)-4-methyl-2-oxo-3-(p-tolylethynyl)tetrahydrofuran-3-yl) carbamate**

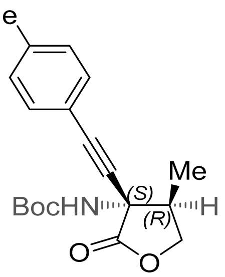

(*S,R*)-**3c**

Following the General Procedure B1, (*S,R*)-**3c** was obtained as white solid (14 h, 26.9 mg, 82% yield, >20:1 *anti/syn*, 98% ee) after flash chromatography (elution gradient: ethyl acetate:petroleum ether = 1:10); $[\alpha]_D^{20}$ = +21.6 (*c* 1.0, $CHCl_3$); **^{1}H NMR** (600 MHz, $CDCl_3$, ppm): δ 7.33 (d, J = 8.4 Hz, 2H), 7.13 (d, J = 7.8 Hz, 2H), 5.28 (s, 1H), 4.45 (t, J = 8.4 Hz, 1H), 3.93 (dd, J = 8.4, 10.8 Hz, 1H), 3.40 (s, 1H), 2.36 (s, 3H), 1.46 (s, 9H), 1.27 (d, J = 6.6 Hz, 3H); **^{13}C NMR** (150 MHz, $CDCl_3$, ppm): δ 171.8, 153.7, 139.8, 131.9, 129.2, 117.9, 89.9, 80.4, 78.9, 70.9, 59.6, 38.4, 28.2, 21.5, 11.4; **HPLC analysis:** Daicel CHIRALPAK IA, *n*-hexane:*i*-PrOH = 95:5, flow rate = 1.0 mL·min^{-1}, λ = 254 nm, retention time: t_R = 13.1 min (minor), t_R = 16.3 min (major); **HRMS** (ESI)

m/z calcd. for $C_{19}H_{23}NNaO_4$ $[M + Na]^+$: 352.1519, found:352.1519.

***Tert*-butyl((3*R*,4*S*)-4-methyl-2-oxo-3-(p-tolylethynyl)tetrahydrofuran-3-yl) carbamate**

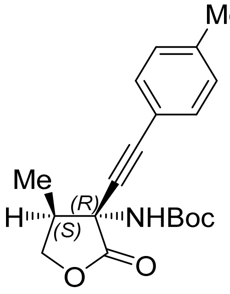

(*R*,*S*)-**3c**

Following the General Procedure B2, (*R,S*)-**3c** was obtained as white solid (14 h, 27.3 mg, 83% yield, >20:1 *anti/syn*, 92% ee) after flash chromatography (elution gradient: ethyl acetate:petroleum ether = 1:10); **HPLC analysis:** Daicel CHIRALPAK IA, *n*-hexane:*i*-PrOH = 95:5, flow rate = 1.0 mL·min^{-1}, λ = 254 nm, retention time: t_R = 13.1 min (major), t_R =15.9 min (minor).

***Tert*-butyl((3*S*,4*R*)-4-methyl-2-oxo-3-(m-tolylethynyl)tetrahydrofuran-3-yl) carbamate**

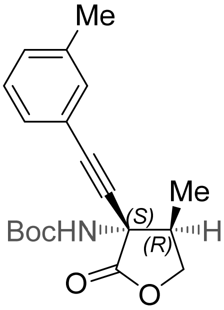

(*S,R*)-**3d**

Following the General Procedure B1, (*S,R*)-**3d** was obtained as white solid (14 h, 27.6 mg, 84% yield, >20:1 *anti/syn*, 98% ee) after flash chromatography (elution gradient: ethyl acetate:petroleum ether = 1:10); $[\alpha]_D^{20}$ = +15.3 (*c* 1.0, $CHCl_3$); **^{1}H NMR** (600 MHz, $CDCl_3$, ppm): *δ* 7.26-7.20 (m, 3H), 7.19-7.17 (m, 1H), 5.30 (s, 1H), 4.46 (t, *J* = 7.8 Hz, 1H), 3.93 (dd, *J* = 9.0, 11.4 Hz, 1H), 3.40 (s, 1H), 2.33 (s, 3H), 1.46 (s, 9H), 1.27 (d, *J* = 6.0 Hz, 3H); **^{13}C NMR** (150 MHz, $CDCl_3$, ppm): *δ* 171.8, 153.9, 138.2, 132.6, 130.4, 129.1, 128.4, 120.7, 89.9, 81.2, 79.2, 71.0, 59.6, 38.3, 28.2, 21.1, 11.5;

HPLC analysis: Daicel CHIRALPAK IC, *n*-hexane:*i*-PrOH = 90:10, flow rate = 1.0 mL·min^{-1}, λ = 254 nm, retention time: t_R = 28.0 min (minor), t_R = 34.2 min (major); **HRMS** (ESI) m/z calcd. for $C_{19}H_{23}NNaO_4$ $[M + Na]^+$: 352.1519, found:352.1523.

***Tert*-butyl((3*R*,4*S*)-4-methyl-2-oxo-3-(m-tolylethynyl)tetrahydrofuran-3-yl) carbamate**

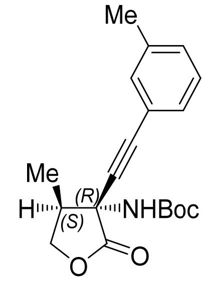

(*R*,*S*)-**3d**

Following the General Procedure B2, (*R,S*)-**3d** was obtained as white solid (14 h, 26.9 mg, 82% yield, >20:1 *anti/syn*, 96% ee) after flash chromatography (elution gradient: ethyl acetate:petroleum ether = 1:10); **HPLC analysis:** Daicel CHIRALPAK IC, *n*-hexane:*i*-PrOH = 90:10, flow rate = 1.0 mL·min^{-1}, λ = 254 nm, retention time: t_R = 27.6 min (major), t_R = 34.5 min (minor).

***Tert*-butyl((3*S*,4*R*)-3-((4-chlorophenyl)ethynyl)-4-methyl-2-oxotetrahydrofuran-3-yl)carbamate**

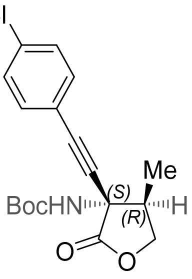

(*S*,*R*)-**3e**

Following the General Procedure B1, (*S,R*)-**3e** was obtained as white solid (14 h, 28.6 mg, 82% yield, >20:1 *anti/syn*, 99% ee) after flash chromatography (elution gradient: ethyl acetate:petroleum ether = 1:8); $[\alpha]_D^{20}$ = −8.4 (*c* 1.0, $CHCl_3$); **^{1}H NMR** (600 MHz, $CDCl_3$, ppm): *δ* 7.38-7.36 (m, 2H), 7.32-7.29 (m, 2H), 5.30 (s, 1H), 4.46 (t, *J* = 7.8 Hz, 1H), 3.92 (dd, *J* = 9.0, 10.8 Hz, 1H), 3.39 (s, 1H), 1.46 (s, 9H), 1.27 (d, *J* = 6.6 Hz, 3H); **^{13}C NMR** (150 MHz, $CDCl_3$, ppm): *δ* 171.6, 153.9, 135.7, 133.2, 128.9, 119.4, 88.4,

81.4, 80.7, 71.0, 59.6, 38.4, 28.2, 11.6; **HPLC analysis:** Daicel CHIRALPAK IC, *n*-hexane:*i*-PrOH = 80:20, flow rate = 1.0 mL·min^{-1}, λ = 254 nm, retention time: t_R = 10.8 min (minor), t_R = 14.3 min (major); **HRMS** (ESI) m/z calcd. for $C_{18}H_{20}ClNNaO_4$ [M + Na]$^+$: 372.0973, found: 372.0977.

***Tert*-butyl((3*R*,4*S*)-3-((4-chlorophenyl)ethynyl)-4-methyl-2-oxotetrahydrofuran-3-yl)carbamate**

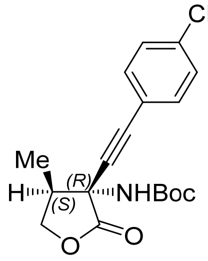

(*R*,*S*)-**3e**

Following the General Procedure B2, (*R,S*)-**3e** was obtained as white solid (14 h, 27.2 mg, 78 % yield, >20:1 *anti/syn*, 96 % ee) after flash chromatography (elution gradient: ethyl acetate:petroleum ether = 1:8); **HPLC analysis:** Daicel CHIRALPAK IC, *n*-hexane:*i*-PrOH = 80:20, flow rate = 1.0 mL·min^{-1}, λ = 254 nm, retention time: t_R = 10.8 min (major), t_R = 14.4 min (minor).

***Tert*-butyl((3*S*,4*R*)-3-((2-fluorophenyl)ethynyl)-4-methyl-2-oxotetrahydrofuran-3-yl)carbamate**

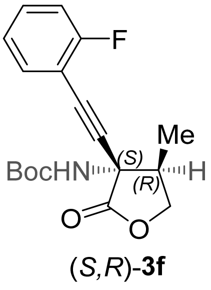

(*S*,*R*)-**3f**

Following the General Procedure B1, (*S,R*)-**3f** was obtained as white solid (14 h, 26.6 mg, 80% yield, >20:1 *anti/syn*, 98% ee) after flash chromatography (elution gradient: ethyl acetate:petroleum ether = 1:10); $[\alpha]_D^{20}$ = +15.7 (*c* 1.0, $CHCl_3$); ^{1}**H NMR** (600 MHz, $CDCl_3$, ppm): *δ* 7.42 (t, *J* = 7.2 Hz, 1H), 7.39-7.35 (m, 1H), 7.13-7.07 (m, 2H), 5.35 (s, 1H), 4.47 (t, *J* = 8.4 Hz, 1H), 3.96 (dd, *J* = 9.0, 11.4 Hz, 1H), 3.41 (s, 1H), 1.47 (s, 9H), 1.30 (d, *J* = 6.6 Hz, 3H); ^{13}**C NMR** (150 MHz, $CDCl_3$, ppm): *δ* 171.5, 163.1

(d, $^1J_{CF}$ = 253.7 Hz), 153.9, 133.6, 131.3 (d, $^3J_{CF}$ = 7.8 Hz), 124.1 (d, $^4J_{CF}$ = 3.3 Hz), 115.6 (d, $^2J_{CF}$ = 20.0 Hz), 109.7 (d, $^2J_{CF}$ = 15.4 Hz), 84.8, 83.1, 81.4, 71.0, 59.8, 38.5, 28.2, 11.3; **^{19}F NMR** (565 MHz, $CDCl_3$, ppm): *δ* -109.1 (s); **HPLC analysis:** Daicel CHIRALPAK IC, *n*-hexane:*i*-PrOH = 90:10, flow rate = 1.0 mL·min^{-1}, λ = 254 nm, retention time: t_R = 23.1 min (major), t_R = 25.1 min (minor); **HRMS** (ESI) m/z calcd. for $C_{18}H_{20}FNNaO_4$ [M + Na]$^+$: 356.1269, found: 356.1271.

***Tert*-butyl((3*R*,4*S*)-3-((2-fluorophenyl)ethynyl)-4-methyl-2-oxotetrahydrofuran-3-yl)carbamate**

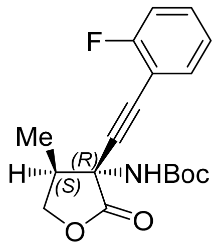

(*R,S*)-**3f**

Following the General Procedure B2, (*R,S*)-**3f** was obtained as white solid (14 h, 26.9 mg, 81% yield, >20:1 *anti/syn*, 96% ee) after flash chromatography (elution gradient: ethyl acetate:petroleum ether = 1:10); **HPLC analysis:** Daicel CHIRALPAK IC, *n*-hexane:*i*-PrOH = 90:10, flow rate = 1.0 mL·min^{-1}, λ = 254 nm, retention time: t_R = 23.2 min (minor), t_R = 24.7 min (major).

***Tert*-butyl((3*S*,4*R*)-4-methyl-2-oxo-3-(thiophen-2-ylethynyl)tetrahydrofuran-3-yl)carbamate**

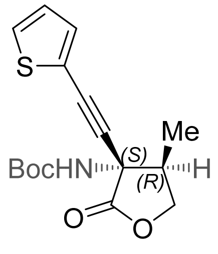

(*S,R*)-**3g**

Following the General Procedure B1, (*S,R*)-**3g** was obtained as white solid (14 h, 25.0 mg, 78% yield, 10:1 *anti/syn*, 93% ee) after flash chromatography (elution gradient: ethyl acetate:petroleum ether = 1:8); **$[\alpha]_D^{20}$** = +17.2 (*c* 1.0, $CHCl_3$); **^{1}H NMR** (600 MHz, $CDCl_3$, ppm): *δ* 7.33 (dd, *J* = 0.6, 4.8 Hz, 1H), 7.26 (d, *J* = 4.8 Hz, 1H), 6.99 (dd, *J* =

3.6, 5.4 Hz, 1H), 5.30 (s, 1H), 4.46 (t, J = 8.4 Hz, 1H), 3.92 (dd, J = 9.0, 10.8 Hz, 1H), 3.40 (s, 1H), 1.46 (s, 9H), 1.26 (d, J = 6.6 Hz, 3H); **^{13}C NMR** (150 MHz, $CDCl_3$, ppm): δ 171.5, 153.8, 133.8, 128.6, 127.1, 120.6, 83.4, 83.1, 81.3, 70.9, 59.8, 38.5, 28.2, 11.5; **HPLC analysis:** Daicel CHIRALPAK AD-H, *n*-hexane:*i*-PrOH = 90:10, flow rate = 1.0 mL·min^{-1}, λ = 254 nm, retention time: t_R = 8.8 min (major), t_R =.11.7 min (minor); **HRMS** (ESI) m/z calcd. for $C_{16}H_{19}NNaO_4S$ $[M + Na]^+$: 344.0927, found: 344.0926.

***Tert*-butyl((3*R*,4*S*)-4-methyl-2-oxo-3-(thiophen-2-ylethynyl)tetrahydrofuran-3-yl)carbamate**

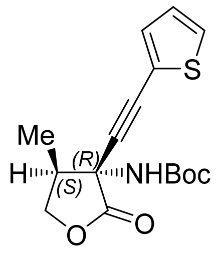

(*R,S*)-**3g**

Following the General Procedure B2, (*R,S*)-**3g** was obtained as white solid (14 h, 23.1 mg, 72% yield, 13:1 *anti/syn*, 90% ee) after flash chromatography (elution gradient: ethyl acetate:petroleum ether = 1:8); **HPLC analysis:** Daicel CHIRALPAK AD-H, *n*-hexane:*i*-PrOH = 90:10, flow rate = 1.0 mL·min^{-1}, λ = 254 nm, retention time: t_R = 8.9 min (minor), t_R = 12.2 min (major).

***Tert*-butyl((3*S*,4*R*)-4-methyl-2-oxo-3-(4-phenylbut-1-yn-1-yl)tetrahydrofuran-3-yl)carbamate**

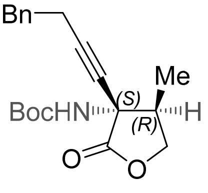

(*S,R*)-**3h**

Following the General Procedure B1, (*S,R*)-**3h** was obtained as white solid (14 h, 26.4 mg, 77% yield, >20:1 *anti/syn*, 98% ee) after flash chromatography (elution gradient: ethyl acetate:petroleum ether = 1:10); **$[\alpha]_D^{20}$** = +12.8 (c 1.0, $CHCl_3$); **^{1}H NMR** (600 MHz, $CDCl_3$, ppm): δ 7.30 (t, J = 7.2 Hz, 2H), 7.22 (t, J = 7.8 Hz, 1H), 7.17 (d, J = 7.8 Hz, 1H), 5.06 (s, 1H), 4.31 (t, J = 7.8 Hz, 1H), 3.64 (dd, J = 8.4, 10.8 Hz, 1H), 3.21 (s, 1H), 2.81 (t, J = 6.6 Hz, 1H), 2.54 (t, J = 7.2 Hz, 1H), 1.43 (s, 9H), 1.02 (d, J = 6.6 Hz,

3H); **^{13}C NMR** (150 MHz, $CDCl_3$, ppm): δ 172.1, 153.9, 139.9, 128.5, 128.4, 126.6, 90.0, 81.0, 72.1, 70.8, 59.1, 37.8, 34.4, 28.2, 20.7, 11.2; **HPLC analysis:** Daicel CHIRALPAK IC, *n*-hexane:*i*-PrOH = 80:20, flow rate = 1.0 mL·min^{-1}, λ = 210 nm, retention time: t_R = 17.2 min (minor), t_R = 25.4 min (major); **HRMS** (ESI) m/z calcd. for $C_{20}H_{25}NNaO_4$ $[M + Na]^+$: 366.1676, found: 366.1676.

***Tert*-butyl((3*R*,4*S*)-4-methyl-2-oxo-3-(4-phenylbut-1-yn-1-yl)tetrahydrofuran-3-yl)carbamate**

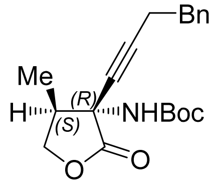

(*R,S*)-**3h**

Following the General Procedure B2, (*R,S*)-**3h** was obtained as white solid (14 h, 23.3 mg, 68% yield, >20:1 *anti/syn*, 90% ee) after flash chromatography (elution gradient: ethyl acetate:petroleum ether = 1:10); **HPLC analysis:** Daicel CHIRALPAK IC, *n*-hexane:*i*-PrOH = 80:20, flow rate = 1.0 mL·min^{-1}, λ = 210 nm, retention time: t_R = 17.8 min (major), t_R = 26.2 min (minor).

***Tert*-butyl((3*S*,4*R*)-4-ethyl-2-oxo-3-(phenylethynyl)tetrahydrofuran-3-yl) carbamate**

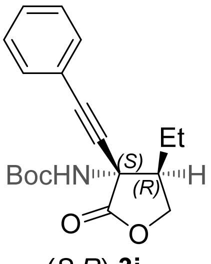

(*S,R*)-**3i**

Following the General Procedure B1, (*S,R*)-**3i** was obtained as white solid (14 h, 23.3 mg, 75% yield, >20:1 *anti/syn*, 95% ee) after flash chromatography (elution gradient: ethyl acetate:petroleum ether = 1:10); **$[\alpha]_D^{20}$** = +18.7 (*c* 1.0, $CHCl_3$); **^{1}H NMR** (600 MHz, $CDCl_3$, ppm): δ 7.46-7.43 (m, 2H), 7.39-7.36 (m, 1H), 7.34-7.32 (m, 2H), 5.31 (s, 1H), 4.52 (t, *J* = 8.4 Hz, 1H), 3.95 (dd, *J* = 9.0, 10.8 Hz, 1H), 3.29 (s, 1H), 1.93-1.86 (m, 1H), 1.71-1.63 (m, 1H), 1.46 (s, 9H), 1.03 (t, *J* = 7.8 Hz, 3H); **^{13}C NMR** (150 MHz,

$CDCl_3$, ppm): δ 171.9, 153.9, 132.0, 129.5, 128.5, 120.9, 89.4, 81.2, 79.8, 70.2, 59.2, 44.3, 28.3, 21.3, 11.5; **HPLC analysis:** Daicel CHIRALPAK IC, *n*-hexane:*i*-PrOH = 80:20, flow rate = 1.0 mL·min^{-1}, λ = 254 nm, retention time: t_R = 14.3 min (minor), t_R = 17.3 min (major); **HRMS** (ESI) m/z calcd.for $C_{19}H_{23}NNaO_4$ $[M + Na]^+$: 352.1519, found: 352.1517.

***Tert*-butyl((3*R*,4*S*)-4-ethyl-2-oxo-3-(phenylethynyl)tetrahydrofuran-3-yl) carbamate**

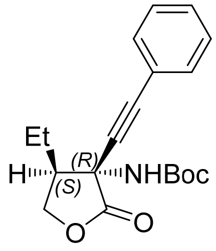

(*R,S*)-**3i**

Following the General Procedure B2, (*R,S*)-**3i** was obtained as white solid (14 h, 23.5 mg, 76% yield, 18:1 *anti/syn*, 90% ee) after flash chromatography (elution gradient: ethyl acetate:petroleum ether = 1:10); **HPLC analysis:** Daicel CHIRALPAK IC, *n*-hexane:*i*-PrOH = 80:20, flow rate = 1.0 mL·min^{-1}, λ = 254 nm, retention time: t_R = 14.3 min (major), t_R = 17.4 min (minor).

***Tert*-butyl((3*S*,4*R*)-4-benzyl-2-oxo-3-(phenylethynyl)tetrahydrofuran-3-yl) carbamate**

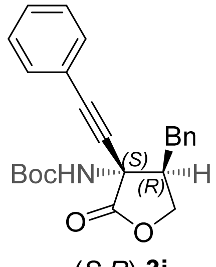

(*S,R*)-**3j**

Following the General Procedure B1, (*S,R*)-**3j** was obtained as white solid (14 h, 31.2 mg, 80% yield, >20:1 *anti/syn*, 93% ee) after flash chromatography (elution gradient: ethyl acetate:petroleum ether = 1:10); $[\alpha]_D^{20}$ = +45.8 (*c* 1.0, $CHCl_3$); **^{1}H NMR** (600 MHz, $CDCl_3$, ppm): δ 7.51-7.49 (m, 2H), 7.43-7.40 (m, 1H), 7.38-7.35 (m, 2H), 7.31 (t, *J* = 7.8 Hz, 2H), 7.25-7.21 (m, 3H), 5.08 (s, 1H), 4.35 (t, *J* = 7.2 Hz, 1H), 4.06 (dd, *J* = 9.0, 10.8 Hz, 1H), 3.73 (s, 1H), 3.19 (dd, *J* = 6.6, 13.2 Hz, 1H), 2.92 (dd, *J* = 9.0,

14.4 Hz, 1H), 1.43 (s, 9H); **^{13}C NMR** (150 MHz, $CDCl_3$, ppm): δ 171.4, 153.6, 137.8, 132.1, 129.7, 128.8, 128.6, 128.5, 126.8, 120.8, 90.0, 81.1, 79.7, 69.9, 59.0, 44.1, 34.1, 28.2; **HPLC analysis:** Daicel CHIRALPAK IC, *n*-hexane:*i*-PrOH = 90:10, flow rate = 1.0 mL·min^{-1}, λ = 254 nm, retention time: t_R = 26.1 min (minor), t_R = 47.0 min (major); **HRMS** (ESI) m/z calcd. for $C_{24}H_{25}NNaO_4$ [M + Na]$^+$: 414.1676, found: 414.1677.

***Tert*-butyl((3*R*,4*S*)-4-benzyl-2-oxo-3-(phenylethynyl)tetrahydrofuran-3-yl) carbamate**

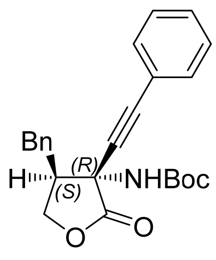

(*R,S*)-**3j**

Following the General Procedure B2, (*R,S*)-**3j** was obtained as white solid (14 h, 31.4 mg, 81% yield, >20:1 *anti/syn*, 91% ee) after flash chromatography (elution gradient: ethyl acetate:petroleum ether = 1:10); **HPLC analysis:** Daicel CHIRALPAK IC, *n*-hexane:*i*-PrOH = 90:10, flow rate = 1.0 mL·min^{-1}, λ = 254 nm, retention time: t_R = 27.2 min (major), t_R = 46.6 min (minor).

***Tert*-butyl((3*S*,4*R*)-4-allyl-2-oxo-3-(phenylethynyl)tetrahydrofuran-3-yl) carbamate**

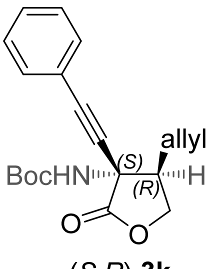

(*S,R*)-**3k**

Following the General Procedure B1, (*S,R*)-**3k** was obtained as white solid (14 h, 26.2 mg, 77% yield, >20:1 *anti/syn*, 98% ee) after flash chromatography (elution gradient: ethyl acetate:petroleum ether = 1:10); **$[\alpha]_D^{20}$** = +16.8 (*c* 1.0, $CHCl_3$); **^{1}H NMR** (600 MHz, $CDCl_3$, ppm): δ 7.46-7.43 (m, 2H), 7.39-7.37 (m, 1H), 7.34-7.32 (m, 2H), 5.83-5.76 (m, 1H), 5.28 (s, 1H), 5.17 (dd, *J* = 1.8, 17.4 Hz, 1H), 5.09 (d, *J* = 10.2 Hz, 1H),

4.47 (t, J = 8.4 Hz, 1H), 3.96 (dd, J = 9.6, 11.4 Hz, 1H), 3.44 (s, 1H), 2.68-2.63 (m, 1H), 2.39-2.34 (m, 1H), 1.46 (s, 9H); **^{13}C NMR** (150 MHz, $CDCl_3$, ppm): δ 171.5, 153.9, 134.1, 132.0, 129.6, 128.5, 120.8, 117.5, 89.7, 81.3, 79.7, 69.9, 58.9, 42.4, 32.6, 28.2; **HPLC analysis:** Daicel CHIRALPAK IC, *n*-hexane:*i*-PrOH = 90:10, flow rate = 1.0 mL·min^{-1}, λ = 254 nm, retention time: t_R = 22.8 min (minor), t_R = 33.6 min (major); **HRMS** (ESI) m/z calcd.for $C_{20}H_{23}NNaO_4$ [M + Na]$^+$: 364.1519, found: 364.1518.

***Tert*-butyl((3*R*,4*S*)-4-allyl-2-oxo-3-(phenylethynyl)tetrahydrofuran-3-yl) carbamate**

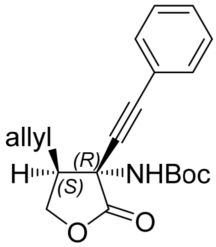

(*R,S*)-**3k**

Following the General Procedure B2, (*R,S*)-**3k** was obtained as white solid (14 h, 23.2 mg, 68% yield, >20:1 *anti/syn*, 90% ee) after flash chromatography (elution gradient: ethyl acetate:petroleum ether = 1:10); **HPLC analysis:** Daicel CHIRALPAK IC, *n*-hexane:*i*-PrOH = 90:10, flow rate = 1.0 mL·min^{-1}, λ = 254 nm, retention time: t_R = 22.9 min (major), t_R = 33.4 min (minor).

***Tert*-butyl((3*S*,4*R*)-4-butyl-2-oxo-3-(phenylethynyl)tetrahydrofuran-3-yl) carbamate**

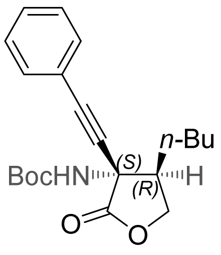

(*S,R*)-**3l**

Following the General Procedure B1, (*S,R*)-**3l** was obtained as white solid (14 h, 28.5 mg, 80% yield, >20:1 *anti/syn*, 98% ee) after flash chromatography (elution gradient: ethyl acetate:petroleum ether = 1:10); **$[\alpha]_D^{20}$** = −25.4 (*c* 1.0, $CHCl_3$); **^{1}H NMR** (600 MHz, $CDCl_3$, ppm): δ 7.45-7.42 (m, 2H), 7.39-7.37 (m, 1H), 7.34-7.32 (m, 2H), 5.31

(s, 1H), 4.49 (t, J = 7.8 Hz, 1H), 3.95 (dd, J = 9.0, 10.8 Hz, 1H), 3.37 (s, 1H), 1.88-1.82 (m, 1H), 1.63-1.57 (m, 1H), 1.46 (s, 9H), 1.40-1.32 (m, 4H), 0.93 (t, J = 7.2 Hz, 3H); **^{13}C NMR** (150 MHz, $CDCl_3$, ppm): δ 171.9, 153.9, 132.0, 129.5, 128.5, 120.9, 89.5, 81.2, 79.8, 70.3, 59.2, 42.5, 29.0, 28.2, 27.7, 22.8, 13.9; **HPLC analysis:** Daicel CHIRALPAK IC, *n*-hexane:*i*-PrOH = 90:10, flow rate = 1.0 mL·min^{-1}, λ = 254 nm, retention time: t_R = 27.4 min (minor), t_R = 35.1 min (major); **HRMS** (ESI) m/z calcd. for $C_{21}H_{27}NNaO_4$ $[M + Na]^+$: 380.1832, found: 380.1834.

***Tert*-butyl((3*R*,4*S*)-4-butyl-2-oxo-3-(phenylethynyl)tetrahydrofuran-3-yl) carbamate**

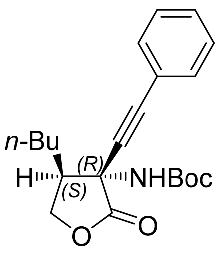

(*R*,*S*)-**3l**

Following the General Procedure B2, (*R*,*S*)-**3l** was obtained as white solid (14 h, 25.3 mg, 71% yield, 13:1 *anti/syn*, 90% ee) after flash chromatography (elution gradient: ethyl acetate:petroleum ether = 1:10); **HPLC analysis:** Daicel CHIRALPAK IC, *n*-hexane:*i*-PrOH = 90:10, flow rate = 1.0 mL·min^{-1}, λ = 254 nm, retention time: t_R = 26.9 min (major), t_R = 35.4 min (minor).

Supplementary Note 5

General Procedure for Asymmetry Catalyzed Mannich Reactions of Trifluoromethylated Alkynyl Ketimines and *α*-Substituted Aldehydes

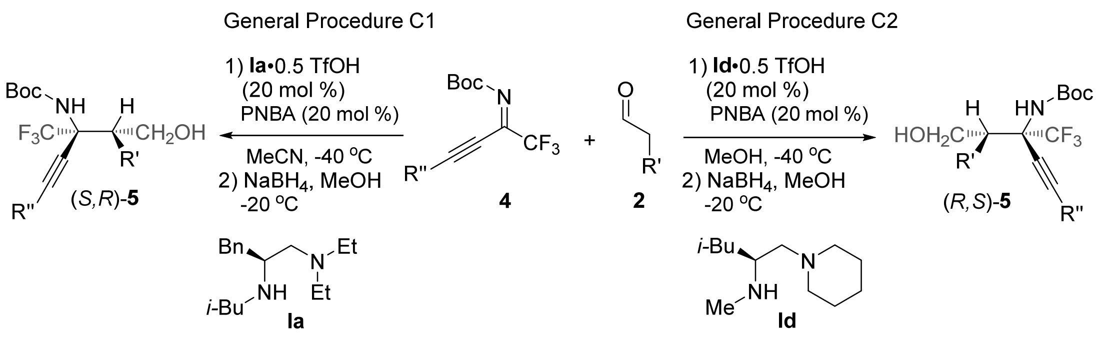

General Procedure C1: Mannich reaction catalyzed by *N*-^{i}Bu amine Ia. To a solution of trifluoromethylated alkynyl ketimines **4** (0.1 mmol), 4-nitrobenzoic acid (3.3 mg, 0.02 mmol), and catalyst **Ia**·0.5TfOH (6.7 mg, 20 mol%) in anhydrous MeCN (0.8 mL) was added α-substituted aldehydes **2** (0.3 mmol) at -20 °C. After the reaction mixture was stirred for the shown time, $NaBH_4$ (19.2 mg, 0.5 mmol) and MeOH (0.5 mL) was added at -20 °C and the resulting mixture was stirred for 0.5 h. Finally, the resulting mixture was purified by silica gel column chromatography (ethyl acetate:petroleum ether = 1:5 to 1:3) to afford Mannich products (*S*,*R*)-**5**.

General Procedure C2: Mannich reaction catalyzed by *N*-Me amine Id: To a solution of trifluoromethylated alkynyl ketimines **4** (0.1 mmol), 4-nitrobenzoic acid (3.3 mg, 0.02 mmol), and catalyst **Id**·0.5TfOH (5.6 mg, 20 mol%) in anhydrous MeOH (0.8 mL) was added α-substituted aldehydes **2** (0.3 mmol) at -20 °C. After the reaction mixture was stirred for the shown time, $NaBH_4$ (19.2 mg, 0.5 mmol) was added at -20 °C and the resulting mixture was stirred for 0.5 h. Finally, the resulting mixture was purified by silica gel column chromatography (ethyl acetate:petroleum ether = 1:5 to 1:3) to afford Mannich products (*R*,*S*)-**5**.

***Tert*-butyl((3*S*,4*R*)-5-hydroxy-4-methyl-1-phenyl-3-(trifluoromethyl)pent-1-yn-3-yl)carbamate**

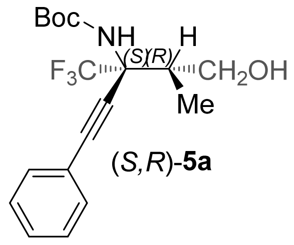

(*S,R*)-**5a**

Following the General Procedure C1, (*S,R*)-**5a** was obtained as white solid (18 h, 29.3 mg, 82% yield, 12:1 *anti/syn*, 91% ee) after flash chromatography (elution gradient: ethyl acetate:petroleum ether = 1:5); **[α]$_D$^{20}** = −21.6 (*c* 1.0, $CHCl_3$); **^{1}H NMR** (600 MHz, $CDCl_3$, ppm): δ 7.48-7.46 (m, 2H), 7.36-7.31 (m, 3H), 5.36 (s, 1H), 3.93-3.89 (m, 2H), 2.73 (s, 1H), 2.47 (s, 1H), 1.47 (s, 9H), 1.24 (d, J = 6.6 Hz, 3H); **^{13}C NMR** (150 MHz, $CDCl_3$, ppm): δ 153.6, 131.9, 129.2, 128.4, 125.6 (q, $^1J_{CF}$ = 289.5 Hz), 121.4, 87.9, 81.5, 81.0, 64.4, 60.9 (q, $^2J_{CF}$ = 28.5 Hz) , 40.2, 28.2, 12.3; **^{19}F NMR** (565 MHz, $CDCl_3$,

ppm): δ -72.5 (s); **HPLC analysis:** Daicel CHIRALPAK OJ-H, *n*-hexane:*i*-PrOH = 96:4, flow rate = 1.0 mL·min^{-1}, λ = 254 nm, retention time: t_R = 8.7 min (major), t_R = 10.3 min (minor); **HRMS** (ESI) m/z calcd. for $C_{18}H_{22}F_3NNaO_3$ $[M + Na]^+$: 380.1444, found: 380.1445.

***Tert*-butyl((3*R*,4*S*)-5-hydroxy-4-methyl-1-phenyl-3-(trifluoromethyl)pent-1-yn-3-yl)carbamate**

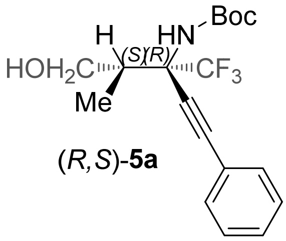

(*R,S*)-**5a**

Following the General Procedure C2, (*R,S*)-**5a** was obtained as white solid (18 h, 23.9 mg, 67% yield, 7:1 *anti/syn*, 95% ee) after flash chromatography (elution gradient: ethyl acetate:petroleum ether = 1:5); **HPLC analysis:** Daicel CHIRALPAK OJ-H, *n*-hexane:*i*-PrOH = 96:4, flow rate = 1.0 mL·min^{-1}, λ = 254 nm, retention time: t_R = 8.9 min (minor), t_R = 10.5 min (major).

***Tert*-butyl((3*S*,4*R*)-5-hydroxy-1-(4-methoxyphenyl)-4-methyl-3-(trifluoromethyl) pent-1-yn-3-yl)carbamate**

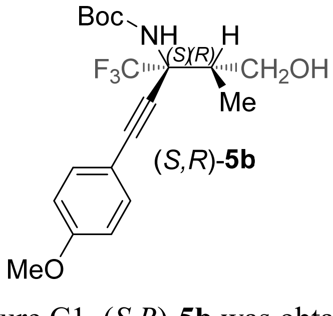

(*S,R*)-**5b**

Following the General Procedure C1, (*S,R*)-**5b** was obtained as white solid (24 h, 32.1 mg, 83% yield, 19:1 *anti/syn*, 96% ee) after flash chromatography (elution gradient: ethyl acetate:petroleum ether = 1:3); **$[\alpha]_D^{20}$** = −18.4 (*c* 1.0, $CHCl_3$); **^{1}H NMR** (600 MHz, $CDCl_3$, ppm): δ 7.40 (d, *J* = 9.0 Hz, 2H), 6.84 (d, *J* = 8.4 Hz, 2H), 5.26 (s, 1H), 3.94-3.88 (m, 2H), 3.82 (s, 3H), 2.76 (s, 1H), 2.30 (s, 1H), 1.46 (s, 9H), 1.23 (d, *J* = 6.6 Hz, 3H); **^{13}C NMR** (150 MHz, $CDCl_3$, ppm): δ 160.3, 153.5, 133.5, 125.6 (q, $^1J_{CF}$ = 286.5 Hz), 114.0, 113.4, 88.0, 80.9, 80.1, 64.4, 61.1 (q, $^2J_{CF}$ = 30.0 Hz) , 55.3, 40.0, 28.2, 12.4;

^{19}F NMR (565 MHz, $CDCl_3$, ppm): δ -72.5 (s); **HPLC analysis:** Daicel CHIRALPAK AD-H, *n*-hexane:*i*-PrOH = 95:5, flow rate = 1.0 mL·min^{-1}, λ = 254 nm, retention time: t_R = 13.8 min (minor), t_R = 17.4 min (major); **HRMS** (ESI) m/z calcd. for $C_{19}H_{24}F_3NNaO_4$ $[M + Na]^+$: 415.1550, found: 415.1547.

***Tert*-butyl((3*R*,4*S*)-5-hydroxy-1-(4-methoxyphenyl)-4-methyl-3-(trifluoromethyl) pent-1-yn-3-yl)carbamate**

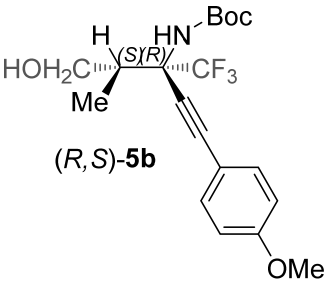

(*R,S*)-**5b**

Following the General Procedure C2, (*R,S*)-**5b** was obtained as white solid (24 h, 25.2 mg, 65% yield, 10:1 *anti/syn*, 94% ee) after flash chromatography (elution gradient: ethyl acetate:petroleum ether = 1:3); **HPLC analysis:** Daicel CHIRALPAK AD-H, *n*-hexane:*i*-PrOH = 95:5, flow rate = 1.0 mL·min^{-1}, λ = 254 nm, retention time: t_R = 13.8 min (major), t_R = 17.5 min (minor).

***Tert*-butyl((3*S*,4*R*)-5-hydroxy-4-methyl-1-(p-tolyl)-3-(trifluoromethyl)pent-1-yn-3-yl)carbamate**

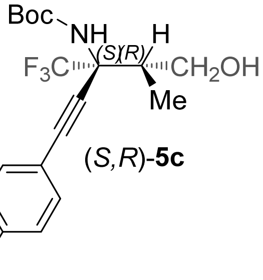

(*S,R*)-**5c**

Following the General Procedure C1, (*S,R*)-**5c** was obtained as white solid (20 h, 30.8 mg, 83% yield, 14:1 *anti/syn*, 96% ee) after flash chromatography (elution gradient: ethyl acetate:petroleum ether = 1:5); **$[\alpha]_D^{20}$** = −5.4 (*c* 1.0, $CHCl_3$); **^{1}H NMR** (600 MHz, $CDCl_3$, ppm): δ 7.36 (d, *J* = 7.8 Hz, 2H), 7.13 (d, *J* = 7.2 Hz, 2H), 5.30 (s, 1H), 3.94-3.87 (m, 2H), 2.76 (s, 1H), 2.35 (s, 3H), 1.46 (s, 9H), 1.23 (d, *J* = 6.6 Hz, 3H); **^{13}C NMR** (150 MHz, $CDCl_3$, ppm): δ 153.5, 139.4, 131.8, 129.1, 125.6 (q, $^1J_{CF}$ = 285.5 Hz), 118.3, 88.1, 80.9, 80.8, 64.4, 60.9 (q, $^2J_{CF}$ = 28.5 Hz) , 40.1, 28.2, 21.5, 12.4; **^{19}F**

NMR (565 MHz, $CDCl_3$, ppm): δ -72.4 (s); **HPLC analysis:** Daicel CHIRALPAK AD-H, *n*-hexane:*i*-PrOH = 90:10, flow rate = 1.0 mL·min^{-1}, λ = 254 nm, retention time: t_R = 6.6 min (minor), t_R = 8.2 min (major); **HRMS** (ESI) m/z calcd. for $C_{19}H_{24}F_3NNaO_3$ $[M + Na]^+$: 394.1600, found: 394.1601.

***Tert*-butyl((3*R*,4*S*)-5-hydroxy-4-methyl-1-(*p*-tolyl)-3-(trifluoromethyl)pent-1-yn-3-yl)carbamate**

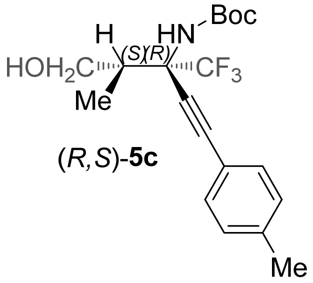

(*R,S*)-**5c**

Following the General Procedure C2, (*R,S*)-**5c** was obtained as white solid (20 h, 26.4 mg, 71% yield, 7:1 *anti/syn*, 95% ee) after flash chromatography (elution gradient: ethyl acetate:petroleum ether = 1:5); **HPLC analysis:** Daicel CHIRALPAK AD-H, *n*-hexane:*i*-PrOH = 90:10, flow rate = 1.0 mL·min^{-1}, λ = 254 nm, retention time: t_R = 6.5 min (major), t_R = 8.1 min (minor).

***Tert*-butyl ((3*S*,4*R*)-1-(4-chlorophenyl)-5-hydroxy-4-methyl-3-(trifluoromethyl) pent-1-yn-3-yl)carbamate**

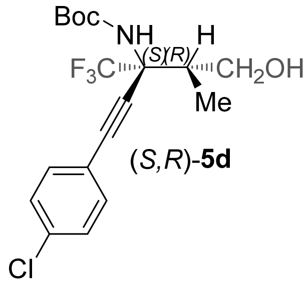

(*S,R*)-**5d**

Following the General Procedure C1, (*S,R*)-**5d** was obtained as white solid (20 h, 34.0 mg, 87% yield, 16:1 *anti/syn*, 97% ee) after flash chromatography (elution gradient: ethyl acetate:petroleum ether = 1:5); **$[\alpha]_D^{20}$** = −29.0 (*c* 1.0, $CHCl_3$); **^{1}H NMR** (600 MHz, $CDCl_3$, ppm): δ 7.40 (d, *J* = 8.4 Hz, 2H), 7.30 (d, *J* = 8.4 Hz, 2H), 5.37 (s, 1H), 3.93-3.88 (m, 2H), 2.68 (s, 1H), 2.46 (s, 1H), 1.46 (s, 9H), 1.22 (d, *J* = 6.6 Hz, 3H); **^{13}C NMR** (150 MHz, $CDCl_3$, ppm): δ 153.6, 135.3, 133.2, 128.7, 125.4 (q, $^1J_{CF}$ = 285 Hz),

120.0, 86.8, 82.5, 81.1, 64.4, 60.8 (q, $^2J_{CF}$ = 30 Hz) , 40.5, 28.2, 12.3; **^{19}F NMR** (565 MHz, $CDCl_3$, ppm): *δ* -72.6 (s); **HPLC analysis:** Daicel CHIRALPAK AD-H, *n*-hexane:*i*-PrOH = 90:10, flow rate = 1.0 mL·min^{-1}, λ = 254 nm, retention time: t_R = 6.3 min (minor), t_R = 7.8 min (major); **HRMS** (ESI) m/z calcd. for $C_{18}H_{21}ClF_3NNaO_3$ [M + Na]$^+$: 414.1054, found: 414.1053.

***Tert*-butyl ((3*R*,4*S*)-1-(4-chlorophenyl)-5-hydroxy-4-methyl-3-(trifluoromethyl) pent-1-yn-3-yl)carbamate**

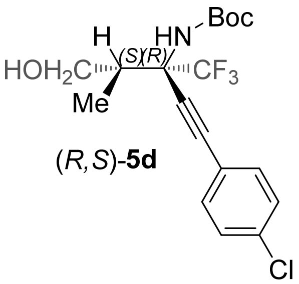

(*R*,*S*)-**5d**

Following the General Procedure C2, (*R,S*)-**5d** was obtained as white solid (20 h, 26.2 mg, 80% yield, 7:1 *anti/syn*, 94% ee) after flash chromatography (elution gradient: ethyl acetate:petroleum ether = 1:5); **HPLC analysis:** Daicel CHIRALPAK AD-H, *n*-hexane:*i*-PrOH = 90:10, flow rate = 1.0 mL·min^{-1}, λ = 254 nm, retention time: t_R = 6.2 min (major), t_R = 7.8 min (minor).

***Tert*-butyl ((3*S*,4*R*)-1-(3-fluorophenyl)-5-hydroxy-4-methyl-3-(trifluoromethyl)pent-1-yn-3-yl)carbamate**

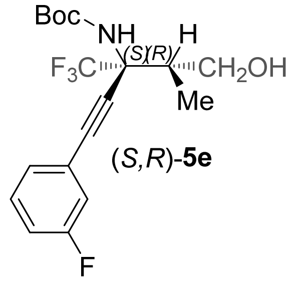

(*S*,*R*)-**5e**

Following the General Procedure C1, (*S,R*)-**5e** was obtained as white solid (20 h, 30.7 mg, 82% yield, 10:1 *anti/syn*, 94% ee) after flash chromatography (elution gradient: ethyl acetate:petroleum ether = 1:5); **$[\alpha]_D^{20}$** = −26.3 (*c* 1.0, $CHCl_3$); **^{1}H NMR** (600 MHz, $CDCl_3$, ppm): *δ* 7.31-7.25 (m, 2H), 7.18-7.16 (m, 1H), 7.09-7.05 (m, 1H), 5.43 (s, 1H), 3.93-3.87 (m, 2H), 2.67 (s, 1H), 2.46 (s, 1H), 1.46 (s, 9H), 1.22 (d, *J* = 7.2 Hz, 3H);

^{13}C NMR (150 MHz, $CDCl_3$, ppm): δ 162.3 (d, $^1J_{CF}$ = 246.7 Hz), 153.6, 135.3, 130.0, (d, $^3J_{CF}$ = 8.8 Hz), 127.8 (d, $^4J_{CF}$ = 3.3 Hz), 125.4 (q, $^1J_{CF}$ = 291 Hz), 123.3 (d, $^3J_{CF}$ = 8.9 Hz), 118.8 (d, $^2J_{CF}$ = 22.8 Hz), 116.6 (d, $^2J_{CF}$ = 21.1 Hz), 86.6, 82.5, 81.1, 64.3, 60.8 (q, $^2J_{CF}$ = 28.2 Hz) , 40.6, 28.2, 12.3; **^{19}F NMR** (565 MHz, $CDCl_3$, ppm): δ -72.6 (s, 3F), -112.6 (s, 1F); **HPLC analysis:** Daicel CHIRALPAK AD-H, *n*-hexane:*i*-PrOH = 90:10, flow rate = 1.0 mL·min^{-1}, λ = 254 nm, retention time: t_R = 5.4 min (minor), t_R =6.4 min (major); **HRMS** (ESI) m/z calcd. for $C_{18}H_{21}F_4NNaO_3$ [M + Na]$^+$: 398.1350, found: 398.1351.

***Tert*-butyl ((3*R*,4*S*)-1-(3-fluorophenyl)-5-hydroxy-4-methyl-3-(trifluoromethyl)pent-1-yn-3-yl)carbamate**

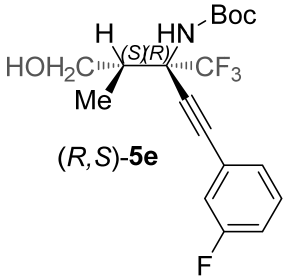

(*R,S*)-**5e**

Following the General Procedure C2, (*R,S*)-**5e** was obtained as white solid (20 h, 24.3 mg, 65% yield, 7:1 *anti/syn*, 95% ee) after flash chromatography (elution gradient: ethyl acetate:petroleum ether = 1:5); **HPLC analysis:** Daicel CHIRALPAK AD-H, *n*-hexane:*i*-PrOH = 90:10, flow rate = 1.0 mL·min^{-1}, λ = 254 nm, retention time: t_R =5.4 min (major), t_R = 6.5 min (minor).

***Tert*-butyl ((3*S*,4*R*)-1-(2-fluorophenyl)-5-hydroxy-4-methyl-3-(trifluoromethyl)pent-1-yn-3-yl)carbamate**

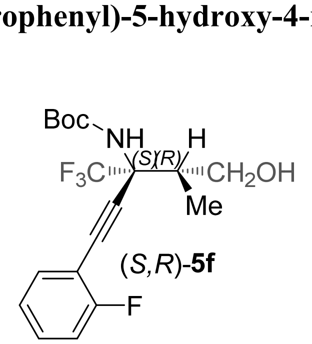

(*S,R*)-**5f**

Following the General Procedure C1, (*S,R*)-**5f** was obtained as white solid (20 h, 31.5 mg, 84% yield, 8:1 *anti/syn*, 96% ee) after flash chromatography (elution gradient: ethyl acetate:petroleum ether = 1:5); **[α]$_D$^{20}** = −28.8 (*c* 1.0, $CHCl_3$); **^{1}H NMR** (600 MHz,

$CDCl_3$, ppm): δ 7.47 (dt, J = 1.2, 7.2 Hz, 1H), 7.36-7.32 (m, 1H), 7.12-7.06 (m, 2H), 5.45 (s, 1H), 3.96-3.91 (m, 2H), 2.72 (s, 1H), 2.52 (s, 1H), 1.47 (s, 9H), 1.25 (d, J = 7.2 Hz, 3H); **^{13}C NMR** (150 MHz, $CDCl_3$, ppm): δ 163.1 (d, $^1J_{CF}$ = 254.4 Hz), 153.5, 133.7, 131.0 (d, $^3J_{CF}$ = 7.7 Hz), 125.4 (q, $^1J_{CF}$ = 292.5 Hz), 124.0 (d, $^4J_{CF}$ = 3.3 Hz), 115.6 (d, $^2J_{CF}$ = 19.9 Hz), 110.1 (d, $^2J_{CF}$ = 15.5 Hz), 86.8, 81.4, 81.1, 64.3, 61.0 (q, $^2J_{CF}$ = 30.2 Hz) , 40.4, 28.2, 12.3; **^{19}F NMR** (565 MHz, $CDCl_3$, ppm): δ -72.2 (s, 3F), -109.4 (s, 1F); **HPLC analysis:** Daicel CHIRALPAK IA, *n*-hexane:*i*-PrOH = 85:15, flow rate = 1.0 mL·min^{-1}, λ = 254 nm, retention time: t_R = 5.0 min (minor), t_R = 6.0 min (major); **HRMS** (ESI) m/z calcd. for $C_{18}H_{21}F_4NNaO_3$ $[M + Na]^+$: 398.1350, found: 398.1351.

***Tert*-butyl ((3*R*,4*S*)-1-(2-fluorophenyl)-5-hydroxy-4-methyl-3-(trifluoromethyl)pent-1-yn-3-yl)carbamate**

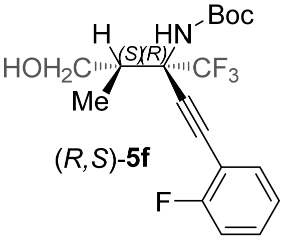

(*R,S*)-**5f**

Following the General Procedure C2, (*R,S*)-**5f** was obtained as white solid (20 h, 25.5 mg, 68% yield, 7:1 *anti/syn*, 94% ee) after flash chromatography (elution gradient: ethyl acetate:petroleum ether = 1:5); **HPLC analysis:** Daicel CHIRALPAK IA, *n*-hexane:*i*-PrOH = 85:15, flow rate = 1.0 mL·min^{-1}, λ = 254 nm, retention time: t_R = 5.2 min (major), t_R = 6.2 min (minor).

***Tert*-butyl((3*S*,4*R*)-1-(cyclohex-1-en-1-yl)-5-hydroxy-4-methyl-3-(trifluoromethyl)pent-1-yn-3-yl)carbamate**

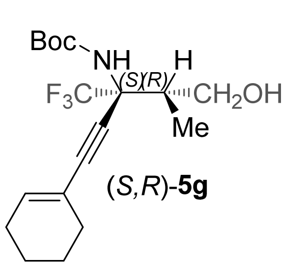

(*S,R*)-**5g**

Following the General Procedure C1, (*S,R*)-**5g** was obtained as white oil liquid (24 h, 29.6 mg, 82% yield, 13:1 *anti/syn*, 96% ee) after flash chromatography (elution gradient:

ethyl acetate:petroleum ether = 1:5); **[α]$_D^{20}$** = −14.8 (*c* 1.0, $CHCl_3$); **^{1}H NMR** (600 MHz, $CDCl_3$, ppm): δ 6.20-6.19 (m, 1H), 5.16 (s, 1H), 3.83 (d, J = 4.8 Hz, 2H), 2.72 (s, 1H), 2.12-2.09 (m, 4H), 1.65-1.61 (m, 2H), 1.60-1.57 (m, 2H), 1.45 (s, 9H), 1.18 (d, 3H, J = 7.2 Hz); **^{13}C NMR** (150 MHz, $CDCl_3$, ppm): δ 153.4, 137.2, 125.5 (q, $^1J_{CF}$ = 291 Hz), 119.3, 89.8, 80.8, 78.6, 64.3, 60.9 (q, $^2J_{CF}$ = 29.1 Hz) , 39.7, 28.6, 28.2, 25.6, 22.0, 21.3, 12.3; **^{19}F NMR** (565 MHz, $CDCl_3$, ppm): δ -72.7 (s); **HPLC analysis:** Daicel CHIRALPAK AD-H, *n*-hexane:*i*-PrOH = 95:5, flow rate = 1.0 mL·min^{-1}, λ = 220 nm, retention time: t_R = 8.6 min (minor), t_R = 11.2 min (major); **HRMS** (ESI) m/z calcd. for $C_{18}H_{26}F_3NNaO_3$ $[M + Na]^+$: 384.1757, found: 384.1761.

***Tert*-butyl((3*R*,4*S*)-1-(cyclohex-1-en-1-yl)-5-hydroxy-4-methyl-3-(trifluoromethyl) pent-1-yn-3-yl)carbamate**

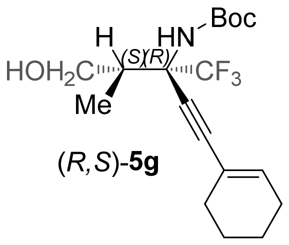

(*R,S*)-**5g**

Following the General Procedure C2, (*R,S*)-**5g** was obtained as white oil liquid (24 h, 22.7 mg, 63% yield, 8:1 *anti/syn*, 95% ee) after flash chromatography (elution gradient: ethyl acetate/petroleum ether = 1/5); **HPLC analysis:** Daicel CHIRALPAK AD-H, *n*-hexane:*i*-PrOH = 95:5, flow rate = 1.0 mL·min^{-1}, λ = 220 nm, retention time: t_R = 8.7 min (major), t_R = 12.4 min (minor).

***Tert*-butyl((3*S*,4*R*)-5-hydroxy-4-methyl-1-(thiophen-2-yl)-3-(trifluoromethyl) pent-1-yn-3-yl)carbamate**

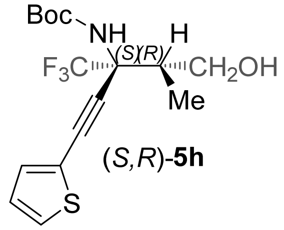

(*S,R*)-**5h**

Following the General Procedure C1, (*S,R*)-**5h** was obtained as white solid (20 h, 28.3 mg, 78% yield, 13:1 *anti/syn*, 94% ee) after flash chromatography (elution gradient: ethyl acetate:petroleum ether = 1:5); **[α]$_D^{20}$** = −24.3 (*c* 1.0, $CHCl_3$); **^{1}H NMR** (600 MHz,

$CDCl_3$, ppm): δ 7.31-7.27 (m, 2H), 6.98 (dd, J = 3.6, 4.8 Hz, 1H), 5.54 (s, 1H), 3.90 (d, J = 5.4 Hz, 2H), 2.67 (s, 1H), 1.47 (s, 9H), 1.22 (d, J = 7.2 Hz, 3H); **^{13}C NMR** (150 MHz, $CDCl_3$, ppm): δ 153.7, 133.2, 128.1, 127.0, 125.4 (q, $^1J_{CF}$ = 286.2 Hz), 121.2, 85.5, 81.1, 79.1, 64.3, 61.1 (q, $^2J_{CF}$ = 29.7 Hz) , 40.5, 28.2, 12.3; **^{19}F NMR** (565 MHz, $CDCl_3$, ppm): δ -72.1 (s, 3F, major diastereoisomer), -72.9 (s, 0.22F, minor diastereoisomer); **HPLC analysis:** Daicel CHIRALPAK IA, *n*-hexane:*i*-PrOH = 90:10, flow rate = 1.0 mL·min^{-1}, λ = 254 nm, retention time: t_R = 7.3 min (minor), t_R = 9.3 min (major); **HRMS** (ESI) m/z calcd.for $C_{16}H_{20}F_3NNaO_3S$ [M + Na]$^+$: 386.1008, found: 386.1010.

***Tert*-butyl((3*R*,4*S*)-5-hydroxy-4-methyl-1-(thiophen-2-yl)-3-(trifluoromethyl) pent-1-yn-3-yl)carbamate**

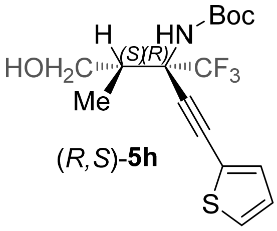

(*R*,*S*)-**5h**

Following the General Procedure C2, (*R,S*)-**5h** was obtained as white solid (20 h, 23.2 mg, 64% yield, 6:1 *anti/syn*, 92% ee) after flash chromatography (elution gradient: ethyl acetate:petroleum ether = 1:5); **HPLC analysis:** Daicel CHIRALPAK IA, *n*-hexane:*i*-PrOH = 90:10, flow rate = 1.0 mL·min^{-1}, λ = 254 nm, retention time: t_R = 7.2 min (major), t_R = 9.2 min (minor).

***Tert*-butyl((3*S*,4*R*)-4-(hydroxymethyl)-1-(4-methoxyphenyl)-3-(trifluoromethyl) hex-1-yn-3-yl)carbamate**

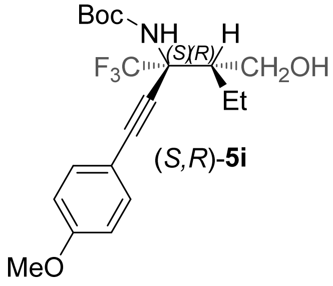

(*S*,*R*)-**5i**

Following the General Procedure C1, (*S,R*)-**5i** was obtained as white solid (24 h, 29.3

mg, 73% yield, 8:1 *anti/syn*, 96% ee) after flash chromatography (elution gradient: ethyl acetate:petroleum ether = 1:5); **$[\alpha]_D^{20}$** = −17.8 (*c* 1.0, $CHCl_3$); **^{1}H NMR** (600 MHz, $CDCl_3$, ppm): δ 7.40 (d, J = 9.0 Hz, 2H), 6.84 (d, J = 9.0 Hz, 2H), 5.37 (s, 1H), 4.16-4.13 (m, 1H), 3.94-3.90 (m, 1H), 3.81 (s, 3H), 2.56 (s, 1H), 2.37 (s, 1H), 1.76-1.72 (m, 1H), 1.69-1.66 (m, 1H), 1.46 (s, 9H), 1.06 (t, J = 7.8 Hz, 3H); **^{13}C NMR** (150 MHz, $CDCl_3$, ppm): δ 160.3, 153.7, 133.5, 125.6 (q, $^1J_{CF}$ = 285.2 Hz), 114.0, 113.4, 88.0, 81.0, 80.7, 61.1 (q, $^2J_{CF}$ = 28.3 Hz) , 60.8, 55.3, 46.7, 28.2, 18.9, 12.3; **^{19}F NMR** (565 MHz, $CDCl_3$, ppm): δ -72.6 (s); **HPLC analysis:** Daicel CHIRALPAK AD-H, *n*-hexane:*i*-PrOH = 95:5, flow rate = 1.0 mL·min^{-1}, λ = 254 nm, retention time: t_R =16.2 min (major), t_R = 17.5 min (minor); **HRMS** (ESI) m/z calcd. for $C_{20}H_{26}F_3NNaO_4$ $[M + Na]^+$: 424.1706, found: 424.1704.

***Tert*-butyl((3*R*,4*S*)-4-(hydroxymethyl)-1-(4-methoxyphenyl)-3-(trifluoromethyl) hex-1-yn-3-yl)carbamate**

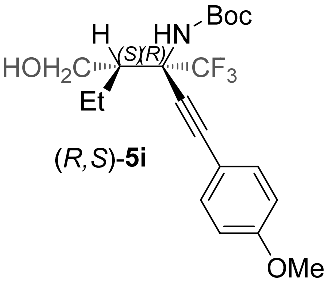

(*R,S*)-**5i**

Following the General Procedure C2, (*R,S*)-**5i** was obtained as white solid (24 h, 25.3 mg, 63% yield, 5:1 *anti/syn*, 94% ee) after flash chromatography (elution gradient: ethyl acetate:petroleum ether = 1:5); **HPLC analysis:** Daicel CHIRALPAK AD-H, *n*-hexane:*i*-PrOH = 95:5, flow rate = 1.0 mL·min^{-1}, λ = 254 nm, retention time: t_R = 16.3 min (minor), t_R = 17.6 min (major).

***Tert*-butyl((3*S*,4*R*)-4-benzyl-5-hydroxy-1-(4-methoxyphenyl)-3-(trifluoromethyl) pent-1-yn-3-yl)carbamate**

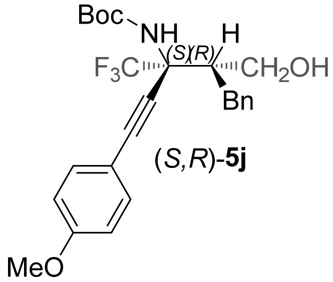

(*S,R*)-**5j**

Following the General Procedure C1, (*S,R*)-**5j** was obtained as white solid (20 h, 36.1 mg, 78% yield, 10:1 *anti/syn*, 97% ee) after flash chromatography (elution gradient: ethyl acetate:petroleum ether = 1:5); **$[\alpha]_D^{20}$** = −8.6 (*c* 1.0, $CHCl_3$); **^{1}H NMR** (600 MHz, $CDCl_3$, ppm): δ 7.43 (d, J = 9.0 Hz, 2H), 7.30 (d, J = 4.8 Hz, 4H), 7.24-7.20 (m, 1H), 6.85 (d, J = 8.4 Hz, 2H), 5.56 (s, 1H), 4.03 (d, J = 12.6 Hz, 1H), 3.82 (s, 3H), 3.69-3.65 (m, 1H), 3.12-3.08 (m, 1H), 2.90-2.87 (m, 2H), 1.49 (s, 9H); **^{13}C NMR** (150 MHz, $CDCl_3$, ppm): δ 160.4, 153.5, 139.6, 133.5, 129.4, 128.6, 126.4, 125.7 (q, $^1J_{CF}$ = 292.5 Hz), 114.1, 113.2, 88.5, 81.1, 80.6, 61.2 (q, $^2J_{CF}$ = 30.0 Hz) , 60.4, 55.3, 46.7, 32.3, 28.3; **^{19}F NMR** (565 MHz, $CDCl_3$, ppm): δ -72.0 (s); **HPLC analysis:** Daicel CHIRALPAK IA, *n*-hexane:*i*-PrOH = 95:5, flow rate = 1.0 mL·min^{-1}, λ = 254 nm, retention time: t_R = 11.3 min (major), t_R = 16.2 min (minor); **HRMS** (ESI) m/z calcd. for $C_{25}H_{28}F_3NNaO_4$ $[M + Na]^+$: 386.1863, found: 386.1865.

***Tert*-butyl((3*R*,4*S*)-4-benzyl-5-hydroxy-1-(4-methoxyphenyl)-3-(trifluoromethyl) pent-1-yn-3-yl)carbamate**

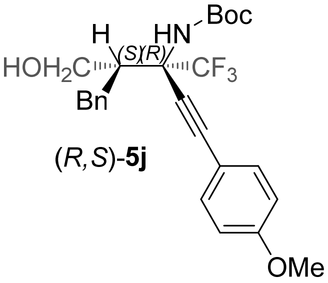

(*R,S*)-**5j**

Following the General Procedure C2, (*R,S*)-**5j** was obtained as white solid (20 h, 30.5

mg, 66% yield, 9:1 *anti/syn*, 93% ee) after flash chromatography (elution gradient: ethyl acetate:petroleum ether = 1:5); **HPLC analysis:** Daicel CHIRALPAK IA, *n*-hexane:*i*-PrOH = 95:5, flow rate = 1.0 mL·min^{-1}, λ = 254 nm, retention time: t_R = 11.4 min (minor), t_R = 16.2 min (major).

***Tert*-butyl((3*R*,4*R*)-5-hydroxy-4-methyl-1-phenylpent-1-yn-3-yl)carbamate**

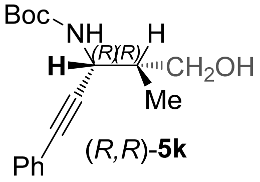

(*R,R*)-**5k**

Following the General Procedure C1 without 4-nitrobenzoic acid, (*R,R*)-**5k** was obtained as yellow oil (14 h, 23.7 mg, 82% yield, 16:1 *anti/syn*, 94% ee) after flash chromatography (elution gradient: ethyl acetate:petroleum ether = 1:5); **[α]$_D^{20}$** = +154.7 (*c* 1.0, CH_2Cl_2); **^{1}H NMR** (600 MHz, $CDCl_3$, ppm): *δ* 7.37-7.35 (m, 2H), 7.28-7.26 (m, 3H), 7.22 (t, *J* = 8.0 Hz, 2H), 6.80-6.78 (m, 2H), 4.46 (d, *J* = 4.0 Hz, 1H), 3.95 (dd, *J* = 10.8, 8.0 Hz, 1H), 3.79 (dd, *J* = 10.8, 4.0 Hz, 1H), 2.29-2.61 (m, 1H), 1.14 (d, *J* = 7.2 Hz, 3H); **^{13}C NMR** (150 MHz, $CDCl_3$, ppm): *δ* 147.0, 131.9, 129.3, 128.4, 128.3, 123.0, 118.9, 114.6, 88.2, 84.6, 66.1 , 50.8, 39.8, 13.9; **HPLC analysis:** Daicel CHIRALPAK IB-3, *n*-hexane:*i*-PrOH = 93:7, flow rate = 1.0 mL·min^{-1}, λ = 254 nm, retention time: t_R = 13.7 min (major), t_R = 14.3 min (minor).

***Tert*-butyl((3*S*,4*S*)-5-hydroxy-4-methyl-1-phenylpent-1-yn-3-yl)carbamate**

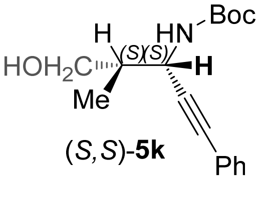

(*S,S*)-**5k**

Following the General Procedure C2 without 4-nitrobenzoic acid, (*S,S*)-**5k** was obtained as yellow oil (18 h, 21.7mg, 75% yield, 10:1 *anti/syn*, 87% ee) after flash chromatography (elution gradient: ethyl acetate:petroleum ether = 1:5); **[α]$_D^{20}$** = -121.3 (*c* 1.0, CH_2Cl_2); **HPLC analysis:** Daicel CHIRALPAK IB-3, *n*-hexane:*i*-PrOH = 93:7,

flow rate = 1.0 mL·min^{-1}, λ = 254 nm, retention time: t_R = 13.7 min (minor), t_R = 14.2 min (major).

Supplementary Note 6

General Procedure for Asymmetric Catalyzed Mannich Reactions of Isatin Ketimines and *α*-Substituted Aldehydes

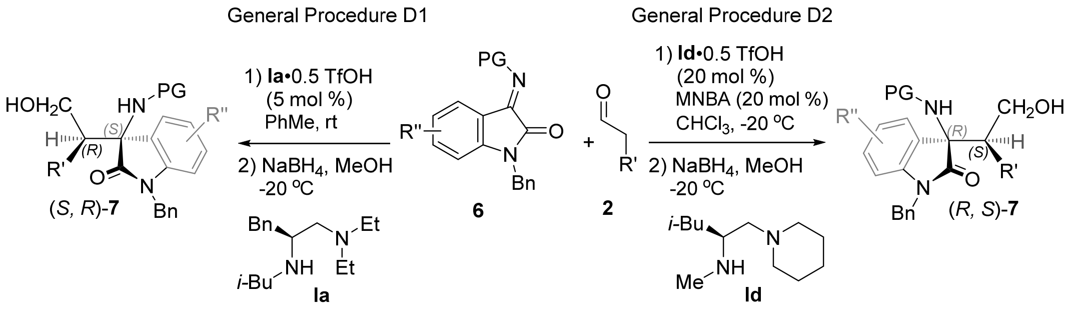

General Procedure D1: Mannich reaction catalyzed by *N*-^{i}Bu amine Ia. To a solution of isatin ketimines **6** (0.1 mmol), and catalyst **Ia**·0.5TfOH (1.7 mg, 5 mol%) in anhydrous toluene (0.8 mL) was added *α*-substituted aldehydes **2** (0.2 mmol) at room temperature. After the reaction mixture was stirred for the shown time, $NaBH_4$ (15.2 mg, 0.4 mmol) and MeOH (0.4 mL) were added at -20 °C, and the resulting mixture was stirred for 0.5 h. Finally, the resulting mixture was purified by silica gel column chromatography (ethyl acetate:petroleum ether = 1:10 to 1:4) to afford Mannich products (*S*, *R*)-**7**.

General Procedure D2: Mannich reaction catalyzed by *N*-Me amine Id. To a solution of isatin ketimines **6** (0.1 mmol), 3-nitrobenzoic acid (3.3 mg, 0.02 mmol), and catalyst **Id**·0.5TfOH (5.6 mg, 20 mol%) in anhydrous $CHCl_3$ (0.8 mL) was added *α*-substituted aldehydes **2** (0.3 mmol) at -20 °C. After the reaction mixture was stirred for the shown time, $NaBH_4$ (19.2 mg, 0.5 mmol) and MeOH (0.4 mL) were added at -20 °C, and the resulting mixture was stirred for 0.5 h. Finally, the resulting mixture was purified by silica gel column chromatography (ethyl acetate:petroleum ether = 1:10 to 1:4) to afford Mannich products (*R*, *S*)-**7**.

***Tert*-butyl((*S*)-1-benzyl-3-((*R*)-1-hydroxypropan-2-yl)-2-oxoindolin-3-yl) carbamate**

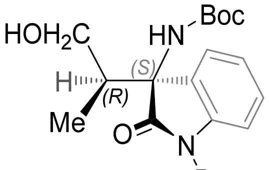

(*S,R*)-**7a**

Following the General Procedure D1, (*S,R*)-**7a** was obtained as white solid (8 h, 34.9 mg, 89% yield, >20:1 *anti/syn*, 94% ee) after flash chromatography (elution gradient: ethyl acetate:petroleum ether = 1:5); **[α]$_D$^{20}** = −18.6 (*c* 1.0, $CHCl_3$); **^{1}H NMR** (400 MHz, $CDCl_3$, ppm): *δ* (d, *J* = 7.2 Hz, 2H), 7.32 (t, *J* = 7.2 Hz, 2H), 7.27-7.24 (m, 2H), 7.18 (t, *J* = 7.6 Hz, 1H), 7.04 (t, *J* = 7.6 Hz, 1H), 6.84 (s, 1H), 6.72 (d, *J* = 8.0 Hz, 1H), 5.12 (br, 1H), 4.78 (br, 1H), 4.18-4.11 (m, 1H), 3.75-3.72 (m, 1H), 3.17 (br, 1H), 2.31-2.28 (m, 1H), 1.26 (s, 9H), 0.61 (d, *J* = 7.2 Hz, 3H); **^{13}C NMR** (100 MHz, $CDCl_3$, ppm): *δ* 177.0, 154.1, 142.9, 135.9, 128.7, 128.5, 127.6, 123.0, 122.2, 108.9, 80.0, 64.7, 63.9, 44.3, 41.7, 28.1, 11.6; **HPLC analysis:** Daicel CHIRALPAK AD-H, *n*-hexane:*i*-PrOH = 75:25, flow rate = 0.8 mL·min^{-1}, λ = 254 nm, retention time: t_R = 9.1 min (minor), t_R = 14.3 min (major).

***Tert*-butyl((*R*)-1-benzyl-3-((*S*)-1-hydroxypropan-2-yl)-2-oxoindolin-3-yl) carbamate**

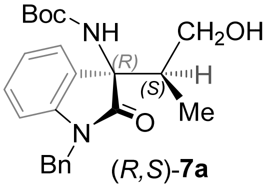

(*R,S*)-**7a**

Following the General Procedure D2, toluene as the solvent was employed and (*R,S*)-**7a** was obtained as white solid (12 h, 37.2 mg, 94% yield, >20:1 *anti/syn*, 91% ee) after flash chromatography (elution gradient: ethyl acetate:petroleum ether = 1:5);

HPLC analysis: Daicel CHIRALPAK AD-H, *n*-hexane:*i*-PrOH = 75:25, flow rate = 0.8 mL·min^{-1}, λ = 254 nm, retention time: t_R = 9.1 min (major), t_R = 14.9 min (minor).

***Tert*-butyl((*S*)-1-benzyl-3-((*R*)-1-hydroxybutan-2-yl)-2-oxoindolin-3-yl) carbamate**

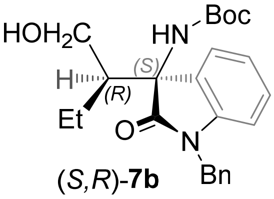

(*S,R*)-**7b**

Following the General Procedure D1, (*S,R*)-**7b** was obtained as white solid (4 h, 36.9 mg, 90% yield, >20:1 *anti/syn*, 96% ee) after flash chromatography (elution gradient: ethyl acetate:petroleum ether = 1:8); **[α]$_D$^{20}** = −13.6 (*c* 0.8, $CHCl_3$); **^{1}H NMR** (400 MHz, $CDCl_3$, ppm): δ 7.39-7.37 (m, 2H), 7.32-7.24 (m, 4H), 7.18 (t, *J* = 7.6 Hz, 1H), 7.05 (t, *J* = 7.6 Hz, 1H), 6.71 (d, *J* = 7.6 Hz, 1H), 6.59 (br, 1H), 5.12 (d, *J* = 12.8 Hz, 1H), 4.74 (br, 1H), 4.20-4.14 (m, 1H), 3.95 (d, *J* = 12 Hz, 1H), 3.05 (d, *J* = 3.6 Hz, 1H), 1.95 (s, 1H), 1.26 (s, 9H), 1.14-1.04 (m, 1H), 0.93-0.87 (m, 1H), 0.77 (t, *J* = 7.2 Hz, 3H); **^{13}C NMR** (100 MHz, $CDCl_3$, ppm): δ 177.3, 154.1, 143.1, 135.8, 131.4, 128.7, 128.6, 127.6, 127.5, 123.1, 122.5, 108.9, 80.1, 64.6, 60.4, 48.5, 44.3, 28.1, 17.8, 12.5; **HPLC analysis:** Daicel CHIRALPAK AD-H, *n*-hexane:*i*-PrOH = 80:20, flow rate = 0.8 mL·min^{-1}, λ = 254 nm, retention time: t_R = 8.6 min (minor), t_R = 13.2 min (major).

***Tert*-butyl((*R*)-1-benzyl-3-((*S*)-1-hydroxybutan-2-yl)-2-oxoindolin-3-yl) carbamate**

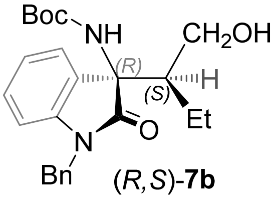

(*R,S*)-**7b**

Following the General Procedure D2, (*R,S*)-**7b** was obtained as white solid (14 h, 37.1 mg, 91% yield, 9:1 *anti/syn*, 91% ee) after flash chromatography (elution gradient: ethyl acetate:petroleum ether = 1:8); **HPLC analysis:** Daicel CHIRALPAK AD-H, *n*-hexane:*i*-PrOH = 80:20, flow rate = 0.8 mL·min^{-1}, λ = 254 nm, retention time: t_R = 8.8 min (major), t_R = 14.3 min (minor).

***Tert*-butyl((*S*)-1-benzyl-3-((*R*)-1-hydroxybutan-2-yl)-5-methoxy-2-oxoindolin-3-yl)carbamate**

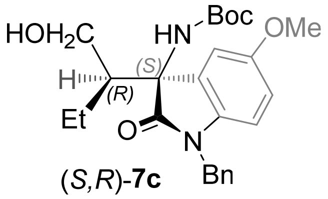

Following the General Procedure D1, (*S,R*)-**7c** was obtained as white solid (30 h, 37.8 mg, 86% yield, >20:1 *anti/syn*, 95% ee) after flash chromatography (elution gradient: ethyl acetate:petroleum ether = 1:6); **[α]$_D^{20}$** = −42.8 (*c* 0.5, $CHCl_3$); **^{1}H NMR** (400 MHz, $CDCl_3$, ppm): *δ* 7.37 (d, *J* = 7.6 Hz, 2H), 7.32 (t, *J* = 7.6 Hz, 2H), 7.27-7.24 (m, 1H), 6.91 (s, 1H), 6.69 (d, *J* = 8.4 Hz, 1H), 6.60-6.56 (m, 2H), 5.05 (brs, 1H), 4.76 (brs, 1H), 4.20-4.13 (m, 1H), 3.96 (d, *J* = 11.6 Hz, 1H), 3.76 (s, 3H), 3.12 (d, *J* = 6.8 Hz, 1H), 1.94 (s, 1H), 1.29 (s, 9H), 1.14-1.06 (m, 1H), 0.92-0.86 (m, 1H), 0.79 (t, *J* = 7.2 Hz, 3H); **^{13}C NMR** (100 MHz, $CDCl_3$, ppm): *δ* 177.0, 156.4, 154.1, 136.5, 135.8, 132.8, 128.7, 127.6, 127.5, 112.7, 110.0, 109.3, 80.1, 65.0, 60.3, 55.7, 48.5, 44.4, 28.2, 17.8, 12.4; **HPLC analysis:** Daicel CHIRALPAK AD-H, *n*-hexane:*i*-PrOH = 75:25, flow rate = 0.8 mL·min^{-1}, λ = 254 nm, retention time: t_R = 10.0 min (minor), t_R = 17.1 min (major).

***Tert*-butyl((*R*)-1-benzyl-3-((*S*)-1-hydroxybutan-2-yl)-5-methoxy-2-oxoindolin-3-yl)carbamate**

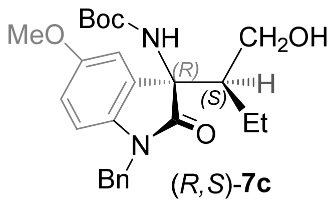

Following the General Procedure D2, (*R,S*)-**7c** was obtained as white solid (18 h, 40.8 mg, 93% yield, 10:1 *anti/syn*, 90% ee) after flash chromatography (elution gradient: ethyl acetate:petroleum ether = 1:6); **HPLC analysis:** Daicel CHIRALPAK AD-H, *n*-hexane:*i*-PrOH = 75:25, flow rate = 0.8 mL·min^{-1}, λ = 254 nm, retention time: t_R = 11.2 min (major), t_R = 16.5 min (minor).

***Tert*-butyl((*S*)-1-benzyl-6-bromo-3-((*R*)-1-hydroxybutan-2-yl)-2-oxoindolin-3-yl) carbamate**

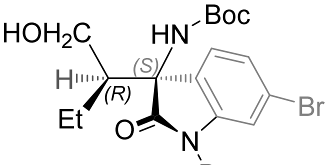

(*S*,*R*)-**7d**

Following the General Procedure D1, (*S,R*)-**7d** was obtained as white solid (7 h, 42.9 mg, 88% yield, >20:1 *anti/syn*, >99% ee) after flash chromatography (elution gradient: ethyl acetate:petroleum ether = 1:8); **$[\alpha]_D^{20}$** = +25.5 (*c* 0.8, $CHCl_3$); **^{1}H NMR** (400 MHz, $CDCl_3$, ppm): δ 7.37-7.32 (m, 4H), 7.30-7.27 (m, 1H), 7.18 (d, J = 7.6 Hz, 1H), 7.13 (d, J = 8 Hz, 1H), 6.84 (s, 1H), 6.76 (s, 1H), 5.04 (br, 1H), 4.75 (br, 1H), 4.24-4.17 (m, 1H), 3.89 (d, J = 11.6 Hz, 1H), 2.80 (s, 1H), 1.90 (s, 1H), 1.30 (s, 9H), 1.07-0.99 (m, 1H), 0.93-0.88 (m, 1H), 0.78 (t, J = 7.2 Hz, 3H); **^{13}C NMR** (100 MHz, $CDCl_3$, ppm): δ 177.1, 154.0, 144.5, 135.3, 130.5, 128.8, 127.8, 127.5, 125.9, 123.7, 122.1, 112.2, 80.3, 64.2, 60.4, 48.5, 44.4, 28.2, 17.9, 12.5; **HPLC analysis:** Daicel CHIRALPAK AD-H, *n*-hexane:*i*-PrOH = 95:5, flow rate = 0.6 mL·min^{-1}, λ = 254 nm, retention time: t_R = 33.3 min (minor), t_R = 58.5 min (major).

***Tert*-butyl((*R*)-1-benzyl-6-bromo-3-((*S*)-1-hydroxybutan-2-yl)-2-oxoindolin-3-yl) carbamate**

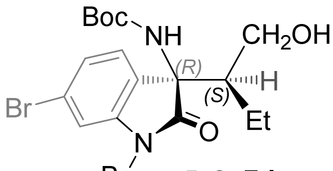

(*R*,*S*)-**7d**

Following the General Procedure D2, (*R,S*)-**7d** was obtained as white solid (18 h, 44.8 mg, 92% yield, >20:1 *anti/syn*, 98% ee) after flash chromatography (elution gradient: ethyl acetate:petroleum ether = 1:8); **HPLC analysis:** Daicel CHIRALPAK AD-H, *n*-hexane:*i*-PrOH = 95:5, flow rate = 0.6 mL·min^{-1}, λ = 254 nm, retention time: t_R = 35.1 min (major), t_R = 63.8 min (minor).

***Tert*-butyl((*S*)-1-benzyl-7-chloro-3-((*R*)-1-hydroxybutan-2-yl)-2-oxoindolin-3-yl) carbamate**

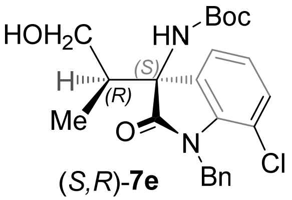

(*S,R*)-**7e**

Following the General Procedure D1, (*S,R*)-**7e** was obtained as white solid (32 h, 37.3 mg, 84% yield, 14:1 *anti/syn*, 93% ee) after flash chromatography (elution gradient: ethyl acetate:petroleum ether = 1:8); **[α]$_D$^{20}** = −13.9 (*c* 1.0, $CHCl_3$); **^{1}H NMR** (400 MHz, $CDCl_3$, ppm): δ 7.37 (d, J = 7.6 Hz, 2H), 7.30 (t, J = 7.2 Hz, 2H), 7.25-7.22 (m, 1H), 7.18 (d, J = 7.6 Hz, 2H), 7.00 (t, J = 7.6 Hz, 1H), 6.87 (s, 1H), 5.38 (d, J = 16 Hz, 1H), 5.29 (d, J = 16 Hz, 1H), 4.23-4.17 (m, 1H), 3.86 (d, J = 15.2 Hz, 1H), 2.85 (s, 1H), 1.87 (s, 1H), 1.30 (s, 9H), 0.99-0.87 (m, 2H), 0.76 (t, J = 7.2 Hz, 3H); **^{13}C NMR** (100 MHz, $CDCl_3$, ppm): δ 177.8, 154.0, 139.2, 137.6, 131.2, 128.4, 127.0, 123.9, 120.8, 115.1, 80.3, 64.0, 60.4, 49.0, 45.4, 28.2, 17.9, 12.5; **HPLC analysis:** Daicel CHIRALPAK AD-H, *n*-hexane:*i*-PrOH = 80:20, flow rate = 0.8 mL·min^{-1}, λ = 254 nm, retention time: t_R = 6.9 min (minor), t_R = 13.1 min (major).

***Tert*-butyl((*R*)-1-benzyl-7-chloro-3-((*S*)-1-hydroxybutan-2-yl)-2-oxoindolin-3-yl) carbamate**

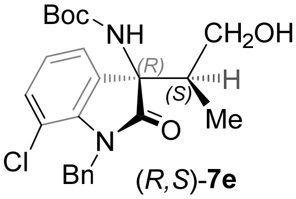

(*R,S*)-**7e**

Following the General Procedure D2, (*R,S*)-**7e** was obtained as white solid (18 h, 40.8 mg, 92% yield, 9:1 *anti/syn*, 92% ee) after flash chromatography (elution gradient: ethyl acetate:petroleum ether = 1:8); **HPLC analysis:** Daicel CHIRALPAK AD-H, *n*-hexane:*i*-PrOH = 80:20, flow rate = 0.8 mL·min^{-1}, λ = 254 nm, retention time: t_R = 7.8 min (major), t_R = 15.5 min (minor).

***Tert*-butyl((*S*)-1-benzyl-3-((*R*)-1-hydroxy-3-phenylpropan-2-yl)-2-oxoindolin-3-yl)carbamate**

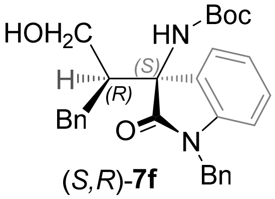

(*S,R*)-**7f**

Following the General Procedure D1, (*S,R*)-**7f** was obtained as white solid (6 h, 42 mg, 89% yield, >20:1 *anti/syn*, 93% ee) after flash chromatography (elution gradient: ethyl acetate:petroleum ether = 1:8); **$[\alpha]_D^{20}$** = −30.9 (*c* 1.0, $CHCl_3$); **^{1}H NMR** (400 MHz, $CDCl_3$, ppm): δ 7.41 (d, J = 7.2 Hz, 3H), 7.32 (t, J = 7.6 Hz, 2H), 7.27-7.09 (m, 6H), 6.93 (d, J = 7.6 Hz, 2H), 6.76 (d, J = 7.6 Hz, 1H), 5.16 (br, 1H), 4.79 (br, 1H), 4.10-4.04 (m, 1H), 3.81 (d, J = 11.6 Hz, 1H), 3.05 (s, 1H), 2.31-2.25 (m, 3H), 1.27 (s, 9H); **^{13}C NMR** (100 MHz, $CDCl_3$, ppm): δ 177.0, 154.2, 143.1, 139.4, 135.9, 128.9, 128.8, 128.4, 127.7, 127.6, 126.3, 123.3, 122.5, 109.1, 80.2, 64.5, 59.8, 48.8, 44.4, 30.9, 28.2; **HPLC analysis:** Daicel CHIRALPAK AD-H, *n*-hexane:*i*-PrOH = 75:25, flow rate = 0.9 mL·min^{-1}, λ = 254 nm, retention time: t_R = 9.0 min (minor), t_R = 17.8 min (major).

***Tert*-butyl((*R*)-1-benzyl-3-((*S*)-1-hydroxy-3-phenylpropan-2-yl)-2-oxoindolin-3-yl)carbamate**

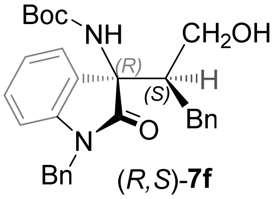

(*R,S*)-**7f**

Following the General Procedure D2, (*R,S*)-**7f** was obtained as white solid (14 h, 43.8 mg, 92% yield, 19:1 *anti/syn*, 90% ee) after flash chromatography (elution gradient: ethyl acetate:petroleum ether = 1:8); **HPLC analysis:** Daicel CHIRALPAK AD-H, *n*-hexane:*i*-PrOH = 75:25, flow rate = 0.9 mL·min^{-1}, λ = 254 nm, retention time: t_R = 10.0 min (major), t_R = 20.9 min (minor).

***Tert*-butyl((*S*)-1-benzyl-3-((*R*)-1-hydroxypent-4-en-2-yl)-2-oxoindolin-3-yl) carbamate**

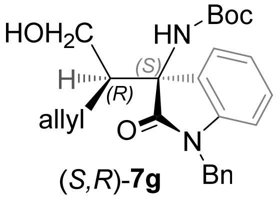

(*S,R*)-**7g**

Following the General Procedure D1, (*S,R*)-**7g** was obtained as white solid (10 h, 35.1 mg, 83% yield, 16:1 *anti/syn*, 94% ee) after flash chromatography (elution gradient: ethyl acetate:petroleum ether = 1:6); **$[\alpha]_D^{20}$** = −18.3 (*c* 1.0, $CHCl_3$); **^{1}H NMR** (400 MHz, $CDCl_3$, ppm): δ 7.39 (d, J = 7.2 Hz, 2H), 7.34-7.24 (m, 4H), 7.19 (t, J = 7.6 Hz, 1H), 7.05 (t, J = 7.6 Hz, 1H), 6.78 (s, 1H), 6.73 (d, J = 7.6 Hz, 1H), 5.57-5.47 (m, 1H), 5.08 (s, 1H), 4.94-4.78(m, 3H), 4.19-4.13 (m, 1H), 3.88 (d, J = 11.6 Hz, 1H), 2.98 (s, 1H), 2.13 (s, 1H), 1.82-1.74 (m, 2H), 1.26 (s, 9H); **^{13}C NMR** (100 MHz, $CDCl_3$, ppm): δ 177.0, 154.1, 143.0, 135.8, 135.6, 131.1, 128.7, 127.6, 123.1, 122.5, 117.2, 108.9, 80.1, 64.3, 60.7, 46.3, 44.3, 29.6, 28.1; **HPLC analysis:** Daicel CHIRALPAK AD-H, *n*-hexane:*i*-PrOH = 80:20, flow rate = 0.8 mL·min^{-1}, λ = 254 nm, retention time: t_R = 11.5 min (minor), t_R = 16.8 min (major).

***Tert*-butyl((*R*)-1-benzyl-3-((*S*)-1-hydroxypent-4-en-2-yl)-2-oxoindolin-3-yl) carbamate**

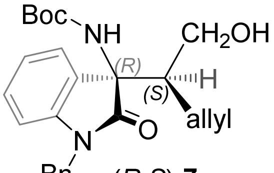

(*R,S*)-**7g**

Following the General Procedure D2, (*R,S*)-**7g** was obtained as white solid (20 h, 38.4 mg, 91% yield, 8:1 *anti/syn*, 90% ee) after flash chromatography (elution gradient: ethyl acetate:petroleum ether = 1:6); **HPLC analysis:** Daicel CHIRALPAK AD-H, *n*-hexane:*i*-PrOH = 80:20, flow rate = 0.8 mL·min^{-1}, λ = 254 nm, retention time: t_R = 11.9 min (major), t_R = 16.8 min (minor).

***Tert*-butyl((*S*)-1-benzyl-3-((*R*)-1-hydroxyhexan-2-yl)-2-oxoindolin-3-yl) carbamate**

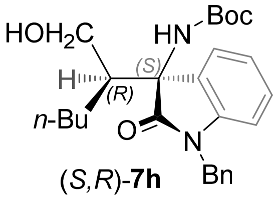

(*S,R*)-**7h**

Following the General Procedure D1, (*S,R*)-**7h** was obtained as white solid (22 h, 38.6 mg, 88% yield, >20:1 *anti/syn*, 95% ee) after flash chromatography (elution gradient: ethyl acetate:petroleum ether = 1:5); **$[\alpha]_D^{20}$** =+9.3 (*c* 0.8, $CHCl_3$); **^{1}H NMR** (400 MHz, $CDCl_3$, ppm): δ 7.38 (d, *J* = 7.6 Hz, 2H), 7.33-7.24 (m, 4H), 7.18 (t, *J* = 7.6 Hz, 1H), 7.04 (t, *J* = 7.6 Hz, 1H), 6.71 (d, *J* = 7.6 Hz, 1H), 6.64 (s, 1H), 5.17 (d, *J* = 12 Hz, 1H), 4.70 (br, 1H), 4.21-4.15 (m, 1H), 3.92 (d, *J* = 11.6 Hz, 1H), 3.06-3.02 (m, 1H), 2.02 (s, 1H), 1.26 (s, 9H), 1.12-1.06 (m, 2H), 1.04-0.99 (m, 2H), 0.89-0.83 (m, 1H), 0.82-0.78 (m, 1H), 0.68 (t, *J* = 7.2 Hz, 3H); **^{13}C NMR** (100 MHz, $CDCl_3$, ppm): δ 177.2, 154.1, 143.1, 135.9, 128.7, 128.6, 127.6, 123.0, 122.4, 108.8, 80.1, 64.5, 60.9, 46.6, 44.3, 29.7, 28.1, 24.4, 22.3, 13.6; **HPLC analysis:** Daicel CHIRALPAK AD-H, *n*-hexane:*i*-PrOH = 80:20, flow rate = 0.8 mL·min^{-1}, λ = 254 nm, retention time: t_R = 8.2 min (minor), t_R = 13.1 min (major).

***Tert*-butyl((*R*)-1-benzyl-3-((*S*)-1-hydroxyhexan-2-yl)-2-oxoindolin-3-yl) carbamate**

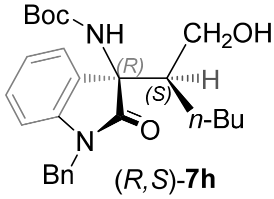

(*R,S*)-**7h**

Following the General Procedure D2, (*R,S*)-**7h** was obtained as white solid (18 h, 39.4 mg, 90% yield, 11:1 *anti/syn*, 92% ee) after flash chromatography (elution gradient: ethyl acetate:petroleum ether = 1:5); **HPLC analysis:** Daicel CHIRALPAK AD-H, *n*-hexane:*i*-PrOH = 80:20, flow rate = 0.8 mL·min^{-1}, λ = 254 nm, retention time: t_R = 8.4 min (major), t_R = 14.0 min (minor).

Benzyl ((*S*)-1-benzyl-3-((*R*)-1-hydroxypropan-2-yl)-2-oxoindolin-3-yl) carbamate

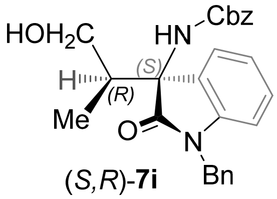

(*S,R*)-**7i**

Following the General Procedure D1, (*S,R*)-**7i** was obtained as white solid (16 h, 36.1 mg, 84% yield, 11:1 *anti/syn*, 92% ee) after flash chromatography (elution gradient: ethyl acetate:petroleum ether = 1:3); **$[\alpha]_D^{20}$** = −14.7 (*c* 1.0, $CHCl_3$); **^{1}H NMR** (400 MHz, $CDCl_3$, ppm): δ 7.70-7.25 (m, 11H), 7.19 (t, J = 7.6 Hz, 2H), 7.02 (t, J = 7.2 Hz, 1H), 6.68 (br, 1H), 4.96 (br, 4H), 3.89-3.83 (m, 1H), 3.74-3.70 (m, 1H), 2.93 (s, 1H), 2.48-2.43 (m, 1H), 0.52 (d, J = 6.8 Hz, 3H); **^{13}C NMR** (100 MHz, $CDCl_3$, ppm): δ 177.2, 154.8, 143.5, 135.9, 128.9, 128.7, 128.4, 128.2, 128.0, 127.5, 127.4, 123.9, 122.6, 109.3, 66.9, 65.4, 64.9, 44.3, 40.4, 11.5; **HPLC analysis:** Daicel CHIRALPAK AD-H, *n*-hexane:*i*-PrOH = 75:25, flow rate = 0.8 mL·min^{-1}, λ = 254 nm, retention time: t_R = 13.3 min (minor), t_R = 19.6 min (major).

Benzyl ((*R*)-1-benzyl-3-((*S*)-1-hydroxypropan-2-yl)-2-oxoindolin-3-yl) carbamate

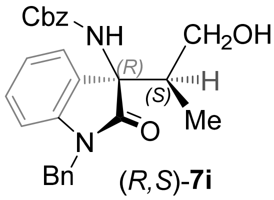

(*R,S*)-**7i**

Following the General Procedure D2, toluene as the solvent was employed and (*R,S*)-**7i** was obtained as white solid (16 h, 39.1 mg, 91% yield, 19:1 *anti/syn*, 88% ee) after flash chromatography (elution gradient: ethyl acetate:petroleum ether = 1:3). **HPLC analysis:** Daicel CHIRALPAK AD-H, *n*-hexane:*i*-PrOH = 75:25, flow rate = 0.8 mL·min^{-1}, λ = 254 nm, retention time: t_R = 12.5 min (major), t_R = 18.0 min (minor).

Supplementary Note 7

General Procedure for Asymmetric Catalytic Mannich Reactions of Monocyclic Ketimine and Propionaldehyde

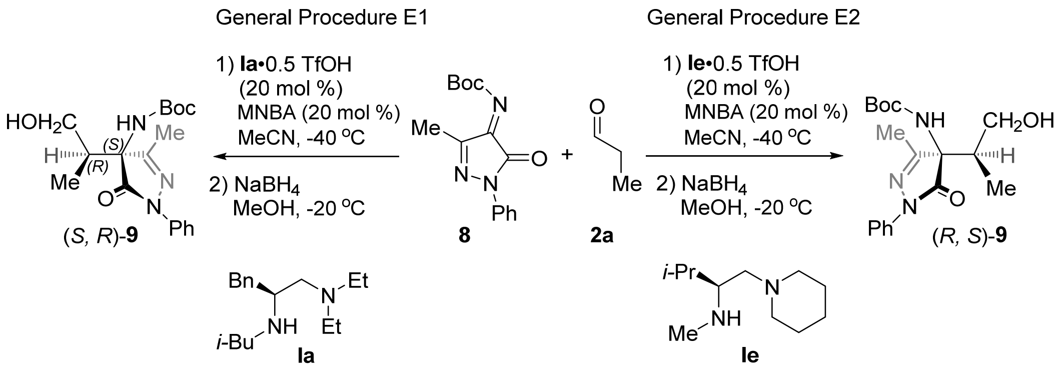

General Procedure E1: Mannich reaction catalyzed by *N*-^{i}Bu amine Ia. To a solution of monocyclic ketimine **8** (0.1 mmol), 3-nitrobenzoic acid (3.3 mg, 0.02 mmol), and catalyst **Ia**·0.5TfOH (6.7 mg, 20 mol%) in anhydrous MeCN (0.8 mL) was added propionaldehyde **2a** (0.3 mmol) at -40 °C. After the reaction mixture was stirred for10 h, $NaBH_4$ (19.2 mg, 0.5 mmol) and MeOH (0.4 mL) were added at -20 °C, and the resulting mixture was stirred for 0.5 h. Finally, the resulting mixture was purified by silica gel column chromatography (ethyl acetate:petroleum ether = 1:3) to afford Mannich product (*S*, *R*)-**9**.

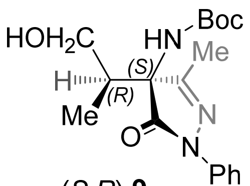

(*S*,*R*)-**9**

***Tert*-butyl((*S*)-4-((*R*)-1-hydroxypropan-2-yl)-3-methyl-5-oxo-1-phenyl-4,5-dihydro-1H-pyrazol-4-yl)carbamate ((*S*,*R*)-9)**: White solid (29.5 mg, 85% yield, >20:1 *anti/syn*, 92% ee). $[\alpha]_D^{20}$ = +24.8 (*c* 1.0, $CHCl_3$); **^{1}H NMR** (600 MHz, $CDCl_3$, ppm): δ 7.90-7.88 (m, 2H), 7.39 (t, J = 7.8 Hz, 1H), 7.25-7.22 (m, 1H), 7.18 (t, J = 7.8 Hz, 1H), 4.27 (s, 1H), 3.65 (d, J = 10.8 Hz, 1H), 2.18-2.15 (m, 1H), 2.09 (s, 3H), 1.40-1.26 (s, 9H), 0.76 (d, J = 7.2 Hz, 3H); **^{13}C NMR** (150 MHz, $CDCl_3$, ppm): δ 172.3, 160.8, 154.1, 137.9, 128.8, 125.1, 119.3, 118.4, 80.7, 69.0, 63.0, 37.3, 28.2, 12.9, 10.9;

HPLC analysis: Daicel CHIRALPAK IA, *n*-hexane:*i*-PrOH = 95:15, flow rate = 1.0 $mL\cdot min^{-1}$, λ = 254 nm, retention time: t_R = 13.2 min (minor), t_R = 14.9 min (major); **HRMS** (ESI) m/z calcd. for $C_{18}H_{25}N_3NaO_4$ $[M + Na]^+$: 370.1737, found: 370.1738.

General Procedure E2: Mannich reaction catalyzed by *N*-Me amine Ie. To a solution of a monocyclic ketimine **8** (0.1 mmol), 3-nitrobenzoic acid (3.3 mg, 0.02 mmol), and catalyst **Ie**·0.5TfOH (5.2 mg, 20 mol%) in anhydrous MeCN (0.8 mL) was added propionaldehyde **2a** (0.3 mmol) at -40 °C. After the reaction mixture was stirred for 10 h, $NaBH_4$ (19.2 mg, 0.5 mmol) and MeOH (0.4 mL) were added at -20 °C, and the resulting mixture was stirred for 0.5 h. Finally, the resulting mixture was purified by silica gel column chromatography (ethyl acetate:petroleum ether = 1:3) to afford Mannich product (*R*, *S*)**-9**.

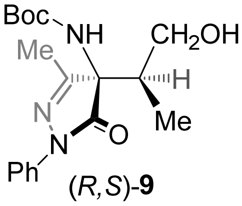

(*R*,*S*)-**9**

***Tert*-butyl((*R*)-4-((*S*)-1-hydroxypropan-2-yl)-3-methyl-5-oxo-1-phenyl-4,5-dihydro-1H-pyrazol-4-yl)carbamate ((*R*,*S*)-9)**: White solid (29.8 mg, 86% yield, 10:1 *anti/syn*, 90% ee). **HPLC analysis:** Daicel CHIRALPAK IA, *n*-hexane:*i*-PrOH = 95:15, flow rate = 1.0 $mL\cdot min^{-1}$, λ = 254 nm, retention time: t_R = 13.2 min (major), t_R = 15.2 min (minor).

Supplementary Note 8

General Procedure for Asymmetric Catalyzed Aldol Reactions of Isatins and *α*-Substituted Aldehydes

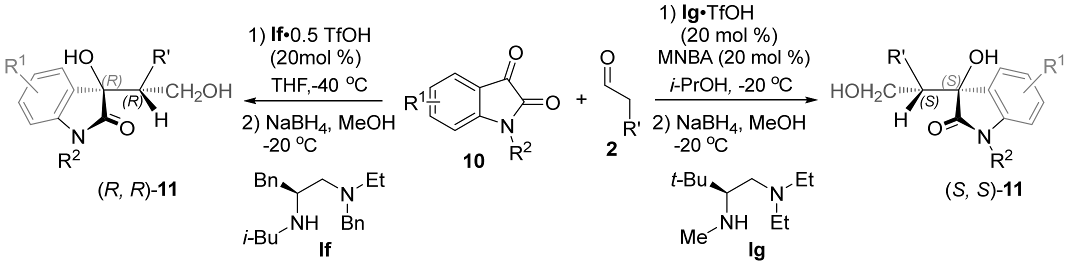

General Procedure F1: Aldol reaction catalyzed by *N*-^{i}Bu amine If. To a solution of isatins **10** (0.1 mmol), and catalyst **If**·0.5TfOH (7.9 mg, 20 mol%) in anhydrous THF (0.8 mL) was added *α*-substituted aldehydes **2** (0.3 mmol) at -40 °C. After the reaction mixture was stirred for the shown time, $NaBH_4$ (19.2 mg, 0.5 mmol) and MeOH (0.4 mL) were added at -20 °C, and the resulting mixture was stirred for 0.5 h. Finally, the resulting mixture was purified by silica gel column chromatography (ethyl acetate:petroleum ether = 1:5 to 1:1) to afford Mannich products (*R*, *R*)-**11**.

General Procedure F2: Aldol reaction catalyzed by *N*-Me amine Ig. To a solution of isatins **10** (0.1 mmol), 3-nitrobenzoic acid (3.3 mg, 0.02 mmol), and catalyst **Ig**·TfOH (6.7 mg, 20 mol%) in anhydrous *i*-PrOH (0.8 mL) was added *α*-substituted aldehydes **2** (0.3 mmol) at -20 °C. After the reaction mixture was stirred for the shown time, $NaBH_4$ (19.2 mg, 0.5 mmol) and MeOH (0.4 mL) were added at -20 °C, and the resulting mixture was stirred for 0.5 h. Finally, the resulting mixture was purified by silica gel column chromatography (ethyl acetate:petroleum ether = 1:5 to 1:1) to afford Mannich products (*S*, *S*)-**11**.

(*R*)-3-hydroxy-3-((*R*)-1-hydroxybutan-2-yl)-1-methylindolin-2-one

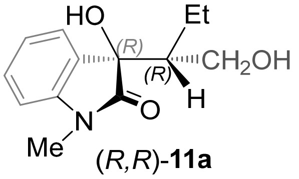

(*R,R*)-**11a**

Following the General Procedure F1, (*R,R*)-**11a** was obtained as white solid (14h, 19 mg, 81% yield, 11:1 anti/syn, 90% ee) after flash chromatography (elution gradient: ethyl acetate:petroleum ether = 1:2); **[α]$_D^{20}$** = +29.2 (*c* 1.0, $CHCl_3$); **^{1}H NMR** (400 MHz, $CDCl_3$): *δ* 7.35-7.31 (m, 2H), 7.12 (t, *J* = 7.6 Hz, 1H), 6.81 (d, *J* = 8 Hz, 1H), 4.98 (br, 1H), 4.12-4.02 (m, 2H), 3.73 (br, 1H), 3.16 (s, 3H), 2.16-2.12 (m, 1H), 1.08-0.98 (m, 1H), 0.90-0.83 (m, 1H), 0.77 (t, *J* = 7.2 Hz, 1H); **^{13}C NMR** (100 MHz, $CDCl_3$): *δ* 178.4, 143.5, 130.7, 129.7, 123.5, 123.4, 108.3, 79.4, 61.1, 48.1, 26.2, 18.7, 12.1; **HPLC analysis:** Daicel CHIRALPAK AD-H, *n*-hexane:*i*-PrOH = 85:15, flow rate = 0.8 mL·min^{-1}, λ = 254 nm, retention time: t_R = 11.5 min (major), t_R = 13.0 min (minor);

HRMS (ESI) m/z calcd.for $C_{13}H_{18}NO_3$ $[M + H]^+$: 236.1281, found: 236.1283.

(*S*)-3-hydroxy-3-((*S*)-1-hydroxybutan-2-yl)-1-methylindolin-2-one

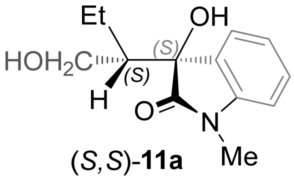

(*S,S*)-**11a**

Following the General Procedure F2, (*S,S*)-**11a** was obtained as white solid (48 h, 14.8 mg, 63% yield, 16:1 anti/syn, 93% ee) after flash chromatography (elution gradient: ethyl acetate:petroleum ether = 1:2); **HPLC analysis:** Daicel CHIRALPAK AD-H, *n*-hexane:*i*-PrOH = 85:15, flow rate = 0.8 mL·min^{-1}, λ = 254 nm, retention time: t_R = 11.6 min (minor), t_R = 12.8 min (major).

(*R*)-3-hydroxy-3-((*R*)-1-hydroxybutan-2-yl)-1-(methoxymethyl)indolin-2-one

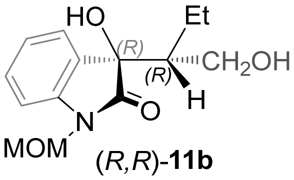

(*R,R*)-**11b**

Following the General Procedure F1, (*R,R*)-**11b** was obtained as white solid (14 h, 21.2 mg, 80% yield, 10:1 anti/syn, 91% ee) after flash chromatography (elution gradient: ethyl acetate:petroleum ether = 1:2); **$[\alpha]_D^{20}$** = +21.6 (*c* 1.0, $CHCl_3$); **^{1}H NMR** (400 MHz, $CDCl_3$): δ 7.38-7.32 (m, 2H), 7.16 (t, *J* = 7.2 Hz, 1H), 7.02 (d, *J* = 7.6 Hz, 1H), 5.15 (d, *J* = 11.2 Hz, 1H), 5.03 (d, *J* = 11.2 Hz, 1H), 4.80 (br, 1H), 4.15-4.11 (m, 2H), 3.34 (s, 3H), 3.21 (br, 1H), 2.23-2.16 (m, 1H), 1.08-1.02 (m, 1H), 0.98-0.90 (m, 1H), 0.80 (t, *J* = 7.2 Hz, 1H); **^{13}C NMR** (100 MHz, $CDCl_3$): δ 178.8, 141.9, 130.0, 129.9, 124.0, 123.7, 109.8, 79.7, 71.6, 61.6, 56.5, 48.1, 19.0, 12.2; **HPLC analysis:** Daicel CHIRALPAK IC, *n*-hexane:*i*-PrOH = 80:20, flow rate = 0.8 mL·min^{-1}, λ = 254 nm, retention time: t_R = 15.2 min (major), t_R = 19.7 min (minor); **HRMS** (ESI) m/z calcd.for $C_{14}H_{19}NNaO_4$ $[M + Na]^+$: 288.1206, found: 288.1206.

(*S*)-3-hydroxy-3-((*S*)-1-hydroxybutan-2-yl)-1-(methoxymethyl)indolin-2-one

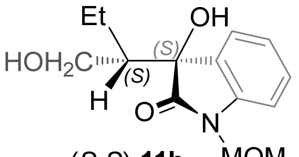

(*S,S*)-**11b**

Following the General Procedure F2, (*S,S*)-**11b** was obtained as white solid (72 h, 17.2 mg, 65% yield, 12:1 anti/syn, 90% ee) after flash chromatography (elution gradient: ethyl acetate:petroleum ether = 1:2); **HPLC analysis:** Daicel CHIRALPAK IC, *n*-hexane:*i*-PrOH = 80:20, flow rate = 0.8 mL·min^{-1}, λ = 254 nm, retention time: t_R =15.6 min (minor), t_R = 20.1 min (major).

(*R*)-4-bromo-3-hydroxy-3-((*R*)-1-hydroxybutan-2-yl)-1-methylindolin-2-one

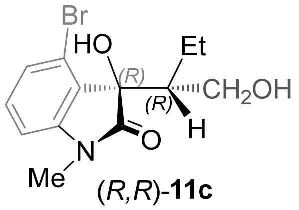

(*R,R*)-**11c**

Following the General Procedure F1, DMF as the solvent was employed and reaction temperature is 0 °C, (*R,R*)-**11c** was obtained as white solid (8 h, 27.2 mg, 87% yield, >20:1 anti/syn, 94% ee) after flash chromatography (elution gradient: ethyl acetate:petroleum ether = 3:1); **$[\alpha]_D^{20}$** = +43.5 (*c* 1.0, $CHCl_3$); **^{1}H NMR** (400 MHz, $CDCl_3$): *δ* 7.23-7.15 (m, 2H), 6.75 (d, *J* = 7.2 Hz, 1H), 5.14 (s, 1H), 4.23-4.13 (m, 2H), 3.61 (br, 1H), 3.16 (s, 3H), 2.94-2.89 (m, 1H), 1.01-0.96 (m, 1H), 0.84-0.76 (m, 4H); **^{13}C NMR** (100 MHz, $CDCl_3$): *δ* 177.4, 145.5, 131.0, 128.2, 127.8, 119.2, 107.4, 81.3, 61.1, 44.1, 26.3, 19.1, 12.0; **HPLC analysis:** Daicel CHIRALPAK AD-H, *n*-hexane:*i*-PrOH = 90:10, flow rate = 0.8 mL·min^{-1}, λ = 254 nm, retention time: t_R = 17.6 min (major), t_R = 20.8 min (minor); **HRMS** (ESI) m/z calcd.for $C_{13}H_{17}BrNO_3$ $[M + H]^+$: 314.0386, found: 314.0384.

(*S*)-4-bromo-3-hydroxy-3-((*S*)-1-hydroxybutan-2-yl)-1-methylindolin-2-one

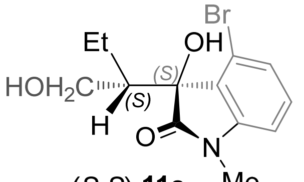

(*S,S*)-**11c**

Following the General Procedure F2, (*S,S*)-**11c** was obtained as white solid (72 h, 19.7 mg, 63% yield, 15:1 anti/syn, 86% ee) after flash chromatography (elution gradient: ethyl acetate:petroleum ether = 3:1); **HPLC analysis:** Daicel CHIRALPAK AD-H, *n*-hexane:*i*-PrOH = 90:10, flow rate = 0.8 mL·min^{-1}, λ = 254 nm, retention time: t_R = 17.9 min (minor), t_R = 20.9 min (major).

(*R*)-3-hydroxy-3-((*R*)-1-hydroxybutan-2-yl)-5-methoxy-1-methylindolin-2-one

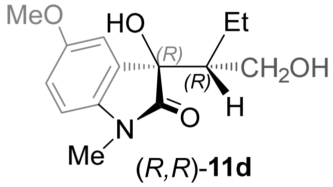

(*R,R*)-**11d**

Following the General Procedure F1, (*R,R*)-**11d** was obtained as white solid (24 h, 20.6 mg, 78% yield, 12:1 anti/syn, 90% ee) after flash chromatography (elution gradient: ethyl acetate:petroleum ether = 1:1); **$[\alpha]_D^{20}$** = +30.5 (*c* 1.0, $CHCl_3$); **^{1}H NMR** (400 MHz, $CDCl_3$): δ 6.96 (d, *J* = 1.8 Hz, 1H), 6.85 (dd, *J* = 8.4, 2.4 Hz, 1H), 6.73 (d, *J* = 8.4 Hz, 1H), 4.61 (s, 1H), 4.15-4.02 (m, 2H), 3.81 (s, 3H), 3.54-3.51 (m, 1H), 3.16 (s, 3H), 2.14-2.09 (m, 1H), 1.11-1.00 (m, 1H), 0.91-0.84 (m, 1H), 0.79 (t, *J* = 7.2 Hz, 1H); **^{13}C NMR** (100 MHz, $CDCl_3$): δ 180.04, 156.8, 136.9, 131.8, 114.1, 110.7, 108.9, 79.8, 61.3, 55.9,48.2, 26.3, 18.8, 12.0; **HPLC analysis:** Daicel CHIRALPAK AD-H, *n*-hexane:*i*-PrOH = 85:15, flow rate = 0.8 mL·min^{-1}, λ = 254 nm, retention time: t_R = 14.1 min (major), t_R = 19.0 min (minor); **HRMS** (ESI) m/z calcd. for $C_{14}H_{19}NNaO_4$ $[M + Na]^+$: 288.1206, found: 288.1207.

(*S*)-3-hydroxy-3-((*S*)-1-hydroxybutan-2-yl)-5-methoxy-1-methylindolin-2-one

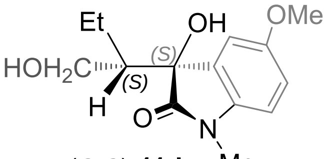

(*S,S*)-**11d**

Following the General Procedure F2, (*S,S*)-**11d** was obtained as white solid (72 h, 14.1 mg, 53% yield, 11:1 anti/syn, 92% ee) after flash chromatography (elution gradient: ethyl acetate:petroleum ether = 1:1); **HPLC analysis:** Daicel CHIRALPAK AD-H, *n*-hexane:*i*-PrOH = 85:15, flow rate = 0.8 mL·min^{-1}, λ = 254 nm, retention time: t_R = 14.4 min (minor), t_R = 19.0 min (major).

(*R*)-5-chloro-3-hydroxy-3-((*R*)-1-hydroxybutan-2-yl)-1-methylindolin-2-one

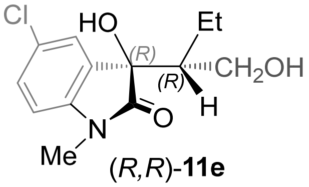

(*R,R*)-**11e**

Following the General Procedure F1, (*R,R*)-**11e** was obtained as white solid (14 h, 21.5 mg, 80% yield, 10:1 anti/syn, 90% ee) after flash chromatography (elution gradient: ethyl acetate:petroleum ether = 1:2); **$[\alpha]_D^{20}$** = +34.1 (*c* 1.0, $CHCl_3$); **^{1}H NMR** (400 MHz, $CDCl_3$): *δ* 7.33-7.27 (m, 2H), 6.74 (d, *J* = 8.4 Hz, 1H), 5.07 (s, 1H), 4.11-4.05 (m, 2H), 3.35 (br, 1H), 3.16 (s, 3H), 2.18-2.13 (m, 1H), 1.05-0.97 (m, 1H), 0.95-0.86 (m, 1H), 0.80 (t, *J* = 7.2 Hz, 1H); **^{13}C NMR** (100 MHz, $CDCl_3$): *δ* 177.9, 142.1, 132.2, 129.6, 129.0, 124.2, 109.3, 79.5, 61.5, 48.0, 26.3, 18.9, 12.0; **HPLC analysis:** Daicel CHIRALPAK AD-H, *n*-hexane:*i*-PrOH = 85:15, flow rate = 0.8 mL·min^{-1}, λ = 254 nm, retention time: t_R = 10.6 min (major), t_R = 12.3 min (minor); **HRMS** (ESI) m/z calcd.for $C_{13}H_{16}ClNNaO_3$ $[M + Na]^+$: 292.0711, found: 292.0709.

(*S*)-5-chloro-3-hydroxy-3-((*S*)-1-hydroxybutan-2-yl)-1-methylindolin-2-one

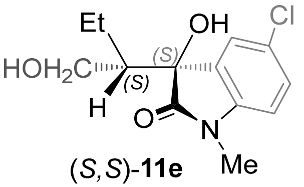

(*S,S*)-**11e**

Following the General Procedure F2, (*S,S*)-**11e** was obtained as white solid (72 h, 18.3 mg, 68% yield, 14:1 anti/syn, 92% ee) after flash chromatography (elution gradient: ethyl acetate:petroleum ether = 1:2); **HPLC analysis:** Daicel CHIRALPAK AD-H, *n*-hexane:*i*-PrOH = 85:15, flow rate = 0.8 mL·min^{-1}, λ = 254 nm, retention time: t_R = 10.8 min (minor), t_R = 12.3 min (major).

(*R*)-7-bromo-3-hydroxy-3-((*R*)-1-hydroxybutan-2-yl)-1-methylindolin-2-one

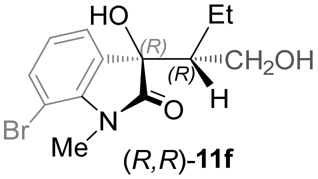

(*R,R*)-**11f**

Following the General Procedure F1, (*R,R*)-**11f** was obtained as white solid (14 h, 24.4 mg, 78% yield, 8:1 anti/syn, 93% ee) after flash chromatography (elution gradient: ethyl acetate:petroleum ether = 1:2); **[α]$_D^{20}$** = +30.6 (*c* 1.0, $CHCl_3$); **^{1}H NMR** (400 MHz, $CDCl_3$): *δ* 7.43 (d, *J* = 8.4 Hz, 1H), 7.27 (d, *J* = 8 Hz, 1H), 6.97 (t, *J* = 8 Hz, 1H), 5.20 (br, 1H), 4.14-4.05 (m, 2H), 3.54 (s, 3H), 2.15-2.09 (m, 1H), 1.02-0.93 (m, 1H), 0.88-0.81 (m, 1H), 0.77 (t, *J* = 7.2 Hz, 1H); **^{13}C NMR** (100 MHz, $CDCl_3$): *δ* 178.7, 140.8, 135.3, 134.0, 124.7, 122.6, 102.6, 78.8, 61.4, 48.2, 29.8, 18.8, 12.1; **HPLC analysis:** Daicel CHIRALPAK AD-H, *n*-hexane:*i*-PrOH = 80:20, flow rate = 0.8 mL·min^{-1}, λ = 254 nm, retention time: t_R = 10.5 min (major), t_R = 11.8 min (minor); **HRMS** (ESI) m/z calcd. for $C_{13}H_{16}BrNNaO_3$ $[M + Na]^+$: 336.0206, found: 336.0208.

(*S*)-7-bromo-3-hydroxy-3-((*S*)-1-hydroxybutan-2-yl)-1-methylindolin-2-one

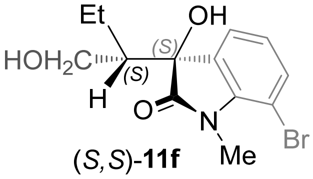

(*S,S*)-**11f**

Following the General Procedure F2, (*S,S*)-**11f** was obtained as white solid (72 h, 24.4

mg, 60% yield, 13:1 anti/syn, 90% ee) after flash chromatography (elution gradient: ethyl acetate:petroleum ether = 1:2); **HPLC analysis:** Daicel CHIRALPAK AD-H, *n*-hexane:*i*-PrOH = 80:20, flow rate = 0.8 mL·min^{-1}, λ = 254 nm, retention time: t_R = 10.6 min (minor), t_R = 11.9 min (major).

(*R*)-3-hydroxy-3-((*R*)-1-hydroxyhexan-2-yl)-1-methylindolin-2-one

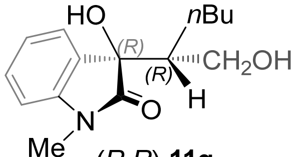

(*R*,*R*)-**11g**

Following the General Procedure F1, the reaction temperature is -20 ºC, (*R,R*)-**11g** was obtained as white solid (14 h, 20.3 mg, 77% yield, 8:1 anti/syn, 91% ee) after flash chromatography (elution gradient: ethyl acetate:petroleum ether = 1:2); **[α]$_D^{20}$** = +22.4 (*c* 1.0, $CHCl_3$); **^{1}H NMR** (400 MHz, $CDCl_3$): *δ* 7.35-7.31 (m, 2H), 7.12 (t, *J* = 7.6 Hz, 1H), 6.82 (d, *J* = 7.6 Hz, 1H), 4.84 (s, 1H), 4.09-4.01 (m, 2H), 3.60 (s, 1H), 3.18 (s, 3H), 2.26-2.22 (m, 1H),1.83 (s, 1H), 126-1.00 (m, 5H), 0.81-0.77 (m, 1H), 0.73 (t, *J* = 6.8 Hz, 1H); **^{13}C NMR** (100 MHz, $CDCl_3$): *δ* 178.3, 143.6, 130.1, 129.8, 123.5, 123.4, 108.4, 79.4, 61.7, 46.2, 29.5, 26.2, 25.2, 22.4, 13.8; **HPLC analysis:** Daicel CHIRALPAK AD-H, *n*-hexane:*i*-PrOH = 85:15, flow rate = 0.8 mL·min^{-1}, λ = 254 nm, retention time: t_R = 10.2 min (major), t_R = 11.2 min (minor); **HRMS** (ESI) m/z calcd. for $C_{15}H_{22}NO_3$ $[M + H]^+$: 264.1594, found: 264.1593.

(*S*)-3-hydroxy-3-((*S*)-1-hydroxyhexan-2-yl)-1-methylindolin-2-one

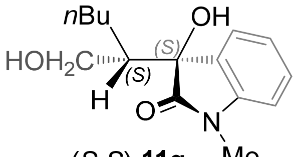

(*S*,*S*)-**11g**

Following the General Procedure F2, (*S,S*)-**11g** was obtained as white solid (72 h, 19.7 mg, 75% yield, 10:1 anti/syn, 90% ee) after flash chromatography (elution gradient: ethyl acetate:petroleum ether = 1:2); **HPLC analysis:** Daicel CHIRALPAK AD-H, *n*-hexane:*i*-PrOH = 85:15, flow rate = 0.8 mL·min^{-1}, λ = 254 nm, retention time: t_R = 10.6 min (minor), t_R = 11.4 min (major).

Supplementary Note 9

General Procedure for Asymmetric Catalytic Cross-Aldol Reactions of Aryl Aldehyde and Propionaldehyde

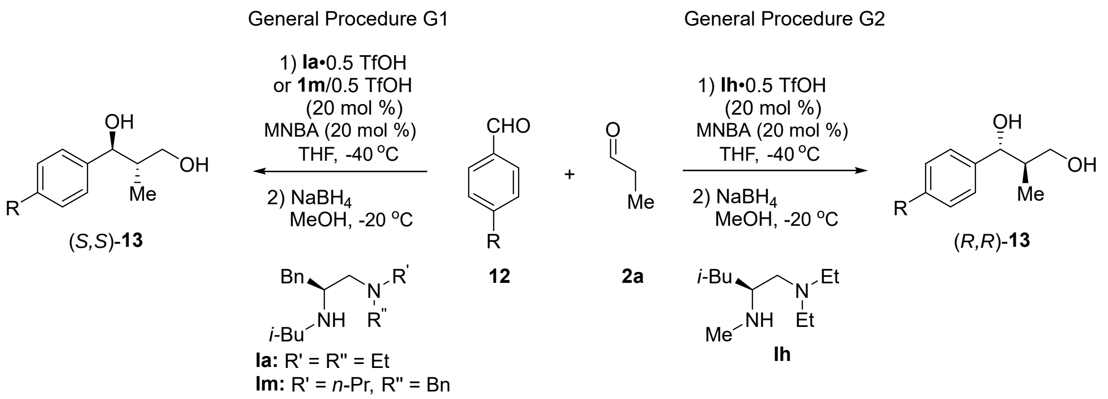

General Procedure G1: Cross-aldol reaction catalyzed by *N*-^{i}Bu amine Ia. To a solution of aryl aldehyde **12** (0.1 mmol), 3-nitrobenzoic acid (3.3 mg, 0.02 mmol), and catalyst **Ia**·0.5TfOH (6.7 mg, 20 mol%) or **1m**·0.5TfOH (8.3 mg, 20 mol%) in anhydrous THF (0.8 mL) was added propionaldehyde **2a** (0.3 mmol) at -40 °C. After the reaction mixture was stirred for 14 h, $NaBH_4$ (19.2 mg, 0.5 mmol) and MeOH (0.4 mL) were added at -20 °C, and the resulting mixture was stirred for 0.5 h. Finally, the resulting mixture was purified by silica gel column chromatography (ethyl acetate:petroleum ether = 1:1) to afford cross-aldol product (*S*, *S*)**-13**.

General Procedure G2: Cross-aldol reaction catalyzed by *N*-Me amine Ih. To a solution of aryl aldehyde **12** (0.1 mmol), 3-nitrobenzoic acid (3.3 mg, 0.02 mmol), and catalyst **Ih**·0.5TfOH (5.2 mg, 20 mol%) in anhydrous THF (0.8 mL) was added propionaldehyde **2a** (0.3 mmol) at -40 °C. After the reaction mixture was stirred for 18 h, $NaBH_4$ (19.2 mg, 0.5 mmol) and MeOH (0.4 mL) were added at -20 °C, and the resulting mixture was stirred for 0.5 h. Finally, the resulting mixture was purified by silica gel column chromatography (ethyl acetate:petroleum ether = 1:1) to afford cross-aldol product (*R*, *R*)**-13**.

(1*S*,2*S*)-2-methyl-1-(4-nitrophenyl)propane-1,3-diol

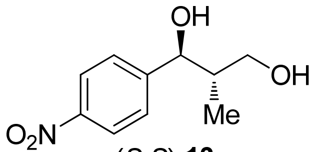

(*S,S*)-**13a**

Following the General Procedure G1, (*S,S*)-**13a** was obtained as colourless liquid (14 h, 17.9 mg, 85% yield, 16:1 *anti/syn*, 84% ee) after flash chromatography (elution gradient: ethyl acetate:petroleum ether = 1:1); **[α]$_D$^{20}** = +7.4 (*c* 1.0, $CHCl_3$); **^{1}H NMR** (400 MHz, $CDCl_3$, ppm): *δ* 8.21 (d, *J* = 8.8 Hz, 2H), 7.52 (d, *J* = 8.8 Hz, 2H), 4.70 (dd, *J* = 2.0, 8.0 Hz, 1H), 3.95 (d, *J* = 2.8 Hz, 1H), 3.82-3.78 (m, 1H), 3.72-3.67 (m, 1H), 2.72 (s, 1H), 2.05-1.99 (m, 1H), 0.76 (d, *J* = 7.2 Hz, 3H); **^{13}C NMR** (100 MHz, $CDCl_3$, ppm): *δ* 150.7, 147.4, 127.5, 123.6, 79.4, 67.5, 41.5, 13.7; **HPLC analysis:** Daicel CHIRALPAK AD-H, *n*-hexane:*i*-PrOH = 80:20, flow rate = 1.0 mL·min^{-1}, λ = 254 nm, retention time: t_R = 16.7 min (minor), t_R = 17.5 min (major).

(1*R*,2*R*)-2-methyl-1-(4-nitrophenyl)propane-1,3-diol

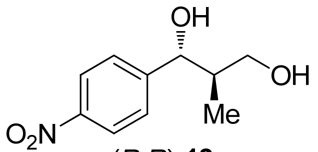

(*R,R*)-**13a**

Following the General Procedure G2, (*R,R*)-**13a** was obtained as colourless liquid (18h, 17.5 mg, 83% yield, >20:1 *anti/syn*, 90% ee) after flash chromatography (elution gradient: ethyl acetate:petroleum ether = 1:1); **[α]$_D$^{20}** = -11.7 (*c* 1.0, $CHCl_3$); **HPLC analysis:** Daicel CHIRALPAK AD-H, *n*-hexane:*i*-PrOH = 80:20, flow rate = 1.0 mL·min^{-1}, λ = 254 nm, retention time: t_R = 16.6 min (major), t_R = 17.4 min (minor).

(1*S*,2*S*)-1-(4-bromophenyl)-2-methylpropane-1,3-diol

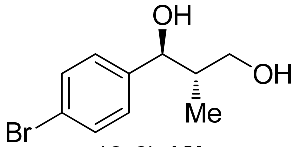

(*S,S*)-**13b**

Following the General Procedure G1, with the employment of catalyst **Im**/0.5TfOH

(8.3 mg, 20 mol%) and propionaldehyde **2a** (0.5 mmol). (*S,S*)-**13b** was obtained as colourless liquid (14h, 16.3 mg, 67% yield, 12:1 *anti/syn*, 81% ee) after flash chromatography (elution gradient: ethyl acetate:petroleum ether = 2:1); **[α]$_D$^{20}** = -26.1 (*c* 1.0, $CHCl_3$); **^{1}H NMR** (600 MHz, $CDCl_3$, ppm): δ 7.48 (d, J = 8.4 Hz, 2H), 7.22 (d, J = 8.4 Hz, 2H), 4.53 (d, J = 7.8 Hz, 1H), 3.78 (dd, J = 2.8, 10.6 Hz, 1H), 3.69 (dd, J = 7.8, 10.6 Hz, 1H), 3.18 (s, 1H), 2.67 (s, 1H), 2.02-1.98 (m, 1H), 0.71 (d, J = 7.2 Hz, 3H); **^{13}C NMR** (150 MHz, $CDCl_3$, ppm): δ 142.4, 131.5, 128.4, 121.6, 79.9, 67.7, 41.7, 13.7; **HPLC analysis:** Daicel CHIRALPAK OZ-3, *n*-hexane:*i*-PrOH = 90:10, flow rate = 1.0 mL·min^{-1}, λ = 220 nm, retention time: t_R = 11.0 min (major), t_R = 13.1 min (minor).

(1*R*,2*R*)-1-(4-bromophenyl)-2-methylpropane-1,3-diol

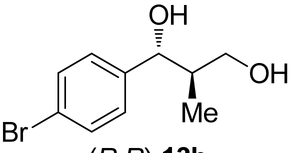

(*R,R*)-**13b**

Following the General Procedure G2, (*R,R*)-**13b** was obtained as colourless liquid (18h, 15.4 mg, 63% yield, 11:1 *anti/syn*, 83% ee) after flash chromatography (elution gradient: ethyl acetate:petroleum ether = 2:1); **[α]$_D$^{20}** = +31.4 (*c* 1.0, $CHCl_3$); **HPLC analysis:** Daicel CHIRALPAK OZ-3, *n*-hexane:*i*-PrOH = 90:10, flow rate = 1.0 mL·min^{-1}, λ = 220 nm, retention time: t_R = 11.0 min (minor), t_R = 13.1 min (major).

(1*S*,2*S*)-2-methyl-1-phenylpropane-1,3-diol

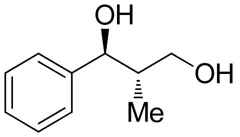

(*S,S*)-**13c**

Following the General Procedure G1, with the employment of catalyst **Im**/0.5TfOH (8.3 mg, 20 mol%) and propionaldehyde **2a** (0.5 mmol). (*S,S*)-**13c** was obtained as colourless liquid (14h, 10.3 mg, 62% yield, 12:1 *anti/syn*, 82% ee) after flash chromatography (elution gradient: ethyl acetate:petroleum ether = 2:1); **[α]$_D$^{20}** = -21.3 (*c* 1.0, $CHCl_3$); **^{1}H NMR** (600 MHz, $CDCl_3$, ppm): δ 7.37-73.4 (m, 4H), 7.32-7.30 (m,

1H), 4.55 (d, J = 8.4 Hz, 1H), 3.79-3.71 (m, 2H), 2.85 (s, 1H), 2.81 (s, 1H), 2.08-2.058 (m, 1H), 0.71 (d, J = 7.2 Hz, 3H); **^{13}C NMR** (150 MHz, $CDCl_3$, ppm): δ 143.4, 128.5, 127.9, 126.7, 80.8, 67.9, 41.8, 13.8; **HPLC analysis:** Daicel CHIRALPAK IC, *n*-hexane:*i*-PrOH = 95:5, flow rate = 1.0 mL·min^{-1}, λ = 215 nm, retention time: t_R = 23.5 min (major), t_R = 29.3 min (minor).

(1*R*,2*R*)-2-methyl-1-phenylpropane-1,3-diol

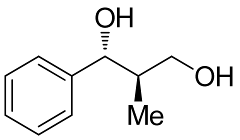

(*R,R*)-**13c**

Following the General Procedure G2, (*R,R*)-**13c** was obtained as colourless liquid (18h, 9.5 mg, 57% yield, 8:1 *anti/syn*, 80% ee) after flash chromatography (elution gradient: ethyl acetate:petroleum ether = 2:1); **$[\alpha]_D^{20}$** = +27.9 (*c* 1.0, $CHCl_3$); **HPLC analysis:** Daicel CHIRALPAK IC, *n*-hexane:*i*-PrOH = 95:5, flow rate = 1.0 mL·min^{-1}, λ = 215 nm, retention time: t_R = 24.4 min (minor), t_R = 29.4 min (major).

Supplementary Note 10

General Procedure for Asymmetric Catalytic *α*-Amination Reactions of Aldehyde 2

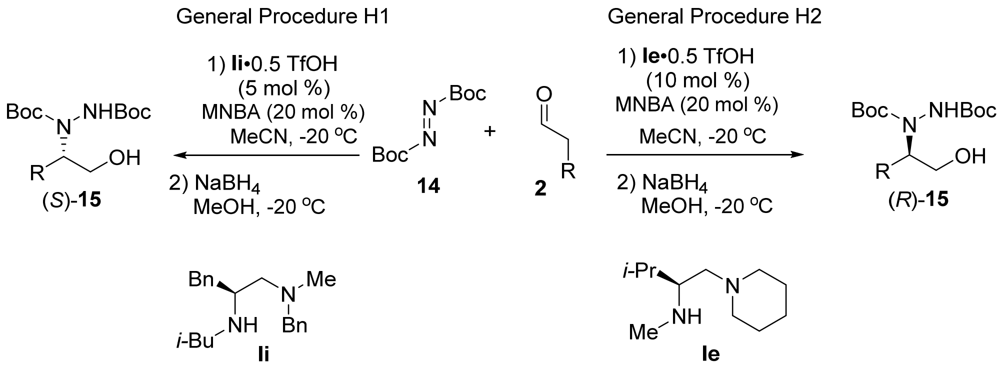

General Procedure H1: *α*-Amination reaction catalyzed by *N*-^{i}Bu amine Ii. To a solution of Di-*tert*-Butyl azodicarboxylate **14** (0.1 mmol), 3-nitrobenzoic acid (3.3 mg, 0.02 mmol), and catalyst **Ii**·0.5TfOH (1.9 mg, 5 mol%) in anhydrous MeCN (0.8 mL) was added *α*-substituted aldehydes **2** (0.3 mmol) at -20 °C. After the reaction mixture

was stirred for the shown time, $NaBH_4$ (19.2 mg, 0.5 mmol) and MeOH (0.4 mL) were added at -20 °C, and the resulting mixture was stirred for 0.5 h. Finally, the resulting mixture was purified by silica gel column chromatography (ethyl acetate:petroleum ether = 1:5) to afford product (*S*)-**15**.

General Procedure H2: *α*-Amination reaction catalyzed by *N*-Me amine Ie. To a solution of di-*tert*-butyl azodicarboxylate **14** (0.1 mmol), 3-nitrobenzoic acid (3.3 mg, 0.02 mmol), and catalyst **Ie**·0.5TfOH (2.6 mg, 10 mol%) in anhydrous MeCN (0.8 mL) was added *α*-substituted aldehydes **2** (0.3 mmol) at -20 °C. After the reaction mixture was stirred for the shown time, $NaBH_4$ (19.2 mg, 0.5 mmol) and MeOH (0.4 mL) were added at -20 °C, and the resulting mixture was stirred for 0.5 h. Finally, the resulting mixture was purified by silica gel column chromatography (ethyl acetate:petroleum ether = 1:5) to afford product (*R*)-**15**.

(*S*)-di-*tert*-butyl 1-(1-hydroxybutan-2-yl)hydrazine-1,2-dicarboxylate

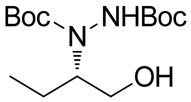

(*S*)-**15a**

Following the General Procedure H1, (*S*)-**15a** was obtained as white solid (2 h, 27.7 mg, 91% yield, 90% ee) after flash chromatography (elution gradient: ethyl acetate:petroleum ether = 1:5); $[\alpha]_D^{20}$ = +10.6 (*c* 1.2, $CHCl_3$); **^{1}H NMR** (600 MHz, $CDCl_3$, ppm): δ 6.27 (s, 1H), 4.51-4.14 (m, 2H), 3.53-3.37 (m, 2H), 1.49 (s, 18H), 1.38-1.32 (m, 2H), 0.95-0.87 (m, 3H); **^{13}C NMR** (150 MHz, $CDCl_3$, ppm): δ 158.5, 157.9, 156.3, 155.3, 82.4, 82.2, 81.9, 81.2, 62.2, 62.0, 59.9, 28.2, 28.1, 21.1, 10.5; **HPLC analysis:** The enantiomeric excess wasdetermined using the 4-nitrobenzoyl ester derivative, Daicel CHIRALPAK IA, *n*-hexane:*i*-PrOH = 80:20, flow rate = 1.0 mL·min^{-1}, λ = 254 nm, retention time: t_R = 9.3 min (major), t_R = 14.9 min (minor).

(*R*)-di-*tert*-butyl 1-(1-hydroxybutan-2-yl)hydrazine-1,2-dicarboxylate

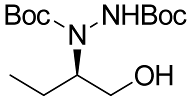

(*R*)-**15a**

Following the General Procedure H2, (*R*)-**15a** was obtained as white solid (2 h, 28.3 mg, 93% yield, 90% ee) after flash chromatography (elution gradient: ethyl acetate:petroleum ether = 1:5); **HPLC analysis:** The enantiomeric excess wasdetermined using the 4-nitrobenzoyl ester derivative, Daicel CHIRALPAK IA, *n*-hexane:*i*-PrOH = 80:20, flow rate = 1.0 mL·min^{-1}, λ = 254 nm, retention time: t_R = 9.3 min (minor), t_R = 14.9 min (major).

(*S*)-di-*tert*-butyl 1-(1-hydroxypropan-2-yl)hydrazine-1,2-dicarboxylate

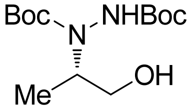

(*S*)-**15b**

Following the General Procedure H1, (*S*)-**15a** was obtained as white solid (2 h, 25.5 mg, 88% yield, 90% ee) after flash chromatography (elution gradient: ethyl acetate:petroleum ether = 1:4); **$[\alpha]_D^{20}$** = +13.2 (*c* 1.0, $CHCl_3$); **^{1}H NMR** (600 MHz, $CDCl_3$, ppm): δ 6.28 (s, 1H), 4.52-4.32 (m, 2H), 3.46-3.38 (m, 2H), 1.49 (s, 18H), 1.38-1.32 (m, 2H), 0.98 (d, J = 7.2 Hz, 3H); **^{13}C NMR** (150 MHz, $CDCl_3$, ppm): δ 158.5, 157.9, 155.7, 154.9, 82.2, 81.9, 81.4, 81.2, 63.4, 56.0, 53.7, 28.2, 28.1, 13.7; **HPLC analysis:** The enantiomeric excess wasdetermined using the 4-nitrobenzoyl ester derivative, Daicel CHIRALPAK IA, *n*-hexane:*i*-PrOH = 80:20, flow rate = 1.0 mL·min^{-1}, λ = 254 nm, retention time: t_R = 9.6 min (major), t_R = 10.9 min (minor). **HRMS** (ESI) m/z calcd. for $C_{13}H_{26}N_2NaO_5$ $[M + Na]^+$: 313.1734, found:313.1732.

(*R*)-di-*tert*-butyl 1-(1-hydroxypropan-2-yl)hydrazine-1,2-dicarboxylate

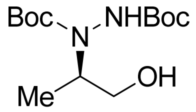

(*R*)-**15b**

Following the General Procedure H2, (*R*)-**15b** was obtained as white solid (3 h, 26.7 mg, 92% yield, 90% ee) after flash chromatography (elution gradient: ethyl acetate:petroleum ether = 1:4); **HPLC analysis:** The enantiomeric excess wasdetermined using the 4-nitrobenzoyl ester derivative, Daicel CHIRALPAK IA, *n*-hexane:*i*-PrOH = 80:20, flow rate = 1.0 mL·min^{-1}, λ = 254 nm, retention time: t_R = 9.5 min (minor), t_R = 10.9min (major).

Supplementary Note 11

Assignment of Absolute Configuration for Products

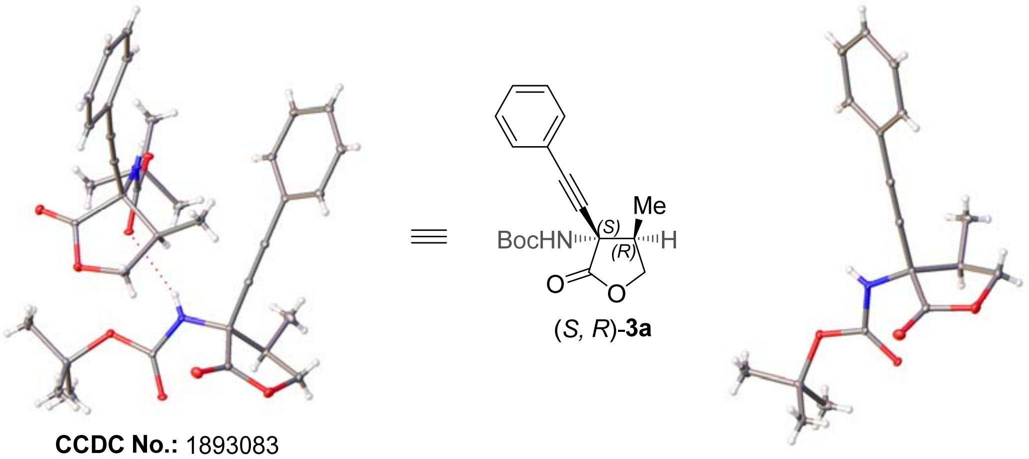

Supplementary Figure 2. Crystal Structure of (*S,R*)-**3a.**

The absolute configuration of **3a** obtained from the Gerneral Precedure B1 was confirmed to be *S,R* by X-ray analysis (Supplementary Fig. 2), and accordingly the reaction enantioselectivity and its enantiomer obtained from the Gerneral Precedure B2 in other cases was assigned by analogy.

Supplementary Table 3. Crystal data and structure refinement for (*S,R*)-**3a**.

Identification code	DJ_1
Empirical formula	$C_{36}H_{42}N_2O_8$
Formula weight	630.71
Temperature/K	100
Crystal system	orthorhombic
Space group	$P2_12_12_1$
a/Å	11.85330(18)
b/Å	15.9437(2)
c/Å	18.5905(3)
α/°	90
β/°	90
γ/°	90
Volume/Å^3	3513.34(9)
Z	4
ρ_{calc}g/cm^3	1.192
μ/mm^{-1}	0.688
F(000)	1344.0
Crystal size/mm^3	0.77 × 0.59 × 0.5
Radiation	CuKα (λ = 1.54184)
2Θ range for data collection/°	7.304 to 138.702
Index ranges	-13 ≤ h ≤ 14, -19 ≤ k ≤ 16, -21 ≤ l ≤ 22
Reflections collected	19353
Independent reflections	6466 [R_{int} = 0.0462, R_{sigma} = 0.0384]
Data/restraints/parameters	6466/0/424
Goodness-of-fit on F^2	1.077
Final R indexes [I>=2σ (I)]	R_1 = 0.0361, wR_2 = 0.0927

Final R indexes [all data]	$R_1 = 0.0382$, $wR_2 = 0.0947$
Largest diff. peak/hole / e Å^{-3}	0.24/-0.26
Flack parameter	0.00(8)

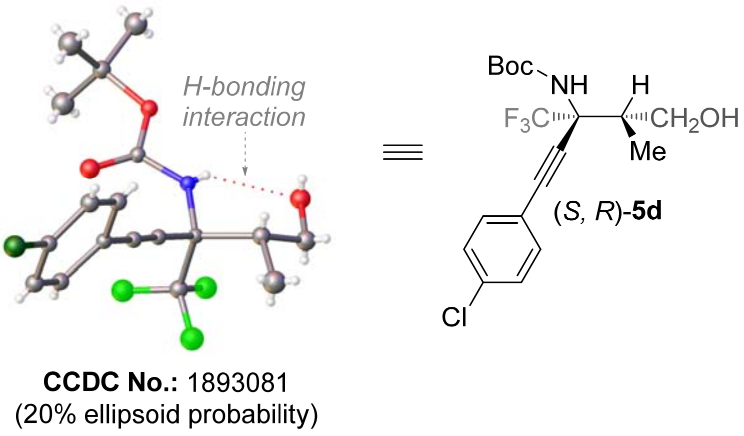

Supplementary Figure 3. Crystal Structure of (*S,R*)-**5d.**

The absolute configuration of **5d** obtained from the Gerneral Precedure C1 was confirmed to be *S,R* by X-ray analysis (Supplementary Fig. 3), and accordingly the reaction enantioselectivity and its enantiomer obtained from the Gerneral Precedure C2 in other cases was assigned by analogy.

Supplementary Table 4. Crystal data and structure refinement for (*S,R*)-**5d**.

Identification code	1
Empirical formula	$C_{18}H_{21}ClF_3NO_3$
Formula weight	391.81
Temperature/K	293(2)
Crystal system	orthorhombic
Space group	$P2_12_12_1$
a/Å	10.682(4)
b/Å	12.522(4)
c/Å	14.908(5)

α/°	90
β/°	90
γ/°	90
Volume/Å^3	1994.0(12)
Z	4
ρ_{calc}g/cm^3	1.305
μ/mm^{-1}	0.235
F(000)	816.0
Crystal size/mm^3	0.240 × 0.170 × 0.130
Radiation	MoKα (λ = 0.71073)
2Θ range for data collection/°	4.248 to 50.294
Index ranges	-12 ≤ h ≤ 11, -14 ≤ k ≤ 14, -17 ≤ l ≤ 17
Reflections collected	11453
Independent reflections	3556 [R_{int} = 0.0659, R_{sigma} = 0.0725]
Data/restraints/parameters	3556/0/240
Goodness-of-fit on F^2	0.901
Final R indexes [I>=2σ (I)]	R_1 = 0.0596, wR_2 = 0.1484
Final R indexes [all data]	R_1 = 0.1337, wR_2 = 0.1855
Largest diff. peak/hole / e Å^{-3}	0.34/-0.21
Flack parameter	0.06(8)

According to the reported similar products,[2] the abosulte configfuration of oxindole-derived Mannich products **7** obtained from Generenal Procedure D1 were assigned to be S,R. And the abosulte configruation of Mannich products **7** obtained from Generenal Procedure D2 were *R,S* by analoy.

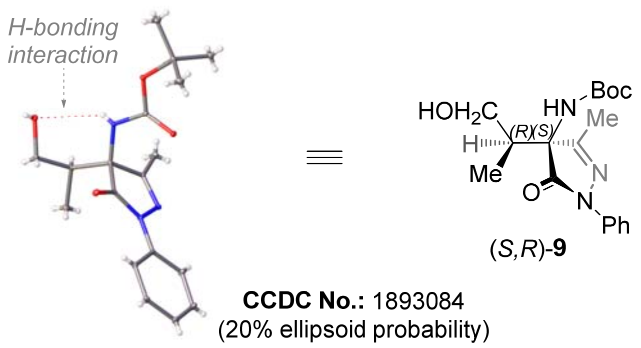

Supplementary Figure 4. Crystal Structure of (*S,R*)-**9**.

The absolute configuration of **9** obtained from the Gerneral Precedure E1 was confirmed to be *S,R* by X-ray analysis (Supplementary Fig. 4), and accordingly its enantiomer obtained from the Gerneral Precedure E2 in other cases was assigned by analogy.

Supplementary Table 5. Crystal data and structure refinement for (*S,R*)-**9**.

Identification code	DJ_2B
Empirical formula	$C_{18}H_{25}N_3O_4$
Formula weight	347.41
Temperature/K	100
Crystal system	monoclinic
Space group	$P2_1$
a/Å	7.43766(9)
b/Å	12.31890(14)
c/Å	10.66767(12)
α/°	90
β/°	108.3505(12)
γ/°	90
Volume/Å^3	927.708(19)
Z	2
ρ_{calc}g/cm^3	1.244

μ/mm^{-1}	0.726
F(000)	372.0
Crystal size/mm^3	0.86 × 0.62 × 0.41
Radiation	CuKα (λ = 1.54184)
2Θ range for data collection/°	11.312 to 138.216
Index ranges	$-8 \leq h \leq 8$, $-14 \leq k \leq 13$, $-12 \leq l \leq 12$
Reflections collected	9103
Independent reflections	3214 [R_{int} = 0.0317, R_{sigma} = 0.0233]
Data/restraints/parameters	3214/1/233
Goodness-of-fit on F^2	1.078
Final R indexes [I>=2σ (I)]	R_1 = 0.0310, wR_2 = 0.0769
Final R indexes [all data]	R_1 = 0.0317, wR_2 = 0.0776
Largest diff. peak/hole / e Å^{-3}	0.21/-0.15
Flack parameter	0.00(11)

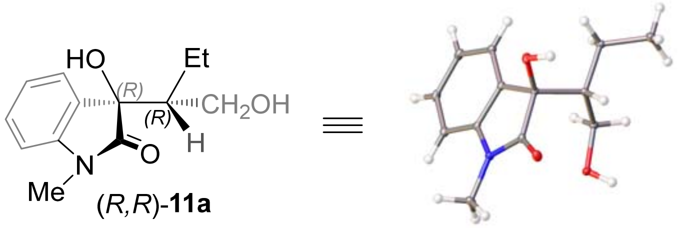

CCDC No.: 1857209
(30% ellipsoid probability)

Supplementary Figure 5. Crystal Structure of (*R,R*)-**11a**.

The absolute configuration of **11a** obtained from the Gerneral Precedure F1 was confirmed to be *R,R* by X-ray analysis (Supplementary Fig. 5), and accordingly the reaction enantioselectivity and its enantiomer obtained from the Gerneral Precedure F2 in other cases was assigned by analogy.

Supplementary Table 6. Crystal data and structure refinement for (*R,R*)-**11a**.

Identification code	cu_zhb_dj1_0m
Empirical formula	$C_{13}H_{17}NO_3$

Formula weight	235.27
Temperature/K	100(2)
Crystal system	monoclinic
Space group	$P2_1$
a/Å	8.6498(2)
b/Å	5.83660(10)
c/Å	12.5642(3)
α/°	90
β/°	106.2330(10)
γ/°	90
Volume/Å^3	609.02(2)
Z	2
ρ_{calc}g/cm^3	1.283
μ/mm^{-1}	0.744
F(000)	252.0
Crystal size/mm^3	0.500 × 0.470 × 0.270
Radiation	CuKα (λ = 1.54178)
2Θ range for data collection/°	10.652 to 139.582
Index ranges	$-10 \leq h \leq 10$, $-7 \leq k \leq 7$, $-14 \leq l \leq 15$
Reflections collected	6765
Independent reflections	2075 [R_{int} = 0.0268, R_{sigma} = 0.0240]
Data/restraints/parameters	2075/1/163
Goodness-of-fit on F^2	1.096
Final R indexes [I>=2σ (I)]	R_1 = 0.0251, wR_2 = 0.0658
Final R indexes [all data]	R_1 = 0.0251, wR_2 = 0.0658
Largest diff. peak/hole / e Å^{-3}	0.21/-0.14
Flack parameter	0.10(5)

Supplementary Note 12

DFT and Transition State Theory Calculations

All the DFT calculations were carried out with the GAUSSIAN 09 series of programs.[3] DFT method B3-LYP-D3[4-7] with a standard 6–31G(d) basis set was used for geometry optimizations. The solvent effects were considered by with an SMD[8] solvation model. Harmonic vibrational frequency calculations were performed for all of the stationary points to confirm them as a local minima or transition structures, and to derive the thermochemical corrections for the enthalpies and free energies. The M06[9] functional in combination with the 6–311+G(d) basis set was used to calculate the solvation single point energies to give more accurate energy information. To investigate whether this discrepancy is due to the employed functional, we have tested some other functionals, such as B3LYP-D3, M11[10], ωB97XD[11] and M06-2X[8], which also take the dispersion energy into consideration. As shown in the Supplementary Table 7, the computational results by different functionals to calculate the selected transtion states were summarized. Although there remain numerical differences, the using of different functionals guide the same conclusion. Thus the reuslts suggest that the theoretically predicted enantioselectivity is independent of functional choice. For the asymmetric Mannich reaction between the *β,γ*-alkynyl-*α*-imino ester **1a** and propionaldehyde **2a** catalyzed by **Ia** (Supplementary Fig. 7), the solvent effects were considered by with an SMD solvation model in the MeCN solvent. For the reversal enantioselective Mannich reaction catalyzed by **Id** (Supplementary Fig. 8), the solvent effects were considered by an SMD solvation model in the DCE solvent. The energies G_{M06} given the text are the M06 calculated Gibbs free energies in solvent, which are obtained through Supplementary Equation 1. All the data of DFT calculations is in the files of Supplementary Data.

$$\boldsymbol{G}_{\text{M06}} = \boldsymbol{E}_{\text{solv-M06}} + \boldsymbol{G}_{\text{corr-B3LYP-D3}} \qquad (1)$$

Supplementary Table 7. Benchmark of Different DFT Functional.

$\Delta\Delta G^{\neq}$ (kcal/mol)	**TS-I-(Ia-*S,R*)**	**TS-II-(Ia-*R,S*)**	**TS-V (Ia-*R,R*)**	**TS-VI (Ia-*S,S*)**
M06	0.0	2.2	7.5	9.8
B3LYP-D3	0.0	2.6	7.0	8.8
M11	0.0	2.1	6.4	8.1
ωB97XD	0.0	2.5	7.3	8.9
M06-2X	0.0	2.0	6.1	8.1

As shown in Supplementary Fig. 6, according to the density functional theory (DFT) calculations, the *E-s-trans* enamine species generated from *N*-R^2 amino-catalysts (R^2 = *i*Bu, *n*Pr, Et) and propionaldehyde **2a** were preferred to *s-syn* C-N skeleton of enamine conformation **int-I** (Supplementary Fig. 6a, 6b, 6c and 6e), while *s-anti* C-N skeleton of enamine conformation **int-II** were favorable for enamine species generated from *N*-Me amino-catalysts (Supplementary Fig. 6d and 6f). Typically, the related free energy of *s-syn* C-N skeleton of enamine conformation **Ia-int-I** generated from *N*-*i*-Bu amines **Ia** and aldehyde **2a** was 1.6 kcal/mol lower than that of *s-anti* **Ia-int-II**, in which the repulsion is severe (Supplementary Fig. 6a). Whereas the enamine species *s-anti* **Id-int-II** generated from *N*-Me amines **Id** and aldehyde **2a** has a less steric crowding than *s-syn* **If-int-I** resulting in an exotherm of 1.9 kcal/mol (Supplementary Fig. 6f). Newman projections about the C−N bond reveals that the repulsion between adjacent groups leads to the different thermal stability of enamine species. The free energy discrepancy well correlates with substituents' volume N atom adjacent to C=C bond. Notably, the corresponding differences in the free energies are in reasonable agreement with the experimental enantiodiscrimination of the Mannich reactions between ketimine **1a** and aldehyde **2a**. With these preliminary results, we presumed that some other challenging and significant stereodivergent enamine reactions could be achieved by the employing of these newly flexible acyclic chiral secondary aminocatalysts with achiral secondary *N*-substitutions.

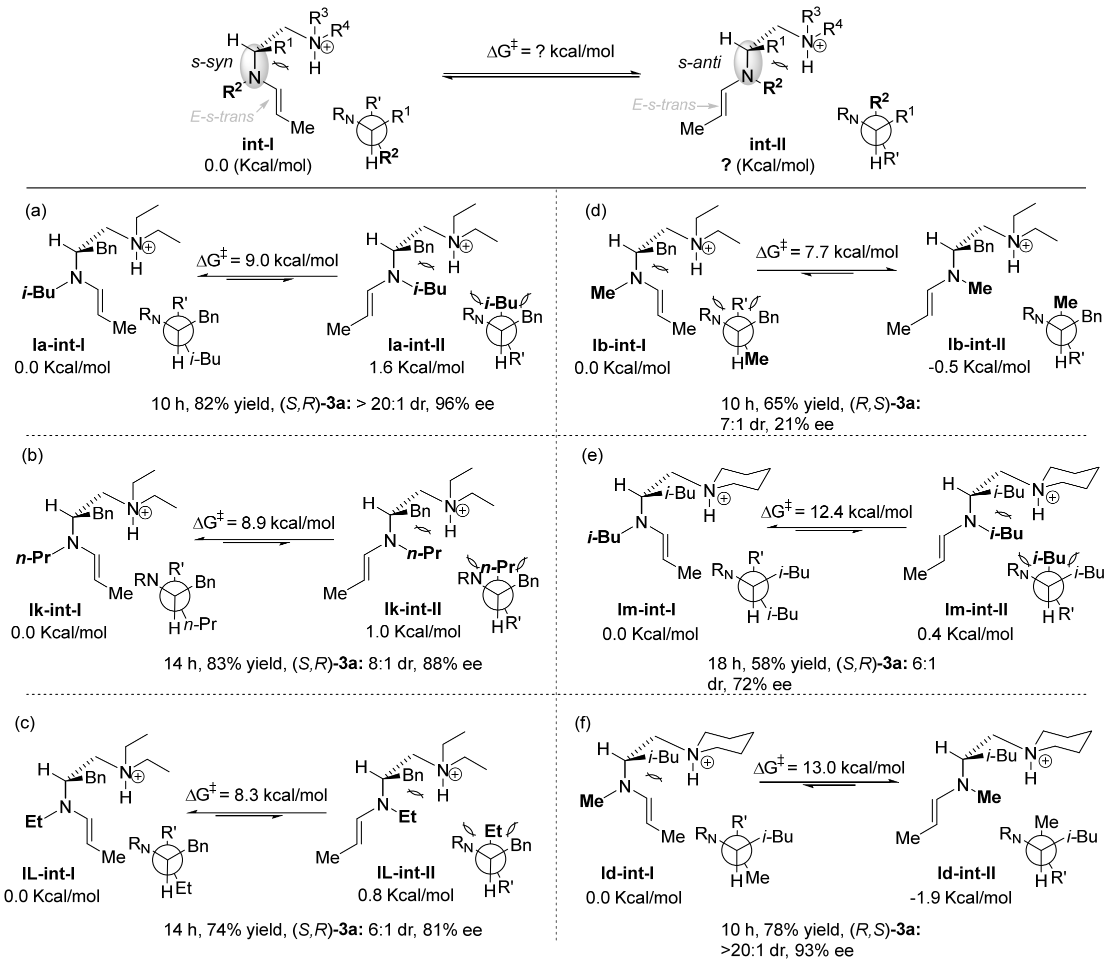

Supplementary Figure 6. DFT calculations for enamine species. (a) Related free energy of enamines from **Ia** and propionaldehyde **2a**. (b) Related free energy of enamines from **Ik** and propionaldehyde **2a**. (c) Related free energy of enamines from **IL** and propionaldehyde **2a**. (d) Related free energy of enamines from **Ib** and propionaldehyde **2a**. (e) Related free energy of enamines from **Im** and propionaldehyde **2a**. (f) Related free energy of enamines of **Id** and propionaldehyde **2a**.

To further shed light on the origin of the observed stereoselective reversal, the *anti*-selective enantiodivergent Mannich reactions exemplified by ketimine **1a** and aldehyde **2a** was investigated computationally by density functional theory (DFT) calculations. As Supplementary Fig. 7 and 8 shown, four diastereomeric transition states for the C-C bond-formation step have been proposed to be a 9-membered cycle.[12] For the Mannich reaction catalyzed by *N*-*i*-Bu amines **1a** (Supplementary Fig. 7), **TS-I, TS-II** lead to the major and the minor enantiomers for major product diastereomers,

respectively. **TS-V** and **TS-VI** lead to the minor product diastereomers. Among these four TS, the **TS-I** had the lowest free energy of activation (2.2, 7.7 and 9.7 kcal/mol lower than **TS-II**, **TS-V** and **TS-VI**, respectively).

Although **TS-II** is favoured in a crown conformation, **TS-II** is energetically disfavourable due to the involving *s-anti* C-N skeleton, in which the repulsion is severe. On the contrary, the energetically favourable *s-syn* C-N skeleton is present in chair-boat conformation **TS-I**. Additionally, the staggered conformation about the forming C-C bonds for **TS-I** (θ = 175.1°) is also more favourable than **TS-II** (θ = 171.9°) by the

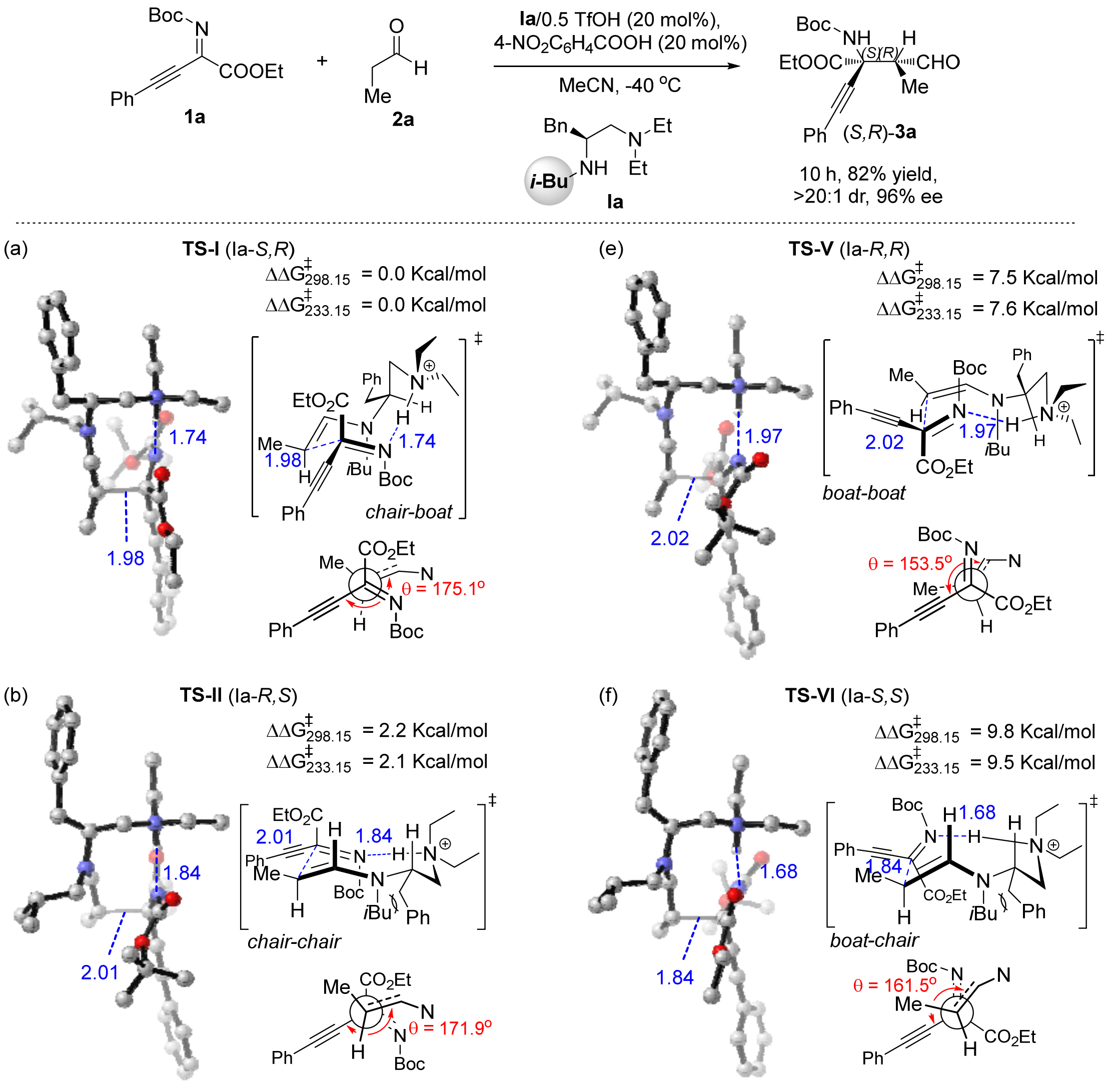

Supplementary Figure 7. Acyclic chiral secondary amines **Ia** catalyzed asymmetric Mannich reaction. (a) Transition State for the (*S,R*)-**3a**. (b) Transition State for the (*R,S*)-**3a**. (e) Transition State for the (*R,R*)-**3a**. (f) Transition State for the (*S,S*)-**3a**.

dihedral-angle value of 3.2°. The relative free energy of **TS-I** is 2.2 kcal/mol than that of **TS-II**, which is in agreement with the computed energy difference of enamines species between *s-syn* **Ia-int-I** and *s-anti* **Ia-int-II**. Thus, we presume that the strain resulted by conformation of C-N skeleton is the main factor for the enantiocontrol in the Mannich reaction catalyzed by *N*-*i*-Bu amines **1a**.

TS-V and **TS-VI** lead to the minor *syn*-product diastereomers. Although **TS-V** has the energetically favourable *s-syn* C-N skeleton in enamine motif, it is disfavoured in a boat-boat conformation. **TS-VI** has the energetically disfavourable *s-syn* C-N skeleton in enamine motif, while it is in a boat-chair conformation. Both transition states are in the less staggered conformation about the forming C-C bonds, making them to be disfavored.

Supplementary Fig. 8 show the enantio- and diastereo-selective transition states for C-C bond-forming step in the Mannich reaction catalyzed by *N*-Me amines **1d**. **TS-III** and **TS-IV** lead to the minor and the major enantiomers for the major product diastereomers, respectively. **TS-VII** and **TS-VIII** lead to the minor product diastereomers. Among these four TS, the **TS-IV** had the lowest free energy of activation (1.7, 7.4 and 7.4 kcal/mol lower than **TS-III**, **TS-VII** and **TS-VIII**, respectively). In **TS-IV**, it is in a favourable crown conformation, in which a more fully staggered conformation about the forming C-C bonds (θ = 177.3°) is favoured by the dihedral-angle value of 0.6°. In addition, **TS-IV** is also present in an energetically favourable *s-anti* C-N skeleton**.** The disfavoured **TS-III**, which is 1.7 kcal/mol higher in energy, is in a disfavourable chair-boat conformation of 9-membered transition state and *s-syn* C-N skeleton.

TS-VII and **TS-VIII** lead to the minor *syn*-product diastereomers. Both transition states are involed in the disfavored *E-s-cis* emaimes. **TS-VII** has the energetically favourable *s-syn* C-N skeleton in enamine motif, and it is in a boat-chair conformation. **TS-VIII** has the energetically disfavourable *s-syn* C-N skeleton in enamine motif. And **TS-VIII** is in a twist boat-boat conformation, which has severe flag replusion. Thus, both transition states are in the less staggered conformation about the forming C-C

bonds, making them to be disfavored.

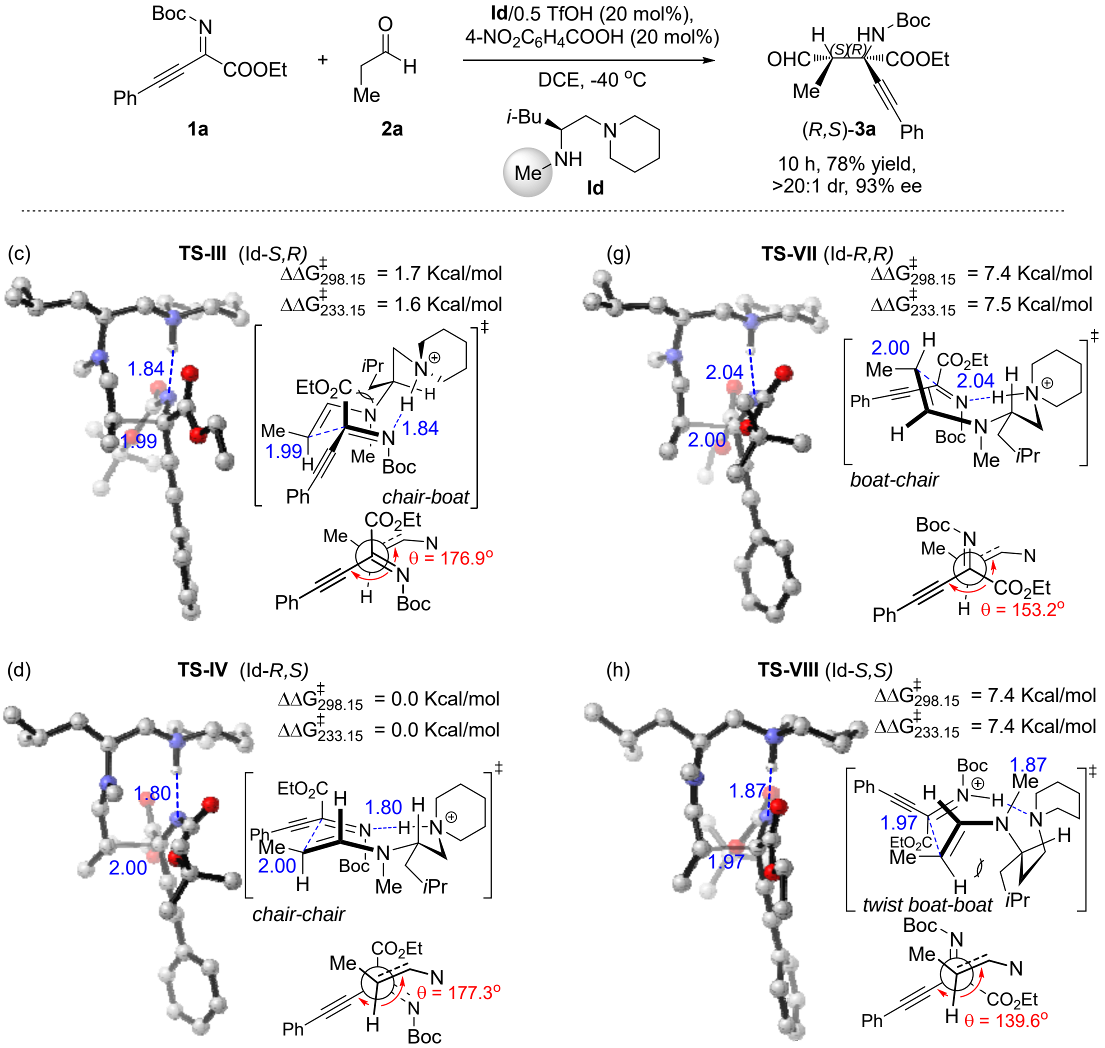

Supplementary Figure 8. Reversal enantioseletive Mannich reaction of **Id**. (c) Transition State for the (*S,R*)-**3a**. (d) Transition State for the (*R,S*)-**3a**. (g) Transition State for the (*R,R*)-**3a**. (h) Transition State for the (*S,S*)-**3a**.

Among all these eight 9-memebered cyclic transition states, **TS-III** and **TS-IV** mediated by *N*-Me amines **Id** have the more fully staggered (less partial eclipsed) conformation than both **TS-I** and **TS-II** mediated by *N*-*i*-Bu amines **Ia**. The result indicates that the repulsion of *i*-Bu is more severe than the Me group, leading to the distortion of staggered conformation about the forming C-C bond. Therefore, in the catalysis of *N*-*i*-Bu amines **Ia**, the favourable conformation of *s-syn* C-N skeleton mainly contributed to the *anti*-selective enantioselective Mannich reactions via the favoured **TS-I**, affording the (*S,R*)-product enantiomer. In the catalysis of *N*-Me amines **Id**, both the favourable conformation of *s-anti* C-N skeleton and crown 9-memebered

cyclic transition states contribute to the energetic discrimination in the chirality-imparting step, involving the favoured **TS-IV** to give the (*R,S*)-product enantiomer.

Whole free energy profiles for Ia catalyzed Mannich reaction of 1a and 2a

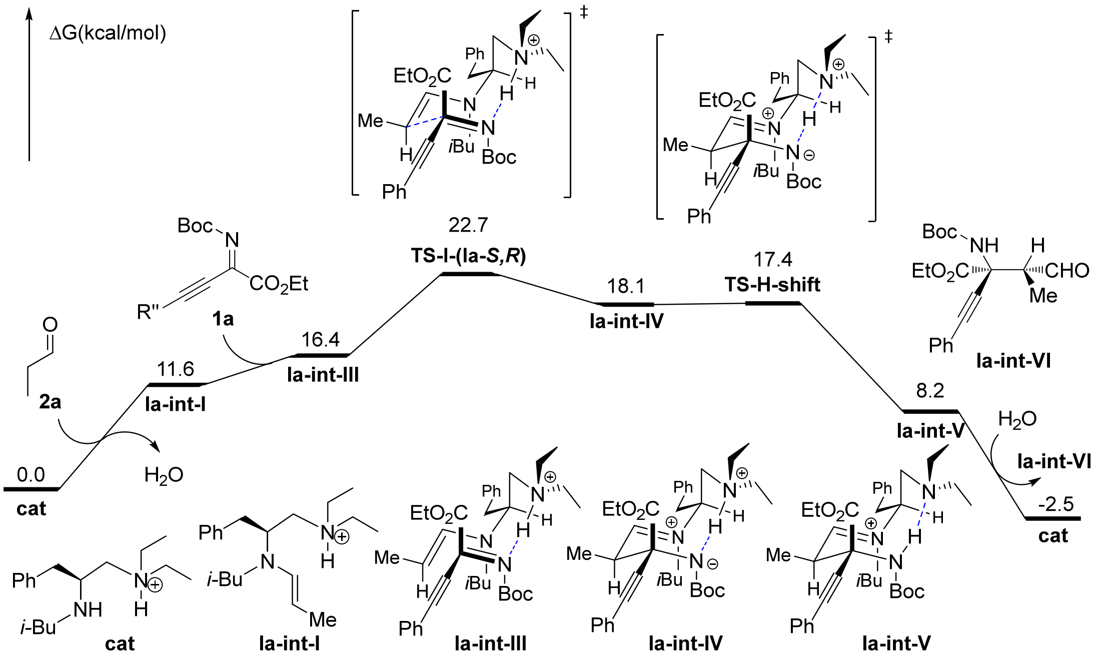

Supplementary Figure 9. Free energy profiles for **Ia** catalyzed Mannich reaction between **1a** and **2a**.

We have calculated the whole free energy profile for the **Ia** catalyzed Mannich reaction of **1a** and **2a**, and the corresponding results were summarized in Supplementary Fig. 9. Starting from the protonated catalyst **Ia** (**cat**), the dehydration condensation of propanal **2a** affords the active enamine **Ia-int-I**, from which the hydrogen-bond interaction leads to the generation of intermediate **Ia-int-III**. Subsequent nucleophilic addition proceeds via transiton state **TS-I-(Ia-*S,R*)** generating a zwitterionic intermediate **Ia-int-IV**. The intramolecular proton transfer then occurs rapidly via **TS-H-shift** to form cationic imine **Ia-int-V**. Final hydrolysis regenerates the active catalyst **cat** and release the intermediate products **Ia-int-V**. The calculated results show that the nucleophilic addition is the rate- and enantioselective-determining step among the reaction pathway, and the overall activation free energy is determined to be 22.7 kcal/mol.

Selected descriptions of transtion states TS-I-(Ia-*S*,*R*) (nucleophilic addition) and TS-H-shift (proton transfer)

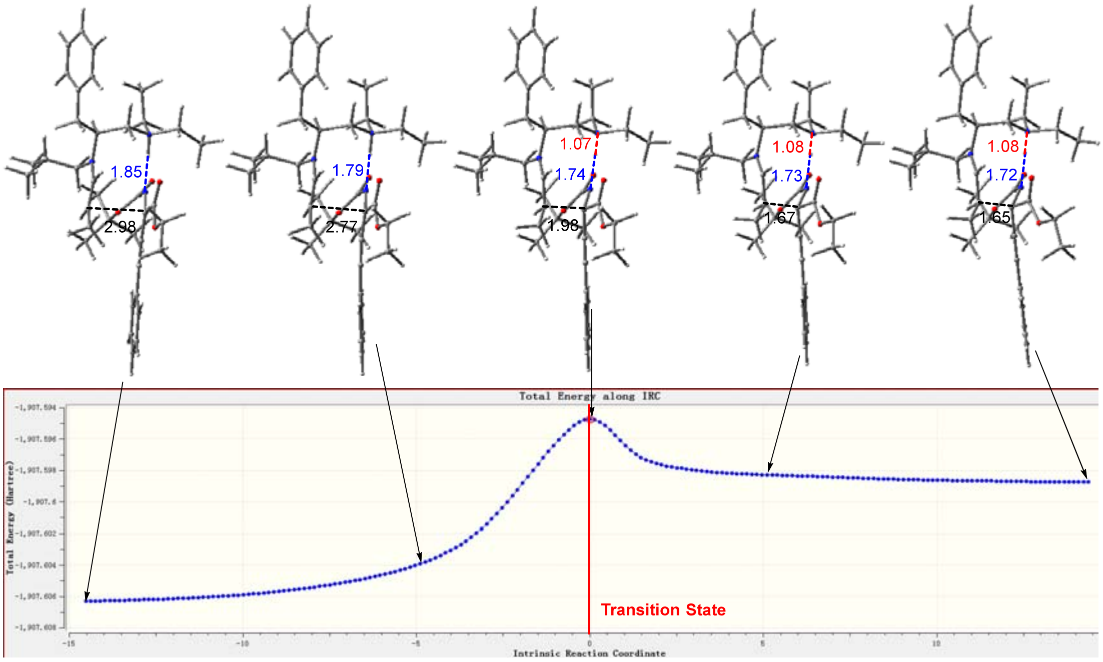

Supplementary Figure 10. Intrinsic reaction coordinate (IRC) calculation of concerted hydride/proton transfer transition state **TS-I-(Ia-*S*,*R*)**.

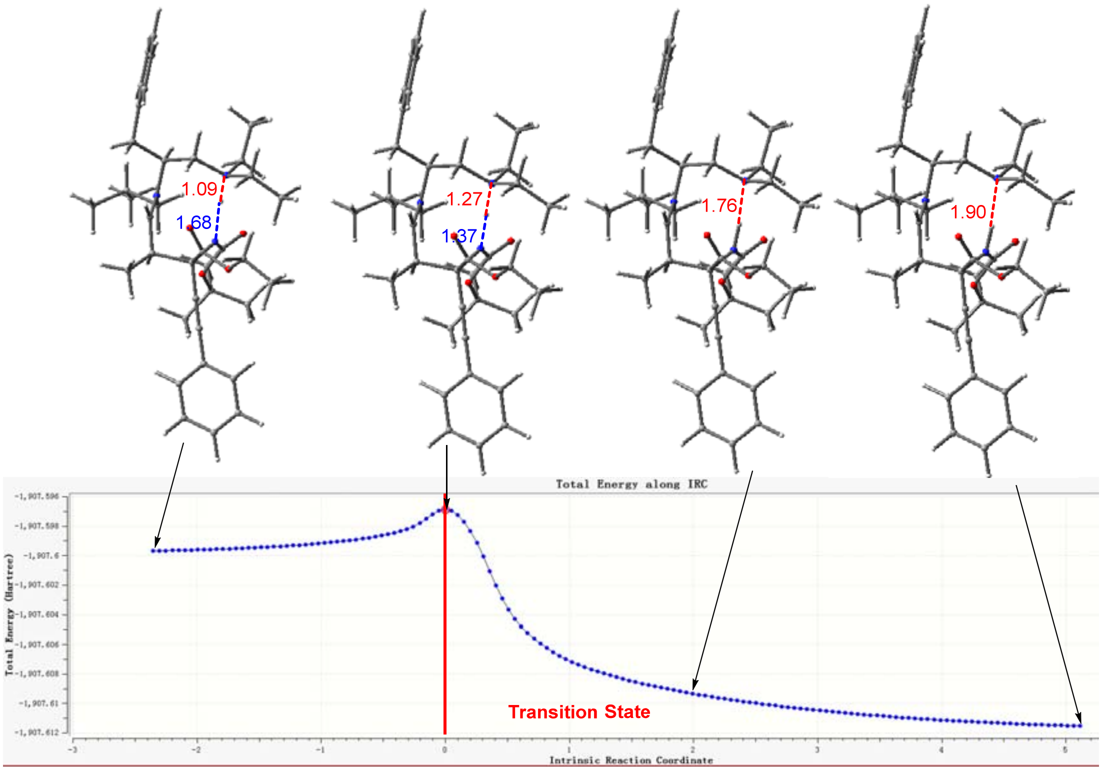

Supplementary Figure 11. Intrinsic reaction coordinate (IRC) calculation of concerted hydride/proton transfer transition state **TS-H-shift**.

Supplementary Table 8. Absolute Calculation Energies, Enthalpies, and Free Energies

Geometry	$E_{(elec\text{-}B3LYP\text{-}D3)}$[a]	$G_{(corr\text{-}B3LYP\text{-}D3)}$[b]	$H_{(corr\text{-}B3LYP\text{-}D3)}$[c]	$E_{(solv,\ M06)}$[d]	IF[e]
**Ia-int-I**	-892.629453	0.471607	0.552661	-892.05291	-
**Ia-int-II**	-892.62734	0.471243	0.552544	-892.04995	-
**TS-rotation-Ia**	-892.61538	0.472337	0.551186	-892.039355	-12.27
**Ik-int-I**	-853.312364	0.442948	0.522902	-852.762219	-
**Ik-int-II**	-853.310964	0.443255	0.522888	-852.760862	-
**TS-rotation-Ik**	-853.299561	0.445598	0.521749	-852.750707	-27.35
**IL-int-I**	-813.995625	0.417399	0.493048	-813.473391	-
**IL-int-II**	-813.994484	0.417182	0.493115	-813.471908	-
**TS-rotation-IL**	-813.982784	0.418915	0.491743	-813.461743	-32.53
**Ib-int-I**	-774.67702	0.390656	0.463437	-774.184326	-
**Ib-int-II**	-774.678087	0.391101	0.463445	-774.1855	-
**TS-rotation-Ib**	-774.66546	0.39098	0.461571	-774.172359	-40.12
**Im-int-I**	-817.626935	0.486853	0.564315	-817.09037	-
**Im-int-II**	-817.626571	0.487223	0.564055	-817.090068	-
**TS-rotation-Im**	-817.610045	0.490489	0.563593	-817.074322	-24.33
**Id-int-I**	-699.674284	0.406468	0.474738	-699.220108	-
**Id-int-II**	-699.67748	0.407092	0.47452	-699.223788	-
**TS-rotation-Id**	-699.653938	0.408228	0.473974	-699.201139	-69.42
**TS-I (Ia-*S,R*)**	-1907.5595	0.780732	0.909947	-1906.518631	-349.67
**TS-V (Ia-*R,R*)**	-1907.546809	0.780385	0.910108	-1906.506347	-367.65
**TS-VI (Ia-*S,S*)**	-1907.547335	0.782927	0.91016	-1906.50522	-349.01
**TS-II (Ia-*R,S*)**	-1907.55567	0.780586	0.909413	-1906.514993	-334.54
**TS-III (Id-*S,R*)**	-1714.600399	0.71732	0.832333	-1713.681124	-336.53
**TS-VII (Id-*R,R*)**	-1714.589714	0.715789	0.832598	-1713.670567	-365.48
**TS-VIII (Id-*S,S*)**	-1714.589269	0.715453	0.831674	-1713.670175	-362.34
**TS-IV (Id-*R,S*)**	-1714.601755	0.71589	0.831983	-1713.682452	-332.27
**cat**	-775.913052	0.415016	0.487338	-775.413709	-

**1a**	-1014.90154	0.276352	0.354723	-1014.451168	-
**2a**	-193.153532	0.057357	0.090797	-193.068917	-
**H_2O**	-76.414981	0.002577	0.024688	-76.413591	-
**Ia-int-III**	-1907.568566	0.777481	0.910595	-1906.525355	-
**Ia-int-IV**	-1907.564656	0.781713	0.911699	-1906.526917	-
**Ia-int-V**	-1907.577117	0.781183	0.911363	-1906.542118	-
**Ia-int-VI**	-1208.086454	0.359971	0.449569	-1207.550367	-
**TS-H-shift**	-1907.561823	0.776966	0.90685	-1906.523234	-1341.04

[a]The electronic energy calculated by B3LYP-D3 in solvent.

[b]The thermal correction to Gibbs free energy calculated by B3LYP-D3 in solvent.

[c]The thermal correction to enthalpy calculated by B3LYP-D3 in solvent.

[d]The electronic energy calculated by M06 in solvent.

[e]The B3LYP-D3 calculated imaginary frequencies for the transition states.

Supplementary Table 9. The Electronic Single Point Energy Calculated by B3LYP-D3, M11, ωB97XD and M06-2X in MeCN

Geometry	$E_{(solv,B3LYP-D3)}$[a]	$E_{(solv,M11)}$[b]	$E_{(solv,\ \omega B97XD)}$[c]	$E_{(solv,M06-2x)}$[d]
**TS-I (Ia-*S,R*)**	-1908.033473	-1906.804808	-1907.316122	-1907.100313
**TS-V (Ia-*R,R*)**	-1908.021942	-1906.794682	-1907.30422	-1907.089716
**TS-VI (Ia-*S,S*)**	-1908.021602	-1906.794109	-1907.304088	-1907.089621
**TS-II (Ia-*R,S*)**	-1908.029132	-1906.801441	-1907.31202	-1907.096815

[a]The electronic energy calculated by B3LYP-D3 in the solvent of MeCN.

[b]The electronic energy calculated by M11 in the solvent of MeCN.

[c]The electronic energy calculated by ωB97XD in the solvent of MeCN.

[d] The electronic energy calculated by M06-2X in the solvent of MeCN.

Supplementary Note 13

Copies of NMR and HPLC Spectrum

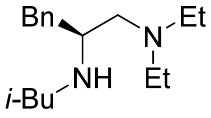

Ia

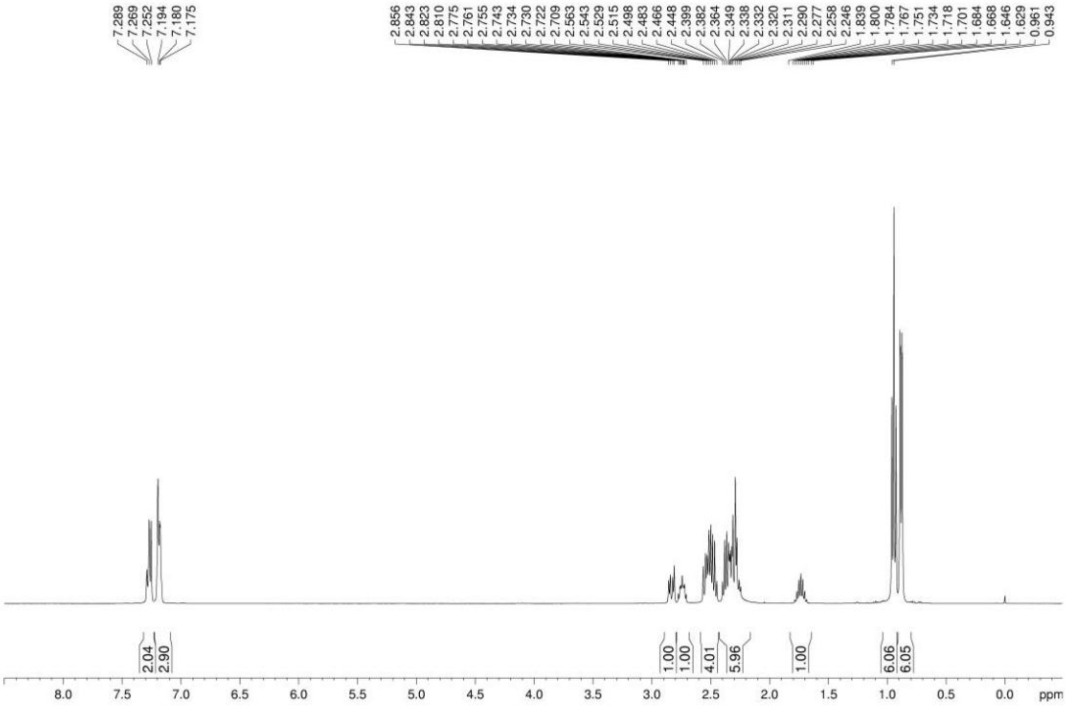

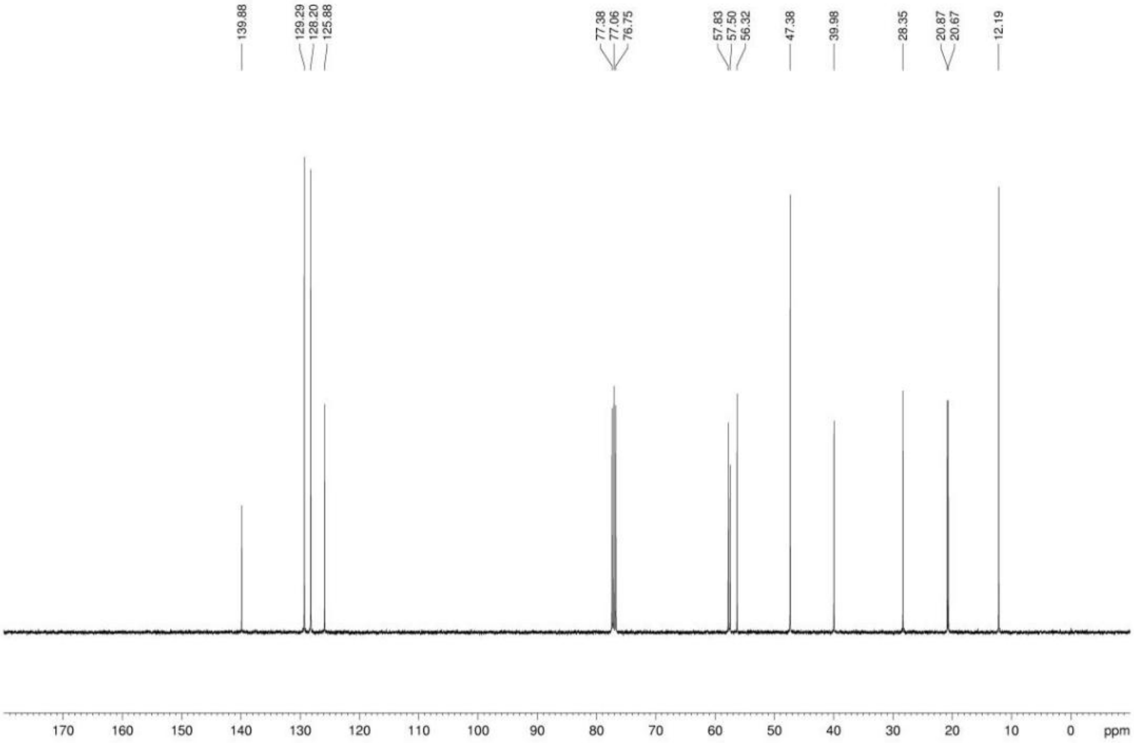

Supplementary Figure 12. ^{1}H and ^{13}C-NMR Spectrum for **Ia.**

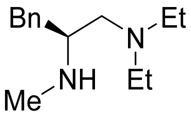

Ib

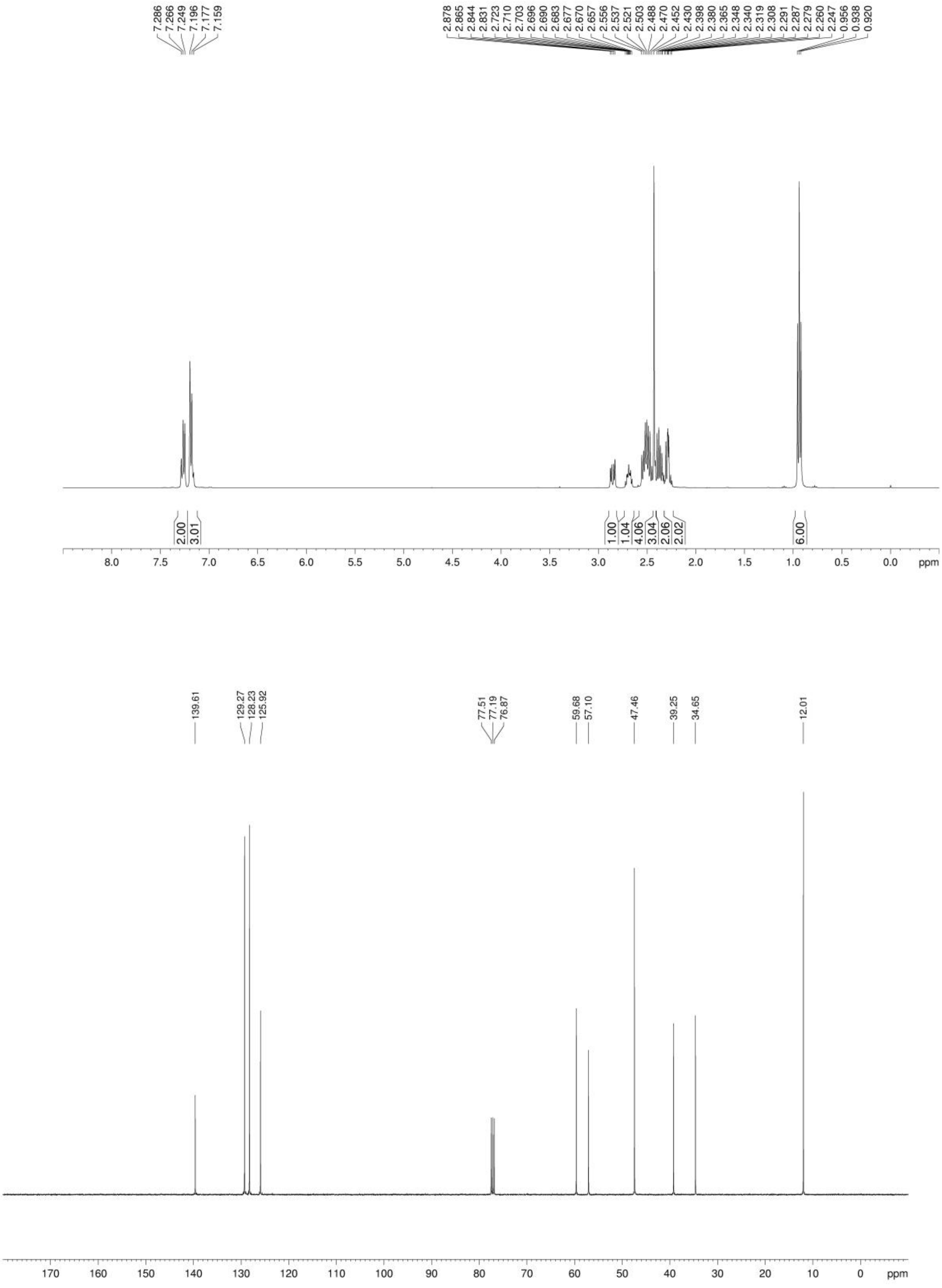

Supplementary Figure 13. ^{1}H and ^{13}C-NMR Spectrum for **Ib.**

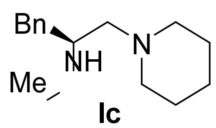

Ic

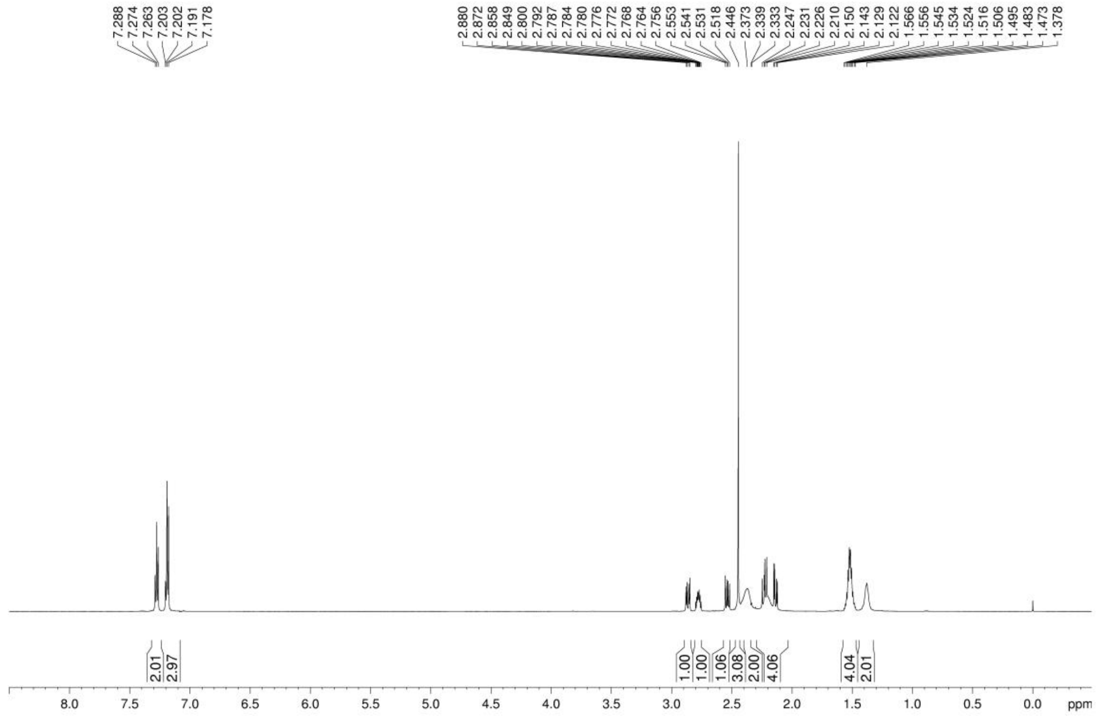

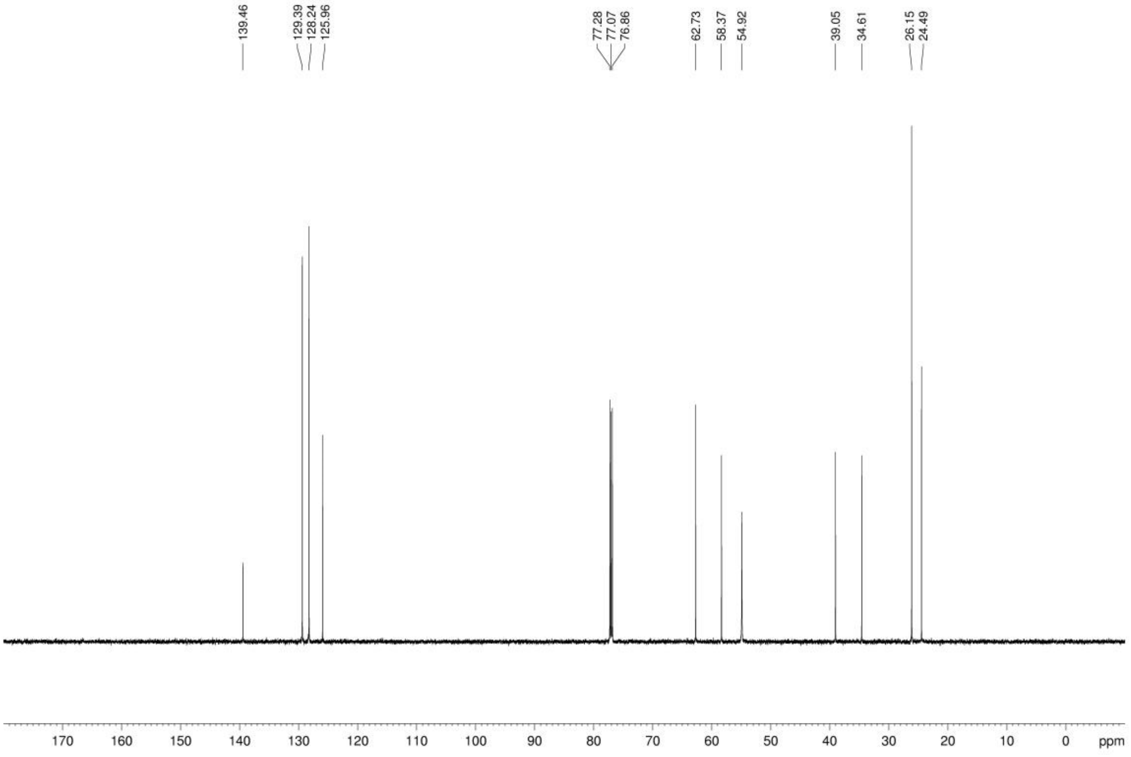

Supplementary Figure 14. ^{1}H and ^{13}C-NMR Spectrum for **Ic.**

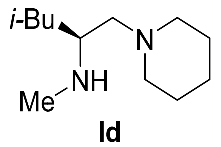

Id

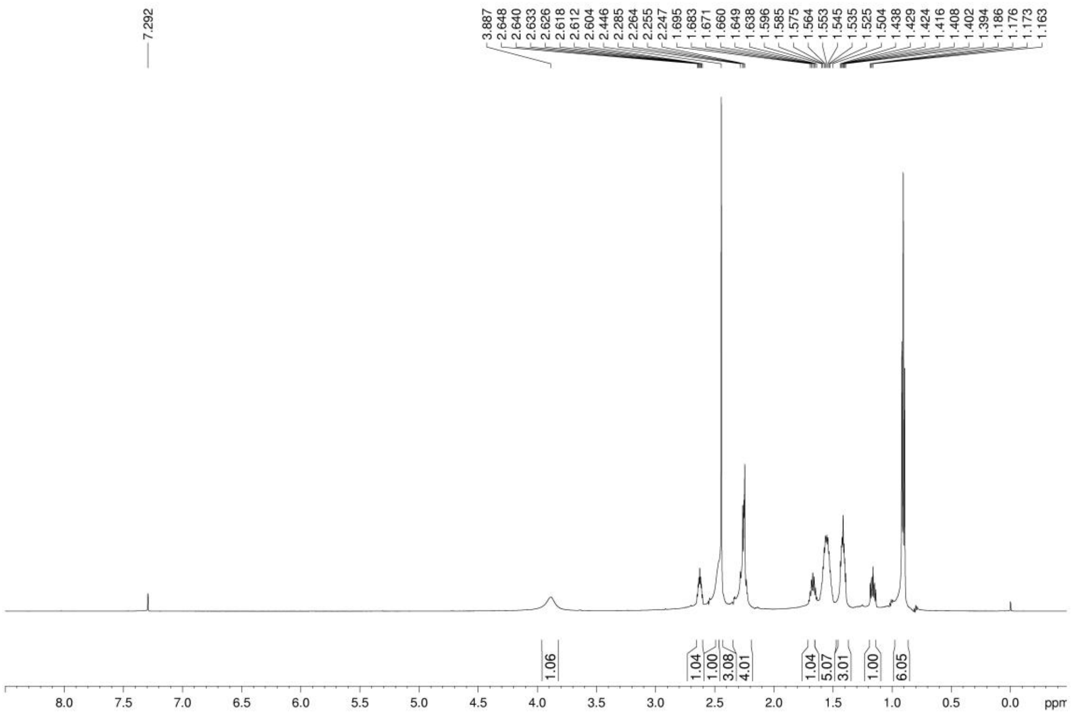

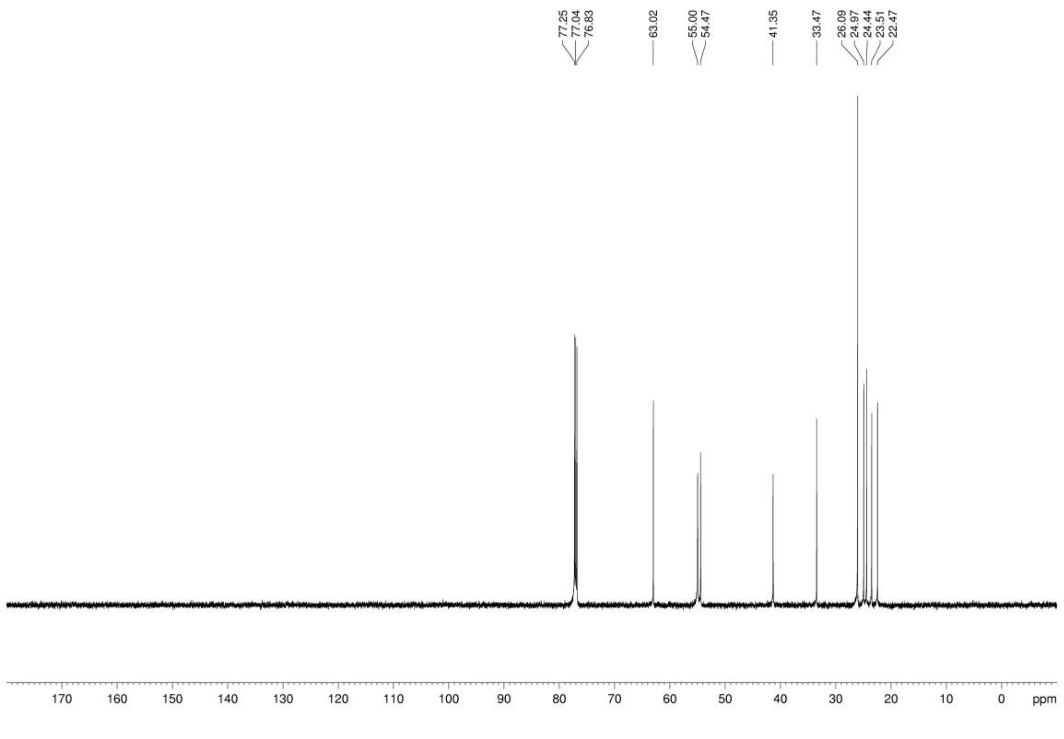

Supplementary Figure 15. ^{1}H and ^{13}C-NMR Spectrum for **Id.**

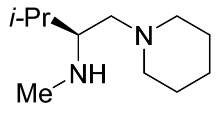

Ie

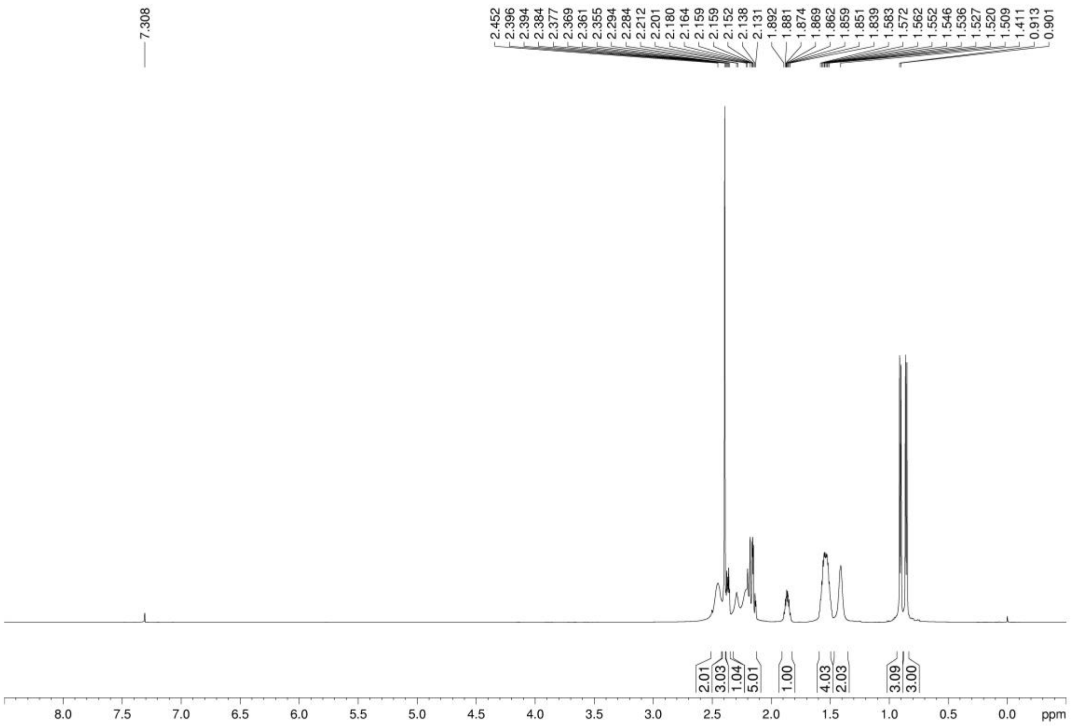

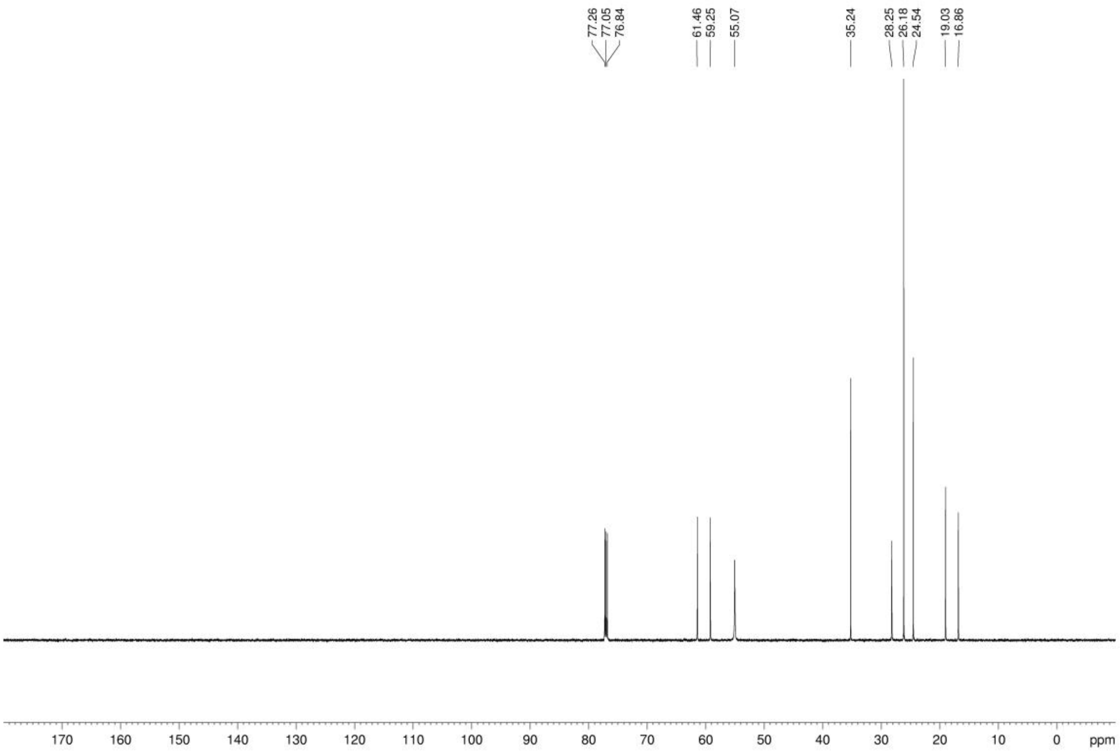

Supplementary Figure 16. ^{1}H and ^{13}C-NMR Spectrum for **Ie**

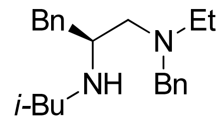

If

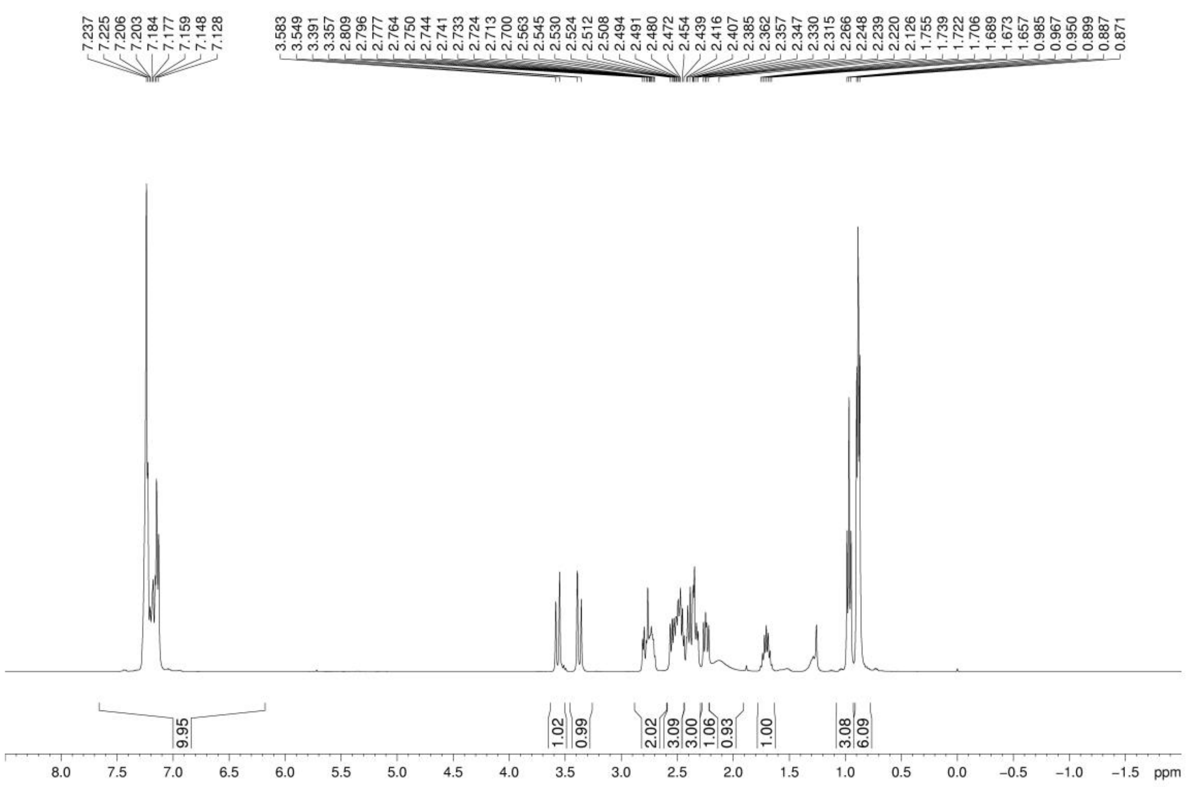

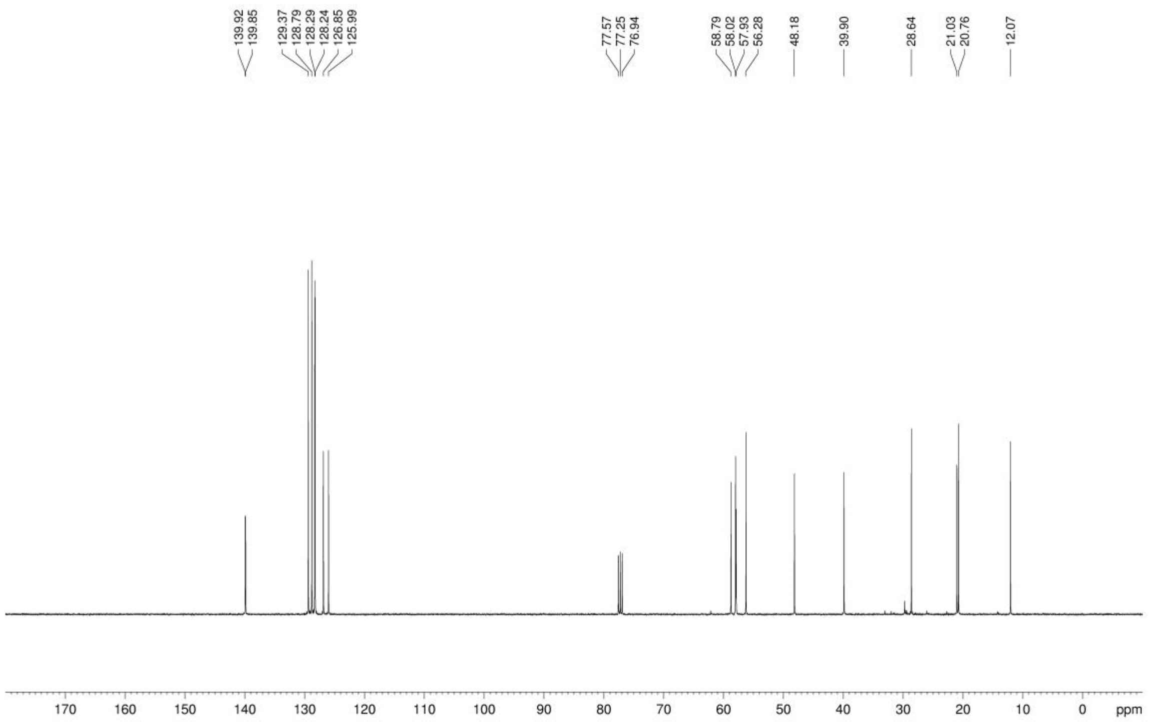

Supplementary Figure 17. ^{1}H and ^{13}C-NMR Spectrum for **If.**

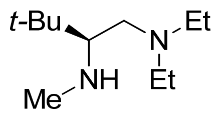

Ig

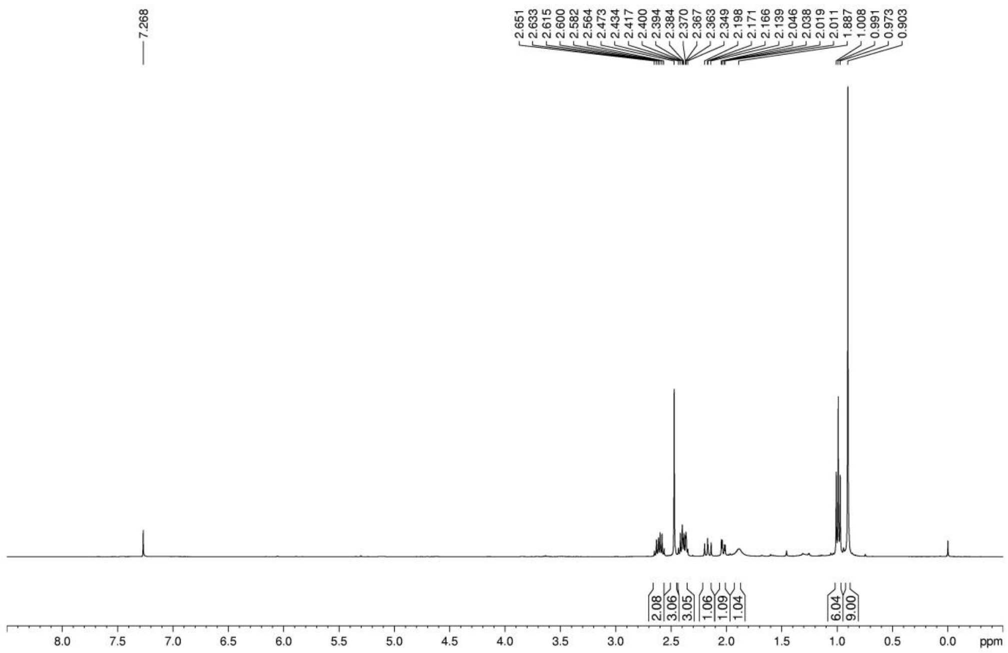

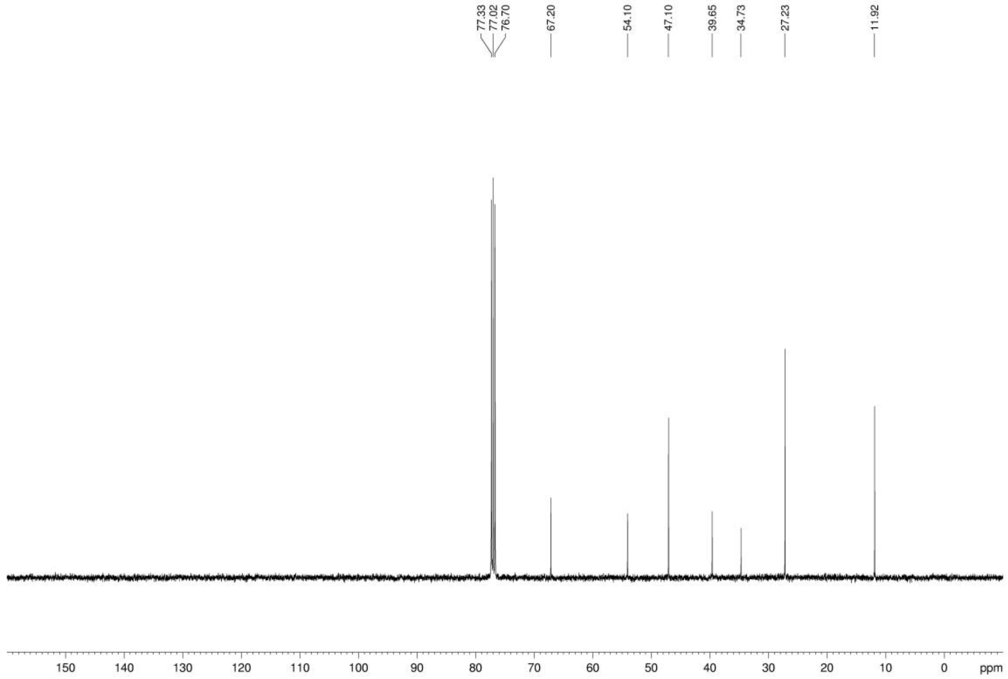

Supplementary Figure 18. ^{1}H and ^{13}C-NMR Spectrum for **Ig.**

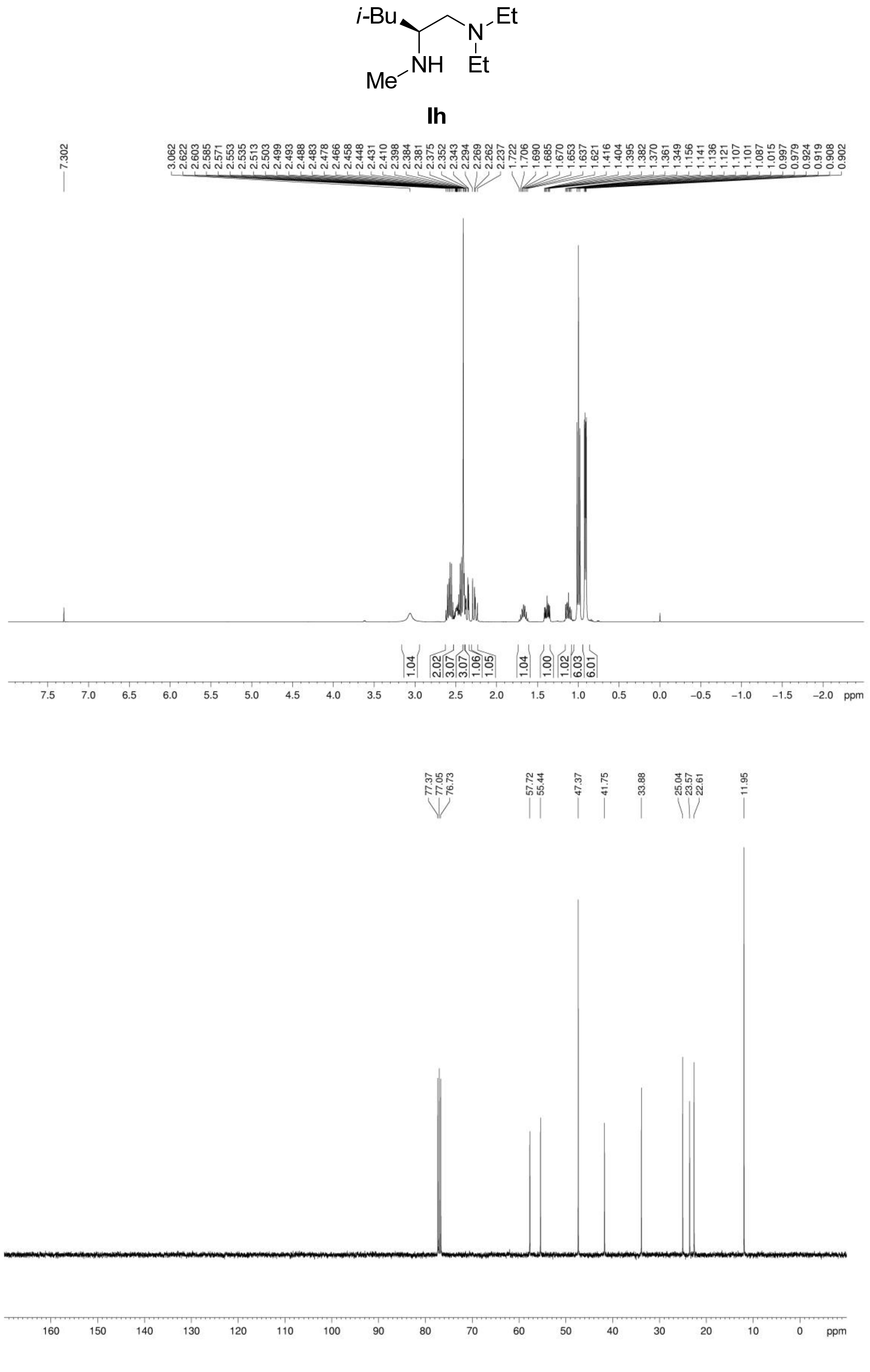

Supplementary Figure 19. ^{1}H and ^{13}C-NMR Spectrum for **Ih**

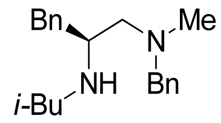

Ii

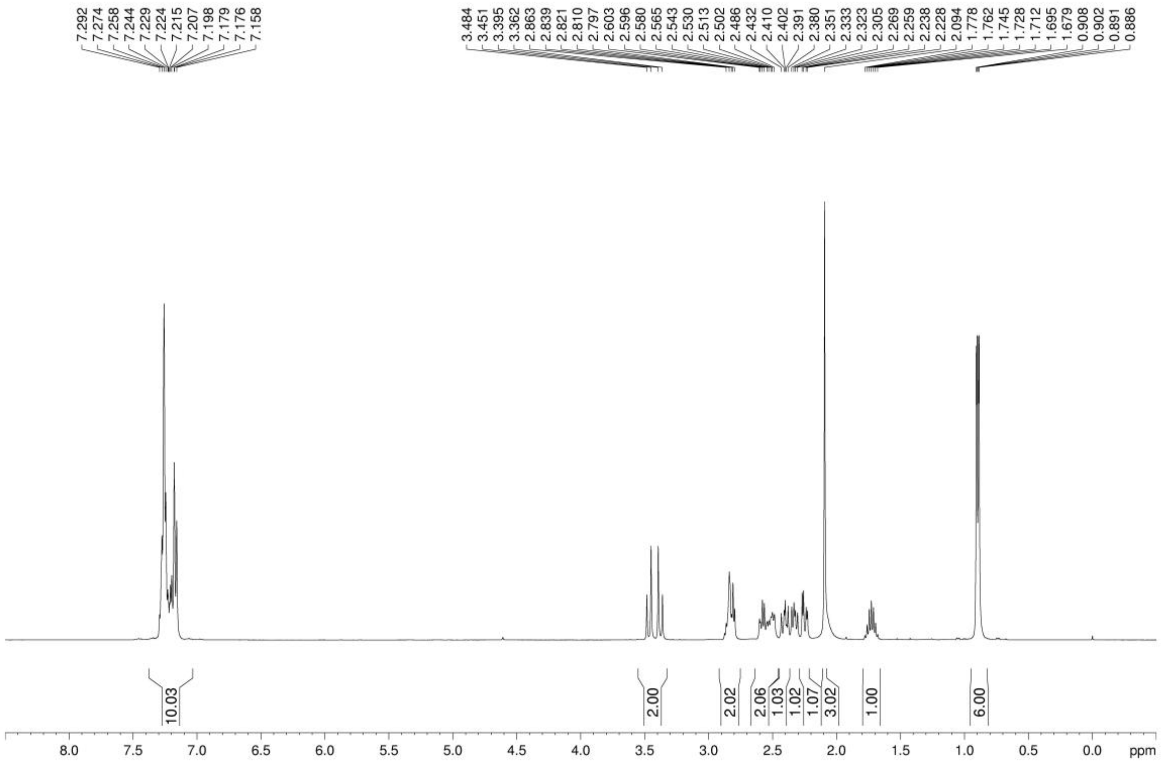

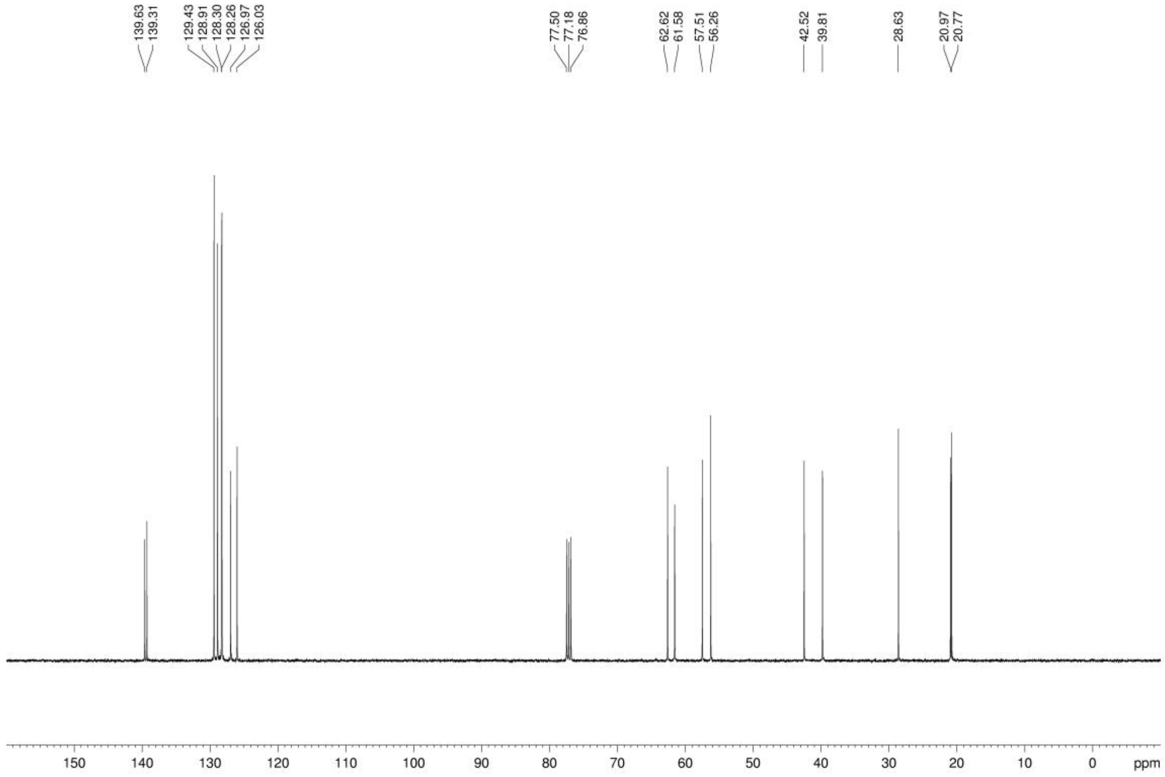

Supplementary Figure 20. ^{1}H and ^{13}C-NMR Spectrum for **Ii.**

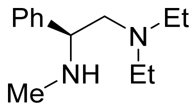

Ij

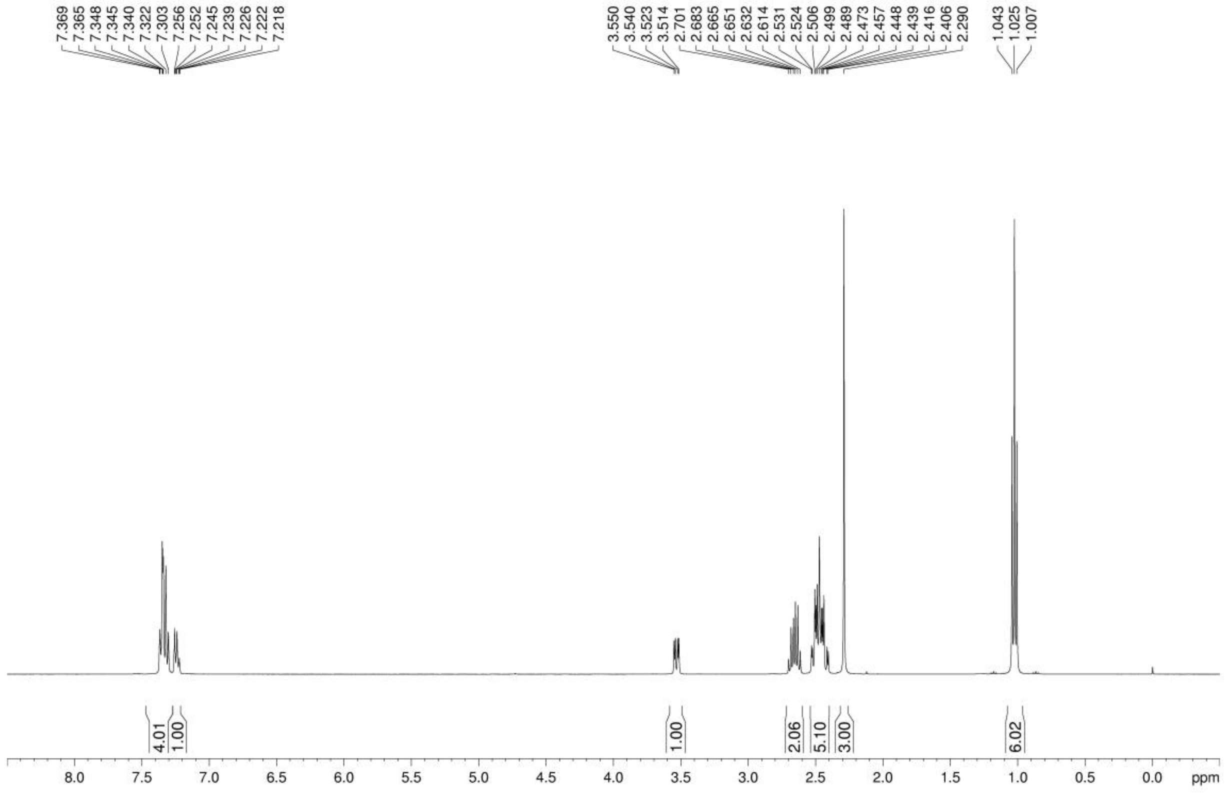

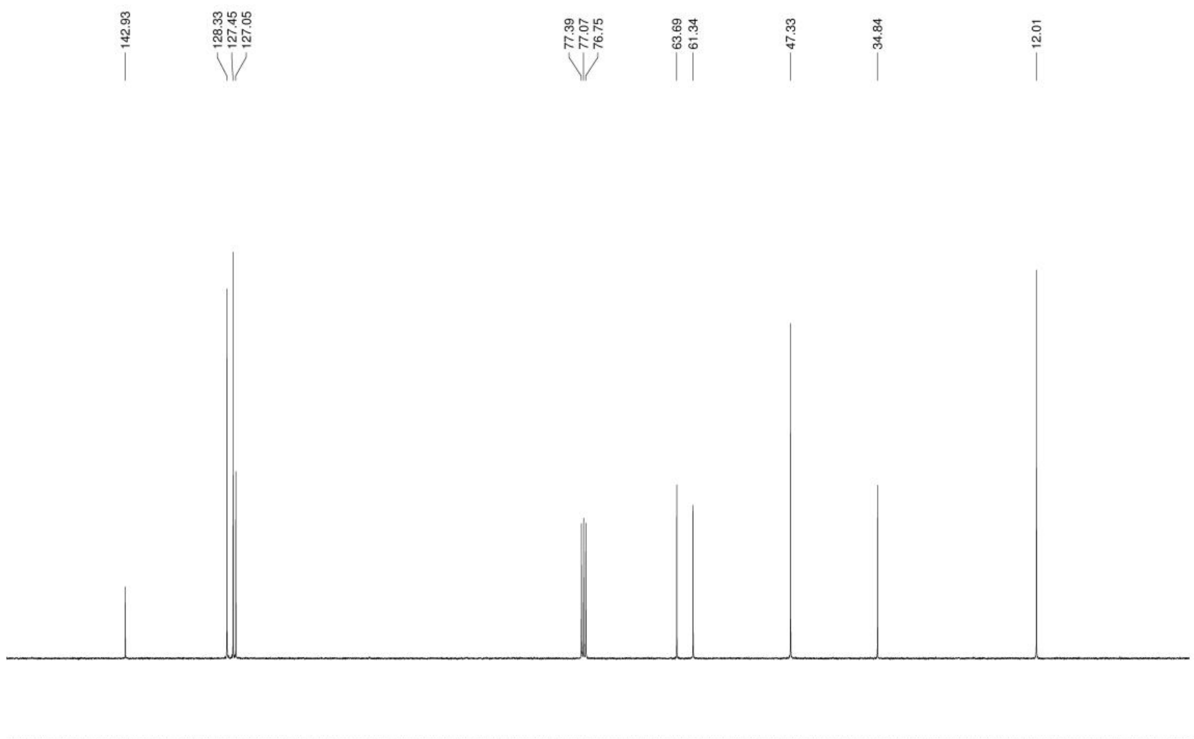

Supplementary Figure 21. ^{1}H and ^{13}C-NMR Spectrum for **Ij.**

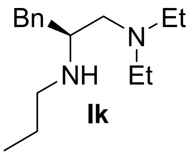

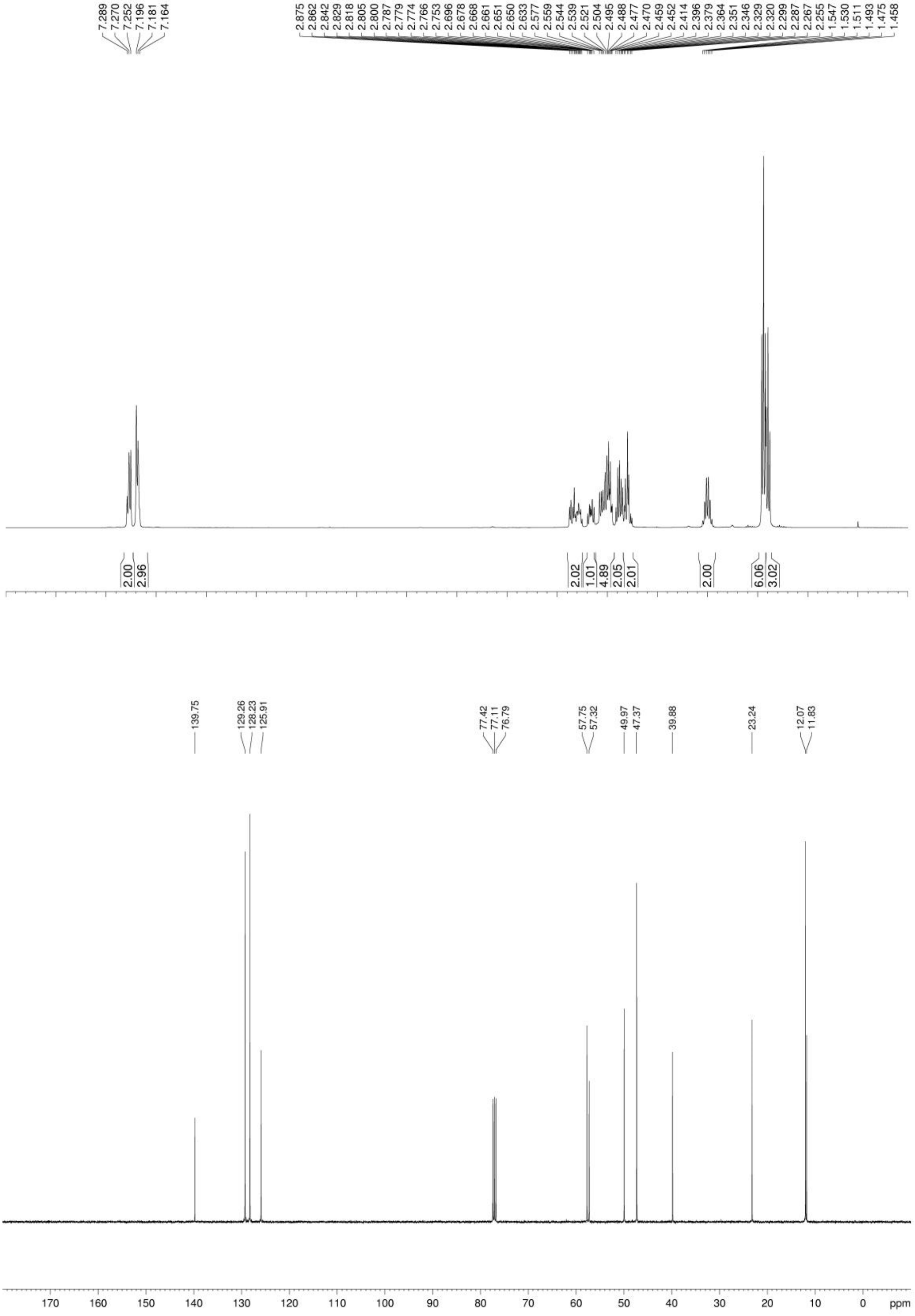

Supplementary Figure 22. ^{1}H and ^{13}C-NMR Spectrum for **Ik.**

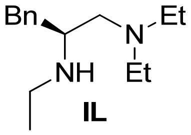

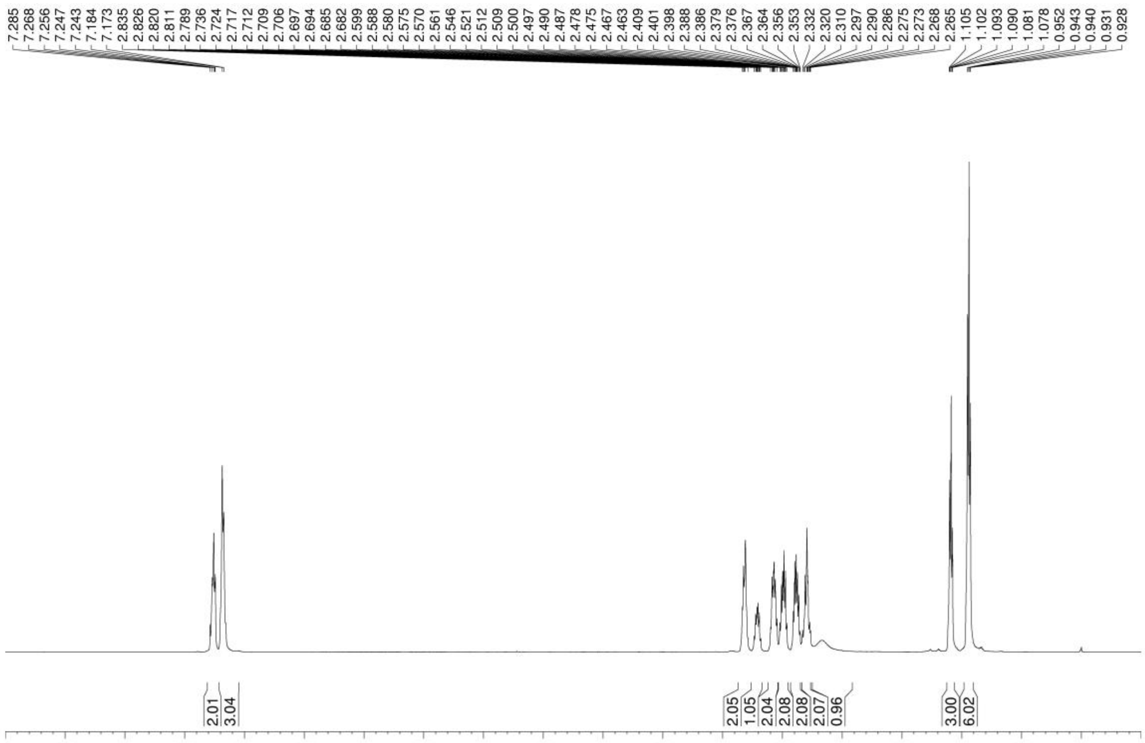

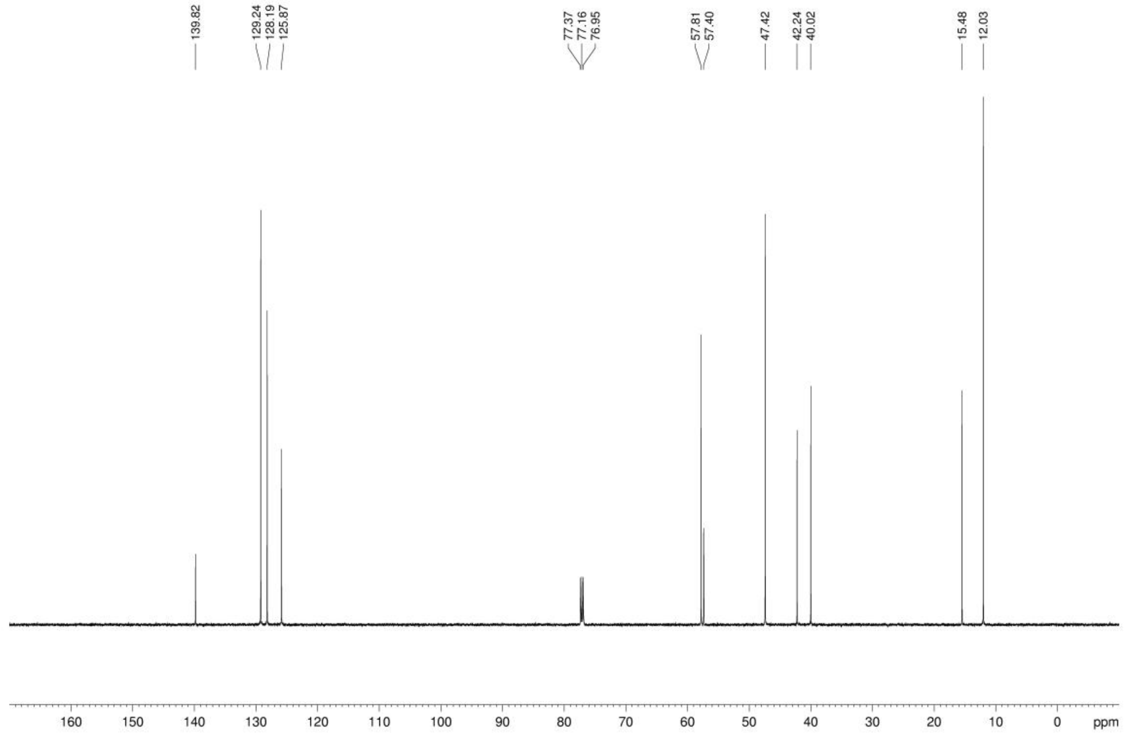

Supplementary Figure 23. ^{1}H and ^{13}C-NMR Spectrum for **IL.**

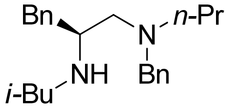

Im

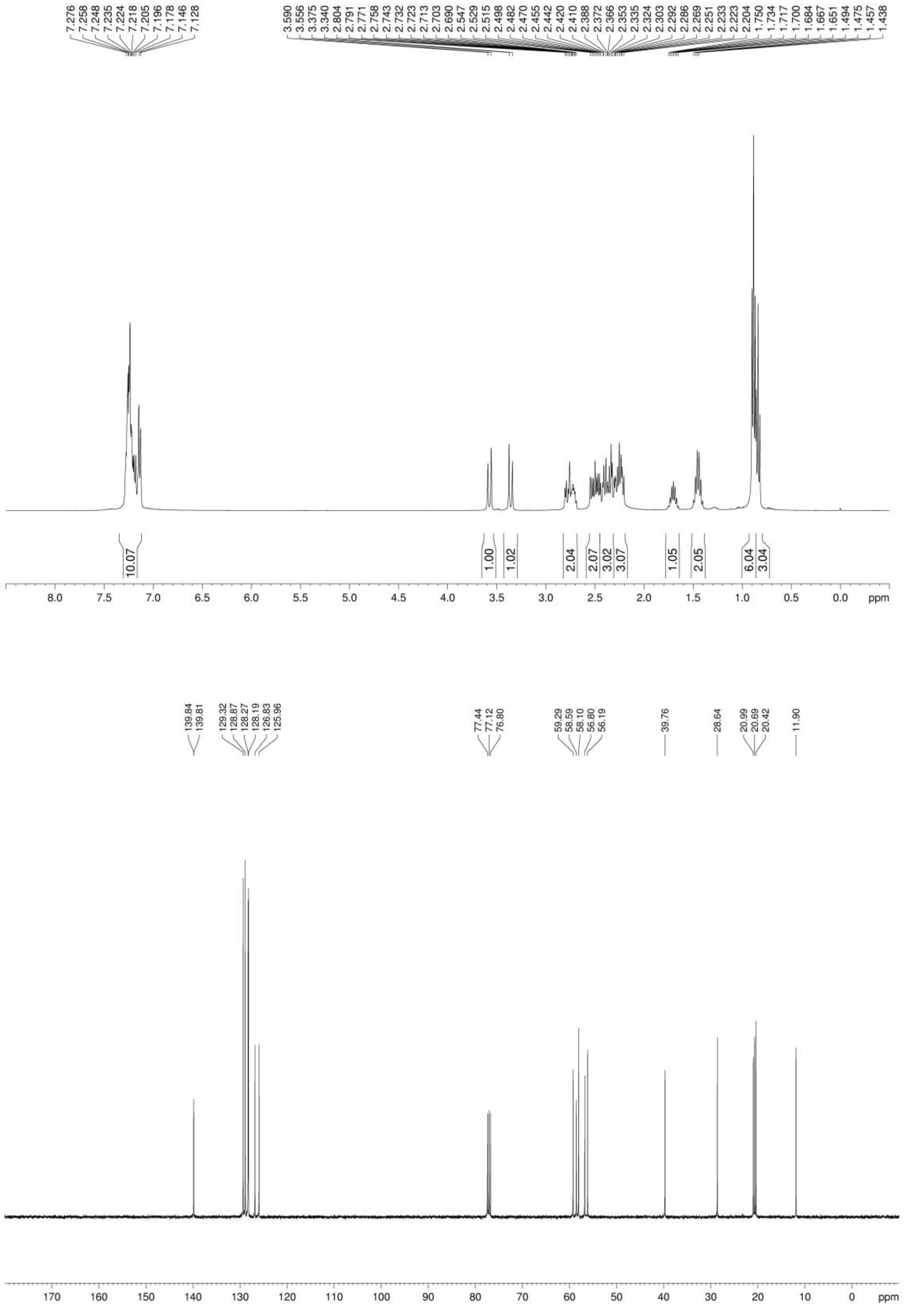

Supplementary Figure 24. ^{1}H and ^{13}C-NMR Spectrum for **Im.**

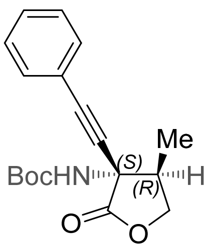

(*S*,*R*)-**3a**

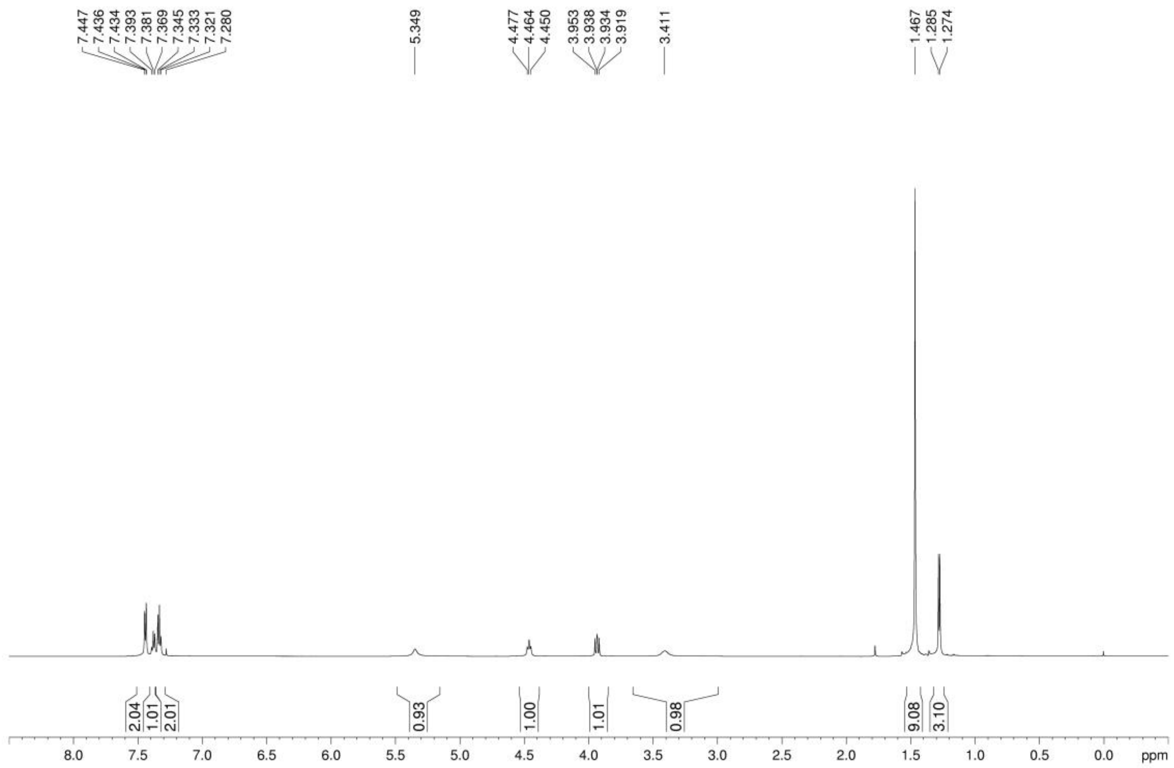

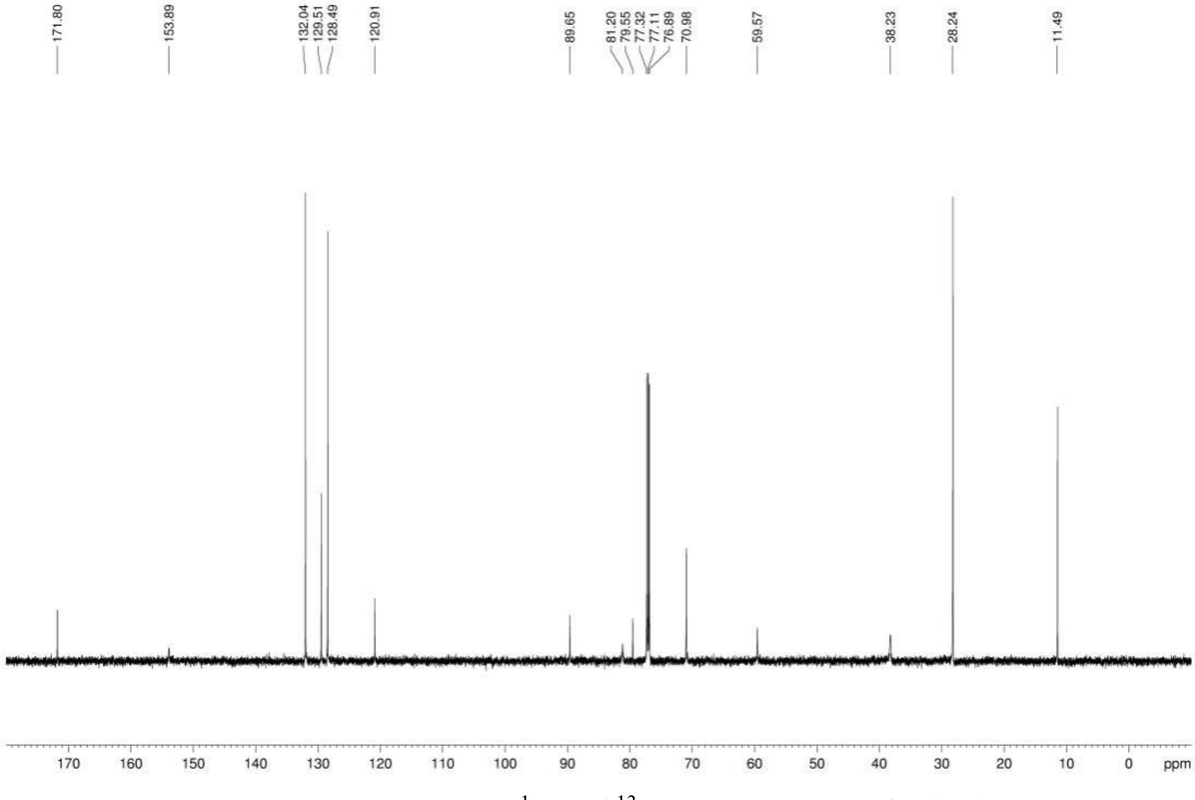

Supplementary Figure 25. ^{1}H and ^{13}C-NMR Spectrum for (*S*,*R*)-**3a.**

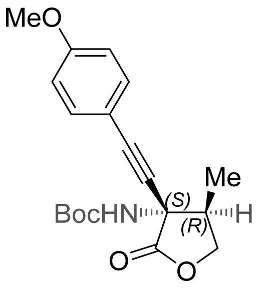

(*S*,*R*)-**3b**

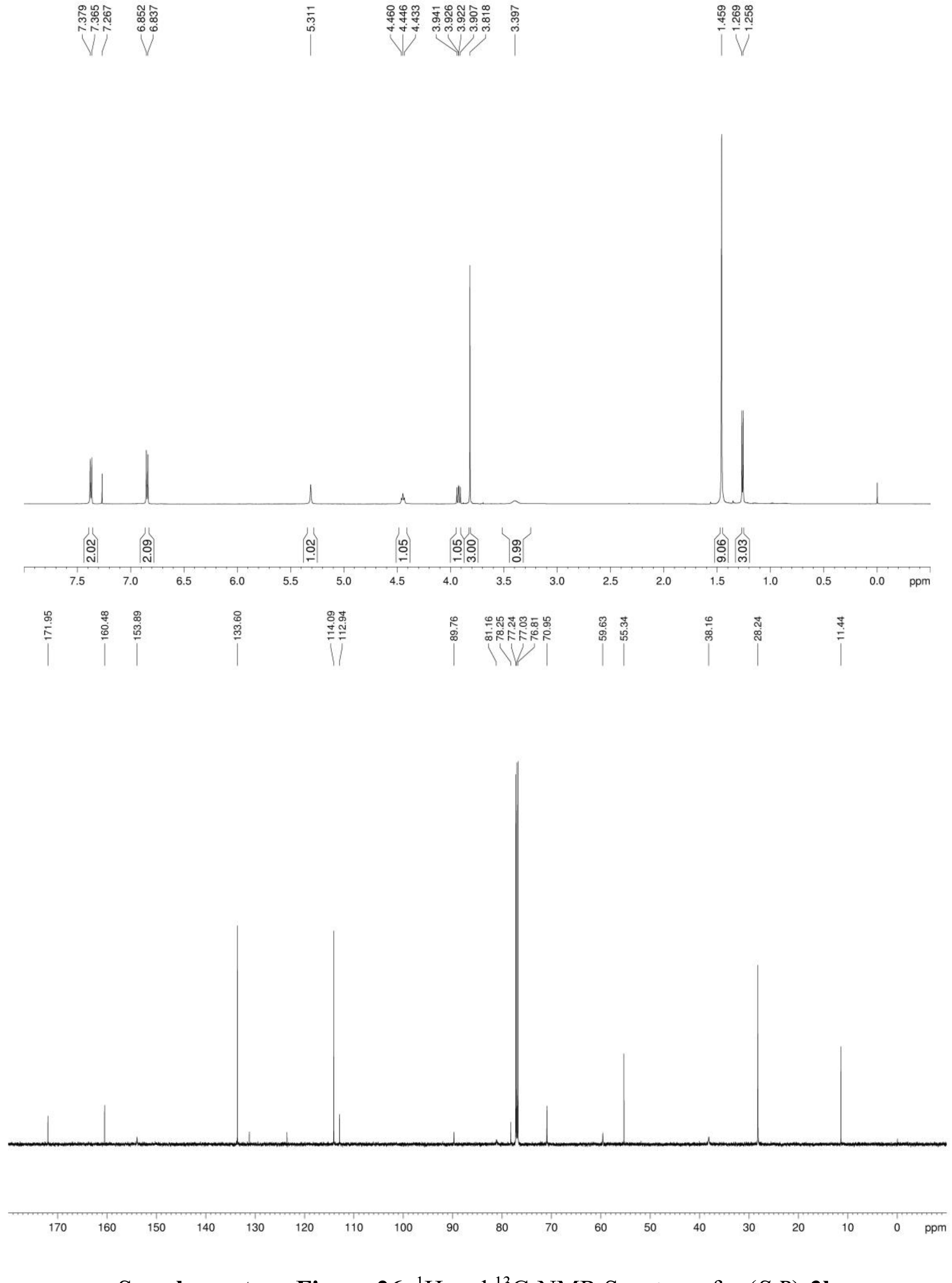

Supplementary Figure 26. ^{1}H and ^{13}C-NMR Spectrum for (*S*,*R*)-**3b**

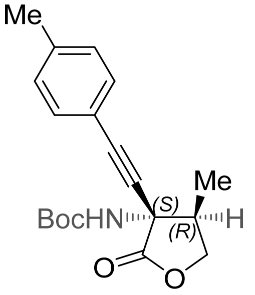

(*S*,*R*)-**3c**

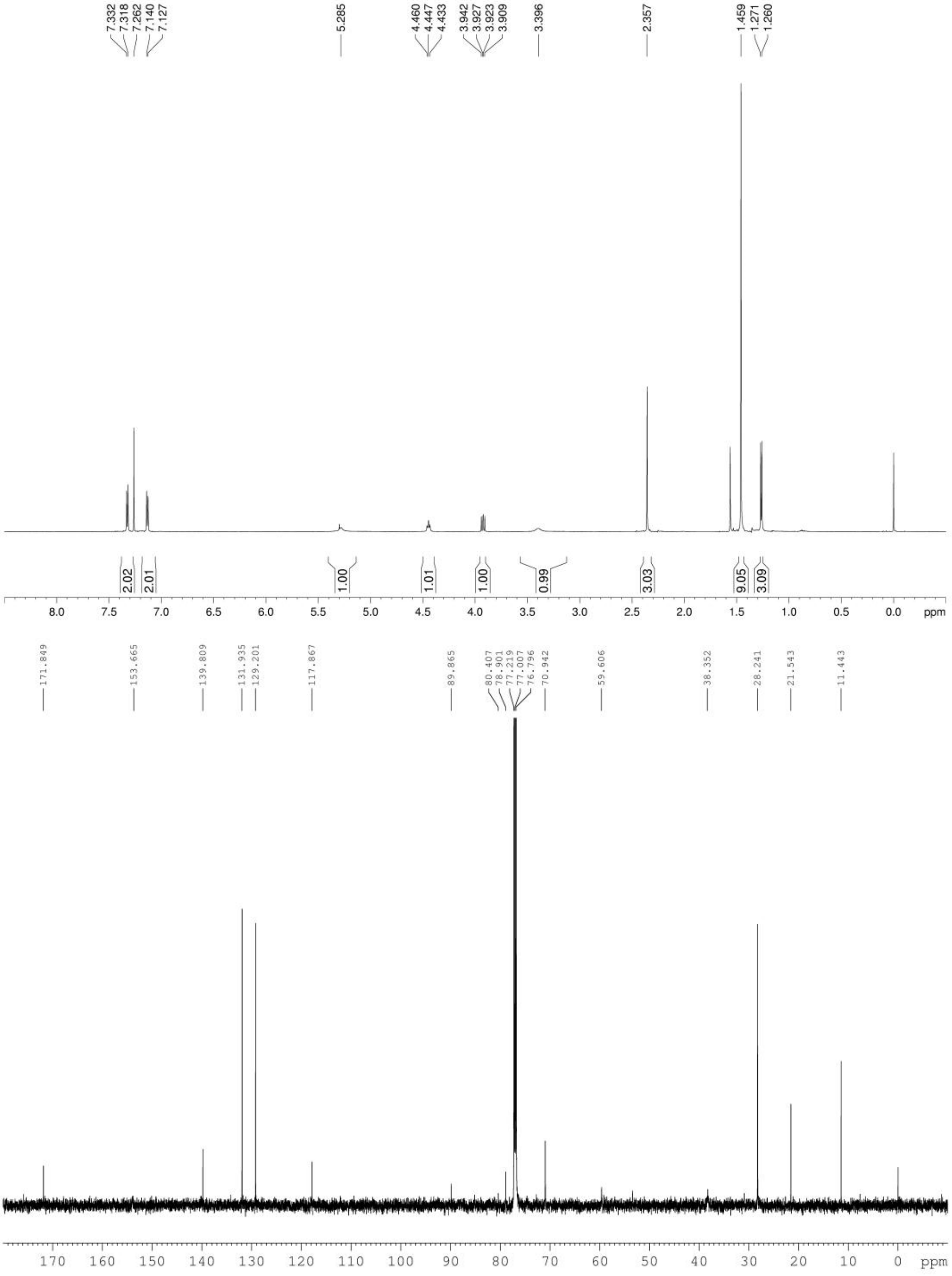

Supplementary Figure 27. ^{1}H and ^{13}C-NMR Spectrum for (*S,R*)-**3c.**

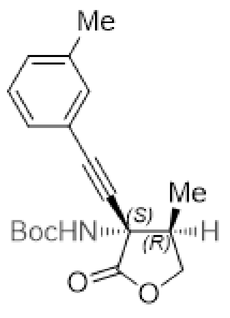

(*S*,*R*)-**3d**

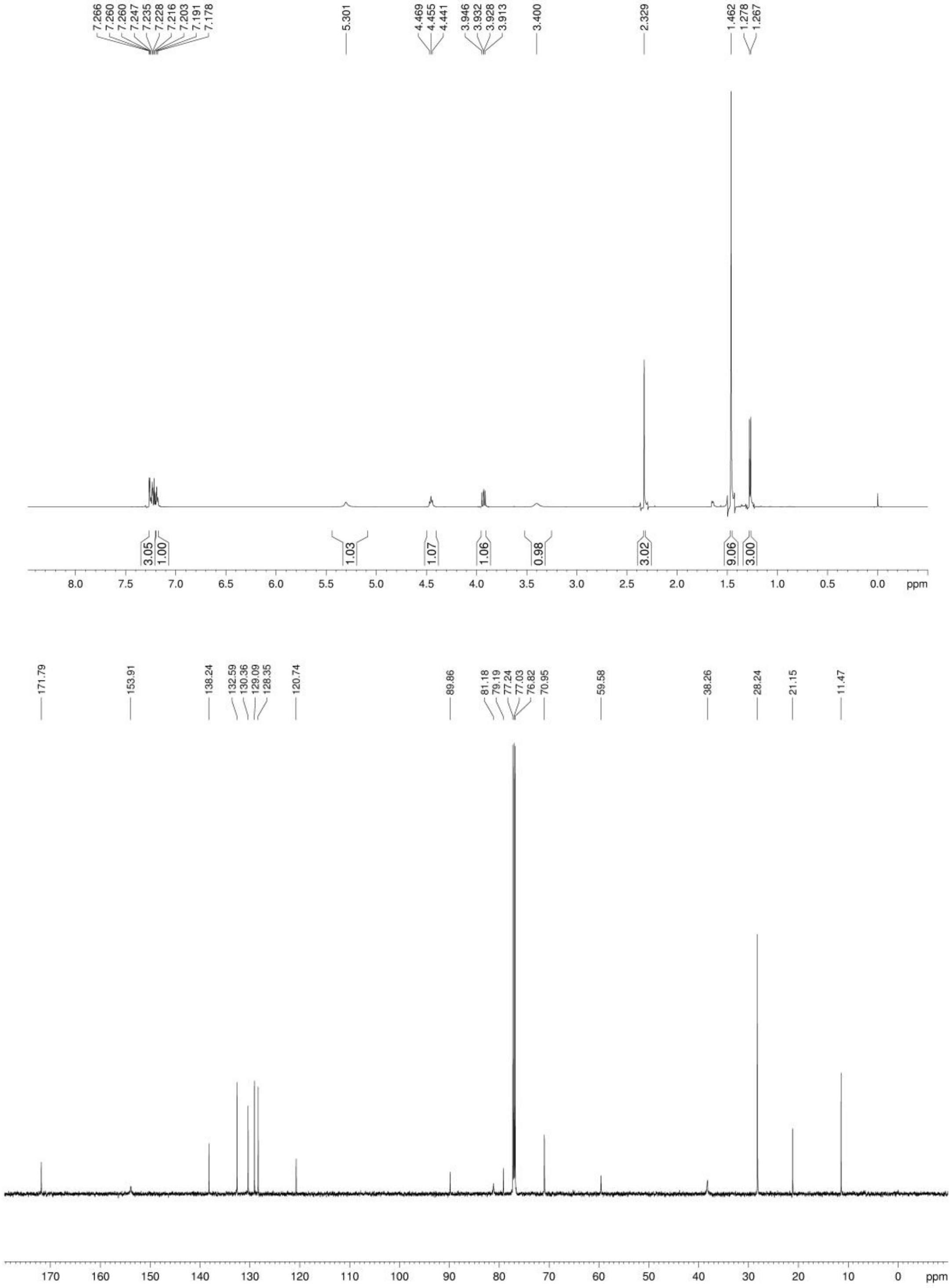

Supplementary Figure 28. ^{1}H and ^{13}C-NMR Spectrum for (*S*,*R*)-**3d.**

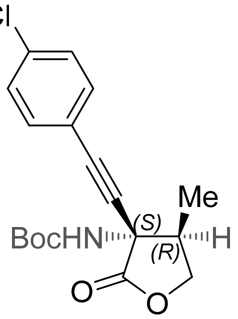

(*S*,*R*)-**3e**

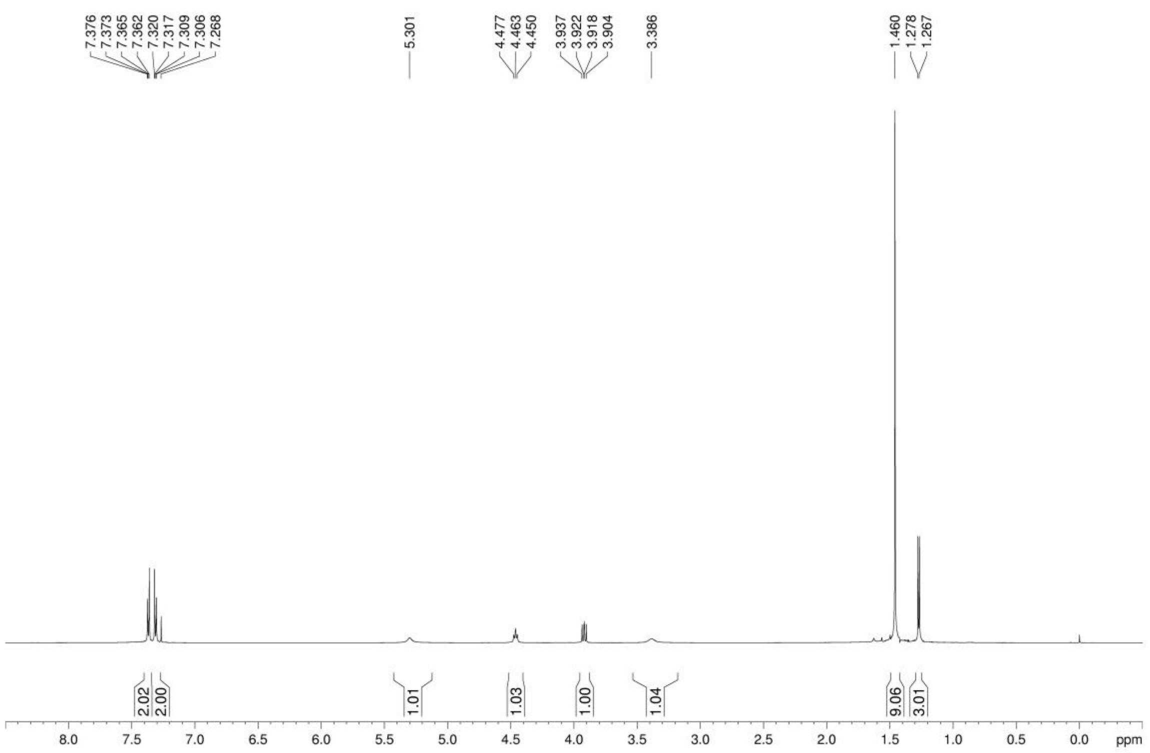

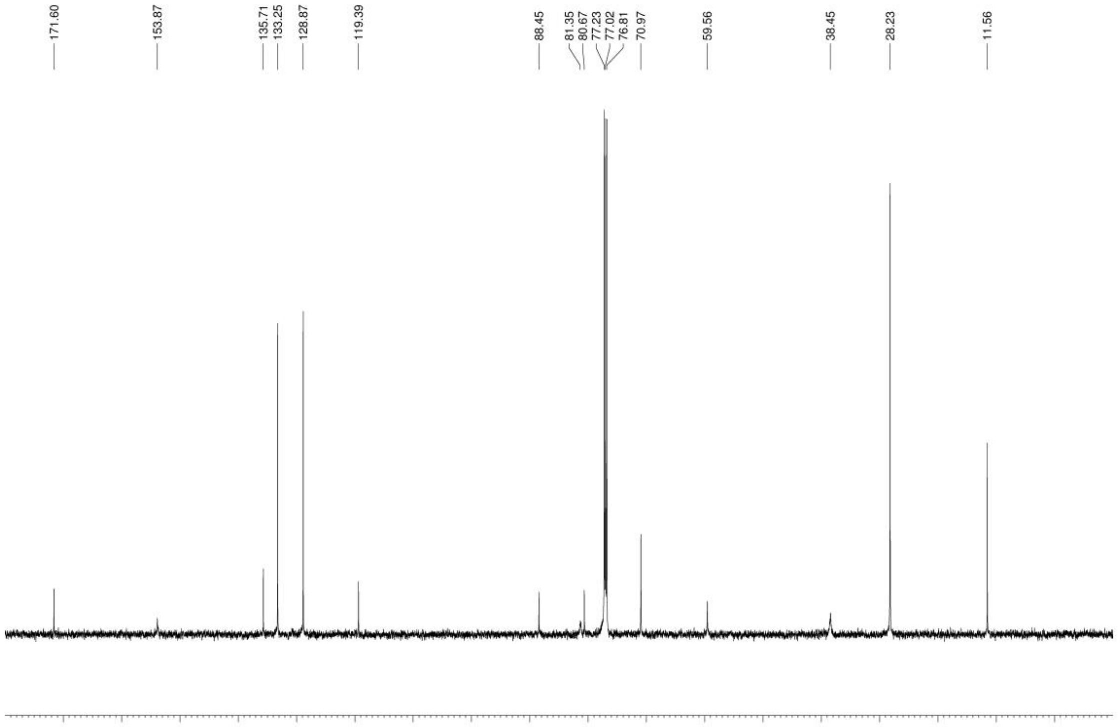

Supplementary Figure 29. ^{1}H and ^{13}C-NMR Spectrum for (*S,R*)-**3e.**

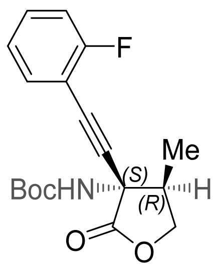

(*S*,*R*)-**3f**

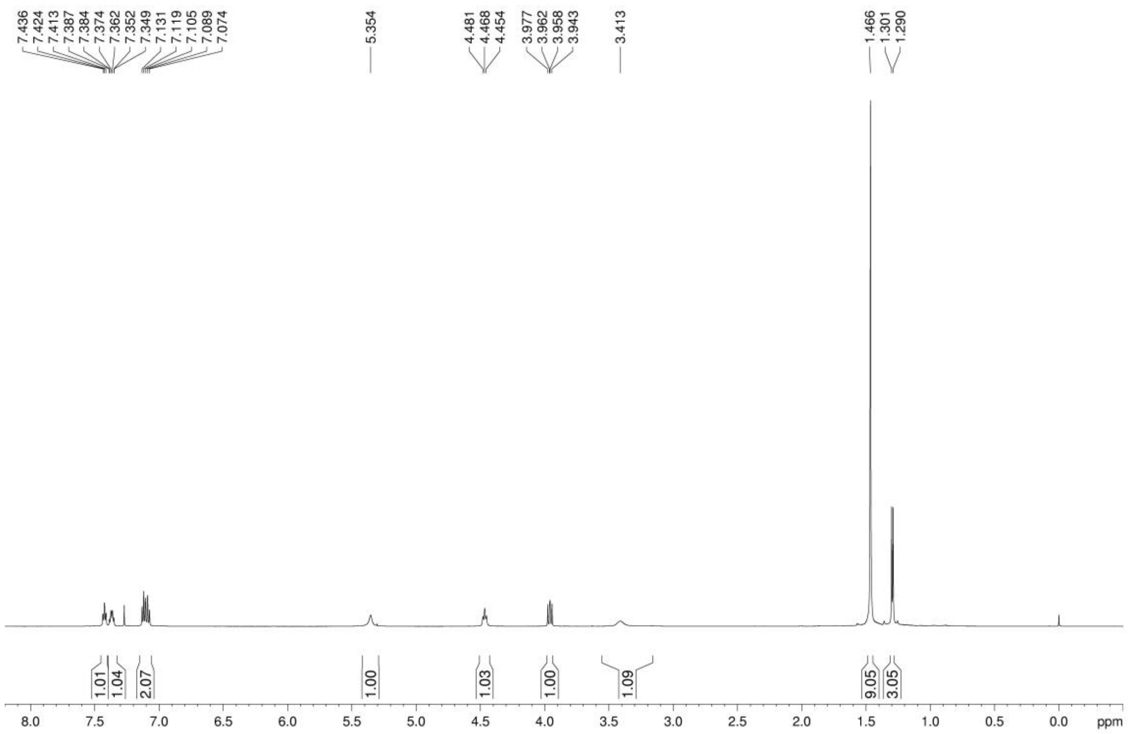

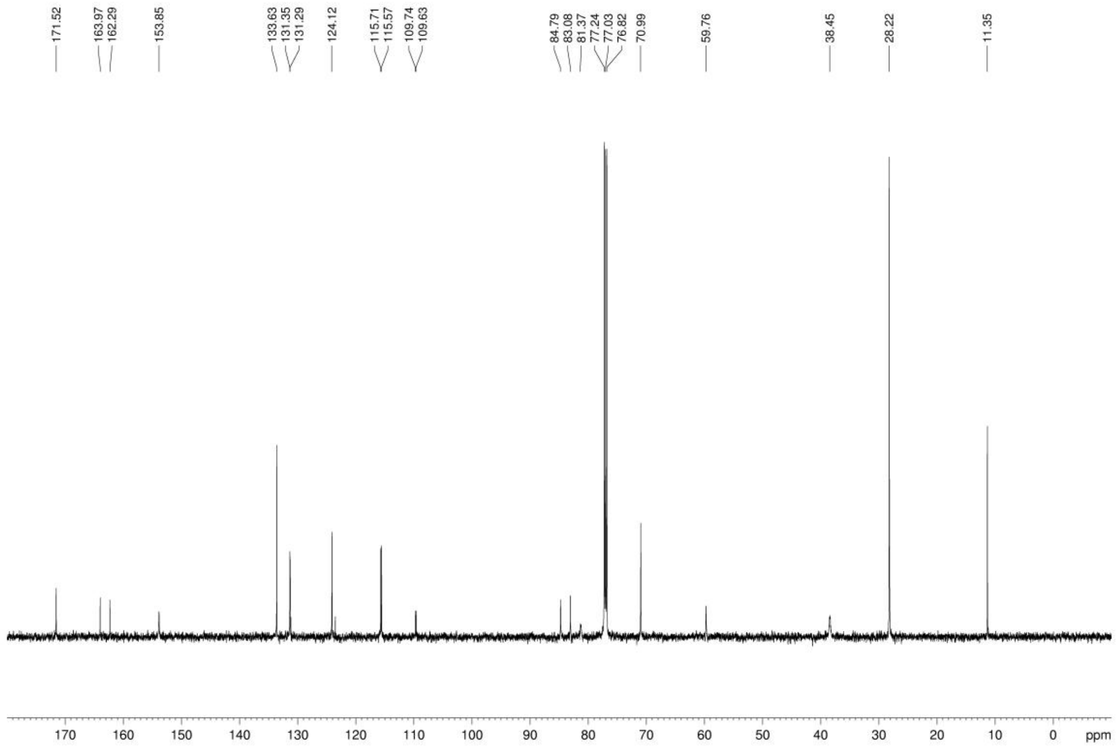

Supplementary Figure 30. ^{1}H and ^{13}C-NMR Spectrum for (*S,R*)-**3f.**

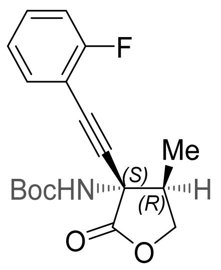

(*S*,*R*)-**3f**

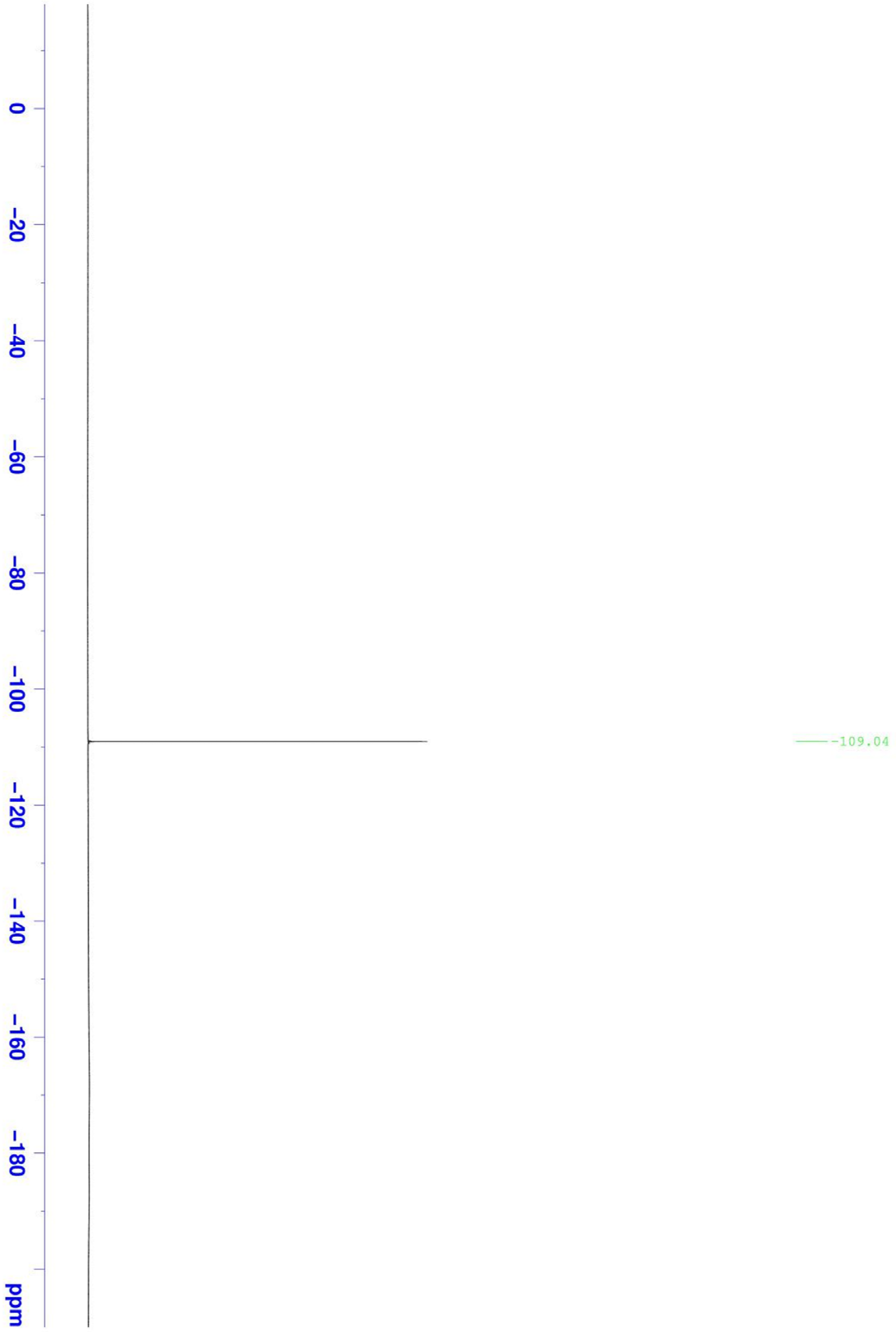

Supplementary Figure 31. ^{19}F-NMR Spectrum for (*S*,*R*)-**3f.**

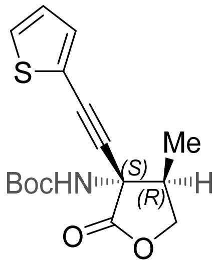

(*S*,*R*)-**3g**

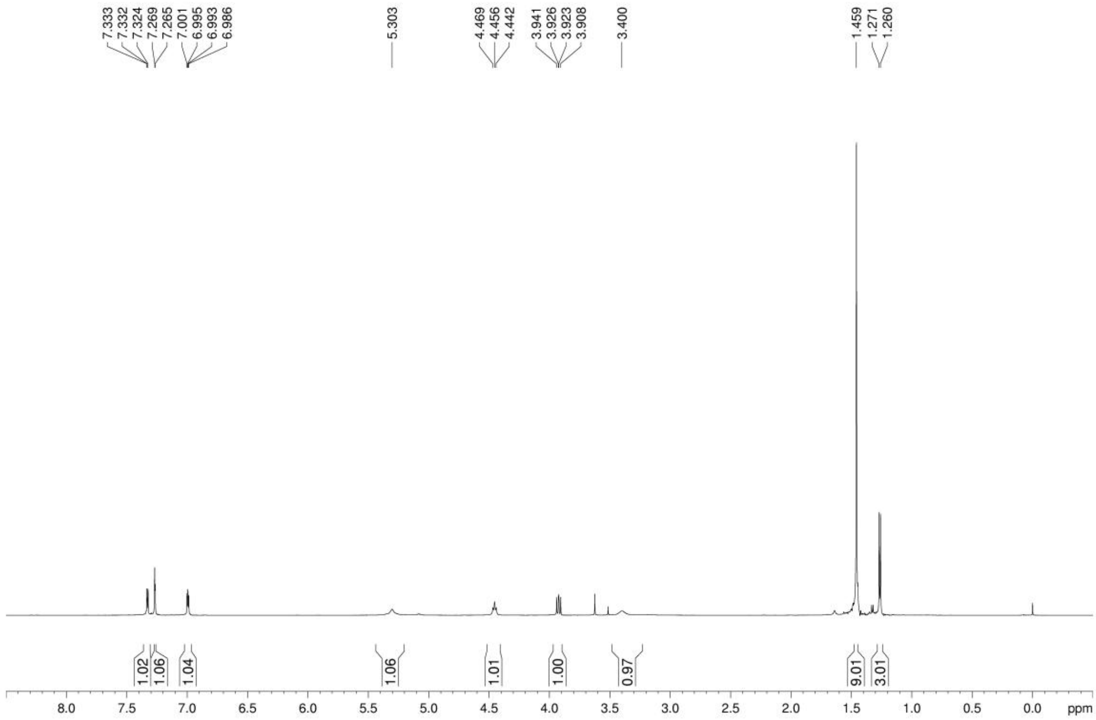

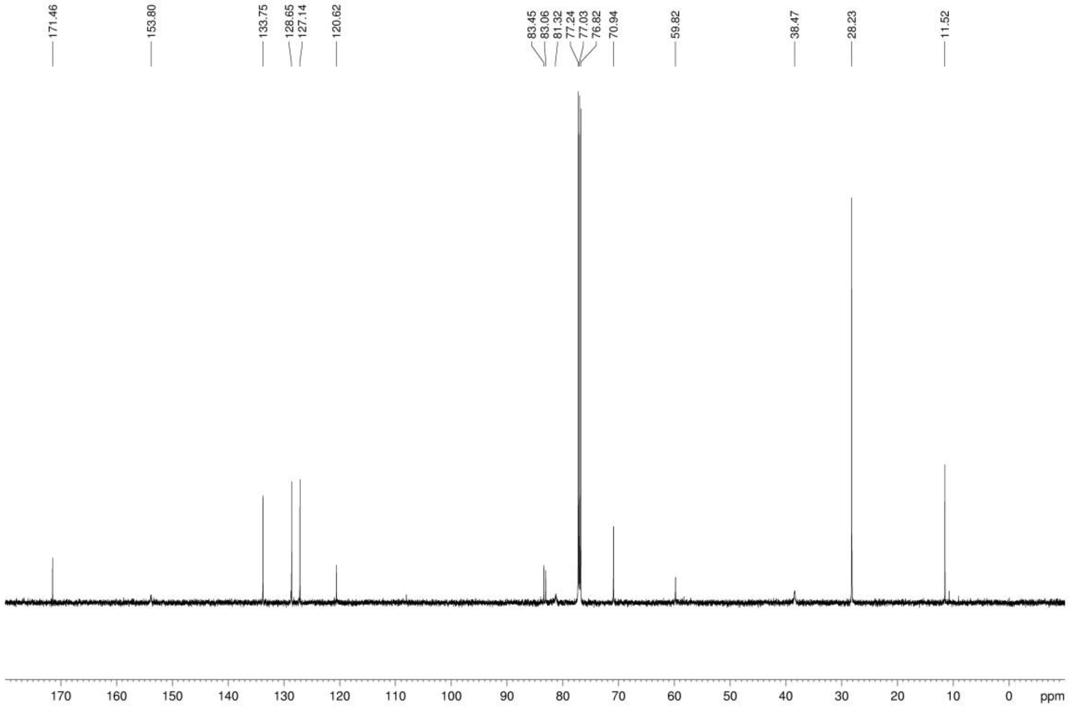

Supplementary Figure 32. ^{1}H and ^{13}C-NMR Spectrum for (*S,R*)-**3g.**

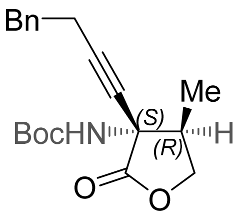

(*S*,*R*)-**3h**

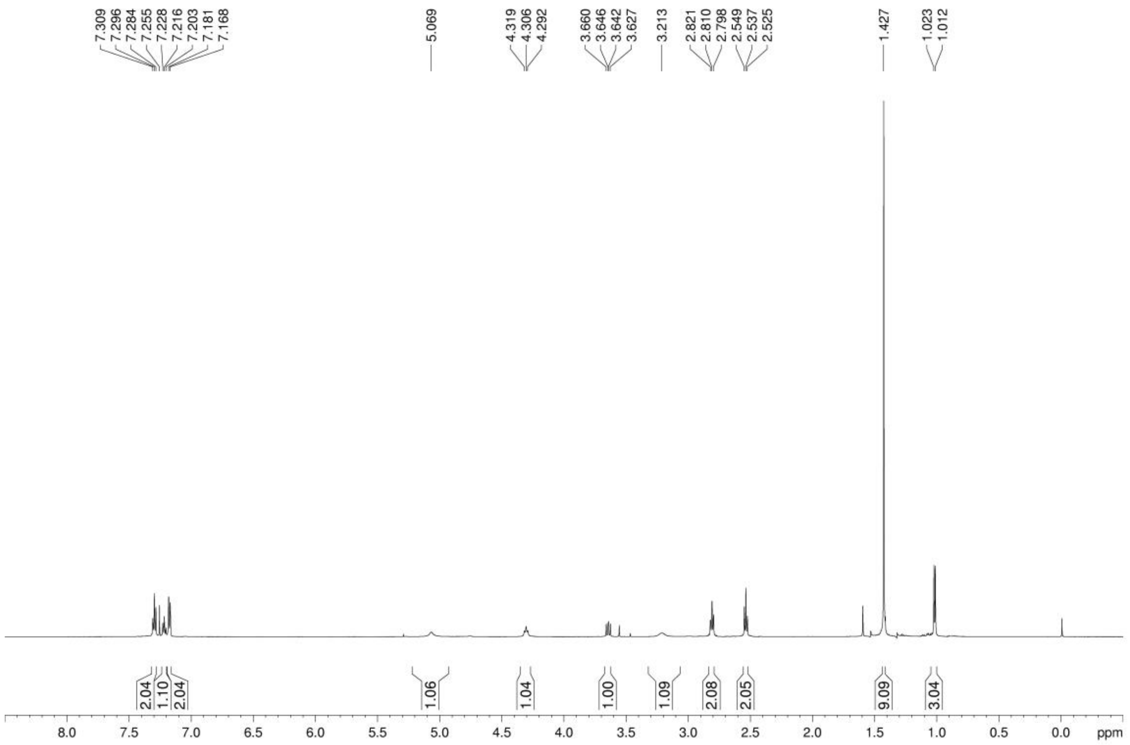

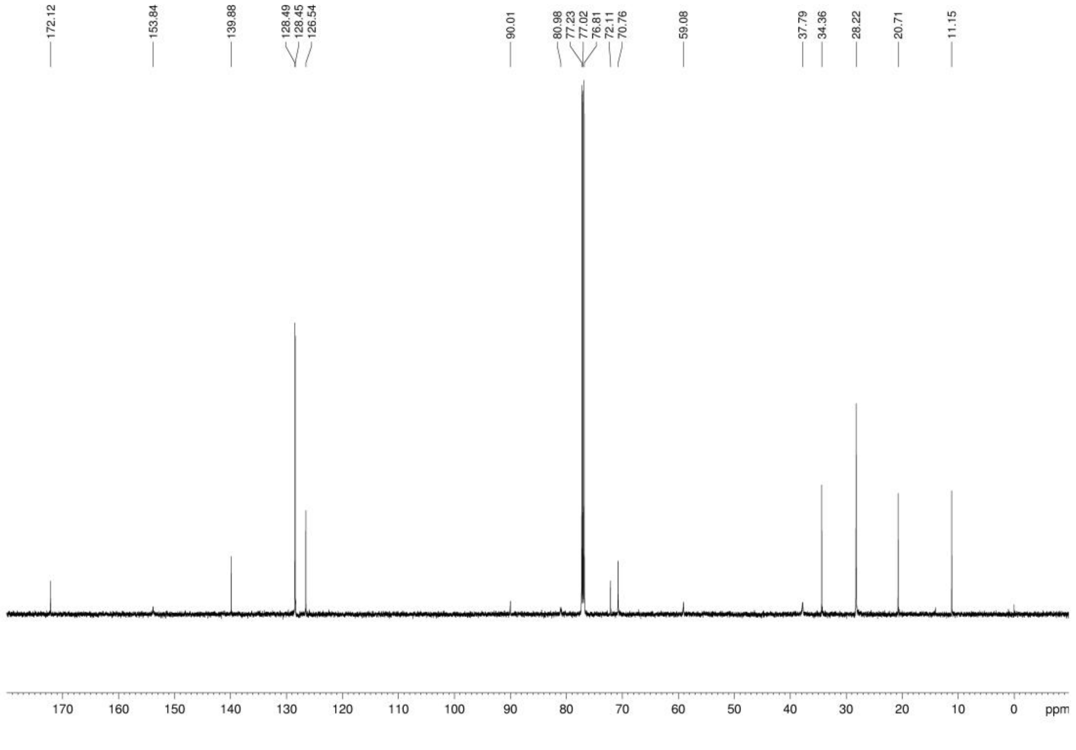

Supplementary Figure 33. ^{1}H and ^{13}C-NMR Spectrum for (*S*,*R*)-**3h.**

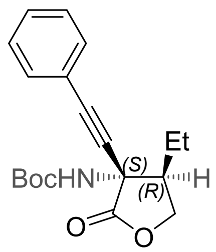

(*S*,*R*)-**3i**

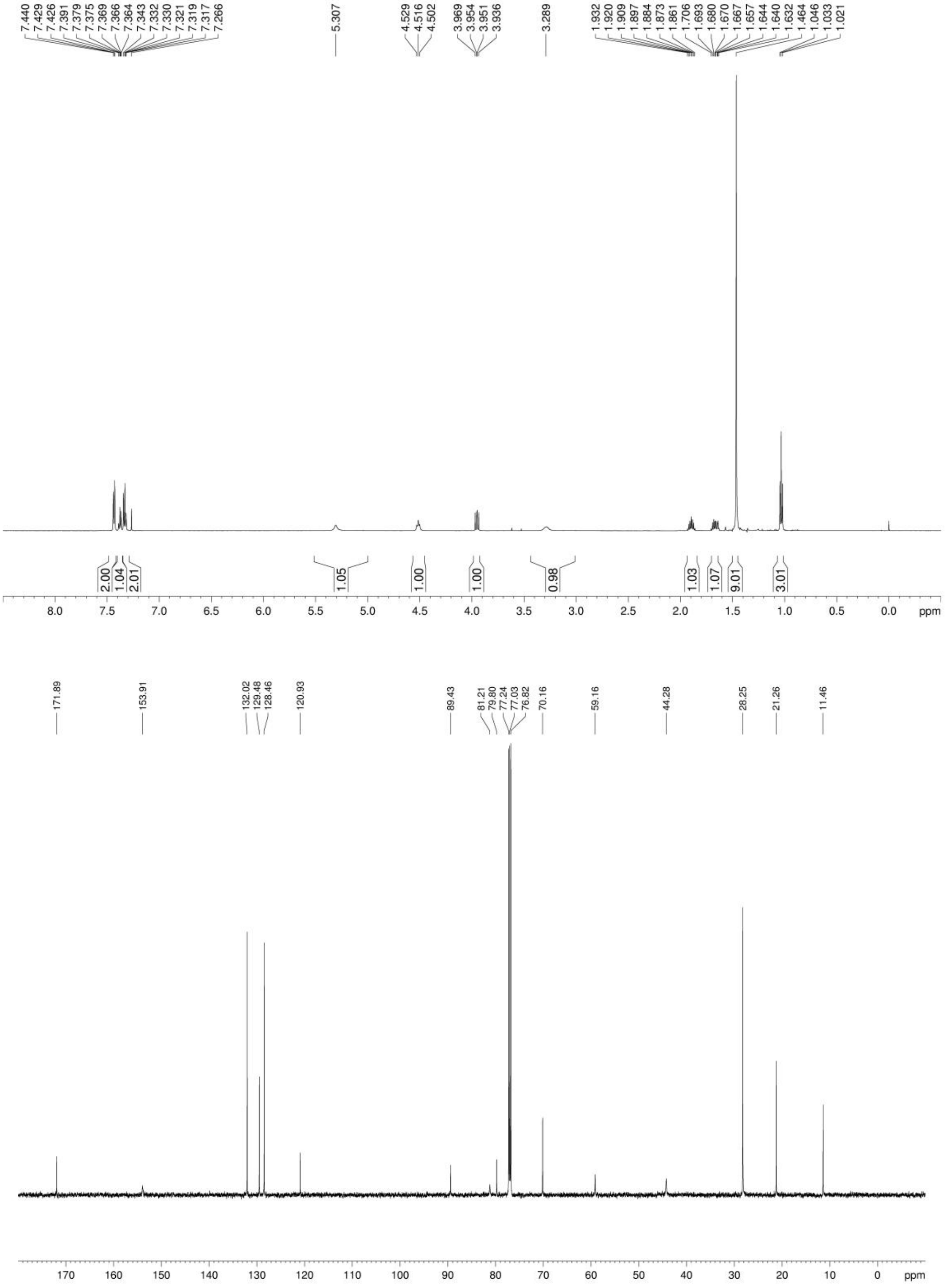

Supplementary Figure 34. ^{1}H and ^{13}C-NMR Spectrum for (*S,R*)-**3i.**

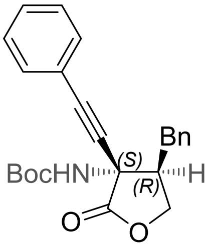

(*S*,*R*)-**3j**

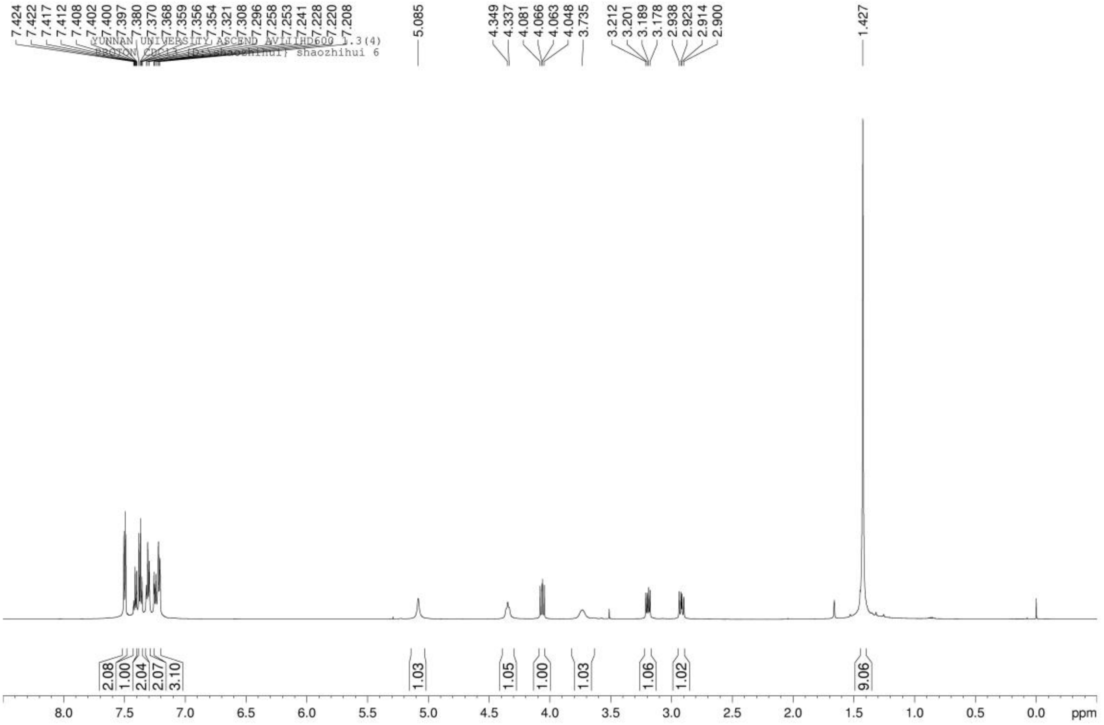

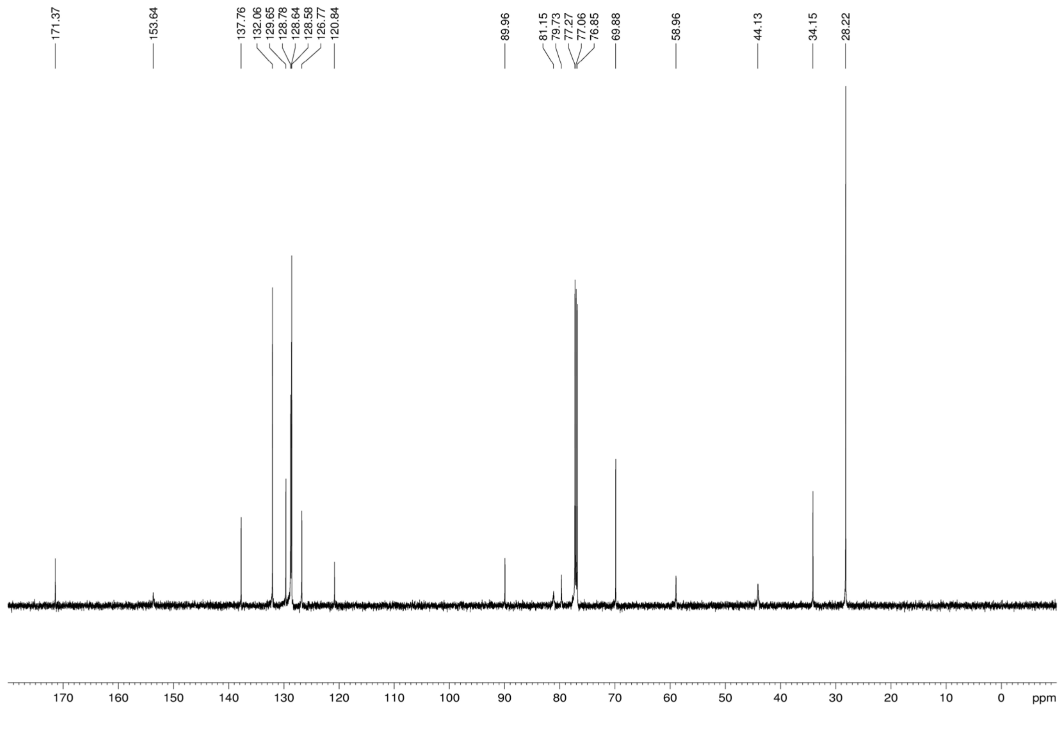

Supplementary Figure 35. ^{1}H and ^{13}C-NMR Spectrum for (*S*,*R*)-**3j.**

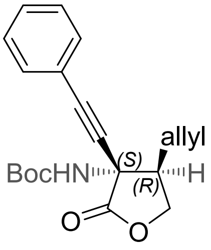

(*S*,*R*)-**3k**

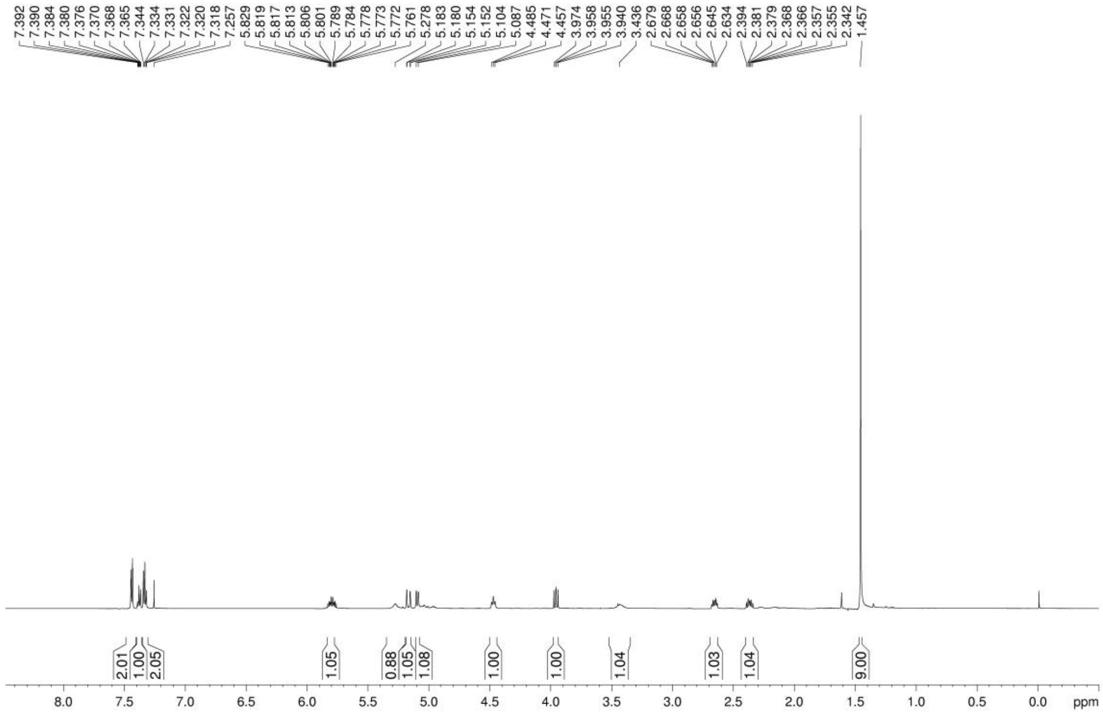

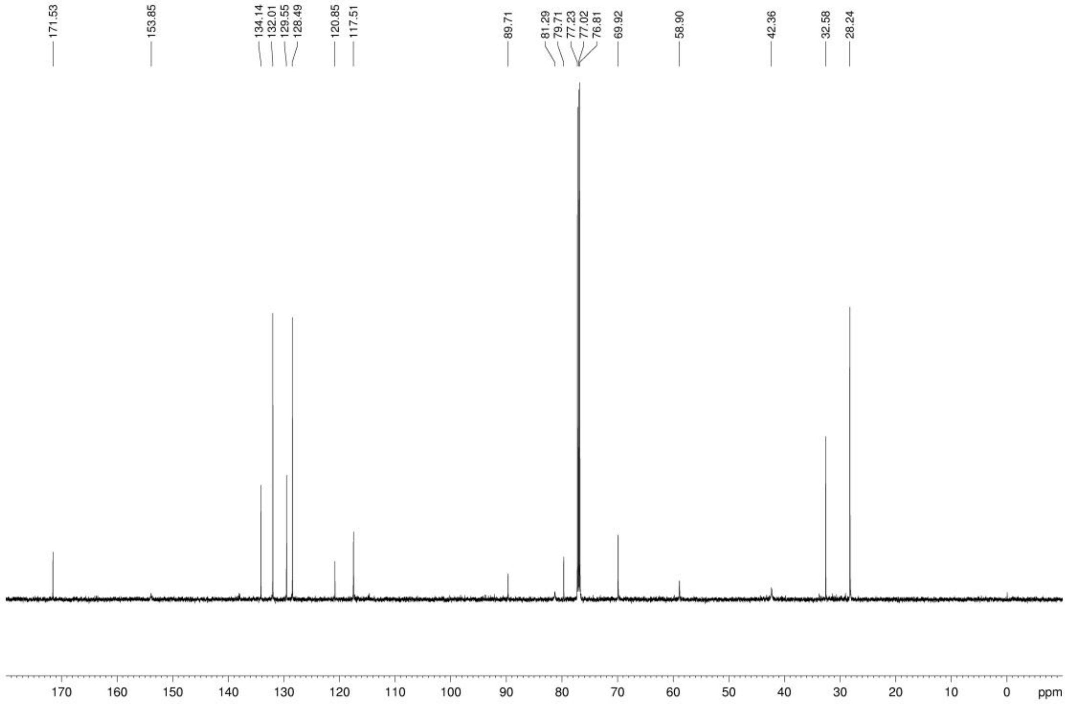

Supplementary Figure 36. ^{1}H and ^{13}C-NMR Spectrum for (*S,R*)-**3k.**

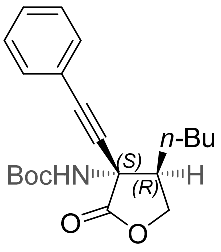

(*S*,*R*)-**3l**

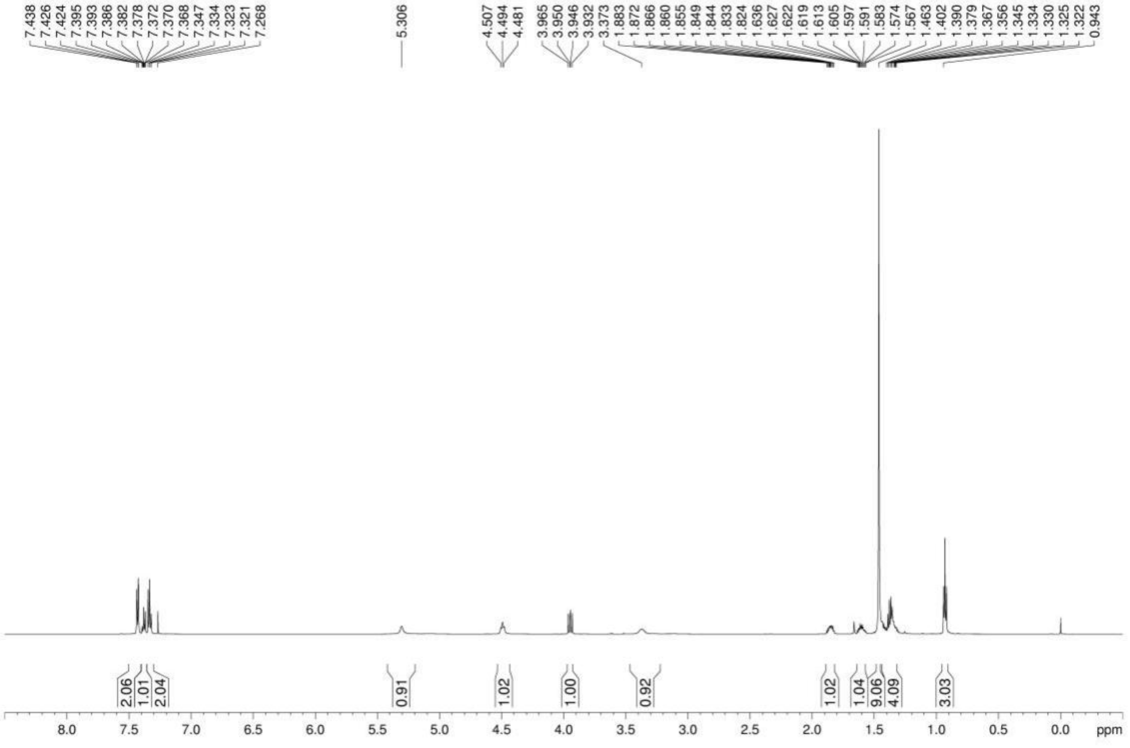

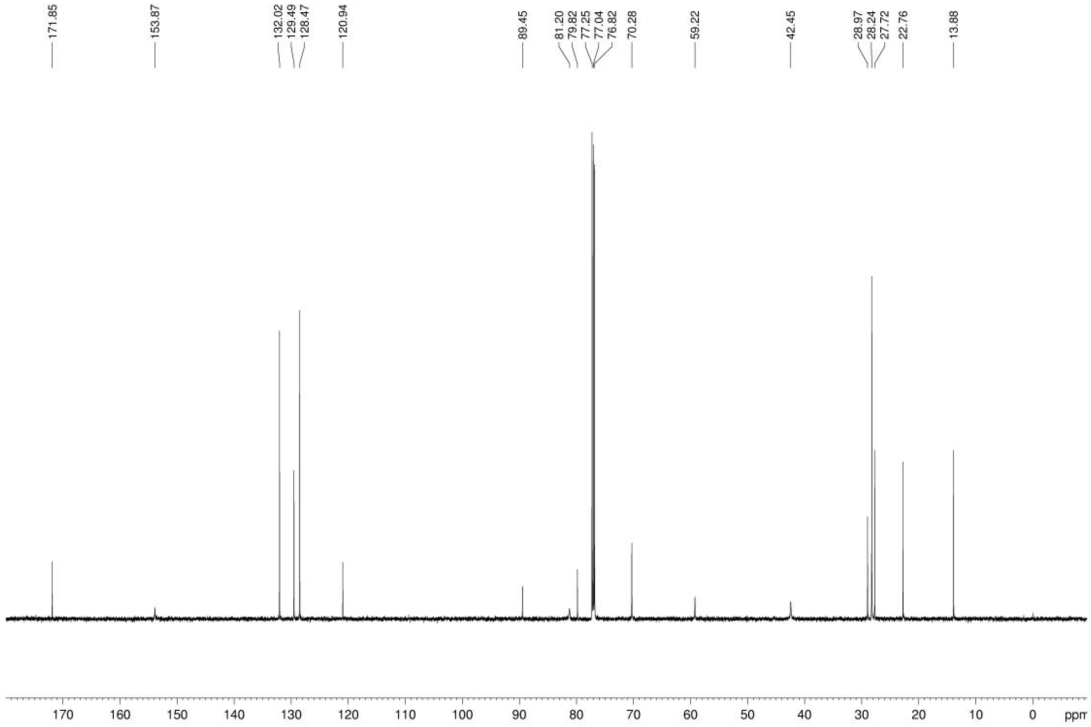

Supplementary Figure 37. ^{1}H and ^{13}C-NMR Spectrum for (*S*,*R*)-**3l.**

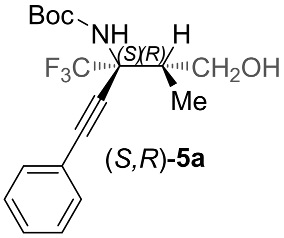

(*S*,*R*)-**5a**

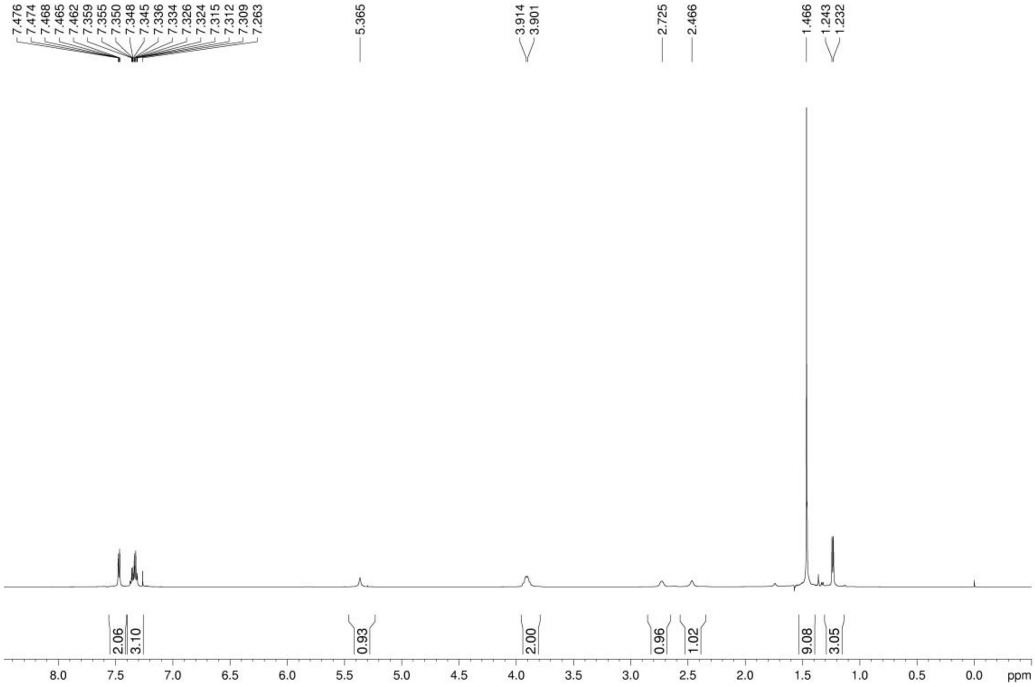

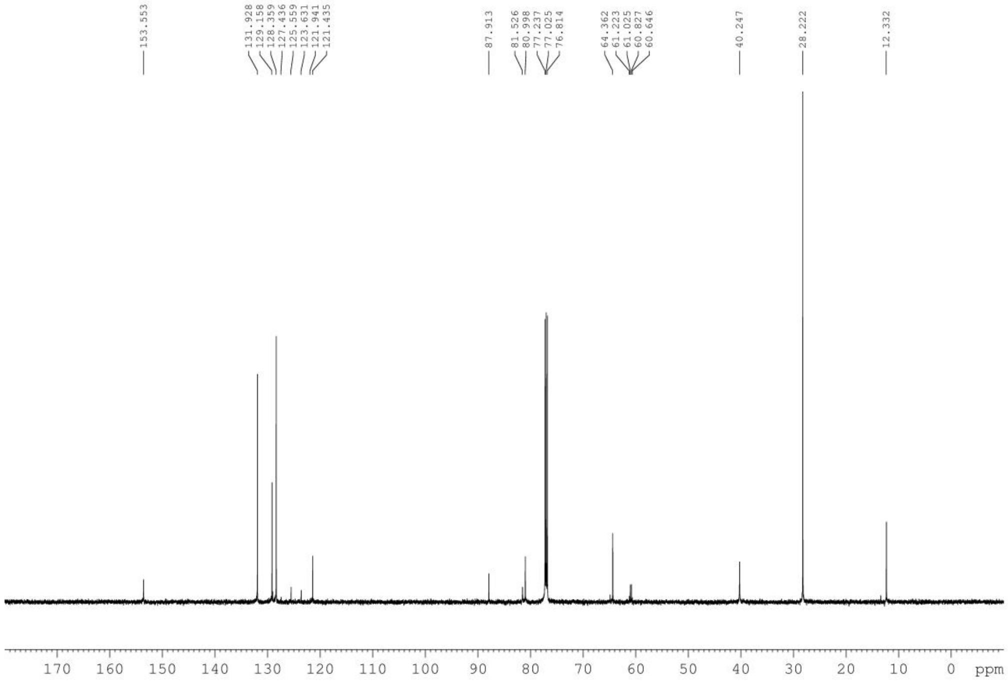

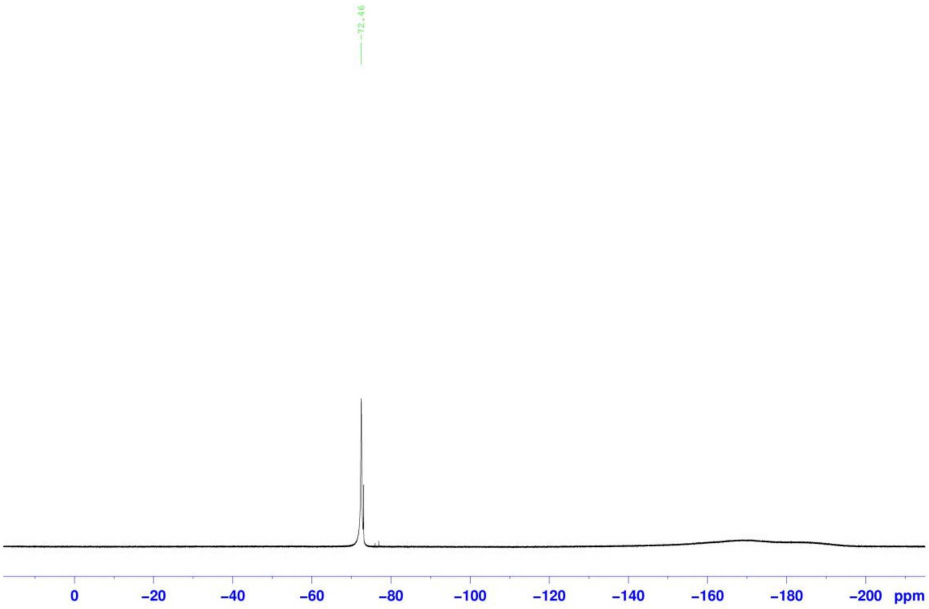

Supplementary Figure 38. ^{1}H, ^{13}C and ^{19}F-NMR Spectrum for (*S*,*R*)-**5a.**

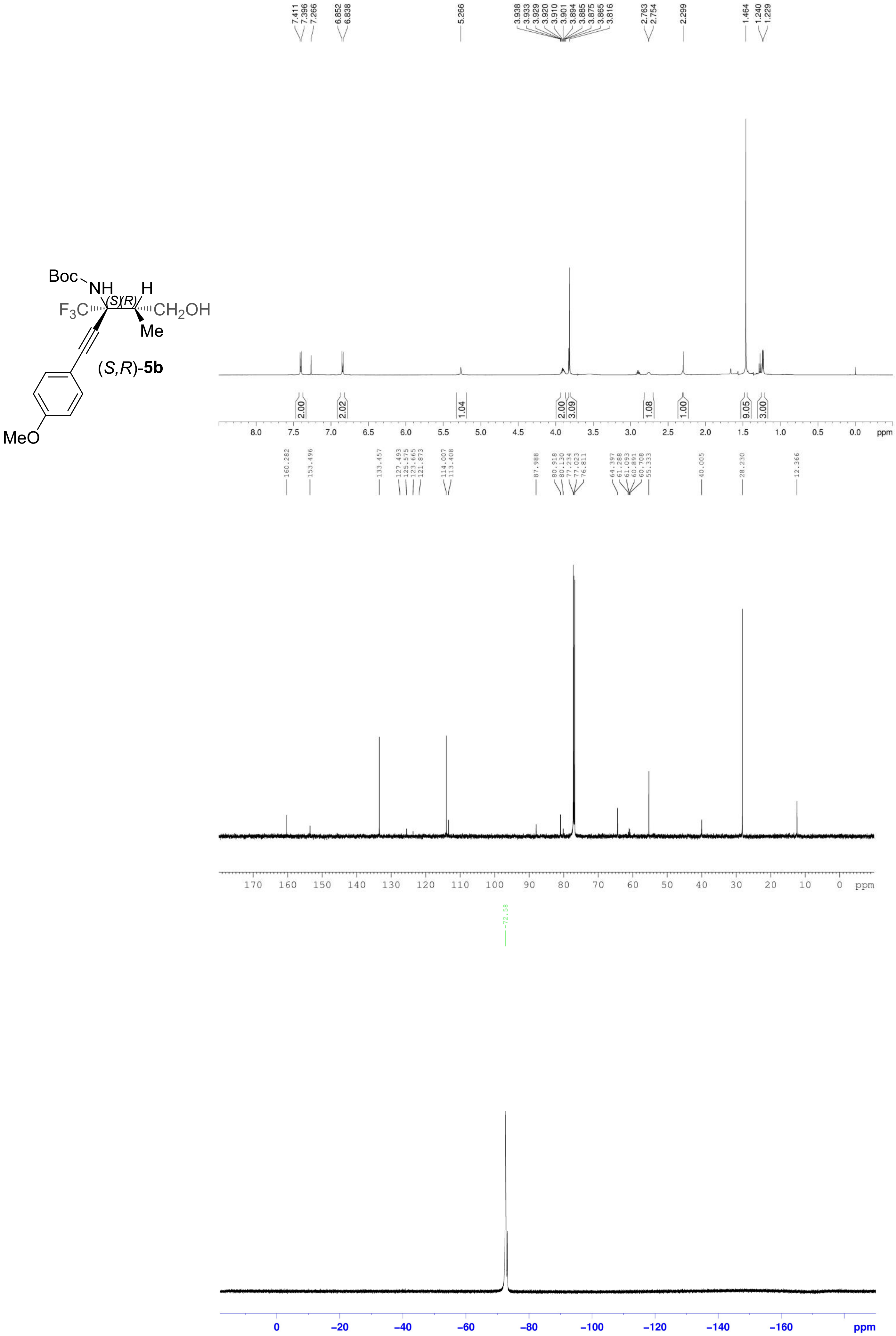

Supplementary Figure 39. ^{1}H, ^{13}C and ^{19}F-NMR Spectrum for (*S*,*R*)-**5b.**

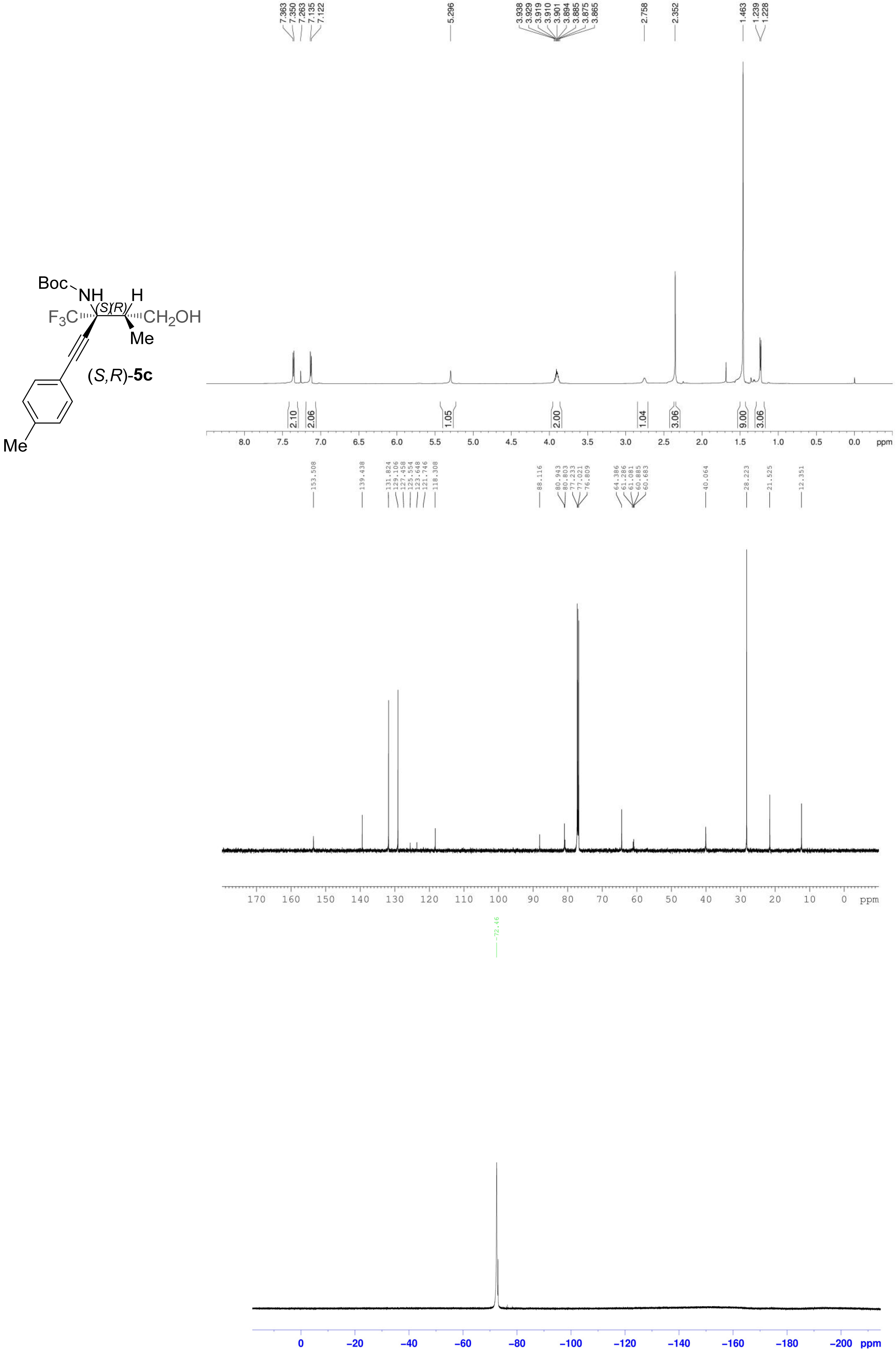

Supplementary Figure 40. ^{1}H, ^{13}C and ^{19}F-NMR Spectrum for (*S,R*)-**5c.**

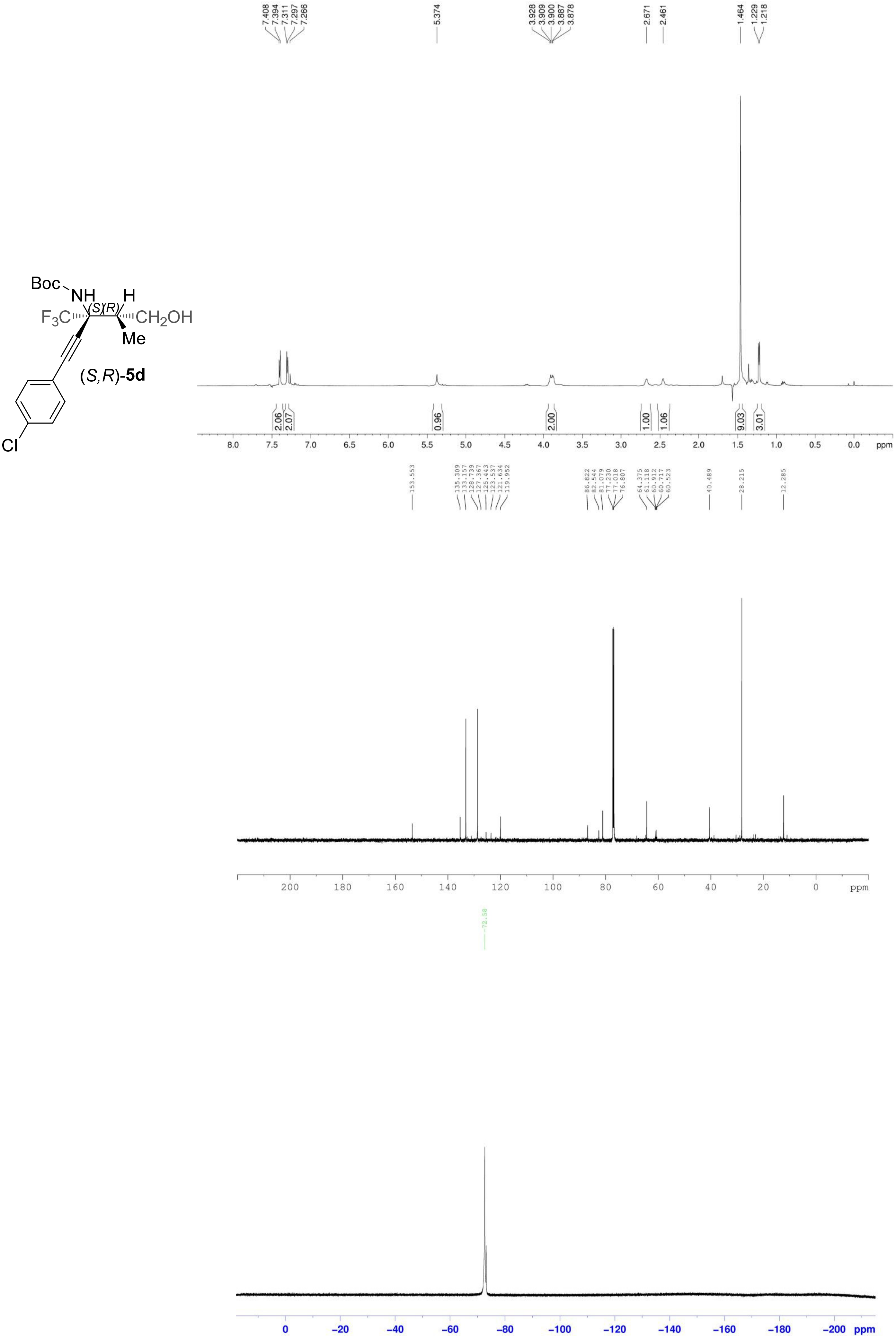

Supplementary Figure 41. ^{1}H, ^{13}C and ^{19}F-NMR Spectrum for (*S*,*R*)-**5d.**

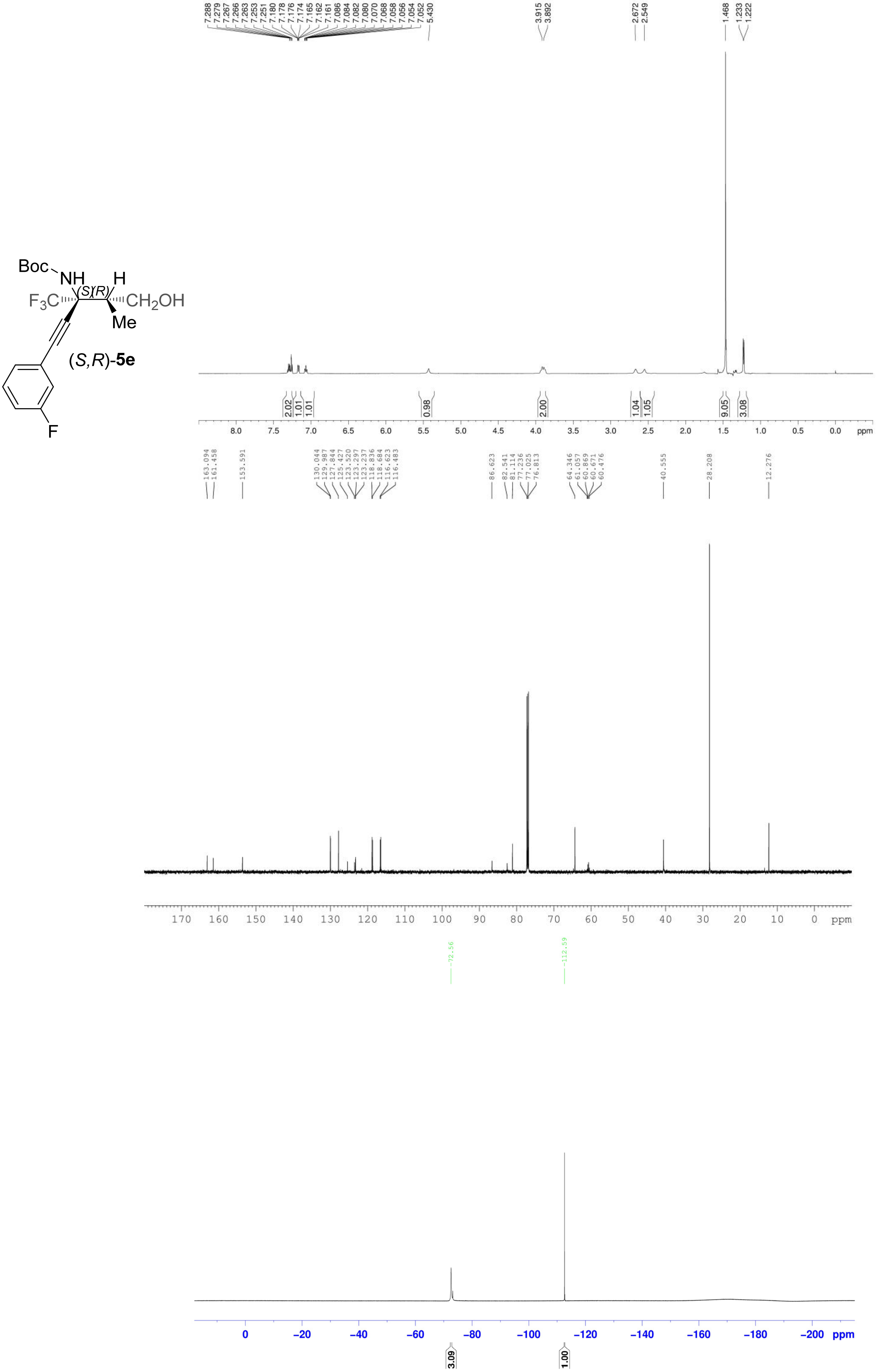

Supplementary Figure 42. ^{1}H, ^{13}C and ^{19}F-NMR Spectrum for (*S*,*R*)-**5e.**

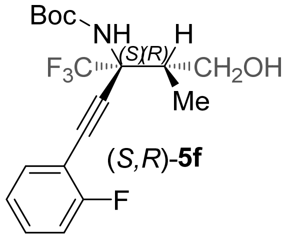

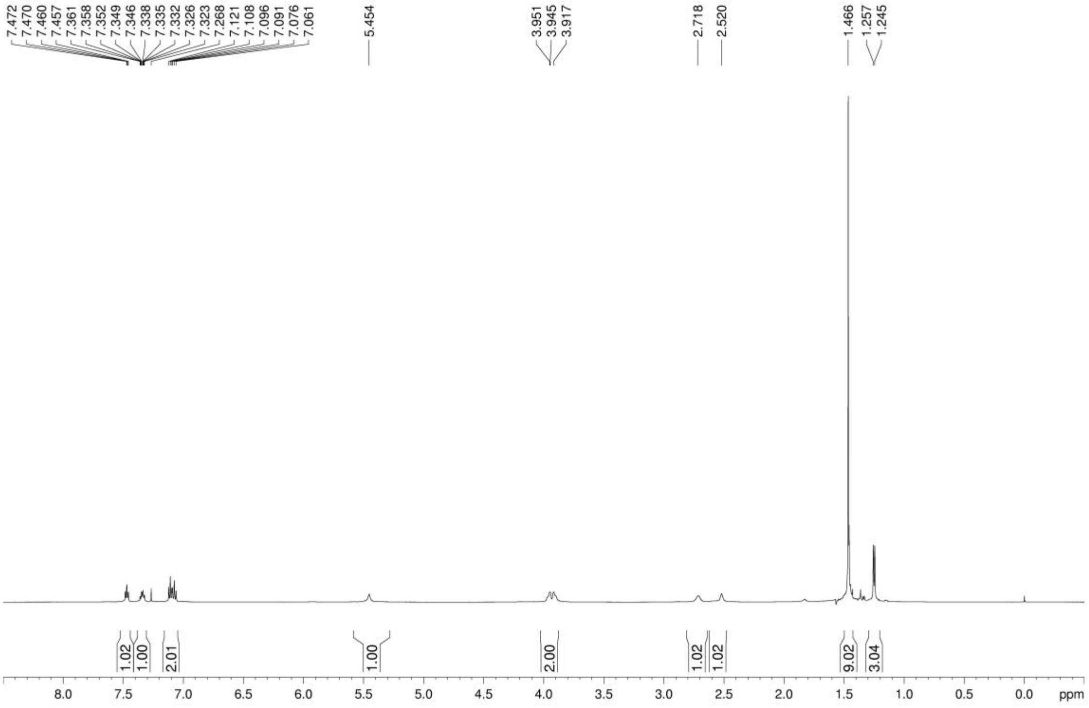

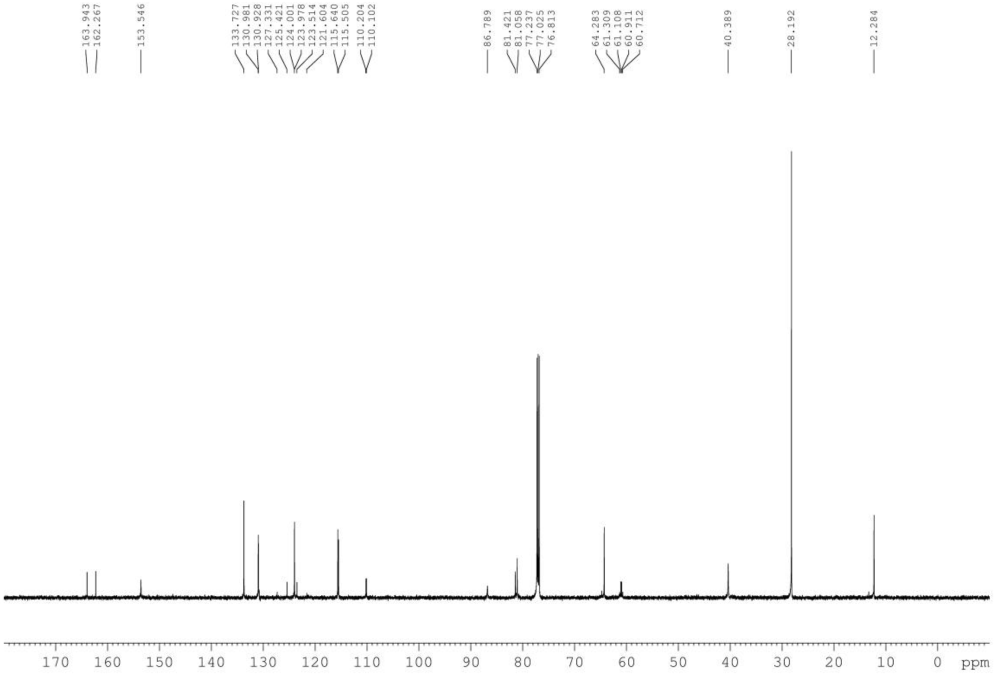

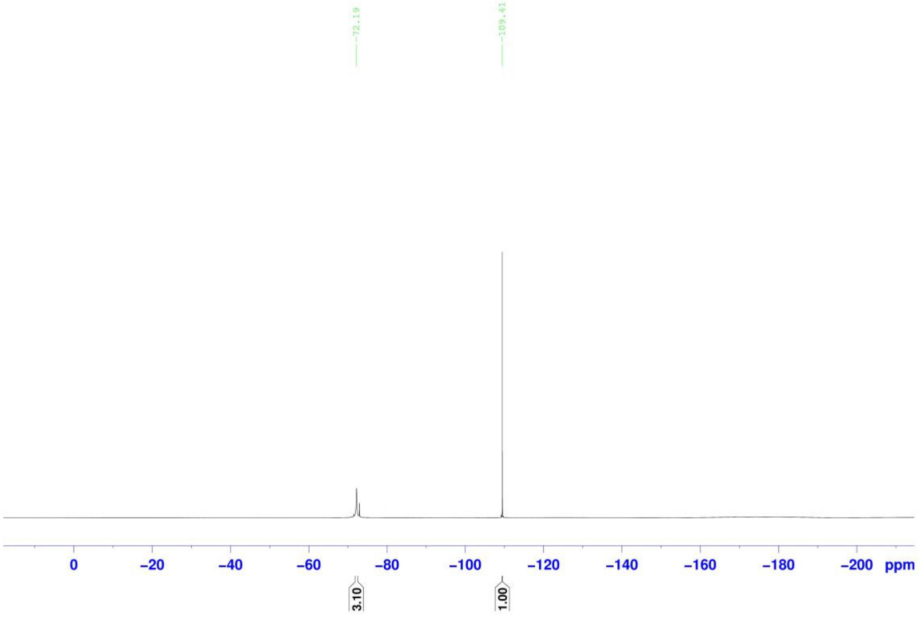

Supplementary Figure 43. ^{1}H, ^{13}C and ^{19}F-NMR Spectrum for (*S,R*)-**5f.**

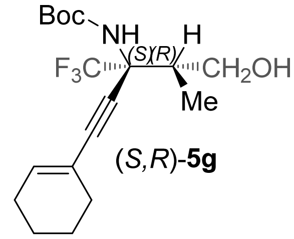

(*S*,*R*)-**5g**

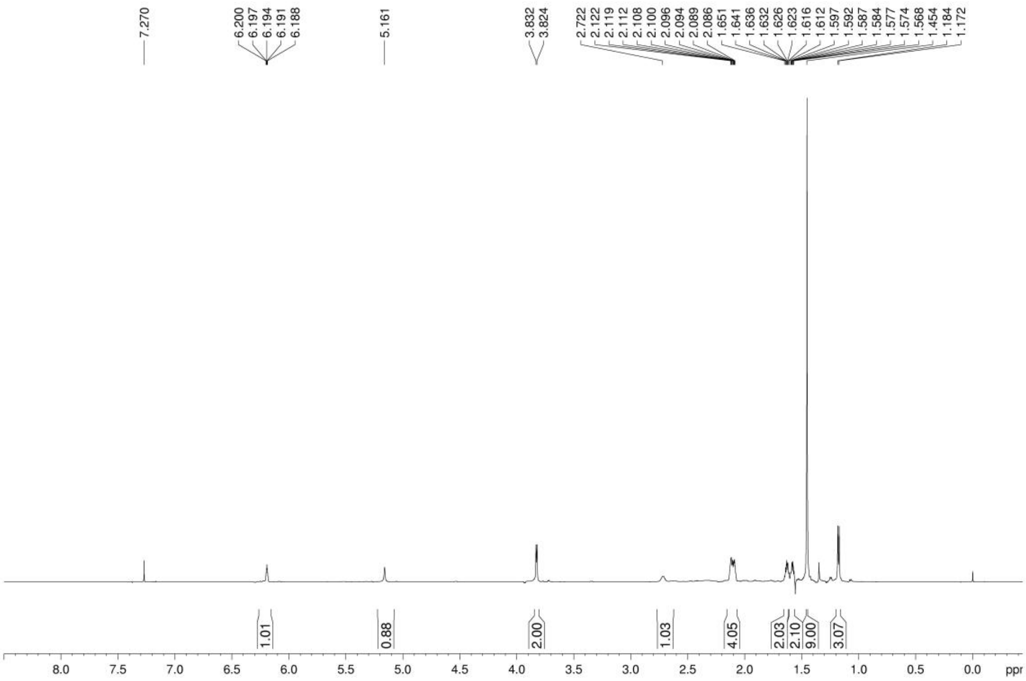

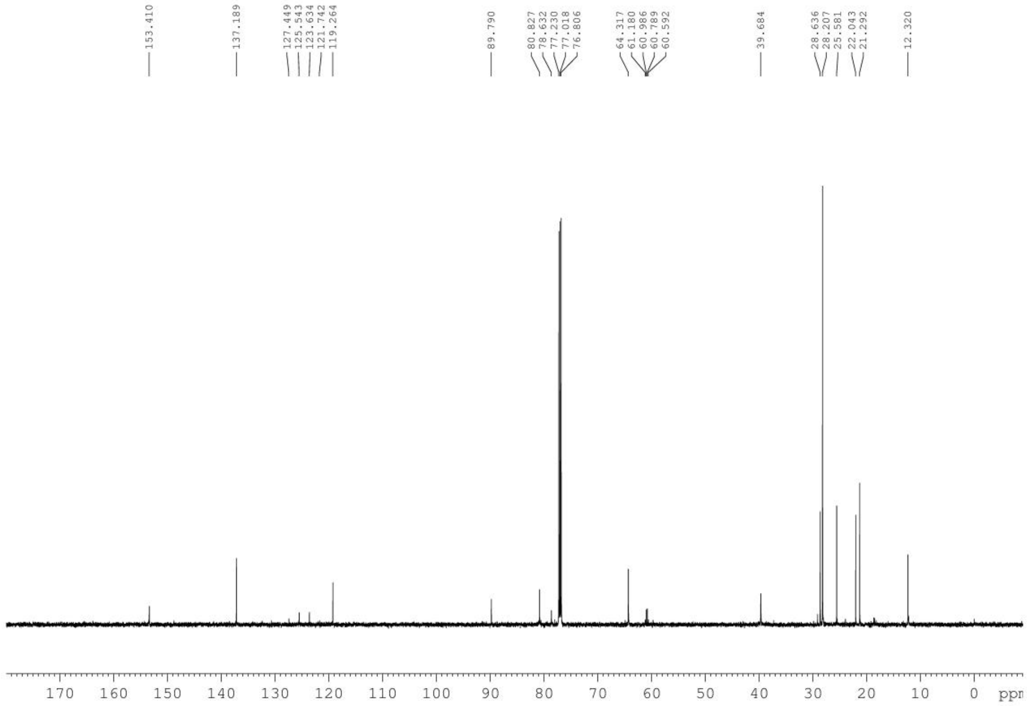

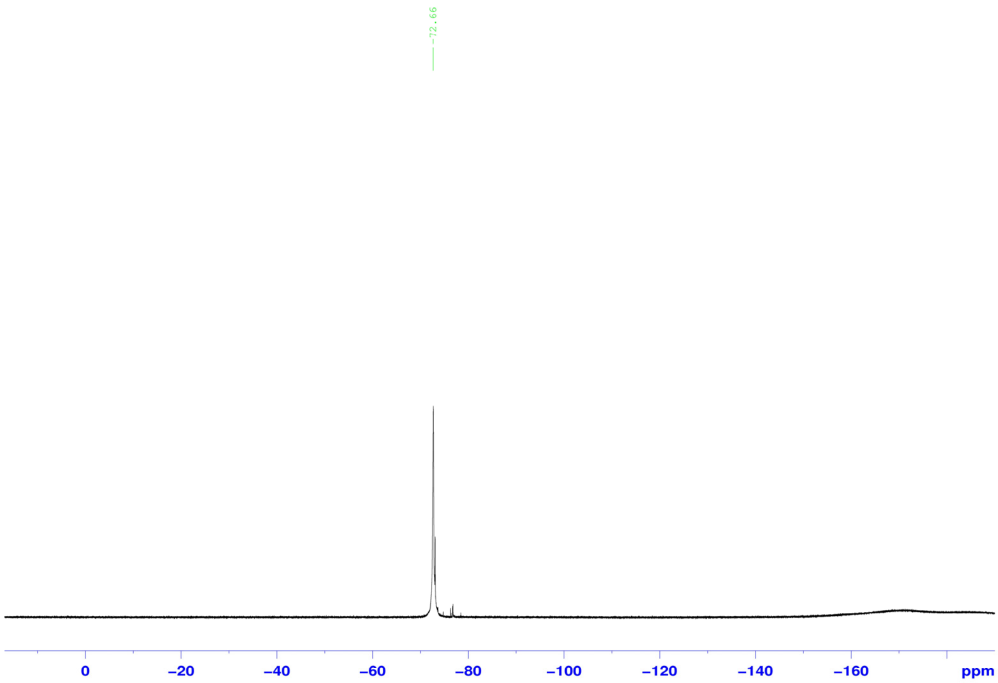

Supplementary Figure 44. ^{1}H, ^{13}C and ^{19}F-NMR Spectrum for (*S*,*R*)-**5g.**

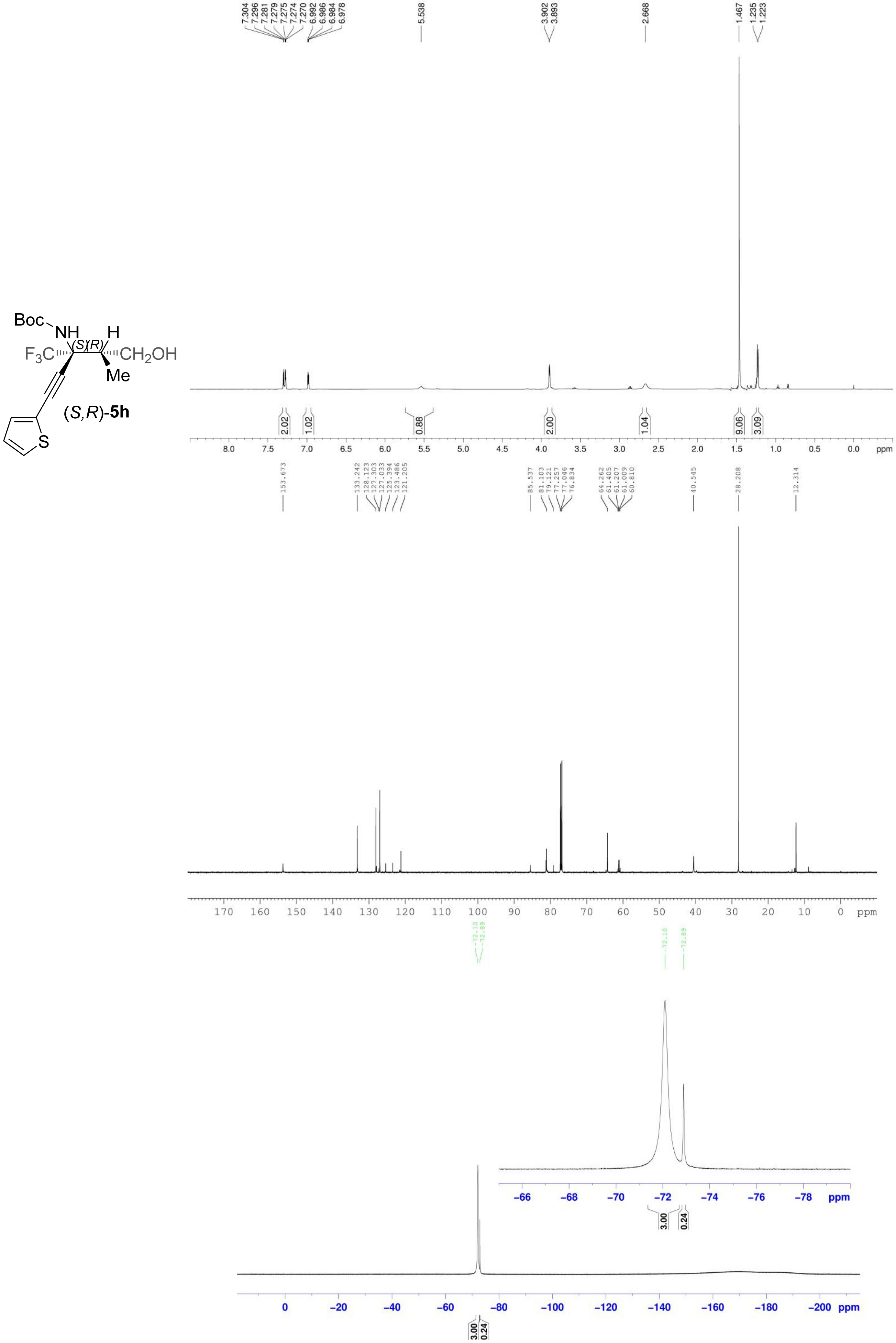

Supplementary Figure 45. ^{1}H, ^{13}C and ^{19}F-NMR Spectrum for (*S,R*)-**5h.**

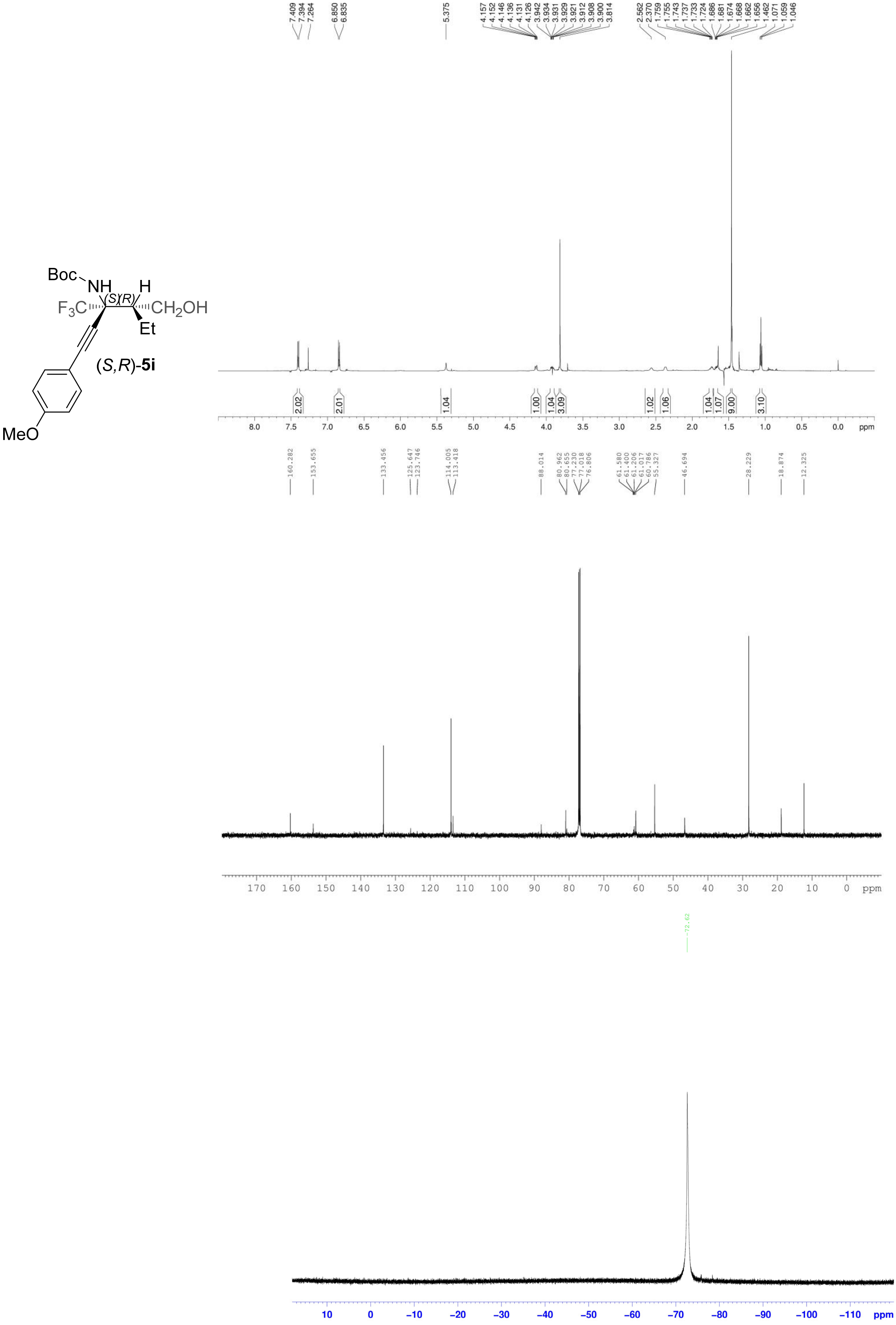

Supplementary Figure 46. ^{1}H, ^{13}C and ^{19}F-NMR Spectrum for (*S,R*)-**5i.**

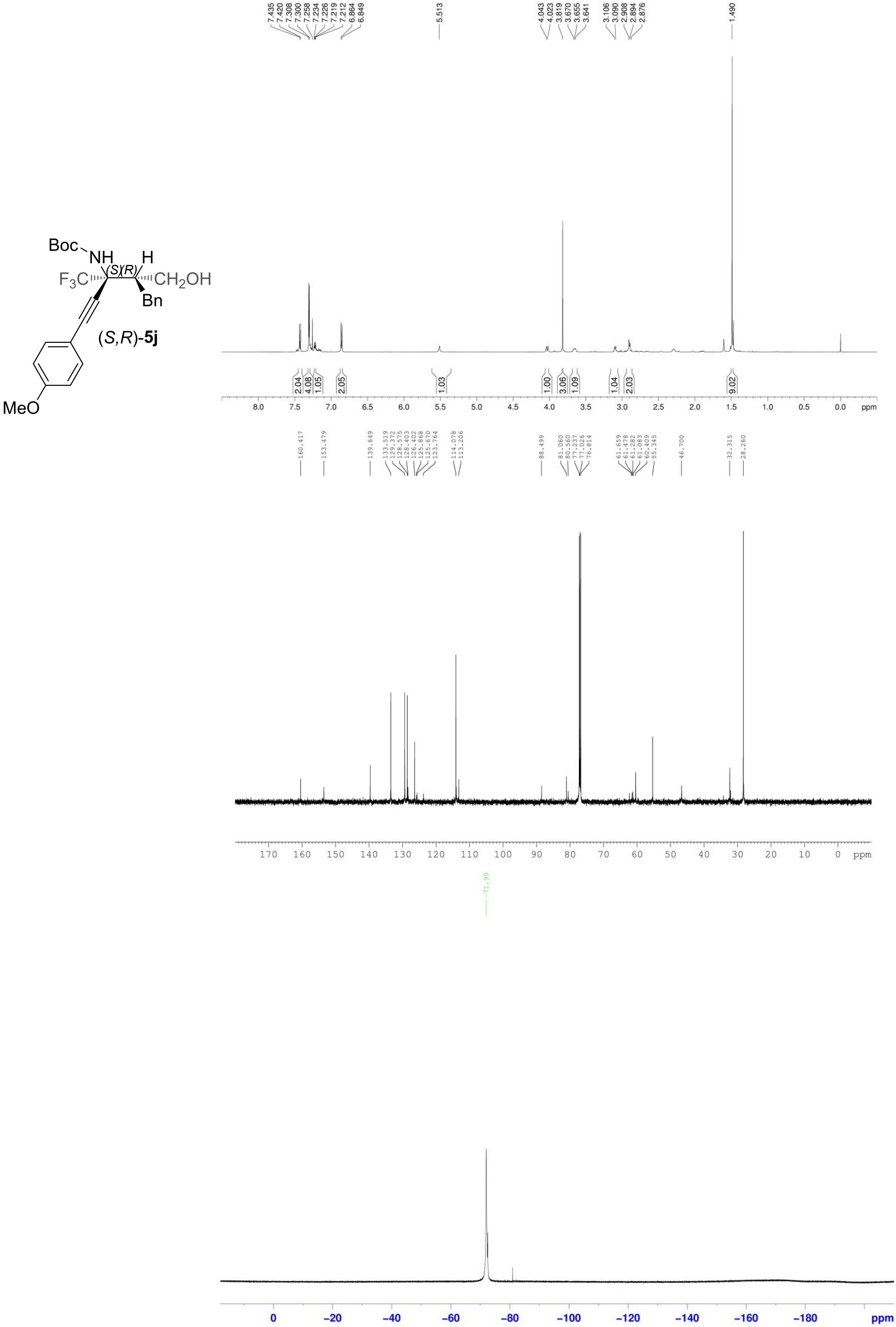

Supplementary Figure 47. ^{1}H, ^{13}C and ^{19}F-NMR Spectrum for (*S,R*)-**5j.**

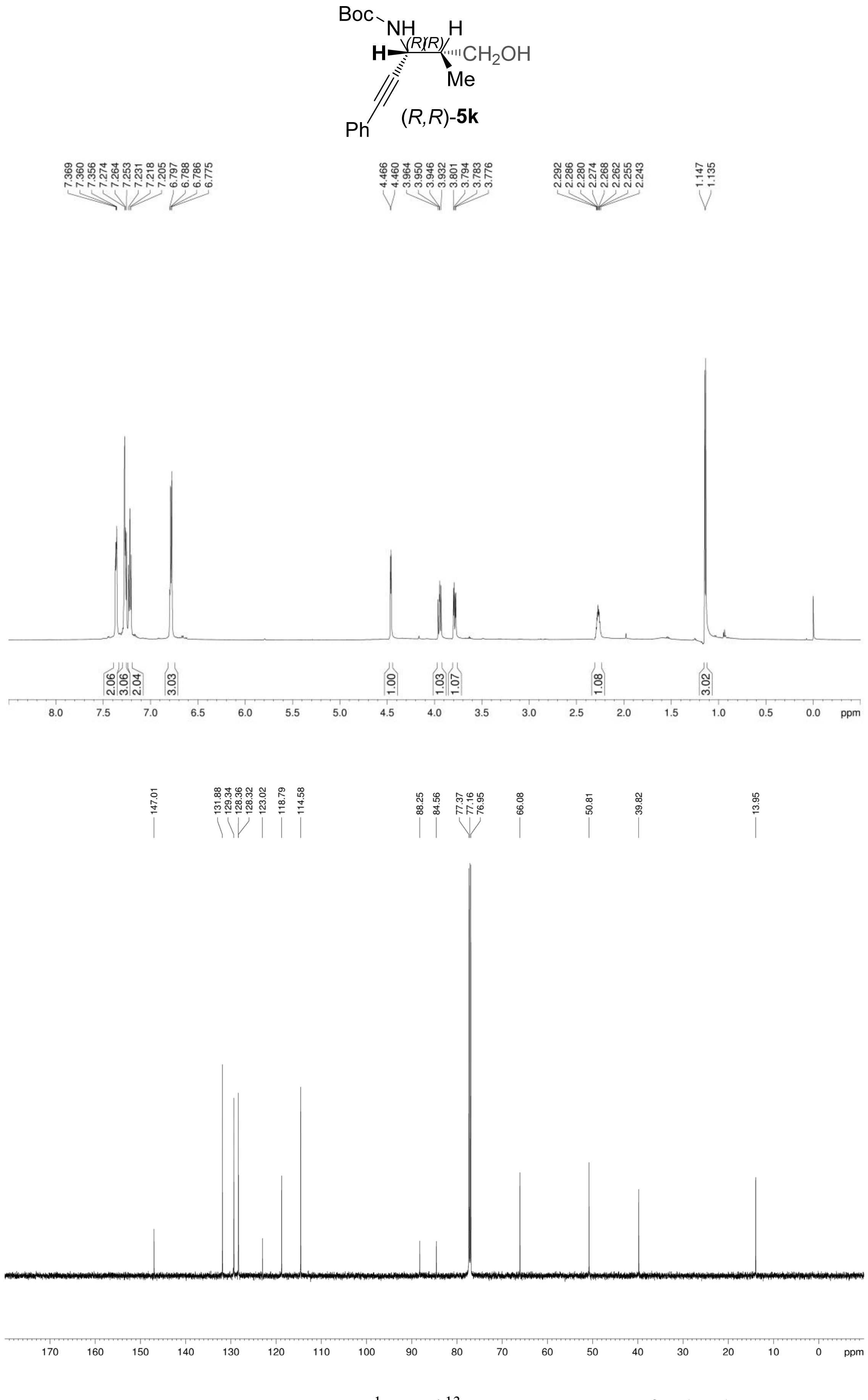

Supplementary Figure 48. ^{1}H and ^{13}C-NMR Spectrum for (*R,R*)-**5k.**

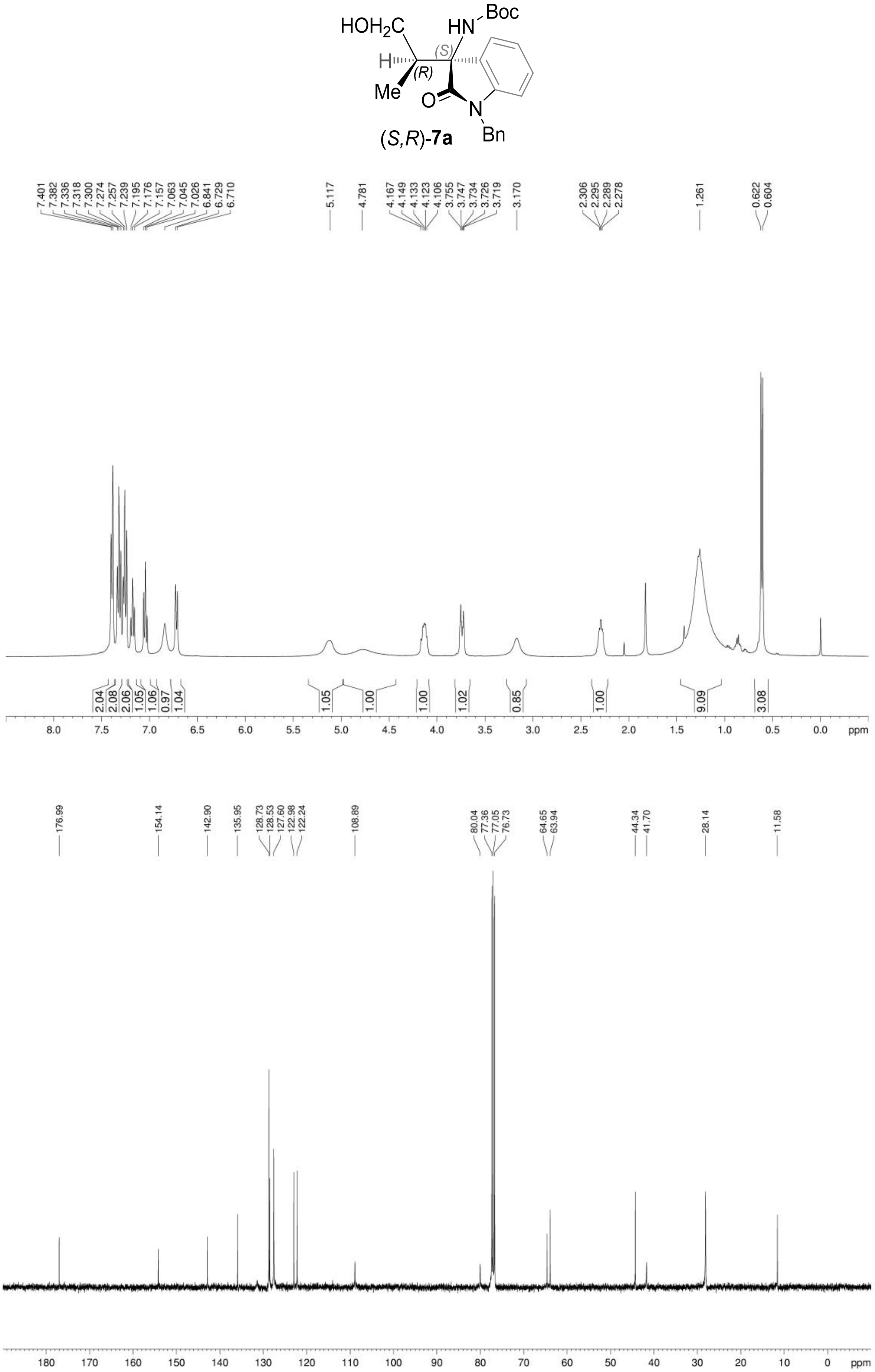

Supplementary Figure 49. ^{1}H and ^{13}C-NMR Spectrum for (*S,R*)-**7a.**

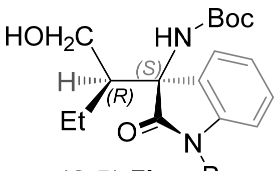

(*S*,*R*)-**7b**

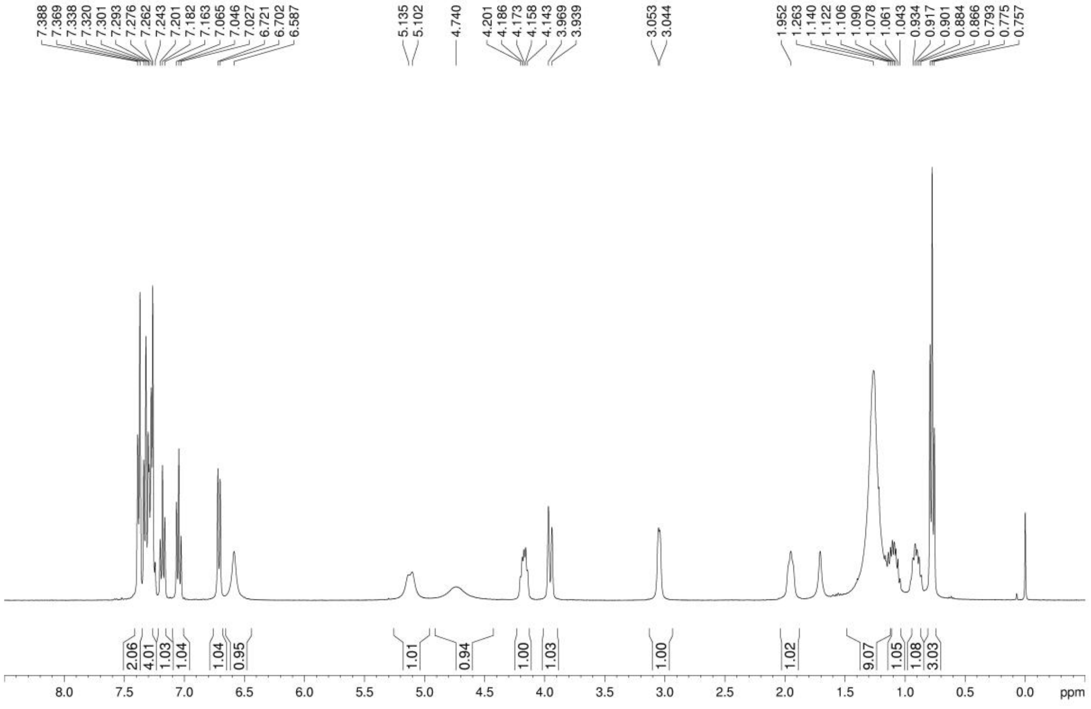

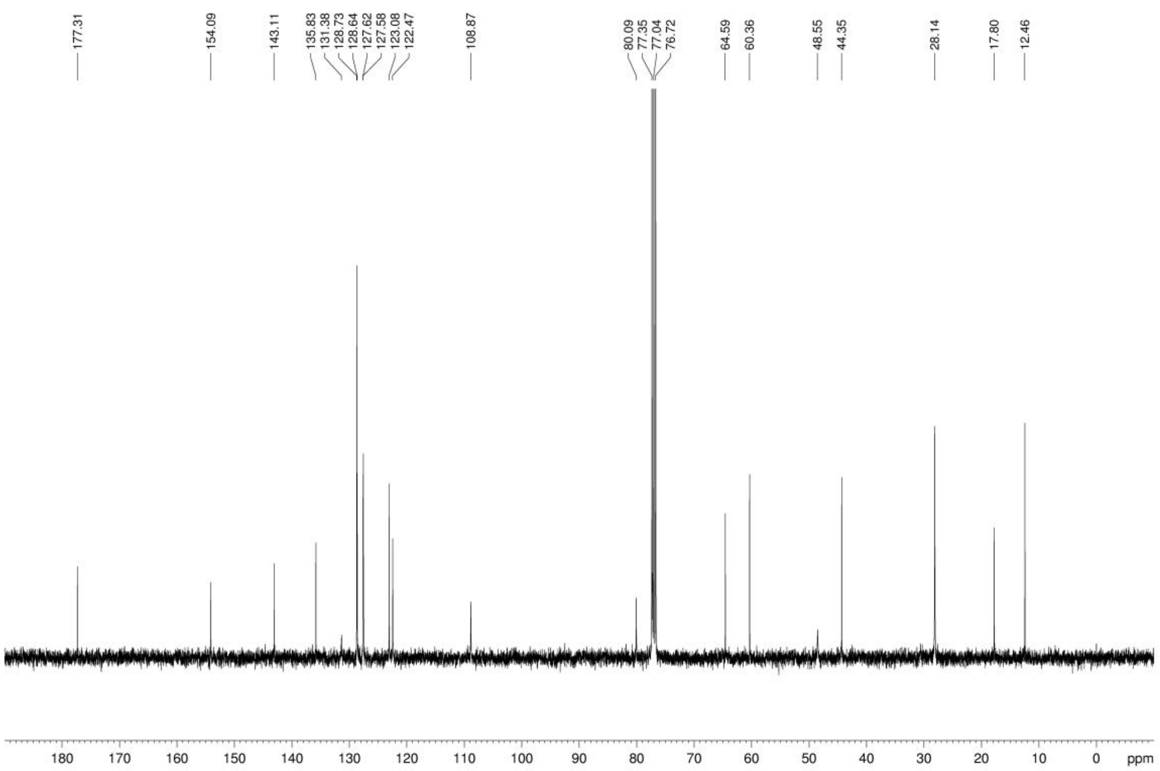

Supplementary Figure 50. ^{1}H and ^{13}C-NMR Spectrum for (*S,R*)-**7b.**

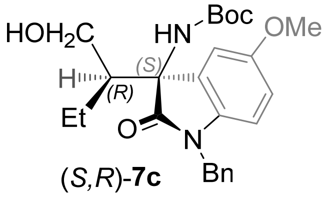

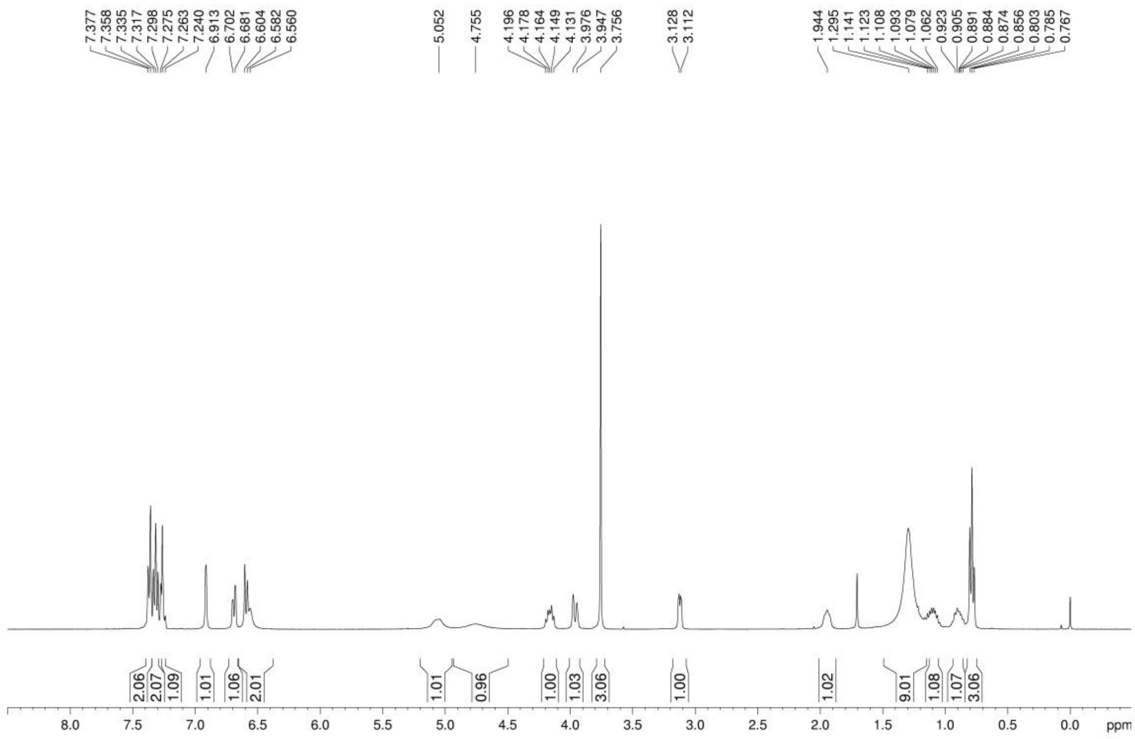

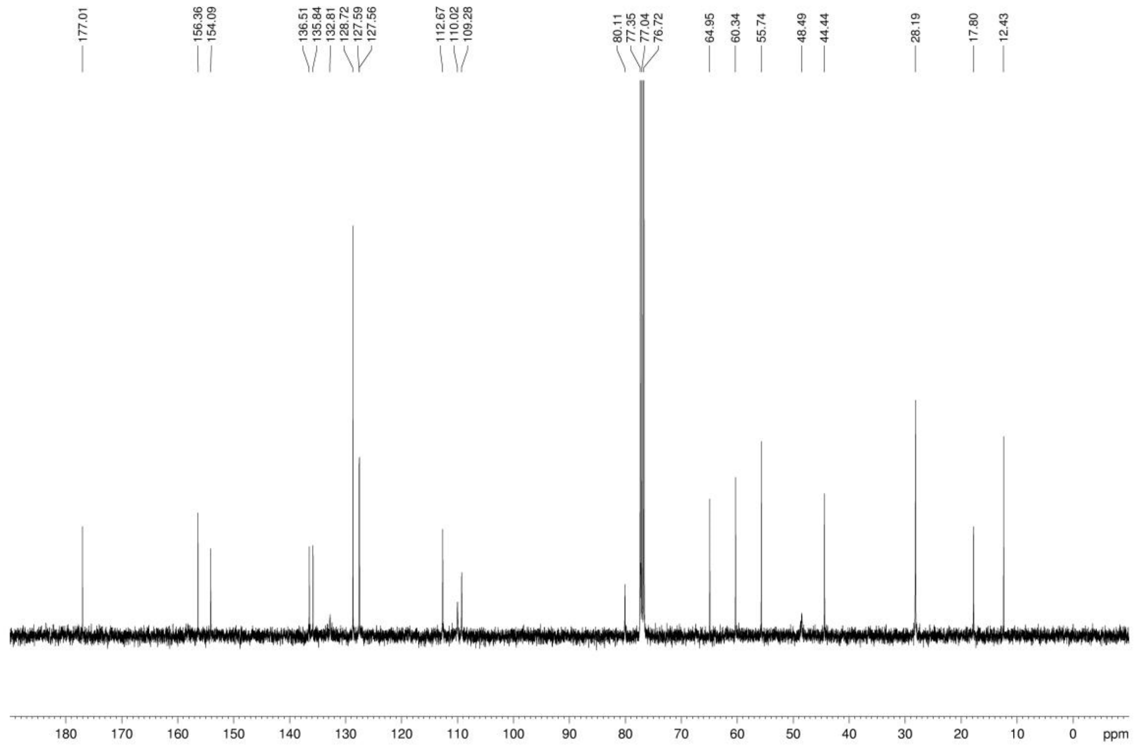

Supplementary Figure 51. ^{1}H and ^{13}C-NMR Spectrum for (*S,R*)-**7c.**

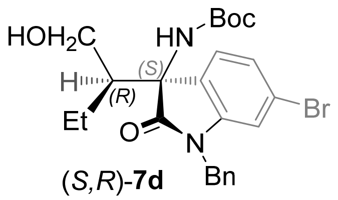

(*S*,*R*)-**7d**

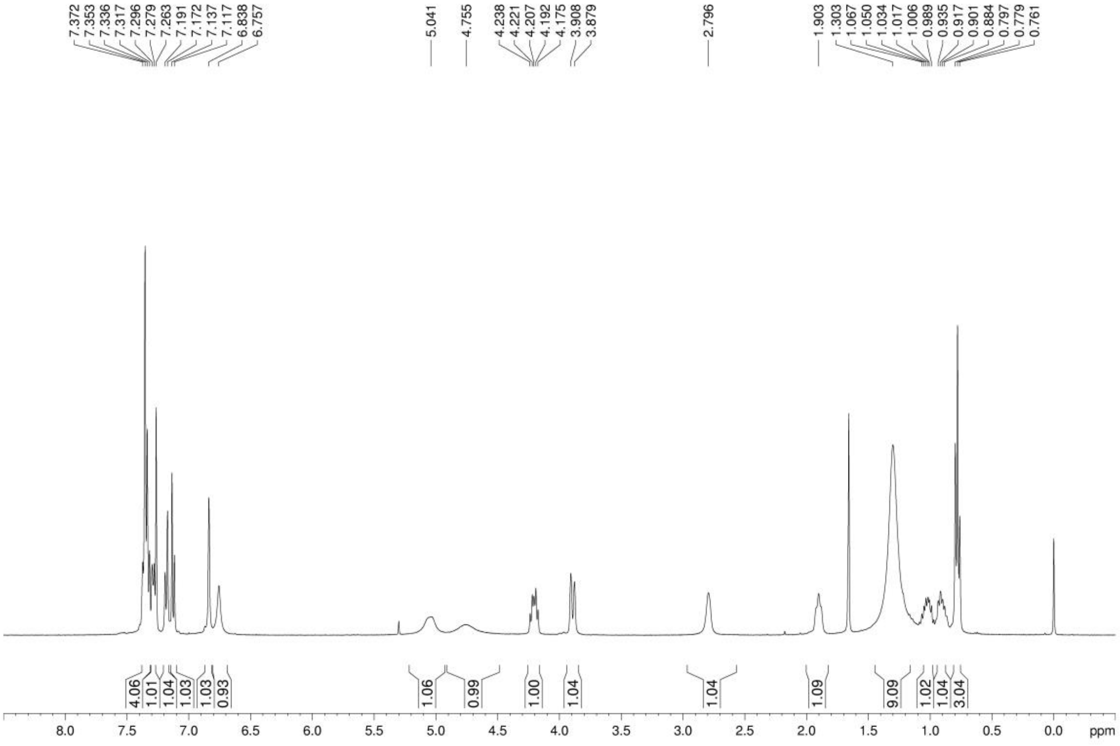

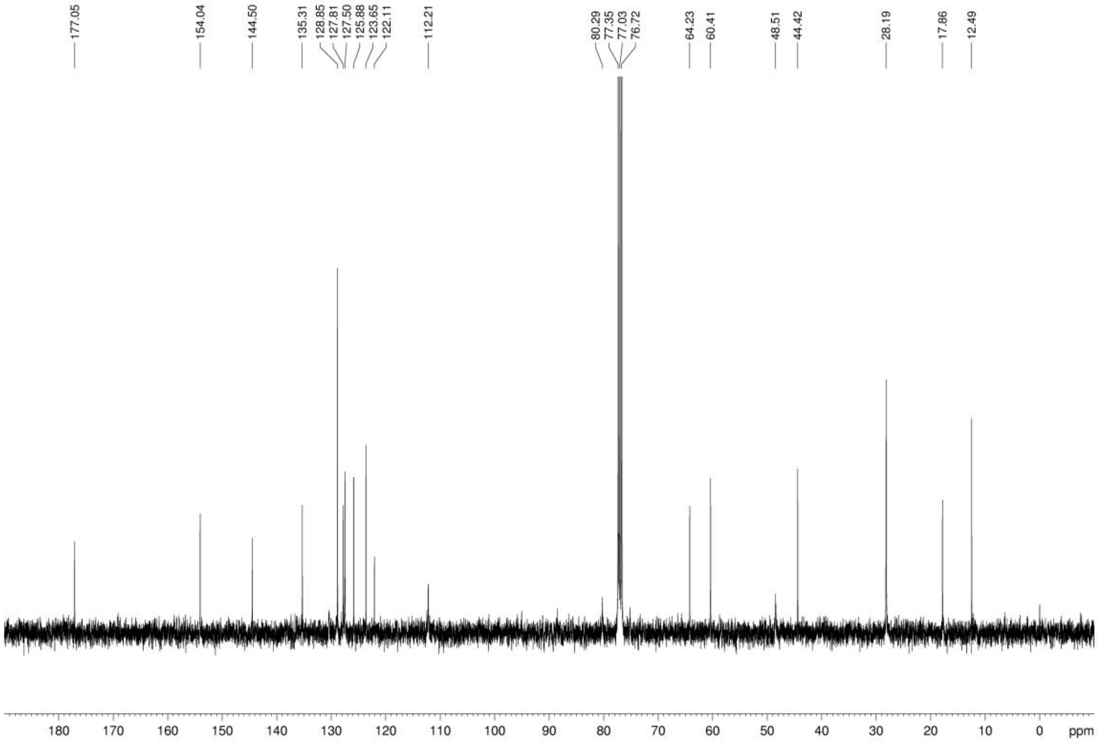

Supplementary Figure 52. ^{1}H and ^{13}C-NMR Spectrum for (*S,R*)-**7d.**

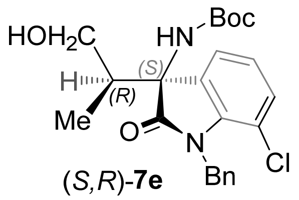

(*S,R*)-**7e**

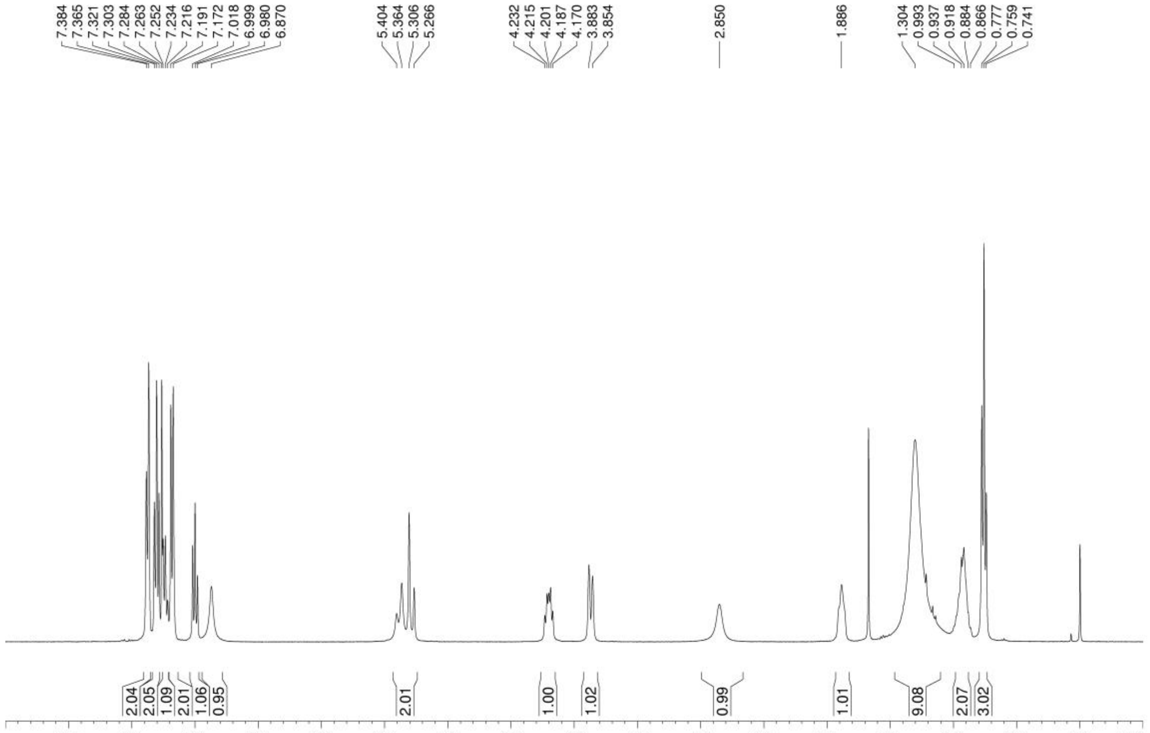

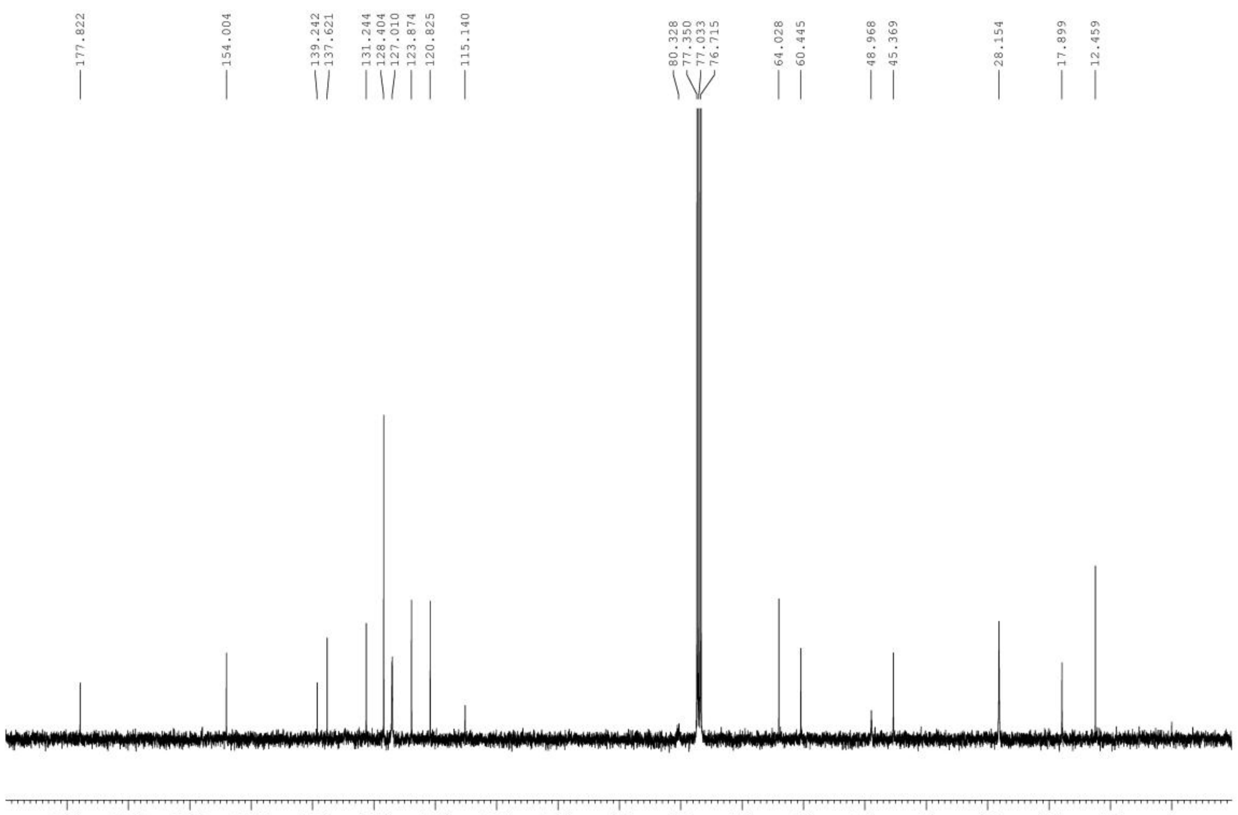

Supplementary Figure 53. ^{1}H and ^{13}C-NMR Spectrum for (*S,R*)-**7e.**

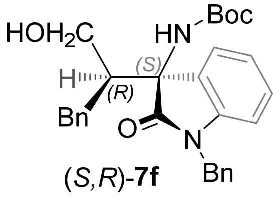

(*S*,*R*)-**7f**

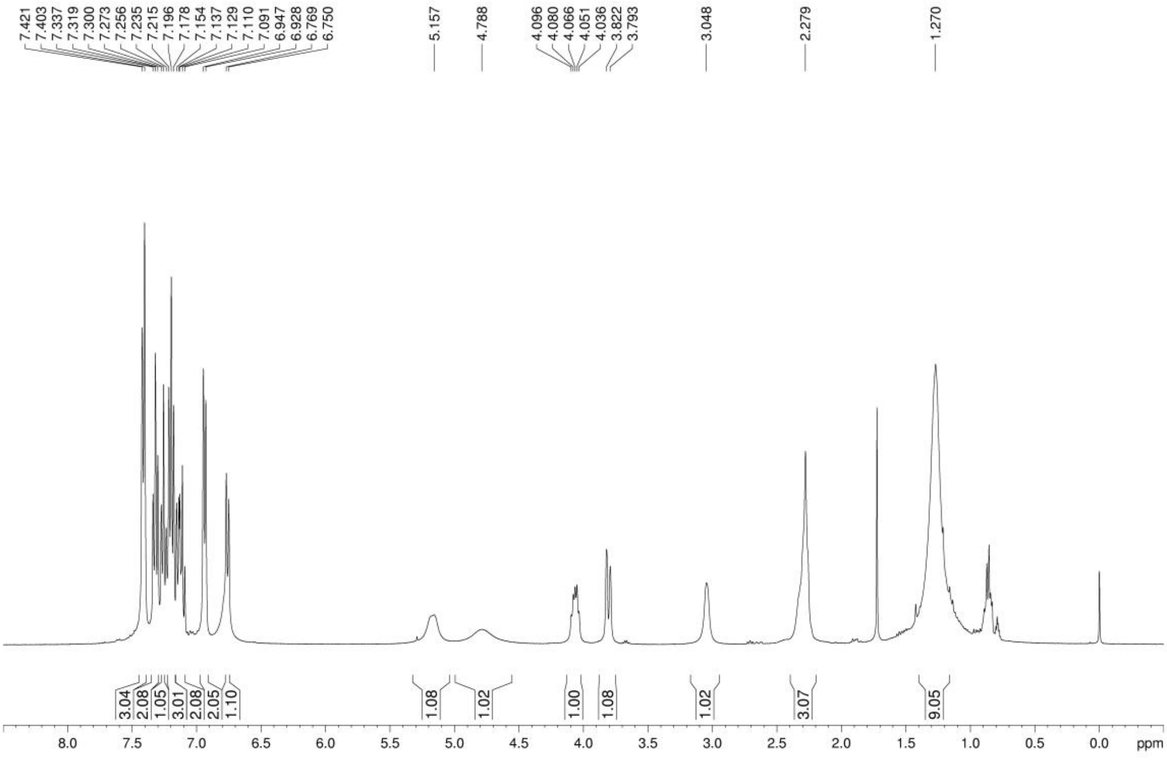

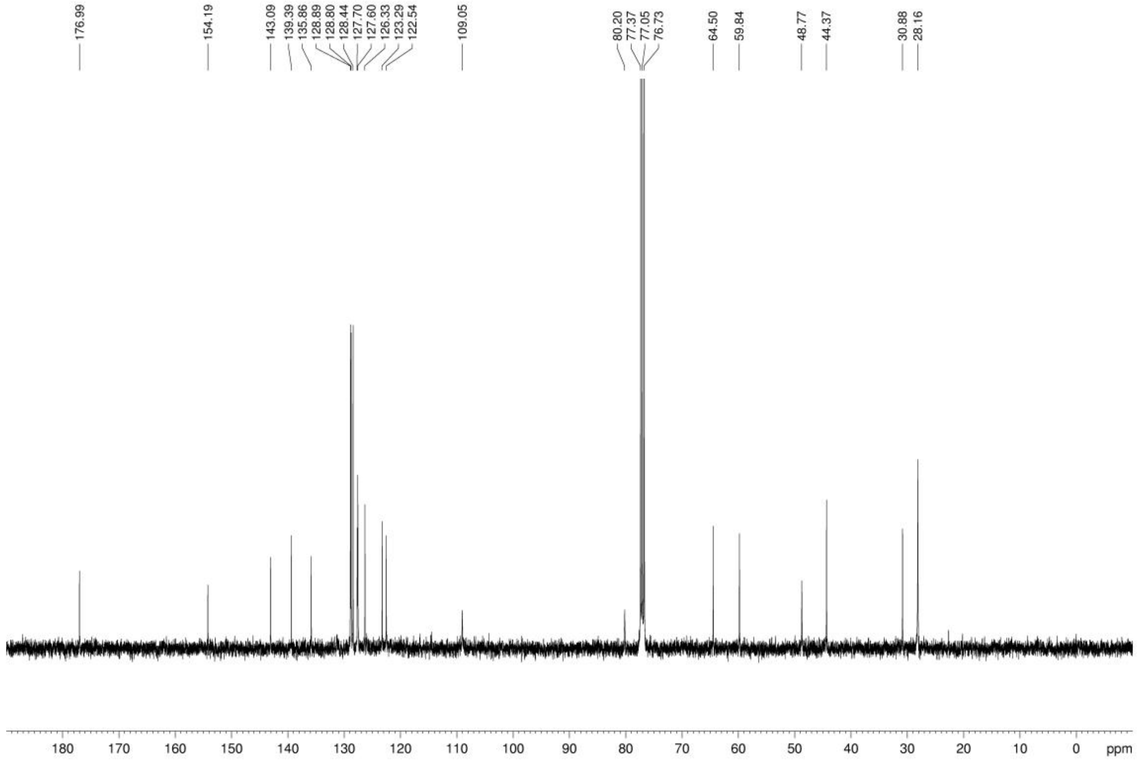

Supplementary Figure 54. ^{1}H and ^{13}C-NMR Spectrum for (*S,R*)-**7f.**

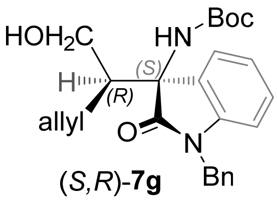

(*S*,*R*)-**7g**

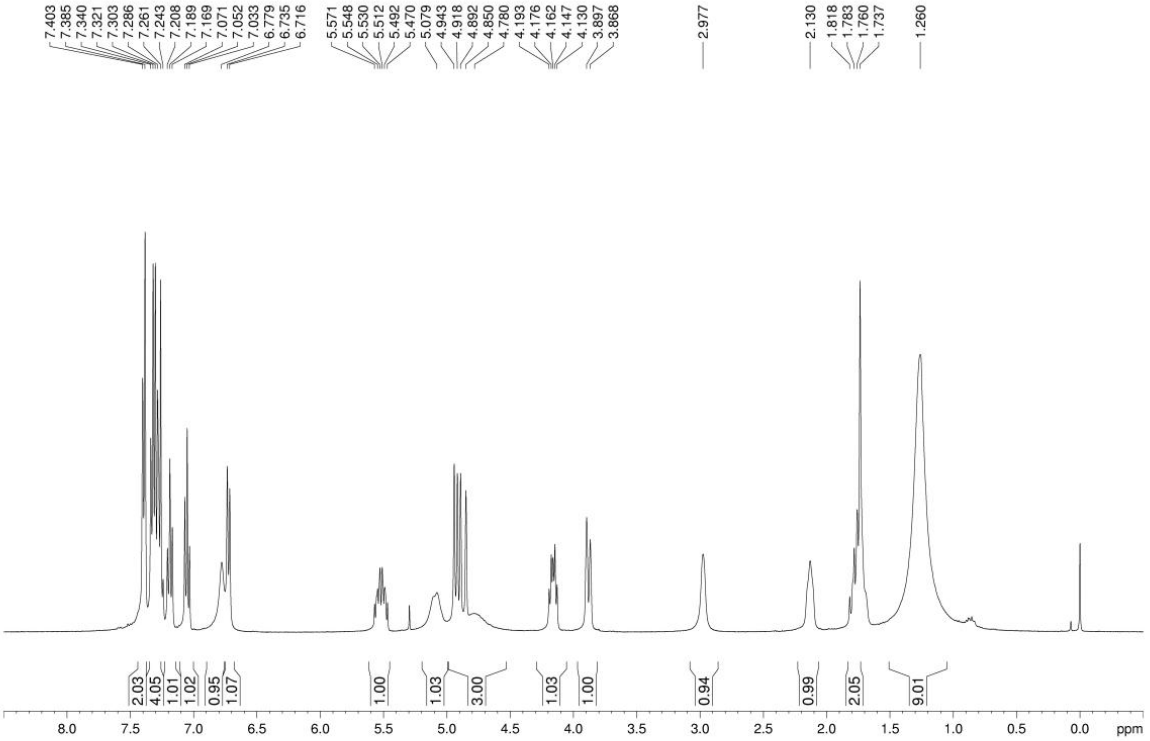

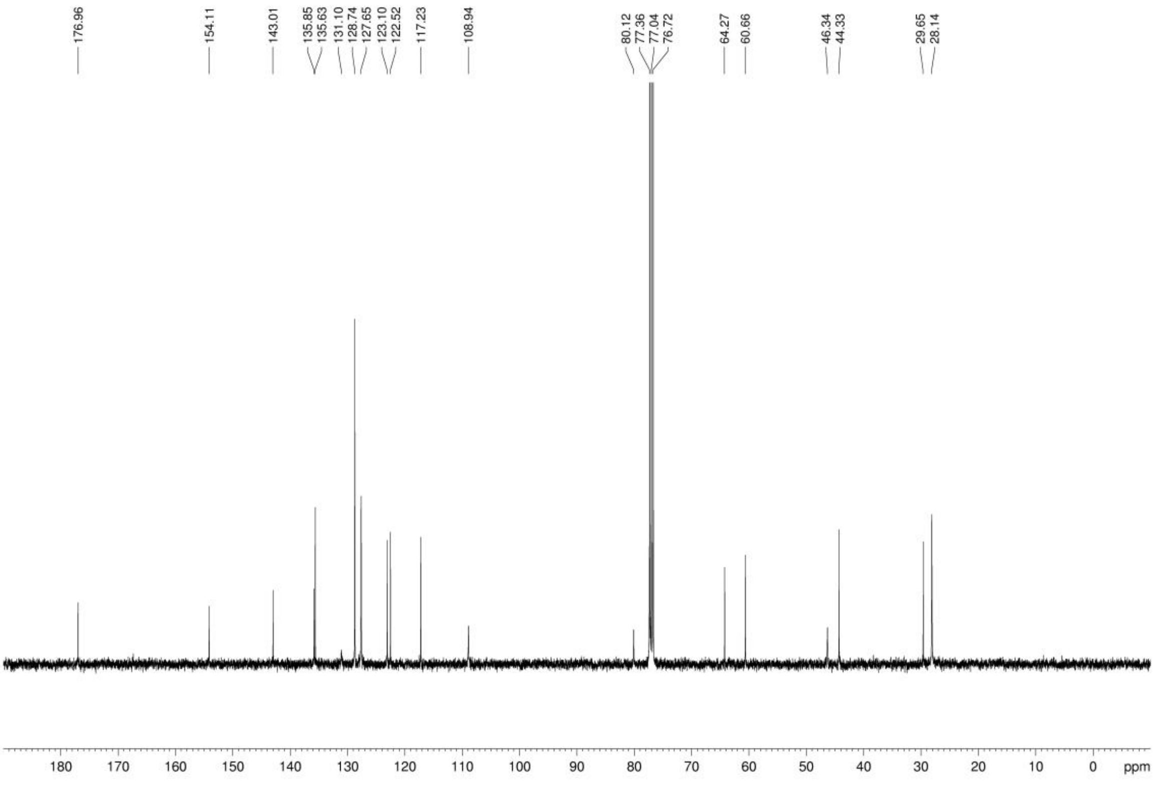

Supplementary Figure 55. ^{1}H and ^{13}C-NMR Spectrum for (*S,R*)-**7g.**

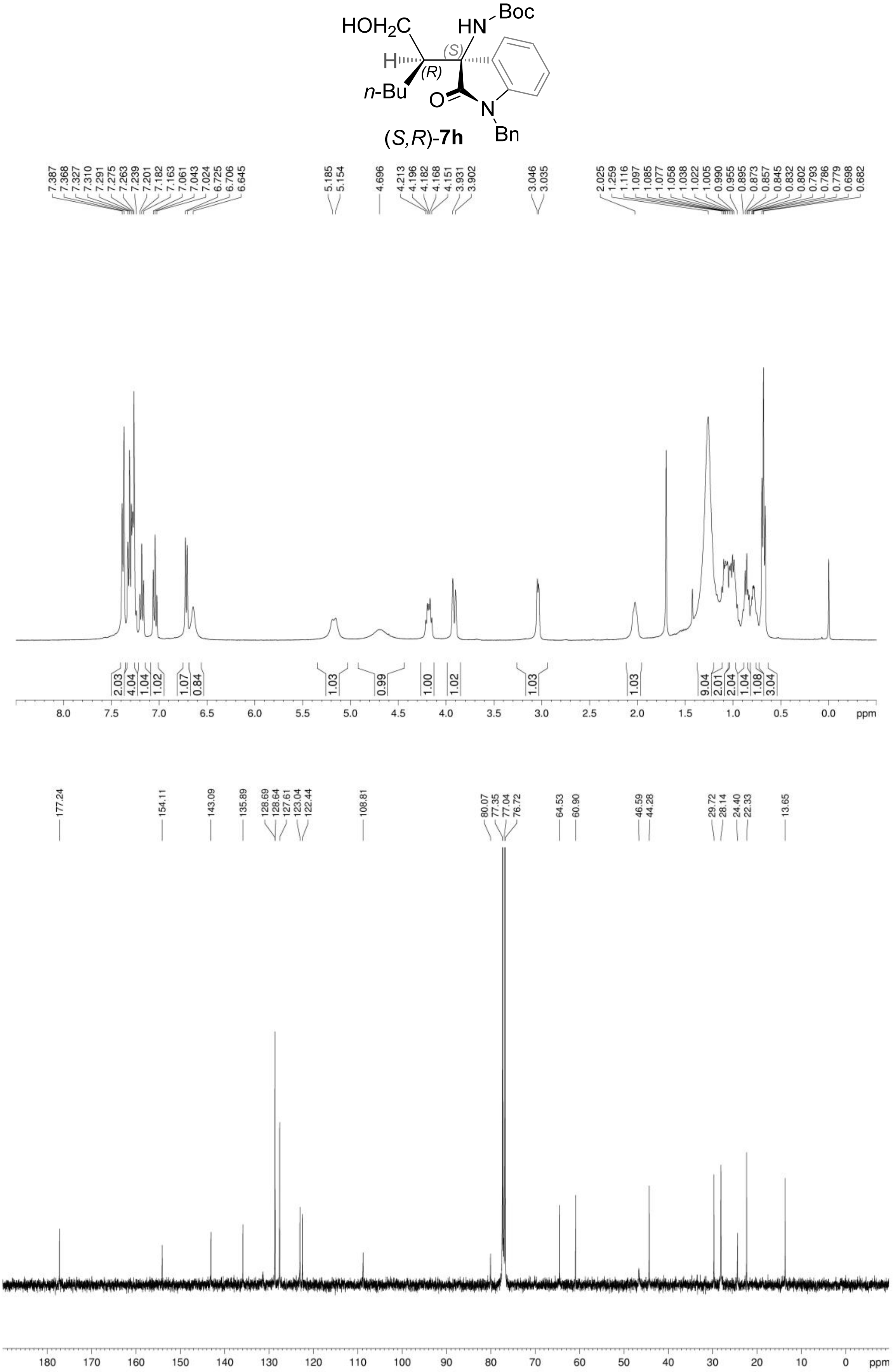

Supplementary Figure 56. ^{1}H and ^{13}C-NMR Spectrum for (*S*,*R*)-**7h.**

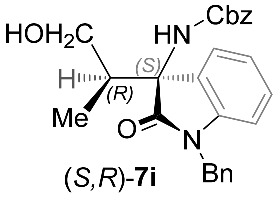

(*S*,*R*)-**7i**

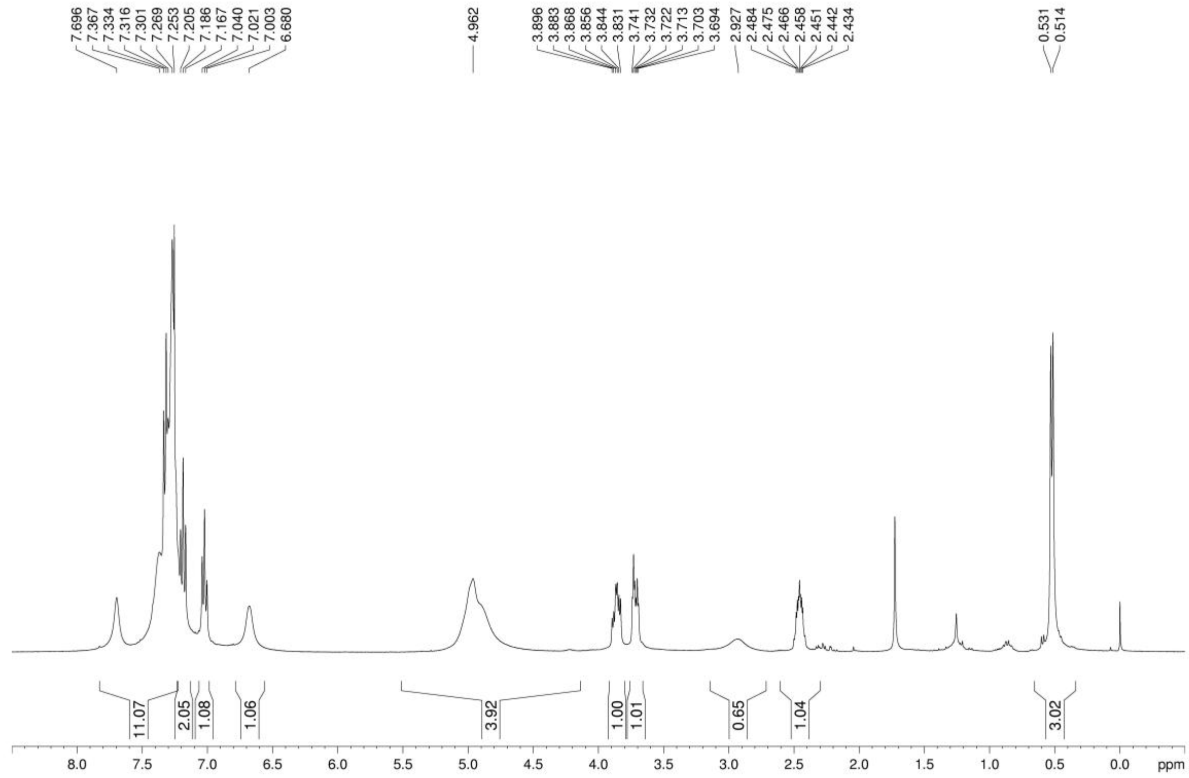

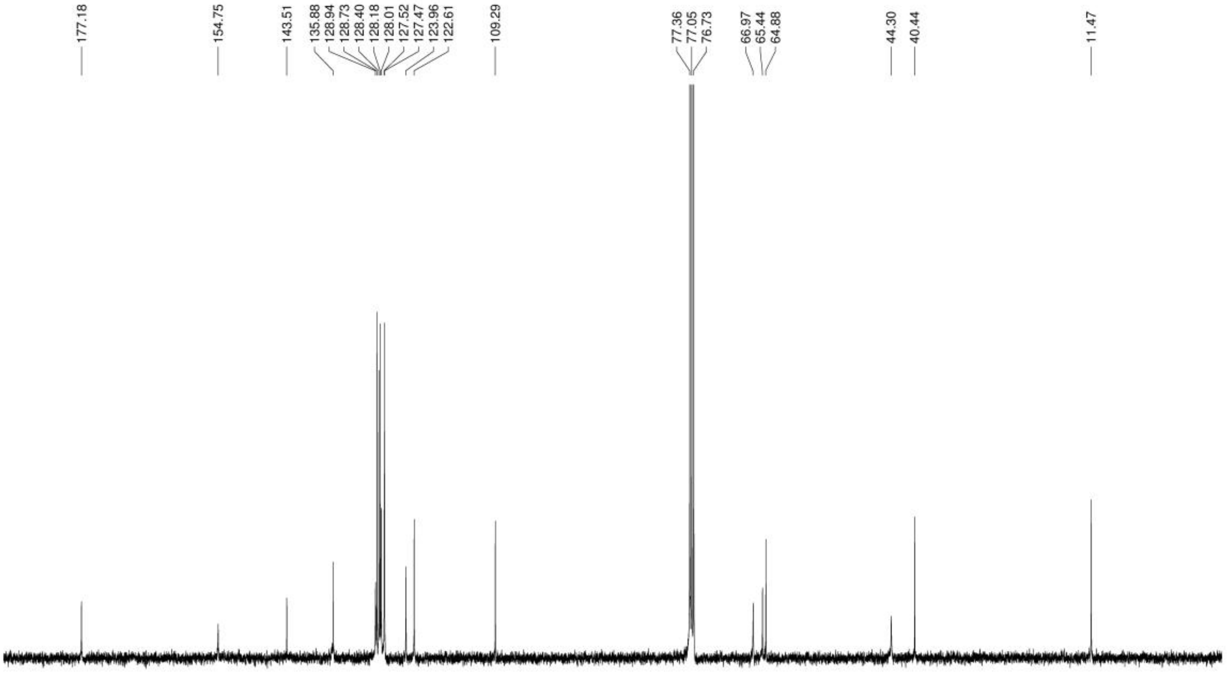

Supplementary Figure 57. ^{1}H and ^{13}C-NMR Spectrum for (*S,R*)-**7i.**

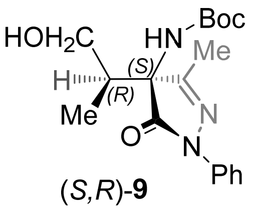

(*S*,*R*)-**9**

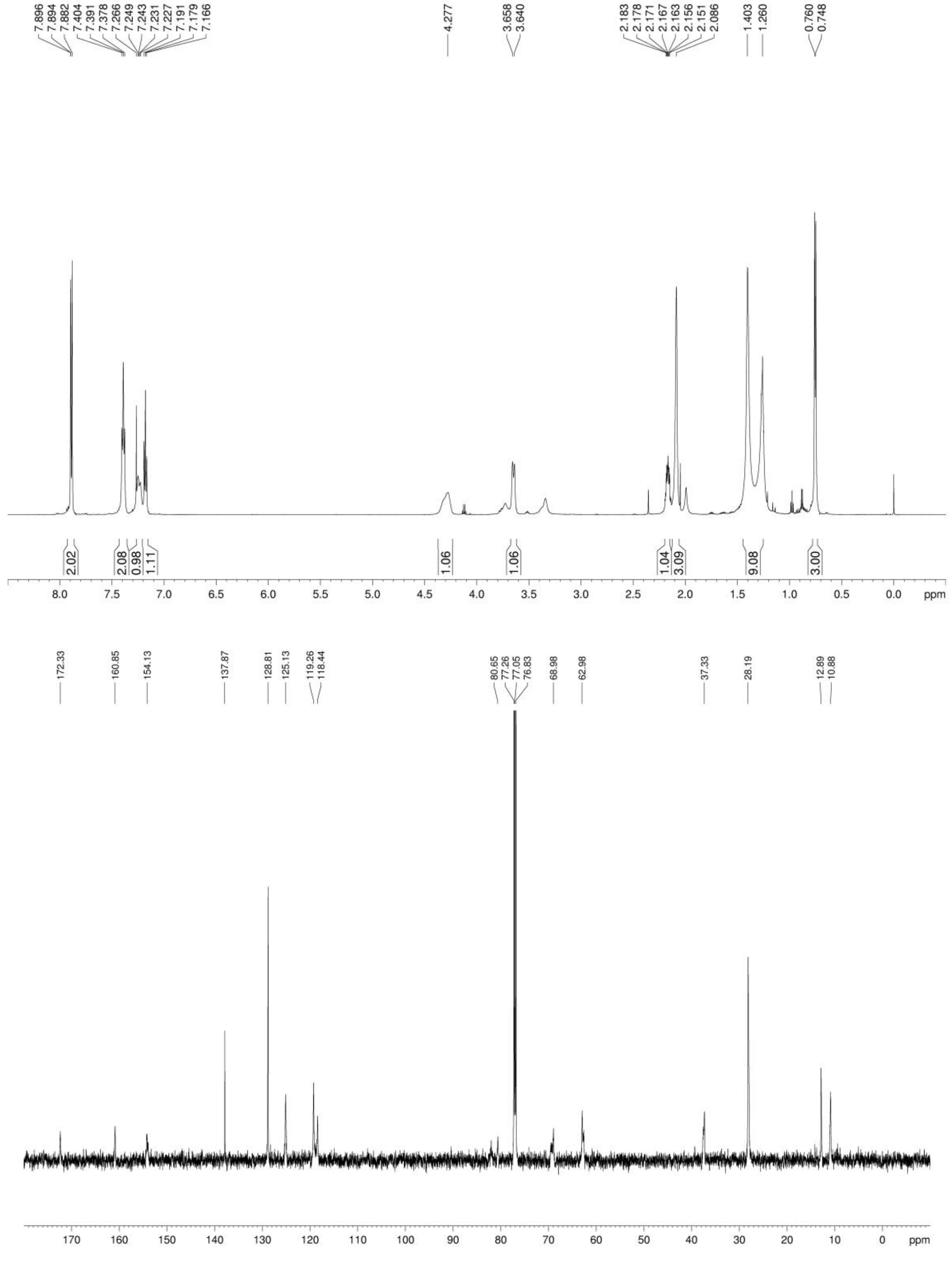

Supplementary Figure 58. ^{1}H and ^{13}C-NMR Spectrum for (*S,R*)-**9.**

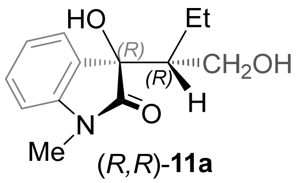

(*R*,*R*)-**11a**

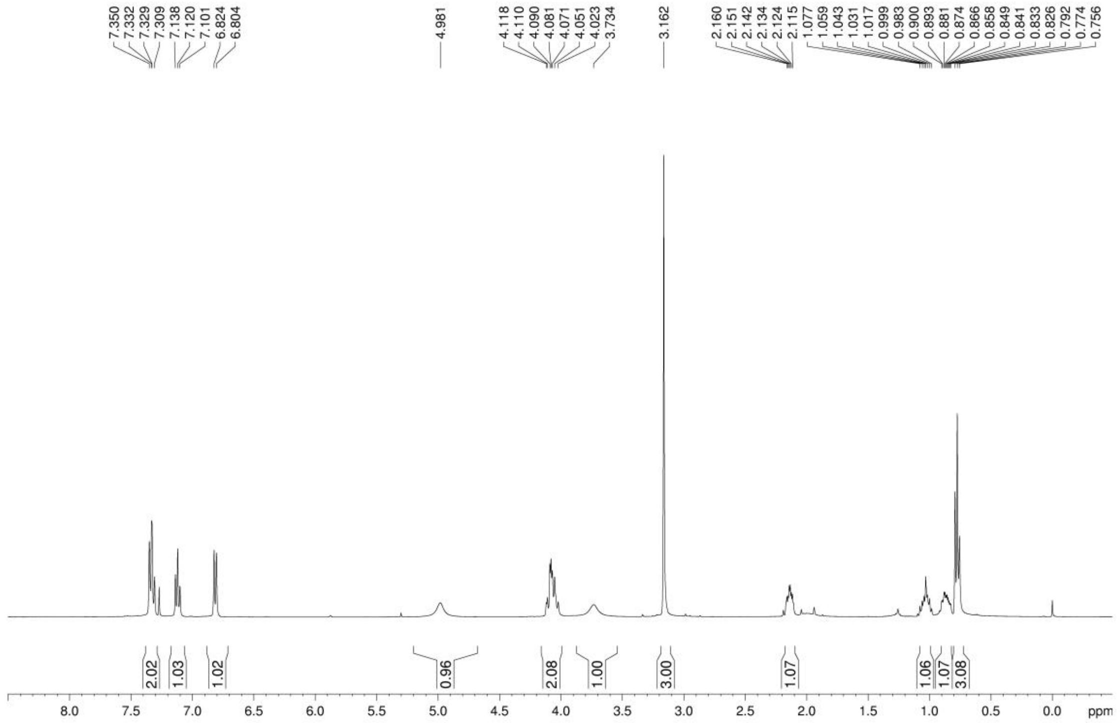

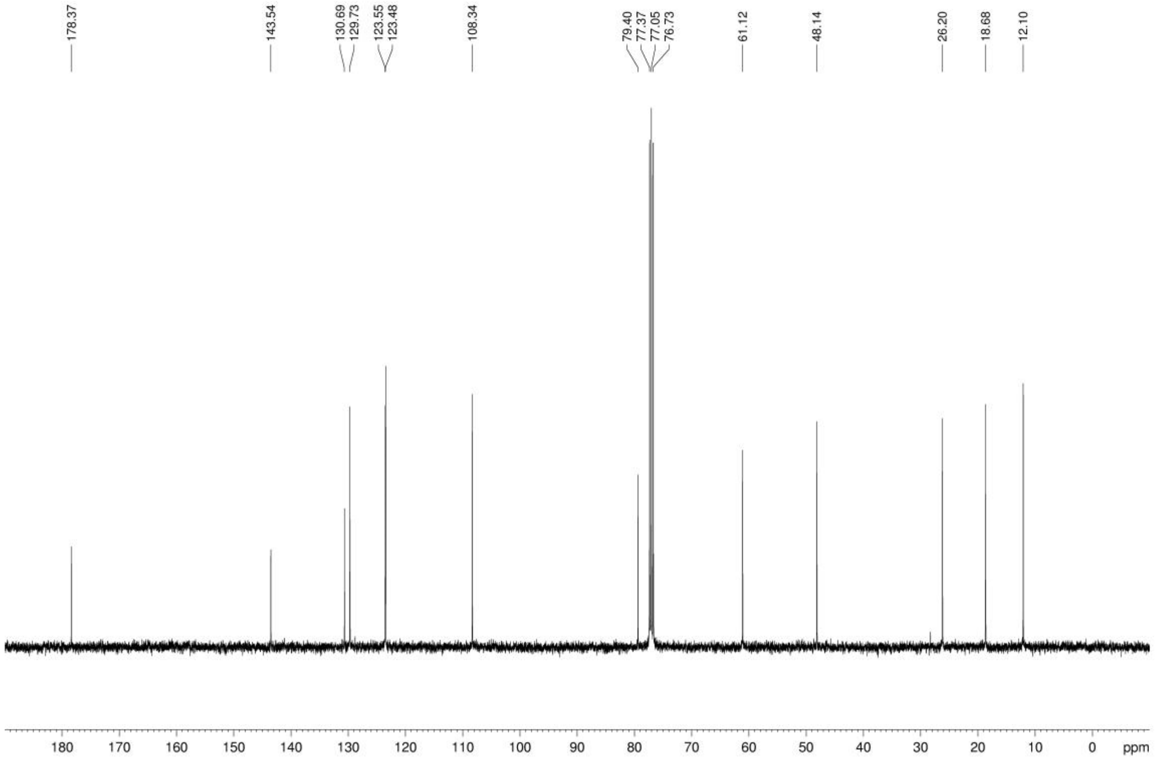

Supplementary Figure 59. ^{1}H and ^{13}C-NMR Spectrum for (*R,R*)-**11a.**

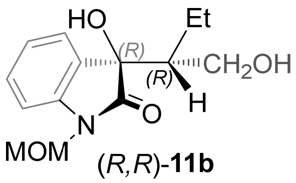

(*R,R*)-**11b**

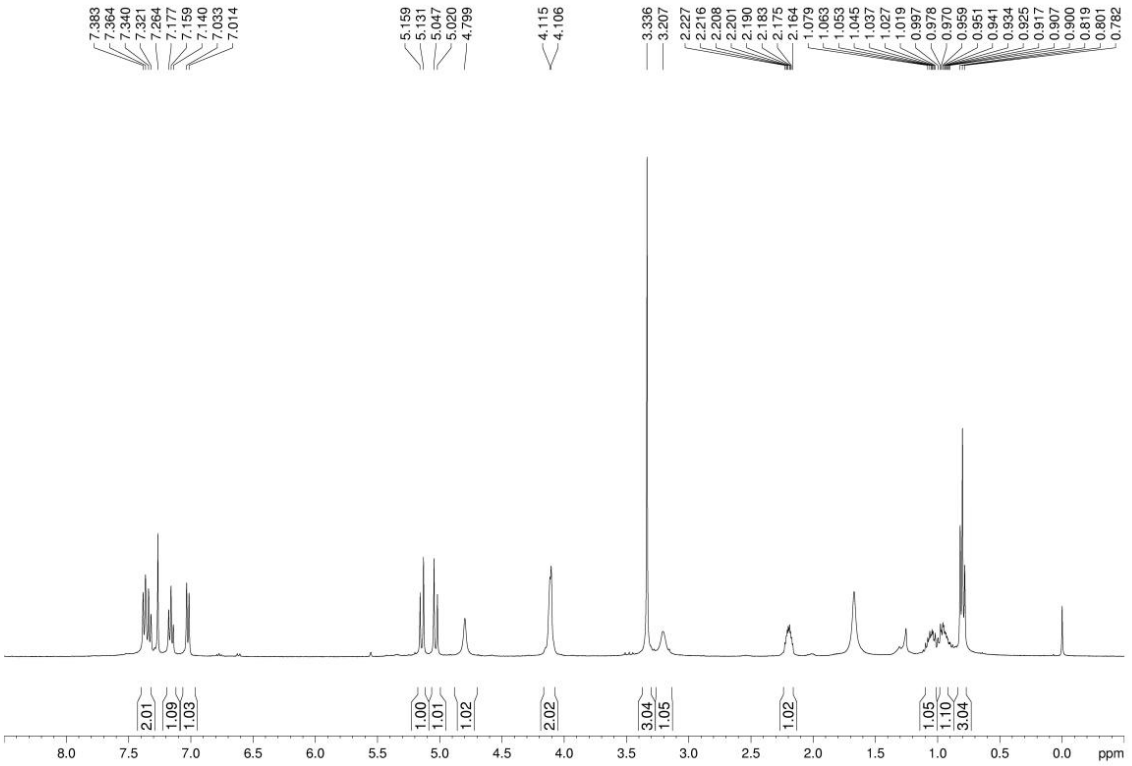

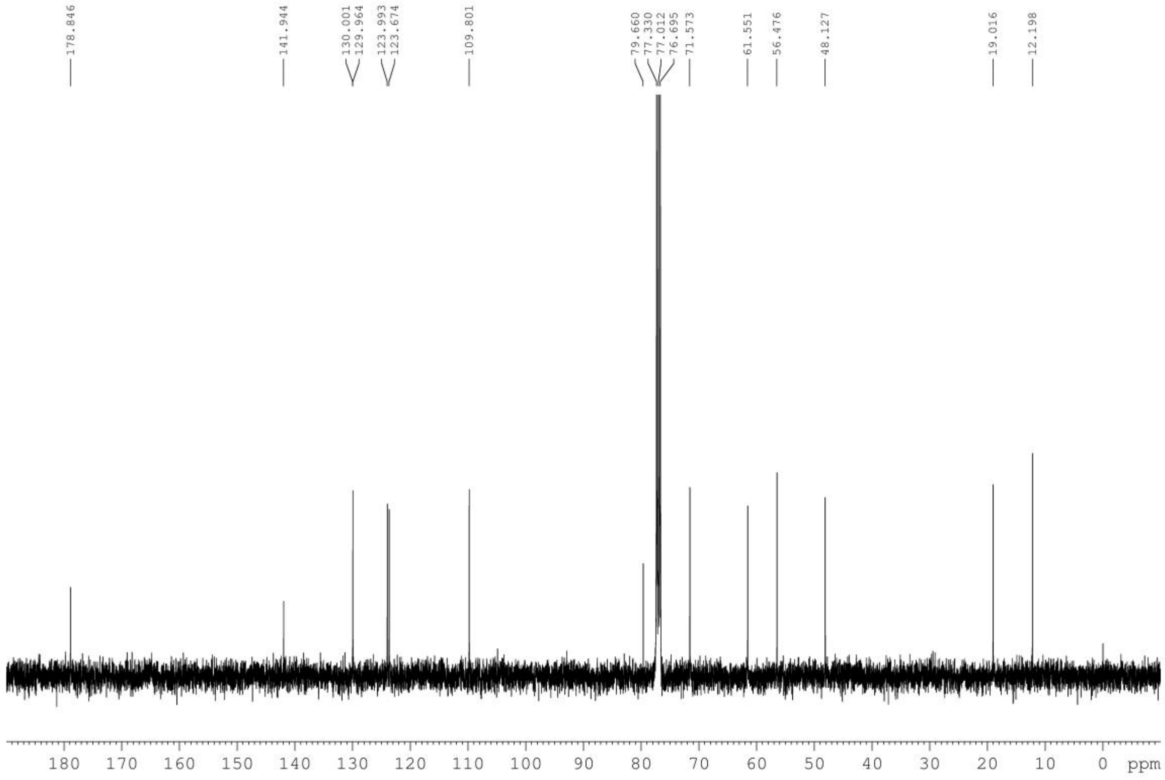

Supplementary Figure 60. ^{1}H and ^{13}C-NMR Spectrum for (*R,R*)**-11b.**

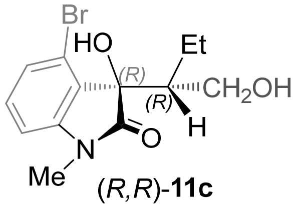

(*R*,*R*)-**11c**

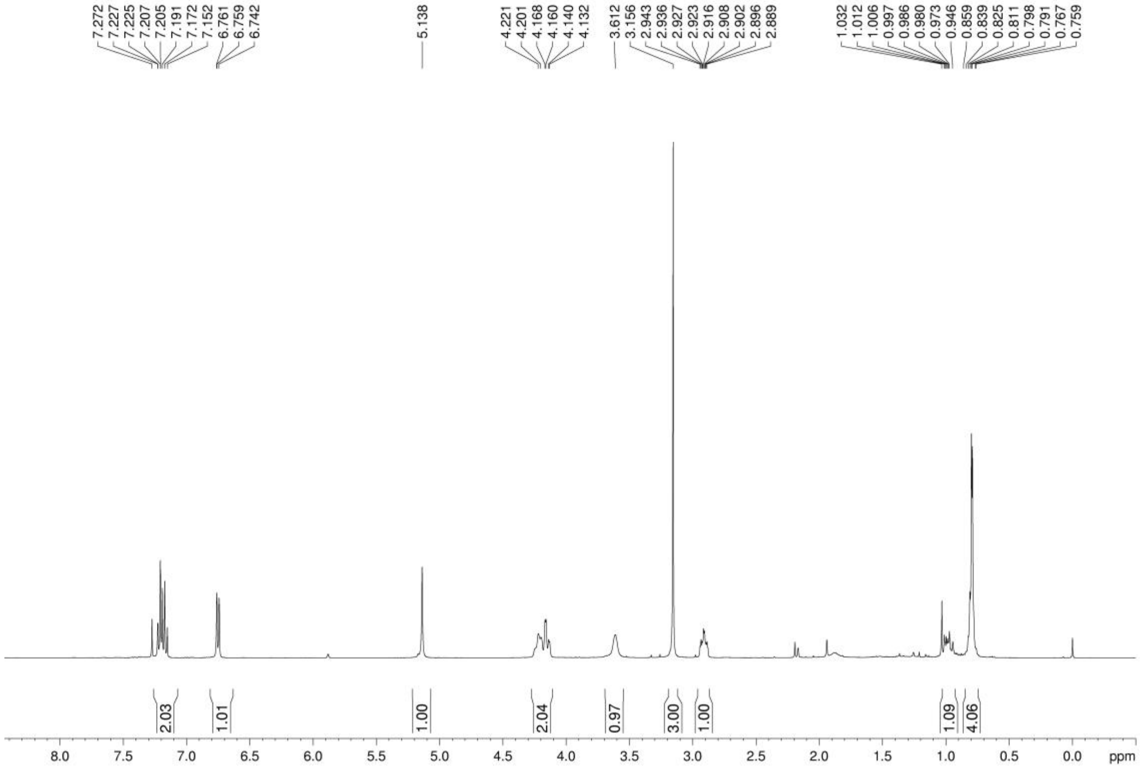

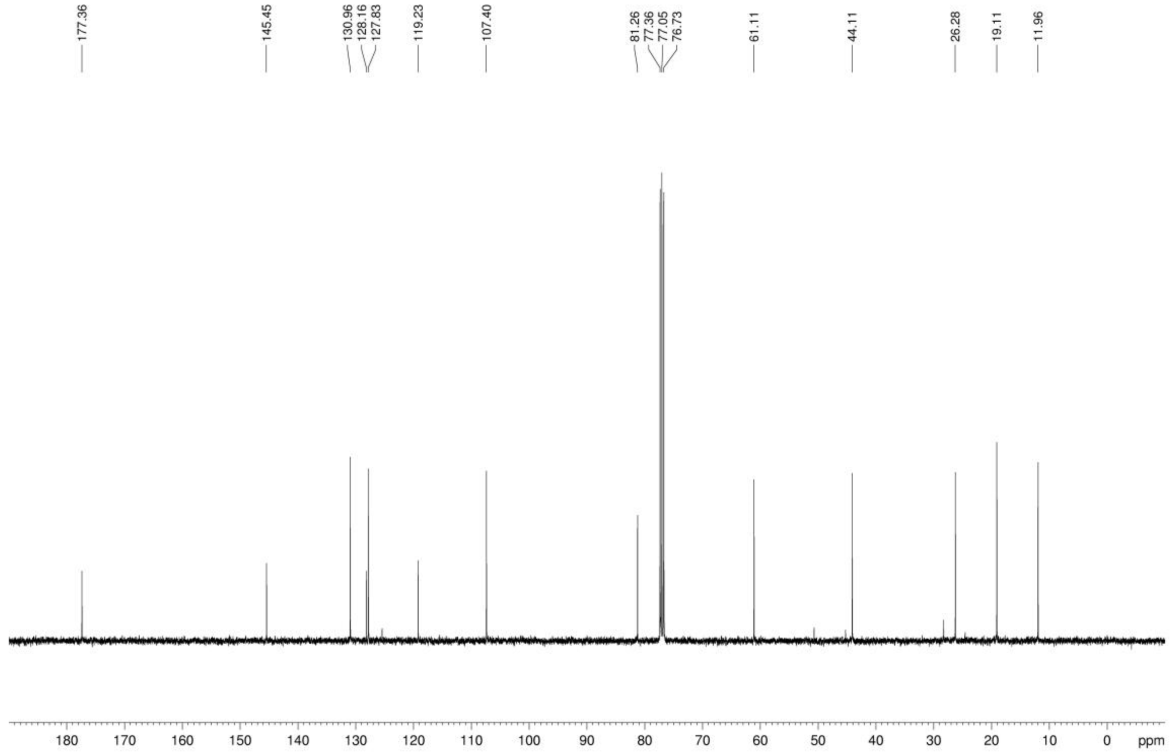

Supplementary Figure 61. ^{1}H and ^{13}C-NMR Spectrum for (*R,R*)-**11c.**

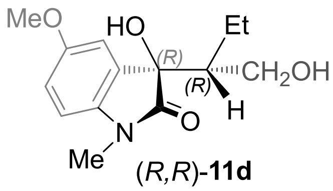

(*R,R*)-**11d**

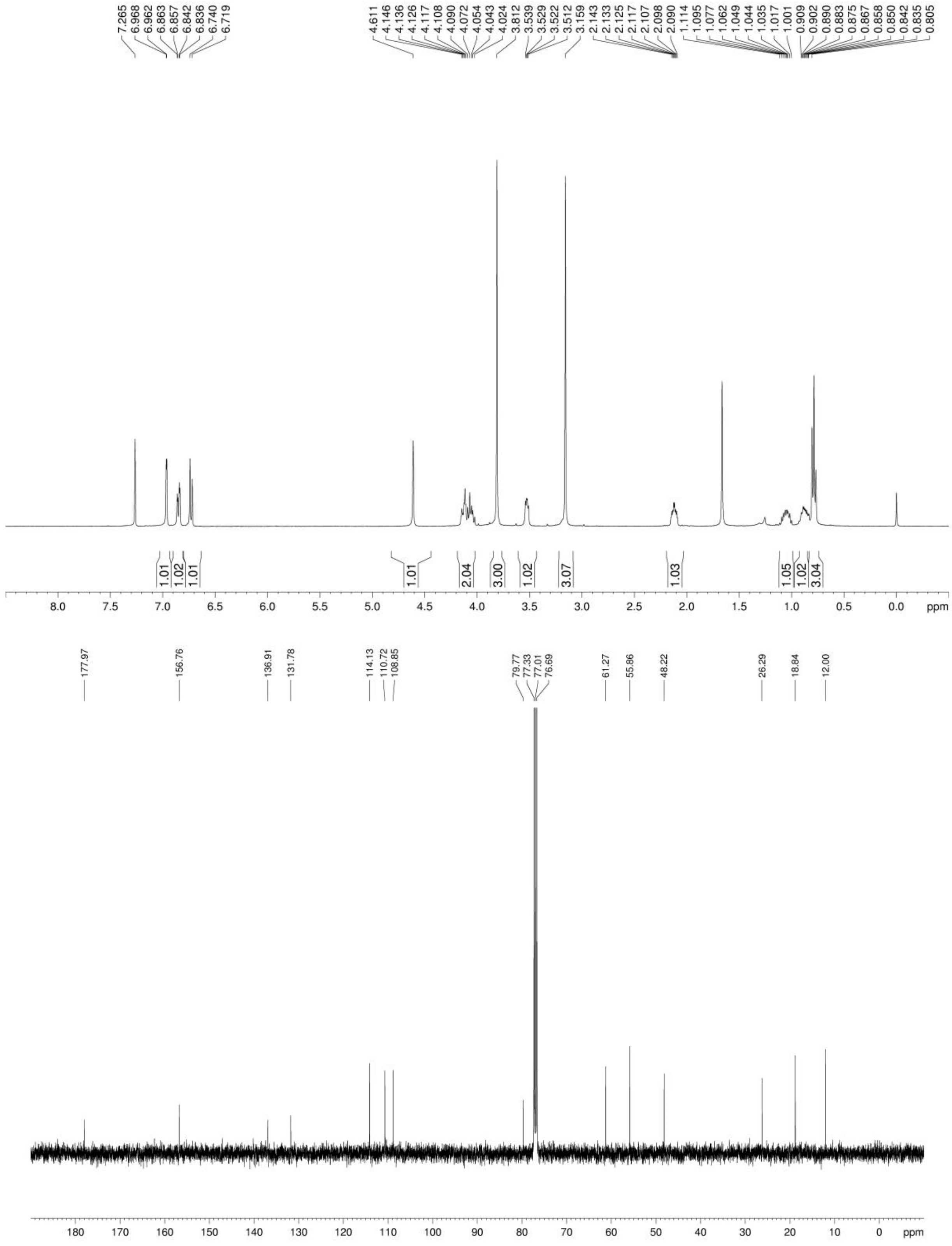

Supplementary Figure 62. ^{1}H and ^{13}C-NMR Spectrum for (*R,R*)-**11d.**

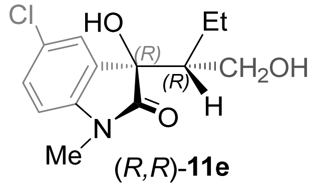

(*R*,*R*)-**11e**

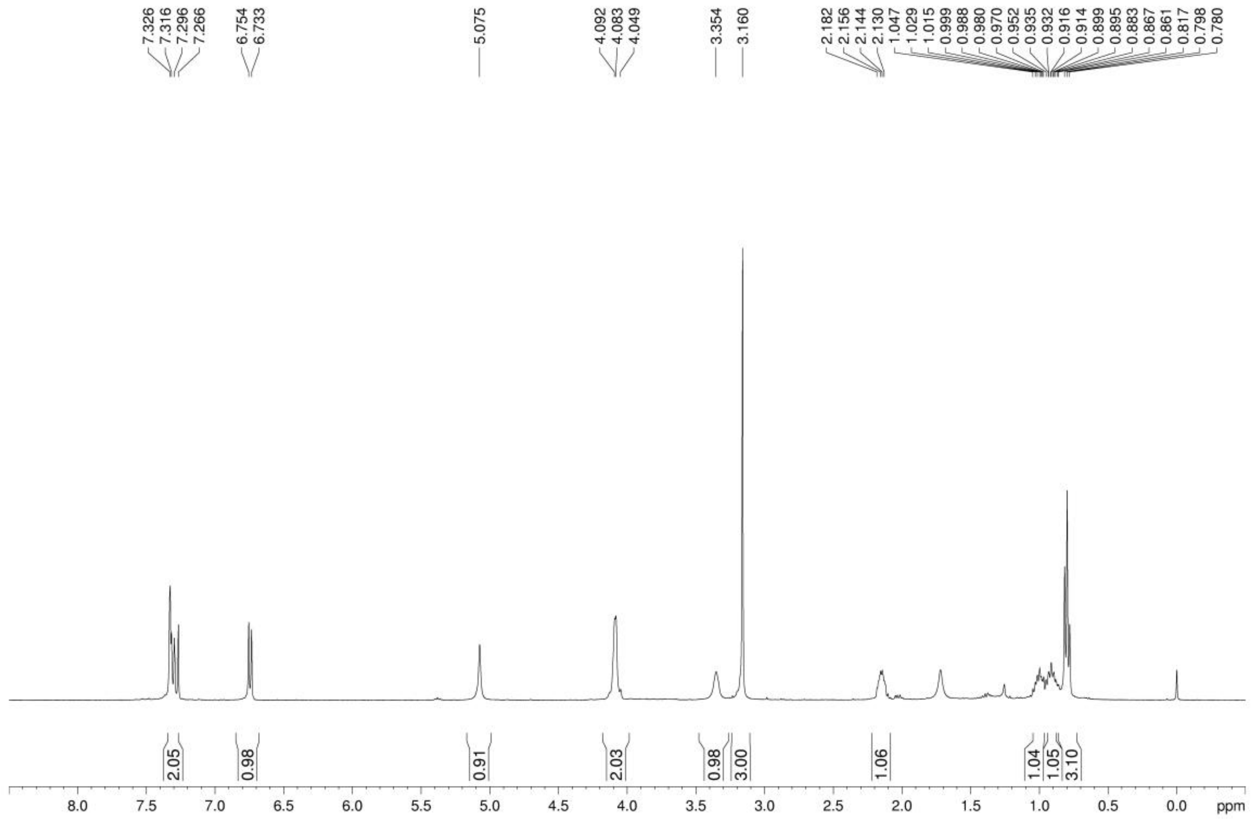

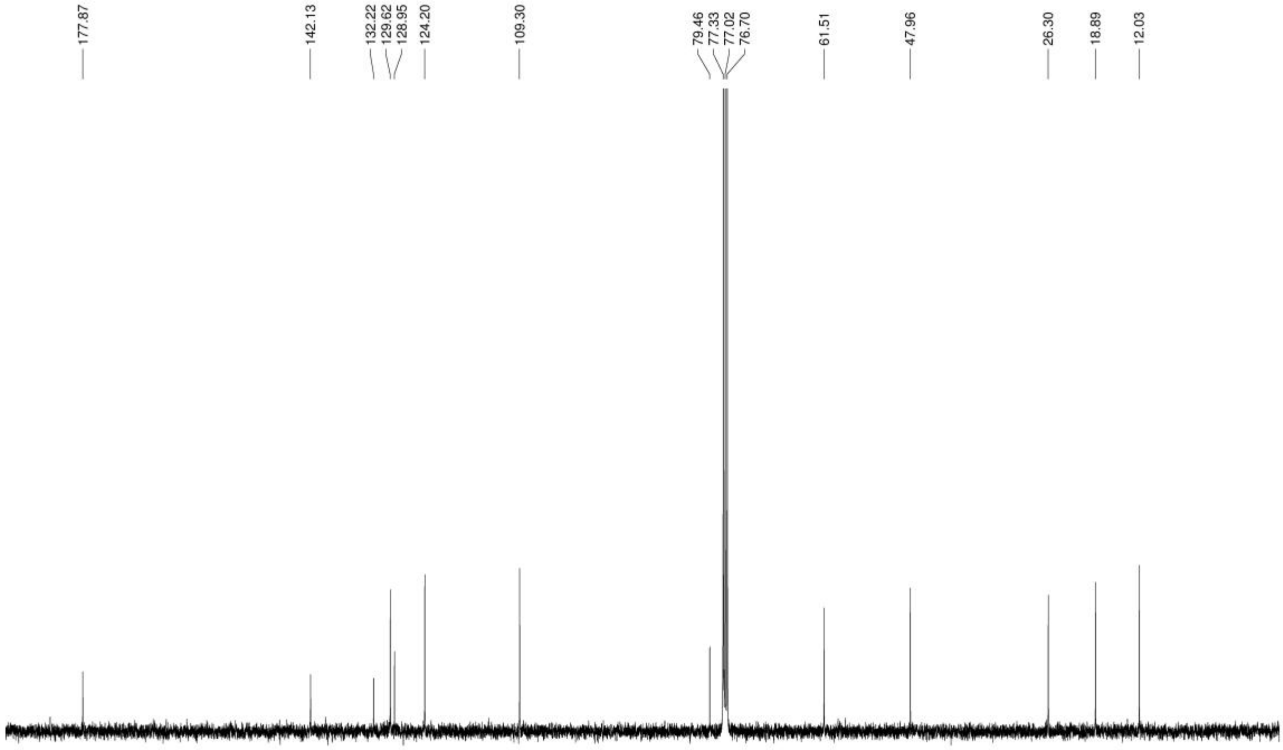

Supplementary Figure 63. ^{1}H and ^{13}C-NMR Spectrum for (*R*,*R*)-**11e.**

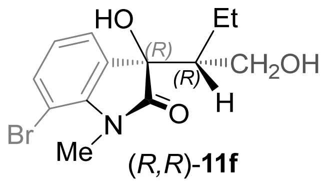

(*R,R*)-**11f**

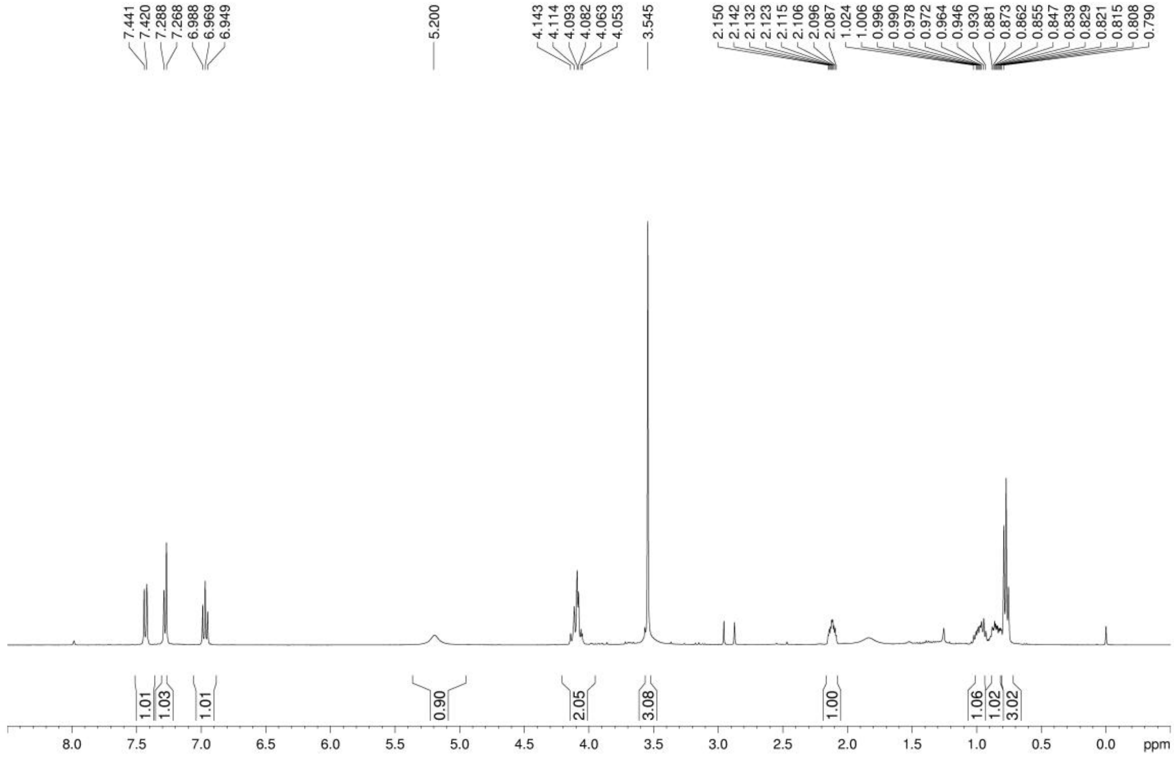

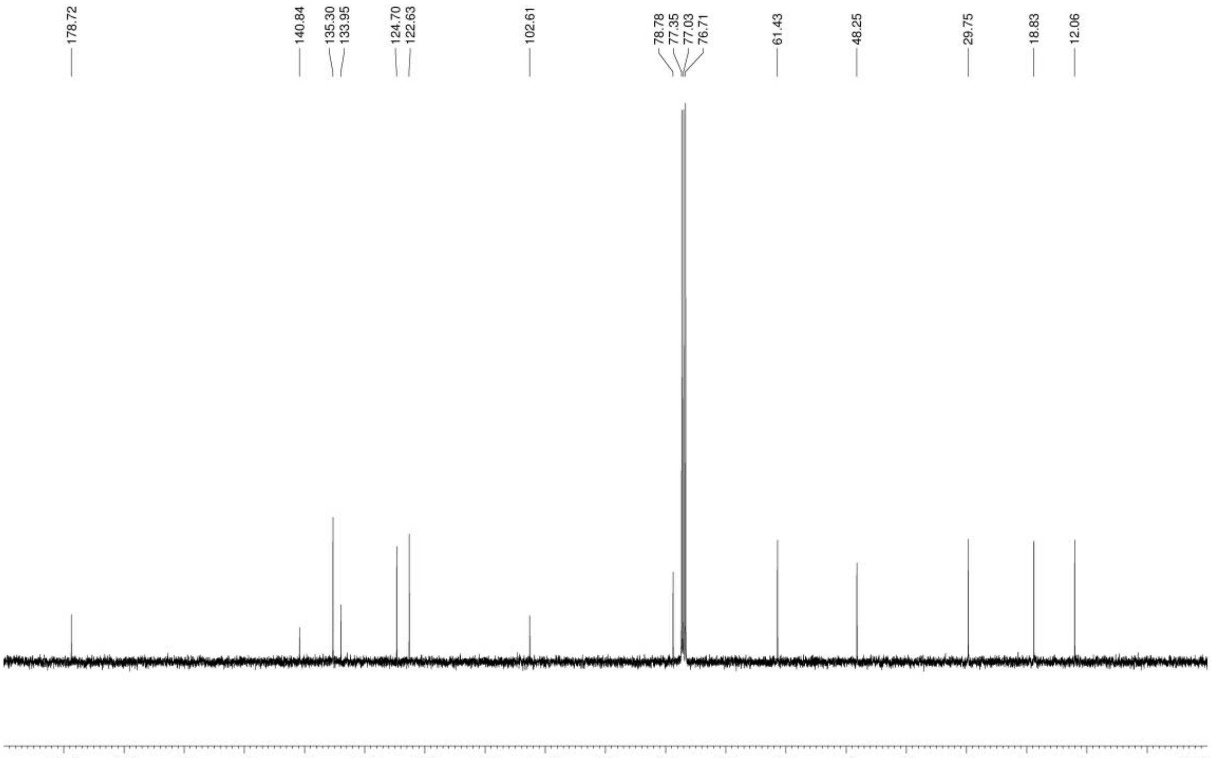

Supplementary Figure 64. ^{1}H and ^{13}C-NMR Spectrum for (*R,R*)-**11f.**

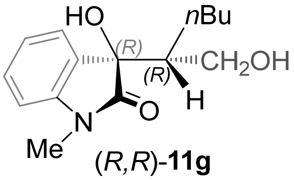

(*R*,*R*)-**11g**

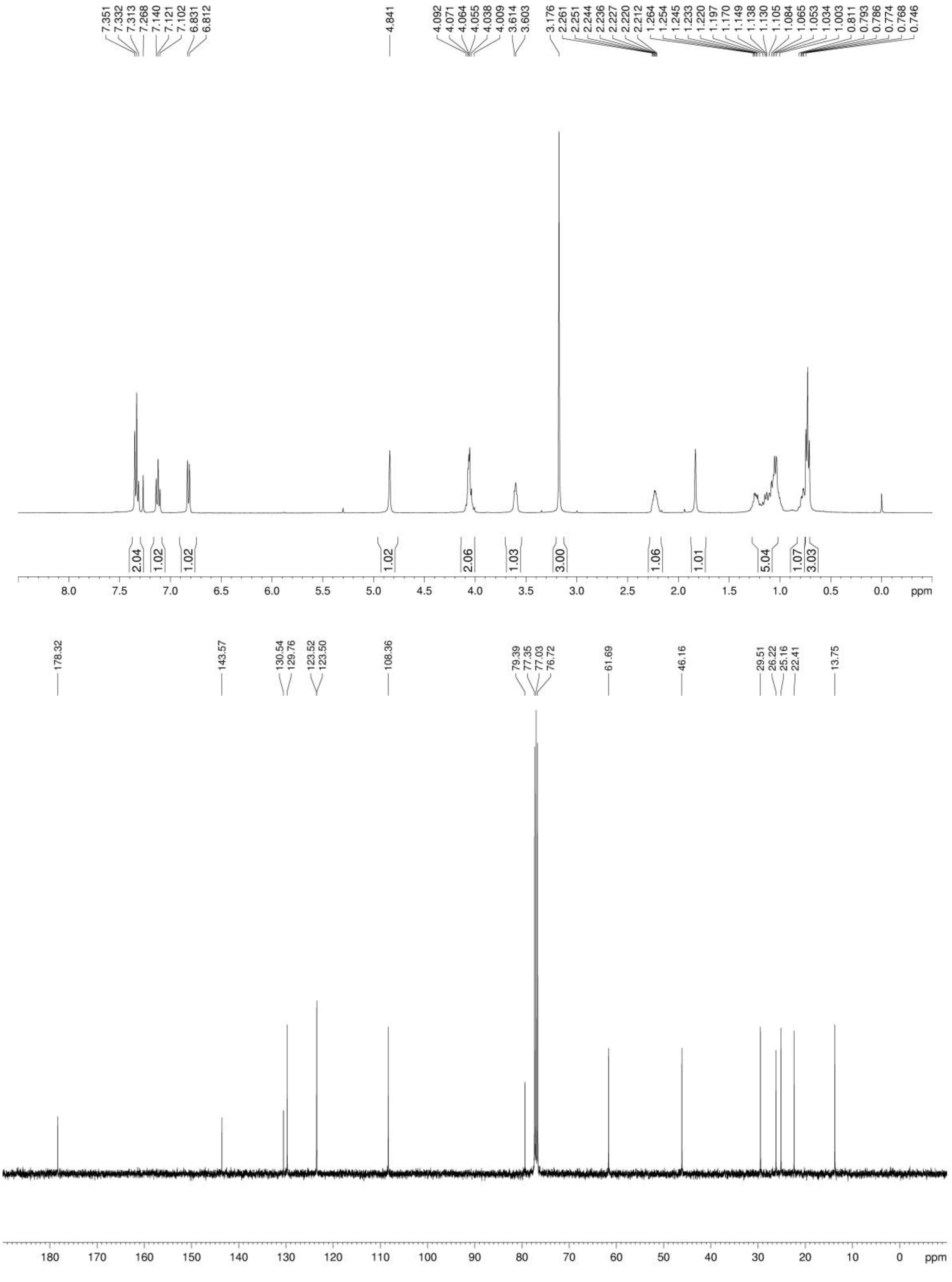

Supplementary Figure 65. ^{1}H and ^{13}C-NMR Spectrum for (*R,R*)-**11g.**

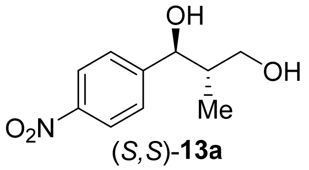

(*S*,*S*)-**13a**

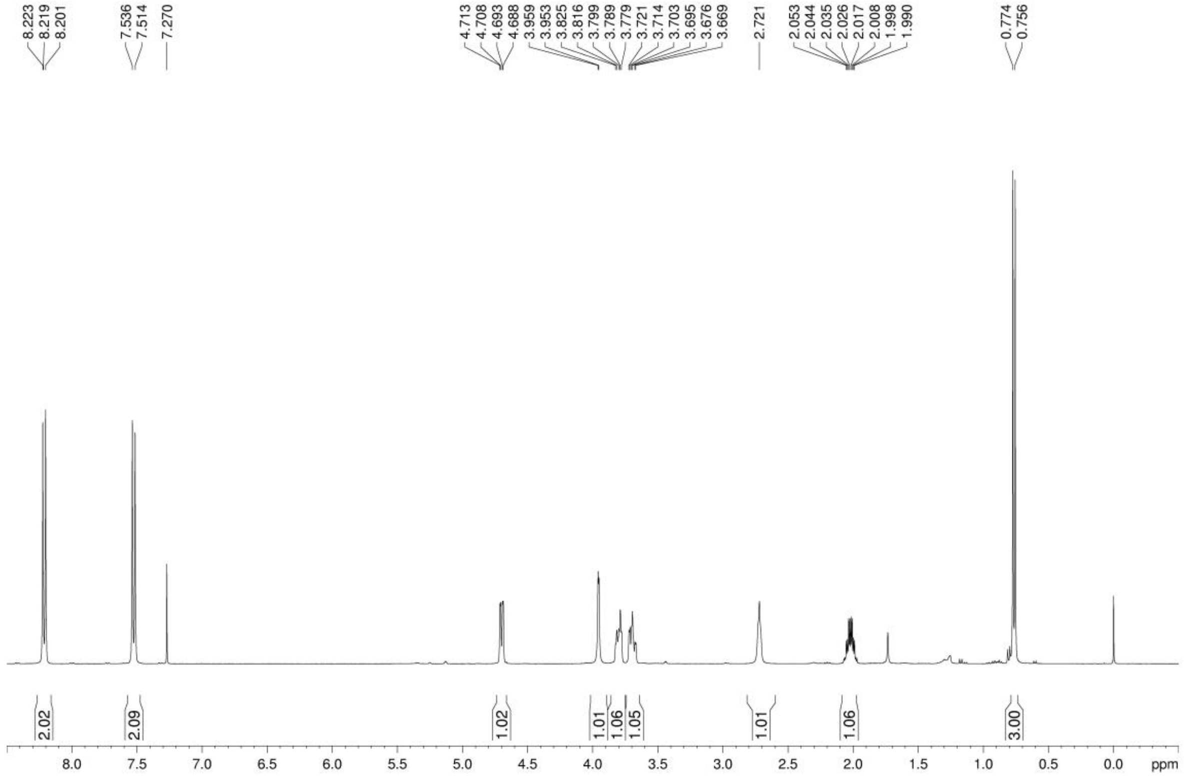

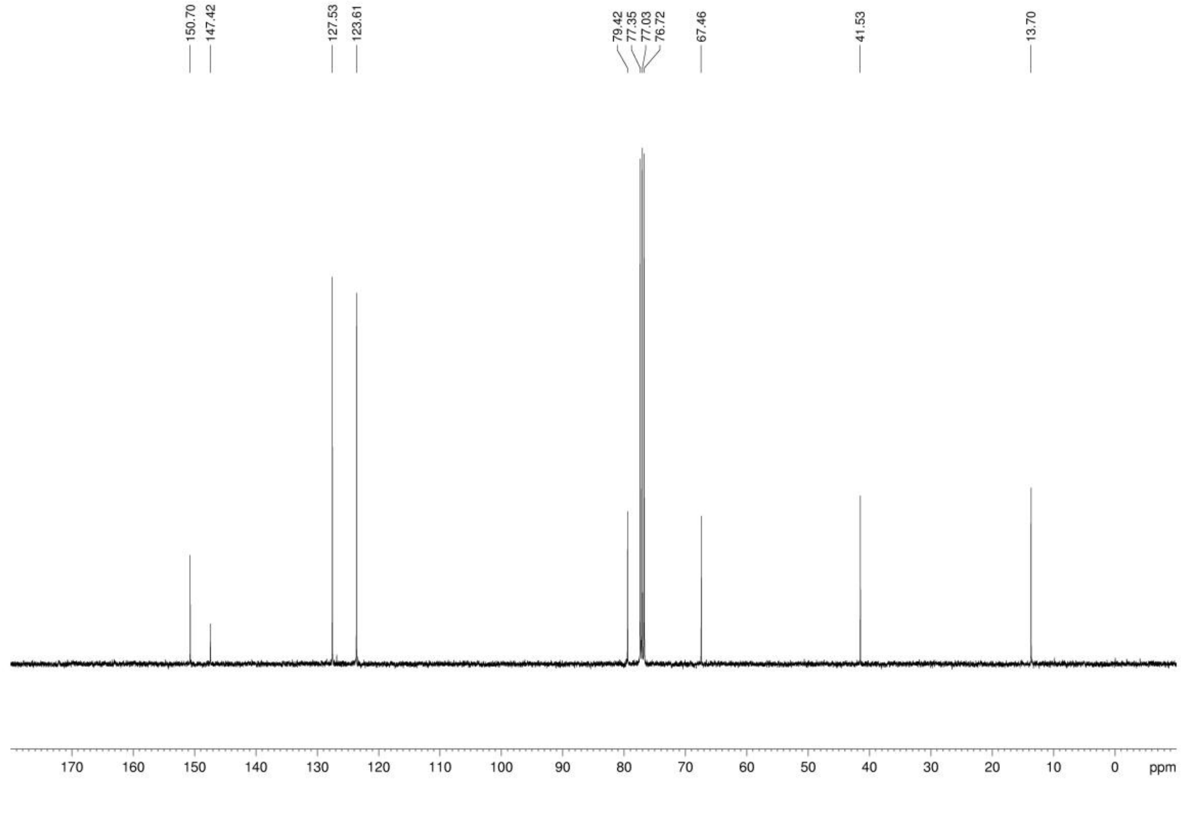

Supplementary Figure 66. ^{1}H and ^{13}C-NMR Spectrum for (*S,S*)-**13a.**

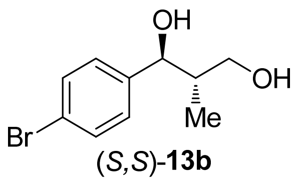

(*S*,*S*)-**13b**

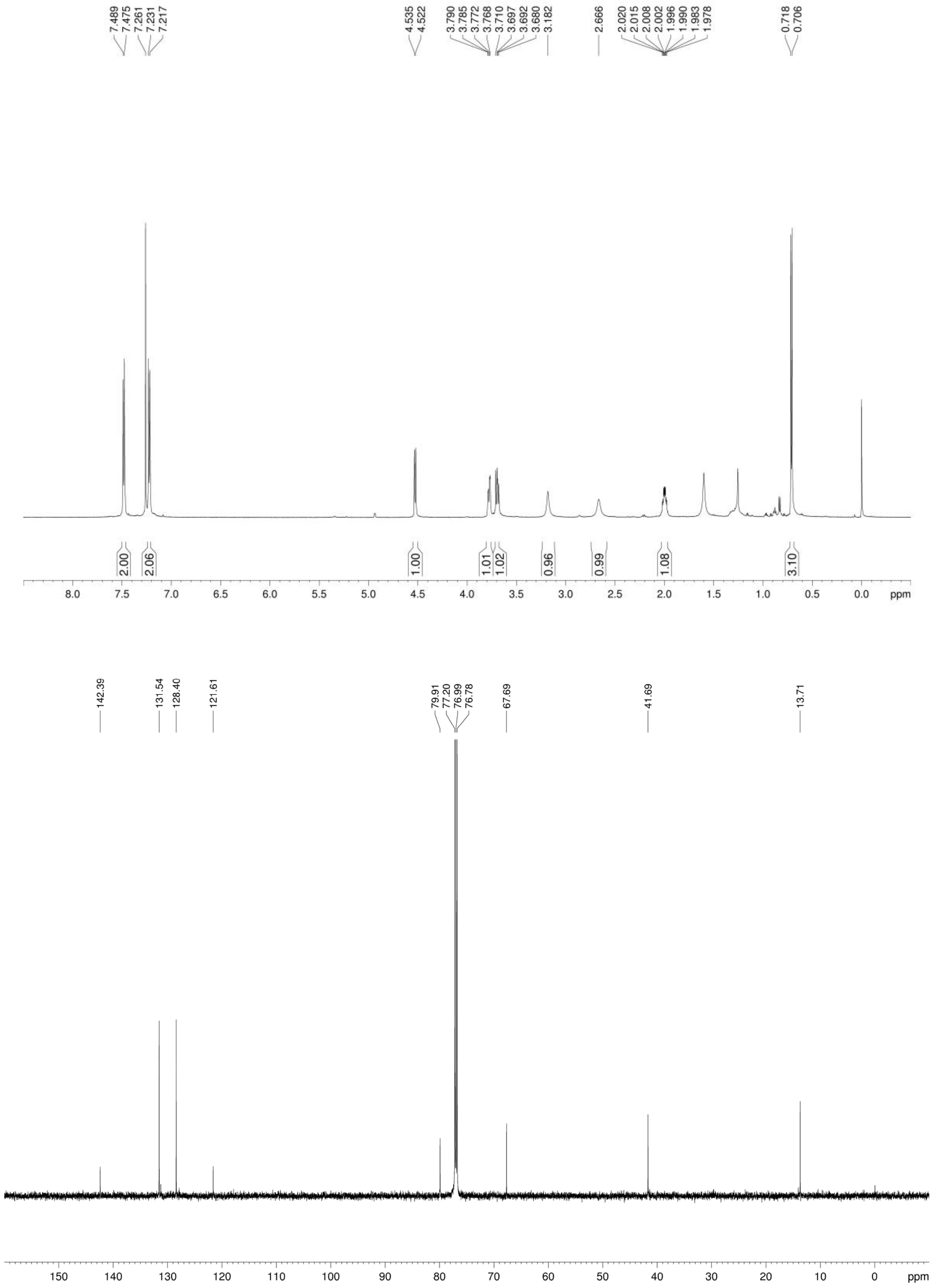

Supplementary Figure 67. ^{1}H and ^{13}C-NMR Spectrum for (*S,S*)-**13b.**

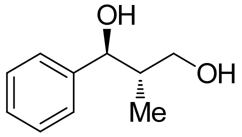

(*S*,*S*)-**13c**

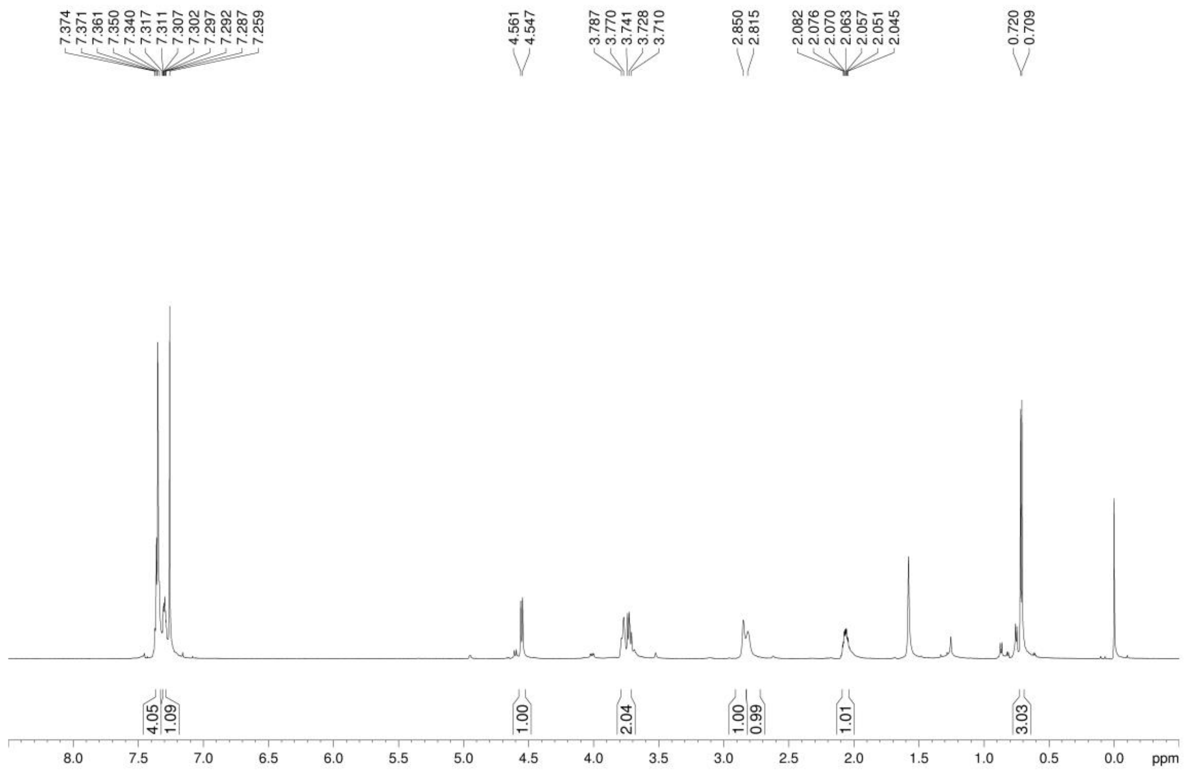

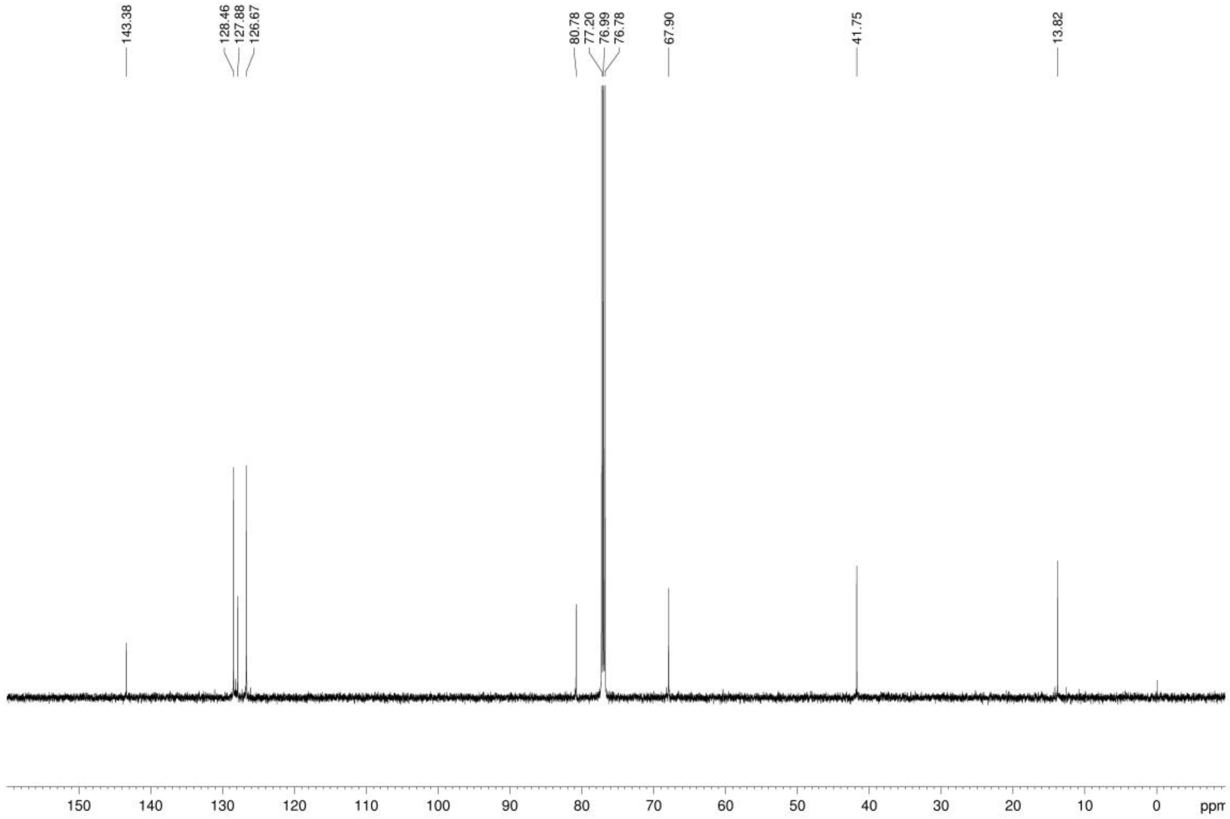

Supplementary Figure 68. ^{1}H and ^{13}C-NMR Spectrum for (*S,S*)-**13c.**

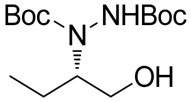

(*S*)-**15a**

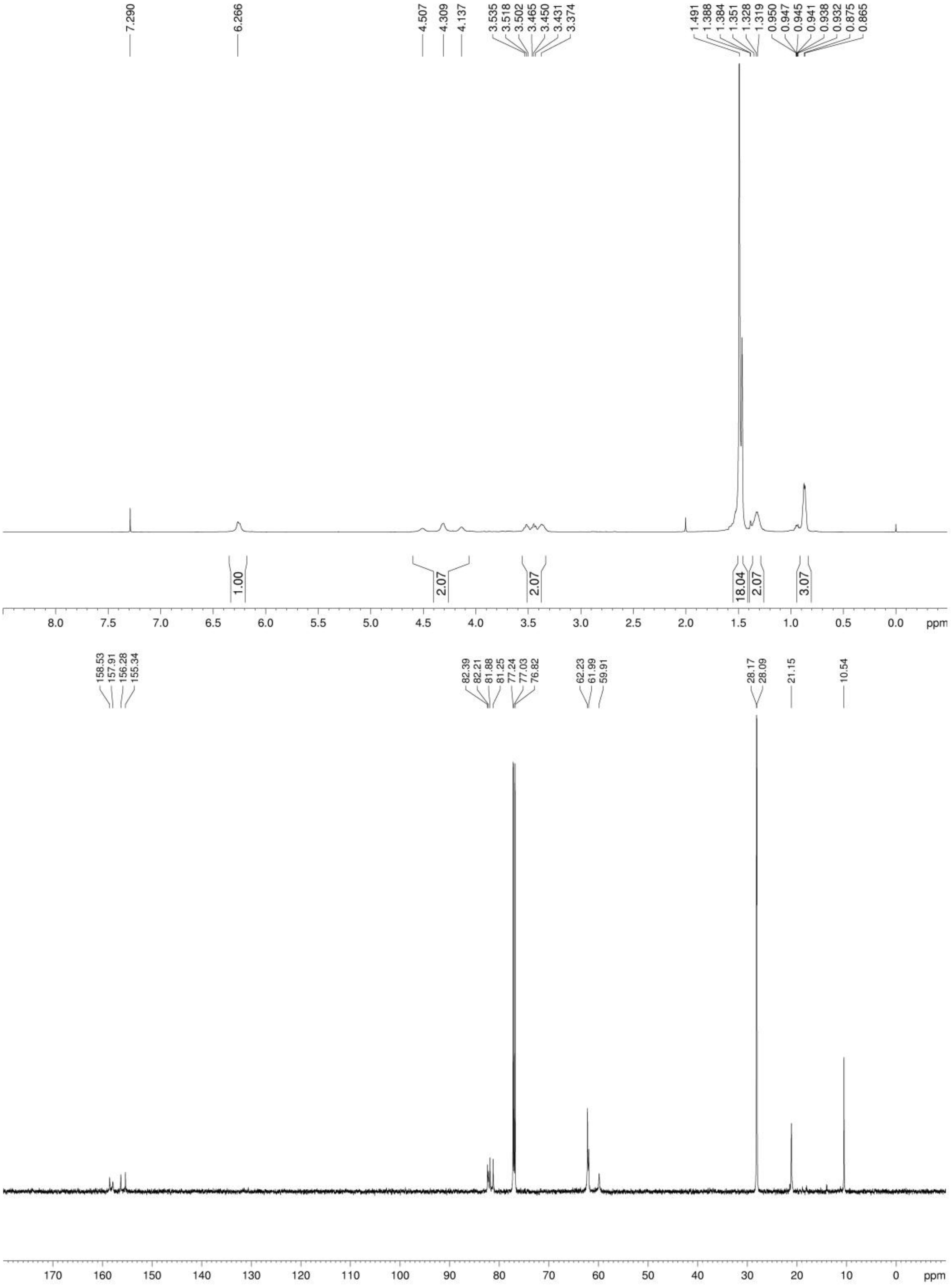

Supplementary Figure 69. ^{1}H and ^{13}C-NMR Spectrum for (*S*)-**15a.**

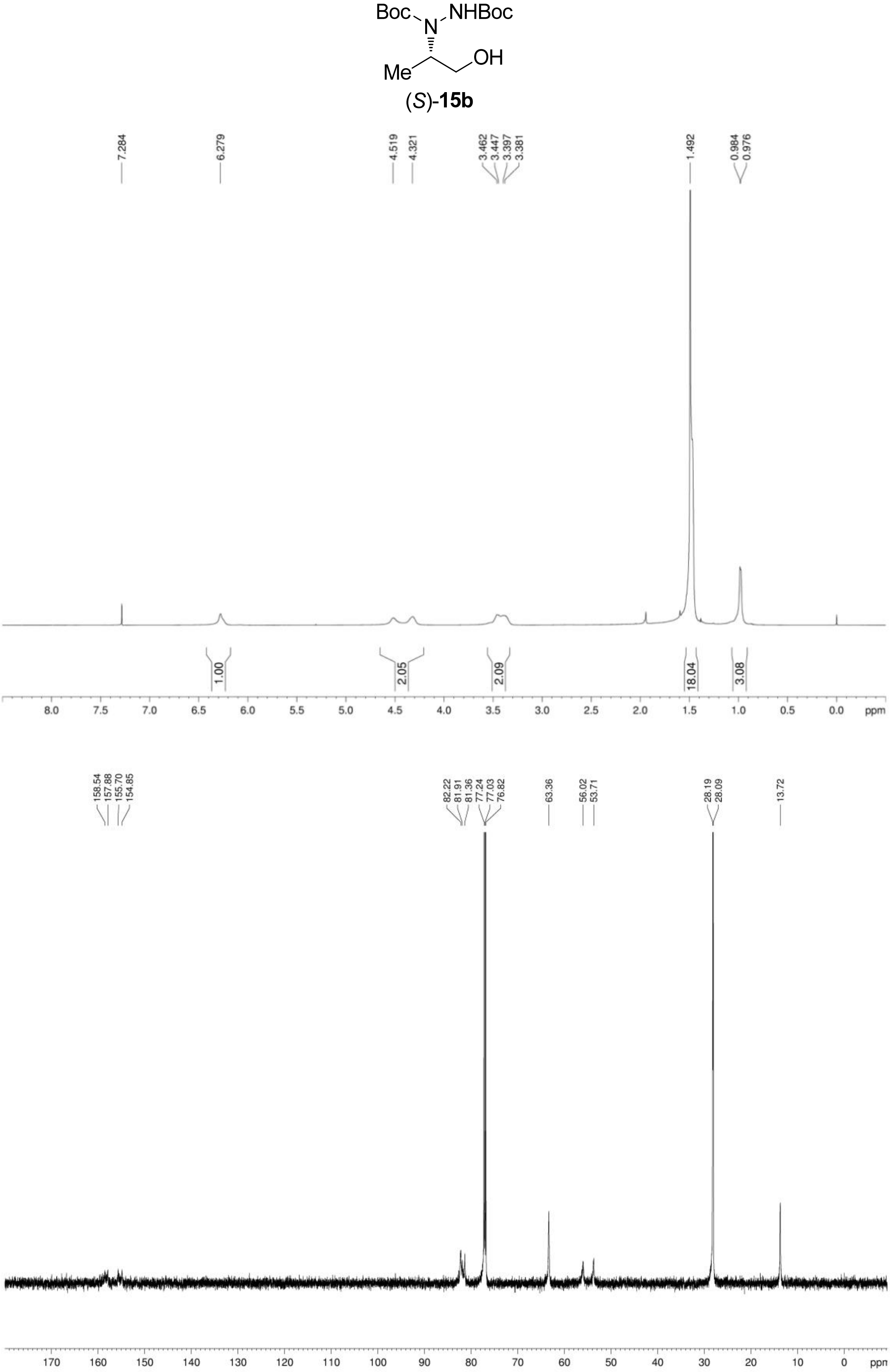

Supplementary Figure 70. ^{1}H and ^{13}C-NMR Spectrum for (*S*)-**15b.**

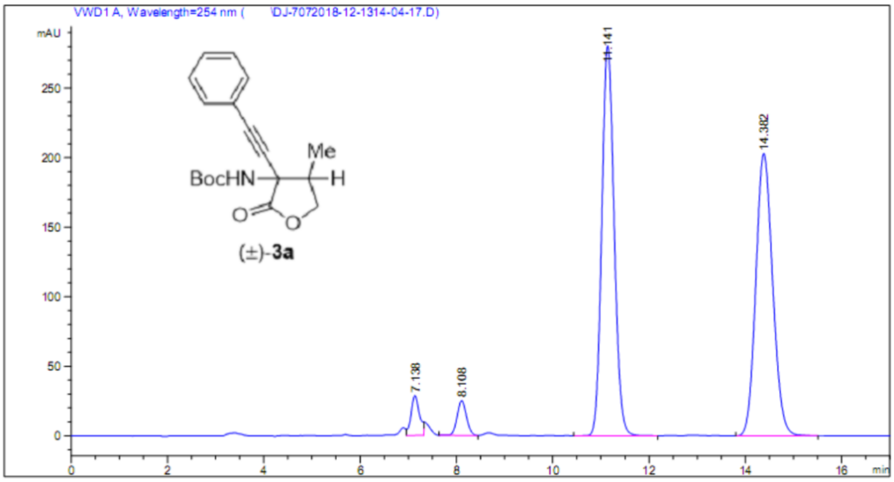

Peak #	RetTime [min]	Type	Width [min]	Area [mAU*s]	Height [mAU]	Area %
1	7.138	VB R	0.2204	485.02829	28.54715	4.5066
2	8.108	VV R	0.2095	336.84583	24.94768	3.1297
3	11.141	BB	0.2793	5007.77148	280.42883	46.5289
4	14.382	BB	0.3735	4933.06689	203.01935	45.8348

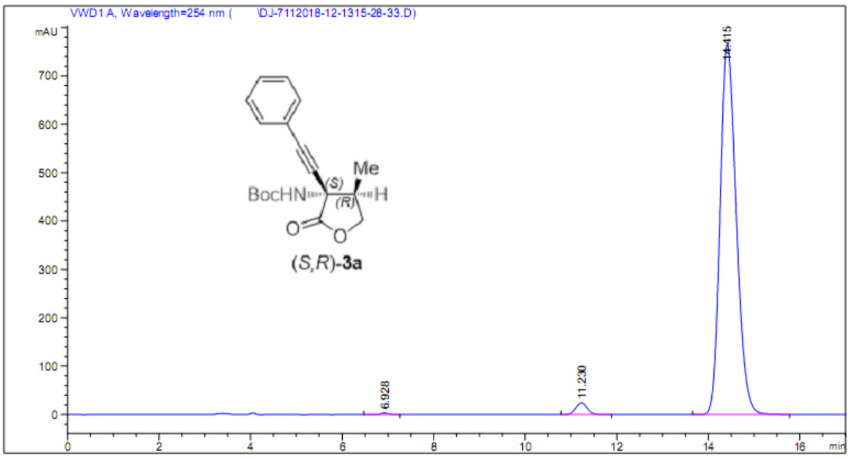

Peak #	RetTime [min]	Type	Width [min]	Area [mAU*s]	Height [mAU]	Area %
1	6.928	BB	0.1625	38.55656	3.64129	0.2002
2	11.230	BB	0.2767	427.17526	23.99775	2.2185
3	14.415	BB	0.3823	1.87896e4	765.55719	97.5813

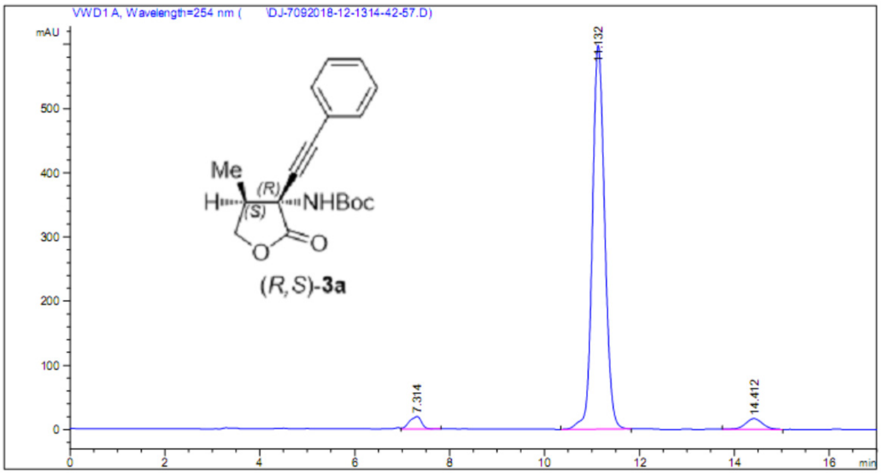

Peak #	RetTime [min]	Type	Width [min]	Area [mAU*s]	Height [mAU]	Area %
1	7.314	VV R	0.2529	367.81561	19.41552	3.1861
2	11.132	BB	0.2801	1.07639e4	597.70715	93.2405
3	14.412	BB	0.3848	412.51556	16.66430	3.5733

Supplementary Figure 71. HPLC Spectrum for **3a.**

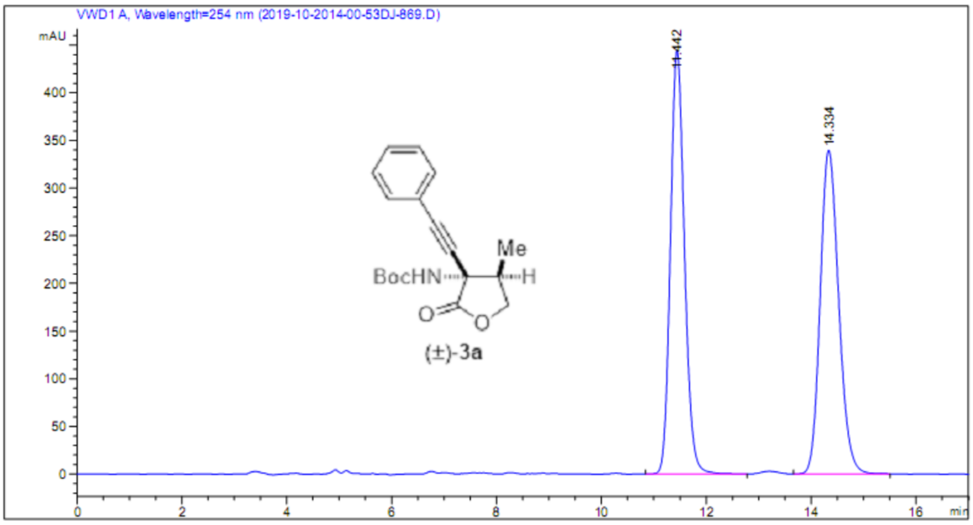

Peak #	RetTime [min]	Type	Width [min]	Area [mAU*s]	Height [mAU]	Area %
1	11.442	BB	0.2867	8262.87500	444.79343	49.8944
2	14.334	BB	0.3800	8297.86719	339.62720	50.1056

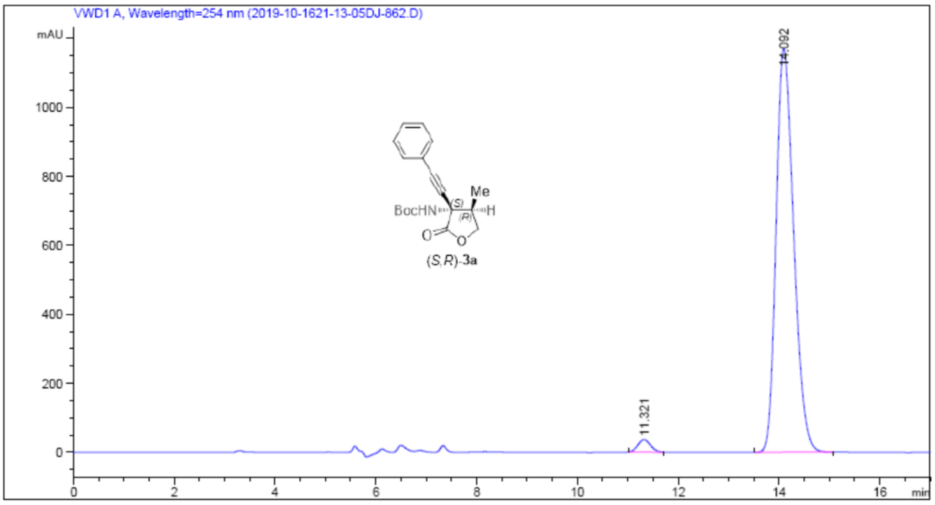

Peak #	RetTime [min]	Type	Width [min]	Area [mAU*s]	Height [mAU]	Area %
1	11.321	MM	0.2905	637.13983	36.54974	2.1948
2	14.092	BB	0.3788	2.83919e4	1171.22192	97.8052

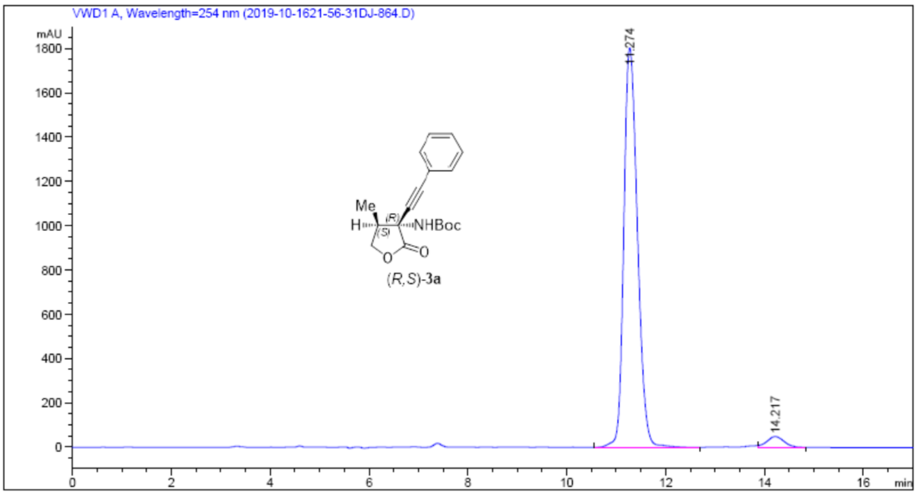

Peak #	RetTime [min]	Type	Width [min]	Area [mAU*s]	Height [mAU]	Area %
1	11.274	BB	0.2984	3.45723e4	1805.84009	96.7212
2	14.217	FM	0.4015	1171.97070	48.65414	3.2788

Supplementary Figure 72. HPLC Spectrum for the Major Diastereomer of **3a.**

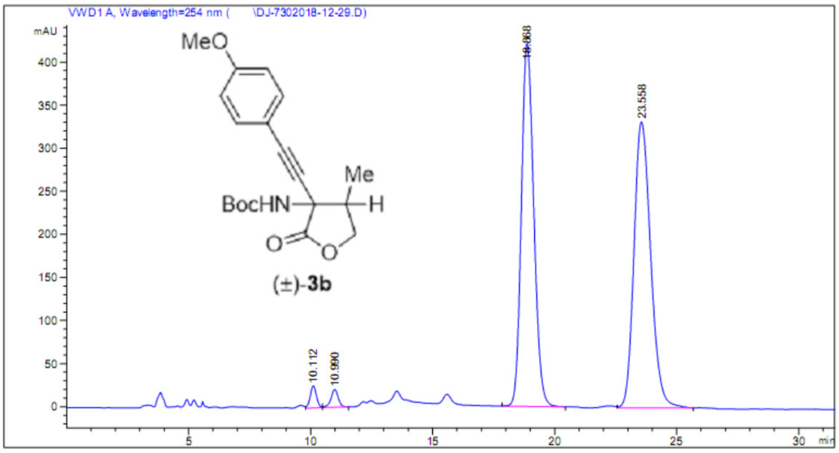

Peak #	RetTime [min]	Type	Width [min]	Area [mAU*s]	Height [mAU]	Area %
1	10.112	VV R	0.2725	495.45087	25.27986	1.5668
2	10.990	VB	0.3136	423.68051	20.54357	1.3398
3	18.868	BB	0.5502	1.48762e4	421.00455	47.0443
4	23.558	VB R	0.7401	1.58264e4	331.62979	50.0491

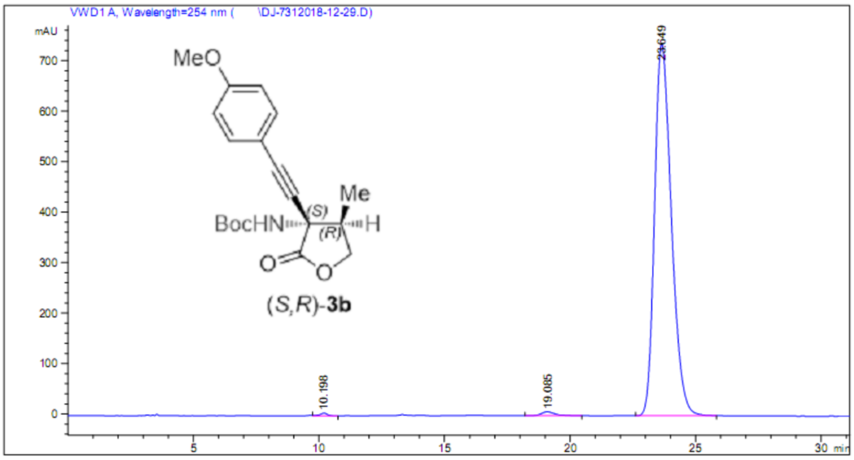

Peak #	RetTime [min]	Type	Width [min]	Area [mAU*s]	Height [mAU]	Area %
1	10.198	VB	0.3300	133.13031	5.81787	0.3832
2	19.085	BB	0.5951	308.72989	7.72693	0.8887
3	23.649	BB	0.7263	3.42967e4	736.50568	98.7280

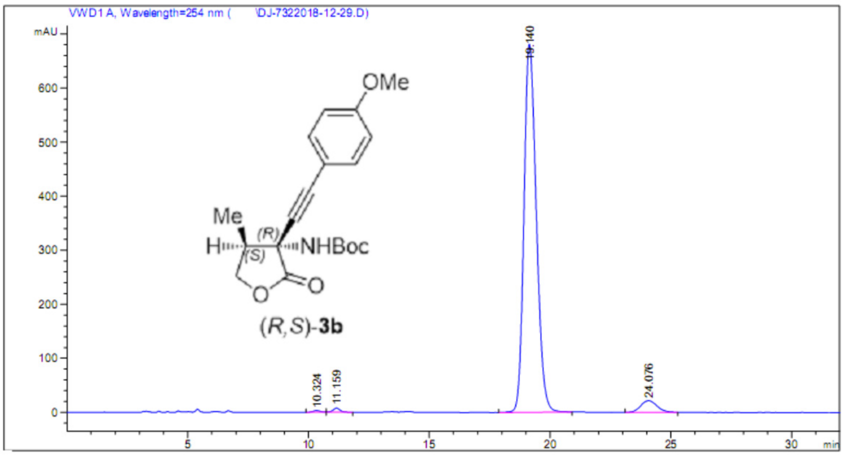

Peak #	RetTime [min]	Type	Width [min]	Area [mAU*s]	Height [mAU]	Area %
1	10.324	BB	0.3234	56.98924	2.74343	0.2248
2	11.159	BB	0.3041	148.53561	7.46686	0.5860
3	19.140	BB	0.5491	2.41238e4	681.23456	95.1716
4	24.076	BB	0.7440	1018.36938	21.60278	4.0176

Supplementary Figure 73. HPLC Spectrum for **3b.**

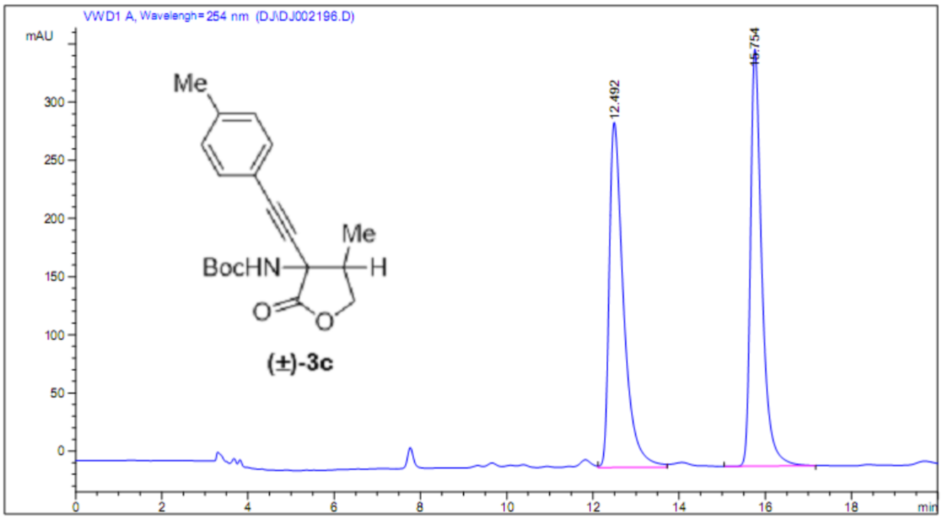

Peak #	RetTime [min]	Type	Width [min]	Area mAU *s	Height [mAU]	Area %
1	12.492	VV	0.3460	6882.02002	297.06097	51.1641
2	15.754	VB	0.2747	6568.84912	358.80588	48.8359

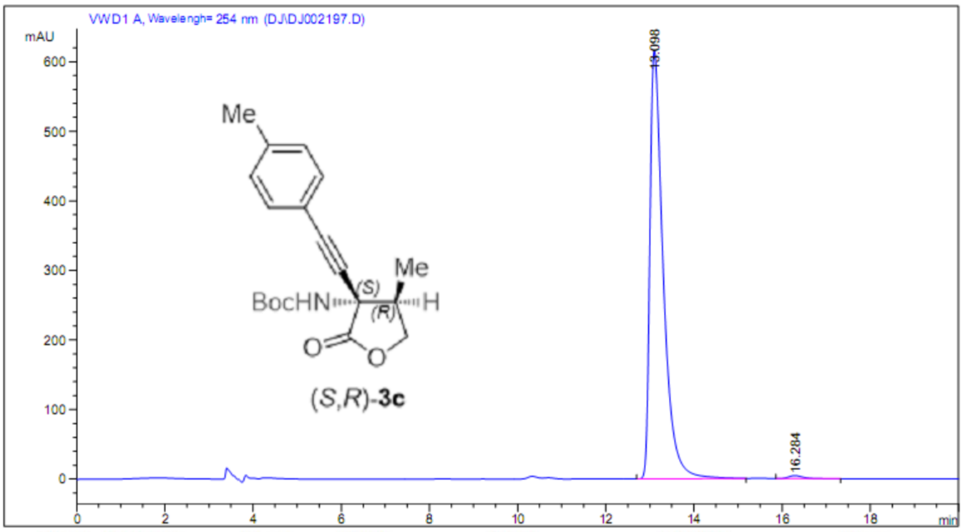

Peak #	RetTime [min]	Type	Width [min]	Area mAU *s	Height [mAU]	Area %
1	13.098	BB	0.3149	1.30139e4	615.95856	99.1443
2	16.284	VB	0.3611	112.31742	4.61560	0.8557

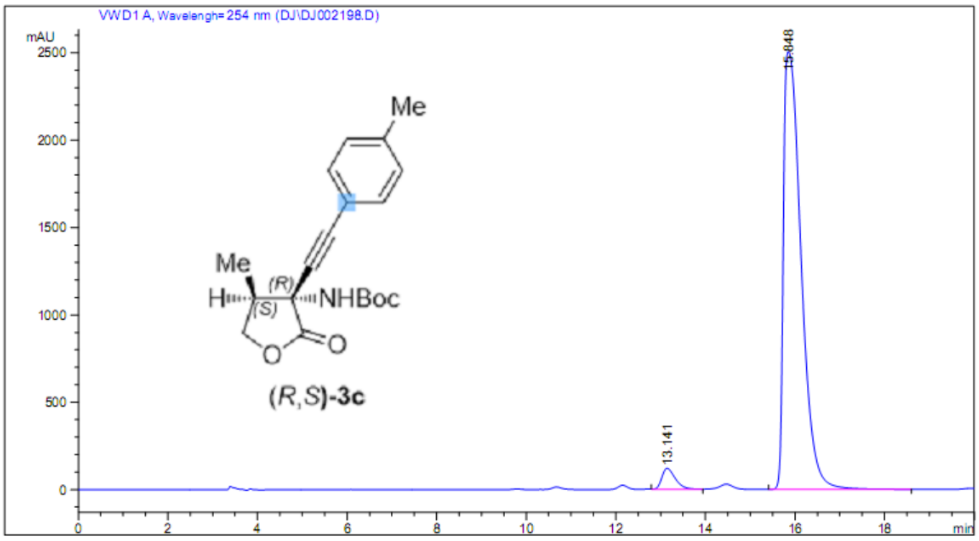

Peak #	RetTime [min]	Type	Width [min]	Area mAU *s	Height [mAU]	Area %
1	13.141	VV	0.3283	2649.66113	122.36133	3.7140
2	15.848	VB	0.4244	6.86929e4	2507.75684	96.2860

Supplementary Figure 74. HPLC Spectrum for **3c.**

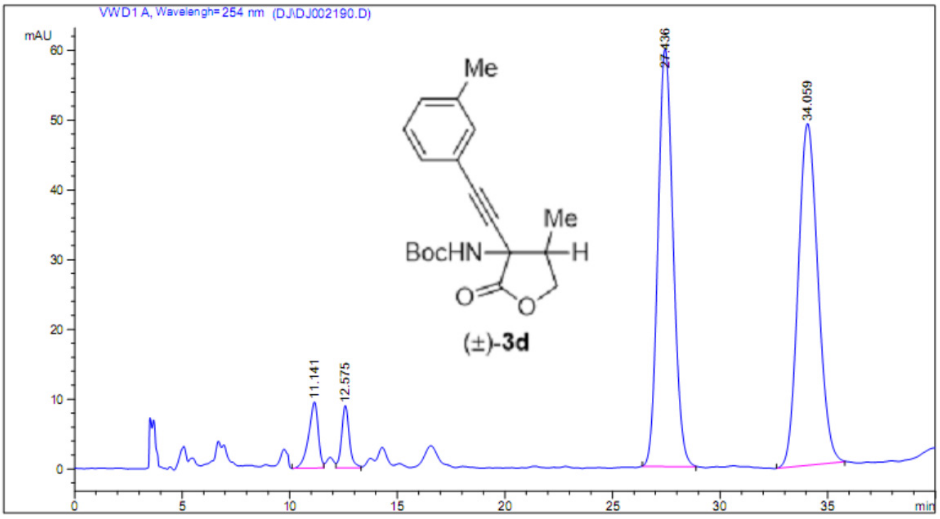

Peak #	RetTime [min]	Type	Width [min]	Area mAU *s	Height [mAU]	Area %
1	11.141	VV	0.4553	296.70929	9.52228	4.4944
2	12.575	VB	0.3764	224.11705	8.95657	3.3948
3	27.436	BB	0.7585	2937.84302	59.90808	44.5005
4	34.059	BB	0.9927	3143.15405	49.01812	47.6104

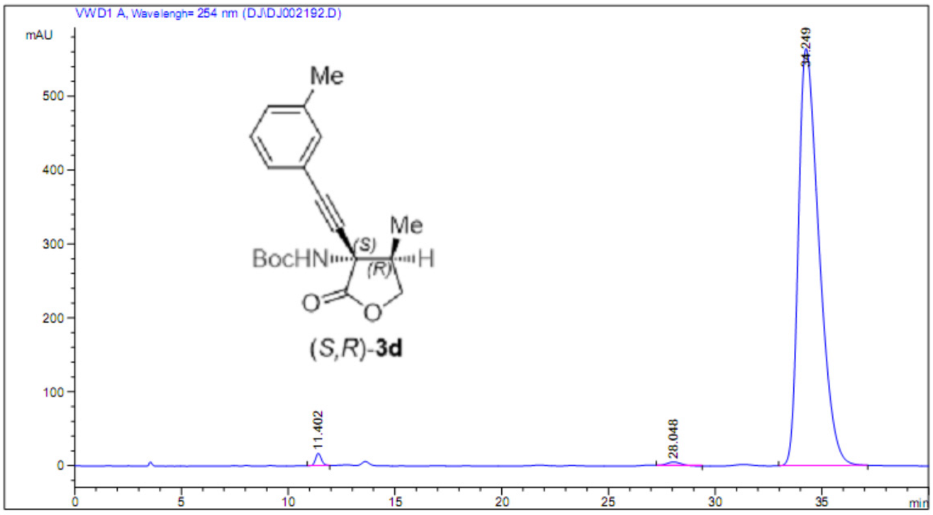

Peak #	RetTime [min]	Type	Width [min]	Area mAU *s	Height [mAU]	Area %
1	11.402	BB	0.2981	321.26422	16.63772	0.8358
2	28.048	VB	0.7470	273.69775	5.36110	0.7120
3	34.249	BB	1.0244	3.78445e4	564.09149	98.4522

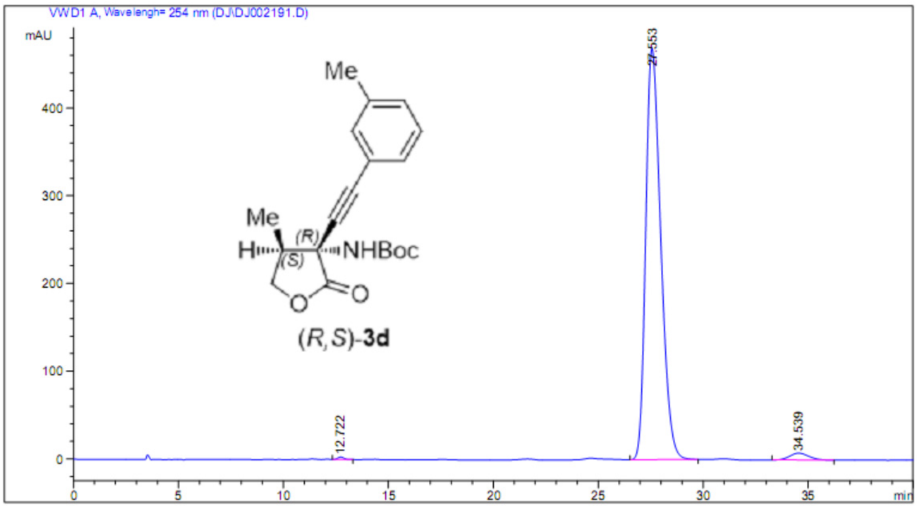

Peak #	RetTime [min]	Type	Width [min]	Area mAU *s	Height [mAU]	Area %
1	12.722	VB	0.3385	62.24638	2.90831	0.2605
2	27.553	BB	0.7662	2.33178e4	469.16528	97.5719
3	34.539	BB	0.9092	518.02893	7.96023	2.1677

Supplementary Figure 75. HPLC Spectrum for **3d.**

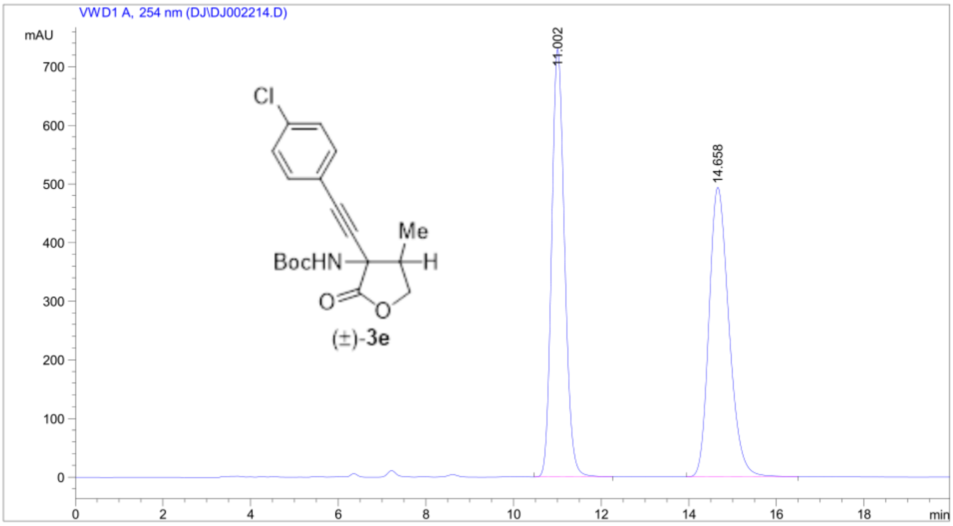

Peak #	RetTime [min]	Type	Width [min]	Area mAU *s	Height [mAU]	Area %
1	11.002	VB	0.3181	1.49951e4	730.66522	49.0294
2	14.658	BB	0.4875	1.55887e4	494.03906	50.9706

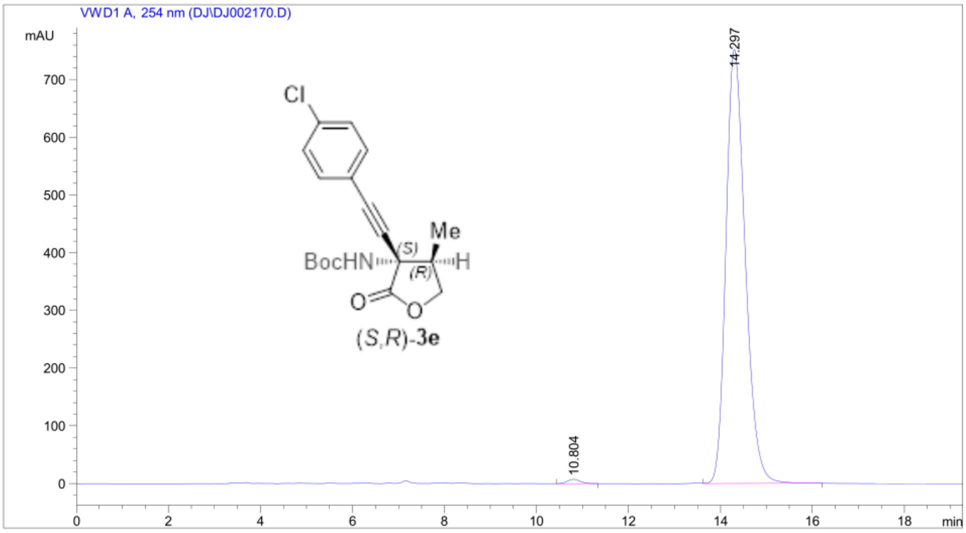

Peak #	RetTime [min]	Type	Width [min]	Area mAU *s	Height [mAU]	Area %
1	10.804	BB	0.3068	146.03923	7.42063	0.6616
2	14.297	VB	0.4492	2.19290e4	752.35840	99.3384

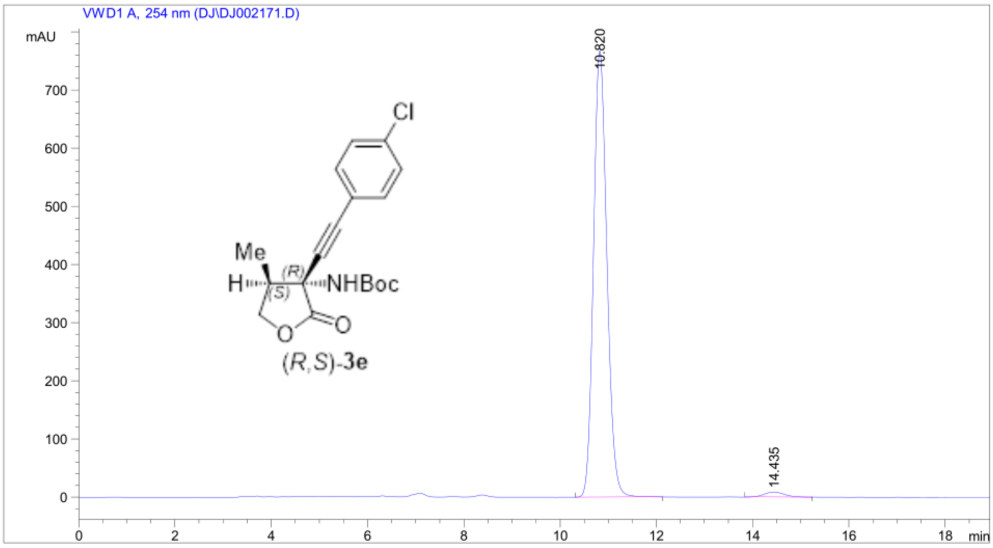

Peak #	RetTime [min]	Type	Width [min]	Area mAU *s	Height [mAU]	Area %
1	10.820	BB	0.2997	1.48278e4	767.25391	98.2592
2	14.435	VB	0.4648	262.69565	8.87212	1.7408

Supplementary Figure 76. HPLC Spectrum for **3e.**

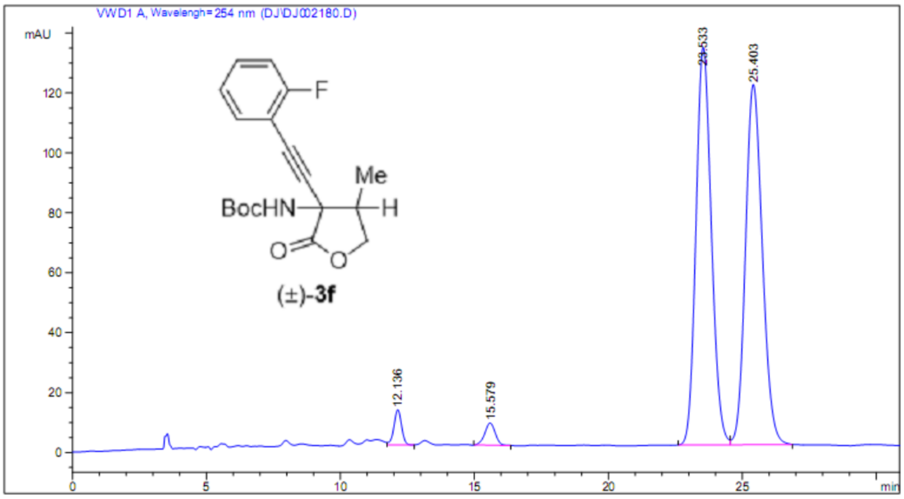

Peak #	RetTime [min]	Type	Width [min]	Area mAU *s	Height [mAU]	Area %
1	12.136	VB	0.3107	242.63432	11.90126	2.2064
2	15.579	BB	0.4230	204.31366	7.42477	1.8579
3	23.533	BV	0.6198	5295.83984	132.73135	48.1579
4	25.403	VB	0.6743	5254.02881	120.25521	47.7777

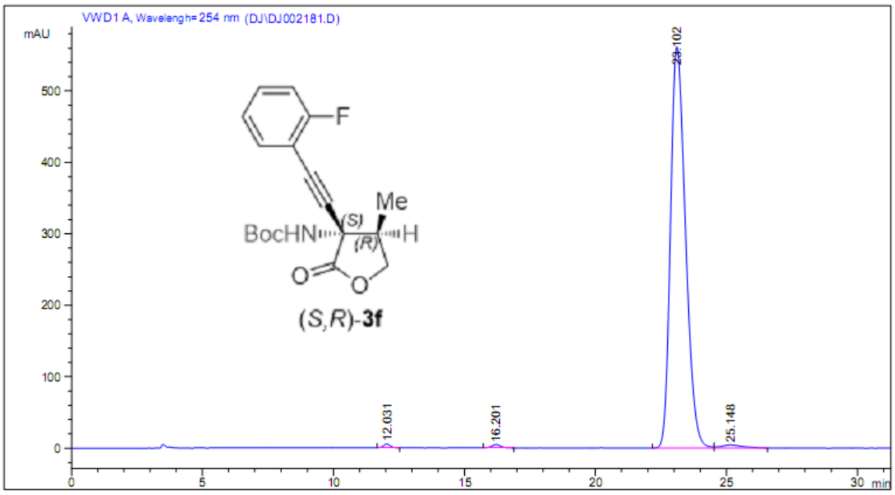

Peak #	RetTime [min]	Type	Width [min]	Area mAU *s	Height [mAU]	Area %
1	12.031	BB	0.2932	99.36092	5.25853	0.4364
2	16.201	BB	0.3808	113.49390	4.67454	0.4984
3	23.102	BB	0.6166	2.23051e4	561.22577	97.9610
4	25.148	BB	0.8109	251.41617	4.38017	1.1042

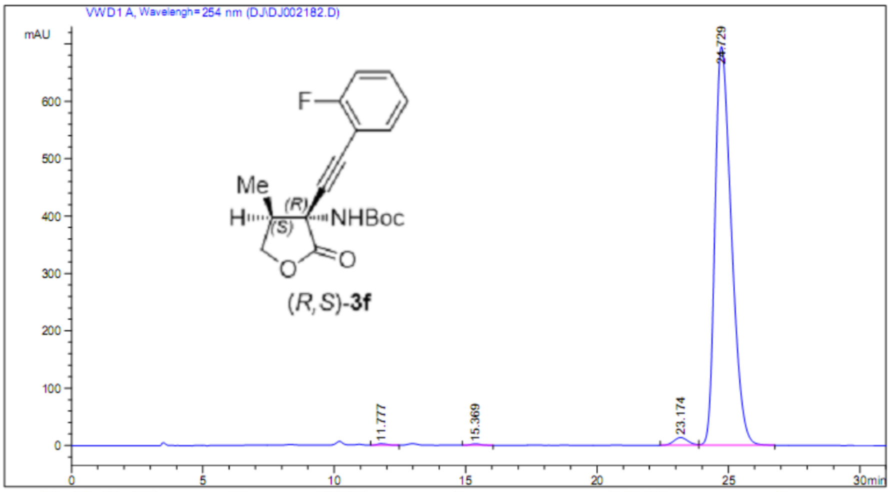

Peak #	RetTime [min]	Type	Width [min]	Area mAU *s	Height [mAU]	Area %
1	11.777	BB	0.3832	66.98087	2.50571	0.2166
2	15.369	BB	0.4031	71.16886	2.64205	0.2301
3	23.174	BV	0.5912	528.19293	13.78318	1.7078
4	24.729	VB	0.6754	3.02611e4	695.06134	97.8455

Supplementary Figure 77. HPLC Spectrum for **3f.**

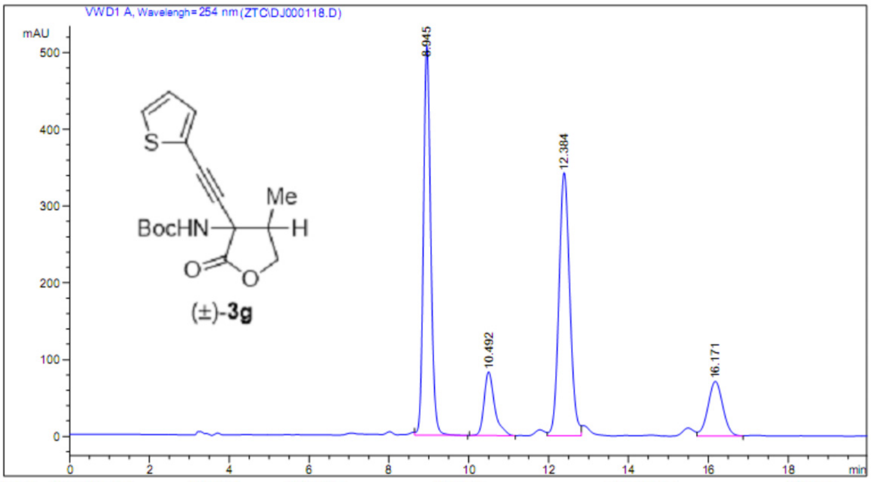

Peak #	RetTime [min]	Type	Width [min]	Area mAU *s	Height [mAU]	Area %
1	8.945	VB	0.1954	6412.20215	507.84042	39.3949
2	10.492	BV	0.2777	1531.63708	83.06625	9.4100
3	12.384	VV	0.2920	6486.03955	342.98270	39.8486
4	16.171	VB	0.4009	1846.83630	71.36015	11.3465

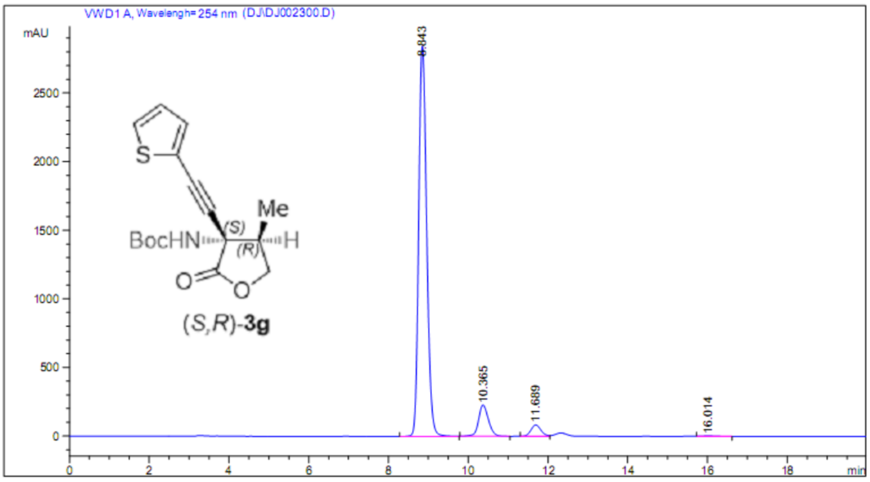

Peak #	RetTime [min]	Type	Width [min]	Area mAU *s	Height [mAU]	Area %
1	8.843	BB	0.2177	3.96283e4	2847.02612	87.5380
2	10.365	BV	0.2685	4024.83740	229.69853	8.8908
3	11.689	VV	0.2771	1513.85510	84.06779	3.3441
4	16.014	VV	0.4244	102.82212	3.65443	0.2271

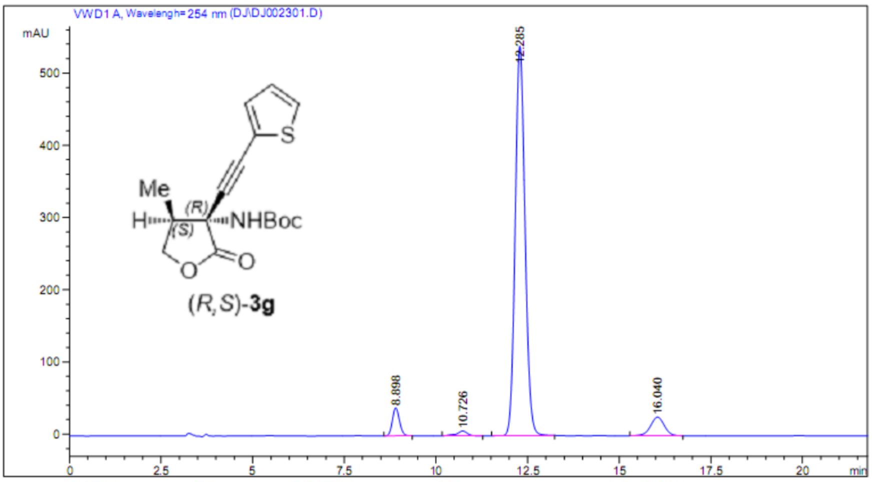

Peak #	RetTime [min]	Type	Width [min]	Area mAU *s	Height [mAU]	Area %
1	8.898	BB	0.2105	526.15491	38.82022	4.6263
2	10.726	BB	0.2949	133.76884	6.71791	1.1762
3	12.285	BB	0.2883	1.00328e4	539.60773	88.2149
4	16.040	BB	0.4060	680.41425	25.85953	5.9826

Supplementary Figure 78. HPLC Spectrum for **3g.**

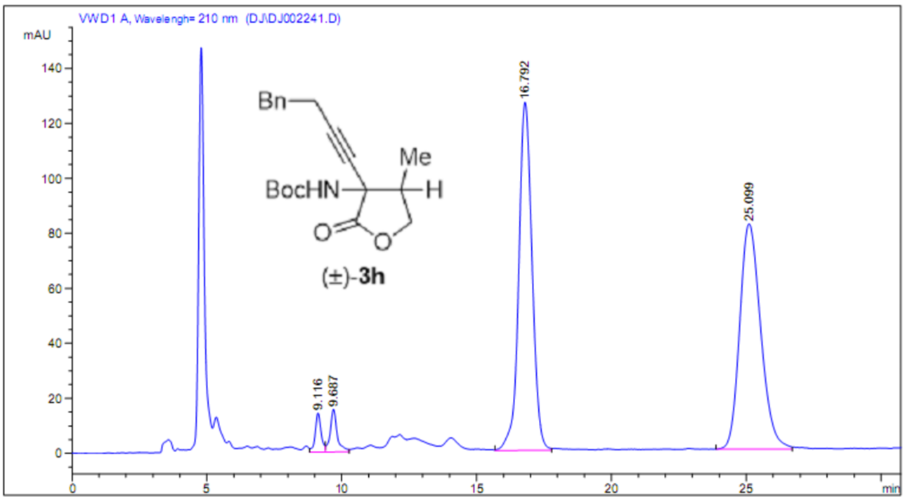

Peak #	RetTime [min]	Type	Width [min]	Area mAU *s	Height [mAU]	Area %
1	9.116	VV	0.2314	216.35081	14.21799	2.3229
2	9.687	VB	0.2688	282.34808	15.53755	3.0314
3	16.792	VB	0.5291	4372.67920	126.75172	46.9474
4	25.099	VV	0.7877	4442.61133	82.02416	47.6983

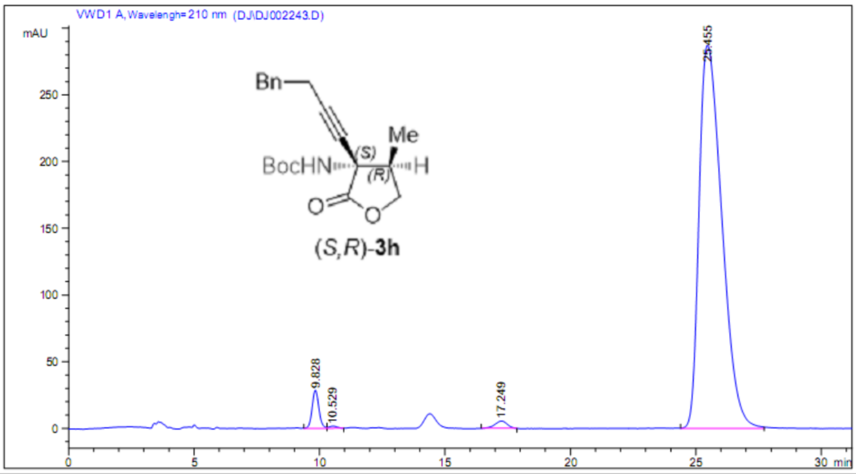

Peak #	RetTime [min]	Type	Width [min]	Area mAU *s	Height [mAU]	Area %
1	9.828	VV	0.2882	541.45129	28.74431	2.8623
2	10.529	VV	0.2816	38.74356	1.70190	0.2048
3	17.249	VV	0.4589	212.28015	5.76182	1.1222
4	25.455	VV	0.9596	1.81243e4	287.53931	95.8107

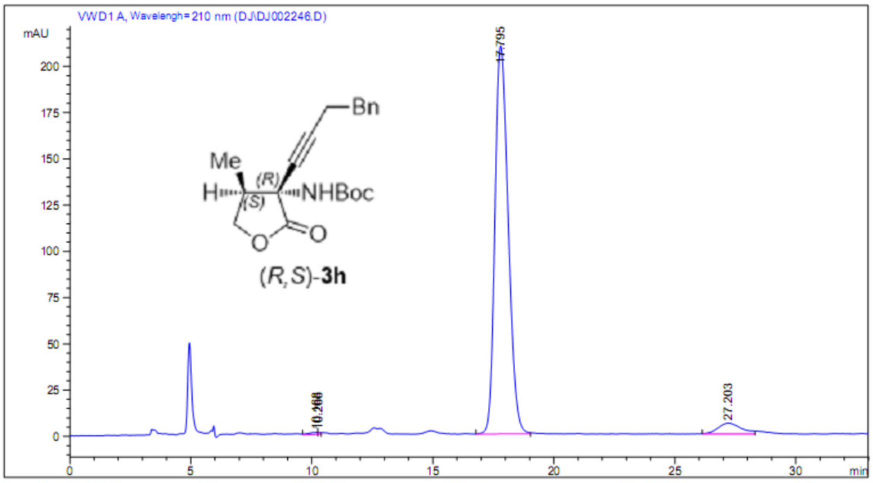

Peak #	RetTime [min]	Type	Width [min]	Area mAU *s	Height [mAU]	Area %
1	10.168	BV	0.3275	31.74338	1.16185	0.3754
2	10.266	VV	0.0937	8.06102	1.08777	0.0953
3	17.795	BB	0.5966	8007.27148	209.86009	94.7025
4	27.203	VV	0.8016	408.11227	6.01616	4.8268

Supplementary Figure 79. HPLC Spectrum for **3h.**

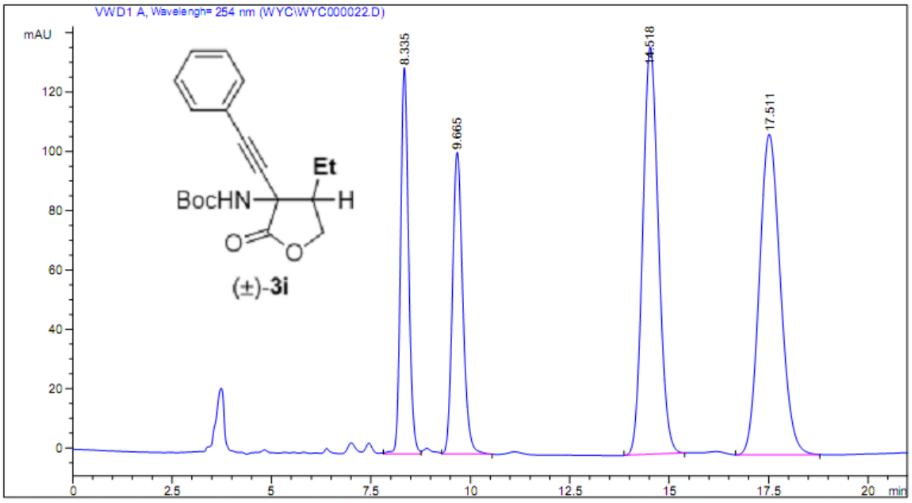

Peak #	RetTime [min]	Type	Width [min]	Area mAU *s	Height [mAU]	Area %
1	8.335	BV	0.2161	1829.32922	130.42084	16.0232
2	9.665	VB	0.2761	1838.14026	101.85986	16.1004
3	14.518	BB	0.4279	3768.88892	137.33658	33.0119
4	17.511	VB	0.5710	3980.39600	108.05679	34.8645

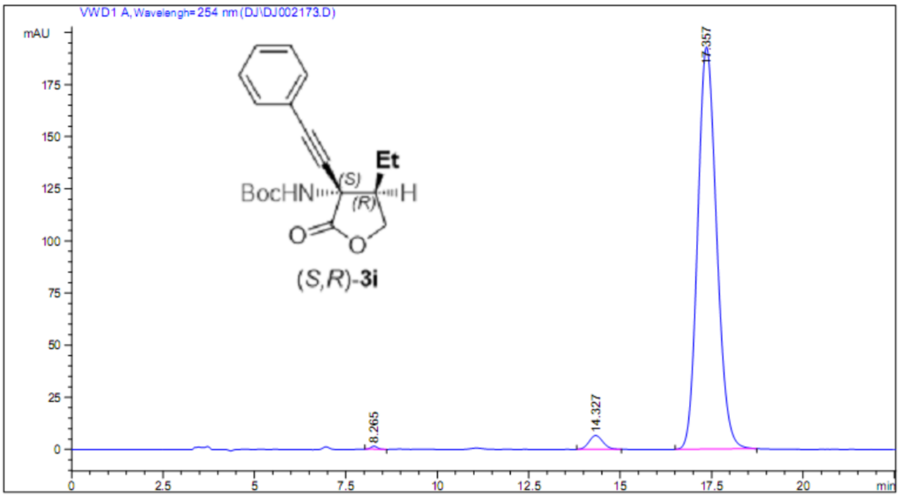

Peak #	RetTime [min]	Type	Width [min]	Area mAU *s	Height [mAU]	Area %
1	8.265	BB	0.2019	19.34431	1.51022	0.2711
2	14.327	BB	0.4118	176.87485	6.66017	2.4792
3	17.357	BB	0.5608	6938.21045	192.91983	97.2497

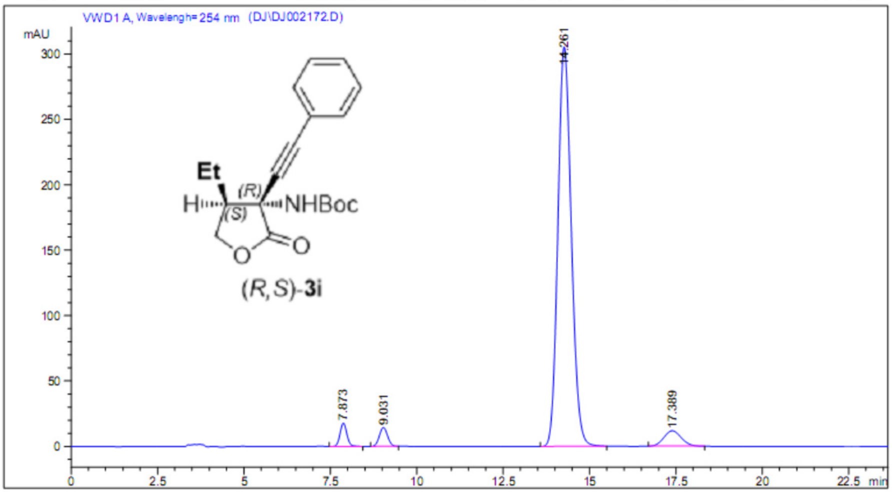

Peak #	RetTime [min]	Type	Width [min]	Area mAU *s	Height [mAU]	Area %
1	7.873	VB	0.2174	250.93414	17.90339	2.7393
2	9.031	BB	0.2623	242.55217	14.48963	2.6478
3	14.261	BB	0.4181	8243.76367	305.56104	89.9923
4	17.389	BB	0.5428	423.26688	11.90739	4.6206

Supplementary Figure 80. HPLC Spectrum for **3i.**

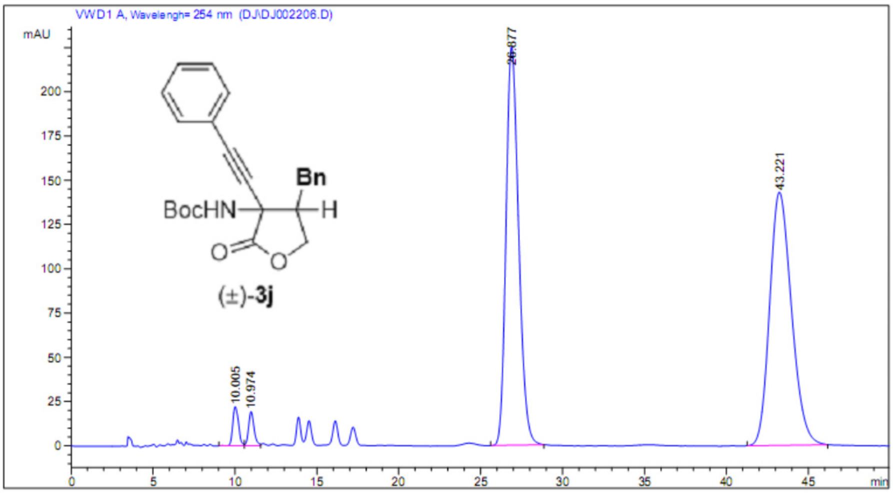

Peak #	RetTime [min]	Type	Width [min]	Area mAU *s	Height [mAU]	Area %
1	10.005	BV	0.4072	549.27277	22.15561	2.0899
2	10.974	VV	0.3606	457.90802	19.34361	1.7423
3	26.877	VB	0.8303	1.20281e4	225.05609	45.7651
4	43.221	BB	1.4514	1.32470e4	143.07997	50.4028

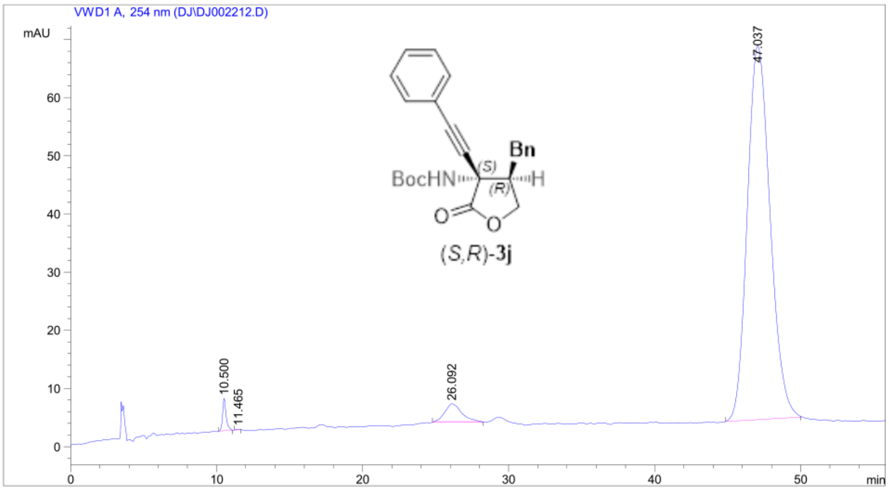

Peak #	RetTime [min]	Type	Width [min]	Area mAU *s	Height [mAU]	Area %
1	10.500	BB	0.2466	94.92022	5.57618	1.2885
2	11.465	MM	0.3154	2.70680	1.43046e-1	0.0367
3	26.092	BB	1.0273	270.42960	3.20753	3.6709
4	47.037	BB	1.6276	6998.84912	64.32231	95.0039

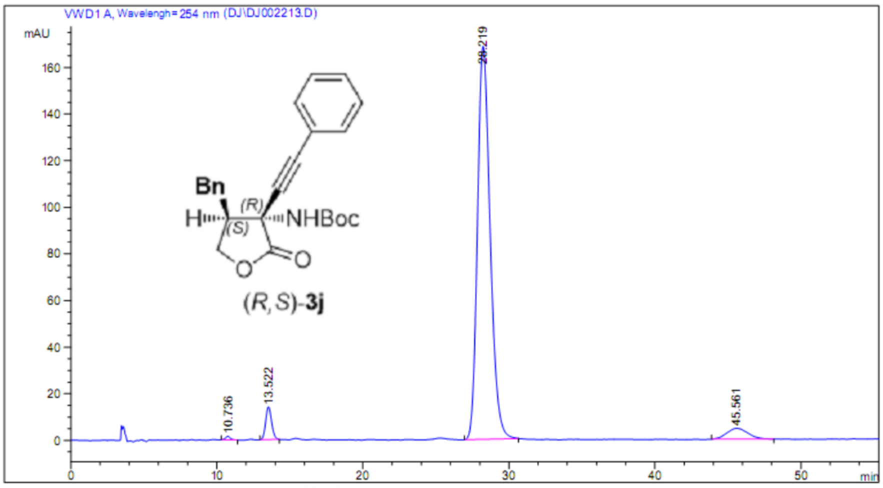

Peak #	RetTime [min]	Type	Width [min]	Area mAU *s	Height [mAU]	Area %
1	10.736	BB	0.3518	37.19602	1.55563	0.3360
2	13.522	BB	0.4448	402.40704	14.11038	3.6354
3	28.219	BB	0.9328	1.01579e4	168.63661	91.7667
4	45.561	BB	1.2035	471.75934	4.60663	4.2619

Supplementary Figure 81. HPLC Spectrum for **3j.**

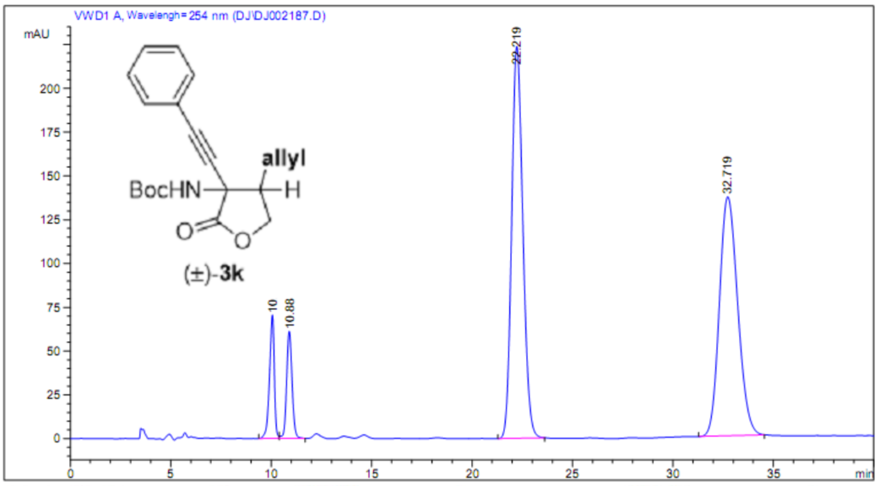

Peak #	RetTime [min]	Type	Width [min]	Area mAU *s	Height [mAU]	Area %
1	10.053	BV	0.2501	1178.23279	70.55910	5.9943
2	10.889	VB	0.2919	1158.12244	61.25323	5.8920
3	22.219	BB	0.6001	8685.91309	223.68654	44.1902
4	32.719	BB	0.9795	8633.45801	136.51692	43.9234

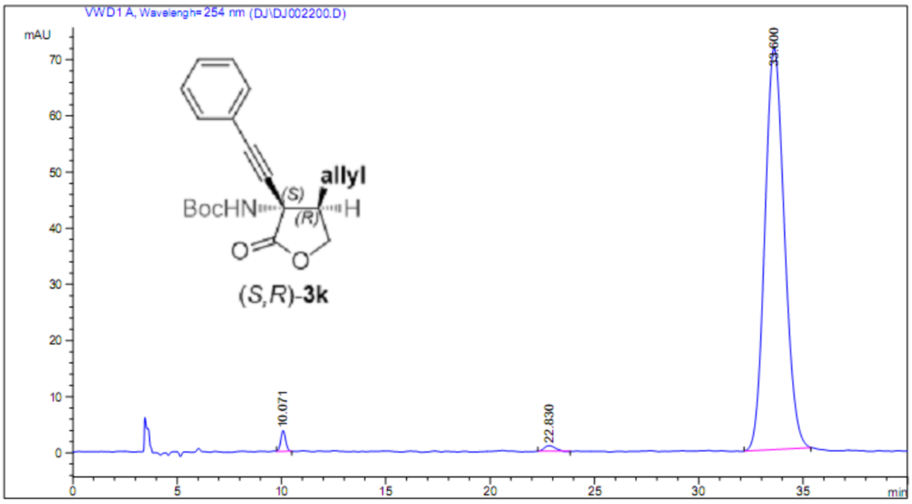

Peak #	RetTime [min]	Type	Width [min]	Area mAU *s	Height [mAU]	Area %
1	10.071	BB	0.2522	60.81165	3.71362	1.2776
2	22.830	BB	0.4700	36.82460	9.87405e-1	0.7737
3	33.600	BB	1.0007	4662.15088	71.52881	97.9487

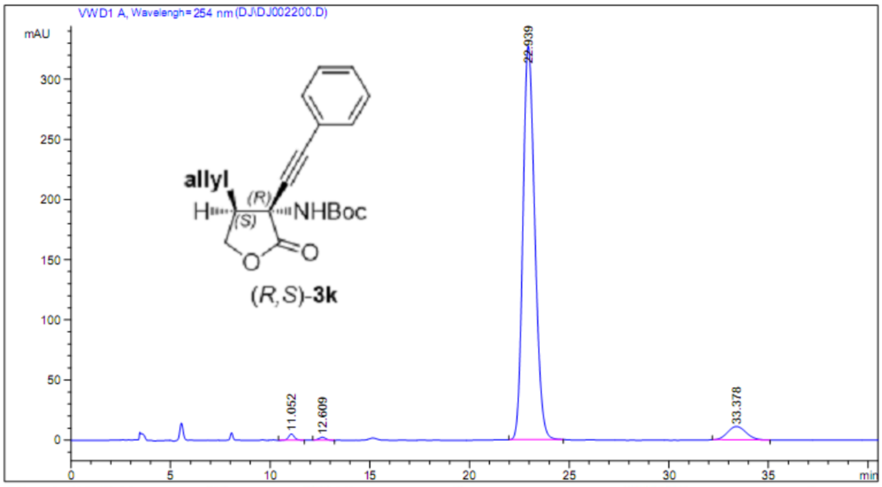

Peak #	RetTime [min]	Type	Width [min]	Area mAU *s	Height [mAU]	Area %
1	11.052	VB	0.3178	106.48357	5.16363	0.7372
2	12.609	BB	0.3614	56.51800	2.39303	0.3913
3	22.939	BB	0.6448	1.35559e4	327.32407	93.8457
4	33.378	BB	0.9796	725.97296	11.19158	5.0258

Supplementary Figure 82. HPLC Spectrum for **3k.**

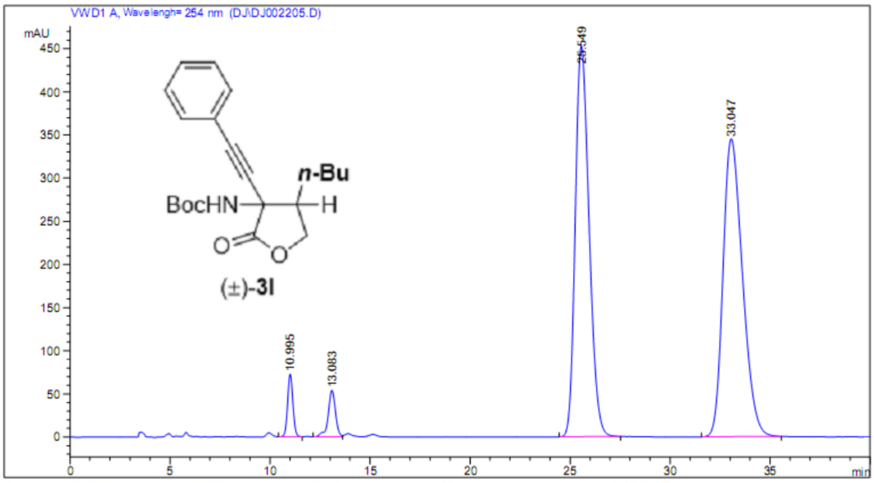

Peak #	RetTime [min]	Type	Width [min]	Area mAU *s	Height [mAU]	Area %
1	10.995	VB	0.2863	1352.03979	72.88917	2.8249
2	13.083	VV	0.3878	1387.01733	54.12148	2.8980
3	25.549	BB	0.7502	2.17724e4	452.84885	45.4901
4	33.047	BB	1.0452	2.33504e4	345.31952	48.7871

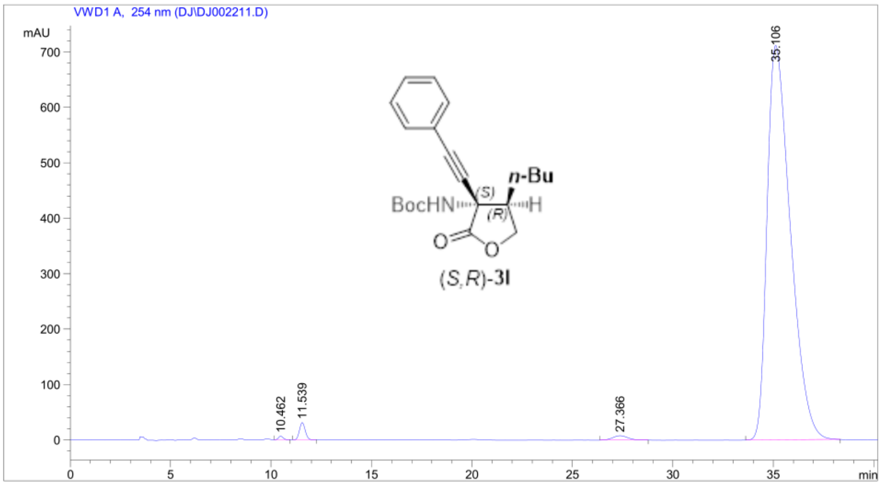

Peak #	RetTime [min]	Type	Width [min]	Area mAU *s	Height [mAU]	Area %
1	10.462	VB	0.2604	120.55244	6.95668	0.2056
2	11.539	BB	0.3172	641.70618	31.37469	1.0942
3	27.366	BB	0.7806	434.25555	8.08960	0.7405
4	35.106	BB	1.2281	5.74489e4	712.06219	97.9597

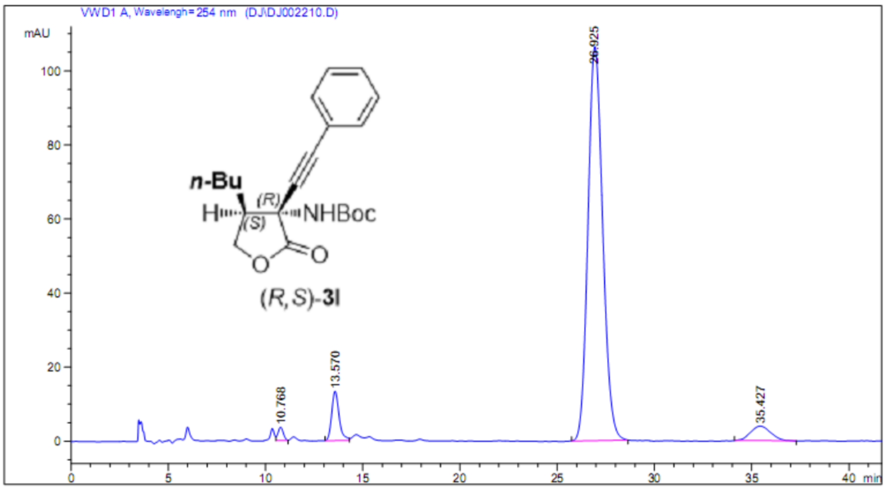

Peak #	RetTime [min]	Type	Width [min]	Area mAU *s	Height [mAU]	Area %
1	10.768	VV	0.3000	73.29544	3.71540	1.1587
2	13.570	BB	0.4080	357.20721	13.35941	5.6468
3	26.925	BB	0.8210	5603.09570	106.42097	88.5755
4	35.427	BB	0.8989	292.18536	3.97248	4.6190

Supplementary Figure 83. HPLC Spectrum for **3l.**

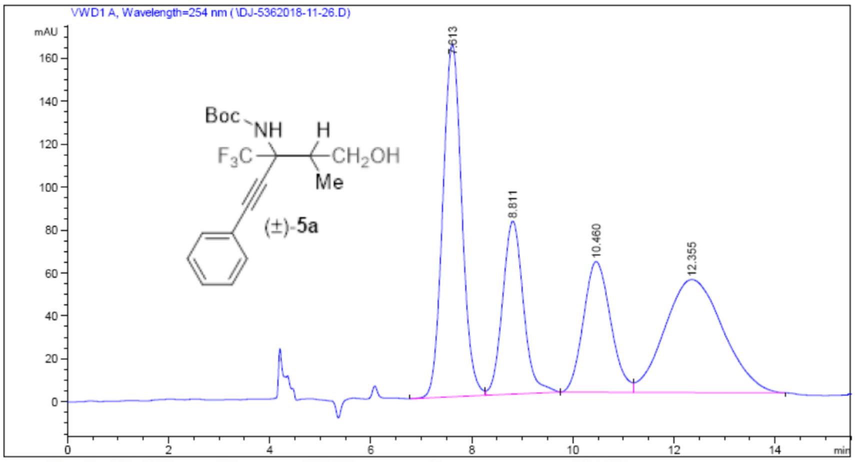

Peak #	RetTime [min]	Type	Width [min]	Area [mAU*s]	Height [mAU]	Area %
1	7.613	BV	0.4078	4302.16699	164.10844	32.8084
2	8.811	VB	0.4450	2308.05273	80.62219	17.6012
3	10.460	BV	0.5860	2281.29053	60.90016	17.3972
4	12.355	VB	1.2316	4221.50244	52.64309	32.1932

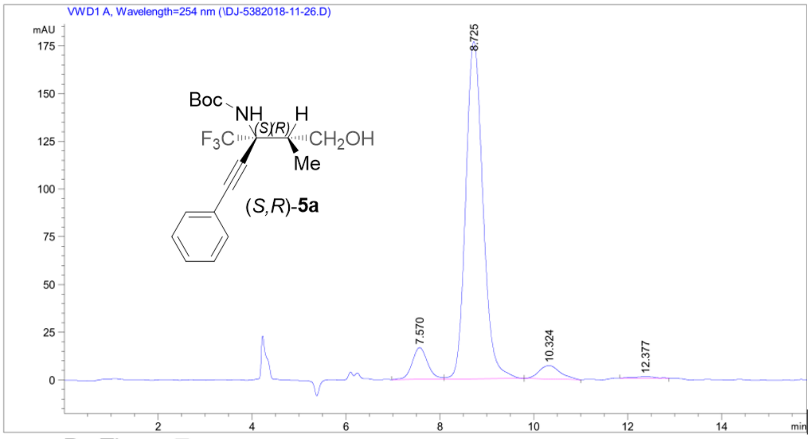

Peak #	RetTime [min]	Type	Width [min]	Area [mAU*s]	Height [mAU]	Area %
1	7.570	BV	0.3459	377.01083	16.60513	7.3643
2	8.725	VB	0.3941	4498.70117	176.64825	87.8744
3	10.324	BB	0.4822	217.99008	7.00292	4.2581
4	12.377	MM	0.5270	25.76276	8.14827e-1	0.5032

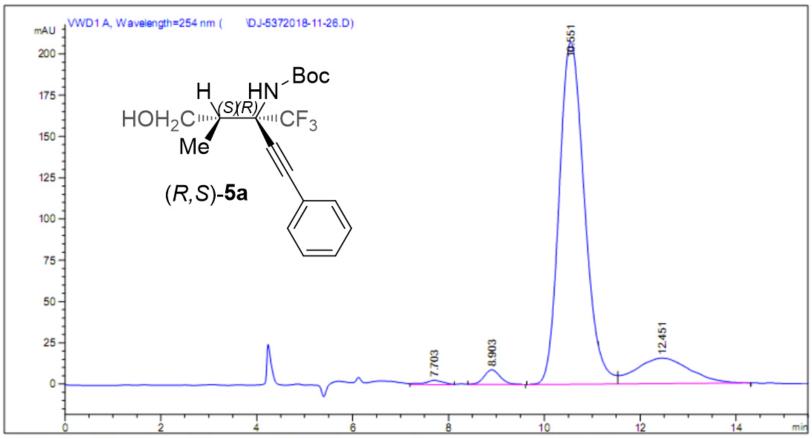

Peak #	RetTime [min]	Type	Width [min]	Area [mAU*s]	Height [mAU]	Area %
1	7.703	VB	0.4971	136.81914	3.66024	1.4983
2	8.903	BB	0.3606	204.51309	8.78453	2.2396
3	10.551	BV R	0.5689	7584.20947	207.26303	83.0522
4	12.451	VB E	1.0809	1206.31946	15.43320	13.2100

Supplementary Figure 84. HPLC Spectrum for **5a.**

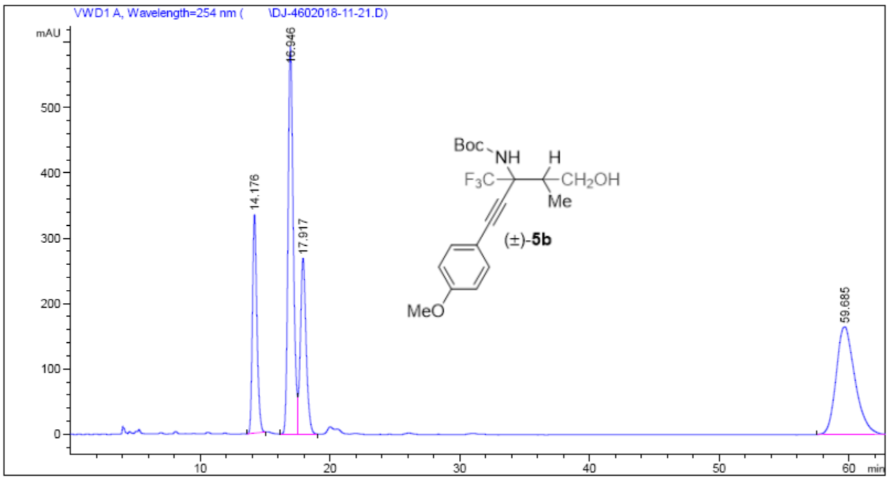

Peak #	RetTime [min]	Type	Width [min]	Area [mAU*s]	Height [mAU]	Area %
1	14.176	BB	0.3685	8069.90527	335.64496	16.2515
2	16.946	BV	0.4361	1.68022e4	593.87543	33.8370
3	17.917	VB	0.4624	8309.13086	269.69235	16.7333
4	59.685	BBA	1.4120	1.64751e4	164.56612	33.1782

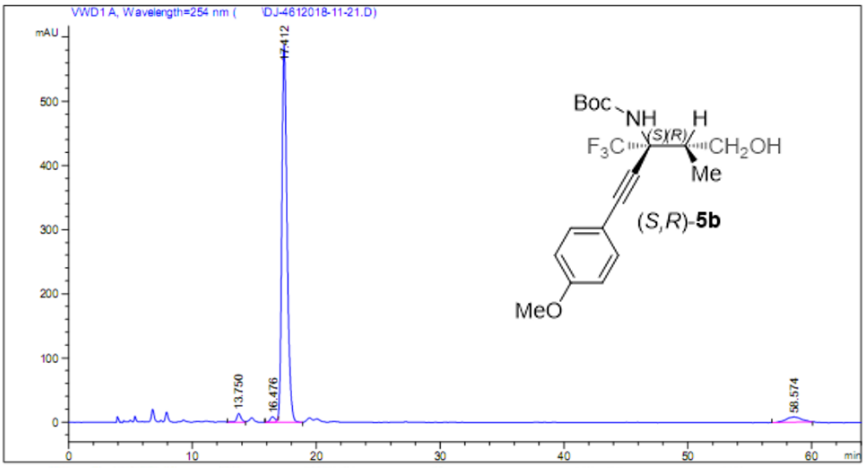

Peak #	RetTime [min]	Type	Width [min]	Area [mAU*s]	Height [mAU]	Area %
1	13.750	BV	0.3689	332.39188	13.61090	1.7896
2	16.476	BV E	0.4125	229.84480	8.55414	1.2375
3	17.412	VB R	0.4407	1.72904e4	588.59174	93.0913
4	58.574	BB	1.0868	720.95276	8.24781	3.8816

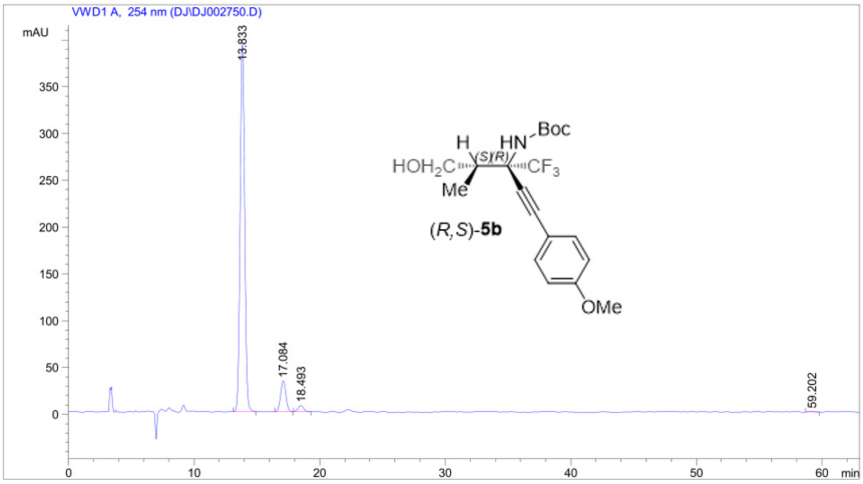

Peak #	RetTime [min]	Type	Width [min]	Area mAU *s	Height [mAU]	Area %
1	13.833	VB	0.3888	9832.47461	391.87131	89.1079
2	17.084	BB	0.4557	967.77563	33.00037	8.7706
3	18.493	BB	0.4892	214.06932	6.72706	1.9400
4	59.202	MM	0.6878	20.03110	4.85385e-1	0.1815

Supplementary Figure 85. HPLC Spectrum for **5b.**

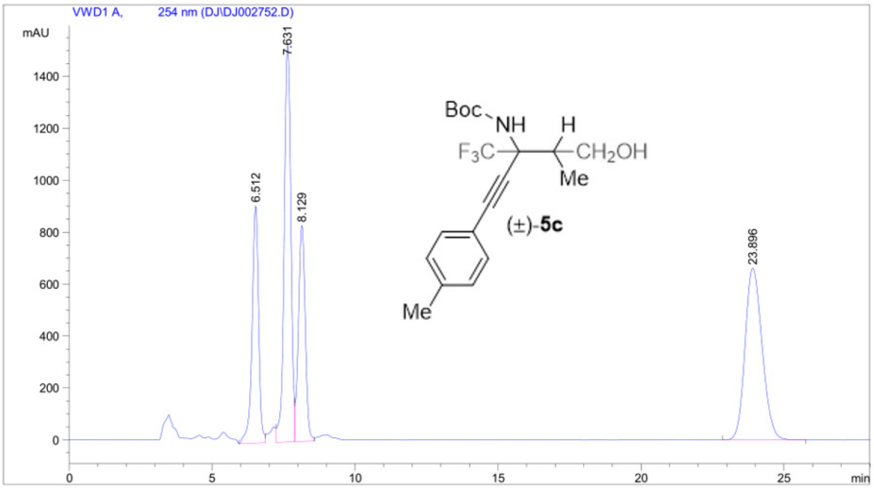

Peak #	RetTime [min]	Type	Width [min]	Area mAU *s	Height [mAU]	Area %
1	6.512	VV	0.2495	1.47592e4	914.44818	18.1779
2	7.631	VV	0.2594	2.51943e4	1527.82544	31.0302
3	8.129	VV	0.2562	1.37155e4	832.73584	16.8925
4	23.896	BB	0.6441	2.75239e4	661.58905	33.8994

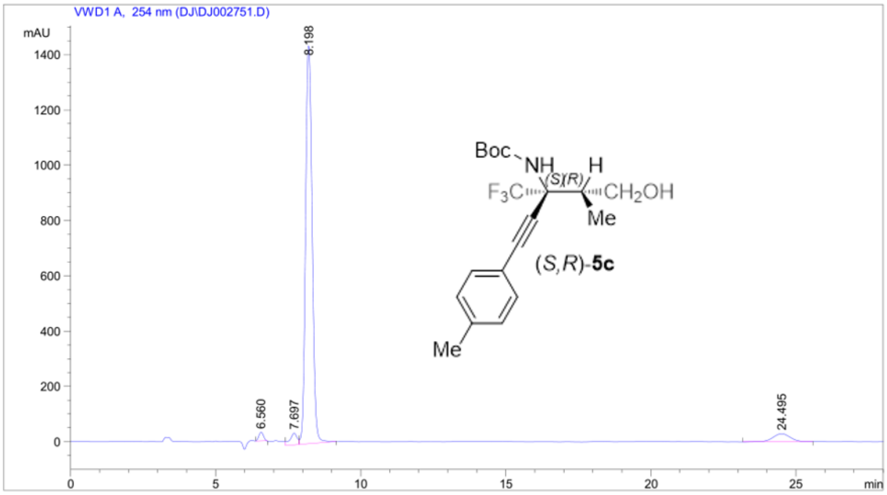

Peak #	RetTime [min]	Type	Width [min]	Area mAU *s	Height [mAU]	Area %
1	6.560	MM	0.1912	371.96246	32.41880	1.4791
2	7.697	VV	0.2536	752.09216	42.99062	2.9908
3	8.198	VB	0.2514	2.27542e4	1439.64063	90.4839
4	24.495	VV	0.6611	1268.99561	29.55585	5.0463

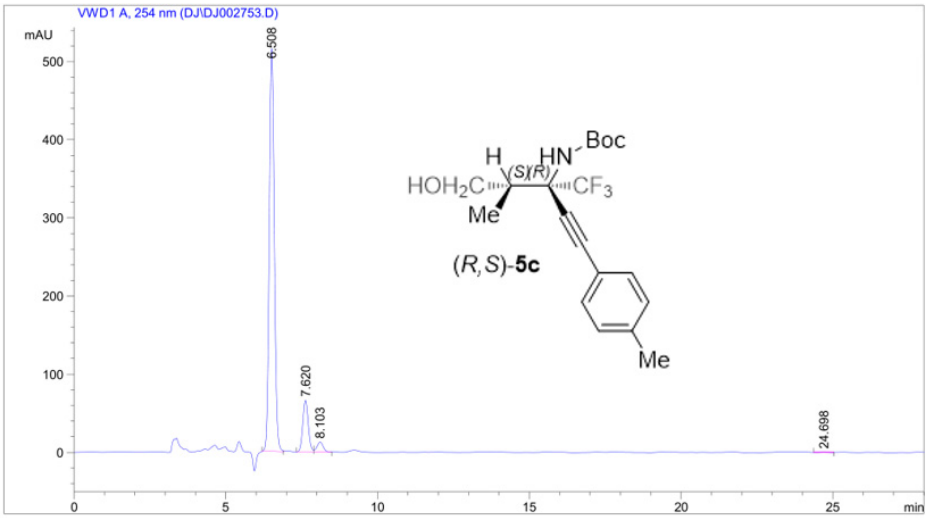

Peak #	RetTime [min]	Type	Width [min]	Area mAU *s	Height [mAU]	Area %
1	6.508	MM	0.1912	5914.43408	515.46942	85.2912
2	7.620	BV	0.1941	825.17615	65.92165	11.8997
3	8.103	VB	0.2201	180.85556	12.69311	2.6081
4	24.698	MM	0.4672	13.93692	4.97154e-1	0.2010

Supplementary Figure 86. HPLC Spectrum for **5c.**

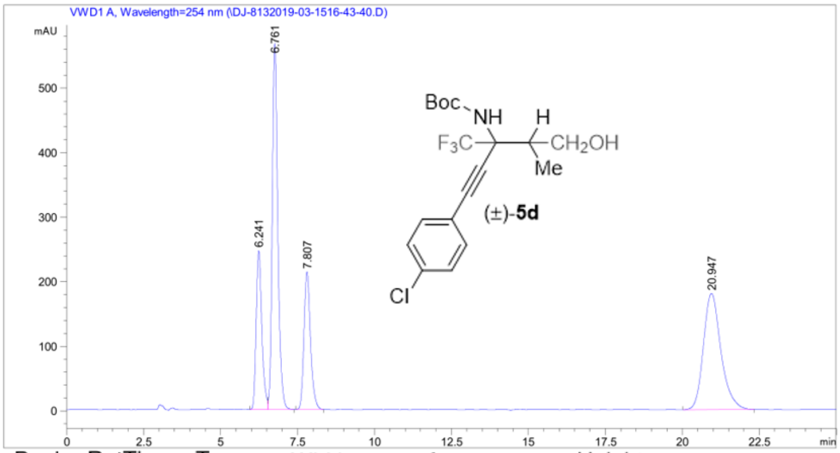

Peak #	RetTime [min]	Type	Width [min]	Area [mAU*s]	Height [mAU]	Area %
1	6.241	BV	0.1973	3148.06177	245.92422	15.3538
2	6.761	VB	0.1901	7104.27393	567.18311	34.6490
3	7.807	BB	0.2244	3126.64697	212.81296	15.2493
4	20.947	BB	0.6039	7124.55322	179.96817	34.7479

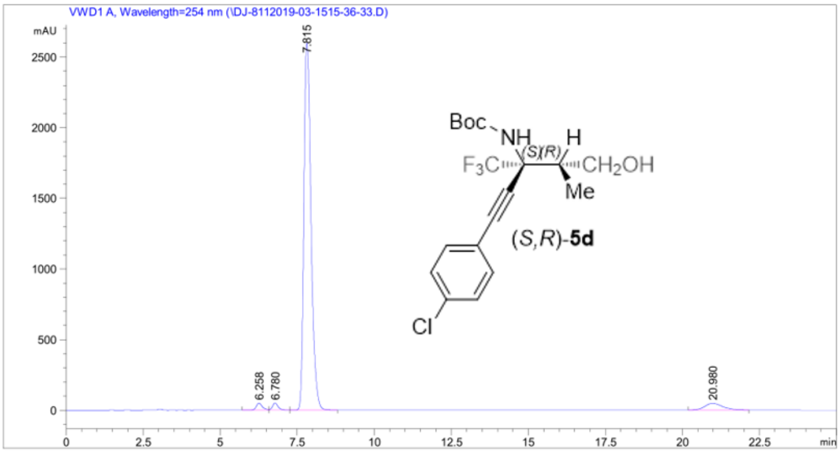

Peak #	RetTime [min]	Type	Width [min]	Area [mAU*s]	Height [mAU]	Area %
1	6.258	BV	0.2108	684.48267	49.04763	1.5203
2	6.780	VB	0.1879	625.41882	50.37276	1.3891
3	7.815	BV	0.2488	4.18237e4	2597.72314	92.8949
4	20.980	BB	0.6137	1889.00293	47.31835	4.1957

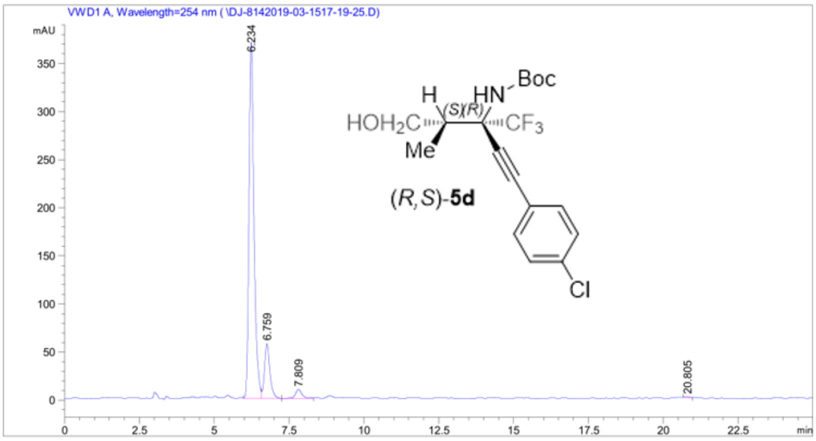

Peak #	RetTime [min]	Type	Width [min]	Area [mAU*s]	Height [mAU]	Area %
1	6.234	VV	0.1757	4303.20215	370.01218	83.3571
2	6.759	VB	0.1881	711.78967	56.84711	13.7880
3	7.809	BV	0.2284	143.21040	9.31429	2.7741
4	20.805	MM	0.1731	4.16583	4.01124e-1	0.0807

Supplementary Figure 87. HPLC Spectrum for **5d.**

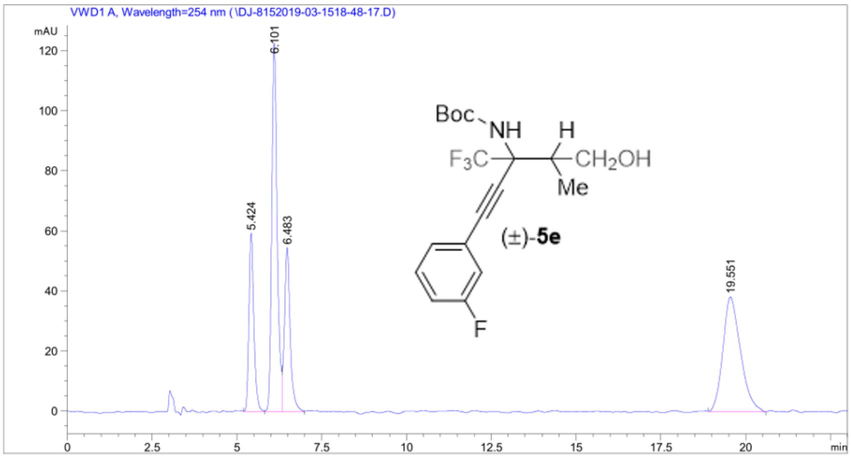

Peak #	RetTime [min]	Type	Width [min]	Area [mAU*s]	Height [mAU]	Area %
1	5.424	VV	0.1556	604.11884	59.44739	15.1013
2	6.101	VV	0.1702	1368.19202	122.58217	34.2010
3	6.483	VV	0.1753	644.00562	54.72282	16.0983
4	19.551	VB	0.5365	1384.13062	38.21633	34.5994

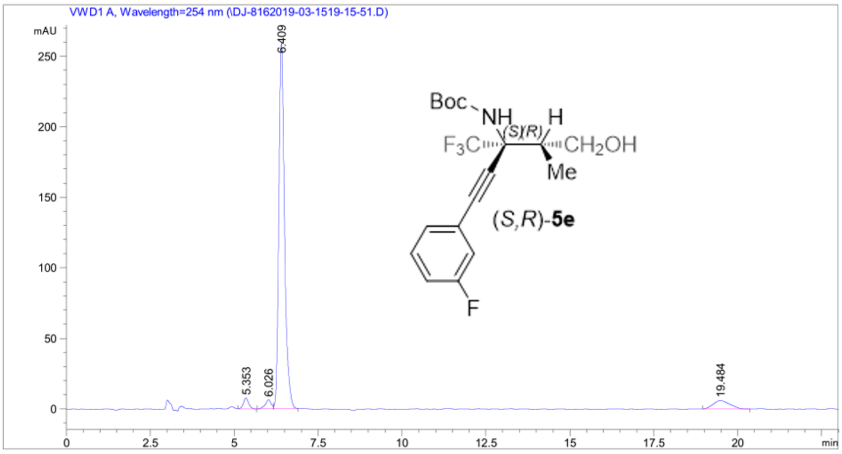

Peak #	RetTime [min]	Type	Width [min]	Area [mAU*s]	Height [mAU]	Area %
1	5.353	VB	0.1683	85.30163	7.69962	2.5029
2	6.026	BV	0.1811	77.56787	6.36707	2.2760
3	6.409	VB	0.1787	3036.09961	259.12433	89.0855
4	19.484	BB	0.4984	209.10342	5.95849	6.1355

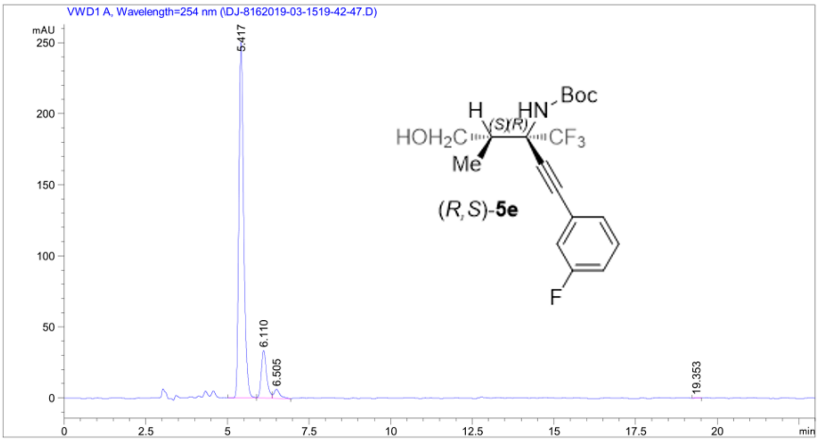

Peak #	RetTime [min]	Type	Width [min]	Area [mAU*s]	Height [mAU]	Area %
1	5.417	BV	0.1584	2585.35889	250.50546	84.7979
2	6.110	VV	0.1725	381.84131	33.62527	12.5241
3	6.505	VB	0.1849	80.29866	6.37657	2.6337
4	19.353	MM	0.1810	1.34886	1.24202e-1	0.0442

Supplementary Figure 88. HPLC Spectrum for **5e.**

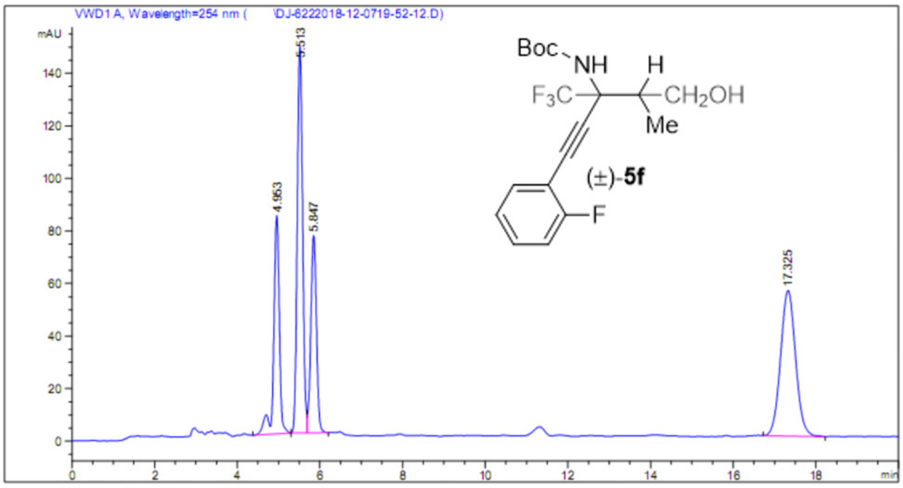

Peak #	RetTime [min]	Type	Width [min]	Area [mAU*s]	Height [mAU]	Area %
1	4.953	VB R	0.1357	790.76935	83.13728	18.7071
2	5.513	BV	0.1435	1329.48755	147.01859	31.4515
3	5.847	VB	0.1480	709.10809	75.18780	16.7752
4	17.325	BB	0.3934	1397.74524	55.39039	33.0662

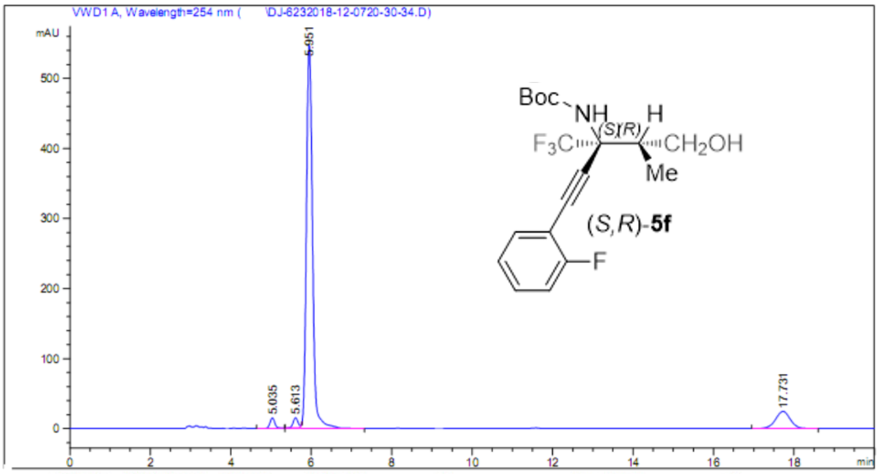

Peak #	RetTime [min]	Type	Width [min]	Area [mAU*s]	Height [mAU]	Area %
1	5.035	BB	0.1281	122.54256	14.88449	1.8440
2	5.613	BV E	0.1346	128.01193	14.84979	1.9263
3	5.951	VB R	0.1651	5767.06445	546.77655	86.7807
4	17.731	BB	0.3896	627.93951	24.69759	9.4490

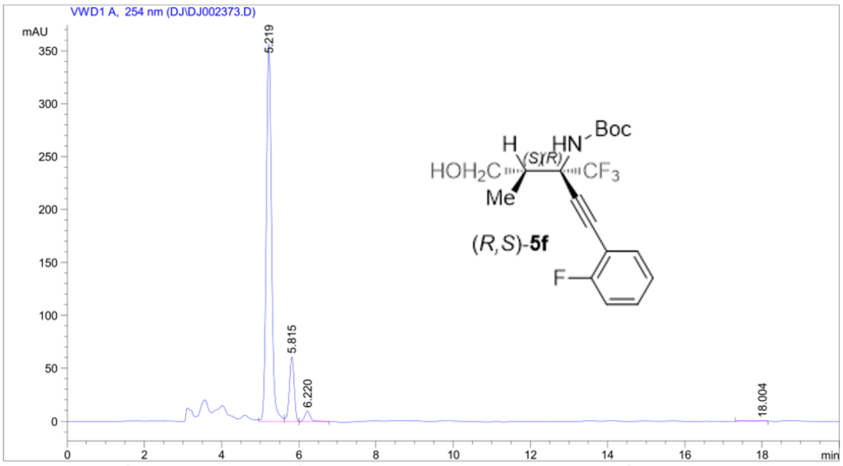

Peak #	RetTime [min]	Type	Width [min]	Area mAU *s	Height [mAU]	Area %
1	5.219	VV	0.1460	3355.24316	357.15723	83.6948
2	5.815	VV	0.1347	531.78168	61.27617	13.2650
3	6.220	VB	0.1635	110.93985	10.06812	2.7673
4	18.004	MM	0.7059	10.93730	2.58228e-1	0.2728

Supplementary Figure 89. HPLC Spectrum for **5f.**

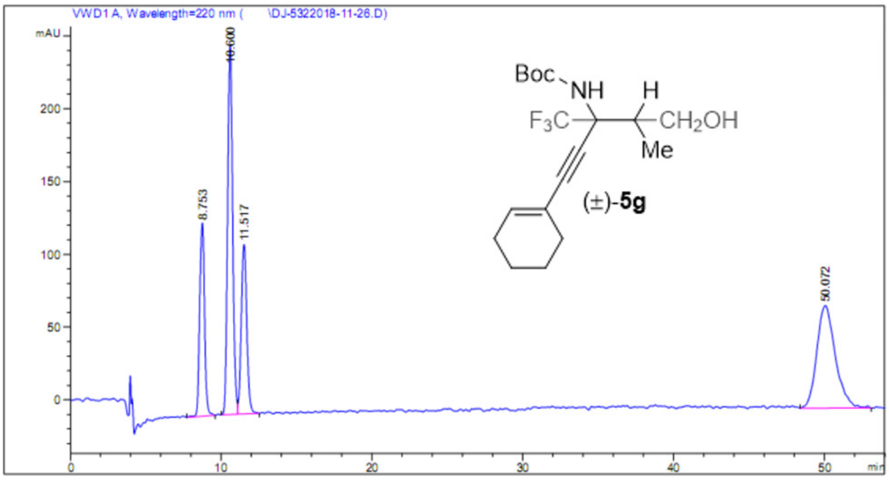

Peak #	RetTime [min]	Type	Width [min]	Area [mAU*s]	Height [mAU]	Area %
1	8.753	VB R	0.3586	2978.28638	132.80447	16.7067
2	10.600	BV	0.3583	5867.71289	253.23584	32.9150
3	11.517	VB	0.4029	2974.44922	116.47874	16.6852
4	50.072	BV R	1.1728	6006.41846	70.36866	33.6931

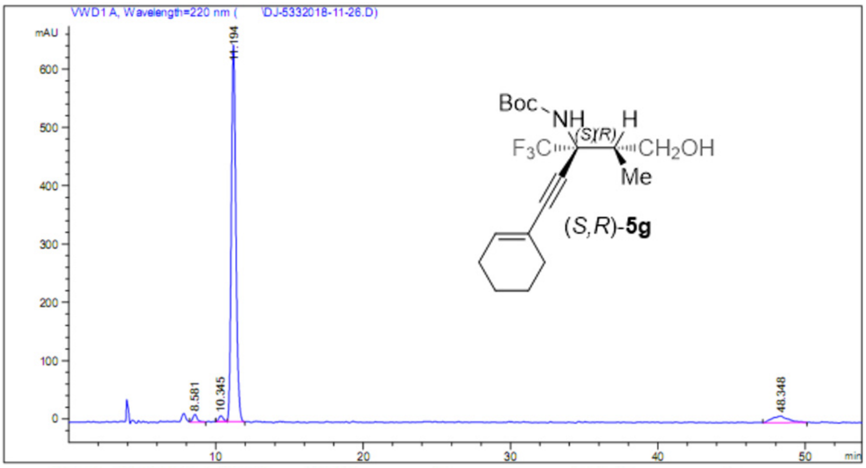

Peak #	RetTime [min]	Type	Width [min]	Area [mAU*s]	Height [mAU]	Area %
1	8.581	VB	0.3101	259.94427	12.95511	1.7040
2	10.345	BV E	0.3339	222.11151	10.36983	1.4560
3	11.194	VB R	0.3301	1.39079e4	646.07910	91.1692
4	48.348	BV R	0.9637	865.09052	11.30548	5.6709

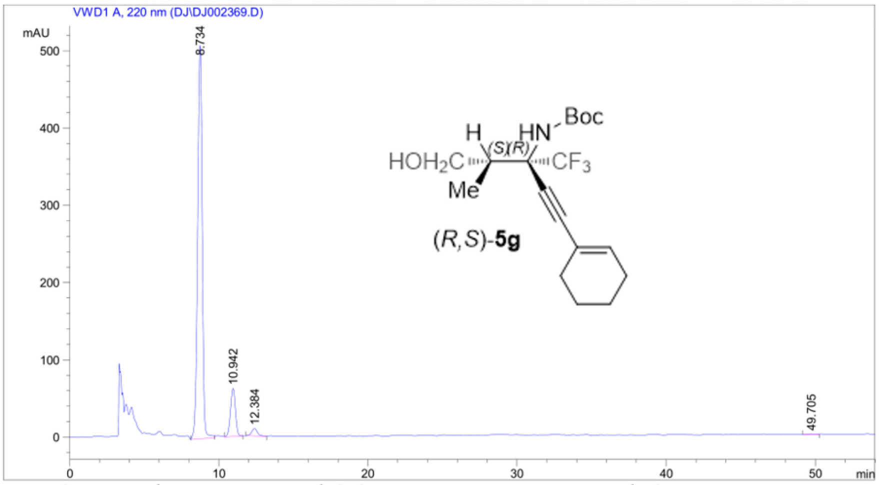

Peak #	RetTime [min]	Type	Width [min]	Area mAU *s	Height [mAU]	Area %
1	8.734	VB	0.3360	1.09195e4	509.41885	86.2737
2	10.942	VB	0.3631	1441.36682	62.30598	11.3881
3	12.384	BB	0.4154	272.30130	9.99492	2.1514
4	49.705	MM	0.4904	23.64847	8.03704e-1	0.1868

Supplementary Figure 90. HPLC Spectrum for **5g.**

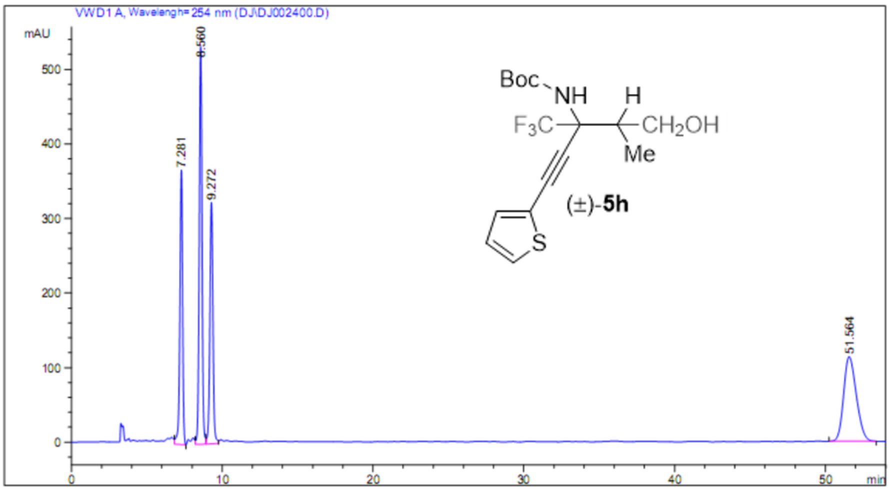

Peak #	RetTime [min]	Type	Width [min]	Area mAU *s	Height [mAU]	Area %
1	7.281	VV	0.1910	4610.02197	368.66132	20.3694
2	8.560	VV	0.1961	6767.27100	533.43323	29.9013
3	9.272	VV	0.2176	4590.23633	324.20523	20.2820
4	51.564	BB	0.9053	6664.51172	113.67397	29.4472

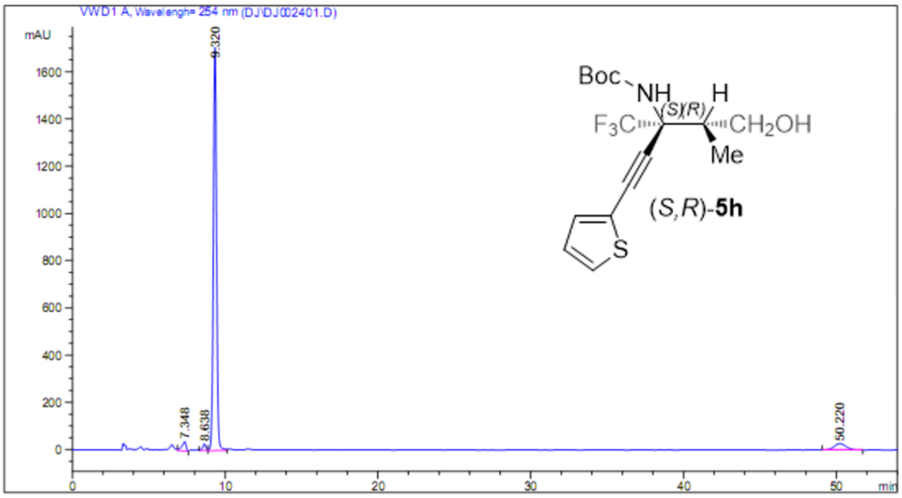

Peak #	RetTime [min]	Type	Width [min]	Area mAU *s	Height [mAU]	Area %
1	7.348	VV	0.2760	789.83466	39.90671	2.8549
2	8.638	VV	0.2299	466.20798	29.90801	1.6851
3	9.320	VV	0.2256	2.49474e4	1709.08765	90.1732
4	50.220	BB	0.8661	1462.64270	26.58619	5.2868

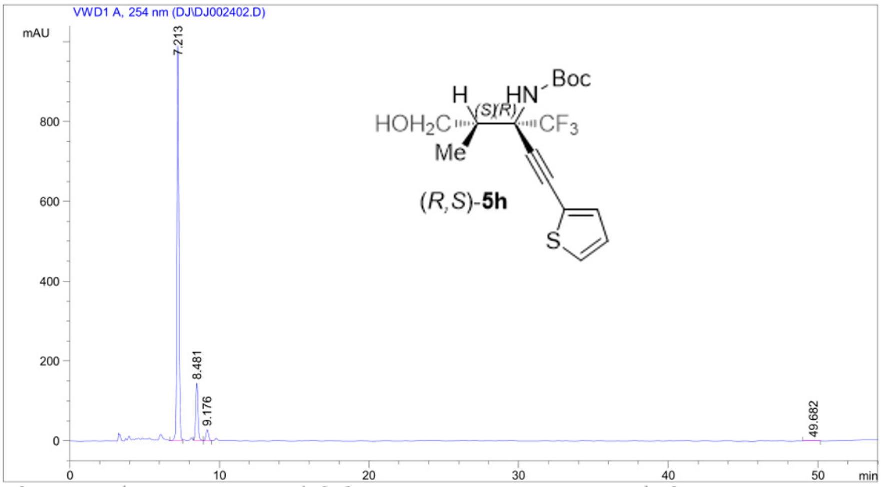

Peak #	RetTime [min]	Type	Width [min]	Area mAU *s	Height [mAU]	Area %
1	7.213	VV	0.1427	9157.49316	991.26044	82.5349
2	8.481	VV	0.1612	1538.88525	145.57460	13.8697
3	9.176	VV	0.1941	363.23688	28.72124	3.2738
4	49.682	MM	0.8287	35.68189	7.17602e-1	0.3216

Supplementary Figure 91. HPLC Spectrum for **5h.**

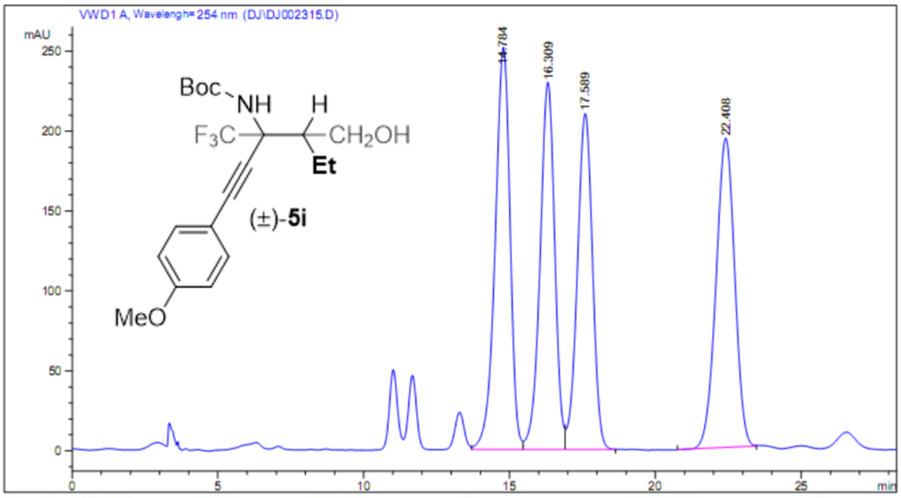

Peak #	RetTime [min]	Type	Width [min]	Area mAU *s	Height [mAU]	Area %
1	14.784	VV	0.5495	8937.45703	251.81972	26.9287
2	16.309	VV	0.5403	8033.20361	229.83755	24.2041
3	17.589	VB	0.5616	7572.36719	210.17014	22.8156
4	22.408	BV	0.6912	8646.36328	193.66606	26.0516

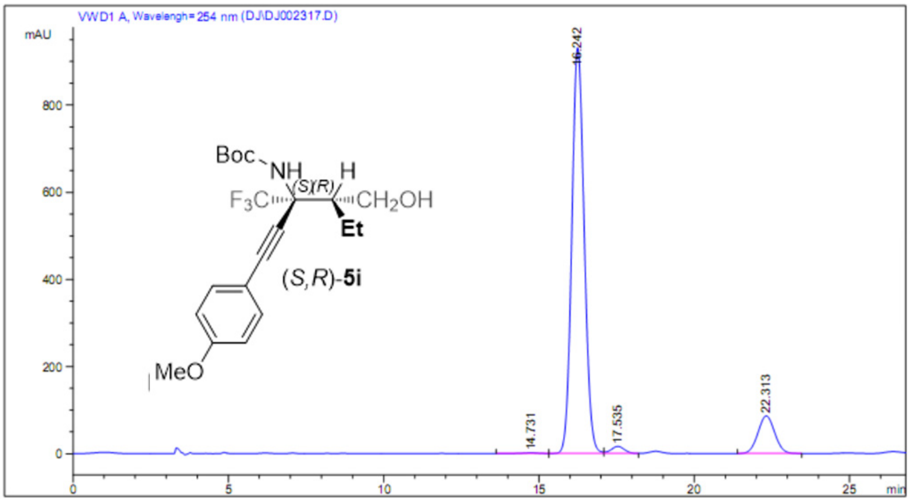

Peak #	RetTime [min]	Type	Width [min]	Area mAU *s	Height [mAU]	Area %
1	14.731	BV	0.5508	77.71417	1.97500	0.2617
2	16.242	VV	0.4314	2.58453e4	931.70001	87.0348
3	17.535	VV	0.4693	510.68549	16.61210	1.7198
4	22.313	BB	0.5868	3261.65088	86.52737	10.9837

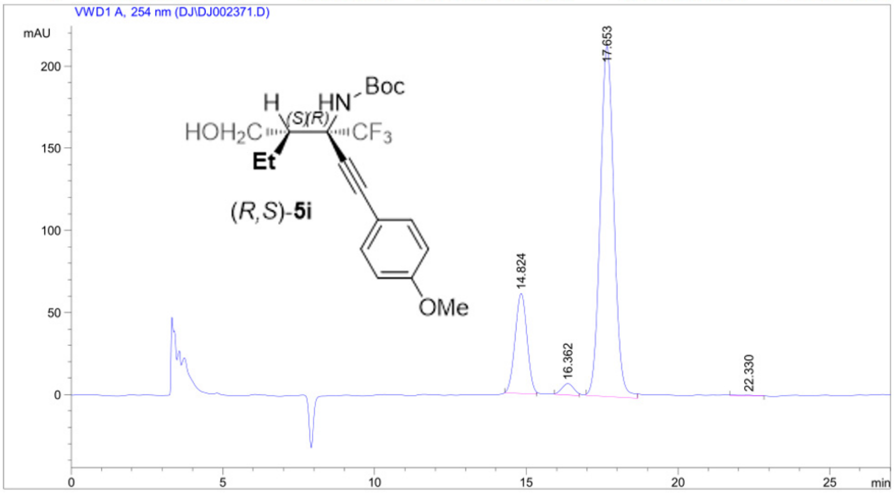

Peak #	RetTime [min]	Type	Width [min]	Area mAU *s	Height [mAU]	Area %
1	14.824	MM	0.4573	1672.19080	60.94326	19.4911
2	16.362	MM	0.4789	209.52579	7.29250	2.4422
3	17.653	MM	0.5213	6675.33838	213.43883	77.8080
4	22.330	MM	0.8626	22.19162	4.28750e-1	0.2587

Supplementary Figure 92. HPLC Spectrum for **5i.**

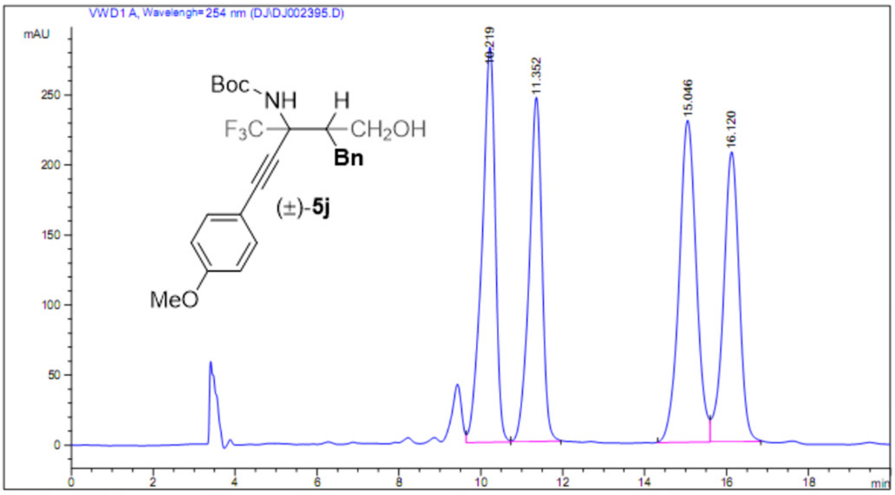

Peak #	RetTime [min]	Type	Width [min]	Area mAU *s	Height [mAU]	Area %
1	10.219	VV	0.3348	6376.02832	282.31946	26.6121
2	11.352	VB	0.3287	5362.10254	245.90195	22.3802
3	15.046	BV	0.4442	6719.24609	230.00104	28.0447
4	16.120	VB	0.4087	5501.71484	207.25943	22.9630

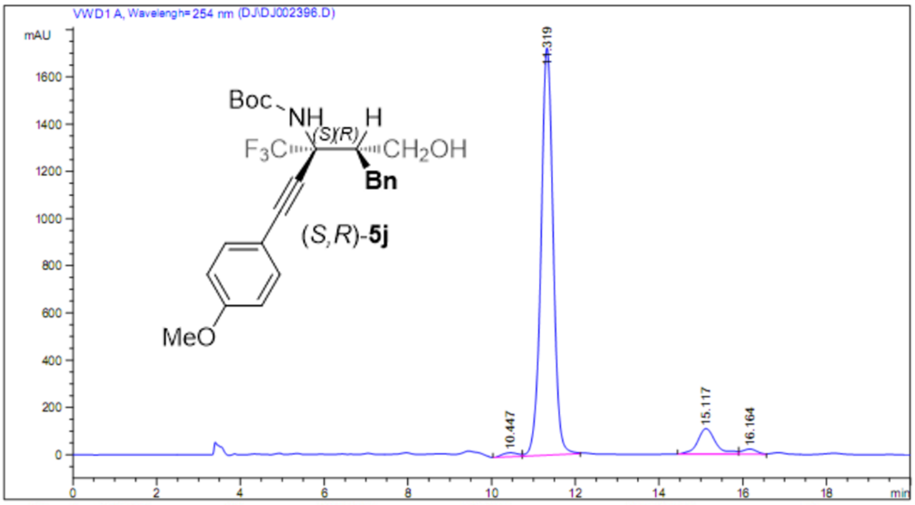

Peak #	RetTime [min]	Type	Width [min]	Area mAU *s	Height [mAU]	Area %
1	10.447	VV	0.4214	448.20432	17.23081	1.1447
2	11.319	VB	0.3127	3.48459e4	1726.21960	88.9974
3	15.117	BB	0.4571	3304.12524	108.53909	8.4388
4	16.164	BV	0.3723	555.58997	22.74912	1.4190

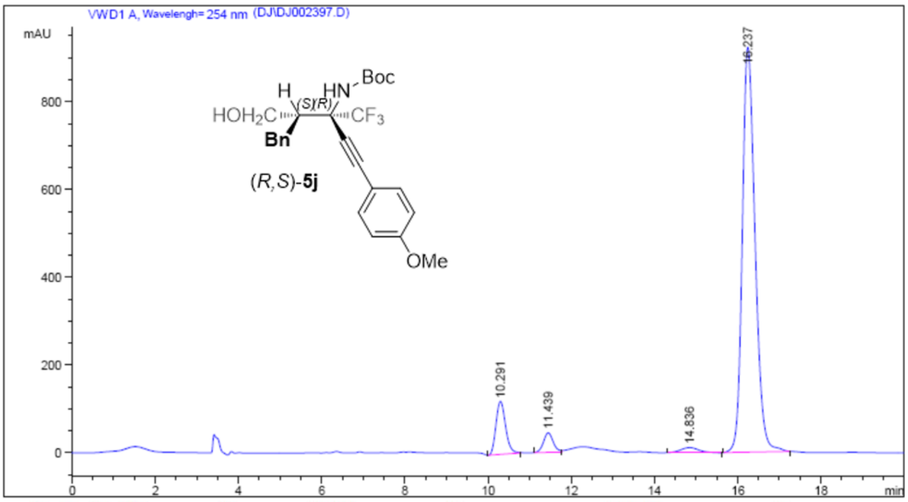

Peak #	RetTime [min]	Type	Width [min]	Area mAU *s	Height [mAU]	Area %
1	10.291	BB	0.2505	1947.60156	120.90959	8.5766
2	11.439	BV	0.2537	749.29993	45.05503	3.2997
3	14.836	BB	0.4684	349.90350	11.64813	1.5409
4	16.237	BB	0.3272	1.96615e4	922.89026	86.5829

Supplementary Figure 93. HPLC Spectrum for **5j.**

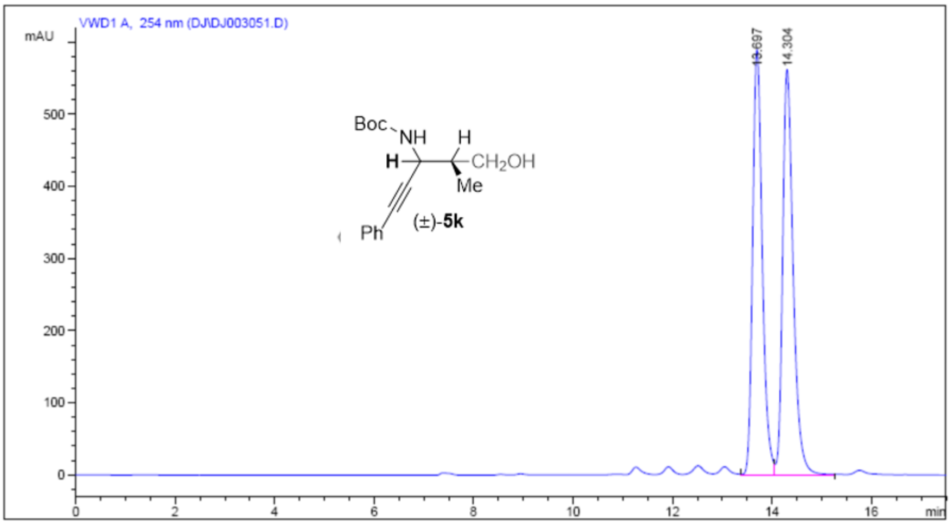

Peak #	RetTime [min]	Type	Width [min]	Area mAU *s	Height [mAU]	Area %
1	13.697	VV	0.2098	7948.51953	589.24994	48.9374
2	14.304	VB	0.2303	8293.70703	562.37811	51.0626

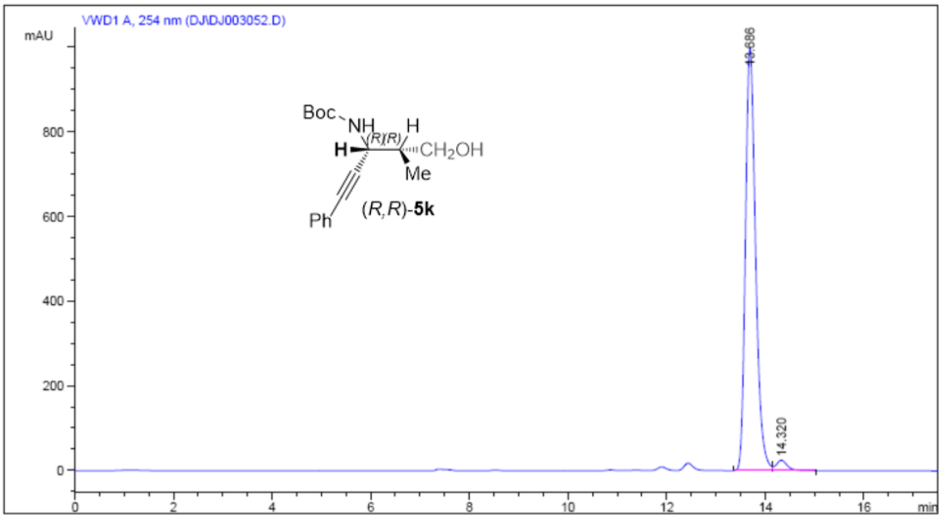

Peak #	RetTime [min]	Type	Width [min]	Area mAU *s	Height [mAU]	Area %
1	13.686	BV	0.2256	1.42245e4	991.69012	97.3585
2	14.320	VB	0.2415	385.93350	24.56618	2.6415

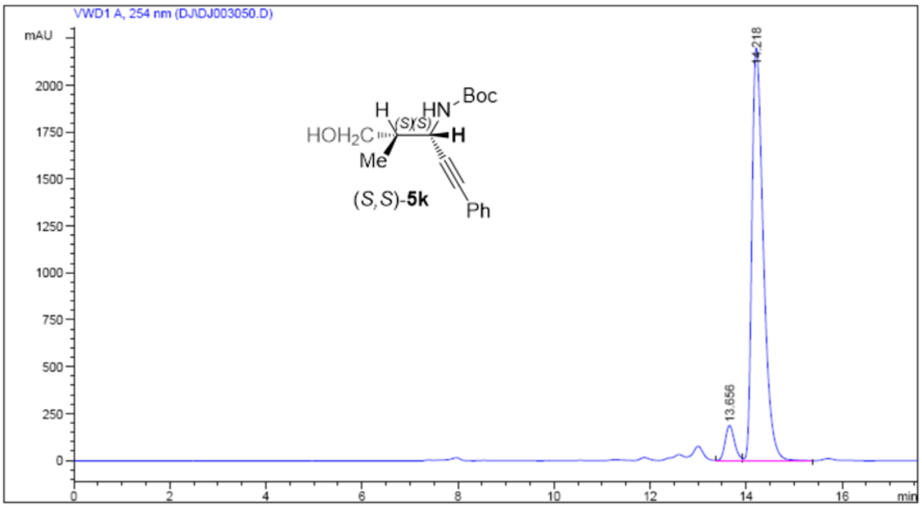

Peak #	RetTime [min]	Type	Width [min]	Area mAU *s	Height [mAU]	Area %
1	13.656	VV	0.2122	2574.54639	187.99557	6.5812
2	14.218	VV	0.2583	3.65451e4	2195.42603	93.4188

Supplementary Figure 94. HPLC Spectrum for **5k.**

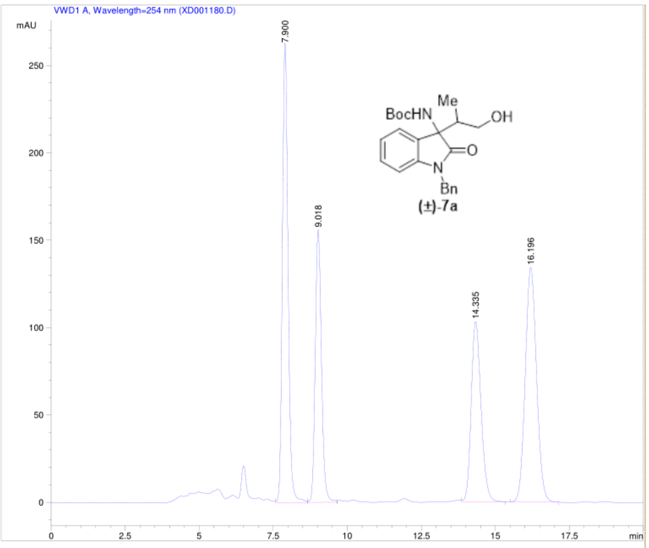

Peak #	RetTime [min]	Type	Width [min]	Area mAU *s	Height [mAU]	Area %
1	7.900	VV	0.2065	3504.93188	262.91690	29.9557
2	9.018	VB	0.2228	2259.72144	156.10149	19.3132
3	14.335	VB	0.3533	2380.33984	103.28003	20.3441
4	16.196	BB	0.4106	3555.39014	134.37750	30.3870

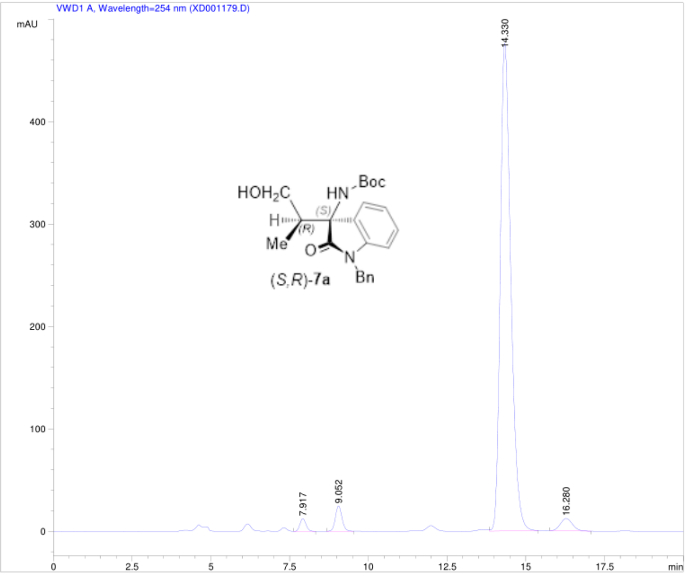

Peak #	RetTime [min]	Type	Width [min]	Area mAU *s	Height [mAU]	Area %
1	7.917	VB	0.2120	167.43877	12.35631	1.4041
2	9.052	BB	0.2285	364.20593	24.74615	3.0541
3	14.330	VB	0.3555	1.10704e4	476.40723	92.8331
4	16.280	PB	0.4151	323.01624	12.08902	2.7087

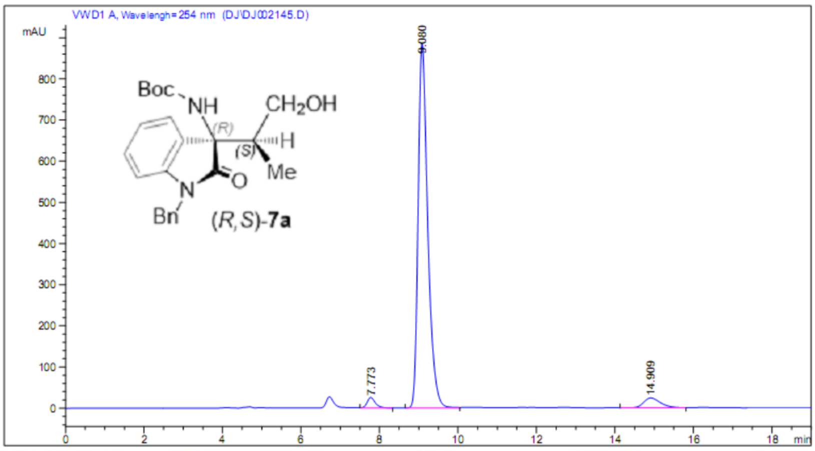

Peak #	RetTime [min]	Type	Width [min]	Area mAU *s	Height [mAU]	Area %
1	7.773	VB	0.2194	373.46701	25.63933	2.3078
2	9.080	BV	0.2583	1.50710e4	885.25720	93.1288
3	14.909	VB	0.4628	738.49579	24.16433	4.5634

Supplementary Figure 95. HPLC Spectrum for **7a.**

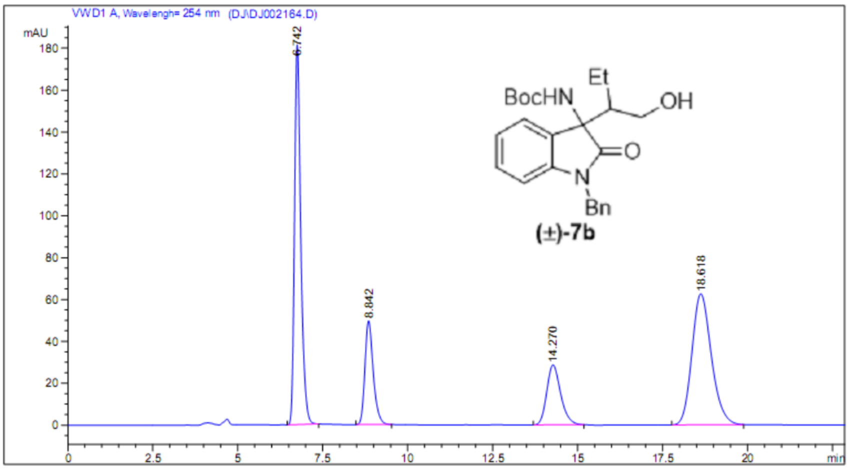

Peak #	RetTime [min]	Type	Width [min]	Area mAU *s	Height [mAU]	Area %
1	6.742	BB	0.1934	2328.54907	181.42346	35.8563
2	8.842	BB	0.2607	848.84576	49.63669	13.0710
3	14.270	BB	0.4357	821.21912	28.70180	12.6456
4	18.618	BB	0.6092	2495.50317	62.60308	38.4271

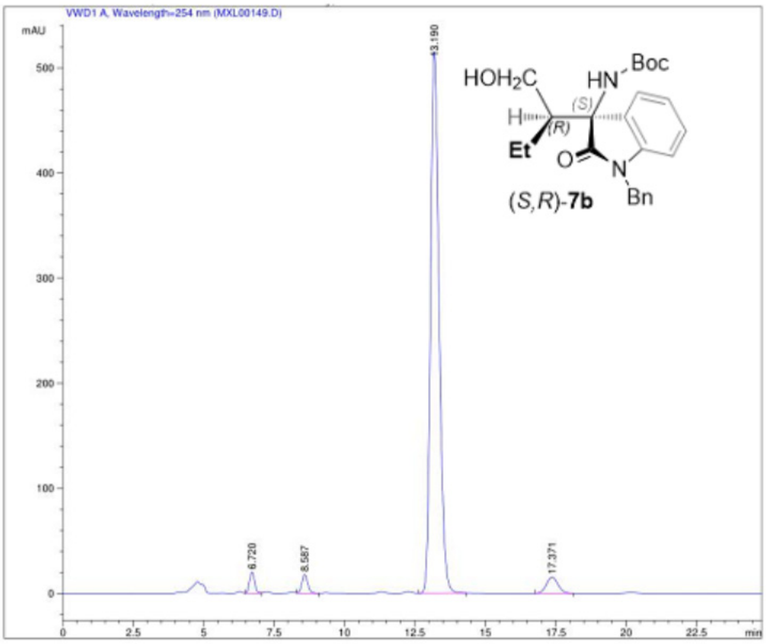

Peak #	RetTime [min]	Type	Width [min]	Area mAU *s	Height [mAU]	Area %
1	6.720	VV	0.2074	263.86383	20.24005	2.2348
2	8.587	VB	0.2225	259.61194	17.96516	2.1988
3	13.190	VB	0.3240	1.08408e4	515.38654	91.8152
4	17.371	BB	0.4456	442.91733	15.49721	3.7513

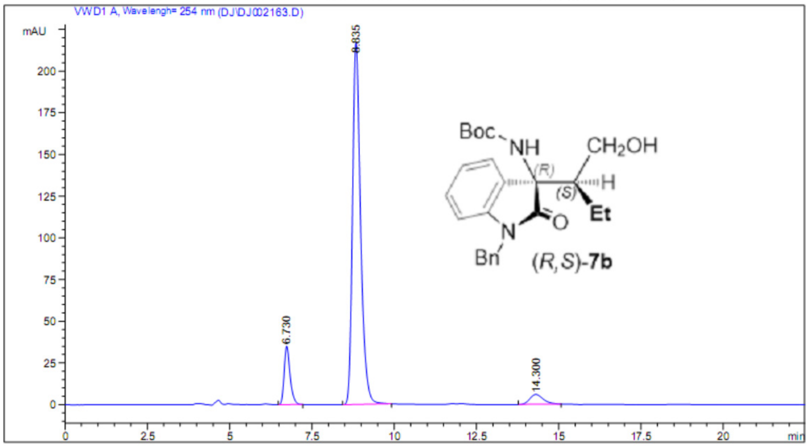

Peak #	RetTime [min]	Type	Width [min]	Area mAU *s	Height [mAU]	Area %
1	6.730	BB	0.1886	440.72897	35.12307	10.1776
2	8.835	BB	0.2595	3716.11548	217.01161	85.8146
3	14.300	BB	0.4380	173.55620	5.99903	4.0079

Supplementary Figure 96. HPLC Spectrum for **7b.**

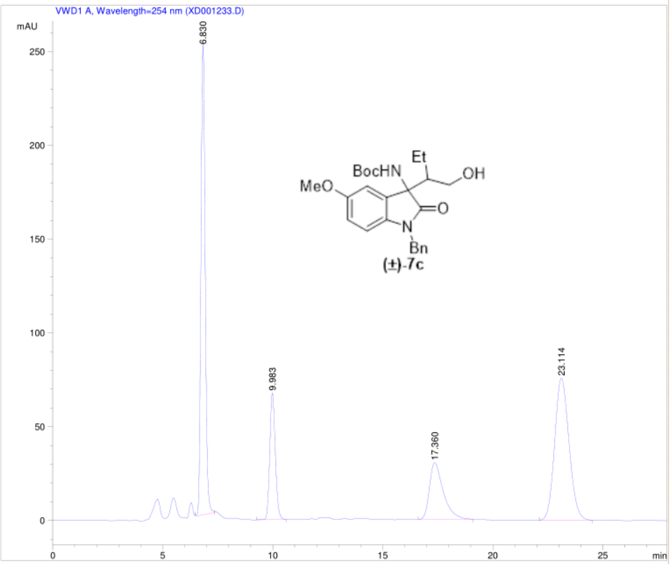

Peak #	RetTime [min]	Type	Width [min]	Area mAU *s	Height [mAU]	Area %
1	6.830	VB	0.2077	3335.05420	250.59618	35.8910
2	9.983	BB	0.2695	1179.83081	67.48739	12.6970
3	17.360	BB	0.6604	1338.52332	30.33161	14.4048
4	23.114	BB	0.6997	3438.77661	75.79308	37.0072

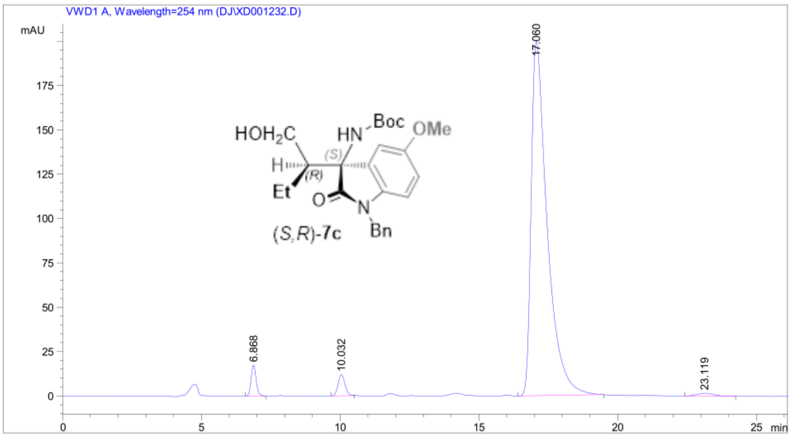

Peak #	RetTime [min]	Type	Width [min]	Area mAU *s	Height [mAU]	Area %
1	6.868	BB	0.1978	221.49245	17.42886	2.6005
2	10.032	BB	0.2696	209.46434	12.06082	2.4593
3	17.060	VB	0.5887	8014.19971	199.83092	94.0936
4	23.119	BB	0.5201	72.10886	1.65473	0.8466

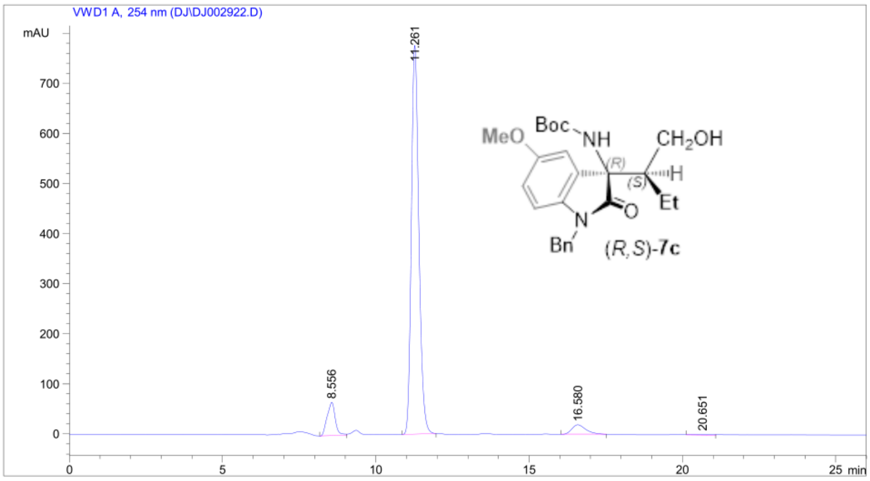

Peak #	RetTime [min]	Type	Width [min]	Area mAU *s	Height [mAU]	Area %
1	8.556	VV	0.2655	1239.20557	66.92921	8.1796
2	11.261	BB	0.2624	1.32075e4	776.71130	87.1785
3	16.580	BB	0.4873	630.36676	19.15558	4.1608
4	20.651	MM	0.7681	72.88160	1.58145	0.4811

Supplementary Figure 97. HPLC Spectrum for **7c.**

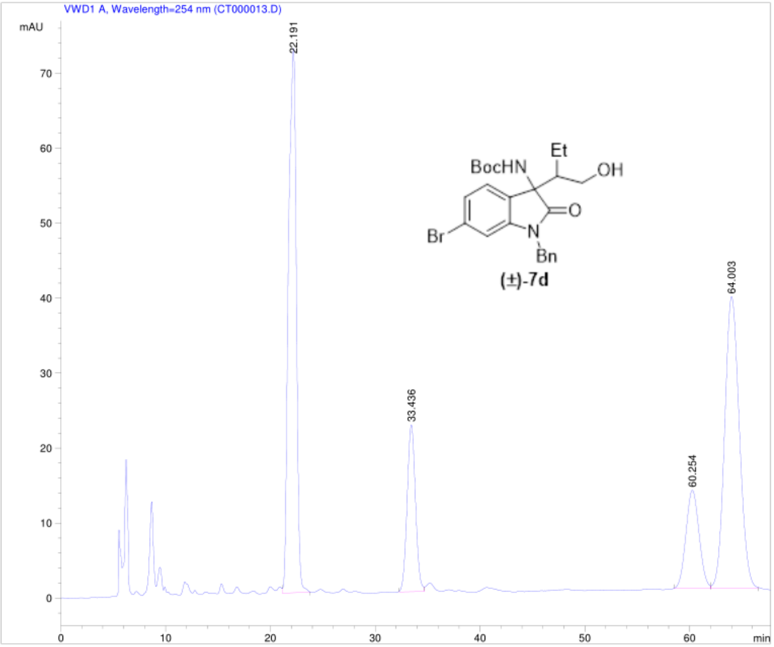

Peak #	RetTime [min]	Type	Width [min]	Area mAU *s	Height [mAU]	Area %
1	22.191	VB	0.7219	3759.34790	72.61868	37.9281
2	33.436	BV	0.8471	1236.03223	22.26492	12.4704
3	60.254	BV	1.2441	1140.73132	13.10565	11.5089
4	64.003	VB	1.4446	3775.65137	38.87944	38.0926

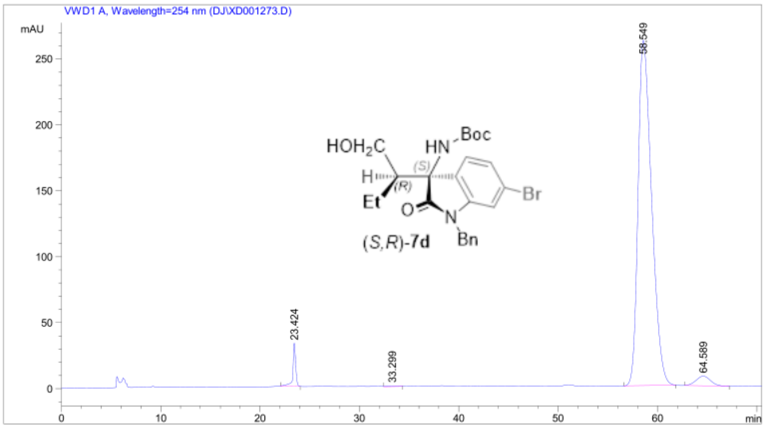

Peak #	RetTime [min]	Type	Width [min]	Area mAU *s	Height [mAU]	Area %
1	23.424	BB	0.2894	650.25122	32.81773	2.4713
2	33.299	MM	1.4527	59.24539	6.79714e-1	0.2252
3	58.549	BB	1.3606	2.48721e4	262.06644	94.5257
4	64.589	BB	1.1907	730.91644	7.27750	2.7778

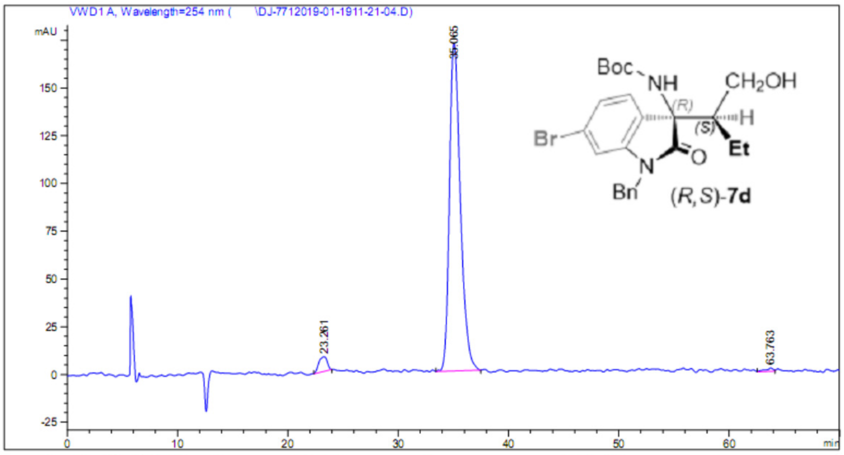

Peak #	RetTime [min]	Type	Width [min]	Area [mAU*s]	Height [mAU]	Area %
1	23.261	BB	0.6621	405.48203	7.66580	3.2912
2	35.065	VB R	0.9734	1.18346e4	171.43272	96.0580
3	63.763	BV	0.5950	80.18317	1.70383	0.6508

Supplementary Figure 98. HPLC Spectrum for **7d.**

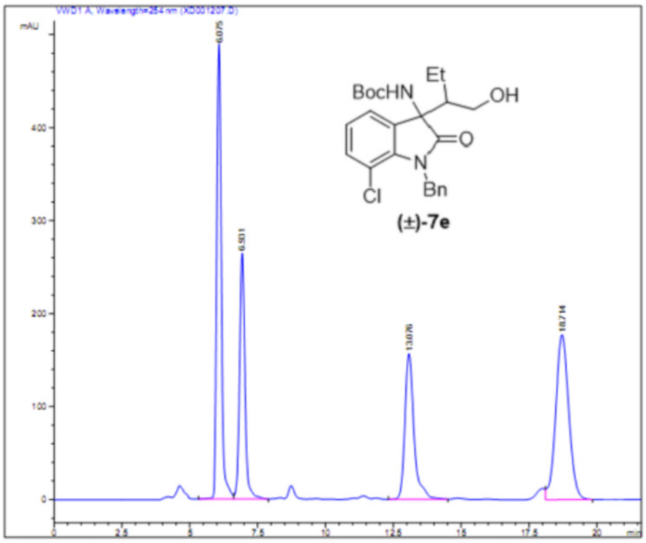

Peak #	RetTime [min]	Type	Width [min]	Area mAU *s	Height [mAU]	Area %
1	6.075	BV	0.1876	5865.58252	490.49036	30.5429
2	6.931	VB	0.2114	3682.82202	265.31702	19.1770
3	13.076	VB	0.3583	3725.66602	157.07729	19.4001
4	18.714	VB	0.5183	5930.30859	177.32228	30.8800

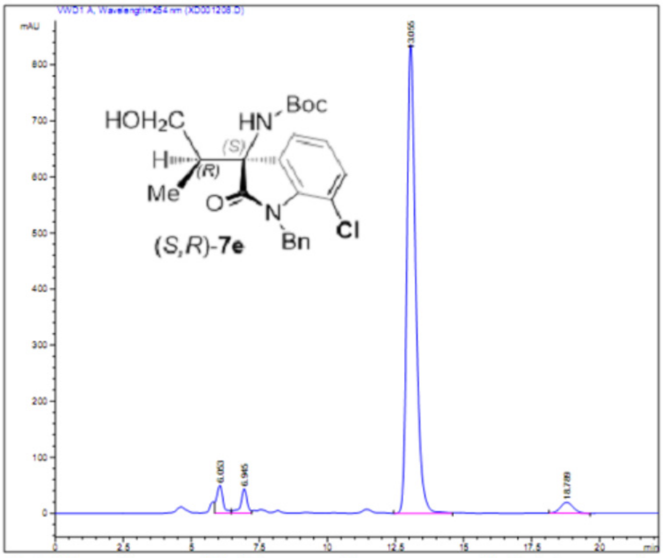

Peak #	RetTime [min]	Type	Width [min]	Area mAU *s	Height [mAU]	Area %
1	6.053	VV	0.2496	812.03674	49.88554	3.8797
2	6.945	VV	0.2399	708.02295	43.72863	3.3827
3	13.055	VB	0.3435	1.87832e4	836.40869	89.7402
4	18.789	BB	0.5019	627.38037	19.35527	2.9974

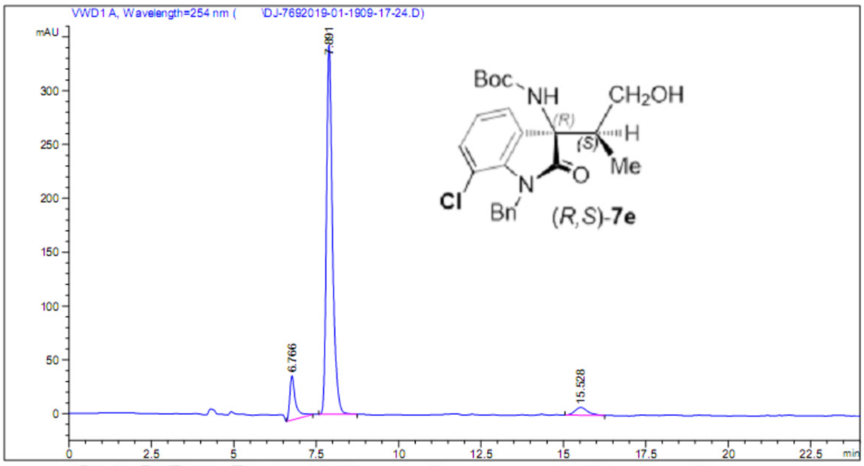

Peak #	RetTime [min]	Type	Width [min]	Area [mAU*s]	Height [mAU]	Area %
1	6.766	BB	0.1750	496.04025	41.04439	9.7865
2	7.891	BB	0.1940	4382.41064	343.24030	86.4613
3	15.528	BB	0.4098	190.18852	7.16210	3.7523

Supplementary Figure 99. HPLC Spectrum for **7e.**

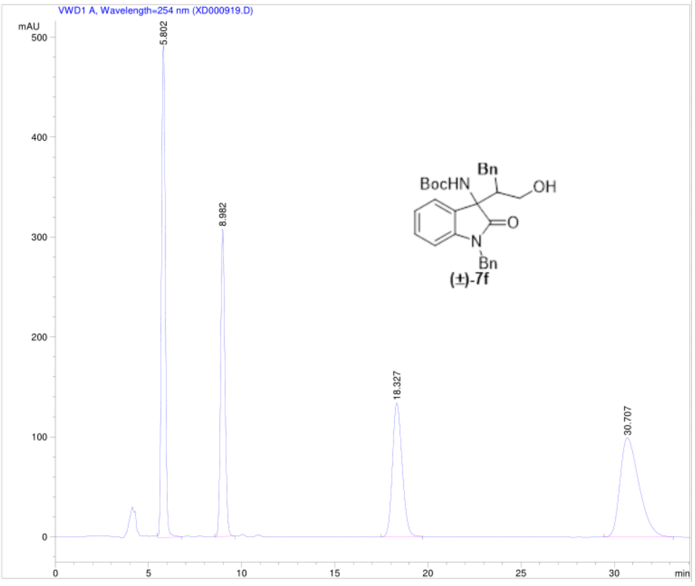

Peak #	RetTime [min]	Type	Width [min]	Area mAU *s	Height [mAU]	Area %
1	5.802	VB	0.2329	7062.78027	492.32367	29.5331
2	8.982	PB	0.2508	4965.42725	307.82114	20.7630
3	18.327	BB	0.5622	4852.00537	133.99635	20.2887
4	30.707	BB	1.0856	7034.56494	99.31020	29.4151

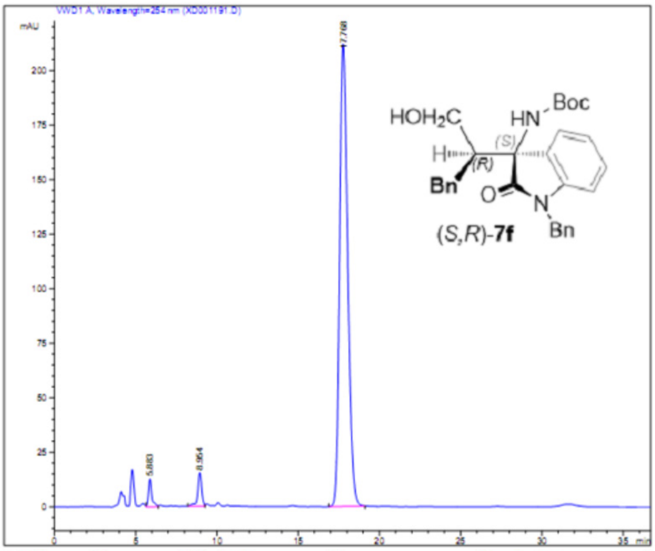

Peak #	RetTime [min]	Type	Width [min]	Area mAU *s	Height [mAU]	Area %
1	5.883	VB	0.2190	185.55945	12.77027	2.3891
2	8.954	BV	0.2612	267.63705	15.49492	3.4459
3	17.768	BB	0.5336	7313.63623	211.93547	94.1650

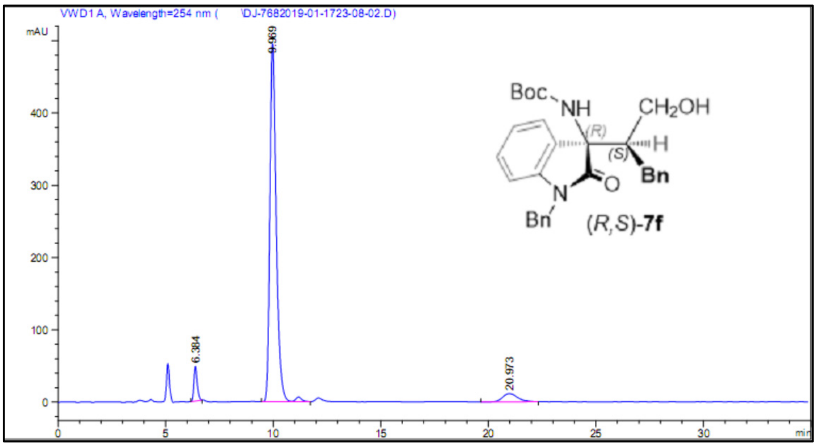

Peak #	RetTime [min]	Type	Width [min]	Area [mAU*s]	Height [mAU]	Area %
1	6.384	BB	0.1610	513.28918	48.29683	4.5213
2	9.969	BV R	0.3120	1.02719e4	495.36624	90.4807
3	20.973	BB	0.7466	567.40668	11.44081	4.9980

Supplementary Figure 100. HPLC Spectrum for **7f.**

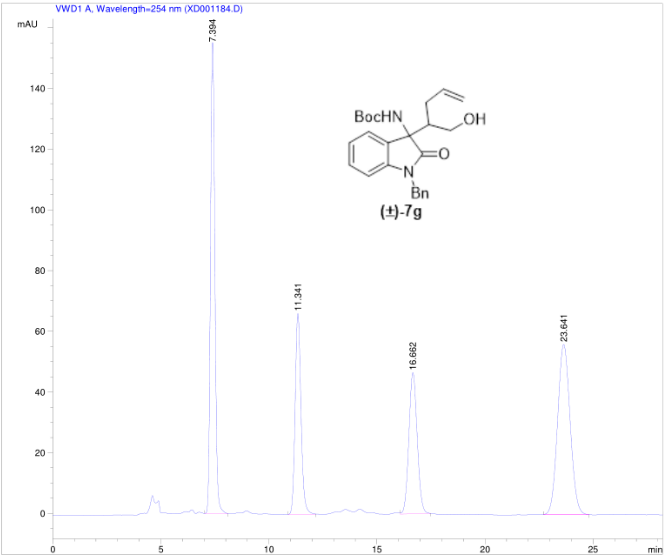

Peak #	RetTime [min]	Type	Width [min]	Area mAU *s	Height [mAU]	Area %
1	7.394	VB	0.2231	2193.62549	155.24570	31.5768
2	11.341	BB	0.2891	1243.43848	66.16306	17.8990
3	16.662	VB	0.4162	1248.56689	46.56895	17.9729
4	23.641	BB	0.6258	2261.32813	56.12806	32.5513

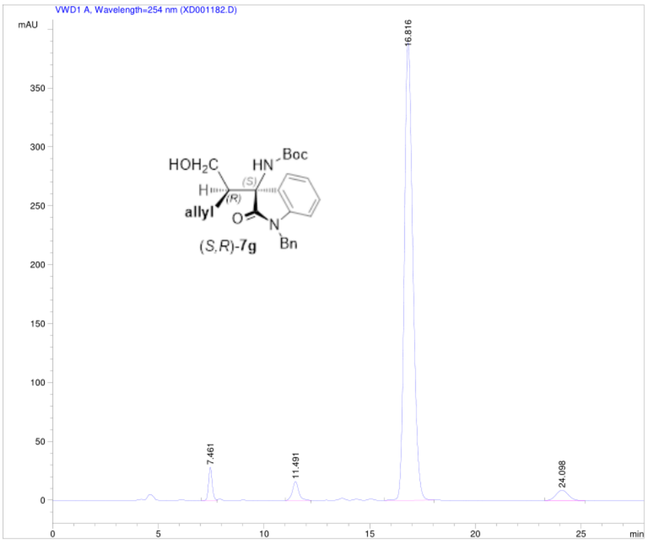

Peak #	RetTime [min]	Type	Width [min]	Area mAU *s	Height [mAU]	Area %
1	7.461	VV	0.1874	345.28036	28.31489	2.9113
2	11.491	BB	0.3102	335.69525	16.10392	2.8305
3	16.816	BB	0.4292	1.08124e4	388.86557	91.1686
4	24.098	BB	0.6262	366.41364	8.86773	3.0895

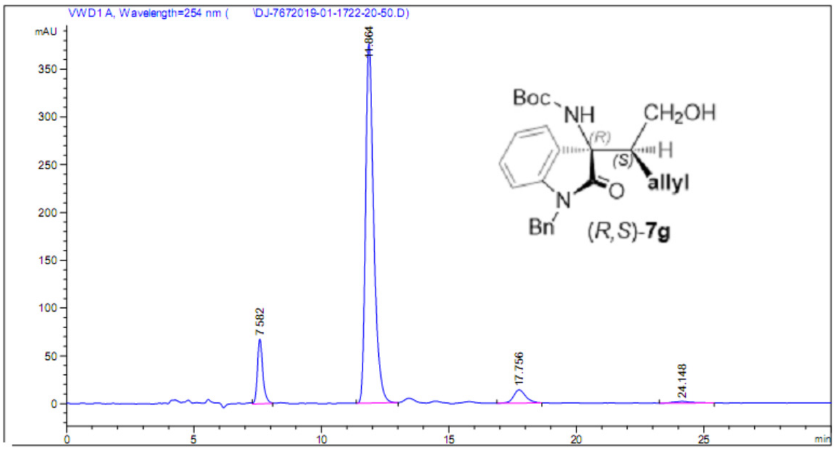

Peak #	RetTime [min]	Type	Width [min]	Area [mAU*s]	Height [mAU]	Area %
1	7.582	MM	0.2455	996.42108	67.65942	10.0196
2	11.864	BB	0.3364	8403.92578	376.53568	84.5068
3	17.756	BB	0.5023	452.23672	13.69217	4.5475
4	24.148	BB	0.6225	92.08834	1.90989	0.9260

Supplementary Figure 101. HPLC Spectrum for **7g.**

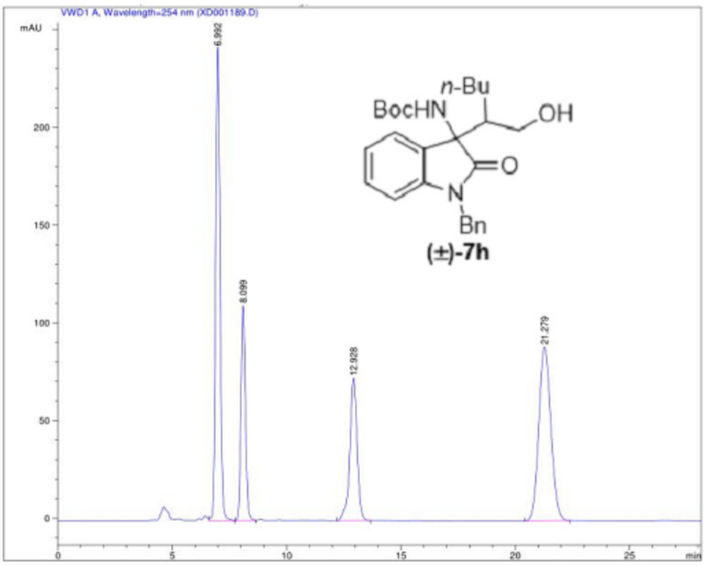

Peak #	RetTime [min]	Type	Width [min]	Area mAU *s	Height [mAU]	Area %
1	6.992	VB	0.2096	3242.00635	242.83875	33.6707
2	8.099	BB	0.2108	1494.42187	110.09826	15.5207
3	12.928	BB	0.3386	1626.57959	72.98550	16.8933
4	21.279	BB	0.5726	3265.56445	88.92184	33.9154

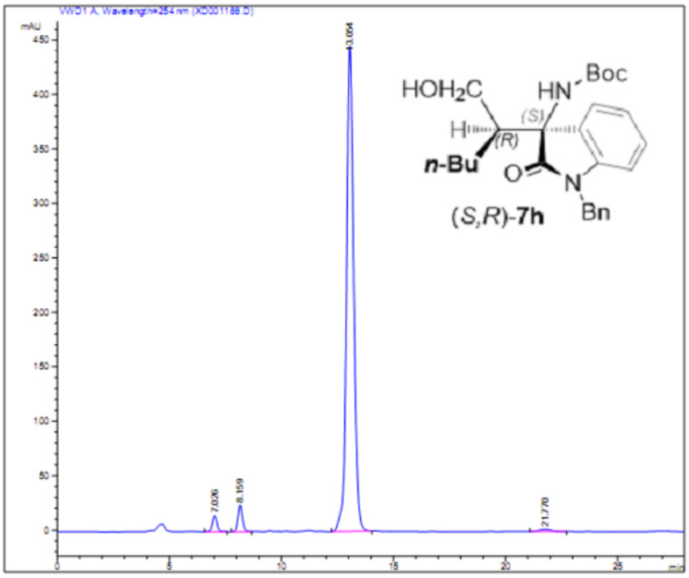

Peak #	RetTime [min]	Type	Width [min]	Area mAU *s	Height [mAU]	Area %
1	7.026	VB	0.2255	212.95267	14.72353	2.0310
2	8.159	PB	0.2163	342.53610	24.38791	3.2669
3	13.054	BB	0.3379	9853.87207	445.83743	93.9809
4	21.770	BB	0.4985	75.61081	2.05117	0.7211

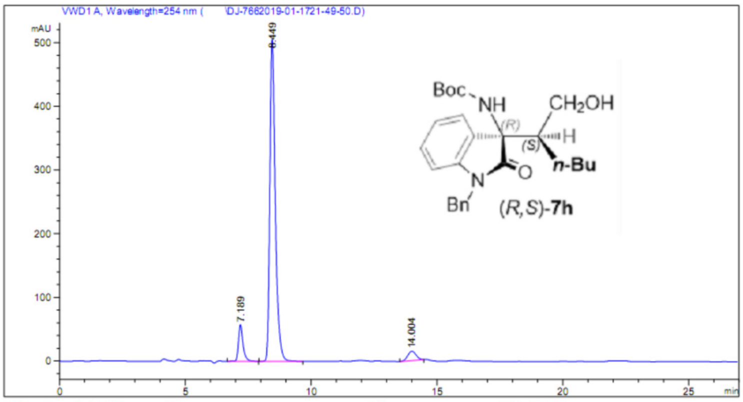

Peak #	RetTime [min]	Type	Width [min]	Area [mAU*s]	Height [mAU]	Area %
1	7.189	VB R	0.1877	720.94751	57.72219	8.2992
2	8.449	BB	0.2279	7630.95801	506.13452	87.8443
3	14.004	BB	0.3611	335.01160	14.52565	3.8565

Supplementary Figure 102. HPLC Spectrum for **7h.**

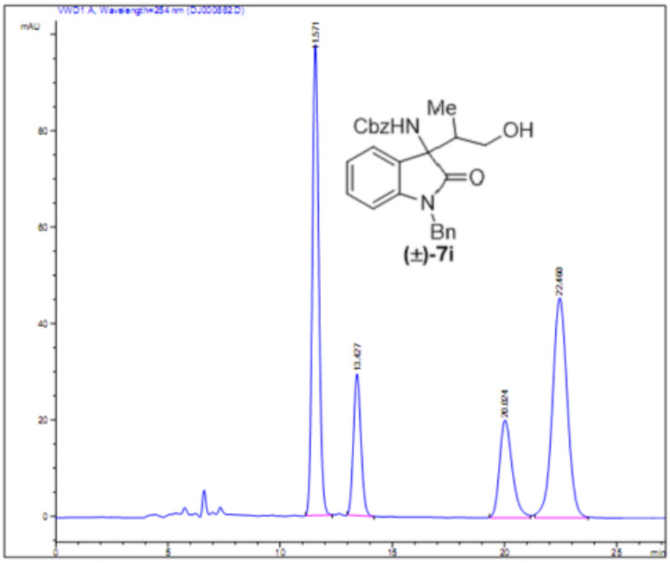

Peak #	RetTime [min]	Type	Width [min]	Area mAU *s	Height [mAU]	Area %
1	11.571	BB	0.3089	1952.31152	97.67686	35.3362
2	13.427	BP	0.3619	683.49646	29.35252	12.3711
3	20.024	BB	0.5975	794.10388	20.24059	14.3730
4	22.460	BB	0.7109	2095.05322	45.58972	37.9198

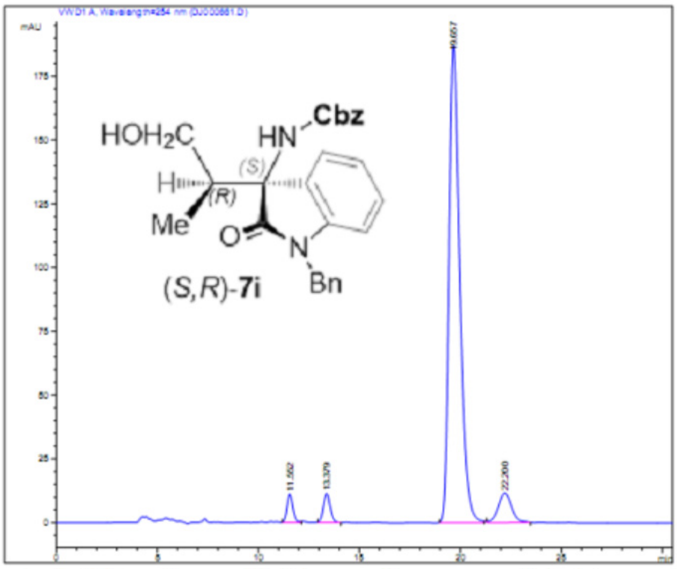

Peak #	RetTime [min]	Type	Width [min]	Area mAU *s	Height [mAU]	Area %
1	11.552	BB	0.3110	223.56282	11.08533	2.8220
2	13.379	BP	0.3628	264.04727	11.30180	3.3330
3	19.657	BB	0.5646	6904.11328	187.05997	87.1494
4	22.200	BB	0.7093	530.43915	11.57531	6.6956

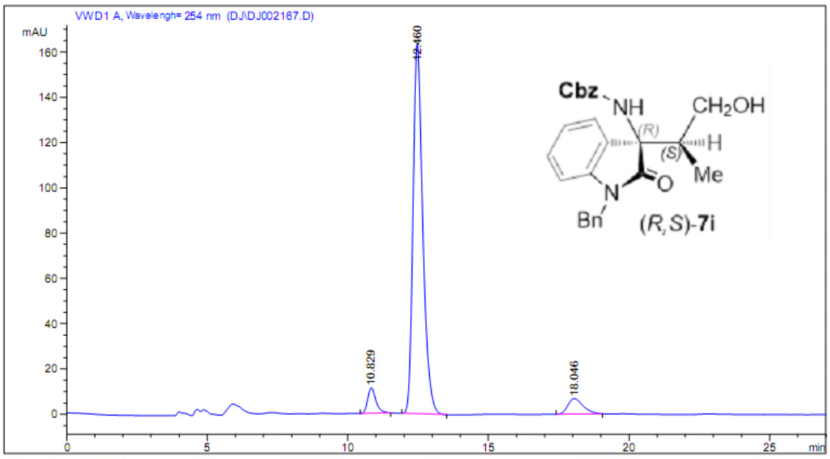

Peak #	RetTime [min]	Type	Width [min]	Area mAU *s	Height [mAU]	Area %
1	10.829	BB	0.3113	232.80655	11.31898	5.2495
2	12.460	BB	0.3671	3938.06421	163.37607	88.7989
3	18.046	BB	0.5702	263.93887	6.92063	5.9515

Supplementary Figure 103. HPLC Spectrum for **7i.**

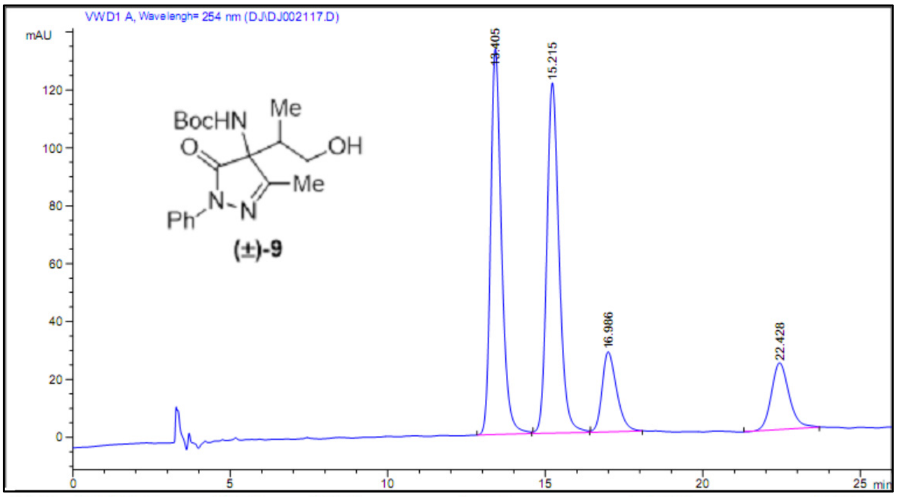

Peak #	RetTime [min]	Type	Width [min]	Area mAU *s	Height [mAU]	Area %
1	13.405	BB	0.3648	3205.41504	133.38287	38.6166
2	15.215	BB	0.4125	3282.88379	121.06412	39.5499
3	16.986	BB	0.4918	896.81989	27.66469	10.8043
4	22.428	BB	0.6002	915.49127	23.12497	11.0292

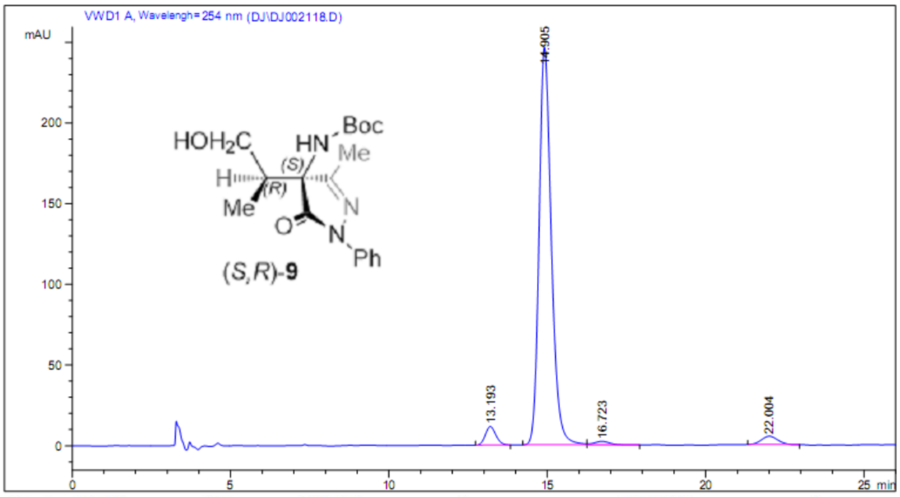

Peak #	RetTime [min]	Type	Width [min]	Area mAU *s	Height [mAU]	Area %
1	13.193	BB	0.3595	269.02731	11.53385	3.7590
2	14.905	BB	0.4094	6619.35791	246.48860	92.4897
3	16.723	BB	0.5192	80.39144	2.27924	1.1233
4	22.004	BB	0.5529	188.08228	5.20133	2.6280

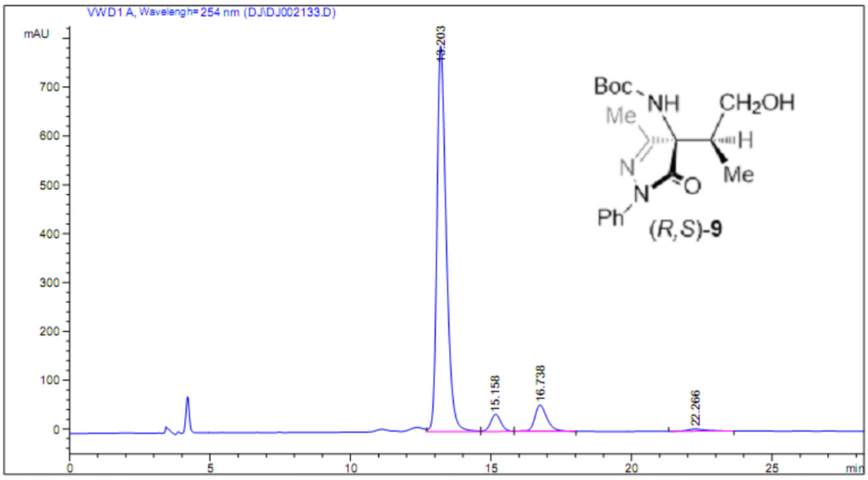

Peak #	RetTime [min]	Type	Width [min]	Area mAU *s	Height [mAU]	Area %
1	13.203	VB	0.3514	1.81981e4	790.90393	86.6320
2	15.158	BV	0.4004	934.59882	35.65981	4.4492
3	16.738	VB	0.4594	1667.99805	54.21575	7.9405
4	22.266	BB	0.6680	205.51144	4.39353	0.9783

Supplementary Figure 104. HPLC Spectrum for **9.**

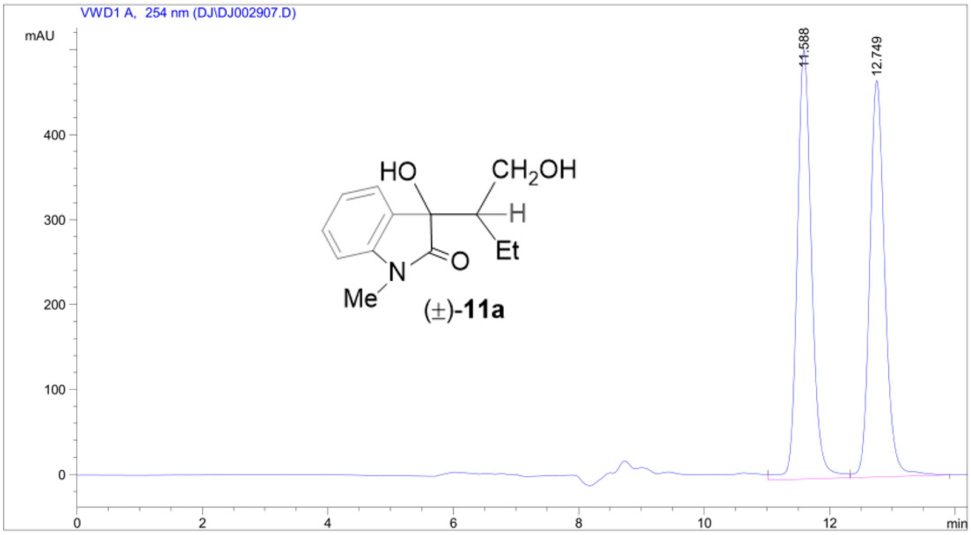

Peak #	RetTime [min]	Type	Width [min]	Area mAU *s	Height [mAU]	Area %
1	11.588	VV	0.2426	8124.32764	506.08484	50.8877
2	12.749	VB	0.2584	7840.87988	467.22644	49.1123

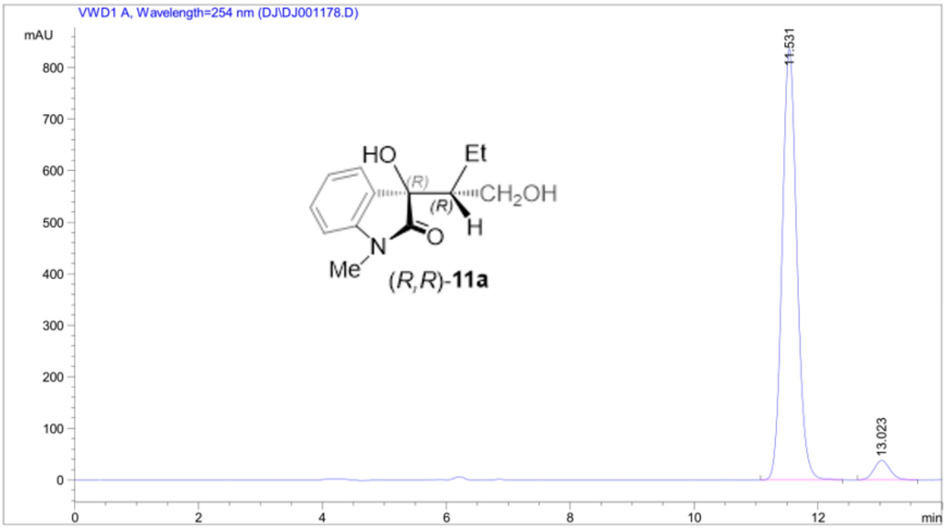

Peak #	RetTime [min]	Type	Width [min]	Area mAU *s	Height [mAU]	Area %
1	11.531	BB	0.2569	1.38230e4	836.35583	95.3738
2	13.023	BB	0.2766	670.49432	37.58277	4.6262

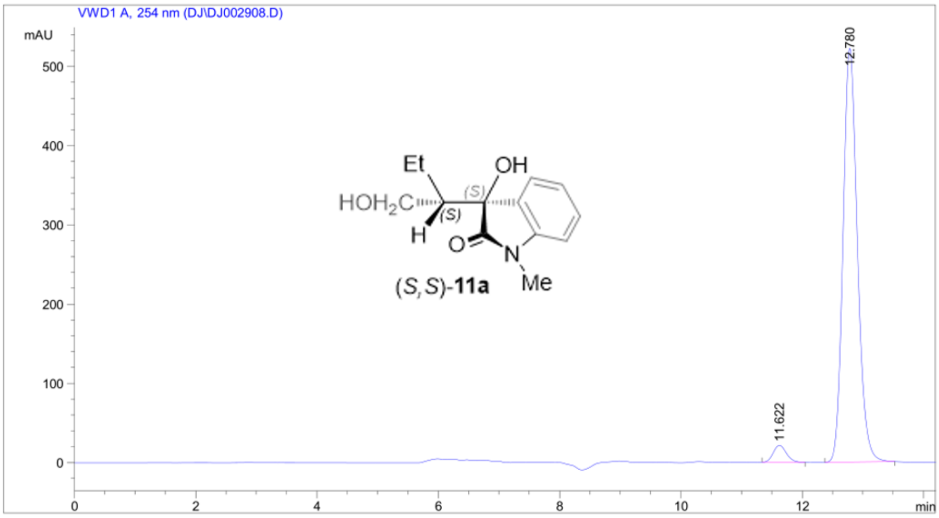

Peak #	RetTime [min]	Type	Width [min]	Area mAU *s	Height [mAU]	Area %
1	11.622	BB	0.2377	325.65891	21.51822	3.6445
2	12.780	BB	0.2563	8609.90332	522.66150	96.3555

Supplementary Figure 105. HPLC Spectrum for **11a.**

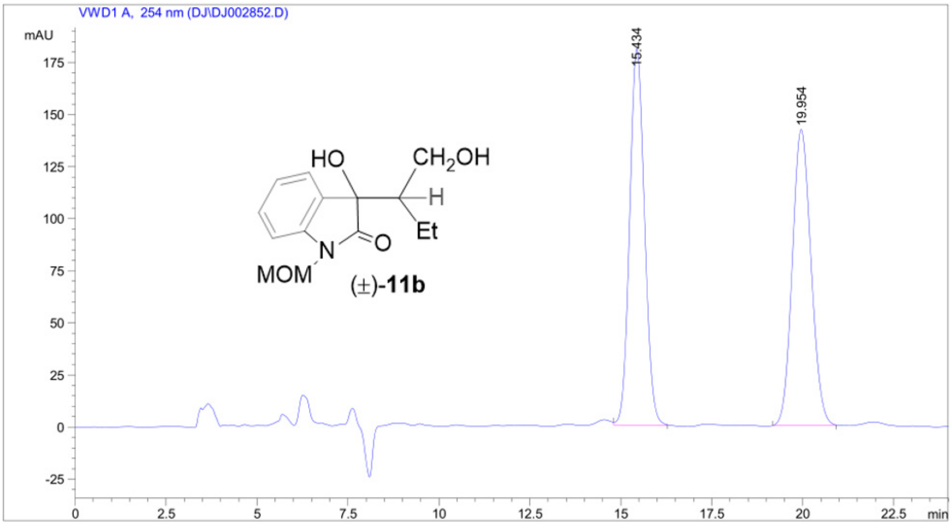

Peak #	RetTime [min]	Type	Width [min]	Area mAU *s	Height [mAU]	Area %
1	15.434	VB	0.4436	5155.35596	180.67175	50.1909
2	19.954	BB	0.5626	5116.14307	142.14583	49.8091

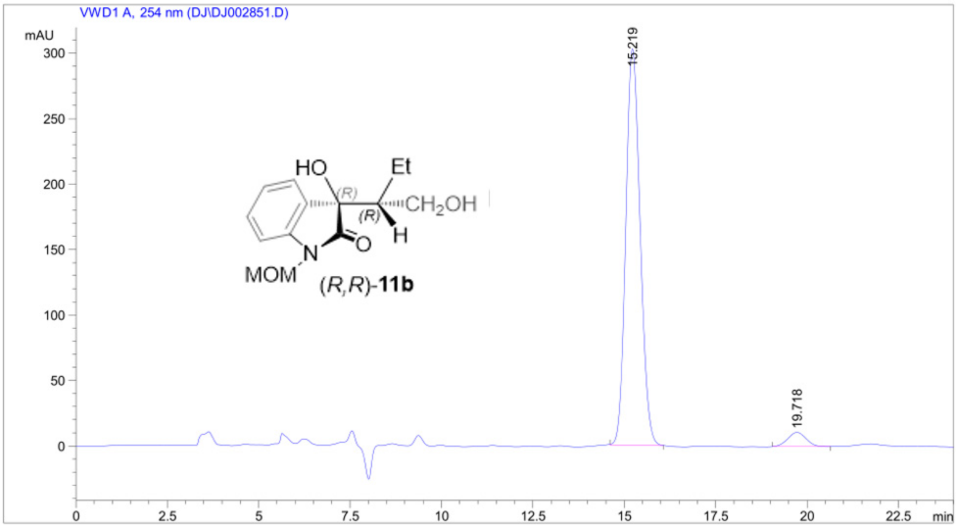

Peak #	RetTime [min]	Type	Width [min]	Area mAU *s	Height [mAU]	Area %
1	15.219	BB	0.4270	8285.44238	302.81927	95.6003
2	19.718	BB	0.5448	381.31094	10.94527	4.3997

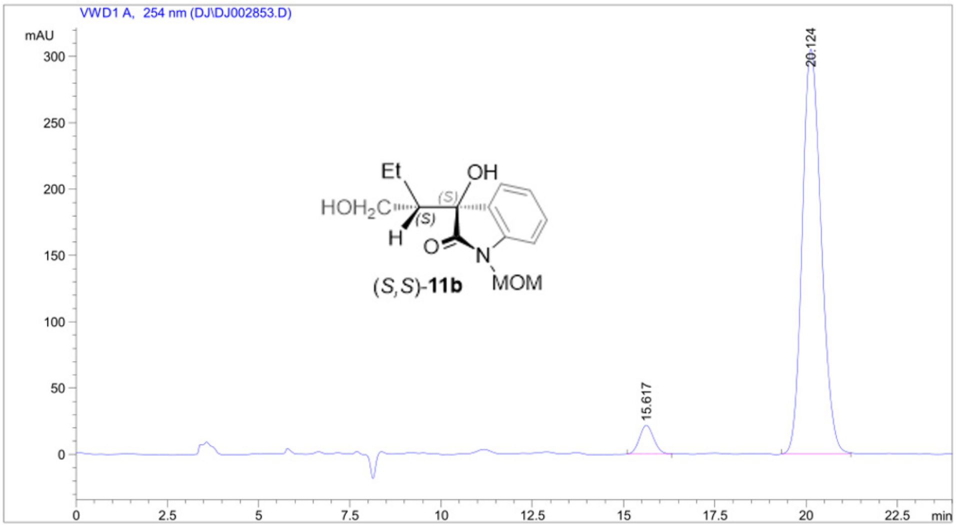

Peak #	RetTime [min]	Type	Width [min]	Area mAU *s	Height [mAU]	Area %
1	15.617	BB	0.4214	589.86218	21.73616	5.0936
2	20.124	BB	0.5598	1.09905e4	305.24347	94.9064

Supplementary Figure 106. HPLC Spectrum for **11b.**

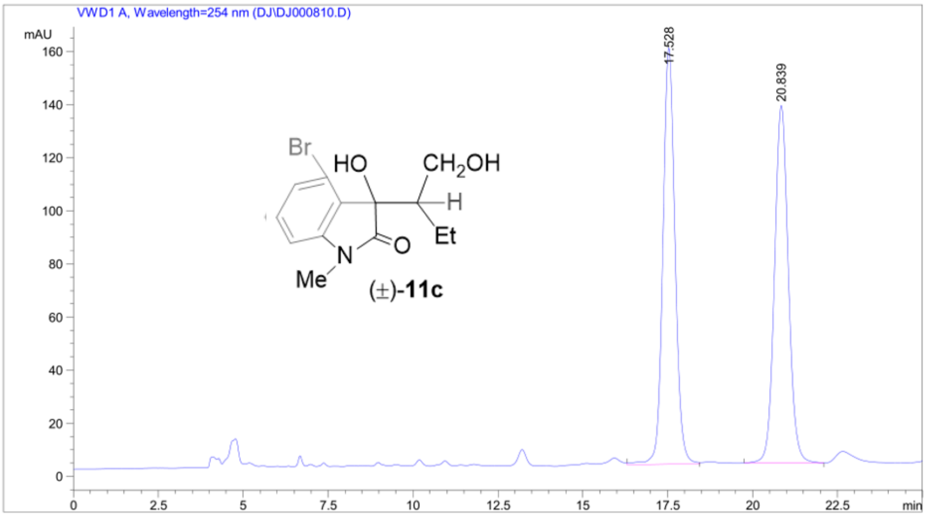

Peak #	RetTime [min]	Type	Width [min]	Area mAU *s	Height [mAU]	Area %
1	17.528	VB	0.3813	3892.23584	156.81693	50.4925
2	20.839	BB	0.4370	3816.30811	134.64302	49.5075

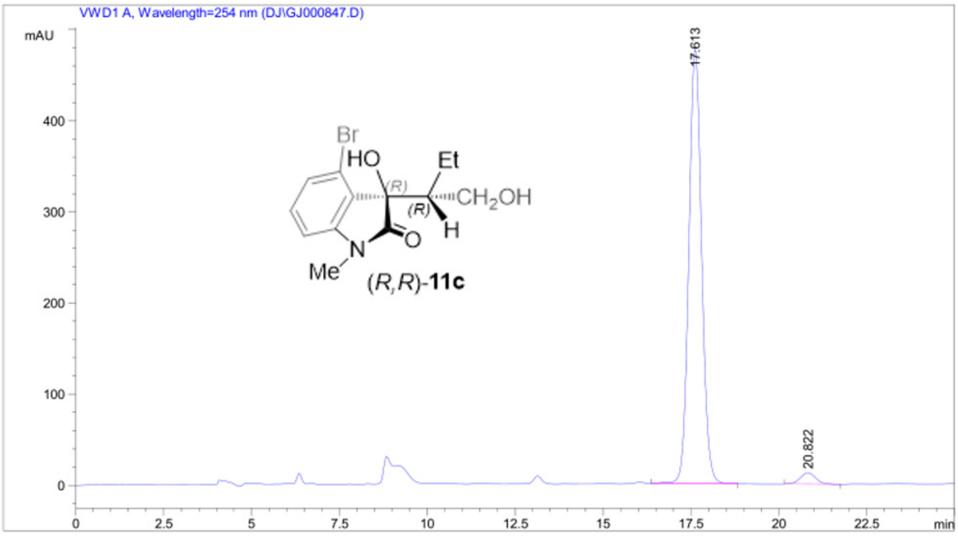

Peak #	RetTime [min]	Type	Width [min]	Area mAU *s	Height [mAU]	Area %
1	17.613	VB	0.3928	1.21260e4	476.78525	96.8644
2	20.822	BB	0.4792	392.53778	12.57378	3.1356

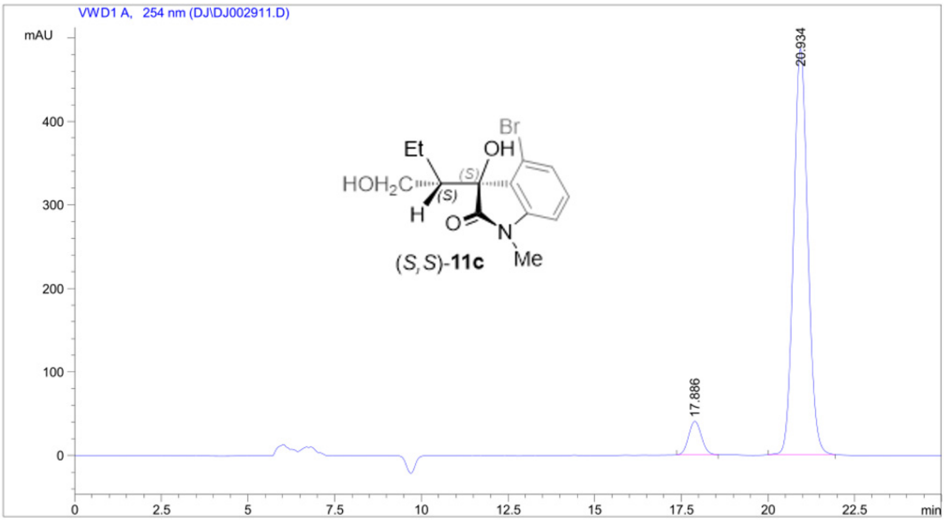

Peak #	RetTime [min]	Type	Width [min]	Area mAU *s	Height [mAU]	Area %
1	17.886	BB	0.3945	1040.95081	40.30560	6.9378
2	20.934	BB	0.4279	1.39632e4	486.74847	93.0622

Supplementary Figure 107. HPLC Spectrum for **11c.**

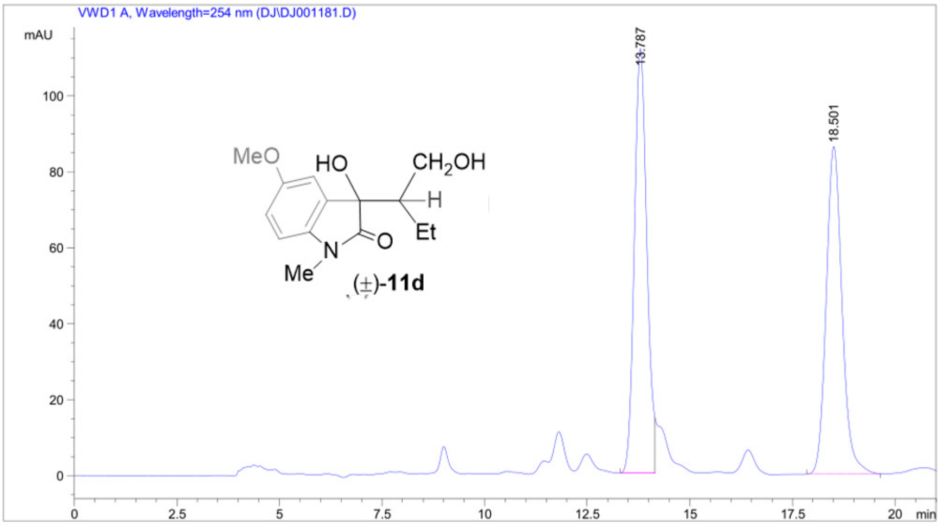

Peak #	RetTime [min]	Type	Width [min]	Area mAU *s	Height [mAU]	Area %
1	13.787	MM	0.3538	2375.03662	111.88577	50.5917
2	18.501	BB	0.4140	2319.47754	86.31737	49.4083

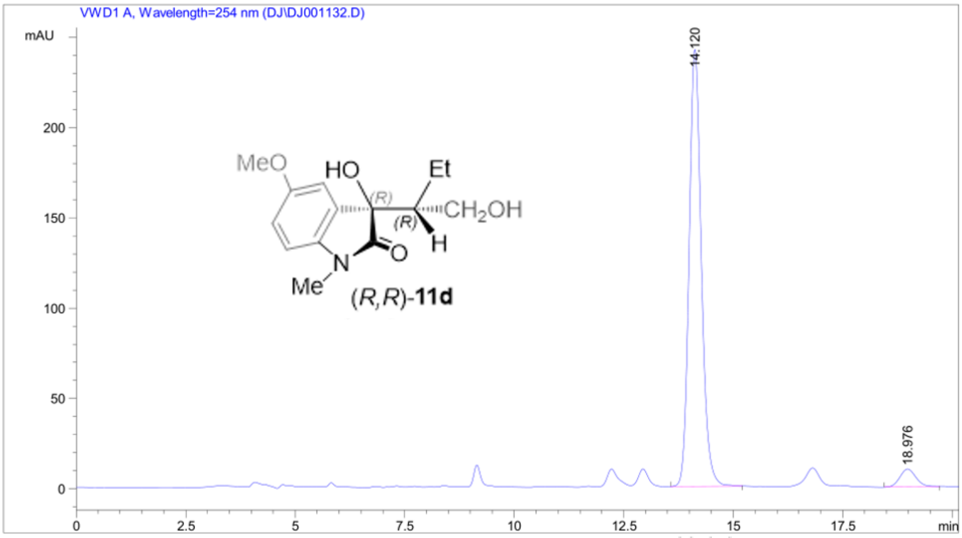

Peak #	RetTime [min]	Type	Width [min]	Area mAU *s	Height [mAU]	Area %
1	14.120	BB	0.2975	4692.04004	242.00201	94.8467
2	18.976	BB	0.3933	254.93237	9.91122	5.1533

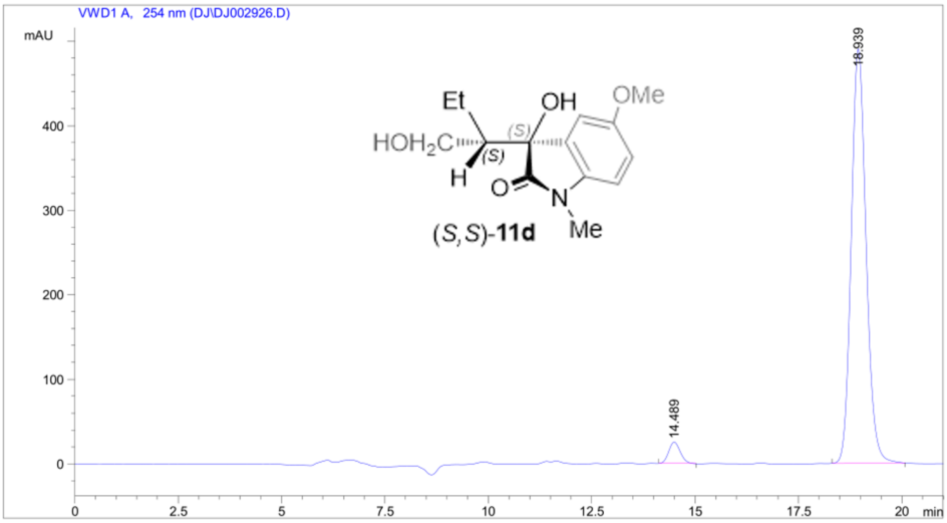

Peak #	RetTime [min]	Type	Width [min]	Area mAU *s	Height [mAU]	Area %
1	14.489	BB	0.3066	500.19037	25.43963	3.9290
2	18.939	BB	0.3826	1.22307e4	490.48605	96.0710

Supplementary Figure 108. HPLC Spectrum for **11d.**

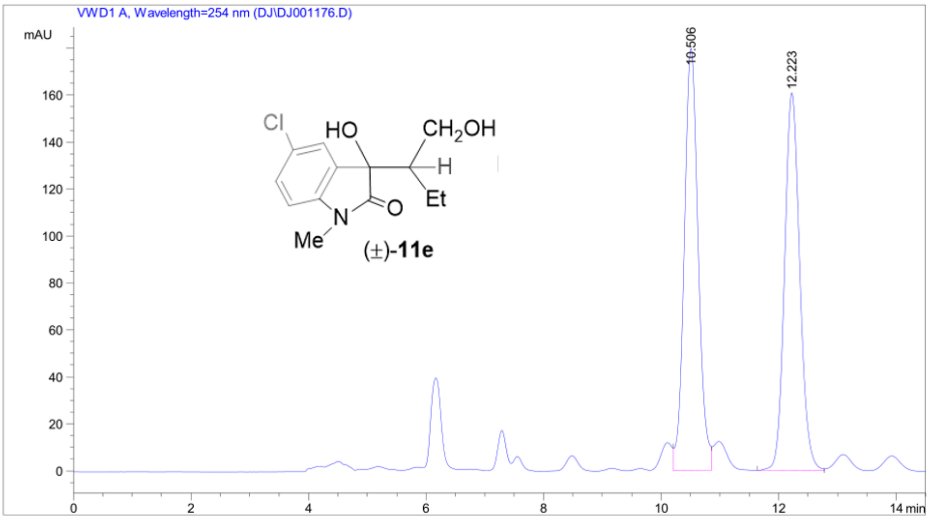

Peak #	RetTime [min]	Type	Width [min]	Area mAU *s	Height [mAU]	Area %
1	10.506	VV	0.2531	2959.25342	179.83696	50.5472
2	12.223	BV	0.2784	2895.18286	160.88260	49.4528

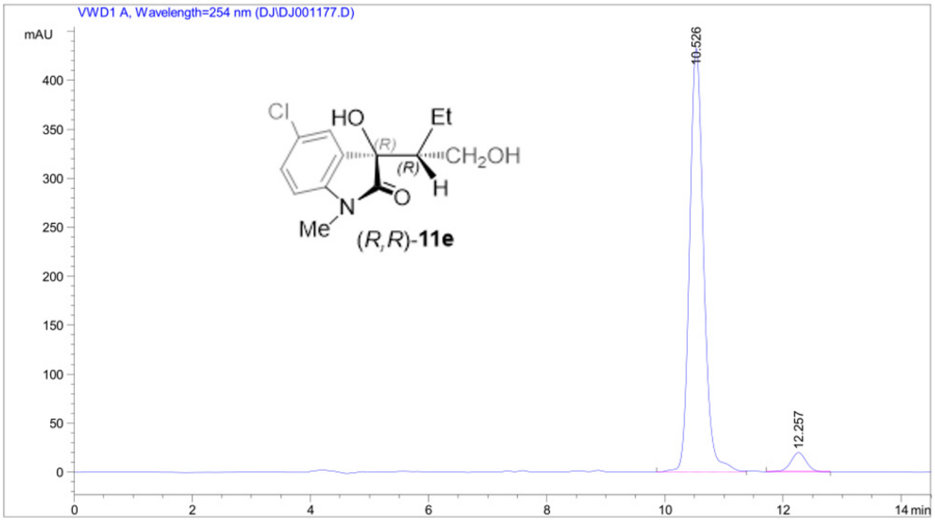

Peak #	RetTime [min]	Type	Width [min]	Area mAU *s	Height [mAU]	Area %
1	10.526	VB	0.2552	7151.52979	433.12177	95.0000
2	12.257	VB	0.2882	376.39508	19.85277	5.0000

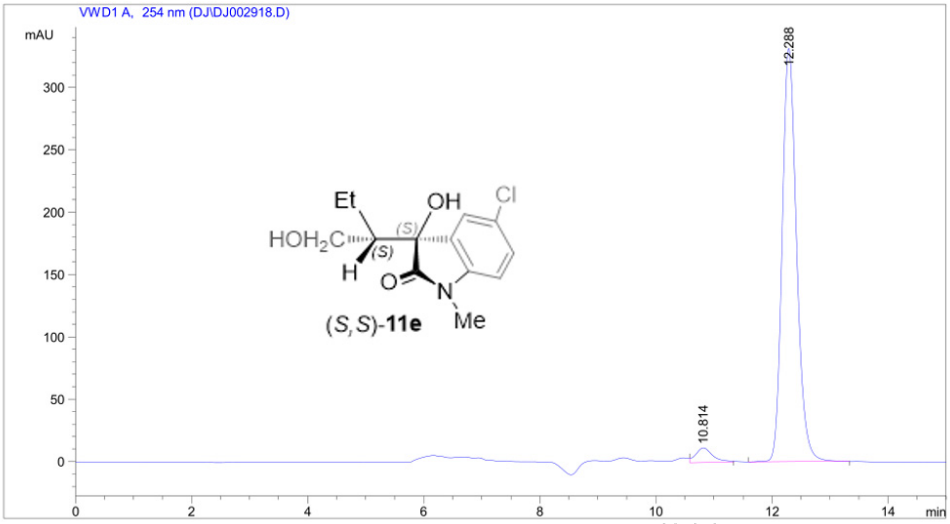

Peak #	RetTime [min]	Type	Width [min]	Area mAU *s	Height [mAU]	Area %
1	10.814	MM	0.3410	245.29152	11.98850	3.9674
2	12.288	VB	0.2715	5937.35010	333.85883	96.0326

Supplementary Figure 109. HPLC Spectrum for **11e.**

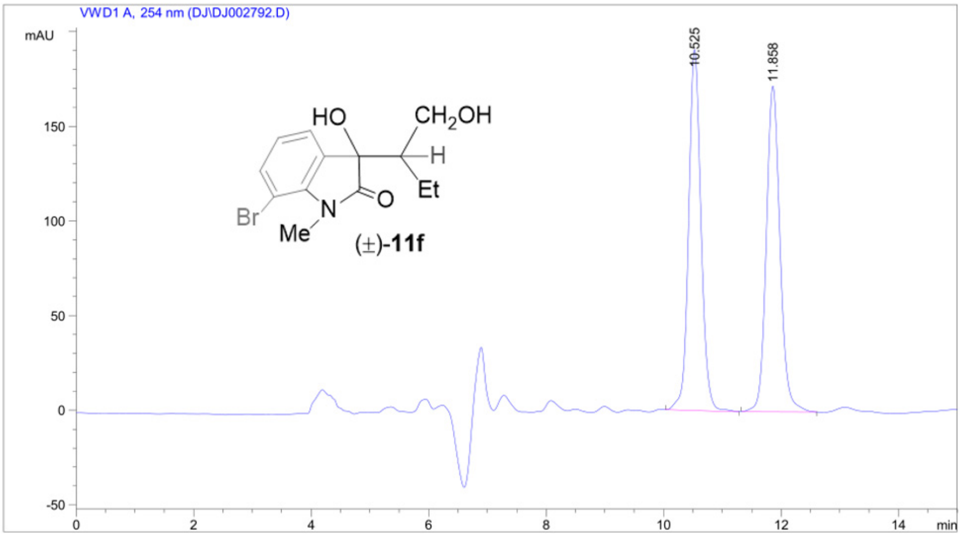

Peak #	RetTime [min]	Type	Width [min]	Area mAU *s	Height [mAU]	Area %
1	10.525	VB	0.2270	2842.87646	191.60237	50.3821
2	11.858	BB	0.2494	2799.76099	172.20282	49.6179

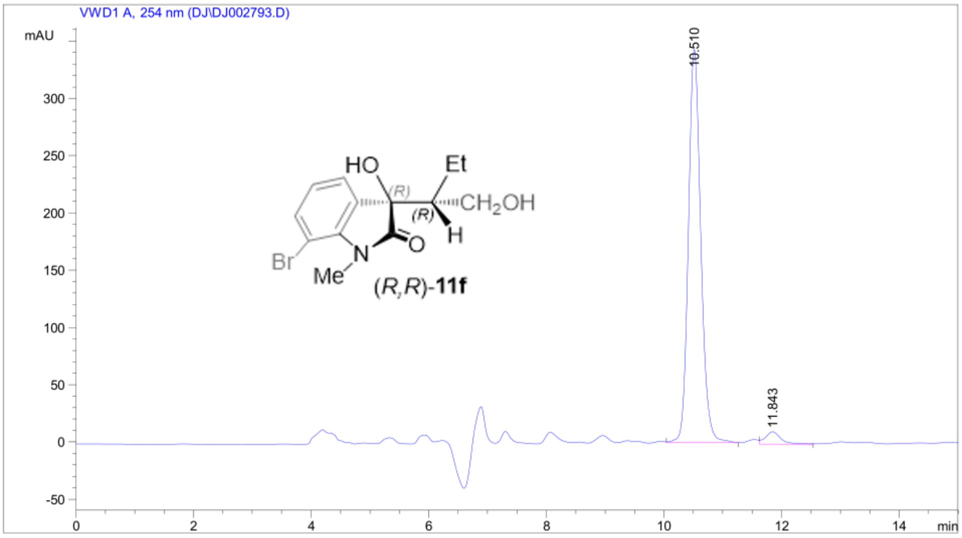

Peak #	RetTime [min]	Type	Width [min]	Area mAU *s	Height [mAU]	Area %
1	10.510	VB	0.2284	5108.38525	344.41458	96.5220
2	11.843	VB	0.2605	184.07402	10.54284	3.4780

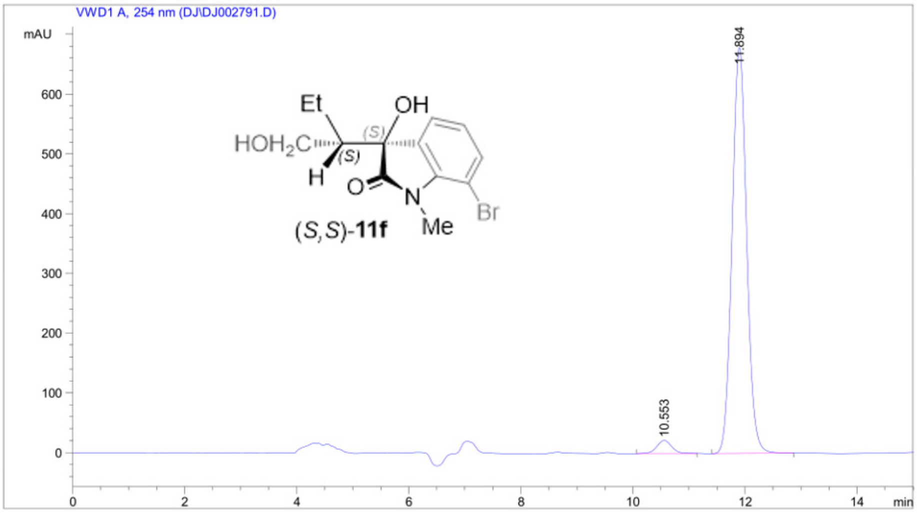

Peak #	RetTime [min]	Type	Width [min]	Area mAU *s	Height [mAU]	Area %
1	10.553	BB	0.2899	416.01483	21.91831	3.2832
2	11.894	BB	0.2788	1.22549e4	679.58081	96.7168

Supplementary Figure 110. HPLC Spectrum for **11f.**

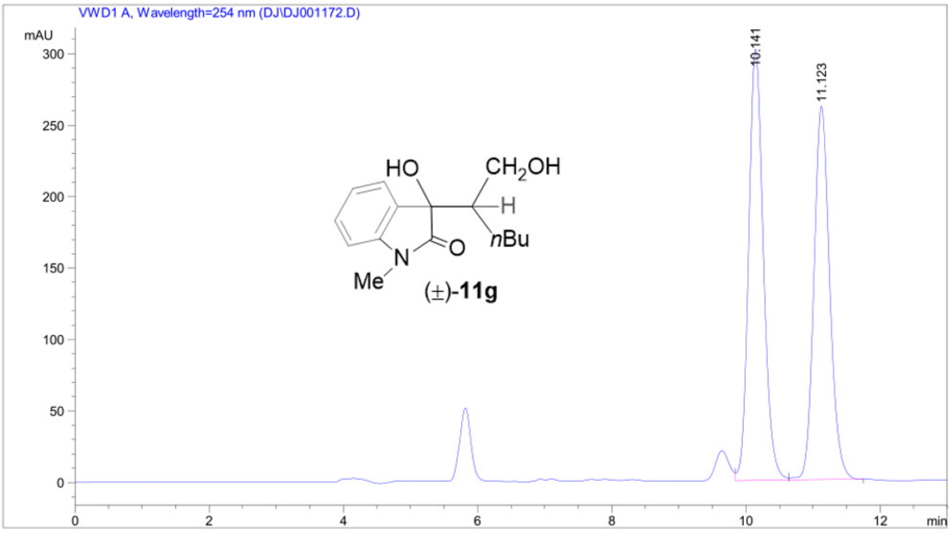

Peak #	RetTime [min]	Type	Width [min]	Area mAU *s	Height [mAU]	Area %
1	10.141	VV	0.2357	4591.41016	301.84912	51.6893
2	11.123	VB	0.2527	4291.29102	261.34805	48.3107

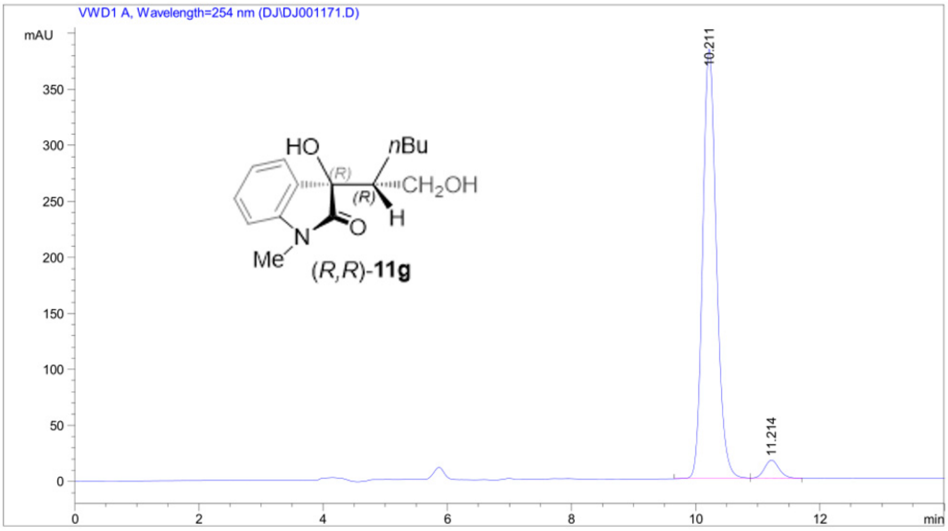

Peak #	RetTime [min]	Type	Width [min]	Area mAU *s	Height [mAU]	Area %
1	10.211	BV	0.2356	5878.67676	383.56653	95.5697
2	11.214	VB	0.2549	272.51672	16.40599	4.4303

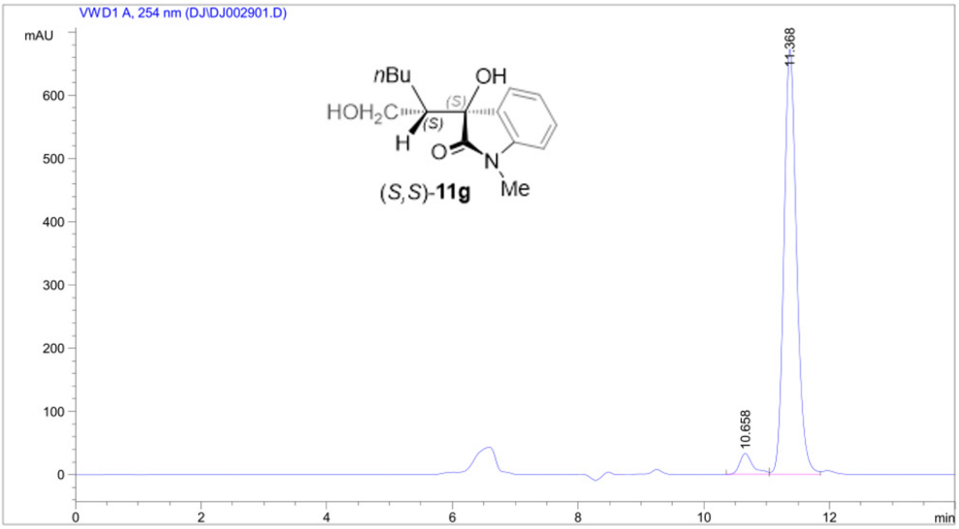

Peak #	RetTime [min]	Type	Width [min]	Area mAU *s	Height [mAU]	Area %
1	10.658	VV	0.2207	493.41010	33.05902	4.9861
2	11.368	VV	0.2155	9402.34863	672.77734	95.0139

Supplementary Figure 111. HPLC Spectrum for **11g.**

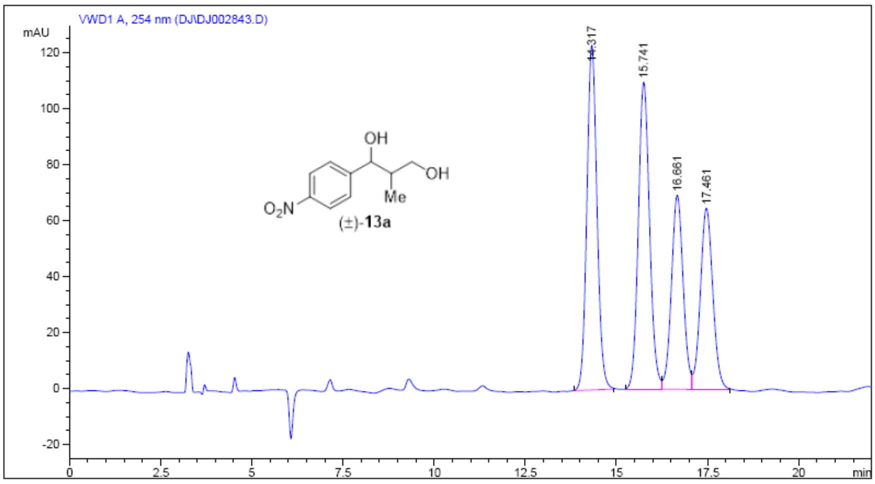

Peak #	RetTime [min]	Type	Width [min]	Area mAU *s	Height [mAU]	Area %
1	14.317	BB	0.2986	2383.03247	123.11797	30.4086
2	15.741	BV	0.3323	2358.94141	109.75499	30.1012
3	16.661	VV	0.3478	1557.30359	69.37752	19.8719
4	17.461	VB	0.3656	1537.42407	64.79512	19.6183

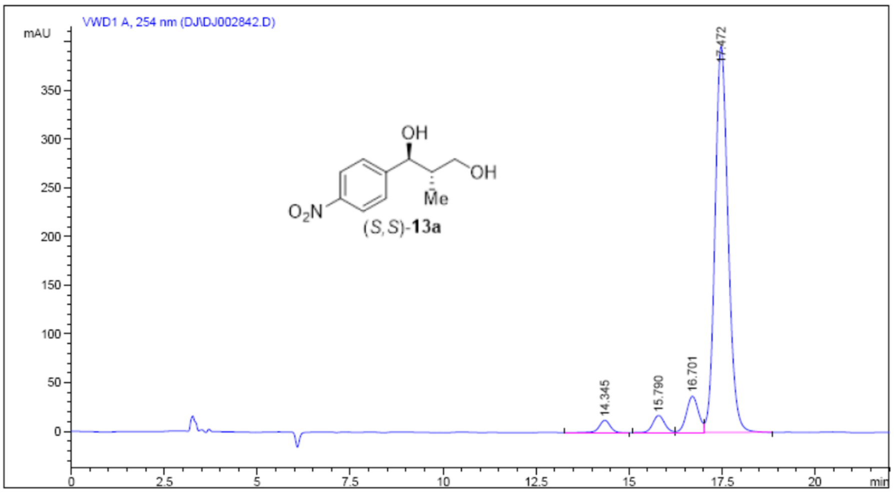

Peak #	RetTime [min]	Type	Width [min]	Area mAU *s	Height [mAU]	Area %
1	14.345	VB	0.3218	268.83469	12.74700	2.4023
2	15.790	BV	0.3457	393.79547	17.58303	3.5190
3	16.701	VV	0.3498	836.77313	37.20290	7.4774
4	17.472	VB	0.3757	9691.26953	396.05817	86.6013

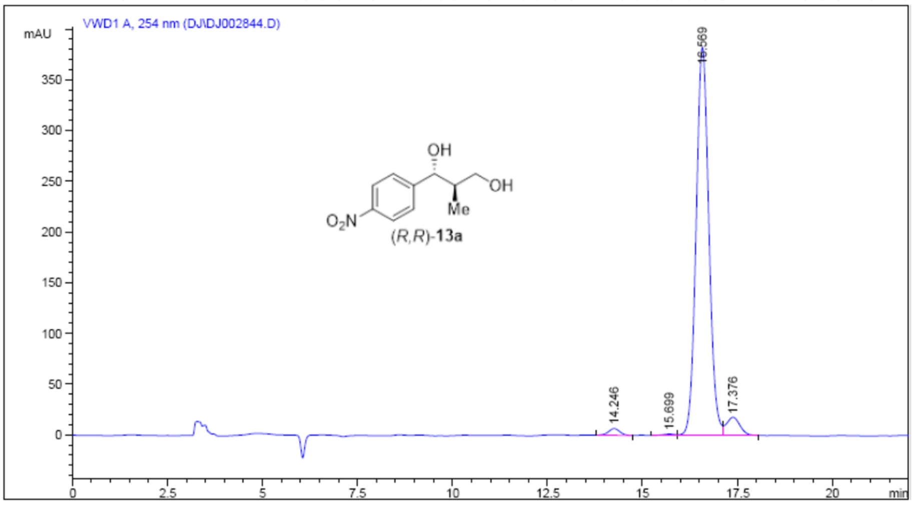

Peak #	RetTime [min]	Type	Width [min]	Area mAU *s	Height [mAU]	Area %
1	14.246	BV	0.3218	139.65439	6.69910	1.4623
2	15.699	VV	0.3331	34.14535	1.47263	0.3575
3	16.569	VV	0.3624	8930.46484	382.80707	93.5126
4	17.376	VB	0.3774	445.74835	18.01742	4.6675

Supplementary Figure 112. HPLC Spectrum for **13a.**

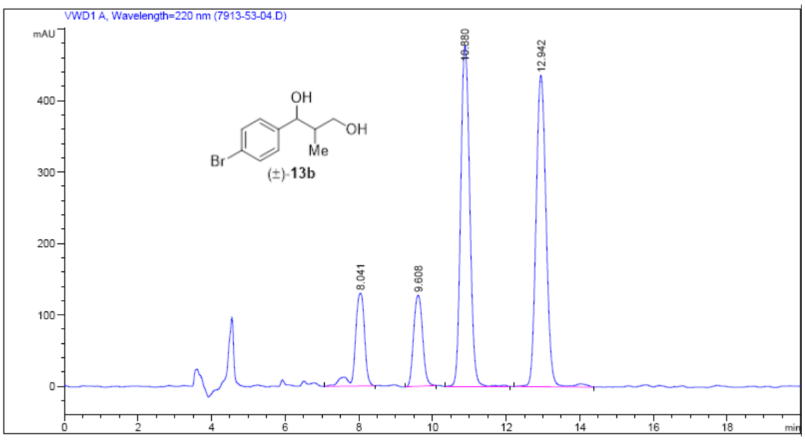

Peak #	RetTime [min]	Type	Width [min]	Area [mAU*s]	Height [mAU]	Area %
1	8.041	VB R	0.2635	2354.04297	130.00317	11.2487
2	9.608	BB	0.2729	2154.84180	127.65204	10.2968
3	10.880	BV R	0.2721	8152.36816	476.76563	38.9557
4	12.942	BV R	0.2960	8266.05078	435.91464	39.4989

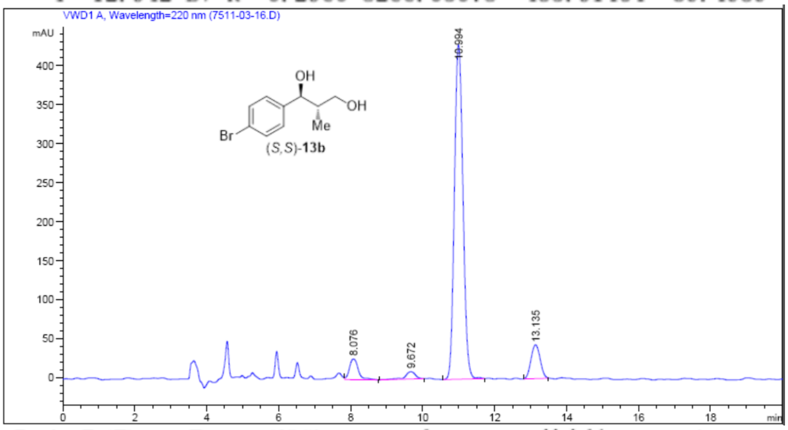

Peak #	RetTime [min]	Type	Width [min]	Area [mAU*s]	Height [mAU]	Area %
1	8.076	VB	0.2672	449.05469	26.30469	5.2919
2	9.672	VB R	0.3071	184.26872	9.30083	2.1715
3	10.994	BV R	0.2616	7085.44238	429.24811	83.4985
4	13.135	MM	0.2951	766.94543	43.32264	9.0381

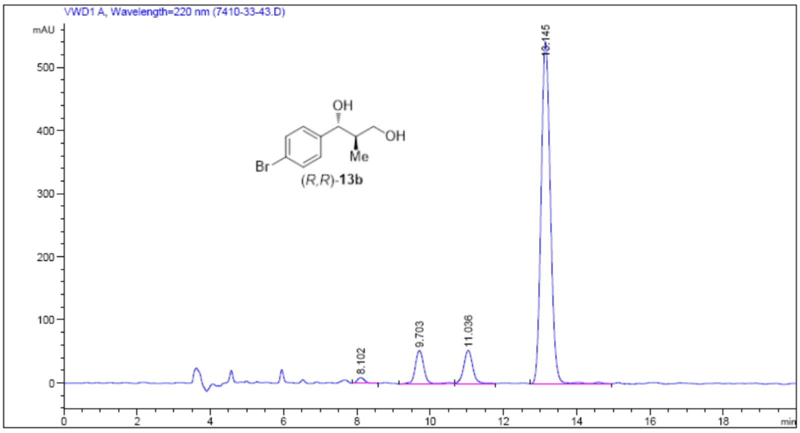

Peak #	RetTime [min]	Type	Width [min]	Area [mAU*s]	Height [mAU]	Area %
1	8.102	VV R	0.2251	122.26236	8.69041	1.0701
2	9.703	BV R	0.2596	874.35303	52.68496	7.6528
3	11.036	VV R	0.2618	903.73059	53.03883	7.9099
4	13.145	BV R	0.2719	9524.95117	542.26892	83.3672

Supplementary Figure 113. HPLC Spectrum for **13b.**

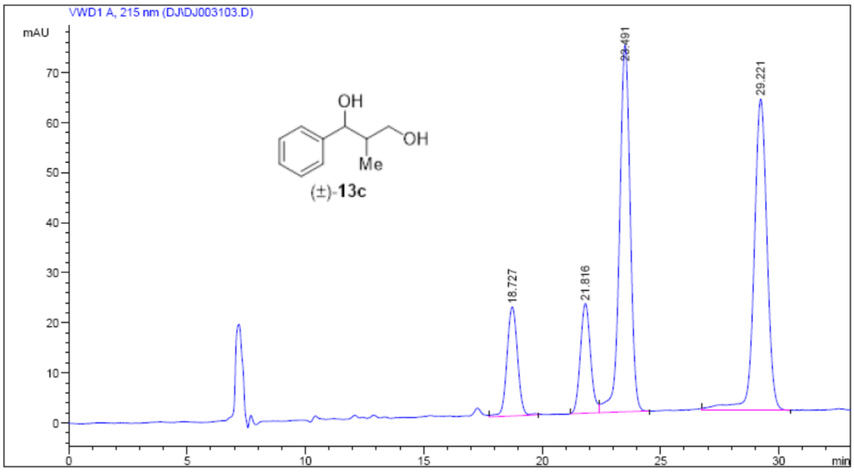

Peak #	RetTime [min]	Type	Width [min]	Area mAU *s	Height [mAU]	Area %
1	18.727	VB	0.4941	684.13593	21.80837	11.4118
2	21.816	BV	0.4711	655.86230	21.94032	10.9401
3	23.491	VB	0.4894	2303.59814	73.21680	38.4253
4	29.221	BB	0.5875	2351.41089	62.08074	39.2228

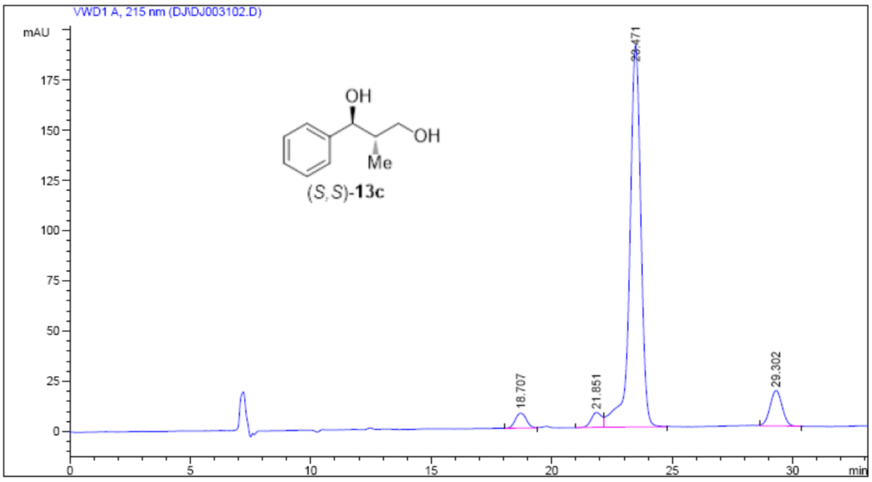

Peak #	RetTime [min]	Type	Width [min]	Area mAU *s	Height [mAU]	Area %
1	18.707	BV	0.5203	244.18150	7.48711	3.3647
2	21.851	BV	0.4695	236.14372	7.55455	3.2540
3	23.471	VB	0.5009	6165.26758	189.99194	84.9554
4	29.302	BB	0.5384	611.47198	17.64017	8.4259

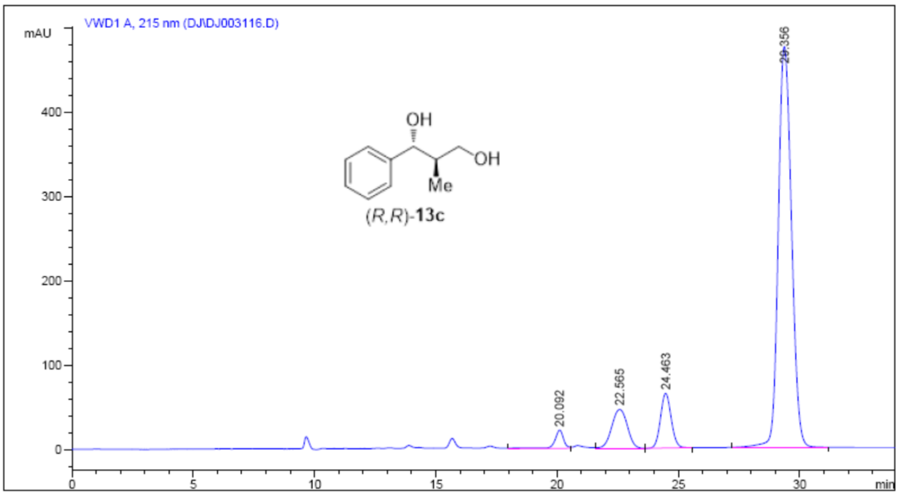

Peak #	RetTime [min]	Type	Width [min]	Area mAU *s	Height [mAU]	Area %
1	20.092	VV	0.4098	620.27197	22.04753	2.7297
2	22.565	VV	0.7160	2074.78979	46.21357	9.1309
3	24.463	VB	0.4822	2002.08337	64.89220	8.8110
4	29.356	BB	0.6001	1.80255e4	474.88019	79.3284

Supplementary Figure 114. HPLC Spectrum for **13c.**

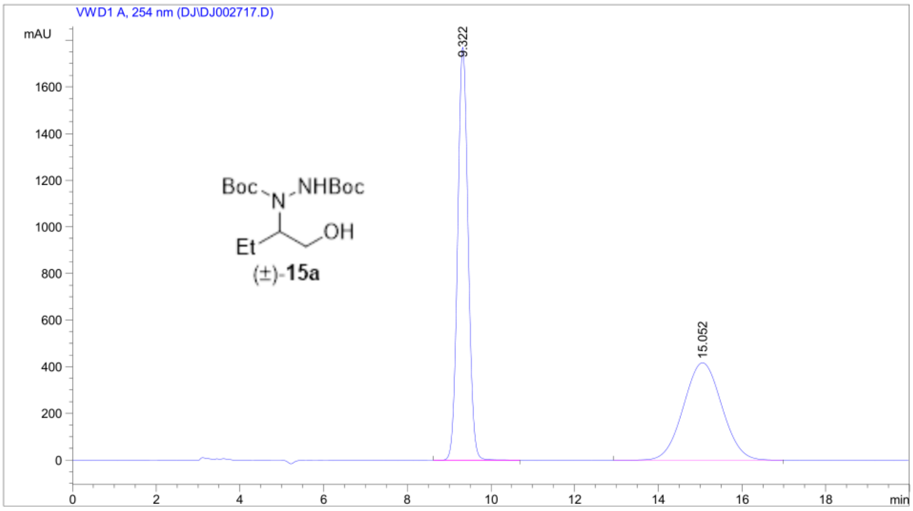

Peak #	RetTime [min]	Type	Width [min]	Area mAU *s	Height [mAU]	Area %
1	9.322	VV	0.2713	3.10694e4	1773.69714	53.6868
2	15.052	BB	1.0001	2.68021e4	418.73578	46.3132

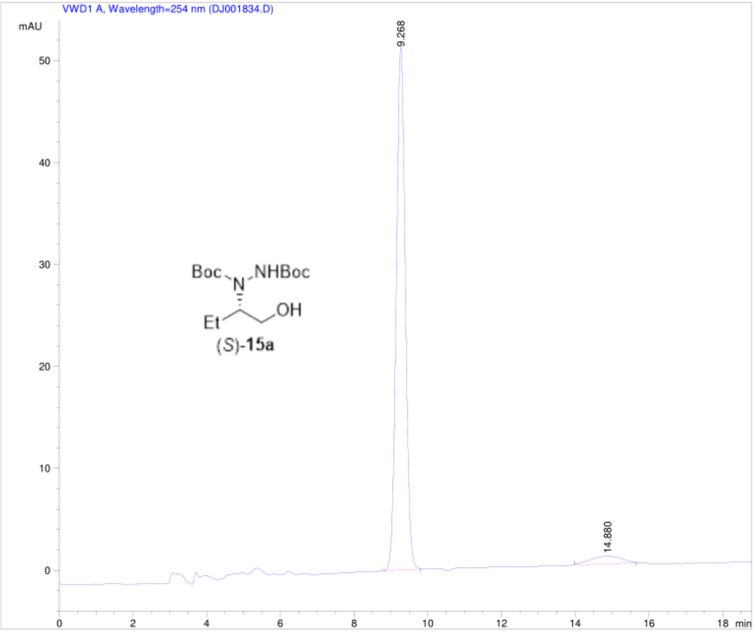

Peak #	RetTime [min]	Type	Width [min]	Area mAU *s	Height [mAU]	Area %
1	9.268	BB	0.2650	870.98987	51.31317	95.2309
2	14.880	MM	0.6973	43.61899	7.44600e-1	4.7691

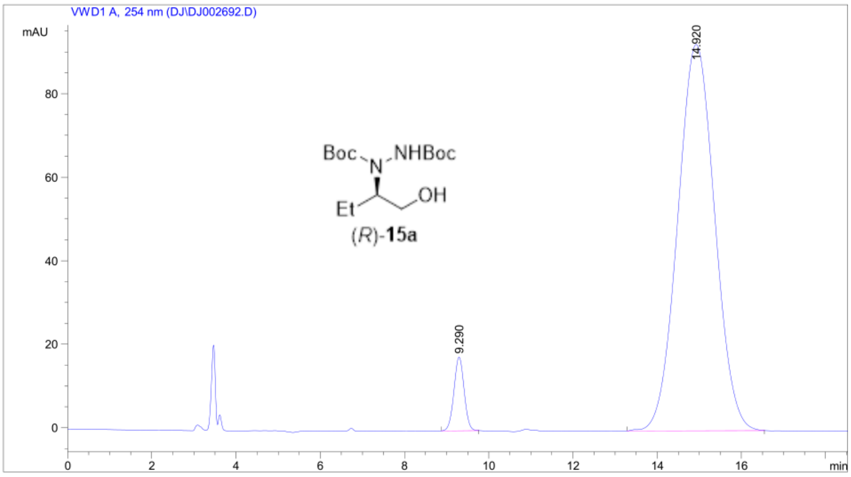

Peak #	RetTime [min]	Type	Width [min]	Area mAU *s	Height [mAU]	Area %
1	9.290	BB	0.2631	299.32608	17.67159	5.0321
2	14.920	BB	0.9521	5649.05957	92.57800	94.9679

Supplementary Figure 115. HPLC Spectrum for **15a.**

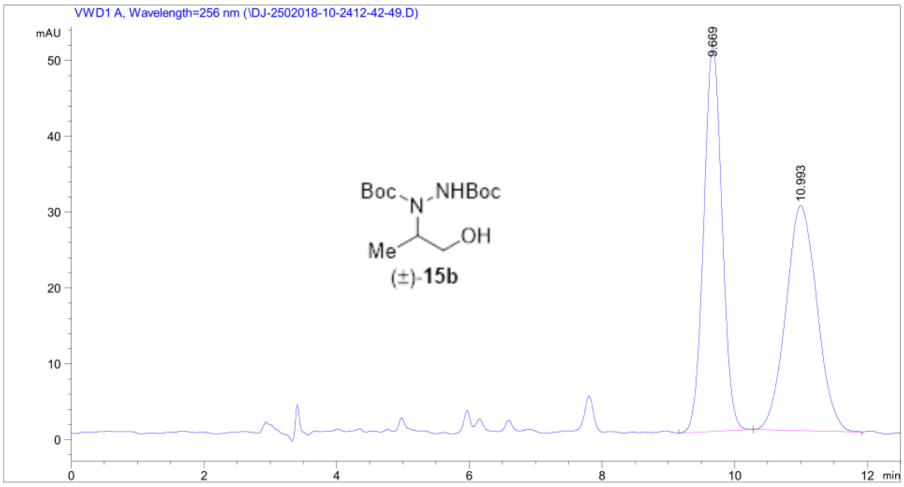

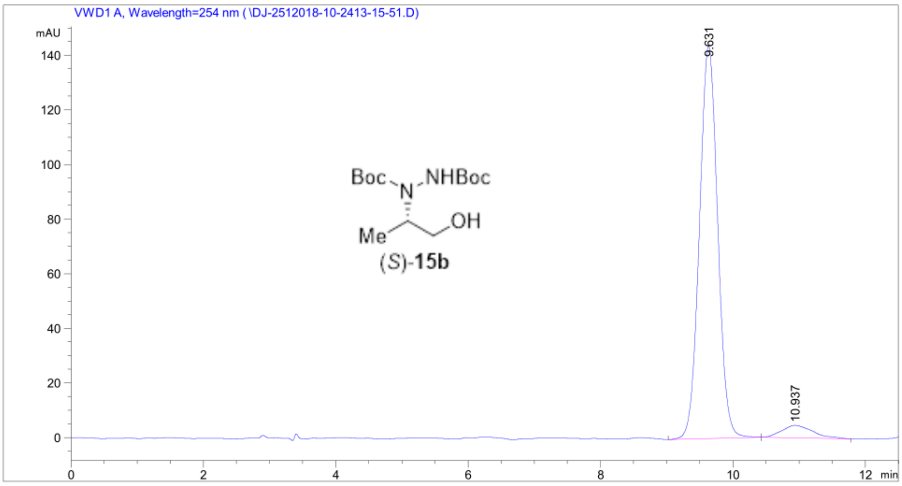

Peak #	RetTime [min]	Type	Width [min]	Area [mAU*s]	Height [mAU]	Area %
1	9.631	BB	0.3012	2749.32153	143.73305	94.8317
2	10.937	BB	0.4629	149.83690	4.54190	5.1683

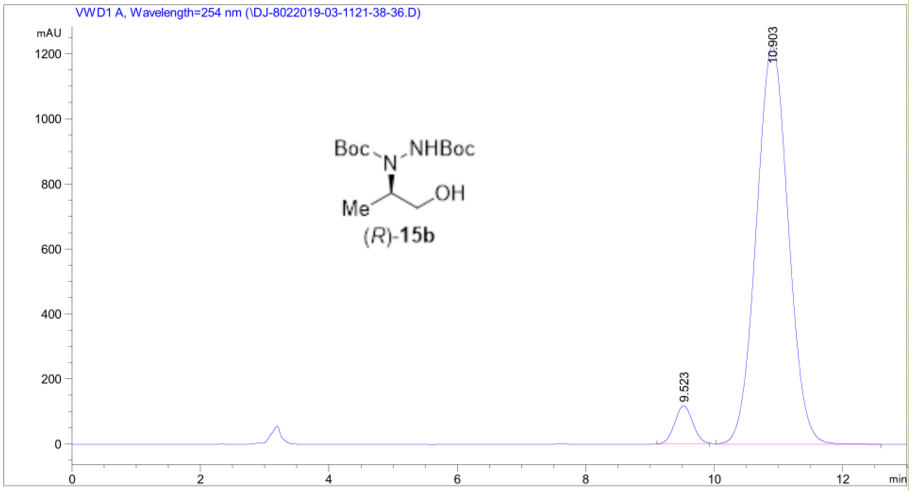

Peak #	RetTime [min]	Type	Width [min]	Area [mAU*s]	Height [mAU]	Area %
1	9.523	MM	0.3262	2284.71826	116.72712	5.1216
2	10.903	VB	0.5395	4.23244e4	1223.90308	94.8784

Supplementary Figure 116. HPLC Spectrum for **15b.**

Supplementary References

1 Trost, B. M., Hung, C.-I. (Joey) & Scharf, M. J. Direct Catalytic Asymmetric Vinylogous Additions of *α,β*- and *β,γ*-Butenolides to Polyfluorinated Alkynyl Ketimines. *Angew. Chem. Int. Ed.* **57**, 11408 (2018).

2 Dai, J. et al. Chiral Primary Amine Catalysis for Asymmetric Mannich Reactions of Aldehydes with Ketimines: Stereoselectivity and Reactivity. *Angew. Chem. Int. Ed.* **56**, 12697−12701 (2017).

3 Frisch, M. J. et al. *Gaussian 09, Revision D.01*. Gaussian, Inc., Wallingford CT (2013).

4 Becke, A. D. Density-functional thermochemistry. III. The role of exact exchange. *J. Chem. Phys.* **98**, 5648-5652 (1993).

5 Lee, C.; Yang, W. & Parr, R. G. Development of the Colle-Salvetti correlation-energy formula into a functional of the electron density. *Phys. Rev. B* **37**, 785-789 (1988).

6 Grimme, S. Accurate description of van der Waals complexes by density functional theory including empirical corrections. *J. Comput. Chem.* **25**, 1463-1474 (2004).

7 Grimme, S., Ehrlich, S. & Goerigk, L. Effect of the damping function in dispersion corrected density functional theory. *J. Comput. Chem.* **32**, 1456-1465 (2011).

8 Zhao, Y. & Truhlar, D. G.The M06 suite of density functionals for main group thermochemistry, thermochemical kinetics, noncovalent interactions, excited states, and transition elements: two new functionals and systematic testing of four M06-class functionals and 12 other functionals. *Theor. Chem. Acc.* **120**, 215-241 (2007).

9 Marenich, A. V., Cramer, C. J. & Truhlar, D. G. Universal Solvation Model Based on Solute Electron Density and on a Continuum Model of the Solvent Defined by the Bulk Dielectric Constant and Atomic Surface Tensions. *J. Phys. Chem. B* **113**, 6378-6396 (2009).

10 Peverati, R. & Truhlar, D. G. Improving the Accuracy of Hybrid Meta-GGA Density Functionals by Range Separation. *J. Phys. Chem. Lett.* **2**, 2810-2817 (2011).

11 Chai, J. D. & Head-Gordon, M. Long-range corrected hybrid density functionals with damped atom–atom dispersion corrections. *Phys. Chem. Chem. Phys.* **10**, 6615-

6620 (2008).

12 Chen, S. & Houk, K. N. Origins of Stereoselectivity in Mannich Reactions Catalyzed by Chiral Vicinal Diamines. *J. Org. Chem.* **83**, 3171-3176 (2018).
